# Supplementary material for: HIVprotI: an integrated web based platform for prediction and design of HIV proteins inhibitors
Source: J Cheminform. 2018 Mar 9;10:12. doi: 10.1186/s13321-018-0266-y (PMC5845081; doi:10.1186/s13321-018-0266-y)
Supplement: Supplementary file 2 — Additional file 2. Source code of HIVProtI web server. [file 13321_2018_266_MOESM2_ESM.zip › HIVprotI_Source-code/hivprotI/csv2tab/table.html]

php require\_once("head.php");?


---

  

| Details | SMILES | Target Protein |
| --- | --- | --- |
| Details | SMILES | Target Protein |
';| CHEMBL174039 | OC(CNNC(=O)c1ccccc1O)COc2ccccc2.Oc3ccccc3C(=O)NNC(=O)c4ccccc4O | Integrase |
| CHEMBL414850 | O\C(=N/Cc1ccc(F)cc1)\c2nc(N3CCCCS3(=O)=O)c4cccnc4c2O | Integrase |
| CHEMBL173854 | OCNC(=O)NCOCN1C(=O)N(CO)C(=O)N(CO)C1=O | Integrase |
| CHEMBL198001 | OC(=O)C(=O)\C=C(/O)\c1cn(Cc2ccc(F)cc2)c3ccccc13 | Integrase |
| CHEMBL196391 | COc1ccc2c(c1)c(cn2Cc3ccc(F)cc3)\C(=C\C(=O)C(=O)O)\O | Integrase |
| CHEMBL174172 | ONC(=O)\C(=N/O)\CC(=O)c1ccc(O)cc1O | Integrase |
| CHEMBL369267 | Oc1ccc(\C=N\NC(=O)N\N=C\c2ccc(O)cc2O)c(O)c1 | Integrase |
| CHEMBL212412 | COc1c2c(C(=O)N(Cc3ccc(F)cc3)C24CC4)c(O)c5ncccc15 | Integrase |
| CHEMBL210234 | CN(C(=O)C(=O)N1CCCCC1)c2nc(C(=O)NCc3ccc(F)cc3)c(O)c4ncccc24 | Integrase |
| CHEMBL212213 | COc1c2CN(Cc3ccc(F)cc3)C(=O)c2c(O)c4ncc(F)cc14 | Integrase |
| CHEMBL204656 | COc1cc2N(C=C(C(=O)O)C(=O)c2cc1Cc3cccc(Cl)c3F)[C@H](CO)C(C)C | Integrase |
| CHEMBL206604 | CC(C)[C@@H](CO)N1C=C(C(=O)O)C(=O)c2cc(Cc3cccc(Cl)c3F)ccc12 | Integrase |
| CHEMBL3288833 | COC(=O)[C@H](NC1=C(C(=O)NCc2ccc(F)cc2F)C(=O)N(O)c3ncccc13)c4ccccc4 | Integrase |
| CHEMBL3288834 | COC(=O)[C@H](CO)NC1=C(C(=O)NCc2ccc(F)cc2F)C(=O)N(O)c3ncccc13 | Integrase |
| CHEMBL198160 | CN1C(CC(=O)N(C)C1=O)c2nc(C(=O)NCc3ccc(F)cc3)c(O)c4ncccc24 | Integrase |
| CHEMBL203443 | COc1cc2N(CCO)C=C(C(=O)O)C(=O)c2cc1Cc3cccc(Cl)c3F | Integrase |
| CHEMBL3286416 | ON1C(=C(C(=O)Nc2ccccc2)c3ccc(cc3C1=O)[N+](=O)[O-])O | Integrase |
| CHEMBL3288265 | ON1C(=C(C(=O)NCc2ccccc2)c3ccc(cc3C1=O)[N+](=O)[O-])O | Integrase |
| CHEMBL32865 | Oc1c(ncc2cccnc12)C(=O)c3cc(CN4CCCS4(=O)=O)cc(Cc5ccccc5)c3 | Integrase |
| CHEMBL3288832 | COC(=O)[C@@H](NC1=C(C(=O)NCc2ccc(F)cc2F)C(=O)N(O)c3ncccc13)c4ccccc4 | Integrase |
| CHEMBL19464 | OC(=O)C(=O)CC(=O)c1cccc(Cc2ccccc2)c1 | Integrase |
| CHEMBL211028 | OC(=O)\C(=C\C(=O)CCc1ccc(Cl)c(Cl)c1)\O | Integrase |
| CHEMBL3288827 | OCCNC1=C(C(=O)NCc2ccc(F)cc2F)C(=O)N(O)c3ncccc13 | Integrase |
| CHEMBL2079773 | [Mg+2].[O-]\C(=N/Cc1ccc(F)cc1)\c2nc(N3CCCCS3(=O)=O)c4cccnc4c2[O-] | Integrase |
| CHEMBL216874 | Oc1nc(nc(C(=O)NCc2ccc(F)cc2)c1O)c3cccs3 | Integrase |
| CHEMBL3288268 | COc1ccc(CNC(=O)C2=C(O)N(O)C(=O)c3cc(ccc23)[N+](=O)[O-])cc1 | Integrase |
| CHEMBL371007 | Oc1c(nc(C2CC(=O)NC(=O)N2)c3cccnc13)C(=O)NCc4ccc(F)cc4 | Integrase |
| CHEMBL3288273 | COc1ccc2C(=C(O)N(O)C(=O)c2c1)C(=O)NCc3ccc(F)cc3 | Integrase |
| CHEMBL254316 | CN1C(=O)C(=C(N=C1C(C)(C)NC(=O)c2oc(C)nn2)C(=O)NCc3ccc(F)cc3)O | Integrase |
| CHEMBL363170 | OC(=O)C(=O)\C=C(/O)\c1cn(Cc2ccc(F)cc2)c3ccc(Cl)cc13 | Integrase |
| CHEMBL193759 | OC(=O)C(=O)\C=C(/O)\c1cccc(Cc2ccccc2)c1 | Integrase |
| CHEMBL379658 | CCN1CCN(C(=O)C1=O)c2nc(C(=O)NCc3ccc(F)cc3)c(O)c4ncccc24 | Integrase |
| CHEMBL194735 | OC(=O)C(=O)\C=C(/O)\c1cn(Cc2ccccc2)c3ccc(Cl)cc13 | Integrase |
| CHEMBL377501 | OC(=O)C(=O)\C=C(/O)\C1=CN(Cc2ccc(F)cc2)c3ccc(cc3C1=O)\C(=C\C(=O)C(=O)O)\O | Integrase |
| CHEMBL425924 | CN1C(CC(=O)NC1=O)c2nc(C(=O)NCc3ccc(F)cc3)c(O)c4ncccc24 | Integrase |
| CHEMBL382965 | CN1C(=O)CC(NC1=O)c2nc(C(=O)NCc3ccc(F)cc3)c(O)c4ncccc24 | Integrase |
| CHEMBL3288835 | COC(=O)[C@@H](CO)NC1=C(C(=O)NCc2ccc(F)cc2F)C(=O)N(O)c3ncccc13 | Integrase |
| CHEMBL378029 | CNC(=O)C(=O)N(C)c1nc(C(=O)NCc2ccc(F)cc2)c(O)c3ncccc13 | Integrase |
| CHEMBL3288825 | CC(C)NC1=C(C(=O)NCc2ccc(F)cc2F)C(=O)N(O)c3ncccc13 | Integrase |
| CHEMBL196689 | COc1ccc2c(c1)c(cn2Cc3ccccc3)\C(=C\C(=O)C(=O)O)\O | Integrase |
| CHEMBL3288830 | COC(=O)[C@H](C)NC1=C(C(=O)NCc2ccc(F)cc2F)C(=O)N(O)c3ncccc13 | Integrase |
| CHEMBL3288817 | ON1C(=O)C(=C(Nc2ccccc2)c3cccnc13)C(=O)NCc4ccc(F)cc4F | Integrase |
| CHEMBL3288837 | NC1=C(C(=O)NCc2ccc(F)cc2F)C(=O)N(O)c3ncccc13 | Integrase |
| CHEMBL368084 | O\C(=C/C(=O)c1nc[nH]n1)\c2oc(Cc3ccc(F)cc3)cc2 | Integrase |
| CHEMBL386710 | CN1CCOCC1C2=NC(=C(O)C(=O)N2C)C(=O)NCc3ccc(F)cc3 | Integrase |
| CHEMBL50605 | OC(=O)C(=O)CC(=O)c1cccn1Cc2ccc(F)cc2 | Integrase |
| CHEMBL186820 | OC(=O)C(=O)\C=C(/O)\c1cn(Cc2ccccc2)c3ccccc13 | Integrase |
| CHEMBL3288281 | ON1C(=O)C(C(=O)NCc2ccc(F)cc2)c3ccc(cc3C1=O)C(F)(F)F | Integrase |
| CHEMBL210233 | CN(C(=O)C(=O)N)c1nc(C(=O)NCc2ccc(F)cc2)c(O)c3ncccc13 | Integrase |
| CHEMBL3288829 | COC(=O)CNC1=C(C(=O)NCc2ccc(F)cc2F)C(=O)N(O)c3ncccc13 | Integrase |
| CHEMBL172072 | C[C@@H]1C(Oc2cc(O)c(C3OC(CO)C(O)C(O)C3O)c(O)c2C1=O)c4ccc(O)c(O)c4 | Integrase |
| CHEMBL3288824 | CCCCCNC1=C(C(=O)NCc2ccc(F)cc2F)C(=O)N(O)c3ncccc13 | Integrase |
| CHEMBL379664 | OCCN1C=C(C(=O)O)C(=O)c2cc(Cc3cccc(Cl)c3F)ccc12 | Integrase |
| CHEMBL377759 | CN(C(=O)C)c1nc(C(=O)NCc2ccc(F)cc2)c(O)c3ncccc13 | Integrase |
| CHEMBL209872 | CC(=O)N1CCN(CC1)c2nc(C(=O)NCc3ccc(F)cc3)c(O)c4ncccc24 | Integrase |
| CHEMBL3288828 | CC(=O)OCCNC1=C(C(=O)NCc2ccc(F)cc2F)C(=O)N(O)c3ncccc13 | Integrase |
| CHEMBL3288818 | CNC1=C(C(=O)NCc2ccc(F)cc2F)C(=O)N(O)c3ncccc13 | Integrase |
| CHEMBL3288831 | COC(=O)[C@@H](C)NC1=C(C(=O)NCc2ccc(F)cc2F)C(=O)N(O)c3ncccc13 | Integrase |
| CHEMBL3259893 | CO[C@H](C(=O)O)c1c(C)nc2ccc(Br)cc2c1c3ccc(Cl)cc3 | Integrase |
| CHEMBL197001 | CCOC(=O)C(=O)\C=C(/O)\c1cn(Cc2ccccc2)c3ccc(Cl)cc13 | Integrase |
| CHEMBL3288274 | ON1C(=C(C(=O)NCc2ccc(F)cc2)c3cc(ccc3C1=O)[N+](=O)[O-])O | Integrase |
| CHEMBL3288266 | ON1C(=C(C(=O)Nc2ccc(F)cc2)c3ccc(cc3C1=O)[N+](=O)[O-])O | Integrase |
| CHEMBL199145 | Oc1c(ncc2cccnc12)C(=O)NCc3ccc(F)cc3 | Integrase |
| CHEMBL212982 | CN(C)S(=O)(=O)Oc1c2CN(Cc3ccc(F)cc3)C(=O)c2c(O)c4nccnc14 | Integrase |
| CHEMBL367001 | CCC\C=C\C=C\[C@@H]1C[C@@H](O)[C@@H](O)C(=O)[C@H]1CO | Integrase |
| CHEMBL212000 | COc1c2C(O)N(Cc3ccc(F)cc3)C(=O)c2c(O)c4ncccc14 | Integrase |
| CHEMBL209854 | Oc1c2C(=O)N(Cc3ccc(F)cc3)Cc2c(C(=O)N4CCOCC4)c5cccnc15 | Integrase |
| CHEMBL3288826 | NCCNC1=C(C(=O)NCc2ccc(F)cc2F)C(=O)N(O)c3ncccc13 | Integrase |
| CHEMBL36070 | Oc1c(ncc2cccnc12)C(=O)c3cccc(Cc4ccccc4)c3 | Integrase |
| CHEMBL209307 | CN(C(=O)C(=O)N1CCCC1)c2nc(C(=O)NCc3ccc(F)cc3)c(O)c4ncccc24 | Integrase |
| CHEMBL441683 | CN1CCN(CC1)c2nc(C(=O)NCc3ccc(F)cc3)c(O)c4ncccc24 | Integrase |
| CHEMBL426686 | CN(C)C(=O)C(=O)N(C)c1nc(C(=O)NCc2ccc(F)cc2)c(O)c3ncccc13 | Integrase |
| CHEMBL380588 | NC(=O)C(=O)Nc1nc(C(=O)NCc2ccc(F)cc2)c(O)c3ncccc13 | Integrase |
| CHEMBL3259891 | COC(C(=O)O)c1c(C)nc2ccc(Br)cc2c1c3ccc(Cl)cc3 | Integrase |
| CHEMBL174455 | Oc1ccccc1C(=O)NC(=O)CC(O)(c2ccccc2)c3ccc(Cl)cc3 | Integrase |
| CHEMBL3219143 | COc1cc(F)ccc1Cn2ccc3c4CCN(O)C(=O)c4ncc23 | Integrase |
| CHEMBL270717 | OC(=O)C1=CNc2ccc(Cc3cccc(Cl)c3F)cc2C1=O | Integrase |
| CHEMBL421353 | OC(=O)C(=O)\C=C(/O)\c1cccn1Cc2ccc(F)cc2 | Integrase |
| CHEMBL209440 | COc1c2CN(Cc3ccc(F)cc3)C(=O)c2c(O)c4ncccc14 | Integrase |
| CHEMBL3288822 | ON1C(=O)C(=C(NCCc2ccccc2)c3cccnc13)C(=O)NCc4ccc(F)cc4F | Integrase |
| CHEMBL142072 | Oc1c(ccc2cccnc12)C(=O)c3cc(Cc4ccccc4)ccn3 | Integrase |
| CHEMBL386515 | CN(C)C(C)(C)c1nc(O)c(O)c(n1)C(=O)NCc2ccc(F)cc2 | Integrase |
| CHEMBL2068731 | [Mg+2].CC(C)Oc1ccc(Cc2ccccc2)cc1\C(=C\C(=O)C(=O)[O-])\[O-] | Integrase |
| CHEMBL217020 | Oc1nc(Cc2ccccc2)nc(C(=O)NCc3ccc(F)cc3)c1O | Integrase |
| CHEMBL3238134 | OC(=O)\C(=C\C(=O)C1=CN(Cc2ccc(F)cc2)c3cc(ccc3C1=O)N4CCN(CCCCl)CC4)\O | Integrase |
| CHEMBL172756 | OCCOC(=O)CCCCC(=O)c1ccc(O)cc1O | Integrase |
| CHEMBL208776 | CN(C)C(=O)CN(C)c1nc(C(=O)NCc2ccc(F)cc2)c(O)c3ncccc13 | Integrase |
| CHEMBL320261 | OC(=O)C(=O)\C=C(/O)\c1cc(OCc2ccccc2)cc(OCc3ccccc3)c1 | Integrase |
| CHEMBL385825 | COc1c2C(SCCC(=O)N3CCNCC3)N(Cc4ccc(F)cc4)C(=O)c2c(O)c5ncccc15.OC(=O)C(F)(F)F | Integrase |
| CHEMBL3288264 | CCCCCCNC(=O)C1=C(O)N(O)C(=O)c2cc(ccc12)[N+](=O)[O-] | Integrase |
| CHEMBL174243 | COc1cc2OC(=CC(=O)c2c(O)c1C3OC(CO)C(O)C(O)C3O)c4ccc(O)cc4 | Integrase |
| CHEMBL3288263 | ON1C(=O)C(C(=O)NCc2ccc(F)cc2)c3ccc(NC(=O)Cc4cccs4)cc3C1=O | Integrase |
| CHEMBL217285 | CC1=NC(=C(O)C(=O)N1)C(=O)NCc2ccc(F)cc2 | Integrase |
| CHEMBL3288280 | ON1C(=O)C(C(=O)NCc2ccc(F)cc2)c3ccc(Br)cc3C1=O | Integrase |
| CHEMBL217566 | Oc1ncnc(C(=O)NCc2ccc(F)cc2)c1O | Integrase |
| CHEMBL3288267 | ON1C(=C(C(=O)NCCc2ccc(F)cc2)c3ccc(cc3C1=O)[N+](=O)[O-])O | Integrase |
| CHEMBL173618 | COc1cc(N)ccc1C(=O)NC(CCC(=O)O)C(=O)O | Integrase |
| CHEMBL384912 | COC1N(Cc2ccc(F)cc2)C(=O)c3c(O)c4ncccc4c(OC)c13 | Integrase |
| CHEMBL409132 | CC1C=C(NC(=O)C2=CC(=CC2C)NC(=O)C3=CC(=CC3C)N(C)C(=O)c4ccc(cc4)C(=O)N(C)C5=CC(C)C(=C5)C(=O)NC6=CC(C)C(=C6)C(=O)NC7=CC(C)C(=C7)C(=O)NCCCN(C)C)C=C1C(=O)NCCCN(C)C | Integrase |
| CHEMBL211634 | Oc1c2C(=O)N(Cc3ccc(F)cc3)Cc2cc4cccnc14 | Integrase |
| CHEMBL385951 | Oc1nc(nc(C(=O)NCc2ccccc2)c1O)c3ccccc3 | Integrase |
| CHEMBL365604 | CCOC(=O)C(=O)\C=C(/O)\c1cn(Cc2ccc(F)cc2)c3ccc(Cl)cc13 | Integrase |
| CHEMBL29660 | OC(=O)[C@@H](OC(=O)\C=C\c1ccc(O)c(O)c1)[C@H](OC(=O)\C=C\c2ccc(O)c(O)c2)C(=O)O | Integrase |
| CHEMBL3288282 | ON1C(=C(C(=O)NCc2ccc(F)cc2)c3ccc(cc3C1=O)C#N)O | Integrase |
| CHEMBL3259890 | CCCC(C(=O)O)c1c(C)nc2ccc(Br)cc2c1c3ccc(Cl)cc3 | Integrase |
| CHEMBL3288820 | ON1C(=O)C(=C(N2CCOCC2)c3cccnc13)C(=O)NCc4ccc(F)cc4F | Integrase |
| CHEMBL3238132 | OC(=O)\C(=C\C(=O)C1=CN(Cc2ccc(F)cc2)c3cc(ccc3C1=O)N4CCOCC4)\O | Integrase |
| CHEMBL3288271 | ON1C(=O)C(C(=O)NCc2ccc(F)cc2)c3ccc(NC(=O)c4ccccc4)cc3C1=O | Integrase |
| CHEMBL216317 | CC(N(C)C)c1nc(O)c(O)c(n1)C(=O)NCc2ccc(F)cc2 | Integrase |
| CHEMBL108760 | OC(=O)C(=O)\C=C(/O)\c1c[nH]c2ccc(Cl)cc12 | Integrase |
| CHEMBL212921 | COc1c2C(=O)N(Cc3ccc(F)cc3)C(=O)c2c(O)c4ncccc14 | Integrase |
| CHEMBL63592 | OC(=O)C(OC(=O)\C=C\c1ccc(O)c(O)c1)C(OC(=O)\C=C\c2ccc(O)c(O)c2)C(=O)O | Integrase |
| CHEMBL3238130 | CC(=O)N1CCN(CC1)c2ccc3C(=O)C(=CN(Cc4ccc(F)cc4)c3c2)C(=O)\C=C(/O)\C(=O)O | Integrase |
| CHEMBL209958 | Oc1c2C(=O)N(Cc3ccc(F)cc3)C(=O)c2c(O)c4ncccc14 | Integrase |
| CHEMBL437708 | Oc1nc(nc(C(=O)NCc2ccccc2)c1O)c3cccs3 | Integrase |
| CHEMBL3288819 | CN(C)C1=C(C(=O)NCc2ccc(F)cc2F)C(=O)N(O)c3ncccc13 | Integrase |
| CHEMBL377267 | COc1c2C(C)N(Cc3ccc(F)cc3)C(=O)c2c(O)c4ncccc14 | Integrase |
| CHEMBL216058 | CC1C=C(NC(=O)C2=CC(=CC2C)NC(=O)C3=CC(=CC3C)NC(=O)c4ccc(cc4)C(=O)NC5=CC(C)C(=C5)C(=O)NC6=CC(C)C(=C6)C(=O)NC7=CC(C)C(=C7)C(=O)NCCC(=N)N)C=C1C(=O)NCCC(=N)N | Integrase |
| CHEMBL386055 | COc1cnc2c(O)c3C(=O)N(Cc4ccc(F)cc4)Cc3c(OC)c2c1 | Integrase |
| CHEMBL413552 | OC1=C(Oc2cc(O)c(O)c(O)c2C1=O)c3ccc(O)c(O)c3 | Integrase |
| CHEMBL145280 | OC(=O)C(=O)CC(=O)c1cccc(Cc2ccccc2F)c1 | Integrase |
| CHEMBL3238128 | CN1CCN(CC1)c2ccc3C(=O)C(=CN(Cc4ccc(F)cc4)c3c2)C(=O)\C=C(/O)\C(=O)O | Integrase |
| CHEMBL403498 | CC1C=C(NC(=O)C2=CC(=CC2C)NC(=O)C3=CC(=CC3C)NC(=O)c4ccc(nc4)C(=O)NC5=CC(C)C(=C5)C(=O)NC6=CC(C)C(=C6)C(=O)NC7=CC(C)C(=C7)C(=O)NCCC(=N)N)C=C1C(=O)NCCC(=N)N | Integrase |
| CHEMBL145705 | COc1ccc(Cc2ccccc2)cc1C(=O)CC(=O)C(=O)O | Integrase |
| CHEMBL147147 | OC(=O)C(=O)CC(=O)c1cccc(Cc2ccc(F)cc2)c1 | Integrase |
| CHEMBL194833 | COc1ccc(Cc2ccccc2)cc1\C(=C\C(=O)C(=O)O)\O | Integrase |
| CHEMBL343646 | CC(C)Oc1ccc(Cc2ccccc2)cc1C(=O)CC(=O)C(=O)O | Integrase |
| CHEMBL148241 | CCOc1ccc(Cc2ccccc2)cc1C(=O)CC(=O)C(=O)O | Integrase |
| CHEMBL3288270 | CC(=O)Nc1ccc2C(C(=O)NCc3ccc(F)cc3)C(=O)N(O)C(=O)c2c1 | Integrase |
| CHEMBL143614 | OC(=O)C(=O)CC(=O)c1csc(Cc2ccccc2)c1 | Integrase |
| CHEMBL357909 | OC(=O)C(=O)CC(=O)c1cccc(Cc2ccccc2Cl)c1 | Integrase |
| CHEMBL145335 | OC(=O)C(=O)CC(=O)c1cccc(Cc2cccc(Cl)c2)c1 | Integrase |
| CHEMBL18635 | Oc1ccc2cc(ccc2c1O)C(=O)NCCCNC(=O)c3ccc4c(O)c(O)ccc4c3 | Integrase |
| CHEMBL3288275 | Nc1ccc2C(C(=O)NCc3ccc(F)cc3)C(=O)N(O)C(=O)c2c1 | Integrase |
| CHEMBL424782 | Oc1c2C(=O)N(Cc3ccc(Cl)c(Cl)c3)C(=O)c2c(O)c4nccnc14 | Integrase |
| CHEMBL3288815 | ON1C(=O)C(=C(Nc2ccc(cc2)c3ccc(cc3)C#N)c4cccnc14)C(=O)NCc5ccc(F)cc5F | Integrase |
| CHEMBL3288816 | Nc1ccc(cc1)c2ccc(NC3=C(C(=O)NCc4ccc(F)cc4F)C(=O)N(O)c5ncccc35)cc2 | Integrase |
| CHEMBL371471 | COc1cc(Cc2ccccc2)cc(c1)\C(=C\C(=O)C(=O)O)\O | Integrase |
| CHEMBL3288279 | ON1C(=O)C(C(=O)NCc2ccc(F)cc2)c3ccc(Cl)cc3C1=O | Integrase |
| CHEMBL147317 | COc1cc(Cc2ccccc2)cc(c1)C(=O)CC(=O)C(=O)O | Integrase |
| CHEMBL259011 | COc1cccc2c1c(cn2Cc3ccc(F)cc3)C(=O)\C=C(/O)\C(=O)O | Integrase |
| CHEMBL213852 | COc1c2C(=C)N(Cc3ccc(F)cc3)C(=O)c2c(O)c4ncccc14 | Integrase |
| CHEMBL194241 | CCOC(=O)C(=O)\C=C(/O)\c1cn(Cc2ccccc2)c3ccc(OC)cc13 | Integrase |
| CHEMBL20342 | OC(=O)C(=O)CC(=O)c1ccc(CN=[N+]=[N-])c(CN=[N+]=[N-])c1 | Integrase |
| CHEMBL146777 | COc1ccc(cc1Cc2ccccc2)C(=O)CC(=O)C(=O)O | Integrase |
| CHEMBL197584 | COc1ccc(cc1Cc2ccccc2)\C(=C\C(=O)C(=O)O)\O | Integrase |
| CHEMBL3238133 | CN(C)c1ccc2C(=O)C(=CN(Cc3ccc(F)cc3)c2c1)C(=O)\C=C(/O)\C(=O)O | Integrase |
| CHEMBL379132 | CN(CCN(C)c1nc(C(=O)NCc2ccc(F)cc2)c(O)c3ncccc13)C(=O)C | Integrase |
| CHEMBL3288261 | ON1C(=O)C(C(=O)NCc2ccc(F)cc2)c3ccc(NC(=O)c4ccccn4)cc3C1=O | Integrase |
| CHEMBL3238123 | CCOC(=O)\C(=C\C(=O)C1=CN(Cc2ccc(F)cc2)c3cc(ccc3C1=O)N4CCN(CC4)C(=O)C)\O | Integrase |
| CHEMBL232801 | Nc1nc[nH]n1 | Integrase |
| CHEMBL368301 | Oc1c2C(=O)N(Cc3ccc(F)c(Cl)c3)C(=O)c2c(O)c4nccnc14 | Integrase |
| CHEMBL197638 | OC(=O)C(=O)\C=C(/O)\c1ccsc1Cc2ccccc2 | Integrase |
| CHEMBL359212 | OC(=O)C(=O)CC(=O)c1ccsc1Cc2ccccc2 | Integrase |
| CHEMBL360752 | Oc1c2C(=O)N(Cc3cc(Cl)cc(Cl)c3)C(=O)c2c(O)c4nccnc14 | Integrase |
| CHEMBL211110 | Oc1c2C(=O)N(Cc3ccc(F)cc3)Cc2c(C(=O)NCc4ccccn4)c5cccnc15 | Integrase |
| CHEMBL130911 | Oc1ccc(\C=C\2/CSC\C(=C/c3ccc(O)c(O)c3)\C2=O)cc1O | Integrase |
| CHEMBL1161516 | Oc1ccc(\C=C\2/CNC\C(=C/c3ccc(O)c(O)c3)\C2=O)cc1O | Integrase |
| CHEMBL419981 | Oc1ccc(\C=C\2/COC\C(=C/c3ccc(O)c(O)c3)\C2=O)cc1O | Integrase |
| CHEMBL282731 | OC(=O)[C@H](OC(=O)\C=C\c1ccc(O)c(O)c1)[C@@H](OC(=O)\C=C\c2ccc(O)c(O)c2)C(=O)O | Integrase |
| CHEMBL217776 | CN(C)C(c1ccccc1)c2nc(O)c(O)c(n2)C(=O)NCc3ccc(F)cc3 | Integrase |
| CHEMBL407451 | CN1CCCCC1c2nc(O)c(O)c(n2)C(=O)NCc3ccc(F)cc3 | Integrase |
| CHEMBL211014 | OC(=O)C(=O)CC(=O)CC(=O)c1ccccc1Cl | Integrase |
| CHEMBL216373 | CN(C)Cc1nc(O)c(O)c(n1)C(=O)NCc2ccc(F)cc2 | Integrase |
| CHEMBL340775 | Cl.Oc1ccc(\C=C\2/CNC\C(=C/c3ccc(O)c(O)c3)\C2=O)cc1O | Integrase |
| CHEMBL370620 | OC(=O)C(=O)\C=C(/O)\C1=CN(Cc2ccccc2)C(=O)N(Cc3ccccc3)C1=O | Integrase |
| CHEMBL85660 | O\C(=C/C(=O)C(=O)NCCCNC(=O)C(=O)\C=C(/O)\c1cccc(Cc2ccccc2F)c1)\c3cccc(Cc4ccccc4F)c3 | Integrase |
| CHEMBL314866 | O\C(=C/C(=O)C(=O)NCCNC(=O)C(=O)\C=C(/O)\c1cccc(Cc2ccccc2F)c1)\c3cccc(Cc4ccccc4F)c3 | Integrase |
| CHEMBL86133 | OC(=O)C(=O)\C=C(/O)\c1cccc(c1)C(c2cccc(c2)\C(=C\C(=O)C(=O)O)\O)c3ccccc3F | Integrase |
| CHEMBL425516 | Oc1c2C(=O)N(Cc3cccc(Br)c3)C(=O)c2c(O)c4nccnc14 | Integrase |
| CHEMBL3238129 | CCN1CCN(CC1)c2ccc3C(=O)C(=CN(Cc4ccc(F)cc4)c3c2)C(=O)\C=C(/O)\C(=O)O | Integrase |
| CHEMBL216389 | CN1CCCCC1C2=NC(=C(O)C(=O)N2C)C(=O)NCc3ccc(F)cc3 | Integrase |
| CHEMBL209488 | COc1c2CN(Cc3ccc(F)cc3)C(=O)c2c(O)c4nccnc14 | Integrase |
| CHEMBL384158 | COc1c2c(C(=O)N(Cc3ccc(F)cc3)C2(C)C)c(O)c4ncccc14 | Integrase |
| CHEMBL367104 | Oc1c2C(=O)N(Cc3cccc(Cl)c3)C(=O)c2c(O)c4nccnc14 | Integrase |
| CHEMBL371867 | OC(=O)C(=O)\C=C(/O)\c1cccn1Cc2ccccc2 | Integrase |
| CHEMBL342295 | OC(=O)C(=O)CC(=O)c1cccn1Cc2ccccc2 | Integrase |
| CHEMBL372325 | CCOC(=O)C(=O)\C=C(/O)\c1cn(Cc2ccc(F)cc2)c3ccc(OC)cc13 | Integrase |
| CHEMBL3288262 | ON1C(=O)C(C(=O)NCc2ccc(F)cc2)c3ccc(NC(=O)Cc4ccccc4)cc3C1=O | Integrase |
| CHEMBL1159990 | CC(Cc1ccc(O)c(O)c1)(C(=O)O)C(C)(Cc2ccc(O)c(O)c2)C(=O)O | Integrase |
| CHEMBL3288276 | ON1C(=O)C(C(=O)NCc2ccc(F)cc2)c3ccc(F)cc3C1=O | Integrase |
| CHEMBL3288277 | ON1C(=O)C(C(=O)NCc2ccc(F)cc2)c3cc(F)ccc3C1=O | Integrase |
| CHEMBL204751 | CCOC(=O)C(=O)\C=C(/O)\C1=CN(Cc2ccc(F)cc2)c3ccc(cc3C1=O)\C(=C\C(=O)C(=O)OCC)\O | Integrase |
| CHEMBL177126 | O[C@@H]1C[C@@](O)(C[C@@H](OC(=O)\C=C\c2ccc(O)c(O)c2)[C@H]1OC(=O)\C=C\c3ccc(O)c(O)c3)C(=O)O | Integrase |
| CHEMBL144727 | OC(=O)C(=O)CC(=O)c1cccc(Cc2cccc(F)c2)c1 | Integrase |
| CHEMBL198000 | OC(=O)C(=O)\C=C(/O)\c1cccc(Cc2cccc(F)c2)c1 | Integrase |
| CHEMBL214371 | CN(C)S(=O)(=O)Oc1c2CN(Cc3ccc(F)cc3)C(=O)c2c(O)c4ncccc14 | Integrase |
| CHEMBL177640 | Cc1ccc2c(O)c3C(=O)N(Cc4cccc(Br)c4)C(=O)c3c(O)c2n1 | Integrase |
| CHEMBL1159993 | OC(=O)C(=O)\C=C(\O)/c1ccc(CN=[N+]=[N-])c(CN=[N+]=[N-])c1 | Integrase |
| CHEMBL3233021 | OC(=O)\C(=C\C(=O)c1cn(Cc2ccc(cc2)C(F)(F)F)c3ccc(Cl)cc13)\O | Integrase |
| CHEMBL3288272 | COc1ccc2C(C(=O)NCc3ccc(F)cc3)C(=O)N(O)C(=O)c2c1 | Integrase |
| CHEMBL329488 | [Na+].Oc1cc(\C=C\c2ccc3ccc(C(=O)[O-])c(O)c3n2)cc(O)c1O | Integrase |
| CHEMBL3259887 | Cc1nc2ccc(Br)cc2c(c1CC(=O)O)c3ccc(Cl)cc3 | Integrase |
| CHEMBL3288823 | ON1C(=O)C(=C(NCCCCc2ccccc2)c3cccnc13)C(=O)NCc4ccc(F)cc4F | Integrase |
| CHEMBL144613 | CC(=O)Nc1cc(cc2cc(cc(O)c12)S(=O)(=O)O)S(=O)(=O)O | Integrase |
| CHEMBL213467 | COc1c2C(SCCC(=O)O)N(Cc3ccc(F)cc3)C(=O)c2c(O)c4ncccc14 | Integrase |
| CHEMBL37869 | OC(=O)c1ccc2ccc(\C=C\c3cc(O)c(O)c(O)c3)nc2c1O | Integrase |
| CHEMBL292992 | CN(C)[C@H]1[C@@H]2C[C@H]3C(=C(O)c4c(C)cccc4C3(C)O)C(=O)C2C(=C(C(=O)NCN5CCN(CC5)C(=N)N=C(N)N)C1=O)O | Integrase |
| CHEMBL85597 | O\C(=C/C(=O)C(=O)N1CCN(CC1)C(=O)C(=O)\C=C(/O)\c2cccc(Cc3ccccc3F)c2)\c4cccc(Cc5ccccc5F)c4 | Integrase |
| CHEMBL87764 | OC(=O)C(=O)\C=C(/O)\c1cccc(OCc2ccccc2COc3cccc(c3)\C(=C\C(=O)C(=O)O)\O)c1 | Integrase |
| CHEMBL209641 | COC(=O)CCSC1N(Cc2ccc(F)cc2)C(=O)c3c(O)c4ncccc4c(OC)c13 | Integrase |
| CHEMBL3288836 | CCOC(=O)[C@@H]1CCCN1C2=C(C(=O)NCc3ccc(F)cc3F)C(=O)N(O)c4ncccc24 | Integrase |
| CHEMBL3259889 | CC(C(=O)O)c1c(C)nc2ccc(Br)cc2c1c3ccc(Cl)cc3 | Integrase |
| CHEMBL278545 | OC(=O)C(=O)CC(=O)c1ccc2C(CCC(N=[N+]=[N-])c2c1)N=[N+]=[N-] | Integrase |
| CHEMBL1159989 | OC(=O)C(=O)\C=C(\O)/c1ccc2C(CCC(N=[N+]=[N-])c2c1)N=[N+]=[N-] | Integrase |
| CHEMBL32711 | OC1=C(C(C2=C(O)Oc3cc(O)ccc3C2=O)c4ccc(cc4)C(C5=C(O)Oc6cc(O)ccc6C5=O)C7=C(O)Oc8cc(O)ccc8C7=O)C(=O)c9ccc(O)cc9O1 | Integrase |
| CHEMBL379382 | Oc1c2C(=O)N(Cc3ccc(F)cc3)Cc2c(c4c(F)cccc4F)c5cccnc15 | Integrase |
| CHEMBL3288813 | ON1C(=O)C(=C(Nc2ccc(cc2)c3ccccc3)c4cccnc14)C(=O)NCc5ccc(F)cc5F | Integrase |
| CHEMBL108253 | OC(=O)C(=O)\C=C(/O)\c1cccc(OCc2ccccc2)c1 | Integrase |
| CHEMBL18927 | OC(=O)C(=O)CC(=O)c1cccc(OCc2ccccc2)c1 | Integrase |
| CHEMBL378395 | CCOC(=O)c1ccccc1c2c3CN(Cc4ccc(F)cc4)C(=O)c3c(O)c5ncccc25 | Integrase |
| CHEMBL3259886 | Cc1ccc(cc1)c2c(CC(=O)O)c(C)nc3ccc(Br)cc23 | Integrase |
| CHEMBL414654 | Oc1c2C(=O)N(Cc3cc(Br)ccc3F)C(=O)c2c(O)c4nccnc14 | Integrase |
| CHEMBL379974 | CN(C)S(=O)(=O)Oc1c2c(C(=O)N(Cc3ccc(F)cc3)C24CC4)c(O)c5ncccc15 | Integrase |
| CHEMBL3238131 | OC(=O)\C(=C\C(=O)C1=CN(Cc2ccc(F)cc2)c3cc(ccc3C1=O)N4CCSCC4)\O | Integrase |
| CHEMBL20592 | OC(=O)C(=O)CC(=O)c1ccc2C(CC(N=[N+]=[N-])c2c1)N=[N+]=[N-] | Integrase |
| CHEMBL1159988 | OC(=O)C(=O)\C=C(\O)/c1ccc2C(CC(N=[N+]=[N-])c2c1)N=[N+]=[N-] | Integrase |
| CHEMBL35842 | Oc1c(ccc2cccnc12)C(=O)c3cccc(Cc4ccccc4)c3 | Integrase |
| CHEMBL472863 | OC(=O)C(CNC(=O)\C=C\c1ccc(O)c(O)c1)OC(=O)\C=C\c2ccc(O)c(O)c2 | Integrase |
| CHEMBL362252 | Oc1c2C(=O)N(Cc3cc(F)cc(F)c3)C(=O)c2c(O)c4nccnc14 | Integrase |
| CHEMBL3286423 | Oc1cc(O)c2C(=O)C(=C(Oc2c1)c3ccc(O)c(O)c3)OCc4ccc(F)cc4 | Integrase |
| CHEMBL366982 | Oc1c2C(=O)N(Cc3cccc(Br)c3)C(=O)c2c(O)c4ncccc14 | Integrase |
| CHEMBL286477 | Oc1ccc(\C=C\C(=O)OCCOC(=O)\C=C\c2ccc(O)c(O)c2)cc1O | Integrase |
| CHEMBL85802 | OC(=O)C(=O)\C=C(/O)\c1cccc(Cc2ccccc2F)c1 | Integrase |
| CHEMBL86448 | OC(=O)C(=O)\C=C(/O)\c1cccc(OCc2cccc(COc3cccc(c3)\C(=C\C(=O)C(=O)O)\O)c2)c1 | Integrase |
| CHEMBL369841 | Oc1c2C(=O)N(Cc3ccc(Cl)cc3)C(=O)c2c(O)c4nccnc14 | Integrase |
| CHEMBL360274 | Oc1c2C(=O)N(Cc3ccc(F)cc3)C(=O)c2c(O)c4nccnc14 | Integrase |
| CHEMBL3233908 | COc1ccc2c(c1)c(cn2Cc3c(C)cc(C)cc3C)C(=O)\C=C(/O)\C(=O)O | Integrase |
| CHEMBL377621 | CN(C)S(=O)(=O)Oc1c2C(=C)N(Cc3ccc(F)cc3)C(=O)c2c(O)c4ncccc14 | Integrase |
| CHEMBL177215 | COc1ccc(CN2C(=O)c3c(O)c4nccnc4c(O)c3C2=O)c(OC)c1 | Integrase |
| CHEMBL177622 | Oc1c2C(=O)N(Cc3cccc(c3)C(F)(F)F)C(=O)c2c(O)c4nccnc14 | Integrase |
| CHEMBL177515 | COc1cccc(CN2C(=O)c3c(O)c4nccnc4c(O)c3C2=O)c1OC | Integrase |
| CHEMBL378373 | OC(=O)C(=O)\C=C(/O)\c1ccc2ncc(\C(=C\C(=O)C(=O)O)\O)c(O)c2c1 | Integrase |
| CHEMBL415678 | OC(=O)C(OC(=O)c1ccc(O)c(O)c1)C(OC(=O)c2ccc(O)c(O)c2)C(=O)O | Integrase |
| CHEMBL424780 | Oc1c2C(=O)N(Cc3ccc(Br)cc3F)C(=O)c2c(O)c4nccnc14 | Integrase |
| CHEMBL151 | Oc1cc(O)c2C(=O)C=C(Oc2c1)c3ccc(O)c(O)c3 | Integrase |
| CHEMBL3288821 | ON1C(=O)C(=C(NC2CCCCCC2)c3cccnc13)C(=O)NCc4ccc(F)cc4F | Integrase |
| CHEMBL19977 | OC(=O)C(=O)CC(=O)c1cc(OCc2ccccc2)cc(OCc3ccccc3)c1 | Integrase |
| CHEMBL196421 | OC(=O)C(=O)\C=C(/O)\c1ccc(Cc2ccccc2)s1 | Integrase |
| CHEMBL553067 | Cl.CN(C)[C@H]1[C@@H]2C[C@H]3C(=C(O)c4c(C)cccc4C3(C)O)C(=O)C2C(=C(C(=O)NCNC(CO)(CO)CO)C1=O)O | Integrase |
| CHEMBL174366 | ONC(=O)c1cc(O)c(O)c(O)c1 | Integrase |
| CHEMBL145946 | OC(=O)C(=O)CC(=O)c1ccn(Cc2ccccc2)c1 | Integrase |
| CHEMBL199874 | OC(=O)C(=O)\C=C(/O)\C1=CN(Cc2ccccc2)C(=O)NC1=O | Integrase |
| CHEMBL3290944 | ON1C(=O)N=C(Nc2ccc(cc2)N3CCOCC3)c4cccnc14 | Integrase |
| CHEMBL321391 | COc1ccc(\C=C\2/C(=NN(C(=O)c3ccccc3O)C2=O)C)cc1OC | Integrase |
| CHEMBL358506 | OC(=O)C(=O)CC(=O)c1ccc(Cc2ccccc2)s1 | Integrase |
| CHEMBL144118 | OC(=O)C(=O)CC(=O)c1cccc(Cc2ccc(Cl)cc2)c1 | Integrase |
| CHEMBL174793 | Oc1c2C(=O)N(Cc3cccc(c3)c4ccccc4F)C(=O)c2c(O)c5nccnc15 | Integrase |
| CHEMBL30868 | OC(=O)C(COC(=O)\C=C\c1ccc(O)c(O)c1)OC(=O)\C=C\c2ccc(O)c(O)c2 | Integrase |
| CHEMBL177724 | Oc1c2C(=O)N(Cc3ccc4OCOc4c3)C(=O)c2c(O)c5nccnc15 | Integrase |
| CHEMBL174001 | COc1ccc(CN2C(=O)c3c(O)c4nccnc4c(O)c3C2=O)cc1 | Integrase |
| CHEMBL177690 | Oc1c2C(=O)N(Cc3cccc(F)c3)C(=O)c2c(O)c4nccnc14 | Integrase |
| CHEMBL50 | OC1=C(Oc2cc(O)cc(O)c2C1=O)c3ccc(O)c(O)c3 | Integrase |
| CHEMBL108031 | COc1ccc(\C=C\2/C(=NN(C(=O)c3ccccc3O)C2=O)C)cc1 | Integrase |
| CHEMBL111182 | CC1=NN(C(=O)c2ccccc2O)C(=O)/C/1=C/c3ccccc3O | Integrase |
| CHEMBL164 | OC1=C(Oc2cc(O)cc(O)c2C1=O)c3cc(O)c(O)c(O)c3 | Integrase |
| CHEMBL288097 | Oc1ccc2Oc3ccc(Cc4ccccc4)cc3C(=O)c2c1O | Integrase |
| CHEMBL3238121 | CCOC(=O)\C(=C\C(=O)C1=CN(Cc2ccc(F)cc2)c3cc(ccc3C1=O)N4CCN(C)CC4)\O | Integrase |
| CHEMBL3143741 | CC[C@H](C)[C@@H](N)C(=O)N[C@@H](CC(C)C)C(=O)N1CCC[C@H]1C(=O)N[C@@H](Cc2c[nH]c3ccccc23)C(=O)N[C@@H](CCCCN)C(=O)N[C@@H](Cc4c[nH]c5ccccc45)C(=O)N6CCC[C@H]6C(=O)N[C@@H](Cc7c[nH]c8ccccc78)C(=O)N[C@@H](Cc9c[nH]c%10ccccc9%10)C(=O)N%11CCC[C@H]%11C(=O)N[C@@H](Cc%12c[nH]c%13ccccc%12%13)C(=O)N%14CCC[C@H]%14C(=O)N%15CCC[C@H]%15C(=O)NCCCCC(NC(=O)[C@@H]%16CCCN%16C(=O)[C@@H]%17CCCN%17C(=O)[C@H](Cc%18c[nH]c%19ccccc%18%19)NC(=O)[C@@H]%20CCCN%20C(=O)[C@H](Cc%21c[nH]c%22ccccc%21%22)NC(=O)[C@H](Cc%23c[nH]c%24ccccc%23%24)NC(=O)[C@@H]%25CCCN%25C(=O)[C@H](Cc%26c[nH]c%27ccccc%26%27)NC(=O)[C@H](CCCCN)NC(=O)[C@H](Cc%28c[nH]c%29ccccc%28%29)NC(=O)[C@@H]%30CCCN%30C(=O)[C@H](CC(C)C)NC(=O)[C@@H](N)[C@@H](C)CC)C(=O)NC(CCCCNC(=O)C(CCCCNC(=O)[C@@H]%31CCCN%31C(=O)[C@@H]%32CCCN%32C(=O)[C@H](Cc%33c[nH]c%34ccccc%33%34)NC(=O)[C@@H]%35CCCN%35C(=O)[C@H](Cc%36c[nH]c%37ccccc%36%37)NC(=O)[C@H](Cc%38c[nH]c%39ccccc%38%39)NC(=O)[C@@H]%40CCCN%40C(=O)[C@H](Cc%41c[nH]c%42ccccc%41%42)NC(=O)[C@H](CCCCN)NC(=O)[C@H](Cc%43c[nH]c%44ccccc%43%44)NC(=O)[C@@H]%45CCCN%45C(=O)[C@H](CC(C)C)NC(=O)[C@@H](N)[C@@H](C)CC)NC(=O)[C@@H]%46CCCN%46C(=O)[C@@H]%47CCCN%47C(=O)[C@H](Cc%48c[nH]c%49ccccc%48%49)NC(=O)[C@@H]%50CCCN%50C(=O)[C@H](Cc%51c[nH]c%52ccccc%51%52)NC(=O)[C@H](Cc%53c[nH]c%54ccccc%53%54)NC(=O)[C@@H]%55CCCN%55C(=O)[C@H](Cc%56c[nH]c%57ccccc%56%57)NC(=O)[C@H](CCCCN)NC(=O)[C@H](Cc%58c[nH]c%59ccccc%58%59)NC(=O)[C@@H]%60CCCN%60C(=O)[C@H](CC(C)C)NC(=O)[C@@H](N)[C@@H](C)CC)C(=O)N | Integrase |
| CHEMBL325111 | COc1cc(\C=C\2/C(=NN(C(=O)c3ccccc3O)C2=O)C)ccc1O | Integrase |
| CHEMBL149674 | O[C@@H]1C[C@](O)(CC(OC(=O)\C=C\c2ccc(O)c(O)c2)[C@@H]1OC(=O)\C=C\c3ccc(O)c(O)c3)C(=O)O | Integrase |
| CHEMBL366356 | OC(=O)c1cc(O)c(O)c(OC(=O)c2cc(O)c(O)c(O)c2)c1 | Integrase |
| CHEMBL202166 | OC(=O)C(=O)\C=C(/O)\C1=CN(Cc2ccccc2F)C(=O)N(Cc3ccccc3F)C1=O | Integrase |
| CHEMBL433434 | COc1ccc(\C=C/2\C(=NN(C(=O)c3ccccc3O)C2=O)C)cc1 | Integrase |
| CHEMBL129073 | Oc1ccc(\C=C\C(=O)\C=C\c2ccc(O)c(O)c2)cc1O | Integrase |
| CHEMBL59548 | OC(=O)c1ccc2ccc(\C=C\c3ccc(O)c(c3)C(=O)O)nc2c1O | Integrase |
| CHEMBL110315 | CC1=NN(C(=O)c2ccccc2O)C(=O)/C/1=C/c3ccc(cc3)[N+](=O)[O-] | Integrase |
| CHEMBL209614 | OC(=O)C(=O)CC(=O)CC(=O)c1cccc2ccccc12 | Integrase |
| CHEMBL109077 | CC1=NN(C(=O)c2ccccc2O)C(=O)/C/1=C/c3ccc(O)cc3 | Integrase |
| CHEMBL3290941 | ON1C(=O)N=C(NCc2ccc(cc2)c3ccccc3)c4cccnc14 | Integrase |
| CHEMBL177380 | Oc1c2C(=O)N(Cc3ccccc3Br)C(=O)c2c(O)c4nccnc14 | Integrase |
| CHEMBL441250 | OC1C(CC(O)(CC1OC(=O)\C=C/c2ccc(O)c(O)c2)C(=O)O)OC(=O)\C=C/c3ccc(O)c(O)c3 | Integrase |
| CHEMBL35109 | O\C(=C/C(=O)c1nn[nH]n1)\c2c[nH]c3ccc(Cl)cc23 | Integrase |
| CHEMBL368424 | Cc1csc(c1)c2cccc(CN3C(=O)c4c(O)c5nccnc5c(O)c4C3=O)c2 | Integrase |
| CHEMBL431948 | OC1CC(CC(OC(=O)\C=C/c2ccc(O)c(O)c2)C1O)(OC(=O)\C=C/c3ccc(O)c(O)c3)C(=O)O | Integrase |
| CHEMBL83025 | O\C(=C/C(=O)C(=O)NCCCCCNC(=O)C(=O)\C=C(/O)\c1cccc(Cc2ccccc2F)c1)\c3cccc(Cc4ccccc4F)c3 | Integrase |
| CHEMBL36640 | COc1cc(\C=C\c2ccc3ccc(C(=O)O)c(O)c3n2)cc(O)c1O | Integrase |
| CHEMBL36303 | Oc1ccccc1C(=O)NNC(=O)c2ccccc2O | Integrase |
| CHEMBL129964 | O\C(=C/C(=O)\C=C\c1ccc(O)c(O)c1)\C=C\c2ccc(O)c(O)c2 | Integrase |
| CHEMBL380548 | OC(=O)C(=O)\C=C(/O)\C1=CN(Cc2ccc(F)cc2)C(=O)N(Cc3ccc(F)cc3)C1=O | Integrase |
| CHEMBL210396 | CN(C)C(=O)Oc1c2CN(Cc3ccc(F)cc3)C(=O)c2c(O)c4ncccc14 | Integrase |
| CHEMBL362916 | CCOC(=O)C(=O)\C=C(/O)\c1cn(Cc2ccccc2)c3ccccc13 | Integrase |
| CHEMBL177405 | COc1ccc(OC)c(CN2C(=O)c3c(O)c4nccnc4c(O)c3C2=O)c1 | Integrase |
| CHEMBL8260 | Oc1cc2OC(=CC(=O)c2c(O)c1O)c3ccccc3 | Integrase |
| CHEMBL3290940 | ON1C(=O)N=C(Nc2ccc(cc2)c3ccccc3)c4cccnc14 | Integrase |
| CHEMBL339815 | Oc1ccc(\C=C\C(=O)c2cccc(c2)C(=O)\C=C\c3ccc(O)c(O)c3)cc1O | Integrase |
| CHEMBL293914 | OC1=C(C(C2=C(O)Oc3ccccc3C2=O)c4ccc(cc4)C(C5=C(O)Oc6ccccc6C5=O)C7=C(O)Oc8ccccc8C7=O)C(=O)c9ccccc9O1 | Integrase |
| CHEMBL100082 | [Na+].Oc1ccc(\C=C\c2ccc3ccc(C(=O)[O-])c(O)c3n2)cc1O | Integrase |
| CHEMBL149893 | COC(=O)O[C@]1(C[C@@H](OC(=O)\C=C\c2ccc(O)c(O)c2)[C@@H](O)C(C1)OC(=O)\C=C\c3ccc(O)c(O)c3)C(=O)O | Integrase |
| CHEMBL267578 | Oc1cc2ccc(cc2cc1O)C(=O)NCCCNC(=O)c3ccc4cc(O)c(O)cc4c3 | Integrase |
| CHEMBL177311 | COc1ccccc1CN2C(=O)c3c(O)c4nccnc4c(O)c3C2=O | Integrase |
| CHEMBL378117 | CC1N(Cc2ccc(F)cc2)C(=O)c3c(O)c4ncccc4c(OS(=O)(=O)N(C)C)c13 | Integrase |
| CHEMBL3288814 | ON1C(=O)C(=C(Nc2ccc3c(Cc4ccccc34)c2)c5cccnc15)C(=O)NCc6ccc(F)cc6F | Integrase |
| CHEMBL3288278 | ON1C(=O)C(C(=O)NCc2ccc(F)cc2)c3cccc(F)c3C1=O | Integrase |
| CHEMBL148364 | OC(=O)C(OC(=O)Cc1ccc(O)c(O)c1)C(OC(=O)Cc2ccc(O)c(O)c2)C(=O)O | Integrase |
| CHEMBL129148 | Oc1ccc(\C=C\2/CCC\C(=C/c3ccc(O)c(O)c3)\C2=O)cc1O | Integrase |
| CHEMBL175266 | CC1=C2C=C(O)C(=O)C=C2Oc3cc(O)c(O)cc13 | Integrase |
| CHEMBL312608 | OC(=O)c1ccc2ccc(nc2c1O)C(=O)Nc3ccc(O)c(O)c3 | Integrase |
| CHEMBL412570 | CC1=NN(C(=O)c2ccccc2O)C(=O)/C/1=C/c3cccc(O)c3 | Integrase |
| CHEMBL37139 | COc1ccc(\C=C\c2ccc3ccc(C(=O)O)c(O)c3n2)cc1O | Integrase |
| CHEMBL190009 | Oc1cc(\C=C\S(=O)(=O)CS(=O)(=O)\C=C\c2cc(O)c(O)c(O)c2)cc(O)c1O | Integrase |
| CHEMBL53463 | COc1cccc2C(=O)c3c(O)c4C[C@](O)(C[C@H](O[C@H]5C[C@H](N)[C@H](O)[C@H](C)O5)c4c(O)c3C(=O)c12)C(=O)CO | Integrase |
| CHEMBL3288712 | OC1=C(Oc2cc(OCc3ccc(F)cc3)cc(O)c2C1=O)c4ccc(O)c(O)c4 | Integrase |
| CHEMBL3288713 | OC1=C(Oc2cc(cc(O)c2C1=O)N3CCOCC3)c4ccc(O)c(O)c4 | Integrase |
| CHEMBL357646 | OC(=O)C(OC(=O)c1cc(O)c(O)c(O)c1)C(OC(=O)c2cc(O)c(O)c(O)c2)C(=O)O | Integrase |
| CHEMBL3138501 | CC(CCC(=O)O)C1CC[C@H]2[C@@H]3C(CC4CC(CC[C@]4(C)[C@H]3CC(OC(=O)\C=C\c5ccc(O)c(O)c5)[C@]12C)OC(=O)\C=C\c6ccc(O)c(O)c6)OC(=O)\C=C\c7ccc(O)c(O)c7 | Integrase |
| CHEMBL39092 | OC(=O)c1ccc2ccc(\C=C\c3ccc(O)c(O)c3)nc2c1O | Integrase |
| CHEMBL174851 | Oc1c2C(=O)N(Cc3ccc(cc3)c4ccccc4F)C(=O)c2c(O)c5nccnc15 | Integrase |
| CHEMBL29898 | Oc1ccc2C(=O)c3c(O)ccc(O)c3C(=O)c2c1O | Integrase |
| CHEMBL349925 | C[C@H]1Oc2cc3c(c(O)cc(O)c3c(O)c2C(=O)[C@H]1C)c4c(O)cc(O)c5c(O)c6C(=O)C(=C(C)Oc6cc45)C | Integrase |
| CHEMBL140319 | Oc1c(oc2cccnc12)C(=O)c3cccc(Cc4ccccc4)c3 | Integrase |
| CHEMBL276080 | Oc1ccc(\C=C\2/CC\C(=C/c3ccc(O)c(O)c3)\C2=O)cc1O | Integrase |
| CHEMBL67076 | Oc1ccc(CCC(=O)c2ccc(O)c(O)c2O)cc1O | Integrase |
| CHEMBL196272 | OC(=O)\C=C\c1ccc(O)c(OC2=Cc3cc(O)c(O)cc3OC2=O)c1 | Integrase |
| CHEMBL301226 | COc1ccc(cc1O)c2c3c4cc(OC)c(O)cc4OC(=O)c3n5ccc6cc(O)c(OC)cc6c25 | Integrase |
| CHEMBL363333 | OC(=O)C(=O)\C=C(/O)\c1cc2ccccc2n1Cc3ccccc3 | Integrase |
| CHEMBL304722 | OC(CCc1ccc(O)c(O)c1)CC(=O)O[C@H]2Cc3cc(O)c(O)cc3O[C@H]2c4ccc(O)c(O)c4 | Integrase |
| CHEMBL146489 | Oc1ccc(\C=C/C(=O)OC2CCC(CC2)OC(=O)\C=C/c3ccc(O)c(O)c3)cc1O | Integrase |
| CHEMBL149826 | Oc1ccc(\C=C\C(=O)OC2CCC(CC2)OC(=O)\C=C\c3ccc(O)c(O)c3)cc1O | Integrase |
| CHEMBL368509 | C[C@@H](N1C(=O)c2c(O)c3nccnc3c(O)c2C1=O)c4ccccc4 | Integrase |
| CHEMBL357831 | OC(=O)C(OC(=O)c1cccc(O)c1O)C(OC(=O)c2cccc(O)c2O)C(=O)O | Integrase |
| CHEMBL3233907 | COc1ccc2c(c1)c(cn2Cc3cc(C)cc(C)c3)C(=O)\C=C(/O)\C(=O)O | Integrase |
| CHEMBL593913 | [Cl-].CCCCCCCCCCCCCCCCCC[n+]1ccn(C)c1 | Integrase |
| CHEMBL44 | Oc1ccc(cc1)C2=COc3cc(O)cc(O)c3C2=O | Integrase |
| CHEMBL366798 | Oc1ccccc1C(=O)NNC(=O)c2cc3ccccc3cc2O | Integrase |
| CHEMBL377693 | CCOc1ccc(cc1)c2c3CN(Cc4ccc(F)cc4)C(=O)c3c(O)c5ncccc25 | Integrase |
| CHEMBL431508 | OC(=O)c1ccc2ccc(\C=C\c3ccc(cc3)[N+](=O)[O-])nc2c1O | Integrase |
| CHEMBL39301 | OC(=O)c1ccc2ccc(\C=C\c3cc(Br)c(O)c(Br)c3)nc2c1O | Integrase |
| CHEMBL39481 | CC(=O)Nc1ccc(\C=C\c2ccc3ccc(C(=O)O)c(O)c3n2)cc1 | Integrase |
| CHEMBL430786 | [Na+].CN(C)[C@H]1[C@@H]2C[C@H]3C(=C(O)c4c(C)cccc4C3(C)O)C(=O)C2C(=C(C(=O)NNC5(CCCC5)C(=O)[O-])C1=O)O | Integrase |
| CHEMBL197554 | CCOC(=O)C(=O)\C=C(/O)\c1cn(Cc2ccc(F)cc2)c3ccccc13 | Integrase |
| CHEMBL315955 | OC1=C(C(C2=C(O)Oc3ccccc3C2=O)c4cccc(c4)C(C5=C(O)Oc6ccccc6C5=O)C7=C(O)Oc8ccccc8C7=O)C(=O)c9ccccc9O1 | Integrase |
| CHEMBL3290943 | ON1C(=O)N=C(NCc2ccc(cc2)c3ccncc3)c4cccnc14 | Integrase |
| CHEMBL108775 | OC(=O)C(=O)\C=C(/O)\c1ccc(cc1)\C(=C\C(=O)C(=O)O)\O | Integrase |
| CHEMBL341135 | Oc1ccc(cc1)C(=O)\C=C\c2ccc(O)c(O)c2 | Integrase |
| CHEMBL249447 | O[C@@H]1[C@@H](C[C@](O)(C[C@H]1OC(=O)\C=C\c2ccc(O)c(O)c2)C(=O)O)OC(=O)\C=C\c3ccc(O)c(O)c3 | Integrase |
| CHEMBL106446 | Oc1cc2CC3OC(Cc4cc(O)c(O)cc34)c2cc1O | Integrase |
| CHEMBL3290946 | ON1C(=O)N=C(Nc2ccc(CN3CCOCC3)cc2)c4cccnc14 | Integrase |
| CHEMBL595912 | [Cl-].CCCCCCCCCCCCCCCC[n+]1ccn(C)c1 | Integrase |
| CHEMBL3238126 | CCOC(=O)\C(=C\C(=O)C1=CN(Cc2ccc(F)cc2)c3cc(ccc3C1=O)N4CCN(CCCCl)CC4)\O | Integrase |
| CHEMBL358821 | OC1C[C@@](O)(C[C@@H](OC(=O)\C=C\c2ccc(O)c(O)c2)[C@H]1OC(=O)\C=C\c3ccc(O)c(O)c3)C(=O)O | Integrase |
| CHEMBL432207 | CC1=NN(C(=O)c2ccccc2O)C(=O)/C/1=C/c3cccc(c3)[N+](=O)[O-] | Integrase |
| CHEMBL594772 | CCCCCCCCCCCCCCCCCC[n+]1ccn(C)c1.F[P-](F)(F)(F)(F)F | Integrase |
| CHEMBL117 | Oc1cc(O)c2C(=O)C=C(Oc2c1)c3ccccc3 | Integrase |
| CHEMBL3288960 | OC1=C(Oc2cc(O)cc(O)c2C1=O)c3ccc(OCc4ccc(F)cc4)c(O)c3 | Integrase |
| CHEMBL175671 | Oc1c2C(=O)N(CCc3ccc4OCOc4c3)C(=O)c2c(O)c5nccnc15 | Integrase |
| CHEMBL129486 | O=C1NC(=S)SC1NS(=O)(=O)c2ccc(cc2)c3ncccn3 | Integrase |
| CHEMBL175404 | Oc1c2C(=O)N(Cc3cccc4ccccc34)C(=O)c2c(O)c5nccnc15 | Integrase |
| CHEMBL323204 | OC(=O)C(=O)\C=C(/O)\c1c[nH]c2ccccc12 | Integrase |
| CHEMBL367945 | Oc1c2C(=O)N(Cc3ccc(cc3)c4ccccc4)C(=O)c2c(O)c5nccnc15 | Integrase |
| CHEMBL28 | Oc1ccc(cc1)C2=CC(=O)c3c(O)cc(O)cc3O2 | Integrase |
| CHEMBL289303 | OC(=O)c1ccc2ccc(CCc3ccc(O)c(O)c3)nc2c1O | Integrase |
| CHEMBL82986 | OC(=O)c1ccc2ccc(nc2c1O)C(=O)NCc3ccc(O)c(O)c3O | Integrase |
| CHEMBL20091 | OC(=O)C(=O)CC(=O)c1cccc(CCC#N)c1 | Integrase |
| CHEMBL1159991 | OC(=O)C(=O)\C=C(\O)/c1cccc(CCC#N)c1 | Integrase |
| CHEMBL20378 | OC(=O)C(=O)CC(=O)c1cccc(CN=[N+]=[N-])c1 | Integrase |
| CHEMBL595010 | CCCCCCCCCCCCCCCCCC[n+]1ccn(C)c1.FC(F)(F)S(=O)(=O)[N-]S(=O)(=O)C(F)(F)F | Integrase |
| CHEMBL3233898 | OC(=O)\C(=C\C(=O)c1cn(Cc2ccc(cc2)C(F)(F)F)c3ccc(F)cc13)\O | Integrase |
| CHEMBL3290945 | ON1C(=O)N=C(NCc2ccc(cc2)N3CCOCC3)c4cccnc14 | Integrase |
| CHEMBL177704 | Cc1ccc(CN2C(=O)c3c(O)c4nccnc4c(O)c3C2=O)cc1 | Integrase |
| CHEMBL3288945 | Oc1ccc(cc1O)C2=CC(=O)c3c(O)cc(cc3O2)N4CCOCC4 | Integrase |
| CHEMBL418613 | OC(=O)c1ccc2ccc(\C=C\c3ccc(O)cc3)nc2c1O | Integrase |
| CHEMBL285415 | COc1cc(C)c2Oc3c(C=O)c(O)c(Cl)c(C)c3C(=O)Oc2c1C | Integrase |
| CHEMBL382680 | OC(=O)c1cnc2ccc(Cc3ccccc3)cc2c1O | Integrase |
| CHEMBL487258 | O[C@@H]1C[C@](C[C@@H](OC(=O)\C=C\c2ccc(O)c(O)c2)[C@H]1O)(OC(=O)\C=C\c3ccc(O)c(O)c3)C(=O)O | Integrase |
| CHEMBL188302 | CCn1c(cc2ccccc12)\C(=C\C(=O)C(=O)O)\O | Integrase |
| CHEMBL170405 | OC1=C(Oc2cc(O)ccc2C1=O)c3cc(O)c(O)c(O)c3 | Integrase |
| CHEMBL440354 | Oc1ccc(\C=C/C(=O)OC2CCCC(C2)OC(=O)\C=C/c3ccc(O)c(O)c3)cc1O | Integrase |
| CHEMBL416207 | OC(=O)C(COC(=O)\C=C\c1ccc(O)c(O)c1)NC(=O)\C=C\c2ccc(O)c(O)c2 | Integrase |
| CHEMBL86930 | OC(=O)C(=O)\C=C(/O)\c1cccc(OCc2ccc(COc3cccc(c3)\C(=C\C(=O)C(=O)O)\O)cc2)c1 | Integrase |
| CHEMBL3288948 | Oc1ccc(cc1)C2=CC(=O)c3c(O)cc(cc3O2)N4CCOCC4 | Integrase |
| CHEMBL9352 | Oc1ccc(cc1)[C@@H]2CC(=O)c3c(O)cc(O)cc3O2 | Integrase |
| CHEMBL3288956 | Oc1ccc(cc1)C2=COc3cc(cc(O)c3C2=O)N4CCOCC4 | Integrase |
| CHEMBL314013 | OC1=C(C(C2=C(O)Oc3cc(O)ccc3C2=O)c4ccc(\C=C\c5ccccc5)cc4)C(=O)c6ccc(O)cc6O1 | Integrase |
| CHEMBL149178 | Oc1ccc(\C=C\C(=O)OC2CCCC(C2)OC(=O)\C=C\c3ccc(O)c(O)c3)cc1O | Integrase |
| CHEMBL108511 | OC(=O)C(=O)\C=C(/O)\c1cccc(c1)\C(=C\C(=O)C(=O)O)\O | Integrase |
| CHEMBL107560 | O\C(=C/C(=O)c1nn[nH]n1)\c2c[nH]c3ccc(F)cc23 | Integrase |
| CHEMBL3288958 | OC1=C(Oc2cc(O)cc(OCc3ccc(F)cc3)c2C1=O)c4ccc(O)c(O)c4 | Integrase |
| CHEMBL3288959 | OC1=C(Oc2cc(O)cc(O)c2C1=O)c3ccc(O)c(OCc4ccc(F)cc4)c3 | Integrase |
| CHEMBL320040 | O\C(=C/C(=O)c1nn[nH]n1)\c2c[nH]c3ccccc23 | Integrase |
| CHEMBL38420 | OC(=O)c1ccc2ccc(\C=C\c3occc3)nc2c1O | Integrase |
| CHEMBL173530 | COc1cc(C)c2C(=O)Oc3c(C)c(O)c4C(=O)OC(O)c4c3Oc2c1C=O | Integrase |
| CHEMBL3290942 | ON1C(=O)N=C(Nc2ccc(cc2)c3ccncc3)c4cccnc14 | Integrase |
| CHEMBL3290947 | ON1C(=O)N=C(NCc2cccc(c2)N3CCOCC3)c4cccnc14 | Integrase |
| CHEMBL132058 | Oc1ccc(\C=C\C(=O)OCCc2ccccc2)c(O)c1O | Integrase |
| CHEMBL177547 | COc1cccc(OC)c1CN2C(=O)c3c(O)c4nccnc4c(O)c3C2=O | Integrase |
| CHEMBL131733 | Oc1cc(\C=C\C(=O)OCCc2ccccc2)cc(O)c1O | Integrase |
| CHEMBL22197 | OC(=O)C(=O)\C=C(/O)\c1cccc(Br)c1 | Integrase |
| CHEMBL20633 | OC(=O)C(=O)CC(=O)c1cc(CN=[N+]=[N-])cc(CN=[N+]=[N-])c1 | Integrase |
| CHEMBL265887 | CC[C@H](C)[C@H](NC(=O)[C@H](CC(=O)O)NC(=O)[C@@H](NC(=O)[C@H](C)NC(=O)[C@@H](NC(=O)[C@@H](NC(=O)[C@H](CC(=O)O)NC(=O)[C@@H](NC(=O)[C@@H](NC(=O)[C@H](CCCN=C(N)N)NC(=O)[C@H](CCC(=O)O)NC(=O)CNC(=O)[C@H](C)N)[C@@H](C)CC)C(C)C)[C@@H](C)CC)[C@@H](C)CC)[C@@H](C)O)C(=O)N[C@@H](CCC(=O)N)C(=O)N | Integrase |
| CHEMBL438262 | O\C(=C/C(=O)c1nc[nH]n1)\c2c[nH]c3ccc(Cl)cc23 | Integrase |
| CHEMBL412522 | CC[C@H](C)[C@H](NC(=O)[C@H](Cc1c[nH]c2ccccc12)NC(=O)[C@@H](NC(=O)[C@H](C)NC(=O)[C@H](CCC(=O)N)NC(=O)[C@H](Cc3c[nH]c4ccccc34)NC(=O)[C@H](Cc5ccc(O)cc5)NC(=O)[C@H](CCC(=O)O)NC(=O)[C@@H](NC(=O)[C@H](Cc6c[nH]c7ccccc67)NC(=O)[C@H](Cc8c[nH]c9ccccc89)NC(=O)[C@@H](NC(=O)[C@H](CCC(=O)O)NC(=O)[C@H](Cc%10c[nH]c%11ccccc%10%11)NC(=O)[C@@H](NC(=O)[C@@H](N)CCC(=O)O)[C@@H](C)O)[C@@H](C)O)[C@@H](C)O)[C@@H](C)O)C(=O)N%12CCC[C@H]%12C(=O)N[C@@H](CCC(=O)O)C(=O)N[C@@H](Cc%13c[nH]c%14ccccc%13%14)C(=O)N[C@@H](CCC(=O)O)C(=O)O | Integrase |
| CHEMBL187821 | Cn1cc(\C(=C\C(=O)C(=O)O)\O)c2ccccc12 | Integrase |
| CHEMBL162783 | CC1Oc2cc3c(c(O)cc(O)c3c(O)c2C(=O)C1C)c4c(O)cc(O)c5c(O)c6C(=O)C(C)C(C)Oc6cc45 | Integrase |
| CHEMBL321212 | COc1cc(\C=C\2/C(=NN(C(=O)c3ccccc3O)C2=O)C)cc(OC)c1OC | Integrase |
| CHEMBL3238127 | OC(=O)\C(=C\C(=O)C1=CN(Cc2ccc(F)cc2)c3cc(ccc3C1=O)N4CCNCC4)\O | Integrase |
| CHEMBL321846 | CC1=NN(C(=O)c2ccccc2O)C(=O)/C/1=C/c3occc3 | Integrase |
| CHEMBL437239 | CC[C@H](C)[C@H](NC(=O)CNC(=O)[C@H](CC(C)C)NC(=O)[C@H](C)NC(=O)[C@H](Cc1ccc(O)cc1)NC(=O)[C@H](CCC(=O)N)NC(=O)[C@H](CO)NC(=O)[C@H](CC(=O)O)NC(=O)[C@@H](NC(=O)[C@@H](NC(=O)[C@@H](NC(=O)[C@H](CC(=O)N)NC(=O)[C@@H](NC(=O)[C@H](CCC(=O)O)NC(=O)[C@H](CC(C)C)NC(=O)CNC(=O)[C@H](CO)NC(=O)[C@H](CC(=O)O)NC(=O)[C@H](CCC(=O)N)NC(=O)[C@@H](N)CC(C)C)C(C)C)[C@@H](C)CC)C(C)C)[C@@H](C)O)C(=O)O | Integrase |
| CHEMBL210374 | OC(=O)C(=O)CC(=O)CC(=O)c1ccc(cc1)c2ccccc2 | Integrase |
| CHEMBL188145 | OC(=O)C(=O)\C=C(/O)\c1cc2cc3OCOc3cc2n1Cc4ccccc4 | Integrase |
| CHEMBL265159 | CC[C@H](C)[C@H](NC(=O)[C@H](CO)NC(=O)[C@H](CCC(=O)O)NC(=O)[C@@H](NC(=O)[C@@H](NC(=O)[C@@H](NC(=O)[C@H](CCCCN)NC(=O)[C@H](CCC(=O)N)NC(=O)[C@@H](NC(=O)[C@H](C)NC(=O)[C@H](CCC(=O)O)NC(=O)[C@@H](NC(=O)[C@H](CC(C)C)NC(=O)[C@H](CCC(=O)N)NC(=O)[C@@H](N)CCCCN)[C@@H](C)O)C(C)C)[C@@H](C)CC)[C@@H](C)O)[C@@H](C)O)C(=O)N[C@@H](C(C)C)C(=O)N[C@@H]([C@@H](C)CC)C(=O)N[C@@H](Cc1c[nH]c2ccccc12)C(=O)NCC(=O)N[C@@H](CCCCN)C(=O)O | Integrase |
| CHEMBL189718 | Cn1c(cc2cc3OCOc3cc12)\C(=C\C(=O)C(=O)O)\O | Integrase |
| CHEMBL197036 | OC(=O)\C=C\c1ccc(OC2=Cc3cc(O)c(O)cc3OC2=O)c(O)c1 | Integrase |
| CHEMBL414737 | CC[C@H](C)C(NC(=O)[C@H](CCC(=O)O)NC(=O)[C@H](CC(C)C)NC(=O)[C@H](CC(=O)O)NC(=O)[C@H](CO)NC(=O)CNC(=O)[C@@H](NC(=O)[C@H](Cc1ccc(O)cc1)NC(=O)[C@H](CC(C)C)NC(=O)[C@H](CC(=O)O)NC(=O)[C@H](CC(=O)O)NC(=O)[C@H](CCSC)NC(=O)[C@H](Cc2ccc(O)cc2)NC(=O)[C@H](CCC(=O)N)NC(=O)[C@H](Cc3ccc(O)cc3)NC(=O)[C@@H](NC(=O)[C@@H](NC(=O)[C@@H](NC(=O)[C@H](CC(=O)O)NC(=O)[C@@H]4CCCN4)[C@@H](C)CC)C(C)C)[C@@H](C)CC)C(C)C)C(=O)O | Integrase |
| CHEMBL209591 | Oc1c(ccc2cccnc12)C(=O)NCc3ccc(F)cc3 | Integrase |
| CHEMBL197251 | OC1=Cc2cc(O)c(O)cc2OC1=O | Integrase |
| CHEMBL177705 | Oc1c2C(=O)N(Cc3ccc(OC(F)(F)F)cc3)C(=O)c2c(O)c4nccnc14 | Integrase |
| CHEMBL435337 | Oc1c2C(=O)N(CCc3ccccc3)C(=O)c2c(O)c4nccnc14 | Integrase |
| CHEMBL366712 | C[C@H](N1C(=O)c2c(O)c3nccnc3c(O)c2C1=O)c4ccccc4 | Integrase |
| CHEMBL40148 | OC(=O)c1ccc2ccc(\C=C\c3ccccc3)nc2c1O | Integrase |
| CHEMBL211275 | CCOC(=O)c1cccc(c1)c2c3CN(Cc4ccc(F)cc4)C(=O)c3c(O)c5ncccc25 | Integrase |
| CHEMBL3238124 | CCOC(=O)\C(=C\C(=O)C1=CN(Cc2ccc(F)cc2)c3cc(ccc3C1=O)N4CCOCC4)\O | Integrase |
| CHEMBL19574 | OC(=O)C(=O)CC(=O)c1cccc(c1)C(=O)c2ccccc2 | Integrase |
| CHEMBL66953 | OC1=CC=Cc2cc(O)c(O)c(O)c2C1=O | Integrase |
| CHEMBL108672 | Oc1ccc(cc1O)c2ccc3cc(O)c(O)cc3c2 | Integrase |
| CHEMBL284749 | CC(=O)Oc1ccc(\C=C\C(=O)OCC(OC(=O)\C=C\c2ccc(OC(=O)C)c(OC(=O)C)c2)C(=O)O)cc1OC(=O)C | Integrase |
| CHEMBL367772 | Oc1c2C(=O)N(CC3CCCCC3)C(=O)c2c(O)c4nccnc14 | Integrase |
| CHEMBL39883 | Nc1ccc(\C=C\c2ccc3ccc(C(=O)O)c(O)c3n2)cc1 | Integrase |
| CHEMBL97641 | Oc1ccc(\C=C\c2ccc3ccc(\C=C\c4ccc(O)c(O)c4)c(O)c3n2)cc1O | Integrase |
| CHEMBL3138268 | CC(CCC=C(c1cc(Cl)c(O)c(c1)C(=O)O)c2cc(Cl)c(O)c(c2)C(=O)O)[C@H]3CC[C@H]4[C@@H]5CC[C@@H]6CC(F)(F)CC[C@]6(C)[C@H]5CC[C@]34C | Integrase |
| CHEMBL3137943 | CC(CCC=C(c1cc(Cl)c(O)c(c1)C(=O)O)c2cc(Cl)c(O)c(c2)C(=O)O)[C@H]3CC[C@H]4[C@@H]5CC[C@@H]6CCCC[C@]6(C)[C@H]5CC[C@]34C | Integrase |
| CHEMBL129510 | Oc1ccc(\C=C\C(=O)c2ccccc2)cc1O | Integrase |
| CHEMBL428673 | CC[C@H](C)[C@@H](N)C(=O)N[C@H](CC(C)C)C(=O)N1CCC[C@@H]1C(=O)N[C@H](Cc2c[nH]c3ccccc23)C(=O)N[C@H](CCCCN)C(=O)N[C@H](Cc4c[nH]c5ccccc45)C(=O)N6CCC[C@@H]6C(=O)N[C@H](Cc7c[nH]c8ccccc78)C(=O)N[C@H](Cc9c[nH]c%10ccccc9%10)C(=O)N%11CCC[C@@H]%11C(=O)N[C@H](Cc%12c[nH]c%13ccccc%12%13)C(=O)N%14CCC[C@@H]%14C(=O)N%15CCC[C@@H]%15C(=O)NCCCCC(NC(=O)[C@H]%16CCCN%16C(=O)[C@H]%17CCCN%17C(=O)[C@@H](Cc%18c[nH]c%19ccccc%18%19)NC(=O)[C@H]%20CCCN%20C(=O)[C@@H](Cc%21c[nH]c%22ccccc%21%22)NC(=O)[C@@H](Cc%23c[nH]c%24ccccc%23%24)NC(=O)[C@H]%25CCCN%25C(=O)[C@@H](Cc%26c[nH]c%27ccccc%26%27)NC(=O)[C@@H](CCCCN)NC(=O)[C@@H](Cc%28c[nH]c%29ccccc%28%29)NC(=O)[C@H]%30CCCN%30C(=O)[C@@H](CC(C)C)NC(=O)[C@H](N)[C@@H](C)CC)C(=O)N | Integrase |
| CHEMBL541123 | Cl.C[C@@H]1O[C@H](C[C@H](N)[C@@H]1O)OC2C[C@@](O)(Cc3c(O)c4C(=O)c5cccc(O)c5C(=O)c4c(O)c23)C(=O)CO | Integrase |
| CHEMBL149475 | OC(=O)C(OC(=O)CCc1ccc(O)c(O)c1)C(OC(=O)CCc2ccc(O)c(O)c2)C(=O)O | Integrase |
| CHEMBL176023 | Oc1c2C(=O)N(C3CCN(CC3)C(=O)c4ccccc4)C(=O)c2c(O)c5nccnc15 | Integrase |
| CHEMBL3238122 | CCOC(=O)\C(=C\C(=O)C1=CN(Cc2ccc(F)cc2)c3cc(ccc3C1=O)N4CCN(CC)CC4)\O | Integrase |
| CHEMBL58057 | Oc1ccc(\C=C\c2ccc3cccc(O)c3n2)cc1O | Integrase |
| CHEMBL212320 | CN(C)C(=O)Oc1c2C(=O)N(Cc3ccc(F)cc3)C(=O)c2c(O)c4ncccc14 | Integrase |
| CHEMBL152850 | O=C1NNC(=O)c2ccccc2SSc3ccccc3C(=O)NNC(=O)c4ccccc4SSc5ccccc15 | Integrase |
| CHEMBL36586 | OC(=O)c1ccc2ccc(\C=C\c3ccc(O)cc3O)nc2c1O | Integrase |
| CHEMBL30434 | COC(=O)C(COC(=O)\C=C\c1ccc(OC(=O)C)c(OC(=O)C)c1)NC(=O)\C=C\c2ccc(OC(=O)C)c(OC(=O)C)c2 | Integrase |
| CHEMBL3233902 | Cc1cc(C)c(Cn2cc(C(=O)\C=C(/O)\C(=O)O)c3cc(F)ccc23)c(C)c1 | Integrase |
| CHEMBL427235 | CC(C)C[C@H](NC(=O)[C@H](CCCCN)NC(=O)[C@H](CC(C)C)NC(=O)[C@H](CC(C)C)NC(=O)[C@H](Cc1ccccc1)NC(=O)[C@H](Cc2ccc(O)cc2)NC(=O)[C@H](C)NC(=O)[C@@H](N)[C@@H](C)O)C(=O)N[C@@H](C)C(=O)NCC(=O)N[C@@H](CCCN=C(N)N)C(=O)N[C@@H](Cc3c[nH]c4ccccc34)C(=O)N | Integrase |
| CHEMBL382139 | OC(=O)C(=O)\C=C(/O)\C1=Nc2c(ncn2Cc3ccccc3)C(=O)N1Cc4ccccc4 | Integrase |
| CHEMBL66033 | OC1=C(C(C2=C(O)C(=O)c3ccccc3C2=O)c4ccc(cc4)C(C5=C(O)C(=O)c6ccccc6C5=O)C7=C(O)C(=O)c8ccccc8C7=O)C(=O)c9ccccc9C1=O | Integrase |
| CHEMBL87771 | O\C(=C/C(=O)C(=O)NCCCCCCNC(=O)C(=O)\C=C(/O)\c1cccc(Cc2ccccc2F)c1)\c3cccc(Cc4ccccc4F)c3 | Integrase |
| CHEMBL367939 | CC(=O)Nc1ccc(N)c2cc(ccc12)S(=O)(=O)O | Integrase |
| CHEMBL3222021 | CCCCCCCC[N+](C)(CCCCCCCC)CCCCCCCC.[O-]C(=O)C(F)(F)F | Integrase |
| CHEMBL40170 | COc1cc(\C=C\c2ccc3ccc(C(=O)O)c(O)c3n2)ccc1O | Integrase |
| CHEMBL440629 | CC[C@H](C)[C@H](N)C(=O)N[C@@H](CC(C)C)C(=O)N1CCC[C@H]1C(=O)N[C@@H](Cc2c[nH]c3ccccc23)C(=O)N[C@@H](CCCCN)C(=O)N[C@@H](Cc4c[nH]c5ccccc45)C(=O)N6CCC[C@H]6C(=O)N[C@@H](Cc7c[nH]c8ccccc78)C(=O)N[C@@H](Cc9c[nH]c%10ccccc9%10)C(=O)N%11CCC[C@H]%11C(=O)N[C@@H](Cc%12c[nH]c%13ccccc%12%13)C(=O)N%14CCC[C@H]%14C(=O)N%15CCC[C@H]%15C(=O)NCCCCC(NC(=O)[C@@H]%16CCCN%16C(=O)[C@@H]%17CCCN%17C(=O)[C@H](Cc%18c[nH]c%19ccccc%18%19)NC(=O)[C@@H]%20CCCN%20C(=O)[C@H](Cc%21c[nH]c%22ccccc%21%22)NC(=O)[C@H](Cc%23c[nH]c%24ccccc%23%24)NC(=O)[C@@H]%25CCCN%25C(=O)[C@H](Cc%26c[nH]c%27ccccc%26%27)NC(=O)[C@H](CCCCN)NC(=O)[C@H](Cc%28c[nH]c%29ccccc%28%29)NC(=O)[C@@H]%30CCCN%30C(=O)[C@H](CC(C)C)NC(=O)[C@@H](N)[C@@H](C)CC)C(=O)N | Integrase |
| CHEMBL34400 | COC(=O)[C@@H](COC(=O)\C=C\c1ccc(OC(=O)C)c(OC(=O)C)c1)NC(=O)\C=C\c2ccc(OC(=O)C)c(OC(=O)C)c2 | Integrase |
| CHEMBL30922 | COC(=O)[C@H](COC(=O)\C=C\c1ccc(OC(=O)C)c(OC(=O)C)c1)NC(=O)\C=C\c2ccc(OC(=O)C)c(OC(=O)C)c2 | Integrase |
| CHEMBL3238125 | CCOC(=O)\C(=C\C(=O)C1=CN(Cc2ccc(F)cc2)c3cc(ccc3C1=O)N(C)C)\O | Integrase |
| CHEMBL76984 | CCCCC(C)\C=C(/C)\C(=O)O[C@@H]1CC[C@H](C(=O)O)[C@@]2(C)C[C@@H](C(=C)C=O)C(=O)C=C12 | Integrase |
| CHEMBL310321 | OC(=O)c1ccc2ccc(nc2c1O)C(=O)NNc3ccc(cc3[N+](=O)[O-])[N+](=O)[O-] | Integrase |
| CHEMBL2370473 | CC[C@H](C)[C@H](NC(=O)[C@H](Cc1ccccc1)NC(=O)[C@@H](NC(=O)[C@H](C)NC(=O)[C@H](CCSC)NC(=O)[C@H](CCC(=O)N)NC(=O)[C@@H](NC(=O)[C@H](C)NC(=O)[C@@H](NC(=O)[C@H](CCCCN)NC(=O)[C@H](CC(C)C)NC(=O)[C@@H](N)Cc2c[nH]cn2)[C@@H](C)O)C(C)C)C(C)C)C(=O)N[C@@H](Cc3c[nH]cn3)C(=O)N[C@@H](CC(=O)N)C(=O)N[C@@H](Cc4ccccc4)C(=O)N[C@@H](CCCCN)C(=O)N[C@@H](CCCN=C(N)N)C(=O)N | Integrase |
| CHEMBL186055 | CCn1cc(\C(=C\C(=O)C(=O)O)\O)c2ccccc12 | Integrase |
| CHEMBL188631 | Cn1cc(\C(=C\C(=O)C(=O)O)\O)c2cc3OCOc3cc12 | Integrase |
| CHEMBL159546 | C[C@@H]1Oc2cc3c(c(O)cc(O)c3c(O)c2C(=O)[C@@H]1C)c4c(O)cc(O)c5c(O)c6OC(=O)[C@H](C)[C@@H](C)Oc6cc45 | Integrase |
| CHEMBL83598 | Oc1ccc(C=C2C(=O)c3ccccc3C2=O)cc1O | Integrase |
| CHEMBL80568 | CCCCC(C)\C=C(/C)\C(=O)O[C@@H]1CC[C@H](C(=O)N[C@@H](CC(C)C)C(=O)OC)[C@@]2(C)C[C@@H](C(=C)C=O)C(=O)C=C12 | Integrase |
| CHEMBL287903 | OC(=O)c1ccc2ccc(\C=C\c3ccsc3)nc2c1O | Integrase |
| CHEMBL373670 | CC[C@H](C)[C@H](NC(=O)[C@H](CC(C)C)NC(=O)[C@H](CC(C)C)NC(=O)[C@H](Cc1ccccc1)NC(=O)[C@H](Cc2ccc(O)cc2)NC(=O)[C@H](C)NC(=O)[C@@H](N)[C@@H](C)O)C(=O)N[C@@H](CC(C)C)C(=O)N[C@@H](C)C(=O)NCC(=O)N[C@@H](CCCN=C(N)N)C(=O)N[C@@H](Cc3c[nH]c4ccccc34)C(=O)N | Integrase |
| CHEMBL18266 | Oc1ccc(CCNC(=O)CCc2ccc(O)c(O)c2)cc1O | Integrase |
| CHEMBL209827 | OC(=O)C(=O)CC(=O)CC(=O)c1ccccc1F | Integrase |
| CHEMBL361659 | CCn1cc(\C(=C\C(=O)C(=O)O)\O)c2cc3OCOc3cc12 | Integrase |
| CHEMBL59446 | Oc1ccc(\C=C\c2ccc3ccc(C#N)c(O)c3n2)cc1O | Integrase |
| CHEMBL67535 | Oc1ccc(\C=C(/C#N)\C(=O)NCCCNC(=O)\C(=C\c2ccc(O)c(O)c2)\C#N)cc1O | Integrase |
| CHEMBL319488 | Oc1ccc(\C=C\C(=O)CC(=O)\C=C\c2ccc(O)c(O)c2)cc1O | Integrase |
| CHEMBL3259888 | Cc1nc2ccccc2c(c1CC(=O)O)c3ccc(Cl)cc3 | Integrase |
| CHEMBL3259892 | CO[C@@H](C(=O)O)c1c(C)nc2ccc(Br)cc2c1c3ccc(Cl)cc3 | Integrase |
| CHEMBL3286442 | ON1C(=O)N=C(NCc2ccc(F)cc2)c3cccnc13 | Integrase |
| CHEMBL39958 | OC(=O)c1ccc2ccc(\C=C\c3cc(O)cc(O)c3)nc2c1O | Integrase |
| CHEMBL157373 | Sc1ccccc1C(=O)NNC(=O)c2ccccc2SSc3ccccc3C(=O)NNC(=O)c4ccccc4S | Integrase |
| CHEMBL340697 | Cc1cc(c(S)cc1Cl)S(=O)(=O)Nc2nnc3c4c(C)csc4ncn23 | Integrase |
| CHEMBL267212 | CC(C)C[C@@H](NC(=O)[C@@H](C)NC(=O)CNC(=O)[C@@H](CCCN=C(N)N)NC(=O)[C@H](N)Cc1c[nH]c2ccccc12)C(=O)N[C@H](CCCCN)C(=O)N[C@H](CC(C)C)C(=O)N[C@H](CC(C)C)C(=O)N[C@H](Cc3ccccc3)C(=O)N[C@H](Cc4ccc(O)cc4)C(=O)N[C@H](C)C(=O)N[C@H]([C@H](C)O)C(=O)O | Integrase |
| CHEMBL130024 | Cc1cc(c(S)cc1Cl)S(=O)(=O)\N=C(/NN)\Nc2ccc(Cl)cc2 | Integrase |
| CHEMBL309502 | OC1=C(C(C2=C(O)Oc3cc(O)ccc3C2=O)c4ccc5ccccc5c4)C(=O)c6ccc(O)cc6O1 | Integrase |
| CHEMBL360065 | Oc1c2C(=O)N(Cc3cccc(Br)c3)C(=O)c2c(O)c4nnccc14 | Integrase |
| CHEMBL284384 | OC1=C(C(C2=C(O)Oc3ccccc3C2=O)c4ccc(\C=C\c5ccccc5)cc4)C(=O)c6ccccc6O1 | Integrase |
| CHEMBL84518 | OC1=C(C(C2=C(O)Oc3ccccc3C2=O)c4cc(OCc5ccccc5)cc(OCc6ccccc6)c4)C(=O)c7ccccc7O1 | Integrase |
| CHEMBL139149 | Oc1ccc(cc1O)C2=C(C(=O)OC2=O)c3ccc(O)c(O)c3 | Integrase |
| CHEMBL58 | OCCNCCNc1ccc(NCCNCCO)c2C(=O)c3c(O)ccc(O)c3C(=O)c12 | Integrase |
| CHEMBL3290939 | ON1C(=O)N=C(NCC2CC2)c3cccnc13 | Integrase |
| CHEMBL338228 | Cc1ccc(cc1)n2cnnc2NS(=O)(=O)c3cc(C(=O)Nc4ccc(Cl)cc4)c(Cl)cc3S | Integrase |
| CHEMBL113197 | CCCCCC=C(c1cc(Cl)c(O)c(c1)C(=O)NO)c2cc(Cl)c(O)c(c2)C(=O)NO | Integrase |
| CHEMBL131904 | Oc1ccc(CCC(=O)NCCc2cc(O)c(O)c(O)c2)cc1O | Integrase |
| CHEMBL37845 | OC(=O)c1ccc2ccc(\C=C\c3cc(O)c(O)c(I)c3)nc2c1O | Integrase |
| CHEMBL191020 | Oc1ccc(\C=C\S(=O)(=O)CS(=O)(=O)\C=C\c2ccc(O)c(O)c2)cc1O | Integrase |
| CHEMBL174150 | Oc1c(Cl)c(c(Nc2c(c(Cl)c(O)c(Cl)c2[N+](=O)[O-])[N+](=O)[O-])c(c1Cl)[N+](=O)[O-])[N+](=O)[O-] | Integrase |
| CHEMBL324842 | OC(=O)[C@@H](Cc1ccc(O)c(O)c1)OC(=O)\C=C\c2ccc(O)c(O)c2 | Integrase |
| CHEMBL208806 | COc1ccccc1C(=O)CC(=O)CC(=O)C(=O)O | Integrase |
| CHEMBL39143 | OC(=O)c1ccc2ccc(\C=C\c3cccnc3)nc2c1O | Integrase |
| CHEMBL29831 | CC(=O)Oc1ccc(\C=C\C(=O)NCC(NC(=O)\C=C\c2ccc(OC(=O)C)c(OC(=O)C)c2)C(=O)O)cc1OC(=O)C | Integrase |
| CHEMBL206981 | OC(=O)C(=O)\C=C(\O)/c1nc2C(=O)N(Cc3ccccc3)C=Nc2n1Cc4ccccc4 | Integrase |
| CHEMBL129147 | Cc1cc2nc3nc(N)nc(NS(=O)(=O)c4cc(C)c(Cl)cc4S)n3c2cc1C | Integrase |
| CHEMBL177021 | COC(=O)c1c(C)c2Oc3c(C=O)c(O)cc(C)c3C(=O)Oc2c(C)c1O | Integrase |
| CHEMBL37431 | COc1cc(\C=C\c2ccc3ccc(C(=O)O)c(O)c3n2)cc(OC)c1O | Integrase |
| CHEMBL366861 | Cc1cc(O)c(C=O)c2Oc3c(C)c(C(=O)O)c(O)c(COC(=O)\C=C\C(=O)O)c3OC(=O)c12 | Integrase |
| CHEMBL366410 | Cc1cc(O)c(C=O)c2Oc3c(C)c(C(=O)O)c(O)c(C)c3OC(=O)c12 | Integrase |
| CHEMBL30455 | CC(=O)Oc1ccc(\C=C\C(=O)O[C@@H]([C@H](OC(=O)\C=C\c2ccc(OC(=O)C)c(OC(=O)C)c2)C(=O)O)C(=O)O)cc1OC(=O)C | Integrase |
| CHEMBL18630 | OC(=O)c1ccc2cc(O)c(O)cc2c1 | Integrase |
| CHEMBL80419 | CCCCC(C)\C=C(/C)\C(=O)O[C@@H]1CC[C@H](C(=O)ON[C@@H](C(=O)OC)c2ccccc2)[C@@]3(C)C[C@@H](C(=C)C=O)C(=O)C=C13 | Integrase |
| CHEMBL210977 | OC(=O)\C(=C\C(=O)CCc1ccccc1Br)\O | Integrase |
| CHEMBL128547 | OCCN(CCO)S(=O)(=O)c1ccc(Nc2ccc(O)c3C(=O)c4ccccc4C(=O)c23)cc1 | Integrase |
| CHEMBL81182 | OC(=O)c1ccc2ccc(nc2c1O)C(=O)NCc3ccc(O)c(O)c3 | Integrase |
| CHEMBL157428 | Sc1ccccc1C(=O)NNC(=O)c2ccccc2S | Integrase |
| CHEMBL183469 | CCn1c(cc2cc3OCOc3cc12)\C(=C\C(=O)C(=O)O)\O | Integrase |
| CHEMBL68073 | C[Si](C)(C)C | Integrase |
| CHEMBL80259 | CCCCC(C)\C=C(/C)\C(=O)O[C@@H]1CC[C@H](C(=O)ON[C@H](C(=O)OC)c2ccccc2)[C@@]3(C)C[C@@H](C(=C)C=O)C(=O)C=C13 | Integrase |
| CHEMBL82633 | OC(=O)c1ccc2ccc(nc2c1O)C(=O)NCc3cccc(O)c3O | Integrase |
| CHEMBL191445 | CC(=O)c1cc(\C=C\S(=O)(=O)CS(=O)(=O)\C=C\c2cc(C(=O)C)c(C(=O)C)c(c2)C(=O)C)cc(C(=O)C)c1C(=O)C | Integrase |
| CHEMBL81714 | OC(=O)c1ccc2ccc(nc2c1O)C(=O)NCc3ccc(O)cc3O | Integrase |
| CHEMBL6246 | Oc1cc2C(=O)Oc3c(O)c(O)cc4C(=O)Oc(c1O)c2c34 | Integrase |
| CHEMBL3259885 | Cc1nc2ccc(Br)cc2c(c1CC(=O)O)c3ccccc3 | Integrase |
| CHEMBL433983 | Oc1ccccc1CNNC(=O)c2ccccc2O | Integrase |
| CHEMBL280299 | OC(=O)C(=O)CC(=O)c1cccc(c1)N=[N+]=[N-] | Integrase |
| CHEMBL2370433 | NCCCC[C@H](NC(=O)[C@H](CSCSC[C@H](NC(=O)[C@@H](N)Cc1c[nH]cn1)C(=O)N[C@@H](CCCCN)C(=O)N[C@@H](Cc2ccccc2)C(=O)N[C@@H](Cc3c[nH]c4ccccc34)C(=O)N[C@@H](Cc5c[nH]c6ccccc56)C(=O)N)NC(=O)[C@@H](N)Cc7c[nH]cn7)C(=O)N[C@@H](Cc8ccccc8)C(=O)N[C@@H](Cc9c[nH]c%10ccccc9%10)C(=O)N[C@@H](Cc%11c[nH]c%12ccccc%11%12)C(=O)N | Integrase |
| CHEMBL342488 | OC(=O)c1[nH]c(C(=O)O)c(c2ccc(O)c(O)c2)c1c3ccc(O)c(O)c3 | Integrase |
| CHEMBL176570 | COc1cc(C(=O)O)c2Oc3c(C=O)c(O)cc(C)c3C(=O)Oc2c1C | Integrase |
| CHEMBL418300 | COC1O[C@H](CO)[C@@H](O)[C@H](OC(=O)c2cc(O)c(O)c(O)c2)[C@H]1OC(=O)c3cc(O)c(O)c(O)c3 | Integrase |
| CHEMBL369471 | Oc1ccc(C[C@H]2COC(=O)[C@@H]2Cc3ccc(O)c(O)c3)cc1O | Integrase |
| CHEMBL147209 | OC(=O)C(=O)CC(=O)c1ccccc1Cc2ccccc2 | Integrase |
| CHEMBL425852 | Oc1ccccc1C(=O)NNC(=O)c2ccccc2S | Integrase |
| CHEMBL1160386 | NC1=NC(=O)N(C=C1)[C@H]2C[C@H](OP(=O)(O)OC[C@H]3OC[C@H]([C@@H]3O)N4C=CC(=O)NC4=O)[C@@H](COP(=O)(O)O)O2 | Integrase |
| CHEMBL210373 | OC(=O)C(=O)CC(=O)CC(=O)c1ccc2ccccc2c1 | Integrase |
| CHEMBL264263 | Oc1ccc(NC(=O)c2cccc3ccccc23)cc1c4oc5ccccc5n4 | Integrase |
| CHEMBL209879 | OC(=O)C(=O)CC(=O)CC(=O)c1ccccc1C(F)(F)F | Integrase |
| CHEMBL381234 | COc1ccc(cc1[N+](=O)[O-])C(=O)Nc2ccc(O)c(c2)c3oc4ccccc4n3 | Integrase |
| CHEMBL193783 | CCc1cc(\C=N\CC\N=C\c2cc(CC)sc2S)c(S)s1 | Integrase |
| CHEMBL365421 | Cn1c(cc2ccccc12)\C(=C\C(=O)C(=O)O)\O | Integrase |
| CHEMBL128376 | Cc1ccc(cc1)n2cnnc2NS(=O)(=O)c3cc(C(=O)Nc4ccccc4)c(Cl)cc3S | Integrase |
| CHEMBL127330 | Sc1cc(Cl)c(cc1S(=O)(=O)Nc2nncn2c3ccc(Cl)cc3)C(=O)Nc4ccccc4 | Integrase |
| CHEMBL369379 | OC(=O)c1cnc2c(ccc3ncc(C(=O)O)c(O)c23)c1O | Integrase |
| CHEMBL20586 | OC(=O)C(=O)CC(=O)c1ccc(CN=[N+]=[N-])cc1 | Integrase |
| CHEMBL3185210 | CCCCCCCC[N+](C)(CCCCCCCC)CCCCCCCC.[O-]S(=O)(=O)C(F)(F)F | Integrase |
| CHEMBL29369 | CC(=O)Oc1ccc(\C=C\C(=O)O[C@H]([C@@H](OC(=O)\C=C\c2ccc(OC(=O)C)c(OC(=O)C)c2)C(=O)O)C(=O)O)cc1OC(=O)C | Integrase |
| CHEMBL172439 | Cc1cc(O)c(C=O)c2Oc3c(OC(=O)c12)c(CO)c(O)c4C(=O)OC(O)c34 | Integrase |
| CHEMBL129778 | COc1cccc(Nc2n[nH]c(NS(=O)(=O)c3cc(C)c(Cl)cc3S)n2)c1 | Integrase |
| CHEMBL128608 | Cc1cc(cc(n1)n2nc(NS(=O)(=O)c3cc(C)c(Cl)cc3S)nc2N)C(F)(F)F | Integrase |
| CHEMBL31044 | Oc1ccc(\C=C\C(=O)NCCOC(=O)\C=C\c2ccc(O)c(O)c2)cc1O | Integrase |
| CHEMBL432793 | OC(=O)C(=O)\C=C(/O)\c1cc(\C(=C\C(=O)C(=O)O)\O)c2ccccc2n1 | Integrase |
| CHEMBL80008 | COc1cc(CNC(=O)c2ccc3ccc(C(=O)O)c(O)c3n2)cc(O)c1O | Integrase |
| CHEMBL83746 | COc1cc(NC(=O)c2ccc3ccc(C(=O)O)c(O)c3n2)cc(O)c1O | Integrase |
| CHEMBL561679 | CCCCCCCC[N+](C)(CCCCCCCC)CCCCCCCC.FC(F)(F)S(=O)(=O)[N-]S(=O)(=O)C(F)(F)F | Integrase |
| CHEMBL369808 | Oc1c2C(=O)N(Cc3ccncc3)C(=O)c2c(O)c4nccnc14 | Integrase |
| CHEMBL146179 | OC(=O)c1cc(OC(=O)\C=C\c2ccc(O)c(O)c2)cc(OC(=O)\C=C\c3ccc(O)c(O)c3)c1 | Integrase |
| CHEMBL108150 | CCOC(=O)C(=O)\C=C(/O)\c1c[nH]c2ccc(Cl)cc12 | Integrase |
| CHEMBL371519 | CCC(=O)C(=O)\C=C(/O)\c1c[nH]c2ccc(Cl)cc12 | Integrase |
| CHEMBL412074 | CC[C@H](C)[C@H](NC(=O)[C@H](CC(C)C)NC(=O)[C@H](CCC(=O)N)NC(=O)[C@H](CCC(=O)O)NC(=O)[C@@H](NC(=O)[C@@H](NC(=O)[C@H](CCC(=O)N)NC(=O)[C@H](CC(=O)N)NC(=O)[C@@H](NC(=O)[C@H](CC(C)C)NC(=O)[C@@H](N)CCC(=O)O)C(C)C)[C@@H](C)CC)[C@@H](C)CC)C(=O)N[C@@H](CCCCN)C(=O)N[C@@H](CCCCN)C(=O)N[C@@H](CCC(=O)O)C(=O)N[C@@H](CCCCN)C(=O)N[C@@H](C(C)C)C(=O)N[C@@H](Cc1ccc(O)cc1)C(=O)N[C@@H](CC(C)C)C(=O)N[C@@H](C)C(=O)N[C@@H](Cc2c[nH]c3ccccc23)C(=O)O | Integrase |
| CHEMBL141281 | OC(=O)c1cc(cc(Cl)c1O)C(=CCC23CC4CC(CC(C4)C2)C3)c5cc(Cl)c(O)c(c5)C(=O)O | Integrase |
| CHEMBL319244 | Oc1ccc(\C=C\C(=O)OCCc2ccccc2)cc1O | Integrase |
| CHEMBL377263 | OC(=O)C(=O)CC(=O)CC(=O)c1ccccc1OCc2ccccc2 | Integrase |
| CHEMBL49201 | OC(=O)C(=O)CC(=O)\C=C\c1cccn1Cc2ccccc2 | Integrase |
| CHEMBL3288714 | Oc1c(O)c2C(=O)C=C(Oc2cc1OCc3ccc(F)cc3)c4ccccc4 | Integrase |
| CHEMBL198442 | OC(=O)c1cc(NC(=O)Cc2ccccc2)c(cc1NC(=O)Cc3ccccc3)C(=O)O | Integrase |
| CHEMBL285489 | CC1=NN(C(=O)c2ccccc2O)C(=O)/C/1=C\c3cccc(O)c3 | Integrase |
| CHEMBL337606 | Cc1cc(c(S)cc1Cl)S(=O)(=O)Nc2nnc3ccc4ccccc4n23 | Integrase |
| CHEMBL131784 | Cc1cc(c(S)cc1Cl)S(=O)(=O)Nc2nnc3c4scc(C)c4ncn23 | Integrase |
| CHEMBL48381 | CCOC(=O)C(=O)CC(=O)\C=C\c1cccn1Cc2ccccc2 | Integrase |
| CHEMBL31026 | Oc1ccc(\C=C\C(=O)OCCCOC(=O)\C=C\c2ccc(O)c(O)c2)cc1O | Integrase |
| CHEMBL128540 | CC(=O)Oc1cc(c(S)cc1Cl)S(=O)(=O)Nc2n[nH]c(Nc3ccc(C)cc3)n2 | Integrase |
| CHEMBL31607 | Oc1ccc(\C=C\C(=O)O[C@H]2CCCC[C@@H]2OC(=O)\C=C\c3ccc(O)c(O)c3)cc1O | Integrase |
| CHEMBL433666 | Cc1cc(c(S)cc1Cl)S(=O)(=O)Nc2nc(N)nc3nc4ccccc4n23 | Integrase |
| CHEMBL108423 | OC(=O)C(=O)\C=C(/O)\c1ccc(Oc2ccccc2)cc1 | Integrase |
| CHEMBL31883 | C[C@H](OC(=O)\C=C\c1ccc(O)c(O)c1)[C@H](C)OC(=O)\C=C\c2ccc(O)c(O)c2 | Integrase |
| CHEMBL310293 | OC1=C(C(C2=C(O)Oc3ccccc3C2=O)c4ccc5c(Cc6ccccc56)c4)C(=O)c7ccccc7O1 | Integrase |
| CHEMBL444478 | C[C@@H]1O[C@@H](O[C@@H]2[C@@H](O)[C@H](OCCc3ccc(O)c(O)c3)O[C@H](CO)[C@H]2OC(=O)\C=C\c4ccc(O)c(O)c4)[C@H](O)[C@H](O)[C@H]1O | Integrase |
| CHEMBL67233 | OC1CC(CC(O)C1OC(=O)\C=C/c2ccc(O)c(O)c2)(OC(=O)\C=C/c3ccc(O)c(O)c3)C(=O)O | Integrase |
| CHEMBL172418 | CCOC(=O)c1cnc(NCCCNc2ncc(C(=O)OCC)c(O)n2)nc1O | Integrase |
| CHEMBL3290938 | ON1C(=O)N=C(N2CCC(F)(F)CC2)c3cccnc13 | Integrase |
| CHEMBL128856 | Cc1cc(c(S)cc1Cl)S(=O)(=O)Nc2nc3ccccc3nc2c4cccc(c4)[N+](=O)[O-] | Integrase |
| CHEMBL129173 | Cc1cc(c(S)cc1Cl)S(=O)(=O)Nc2nc3ccccc3nc2c4ccc(Br)cc4 | Integrase |
| CHEMBL130134 | Cc1ccc(NC(=O)c2cc(c(S)cc2Cl)S(=O)(=O)Nc3nncn3Cc4ccccc4)cc1 | Integrase |
| CHEMBL353069 | COOC(=O)c1ccc2c(O)c(O)ccc2c1 | Integrase |
| CHEMBL131982 | Oc1ccc(\C=C\C(=O)OCCCc2ccccc2)cc1O | Integrase |
| CHEMBL430776 | [Na+].COc1ccc(cc1O)c2c3c4cc(OC)c(OS(=O)(=O)[O-])cc4OC(=O)c3n5ccc6cc(OC)c(OC)cc6c25 | Integrase |
| CHEMBL18276 | COC(=O)c1ccc2c(O)c(O)ccc2c1 | Integrase |
| CHEMBL180164 | O=C(NCc1occc1)c2cc(cc(c2)C(=O)NCc3occc3)C(=O)NCc4occc4 | Integrase |
| CHEMBL349440 | COc1cc(O)c2c(OC)c3C(=O)C(C)C(C)Oc3cc2c1c4c(O)cc(OC)c5c(OC)c6C(=O)C(C)C(C)Oc6cc45 | Integrase |
| CHEMBL210870 | OC(=O)C(=O)CC(=O)CC(=O)c1cccc(Cl)c1 | Integrase |
| CHEMBL128740 | NNS(=O)(=O)c1ccc(OCC(=O)O)cc1 | Integrase |
| CHEMBL129598 | Cc1cc(c(S)cc1Cl)S(=O)(=O)Nc2nnc3cnc4ccccc4n23 | Integrase |
| CHEMBL149305 | Oc1ccc(\C=C/C(=O)OC2CCCCC2OC(=O)\C=C/c3ccc(O)c(O)c3)cc1O | Integrase |
| CHEMBL31574 | OC1=C(Oc2cc(O)ccc2C1=O)c3ccc(O)c(O)c3 | Integrase |
| CHEMBL20702 | COc1ccc(cc1CN=[N+]=[N-])C(=O)CC(=O)C(=O)O | Integrase |
| CHEMBL81797 | OC1=C(C(C2=C(O)Oc3ccccc3C2=O)c4ccc(OCc5ccccc5)cc4)C(=O)c6ccccc6O1 | Integrase |
| CHEMBL133714 | Oc1ccc(\C=C\C(=O)OCc2ccccc2)cc1O | Integrase |
| CHEMBL129120 | Cc1cc(c(S)cc1Cl)S(=O)(=O)Nc2n[nH]c(n2)c3ccc(Cl)cn3 | Integrase |
| CHEMBL103410 | COc1cc(\C=C\C(=O)CC(=O)\C=C\c2ccc(O)c(O)c2)ccc1O | Integrase |
| CHEMBL203666 | NCCCNc1ccc2c3c(nn2CCNCCO)c4c(O)ccc(O)c4C(=O)c13 | Integrase |
| CHEMBL209205 | OC(=O)C(=O)CC(=O)CCc1ccccc1Cl | Integrase |
| CHEMBL316387 | OC1=C(C(C2=C(O)Oc3ccccc3C2=O)c4ccc5ccccc5c4)C(=O)c6ccccc6O1 | Integrase |
| CHEMBL1422607 | Cc1nc2ccc(Cl)cc2c(c1CC(=O)O)c3ccccc3 | Integrase |
| CHEMBL149306 | Oc1ccc(\C=C\C(=O)OCCCCOC(=O)\C=C\c2ccc(O)c(O)c2)cc1O | Integrase |
| CHEMBL177673 | Oc1c2C(=O)N(C(=O)c2c(O)c3nccnc13)c4ccc5OCOc5c4 | Integrase |
| CHEMBL206980 | OC(=O)C(=O)\C=C(\O)/c1nc2cncnc2n1Cc3ccccc3 | Integrase |
| CHEMBL198591 | COc1ccc2OC(=O)C(=Cc2c1)c3csc(N\N=C\c4ccccc4O)n3 | Integrase |
| CHEMBL281929 | COC(=O)[C@@H](CNC(=O)\C=C\c1ccc(OC(=O)C)c(OC(=O)C)c1)NC(=O)\C=C\c2ccc(OC(=O)C)c(OC(=O)C)c2 | Integrase |
| CHEMBL286494 | Cc1cc(O)c2C(=O)c3c(O)cc(O)c4c5c(O)cc(O)c6C(=O)c7c(O)cc(C)c8c1c2c(c34)c(c78)c56 | Integrase |
| CHEMBL307070 | CCCCC(C)\C=C(/C)\C(=O)O[C@@H]1CC[C@H](C(=O)NCCNC(=O)[C@H]2CC[C@@H](OC(=O)\C(=C\C(C)CCCC)\C)C3=CC(=O)[C@@H](C[C@]23C)C(=C)C=O)[C@@]4(C)C[C@@H](C(=C)C=O)C(=O)C=C14 | Integrase |
| CHEMBL348709 | Oc1ccc(\C=C\C(=O)OC2CCCCC2OC(=O)\C=C\c3ccc(O)c(O)c3)cc1O | Integrase |
| CHEMBL425205 | Oc1ccc(NC(=O)c2cccnc2)cc1c3oc4ccc(Cl)cc4n3 | Integrase |
| CHEMBL380528 | Oc1ccc(cc1O)C(c2c[nH]c3ccccc23)c4c[nH]c5ccccc45 | Integrase |
| CHEMBL46745 | Fc1ccc(OCC(=O)Nc2ccccc2)c(c1)C(=O)c3ccccc3 | Reverse transcriptase |
| CHEMBL223228 | FC(F)(F)[C@]1(OC(=O)Nc2ccc(Cl)cc12)C#CC3CC3 | Reverse transcriptase |
| CHEMBL293498 | C[C@H]1CN2C(=S)Nc3cc(Cl)cc(CN1CC=C(C)C)c23 | Reverse transcriptase |
| CHEMBL288971 | Clc1ccc(OCC(=O)N2CCc3ccccc3C2)c(c1)C(=O)c4ccccc4 | Reverse transcriptase |
| CHEMBL54893 | CC(=CCOc1cc(NC(=S)c2ccoc2C)ccc1Cl)C | Reverse transcriptase |
| CHEMBL296939 | Clc1ccc(OCC(=O)Nc2ccccc2)c(c1)C(=O)c3ccccc3 | Reverse transcriptase |
| CHEMBL46925 | Clc1cc(OCC(=O)Nc2ccccc2)c(cc1Cl)C(=O)c3ccccc3 | Reverse transcriptase |
| CHEMBL417183 | Clc1ccc(OCC(=O)c2cc3ccccc3[nH]2)c(c1)C(=O)c4ccccc4 | Reverse transcriptase |
| CHEMBL46650 | CN(C)CCCOc1ccc(NC(=O)COc2ccc(Cl)cc2C(=O)c3ccccc3)cc1 | Reverse transcriptase |
| CHEMBL193952 | CC1=CN([C@H]2C[C@H](N=[N+]=[N-])[C@@H](COP(=O)(O)OP(=O)(O)OP(=O)(O)O)O2)C(=O)NC1=O | Reverse transcriptase |
| CHEMBL297010 | Clc1ccc(OCC(=O)Nc2ccccc2)c(c1)C(=O)c3cccnc3 | Reverse transcriptase |
| CHEMBL593 | CC(C)Nc1cccnc1N2CCN(CC2)C(=O)c3cc4cc(NS(=O)(=O)C)ccc4[nH]3 | Reverse transcriptase |
| CHEMBL43263 | CN(C)CCCOc1ccc(NC(=O)Cc2ccc(Cl)cc2C(=O)c3ccccc3)cc1 | Reverse transcriptase |
| CHEMBL42078 | CN(C)CCCOc1ccc(NC(=O)Cc2ccc(Cl)cc2C(=O)c3ccccc3)c(C)c1 | Reverse transcriptase |
| CHEMBL2115411 | CC1=CN([C@@H]2O[C@H](CO[Si](C)(C)C(C)(C)C)C3(OS(=O)(=O)C=C3N)[C@H]2O[Si](C)(C)C(C)(C)C)C(=O)NC1=O | Reverse transcriptase |
| CHEMBL54224 | NC1=Nc2c(ncn2[C@H]3CC[C@@H](COP(=O)(O)OP(=O)(O)OP(=O)(O)O)O3)C(=O)N1 | Reverse transcriptase |
| CHEMBL211582 | CN1C(=O)N(C=C(C)C1=O)[C@@H]2O[C@H](CO[Si](C)(C)C(C)(C)C)[C@@]3(OS(=O)(=O)C=C3N)[C@H]2O[Si](C)(C)C(C)(C)C | Reverse transcriptase |
| CHEMBL45100 | CCN(CC)CCOc1ccc(NC(=O)COc2ccc(Cl)cc2C(=O)c3ccccc3)cc1 | Reverse transcriptase |
| CHEMBL1383 | Nc1ncnc2c1ncn2[C@H]3CC[C@@H](COP(=O)(O)OP(=O)(O)OP(=O)(O)O)O3 | Reverse transcriptase |
| CHEMBL44002 | Clc1ccc(OCc2nc3ccccc3[nH]2)c(c1)C(=O)c4ccccc4 | Reverse transcriptase |
| CHEMBL338099 | Cc1nc(CCCCCCC(=O)c2ccccc2)n3nc(ccc13)n4ccnc4 | Reverse transcriptase |
| CHEMBL135380 | Clc1ccc(NC(=S)NCCN2C(=O)c3ccccc3C2=O)nc1 | Reverse transcriptase |
| CHEMBL144035 | CCN1c2nc(ccc2N(C)C(=O)c3cccnc13)c4ccc(O)cc4 | Reverse transcriptase |
| CHEMBL88677 | Fc1cccc(F)c1CC\N=C(/S)\Nc2ccc(Br)cn2 | Reverse transcriptase |
| CHEMBL132756 | COc1ccc(F)c(CC\N=C(/S)\Nc2ccc(Br)cn2)c1C#N | Reverse transcriptase |
| CHEMBL81441 | CCN1c2ncccc2C(=O)N(C)c3ccc(nc13)c4c[nH]c5cnccc45 | Reverse transcriptase |
| CHEMBL268871 | CCc1cc(NCc2oc3c(Cl)ccc(Cl)c3n2)c(O)nc1C | Reverse transcriptase |
| CHEMBL253186 | COc1ccc(OC)c(CCNC(=S)Nc2ccc(Cl)cn2)c1F | Reverse transcriptase |
| CHEMBL57 | Cc1ccnc2N(C3CC3)c4ncccc4C(=O)Nc12 | Reverse transcriptase |
| CHEMBL131643 | CCOc1ccc(Cl)c(C2CC2NC(=O)Nc3ccc(Cl)cn3)c1F | Reverse transcriptase |
| CHEMBL37835 | CCOc1ccc(Cl)c([C@H]2C[C@H]2NC(=O)Nc3ccc(Cl)cn3)c1F | Reverse transcriptase |
| CHEMBL37024 | CCOc1ccc(F)c([C@H]2C[C@H]2NC(=O)Nc3ccc(Cl)cn3)c1F | Reverse transcriptase |
| CHEMBL541204 | CCOc1ccc(F)c(C2CC2NC(=O)Nc3ccc(Cl)cn3)c1F | Reverse transcriptase |
| CHEMBL42066 | Clc1ccc(OCC(=O)Nc2ccccc2)c(c1)C(=O)C3CCCC3 | Reverse transcriptase |
| CHEMBL103706 | Fc1cc(F)c(CC\N=C(/S)\Nc2ccc(Cl)cn2)c(F)c1 | Reverse transcriptase |
| CHEMBL436376 | CCOc1ccc(F)c(CC\N=C(/S)\Nc2ccc(Br)cn2)c1C#N | Reverse transcriptase |
| CHEMBL38341 | COc1ccc(OC)c([C@H]2C[C@H]2NC(=O)Nc3ccc(Cl)cn3)c1F | Reverse transcriptase |
| CHEMBL37762 | Fc1cccc(F)c1[C@H]2C[C@H]2NC(=O)Nc3ccc(Cl)cn3 | Reverse transcriptase |
| CHEMBL436177 | CCOc1ccc(Cl)c(CC\N=C(/S)\Nc2ccc(Br)cn2)c1F | Reverse transcriptase |
| CHEMBL341201 | Clc1ccc(NC(=S)NCCN2C(=O)c3c(Cl)ccc(Cl)c3C2=O)nc1 | Reverse transcriptase |
| CHEMBL162118 | CCN1c2nc(Cl)cc(C)c2NC(=O)c3cc(CSc4ccncc4)cnc13 | Reverse transcriptase |
| CHEMBL42823 | CCN(CC)CCOc1ccc(NC(=O)COc2ccccc2C(=O)c3ccccc3)cc1 | Reverse transcriptase |
| CHEMBL46977 | CCc1cc(OCCCN(C)C)ccc1NC(=O)Cc2ccc(Cl)cc2C(=O)c3ccccc3 | Reverse transcriptase |
| CHEMBL46675 | Cc1ccccc1CNC(=O)COc2ccc(Cl)cc2C(=O)c3ccccc3 | Reverse transcriptase |
| CHEMBL2407581 | CC[C@@H]1NC(=O)[C@H]([C@H](O)[C@H](C)C\C=C\C)N(C)C(=O)[C@H](C(C)C)N(C)C(=O)[C@H](CC(C)C)N(C)C(=O)[C@H](CC(C)C)N(C)C(=O)[C@@H](C)NC(=O)[C@H](C)NC(=O)[C@H](CC(C)C)N(C)C(=O)[C@@H](NC(=O)[C@H](CC(C)C)N(C)C(=O)[C@@H](SCCN(CC)CC)N(C)C1=O)C(C)C | Reverse transcriptase |
| CHEMBL134278 | CCC(=O)c1ccc(F)c(C2CC2NC(=O)Nc3ccc(Cl)cn3)c1O | Reverse transcriptase |
| CHEMBL133658 | CCOc1ccc(Cl)c(C2CC2NC(=O)Nc3ccc(cn3)C#N)c1F | Reverse transcriptase |
| CHEMBL334983 | COc1ccc(F)c(F)c1CC\N=C(/S)\Nc2ccc(Br)cn2 | Reverse transcriptase |
| CHEMBL129 | CC1=CN([C@H]2C[C@H](N=[N+]=[N-])[C@@H](CO)O2)C(=O)NC1=O | Reverse transcriptase |
| CHEMBL133195 | Brc1ccc(NC(=S)NCCn2nc3ccccc3n2)nc1 | Reverse transcriptase |
| CHEMBL328258 | COc1ccccc1C(=O)CC\N=C(/S)\Nc2ccc(Br)cn2 | Reverse transcriptase |
| CHEMBL85110 | CCC1=C(Sc2ccccc2)N(COCCOCc3ccccc3)C(=O)NC1=O | Reverse transcriptase |
| CHEMBL36930 | Fc1cccc(F)c1CCNC(=O)Nc2ccc(Cl)cn2 | Reverse transcriptase |
| CHEMBL287317 | COc1ccc(OC)c(CCNC(=O)Nc2ccc(Cl)cn2)c1F | Reverse transcriptase |
| CHEMBL88840 | Clc1ccc(NC(=S)NCCc2ccccc2)nc1 | Reverse transcriptase |
| CHEMBL100980 | CCN1c2nc(Cl)ccc2N(C)C(=O)c3cc(CSc4ccncc4)cnc13 | Reverse transcriptase |
| CHEMBL207839 | Cc1cc(C)c(c(C)c1)n2nnnc2SCC(=O)Nc3ccccc3[N+](=O)[O-] | Reverse transcriptase |
| CHEMBL435600 | COc1ccc(F)c(CC\N=C(/S)\Nc2ccc(cn2)C#N)c1C#N | Reverse transcriptase |
| CHEMBL91624 | Fc1cccc(F)c1C(=O)CC\N=C(/S)\Nc2ccc(Br)cn2 | Reverse transcriptase |
| CHEMBL207512 | Cc1cc(C)c(c(C)c1)n2nnnc2SCC(=O)Nc3ccc(cc3Cl)C(=O)N | Reverse transcriptase |
| CHEMBL382553 | Cc1c(Cl)cccc1n2nnnc2SCC(=O)Nc3ccccc3[N+](=O)[O-] | Reverse transcriptase |
| CHEMBL398766 | Cc1ccc(NC(=S)NCCc2c(F)cccc2F)nc1 | Reverse transcriptase |
| CHEMBL56135 | Cc1cc(Br)cc(c1)S(=O)(=O)c2cccc(N)c2C#N | Reverse transcriptase |
| CHEMBL381134 | Cc1ccc(c(Br)c1)n2nnnc2SCC(=O)Nc3ccccc3[N+](=O)[O-] | Reverse transcriptase |
| CHEMBL206522 | NS(=O)(=O)c1ccc(NC(=O)CSc2nnnn2c3cc(Cl)ccc3Cl)c(Cl)c1 | Reverse transcriptase |
| CHEMBL133559 | CC(=O)c1ccc(F)c(C2CC2NC(=O)Nc3ccc(Br)cn3)c1O | Reverse transcriptase |
| CHEMBL134026 | CCC(=O)c1ccc(F)c(C2CC2NC(=O)Nc3ccc(Br)cn3)c1O | Reverse transcriptase |
| CHEMBL207929 | [O-][N+](=O)c1ccccc1NC(=O)CSc2nnnn2c3cc(Cl)ccc3Cl | Reverse transcriptase |
| CHEMBL379722 | Cc1cc(C)c(c(C)c1)n2nnnc2SCC(=O)Nc3ccc(cc3Cl)S(=O)(=O)N | Reverse transcriptase |
| CHEMBL253169 | COCc1ccc(F)c(CCNC(=S)Nc2ccc(Br)cn2)c1F | Reverse transcriptase |
| CHEMBL36424 | CCOc1ccc(F)c(CCNC(=O)Nc2ccc(Br)cn2)c1Cl | Reverse transcriptase |
| CHEMBL252964 | CCOc1ccc(F)c(CCNC(=S)Nc2ccc(Br)cn2)c1F | Reverse transcriptase |
| CHEMBL205152 | Cc1cc(C)c(c(C)c1)n2nnnc2SCC(=O)Nc3ccc(Cl)cc3[N+](=O)[O-] | Reverse transcriptase |
| CHEMBL134630 | Fc1ccoc1CC\N=C(/S)\Nc2ccc(Cl)cn2 | Reverse transcriptase |
| CHEMBL91332 | Fc1ccccc1C(=O)CC\N=C(/S)\Nc2ccc(Br)cn2 | Reverse transcriptase |
| CHEMBL38318 | CCOc1ccc(F)c([C@H]2C[C@H]2NC(=O)Nc3ccc(cn3)C#N)c1Cl | Reverse transcriptase |
| CHEMBL206962 | [O-][N+](=O)c1ccccc1NC(=O)CSc2nnnn2c3c(Cl)cccc3Cl | Reverse transcriptase |
| CHEMBL421841 | CCN1c2nccc(C)c2NC(=O)c3cc(CSc4cc(C)nc(C)c4)cnc13 | Reverse transcriptase |
| CHEMBL1160964 | Nc1ncnc2c1nnn2C3COC(COP(=O)(O)OP(=O)(O)OP(=O)(O)O)C3 | Reverse transcriptase |
| CHEMBL340047 | CCOc1ccc(F)c(C2CC2NC(=O)Nc3ccc(cn3)C#N)c1Cl | Reverse transcriptase |
| CHEMBL399976 | CCOc1cccc(F)c1CCNC(=S)Nc2ccc(C)cn2 | Reverse transcriptase |
| CHEMBL236161 | CC(C1=C(C)C(=O)NC(=N1)SC2CCCC2)c3c(F)cccc3F | Reverse transcriptase |
| CHEMBL291983 | Cc1cc(Cl)cc(c1)S(=O)(=O)c2cccc(N)c2C#N | Reverse transcriptase |
| CHEMBL207920 | CN(C)CCCNC(=O)c1ccc(NC(=O)CSc2nnnn2c3c(C)cc(C)cc3C)c(Cl)c1 | Reverse transcriptase |
| CHEMBL207900 | [O-][N+](=O)c1ccccc1NC(=O)CSc2nnnn2c3cccc4ccccc34 | Reverse transcriptase |
| CHEMBL402265 | CCOc1ccc(F)c(F)c1CCNC(=S)Nc2ccc(Br)cn2 | Reverse transcriptase |
| CHEMBL400547 | CCc1csc(NC(=S)NCCc2c(F)cccc2F)n1 | Reverse transcriptase |
| CHEMBL377010 | Cc1cc(C)c(c(C)c1)n2nnnc2SCC(=O)Nc3ccccc3Cl | Reverse transcriptase |
| CHEMBL399503 | CCOc1ccc(OC)c(CCNC(=S)Nc2ccc(Cl)cn2)c1F | Reverse transcriptase |
| CHEMBL135254 | COc1ccc(F)c(CC\N=C(/S)\Nc2ccc(Cl)cn2)c1C#N | Reverse transcriptase |
| CHEMBL253822 | COc1ccc(OC)c(CCNC(=S)Nc2ccc(Cl)cn2)c1 | Reverse transcriptase |
| CHEMBL135259 | CCOc1ccc(F)c(CC\N=C(/S)\N[n+]2ccc(Cl)cn2)c1Cl | Reverse transcriptase |
| CHEMBL429488 | CCOc1ccc(F)c(CCNC(=S)Nc2ccc(cn2)C#N)c1Cl | Reverse transcriptase |
| CHEMBL306904 | CC1CN2C(=S)Nc3ccc(Cl)c(CN1CC=C(C)C)c23 | Reverse transcriptase |
| CHEMBL131972 | CCOc1ccc(Cl)c(C2CC2NC(=O)Nc3cc(Cl)cnn3)c1F | Reverse transcriptase |
| CHEMBL135105 | CC(=O)c1ccc(F)c(C2CC2NC(=O)Nc3ccc(cn3)C#N)c1O | Reverse transcriptase |
| CHEMBL253369 | CCOc1cccc(F)c1CCNC(=S)Nc2ccc(Br)cn2 | Reverse transcriptase |
| CHEMBL252137 | Fc1cccc(Cl)c1CCNC(=S)Nc2nccs2 | Reverse transcriptase |
| CHEMBL254007 | Fc1cccc(F)c1CCNC(=S)Nc2nc(cs2)C#N | Reverse transcriptase |
| CHEMBL436546 | CC(C)C1=C(Cc2ccccc2)N(COCc3ccccc3)C(=O)NC1=O | Reverse transcriptase |
| CHEMBL351728 | CCN1c2nc(Cl)cc(C)c2NC(=O)c3cc(CSc4nc(C)cc(C)n4)cnc13 | Reverse transcriptase |
| CHEMBL398890 | COc1ccc(F)c(CCNC(=S)Nc2ccc(Br)cn2)c1F | Reverse transcriptase |
| CHEMBL88710 | Fc1cccc(F)c1C(=O)CC\N=C(/S)\Nc2ccc(Cl)cn2 | Reverse transcriptase |
| CHEMBL253170 | CCOc1ccc(F)c(CCNC(=S)Nc2ccc(Cl)cn2)c1Cl | Reverse transcriptase |
| CHEMBL47214 | CCN(CC)CCOc1ccc(NC(=O)COc2cc(OC)ccc2C(=O)c3ccccc3)cc1 | Reverse transcriptase |
| CHEMBL132425 | CCC(=O)c1ccc(F)c(C2CC2NC(=O)Nc3ccc(cn3)C#N)c1O | Reverse transcriptase |
| CHEMBL415908 | CCN(CC)CCOc1ccc(NC(=O)COc2ccccc2C(=O)c3ccc(OC)cc3)cc1 | Reverse transcriptase |
| CHEMBL162383 | CCN1c2nc(F)cc(C)c2NC(=O)c3cc(CSc4cc(C)nc(C)c4)cnc13 | Reverse transcriptase |
| CHEMBL443063 | CN(C)c1ccc(F)c(CCNC(=S)Nc2ccc(Br)cn2)c1F | Reverse transcriptase |
| CHEMBL164261 | CCN1c2nc(Cl)cc(C)c2NC(=O)c3cc(CSc4cc(C)nc(C)c4)cnc13 | Reverse transcriptase |
| CHEMBL37834 | CCOc1ccc(OC)c([C@H]2C[C@H]2NC(=O)Nc3ccc(Br)cn3)c1F | Reverse transcriptase |
| CHEMBL299001 | Cc1cc(C)cc(c1)S(=O)(=O)c2cccc(N)c2C#N | Reverse transcriptase |
| CHEMBL137111 | COc1ccc(F)c(CC\N=C(/S)\Nc2ccc(Br)cn2)c1OC | Reverse transcriptase |
| CHEMBL381513 | Cc1cc(C)c(c(C)c1)n2nnnc2SCC(=O)Nc3ccccc3Br | Reverse transcriptase |
| CHEMBL422371 | CCN1c2ncc(CSc3oc4ccccc4n3)cc2C(=O)Nc5c(C)cc(Cl)nc15 | Reverse transcriptase |
| CHEMBL88729 | Brc1ccc(NC(=S)N[C@@H]2C[C@@H]2c3ccccc3)nc1 | Reverse transcriptase |
| CHEMBL83412 | CCc1cc(NCc2cc(C)c(C)cc2OC)c(O)nc1C | Reverse transcriptase |
| CHEMBL35033 | CCOCN1C(=O)NC(=O)C(=C1Cc2ccccc2)C(C)C | Reverse transcriptase |
| CHEMBL252963 | CN(C)c1ccc(F)c(CCNC(=S)Nc2ccc(Cl)cn2)c1F | Reverse transcriptase |
| CHEMBL37025 | CCOc1ccc(F)c([C@H]2C[C@H]2NC(=O)Nc3ccc(Cl)cn3)c1Cl | Reverse transcriptase |
| CHEMBL253171 | CCOc1ccc(F)c(CCNC(=S)Nc2ccc(I)cn2)c1Cl | Reverse transcriptase |
| CHEMBL35729 | CCOc1ccc(OC)c(CCNC(=O)Nc2ccc(Cl)cn2)c1F | Reverse transcriptase |
| CHEMBL135808 | CN(C)c1ccc(CC\N=C(/S)\Nc2ccc(Br)cn2)c(F)c1C#N | Reverse transcriptase |
| CHEMBL383088 | NS(=O)(=O)c1ccc(NC(=O)CSc2nnnn2c3cccc(Cl)c3Cl)c(Cl)c1 | Reverse transcriptase |
| CHEMBL84621 | CCc1cc(NCc2cc(C)c(C)nc2OC)c(O)nc1C | Reverse transcriptase |
| CHEMBL253018 | Cc1cnc([nH]1)c2[nH]c3ccc(Cl)cc3c2S(=O)(=O)c4ccccc4 | Reverse transcriptase |
| CHEMBL319224 | Fc1ccc(C#N)c(F)c1CC\N=C(/S)\Nc2ccc(Cl)cn2 | Reverse transcriptase |
| CHEMBL78882 | Clc1ccc2NC(=O)NC(C#Cc3occc3)(C4CC4)c2c1 | Reverse transcriptase |
| CHEMBL99235 | CCc1cc(CCc2oc3c(Cl)ccc(Cl)c3n2)c(O)nc1C | Reverse transcriptase |
| CHEMBL88397 | Clc1ccccc1C(=O)CCNC(=S)Nc2ccc(Br)cn2 | Reverse transcriptase |
| CHEMBL366017 | CCN1c2nc(Cl)ccc2N(C)C(=O)c3cc(CCOc4ccc(cc4C)C(=O)O)cnc13 | Reverse transcriptase |
| CHEMBL319277 | CCN1c2nc(Cl)ccc2N(C)C(=O)c3cc(COc4ccccc4)cnc13 | Reverse transcriptase |
| CHEMBL103386 | CCN1c2nc(Cl)ccc2N(C)C(=O)c3cc(CSc4ccccc4)cnc13 | Reverse transcriptase |
| CHEMBL102166 | CCN1c2ncccc2N(C)C(=O)c3cc(COc4ccncc4)cnc13 | Reverse transcriptase |
| CHEMBL100056 | CCN1c2ncc(CCc3cccc(N)c3)cc2C(=O)N(C)c4ccc(Cl)nc14 | Reverse transcriptase |
| CHEMBL289000 | O=C(COc1ccc(cc1C(=O)c2ccccc2)n3ccnc3)Nc4ccccc4 | Reverse transcriptase |
| CHEMBL69855 | C1CC1N2c3ncccc3C=Cc4cccnc24 | Reverse transcriptase |
| CHEMBL438146 | CCOc1cccc(F)c1CCNC(=S)Nc2nccs2 | Reverse transcriptase |
| CHEMBL252136 | Fc1cccc(F)c1CCNC(=S)Nc2nccs2 | Reverse transcriptase |
| CHEMBL142837 | CCN1c2ncccc2C(=O)Nc3c(C)cc(Cl)nc13 | Reverse transcriptase |
| CHEMBL250438 | COc1cccc(F)c1CCNC(=S)Nc2nccs2 | Reverse transcriptase |
| CHEMBL102113 | CCN1c2ncc(CCc3ccnc(N)c3)cc2C(=O)N(C)c4ccc(Cl)nc14 | Reverse transcriptase |
| CHEMBL144744 | CCN1c2nc(cc(C)c2NC(=O)c3cccnc13)N(C)CCO | Reverse transcriptase |
| CHEMBL55187 | COc1cc(C)cc(c1)S(=O)(=O)c2cccc(N)c2C#N | Reverse transcriptase |
| CHEMBL308907 | CCN1c2ncccc2C=Cc3cccnc13 | Reverse transcriptase |
| CHEMBL381888 | CC1CN(C2C[C@H](CO)C(C2)N=[N+]=[N-])C(=O)NC1=O | Reverse transcriptase |
| CHEMBL205764 | [O-][N+](=O)c1ccccc1NC(=O)CSc2nnnn2c3cc(F)ccc3Cl | Reverse transcriptase |
| CHEMBL295590 | CCN(CC)CCOc1ccc(NC(=O)COc2ccccc2C(=O)c3ccc(F)cc3)cc1 | Reverse transcriptase |
| CHEMBL81227 | Clc1ccc2NC(=O)NC(C#Cc3ccccc3C#N)(C4CC4)c2c1 | Reverse transcriptase |
| CHEMBL79526 | Clc1ccc2NC(=O)NC(C#Cc3nccs3)(C4CC4)c2c1 | Reverse transcriptase |
| CHEMBL171919 | CCc1cc(SCc2oc3c(Cl)ccc(Cl)c3n2)c(O)nc1C | Reverse transcriptase |
| CHEMBL162272 | CCN1c2ncc(CSc3ccncc3C)cc2C(=O)Nc4c(C)cc(Cl)nc14 | Reverse transcriptase |
| CHEMBL106783 | Oc1ncccc1C(OCc2ccccc2)(C#Cc3ccccc3)C(F)(F)F | Reverse transcriptase |
| CHEMBL320282 | Oc1ncccc1C(OCc2ccccc2)(C#CC3CC3)C(F)(F)F | Reverse transcriptase |
| CHEMBL441039 | Clc1ccc(OCC(=O)Cc2ccccc2)c(c1)C(=O)c3ccccc3 | Reverse transcriptase |
| CHEMBL66155 | CC1N(CC=C(C)C)CCN2C(=S)Nc3ccc(Cl)c1c23 | Reverse transcriptase |
| CHEMBL79338 | CCN1c2ncccc2C(=O)N(C)c3ccc(nc13)c4c[nH]c5ccccc45 | Reverse transcriptase |
| CHEMBL398891 | CCOc1ccc(F)c(CCNC(=S)Nc2ccc(Br)cn2)c1Cl | Reverse transcriptase |
| CHEMBL207105 | [O-][N+](=O)c1ccccc1NC(=O)CSc2nnnn2c3cccc(Cl)c3Cl | Reverse transcriptase |
| CHEMBL287872 | CC1=CN(C2CC(N=[N+]=[N-])C(COP(=O)(O)C(F)(F)F)O2)C(=O)NC1=O | Reverse transcriptase |
| CHEMBL309896 | Clc1ccc2NC(=O)N[C@](C#Cc3ccccn3)(C4CC4)c2c1 | Reverse transcriptase |
| CHEMBL350538 | Cc1cc(SCc2cnc3N(C4CC4)c5nc(Cl)cc(C)c5NC(=O)c3c2)cc(C)n1 | Reverse transcriptase |
| CHEMBL82764 | [O-][N+](=O)c1ccccc1C#CC2(NC(=O)Nc3ccc(Cl)cc23)C4CC4 | Reverse transcriptase |
| CHEMBL368116 | CCc1cc(CCc2oc3cccc(F)c3n2)c(S)nc1C | Reverse transcriptase |
| CHEMBL377820 | CN(C)CCCOc1ccc(NC(=O)CSc2nnnn2c3c(C)cc(C)cc3C)c(Cl)c1 | Reverse transcriptase |
| CHEMBL326408 | COC(=O)N[C@@H](C(C)C)C(=O)N[C@@H](Cc1ccccc1)[C@@H](O)CN(Cc2ccc(cc2)c3cncs3)NC(=O)[C@@H](NC(=O)OC)C(C)(C)C | Reverse transcriptase |
| CHEMBL177271 | CCc1cc(CCc2oc3c(F)ccc(F)c3n2)c(O)nc1C | Reverse transcriptase |
| CHEMBL192231 | CCN1c2ncccc2N(C)C(=O)c3cc(CCOc4ccc5cc(ccc5c4)C(=O)O)cnc13 | Reverse transcriptase |
| CHEMBL326513 | COC(=O)N[C@@H](C(C)C)C(=O)NN(C[C@H](O)[C@H](Cc1ccccc1)NC(=O)[C@@H](NC(=O)OC)C(C)(C)C)Cc2ccc(cc2)c3cncs3 | Reverse transcriptase |
| CHEMBL189942 | CCN1c2nc(F)ccc2N(C)C(=O)c3cc(CCOc4ccc(cc4C)C(=O)O)cnc13 | Reverse transcriptase |
| CHEMBL39999 | Brc1ccc(NC(=S)NCCc2ccccn2)nc1 | Reverse transcriptase |
| CHEMBL317356 | Brc1ccc(NC(=S)NCCc2ccccc2)nc1 | Reverse transcriptase |
| CHEMBL191163 | CCN1c2ncc(CCOc3ccc(cc3C)C(=O)O)cc2C(=O)N(C)c4ccc(OC)nc14 | Reverse transcriptase |
| CHEMBL176362 | CCc1cc(CCc2oc3cccc(F)c3n2)c(O)nc1C | Reverse transcriptase |
| CHEMBL336237 | CCc1c(C)nc(O)c(N)c1Sc2cc(C)cc(C)c2 | Reverse transcriptase |
| CHEMBL175162 | CCc1cc(CCc2oc3c(F)ccc(F)c3n2)c(S)nc1C | Reverse transcriptase |
| CHEMBL315289 | Cc1cc(C)cc(c1)[C@]23SCCN2C(=O)c4ccccc34 | Reverse transcriptase |
| CHEMBL133772 | CCOC(=O)NCCC=C(c1cc(C)c(OC)c(c1)C(=O)OC)c2cc(C)c(OC)c(c2)C(=O)OC | Reverse transcriptase |
| CHEMBL326347 | COC(=O)N[C@@H](C(C)C)C(=O)N[C@@H](Cc1ccccc1)[C@@H](O)CN(CCC(C)C)NC(=O)[C@@H](NC(=O)OC)C(C)C | Reverse transcriptase |
| CHEMBL336426 | CCOc1ccc(F)c(C2CC2NC(=O)Nc3cc(Cl)cnn3)c1Cl | Reverse transcriptase |
| CHEMBL339067 | Nc1cc(Cl)nc(SCc2cc3OCOc3cc2Cl)n1 | Reverse transcriptase |
| CHEMBL320379 | Oc1ncccc1C(OCc2ccccc2)(\C=C\C3CC3)C(F)(F)F | Reverse transcriptase |
| CHEMBL280541 | C[C@H](Sc1nc(N)cc(Cl)n1)c2cc3ccoc3cn2 | Reverse transcriptase |
| CHEMBL192530 | CCN1c2ncccc2N(C)C(=O)c3cc(CCOc4ccc(cc4C)C(=O)O)cnc13 | Reverse transcriptase |
| CHEMBL80064 | Clc1ccc2NC(=O)NC(C#Cc3ccccc3)(C4CC4)c2c1 | Reverse transcriptase |
| CHEMBL192790 | CCN1c2ncccc2N(C)C(=O)c3cc(CCOc4ccc(cc4Br)C(=O)O)cnc13 | Reverse transcriptase |
| CHEMBL57995 | Oc1ncc2c(F)c(F)ccc2n1 | Reverse transcriptase |
| CHEMBL3143930 | O[C@H]1C[C@@H](O[C@@H]1COP(=O)(O)C(F)(F)F)N2C=C(F)C(=O)NC2=O | Reverse transcriptase |
| CHEMBL342771 | CCN1c2nc(ccc2N(C)C(=O)c3cccnc13)c4ccccn4 | Reverse transcriptase |
| CHEMBL382454 | Cc1cccc(c1C)n2nnnc2SCC(=O)Nc3ccccc3[N+](=O)[O-] | Reverse transcriptase |
| CHEMBL325900 | COC(=O)N[C@@H](C(C)C)C(=O)N[C@@H](Cc1ccccc1)[C@@H](O)CN(Cc2ccc(cc2)c3nccs3)NC(=O)[C@@H](NC(=O)OC)C(C)(C)C | Reverse transcriptase |
| CHEMBL189928 | CCN1c2ncccc2N(C)C(=O)c3cc(CCOc4ccc(cc4CC)C(=O)O)cnc13 | Reverse transcriptase |
| CHEMBL55445 | CCN1C(=O)c2cccnc2Oc3cc(C)cc(C#N)c13 | Reverse transcriptase |
| CHEMBL86109 | CCc1cc(CNc2cc(CC)c(C)nc2O)c(OC)nc1C | Reverse transcriptase |
| CHEMBL85947 | CC(C)Sc1nc(O)c(C)c(n1)C(C)c2c(F)cccc2F | Reverse transcriptase |
| CHEMBL189423 | CCN1c2nc(C)ccc2N(C)C(=O)c3cc(CCOc4ccc(cc4C)C(=O)O)cnc13 | Reverse transcriptase |
| CHEMBL3038082 | CC[C@@H]1NC(=O)[C@H]([C@H](O)[C@H](C)C\C=C\C)N(C)C(=O)[C@H](C(C)C)N(C)C(=O)[C@H](CC(C)C)N(C)C(=O)[C@H](CC(C)C)N(C)C(=O)[C@@H](C)NC(=O)[C@H](C)NC(=O)[C@H](CC(C)C)N(C)C(=O)[C@@H](NC(=O)[C@H](CC(C)(C)O)N(C)C(=O)[C@@H](SCCN2CCCCC2)N(C)C1=O)C(C)C | Reverse transcriptase |
| CHEMBL3038084 | CC[C@@H]1NC(=O)[C@H]([C@H](O)[C@H](C)C\C=C\C)N(C)C(=O)[C@H](C(C)C)N(C)C(=O)[C@H](CC(C)C)N(C)C(=O)[C@H](CC(C)C)N(C)C(=O)[C@@H](C)NC(=O)[C@H](C)NC(=O)[C@H](CC(C)C)N(C)C(=O)[C@@H](NC(=O)[C@H](CC(C)C)N(C)C(=O)[C@@H](SCCN(C)C(C)C)N(C)C1=O)C(C)C | Reverse transcriptase |
| CHEMBL314012 | CN1C(=O)Nc2ccc(Cl)cc2C1(C#Cc3ccccn3)C4CC4 | Reverse transcriptase |
| CHEMBL433489 | CN1C(=O)c2cc(N)ccc2Oc3cc(C)cc(C)c13 | Reverse transcriptase |
| CHEMBL99896 | CN(C)c1cc(F)c(CC\N=C(/S)\Nc2ccc(Cl)cn2)c(F)c1 | Reverse transcriptase |
| CHEMBL37803 | CCc1cc(NCc2oc3c(C)ccc(C)c3n2)c(O)nc1C | Reverse transcriptase |
| CHEMBL288336 | CCN1c2ncccc2C(=O)N(C)c3ccc(Cl)nc13 | Reverse transcriptase |
| CHEMBL83622 | CCc1cc(CCc2oc3ccccc3n2)c(O)nc1C | Reverse transcriptase |
| CHEMBL145237 | CCN1c2ncccc2C(=O)N(C)c3ccc(nc13)c4cn[nH]c4 | Reverse transcriptase |
| CHEMBL333386 | COC(=O)N[C@@H](C(C)C)C(=O)N[C@@H](Cc1ccccc1)[C@@H](O)CN(Cc2ccc(cc2)c3ccccn3)NC(=O)[C@@H](NC(=O)OC)C(C)(C)C | Reverse transcriptase |
| CHEMBL3143101 | NC(=O)c1cn([C@@H]2O[C@H](CO)[C@@H](O)[C@@H]2F)c3ncnc(N)c13 | Reverse transcriptase |
| CHEMBL144326 | CCN1c2nc(ccc2N(C)C(=O)c3cccnc13)N4CCCC4 | Reverse transcriptase |
| CHEMBL343007 | Cc1cc(Cl)nc2N(C3CC3)c4ncccc4C(=O)Nc12 | Reverse transcriptase |
| CHEMBL85946 | CCC(C)Sc1nc(O)c(C)c(n1)C(C)c2c(F)cccc2F | Reverse transcriptase |
| CHEMBL368084 | O\C(=C/C(=O)c1nc[nH]n1)\c2oc(Cc3ccc(F)cc3)cc2 | Reverse transcriptase |
| CHEMBL212448 | COC(=O)CCC\C=C(/c1cc(C)c2OC(=O)N(C)c2c1)\c3cc(C)c(OC)c(c3)C(=O)OC | Reverse transcriptase |
| CHEMBL101845 | CCN1c2ncc(CCc3ccc4ccccc4c3)cc2C(=O)N(C)c5ccc(F)nc15 | Reverse transcriptase |
| CHEMBL356744 | CCN1c2nc(SC)cc(C)c2NC(=O)c3cccnc13 | Reverse transcriptase |
| CHEMBL145199 | CCN1c2ncccc2C(=O)Nc3c(C)cc(F)nc13 | Reverse transcriptase |
| CHEMBL142659 | CCN1c2nc(ccc2N(C)C(=O)c3cccnc13)c4ccc(N)nc4 | Reverse transcriptase |
| CHEMBL316796 | S=C(NCCc1ccccc1)Nc2ccccn2 | Reverse transcriptase |
| CHEMBL342103 | CCN1c2ncccc2C(=O)Nc3c(C)cc(C)nc13 | Reverse transcriptase |
| CHEMBL310803 | CSc1nc(O)c(C)c(n1)C(C)c2c(F)cccc2F | Reverse transcriptase |
| CHEMBL100833 | CCN1c2nc(F)ccc2N(C)C(=O)c3cc(CCc4ccncc4)cnc13 | Reverse transcriptase |
| CHEMBL338047 | CCC1=C(C)NC(=O)C(=C1Cc2cc(C)cc(C)c2)N(C)C | Reverse transcriptase |
| CHEMBL331237 | CN1N=C(N)c2cn([C@@H]3O[C@H](CO)[C@@H](O)[C@H]3O)c4ncnc1c24 | Reverse transcriptase |
| CHEMBL135509 | Brc1ccc(NC(=S)NCCC2=NC3C=CC=CC3N2)nc1 | Reverse transcriptase |
| CHEMBL287346 | CC1=CN(C2CC(N=[N+]=[N-])C(COP(=O)(O)C(F)F)O2)C(=O)NC1=O | Reverse transcriptase |
| CHEMBL398837 | CCOc1c(Cl)cc(F)c(CCNC(=S)Nc2ccc(Br)cn2)c1Cl | Reverse transcriptase |
| CHEMBL298607 | COC(=O)c1cc(C)cc2Oc3ncccc3C(=O)N(C)c12 | Reverse transcriptase |
| CHEMBL326941 | CCOC(=O)N[C@@H](C(C)C)C(=O)NN(C[C@H](O)[C@H](Cc1ccccc1)NC(=O)[C@@H](NC(=O)OC)C(C)(C)C)Cc2ccc(cc2)c3cncs3 | Reverse transcriptase |
| CHEMBL280752 | CC(Sc1nc(N)cc(Cl)n1)c2cc3ccoc3cn2 | Reverse transcriptase |
| CHEMBL364043 | CCN1c2ncccc2N(C)C(=O)c3cc(CCOc4ccc(CCC(=O)O)cc4C)cnc13 | Reverse transcriptase |
| CHEMBL148372 | Cc1nc([nH]c1C)c2[nH]c3ccc(Cl)cc3c2S(=O)(=O)c4ccccc4 | Reverse transcriptase |
| CHEMBL204993 | Cc1cc(C)c(c(C)c1)n2nnnc2SCC(=O)Nc3ccc(cc3Cl)[N+](=O)[O-] | Reverse transcriptase |
| CHEMBL2368773 | COC[C@H]1O[C@H]([C@H](O)[C@@H]1OP(=O)(C)O)n2cnc3C(=O)N(CC#C)C=Nc23 | Reverse transcriptase |
| CHEMBL370699 | CCN1c2ncccc2N(C)C(=O)c3cc(CCOc4ccc(CC(=O)O)cc4C)cnc13 | Reverse transcriptase |
| CHEMBL56051 | CN1C(=O)c2cccnc2Oc3cc(C)cc(c13)[N+](=O)[O-] | Reverse transcriptase |
| CHEMBL339203 | Cc1nc(O)c(NCc2oc3c(Cl)ccc(Cl)c3n2)cc1C=C | Reverse transcriptase |
| CHEMBL29274 | FC(F)(F)[C@]1(NC(=O)Nc2ccc(Cl)cc12)\C=C\C3CC3 | Reverse transcriptase |
| CHEMBL304294 | CC1N(CC=C(C)C)CCN2C(=S)Nc3cc(Cl)cc1c23 | Reverse transcriptase |
| CHEMBL148371 | Clc1ccc2[nH]c(c3ncc[nH]3)c(c2c1)S(=O)(=O)c4ccccc4 | Reverse transcriptase |
| CHEMBL207527 | Cc1ccc(Cl)cc1n2nnnc2SCC(=O)Nc3ccccc3[N+](=O)[O-] | Reverse transcriptase |
| CHEMBL87765 | CCc1cc(NCc2cc(C)ccc2OC)c(O)nc1C | Reverse transcriptase |
| CHEMBL317574 | CNC(=O)c1ccc(F)c(CC\N=C(/S)\Nc2ccc(Br)cn2)c1F | Reverse transcriptase |
| CHEMBL329720 | CCc1cc(CCc2oc3c(C)ccc(C)c3n2)c(O)nc1C | Reverse transcriptase |
| CHEMBL311996 | Clc1ccc2NC(=O)NC(C#Cc3ccccn3)(C4CC4)c2c1 | Reverse transcriptase |
| CHEMBL172861 | CCc1cc(CCc2oc3ccccc3n2)c(S)nc1C | Reverse transcriptase |
| CHEMBL3038085 | CC[C@@H]1NC(=O)[C@H]([C@H](O)[C@H](C)C\C=C\C)N(C)C(=O)[C@H](C(C)C)N(C)C(=O)[C@H](CC(C)C)N(C)C(=O)[C@H](CC(C)C)N(C)C(=O)[C@@H](C)NC(=O)[C@H](C)NC(=O)[C@H](CC(C)C)N(C)C(=O)[C@@H](NC(=O)[C@H](CC(C)C)N(C)C(=O)[C@@H](SCCN(C)C(C)(C)C)N(C)C1=O)C(C)C | Reverse transcriptase |
| CHEMBL106694 | Oc1ncccc1C(Oc2cccc(Oc3ccccc3)c2)(C#CC4CC4)C(F)(F)F | Reverse transcriptase |
| CHEMBL39665 | CN(C)c1ccc(F)c(CCNC(=O)Nc2ccc(Br)cn2)c1F | Reverse transcriptase |
| CHEMBL91473 | Fc1ccc(F)c(c1)C(=O)CC\N=C(/S)\Nc2ccc(Br)cn2 | Reverse transcriptase |
| CHEMBL283950 | NC(=O)c1[nH]c2ccc(Cl)cc2c1S(=O)(=O)c3ccccc3 | Reverse transcriptase |
| CHEMBL26752 | C[C@@H](Sc1nc(N)cc(Cl)n1)c2cc3ccoc3cn2 | Reverse transcriptase |
| CHEMBL106997 | Oc1ncccc1C(OCc2cccnc2)(C#CC3CC3)C(F)(F)F | Reverse transcriptase |
| CHEMBL104349 | Oc1ncccc1C(OCc2ccccn2)(C#CC3CC3)C(F)(F)F | Reverse transcriptase |
| CHEMBL1163 | COC(=O)N[C@H](C(=O)N[C@@H](Cc1ccccc1)[C@@H](O)CN(Cc2ccc(cc2)c3ccccn3)NC(=O)[C@@H](NC(=O)OC)C(C)(C)C)C(C)(C)C | Reverse transcriptase |
| CHEMBL162629 | CCN1c2nc(Cl)cc(C)c2NC(=O)c3cc(CSc4cncnc4)cnc13 | Reverse transcriptase |
| CHEMBL66350 | CC1Cn2c(S)nc3cccc(CN1CC(=C)C)c23 | Reverse transcriptase |
| CHEMBL362936 | CCN1c2ncccc2N(C)C(=O)c3cc(CCOc4ccc(C(=O)O)c5ccccc45)cnc13 | Reverse transcriptase |
| CHEMBL294171 | CCN1C(=O)c2cc(N)cnc2Oc3cc(C)cc(C)c13 | Reverse transcriptase |
| CHEMBL327777 | Brc1ccc(NC(=S)NCCC(=O)c2ccccc2)nc1 | Reverse transcriptase |
| CHEMBL162905 | CCN1c2ncc(CSc3ccnc(C)c3)cc2C(=O)Nc4c(C)cc(Cl)nc14 | Reverse transcriptase |
| CHEMBL115673 | COC(=O)N[C@H](C(=O)N[C@@H](Cc1ccccc1)[C@@H](O)CN(Cc2ccc(cc2)c3nnn(C)n3)NC(=O)[C@@H](NC(=O)OC)C(C)(C)C)C(C)(C)C | Reverse transcriptase |
| CHEMBL116229 | CCC(C)[C@H](NC(=O)OC)C(=O)N[C@@H](Cc1ccccc1)[C@@H](O)CN(Cc2ccc(cc2)c3ccccn3)NC(=O)[C@@H](NC(=O)OC)C(C)C | Reverse transcriptase |
| CHEMBL174130 | CCc1cc(CCc2oc3c(Cl)cccc3n2)c(S)nc1C | Reverse transcriptase |
| CHEMBL324521 | COC(=O)N[C@@H](C(C)C)C(=O)N[C@@H](Cc1ccccc1)[C@@H](O)CN(Cc2ccc(cc2)c3ccccn3)NC(=O)[C@@H](NC(=O)OC)C(C)C | Reverse transcriptase |
| CHEMBL2373969 | OC(=C)C[C@H]1[C@@H](C(=O)O)[C@]23[C@H]4[C@@H]5c6c7c8[C@H]9[C@@H]%10[C@@H]%11[C@H]%12C%13=C%14C%15=C%11c%16c%10c%17c8c%18c6c%19c5c%20c%21c%22c(c%23C%24=C2c%25c([C@@H]%13[C@H]%24c%26c%14c%27c%15c%28c%16c%29c%17c%18c%30c%19c%21c%31c%30c%29c%28c%32c%27c(c%23%26)c%22c%31%32)c%12c9c7c4%25)[C@]3%20[C@@H]1C(=O)O | Reverse transcriptase |
| CHEMBL440860 | CCN1C(=O)c2cccnc2Oc3cc(C)cc(C)c13 | Reverse transcriptase |
| CHEMBL53043 | Nc1cccc(c1C#N)S(=O)(=O)c2ccc3ccccc3c2 | Reverse transcriptase |
| CHEMBL301222 | CN1C(=O)c2cc(N)ccc2Oc3cc(C)ccc13 | Reverse transcriptase |
| CHEMBL108396 | COc1ccc(cc1)C(=O)CSC2=NC(=C(C(C)C)C(=O)N2)Cc3cccc4ccccc34 | Reverse transcriptase |
| CHEMBL83825 | CCc1cc(NCN2C(=O)c3ccccc3C2=O)c(O)nc1C | Reverse transcriptase |
| CHEMBL416107 | CCc1c(C)nc(O)c(c1Sc2cc(C)cc(C)c2)[N+](=O)[O-] | Reverse transcriptase |
| CHEMBL252135 | COc1ccc(Br)cc1CCNC(=S)Nc2nccs2 | Reverse transcriptase |
| CHEMBL398765 | Cc1ccc(NC(=S)NCCc2ccccc2)nc1 | Reverse transcriptase |
| CHEMBL172311 | CCc1cc(CCc2oc3c(Cl)ccc(Cl)c3n2)c(S)nc1C | Reverse transcriptase |
| CHEMBL321544 | CCN1c2nc(Cl)ccc2N(C)C(=O)c3cc(CCc4ccncc4)cnc13 | Reverse transcriptase |
| CHEMBL318002 | CCN1c2ncc(COc3cc[n+]([O-])cc3)cc2C(=O)N(C)c4ccc(F)nc14 | Reverse transcriptase |
| CHEMBL319091 | CCN1c2ncccc2N(C)C(=O)c3cc(CCc4ccncc4)cnc13 | Reverse transcriptase |
| CHEMBL366402 | CC1=C(Cc2c(F)cccc2F)N=C(NC3CCCC3)NC1=O | Reverse transcriptase |
| CHEMBL85446 | Cc1cccc(c1)C23SCCN2C(=O)c4ccccc34 | Reverse transcriptase |
| CHEMBL55951 | Nc1cccc(c1C#N)S(=O)(=O)c2cc(Cl)cc(Cl)c2 | Reverse transcriptase |
| CHEMBL116350 | Brc1ccc(NC(=S)NCCc2c[nH]c3ccccc23)nc1 | Reverse transcriptase |
| CHEMBL147749 | S\C(=N/CCc1ccccc1N=[N+]=[N-])\Nc2nccs2 | Reverse transcriptase |
| CHEMBL144522 | CN1C(=O)c2cccnc2N(C3CC3)c4nc(Br)ccc14 | Reverse transcriptase |
| CHEMBL100981 | CCN1c2ncc(CSc3ncccn3)cc2C(=O)N(C)c4ccc(Cl)nc14 | Reverse transcriptase |
| CHEMBL100445 | CCN1c2ncc(COc3ccnc(C)c3)cc2C(=O)N(C)c4ccc(Cl)nc14 | Reverse transcriptase |
| CHEMBL103953 | CCN1c2ncccc2N(C)C(=O)c3cc(CCc4ccnc(C)c4)cnc13 | Reverse transcriptase |
| CHEMBL174419 | CCc1cc(CCc2oc3cccc(Cl)c3n2)c(S)nc1C | Reverse transcriptase |
| CHEMBL81319 | CCN1c2ncccc2C(=O)N(C)c3ccc(nc13)c4cc[nH]c4 | Reverse transcriptase |
| CHEMBL144852 | CCN1c2ncccc2C(=O)N(C)c3ccc(nc13)N4CC=CC4 | Reverse transcriptase |
| CHEMBL309674 | CC(C1=CC(=O)NC(=N1)SC2CCCC2)c3c(F)cccc3F | Reverse transcriptase |
| CHEMBL283619 | FC(F)(F)[C@]1(NC(=O)Nc2ccc(Cl)cc12)C#CC3CC3 | Reverse transcriptase |
| CHEMBL114227 | COC(=O)N[C@@H](C(C)C)C(=O)NN(C[C@H](O)[C@H](Cc1ccccc1)NC(=O)[C@@H](NC(=O)OC)C(C)(C)C)Cc2ccc(cc2)c3ccccn3 | Reverse transcriptase |
| CHEMBL291763 | CCN1C(=O)c2cccnc2Oc3cc(C)cc(c13)[N+](=O)[O-] | Reverse transcriptase |
| CHEMBL85059 | Cc1cc(C)cc(c1)S(=O)(=O)c2c([nH]c3ccc(Cl)cc23)C(=O)N | Reverse transcriptase |
| CHEMBL205999 | Cc1cc(C)c(c(C)c1)n2nnnc2SCC(=O)Nc3ccccc3I | Reverse transcriptase |
| CHEMBL82798 | CCc1ccc(OC)c(CNc2cc(CC)c(C)nc2O)c1 | Reverse transcriptase |
| CHEMBL87676 | CCc1cc(NCc2ccc(C)cc2OC)c(O)nc1C | Reverse transcriptase |
| CHEMBL36995 | CCOCN1C(=O)NC(=O)C(=C1Sc2ccccc2)CC | Reverse transcriptase |
| CHEMBL442013 | COC(=O)N[C@@H](C(C)C)C(=O)N[C@@H](Cc1ccccc1)[C@@H](O)CN(Cc2ccc(cc2)c3cncs3)NC(=O)[C@@H](NC(=O)OC)C(C)C | Reverse transcriptase |
| CHEMBL114039 | COC(=O)N[C@H](C(=O)N[C@@H](Cc1ccccc1)[C@@H](O)CN(Cc2ccc(cc2)c3nccs3)NC(=O)[C@@H](NC(=O)OC)C(C)(C)C)C(C)(C)C | Reverse transcriptase |
| CHEMBL83635 | CCc1cc(CNc2cc(CC)c(C)nc2O)c(OC)cc1C | Reverse transcriptase |
| CHEMBL177016 | CCc1cc(CCc2oc3cccc(C)c3n2)c(O)nc1C | Reverse transcriptase |
| CHEMBL113943 | CCOC(=O)N[C@@H](C(C)C)C(=O)NN(C[C@H](O)[C@H](Cc1ccccc1)NC(=O)[C@@H](NC(=O)OC)C(C)C)Cc2ccc(cc2)c3ccccn3 | Reverse transcriptase |
| CHEMBL84933 | CCc1cc(NCc2cc3ccccc3nc2OC)c(O)nc1C | Reverse transcriptase |
| CHEMBL69566 | CC1Cn2c(S)nc3cc(Cl)cc(CN1CC4CC4)c23 | Reverse transcriptase |
| CHEMBL311075 | CC1=CN(C2CC(N=[N+]=[N-])C(COP(=O)(O)P(=O)(O)P(=O)(O)O)O2)C(=O)NC1=O | Reverse transcriptase |
| CHEMBL176957 | CCCc1cc(CCc2oc3c(Cl)ccc(Cl)c3n2)c(O)nc1C | Reverse transcriptase |
| CHEMBL332042 | COC(=O)N[C@@H](C(C)C)C(=O)N[C@@H](Cc1ccccc1)[C@@H](O)CN(Cc2ccc(cc2)c3ccccc3)NC(=O)[C@@H](NC(=O)OC)C(C)C | Reverse transcriptase |
| CHEMBL3038081 | CC[C@@H]1NC(=O)[C@H]([C@H](O)[C@H](C)C\C=C\C)N(C)C(=O)[C@H](C(C)C)N(C)C(=O)[C@H](CC(C)C)N(C)C(=O)[C@H](CC(C)C)N(C)C(=O)[C@@H](C)NC(=O)[C@H](C)NC(=O)[C@H](CC(C)C)N(C)C(=O)[C@@H](NC(=O)[C@H](CC(C)C)N(C)C(=O)[C@@H](SCCN(C)C)N(C)C1=O)C(C)C | Reverse transcriptase |
| CHEMBL37628 | CCN1c2nccc(C)c2NC(=O)c3cccnc13 | Reverse transcriptase |
| CHEMBL430639 | Cc1cccc(c1)[C@]23SCCN2C(=O)c4ccccc34 | Reverse transcriptase |
| CHEMBL368172 | CC(C1=C(C)C(=O)NC(=N1)NC2CCCC2)c3c(F)cccc3F | Reverse transcriptase |
| CHEMBL72472 | Nc1ncnc2c1ncn2C3COC(COP(=O)(O)OP(=O)(O)OP(=O)(O)O)C3 | Reverse transcriptase |
| CHEMBL608901 | CCCCCCC(=O)NC1=NN(C)c2ncnc3c2c1cn3C4O[C@H](CO)[C@@H](O)[C@H]4O | Reverse transcriptase |
| CHEMBL70629 | CCN1c2ccc(C)c(C)c2N(C)C(=O)c3cccnc13 | Reverse transcriptase |
| CHEMBL105863 | Oc1nc2ccccc2cc1C(OCc3ccccc3)(C#CC4CC4)C(F)(F)F | Reverse transcriptase |
| CHEMBL367052 | CCc1cc(CCc2oc3c(F)cccc3n2)c(O)nc1C | Reverse transcriptase |
| CHEMBL337029 | C[C@H]1Oc2c3C=CC(C)(C)Oc3c4C(=CC(=O)Oc4c2[C@@H](O)[C@@H]1C)c5ccccc5 | Reverse transcriptase |
| CHEMBL307220 | CCN1c2cccc(C)c2NC(=O)c3cccnc13 | Reverse transcriptase |
| CHEMBL64233 | CC(C)C#C[C@@]1(NC(=O)Nc2ccc(F)cc12)C(F)(F)F | Reverse transcriptase |
| CHEMBL171920 | CCc1cc(CCc2oc3c(Cl)cccc3n2)c(O)nc1C | Reverse transcriptase |
| CHEMBL2368774 | COC[C@H]1O[C@H]([C@H](OC)[C@@H]1OP(=S)(C)O)n2cnc3C(=O)N(CC=C)C=Nc23 | Reverse transcriptase |
| CHEMBL55186 | COc1cc(cc(c1)S(=O)(=O)c2cccc(N)c2C#N)C(F)(F)F | Reverse transcriptase |
| CHEMBL102034 | CCN1c2nc(F)ccc2N(C)C(=O)c3cc(COc4ccncc4)cnc13 | Reverse transcriptase |
| CHEMBL254201 | COc1ccccc1CCNC(=S)Nc2nccs2 | Reverse transcriptase |
| CHEMBL144145 | CCN1c2nc(ccc2N(C)C(=O)c3cccnc13)c4ccc(N)cc4 | Reverse transcriptase |
| CHEMBL177201 | CC(C1=CC(=O)NC(=N1)NC2CCCC2)c3c(F)cccc3F | Reverse transcriptase |
| CHEMBL99747 | CCN1c2ncc(COc3ccnc(c3)C(F)(F)F)cc2C(=O)N(C)c4ccc(Cl)nc14 | Reverse transcriptase |
| CHEMBL328848 | CCN1c2ncccc2N(C)C(=O)c3cc(CCc4nccs4)cnc13 | Reverse transcriptase |
| CHEMBL439943 | CCN1c2cc(C)c(C)cc2N(C)C(=O)c3cccnc13 | Reverse transcriptase |
| CHEMBL144851 | CCN1c2nc(cc(C)c2NC(=O)c3cccnc13)N4CCC(O)C4 | Reverse transcriptase |
| CHEMBL103946 | CCN1c2nc(Cl)ccc2N(C)C(=O)c3cc(COc4cccc(c4)C(=O)O)cnc13 | Reverse transcriptase |
| CHEMBL101946 | CCN1c2ncccc2N(C)C(=O)c3cc(CCc4cccc(N)c4)cnc13 | Reverse transcriptase |
| CHEMBL176443 | CCc1cc(CCc2oc3c(C)cccc3n2)c(O)nc1C | Reverse transcriptase |
| CHEMBL314883 | CCc1cc(NCc2cc3CCCc3cc2OC)c(O)nc1C | Reverse transcriptase |
| CHEMBL344289 | CCN1c2ncccc2C(=O)N(C)c3ccc(nc13)c4cocc4 | Reverse transcriptase |
| CHEMBL35382 | CC1=CN(C2CC(N=[N+]=[N-])C(COP(=O)(O)CF)O2)C(=O)NC1=O | Reverse transcriptase |
| CHEMBL145397 | CCN1c2nc(OC)ccc2N(C)C(=O)c3cccnc13 | Reverse transcriptase |
| CHEMBL462018 | CN1N=C(N)c2cn([C@@H]3O[C@H](COP(=O)(O)O)[C@@H](O)[C@H]3O)c4ncnc1c24 | Reverse transcriptase |
| CHEMBL313450 | CCc1cc(NCc2nc3CCCc3cc2OC)c(O)nc1C | Reverse transcriptase |
| CHEMBL324601 | CCN(CC)c1ccc(CN(C[C@H](O)[C@H](Cc2ccccc2)NC(=O)[C@@H](NC(=O)OC)C(C)(C)C)NC(=O)[C@@H](NC(=O)OC)C(C)(C)C)cc1 | Reverse transcriptase |
| CHEMBL116165 | COC(=O)N[C@H](C(=O)N[C@@H](Cc1ccccc1)[C@@H](O)CN(Cc2ccc(cc2)c3cncs3)NC(=O)[C@@H](NC(=O)OC)C(C)(C)C)C(C)(C)C | Reverse transcriptase |
| CHEMBL325343 | CCc1cc(c(O)nc1C)C(OCc2c(Cl)cccc2Cl)(C#CC3CC3)C(F)(F)F | Reverse transcriptase |
| CHEMBL125336 | CCc1cc(NCc2oc3c(Cl)ccc(Cl)c3n2)c(S)nc1C | Reverse transcriptase |
| CHEMBL303015 | C[C@@H]1Cn2c(S)nc3cccc([C@@H](C)N1CC=C(C)C)c23 | Reverse transcriptase |
| CHEMBL291987 | CCN1C(=O)c2cc(N)ccc2Oc3cc(C)cc(C)c13 | Reverse transcriptase |
| CHEMBL338263 | CSc1cc(NCc2oc3c(Cl)ccc(Cl)c3n2)c(O)nc1C | Reverse transcriptase |
| CHEMBL296092 | CC1Cn2c(S)nc3cc(Cl)cc(CN1CC=C(C)C)c23 | Reverse transcriptase |
| CHEMBL172583 | CCc1cc(CCc2oc3c(F)cccc3n2)c(S)nc1C | Reverse transcriptase |
| CHEMBL3038083 | CC[C@@H]1NC(=O)[C@H]([C@H](O)[C@H](C)C\C=C\C)N(C)C(=O)[C@H](C(C)C)N(C)C(=O)[C@H](CC(C)C)N(C)C(=O)[C@H](CC(C)C)N(C)C(=O)[C@@H](C)NC(=O)[C@H](C)NC(=O)[C@H](CC(C)C)N(C)C(=O)[C@@H](NC(=O)[C@H](CC(C)(C)O)N(C)C(=O)[C@@H](SCCN(C)C(C)(C)C)N(C)C1=O)C(C)C | Reverse transcriptase |
| CHEMBL322004 | Oc1ncccc1C(OCc2ccccc2)(\C=C\c3ccccc3)C(F)(F)F | Reverse transcriptase |
| CHEMBL56501 | CC(C)N1C(=O)c2cc(N)cnc2Oc3cc(C)c(C)cc13 | Reverse transcriptase |
| CHEMBL400708 | CC(=O)NCc1ccc(F)c(CCNC(=S)Nc2ccc(Br)cn2)c1F | Reverse transcriptase |
| CHEMBL440706 | CCc1c(O)nc(SCC(=O)c2ccc(OC)cc2)nc1Cc3cccc4ccccc34 | Reverse transcriptase |
| CHEMBL1269597 | CC[C@@H]1NC(=O)[C@H]([C@H](O)[C@H](C)C\C=C\C)N(C)C(=O)[C@H](C(C)C)N(C)C(=O)[C@H](CC(C)C)N(C)C(=O)[C@H](CC(C)C)N(C)C(=O)[C@@H](C)NC(=O)[C@H](C)NC(=O)[C@H](CC(C)C)N(C)C(=O)[C@@H](NC(=O)[C@H](CC(C)(C)O)N(C)C(=O)[C@@H](SCCN(C)C)N(C)C1=O)C(C)C | Reverse transcriptase |
| CHEMBL300087 | CCOC(=O)N1c2ccccc2NC(=O)C1(C#CC3CC3)C(F)(F)F | Reverse transcriptase |
| CHEMBL56500 | CN1C(=O)c2cc(N)cnc2Oc3cc(C)cc(C)c13 | Reverse transcriptase |
| CHEMBL31977 | CC1OC(\C=C\C2CC2)(c3cc(Cl)ccc3NC1=O)C(F)(F)F | Reverse transcriptase |
| CHEMBL431868 | CC(C)c1c(O)nc(SCC(=O)c2ccccc2)nc1Cc3cccc4ccccc34 | Reverse transcriptase |
| CHEMBL420367 | CC(=CCN1CCN2C(=S)Nc3ccc(Cl)c(C1)c23)C | Reverse transcriptase |
| CHEMBL135760 | CCOc1ccc(F)c(CC\N=C(/S)\Nc2nc(cs2)C(F)(F)F)c1Cl | Reverse transcriptase |
| CHEMBL3038090 | CC[C@@H]1NC(=O)[C@H]([C@H](O)[C@H](C)C\C=C\C)N(C)C(=O)[C@H](C(C)C)N(C)C(=O)[C@H](CC(C)C)N(C)C(=O)[C@H](CC(C)C)N(C)C(=O)[C@@H](C)NC(=O)[C@H](C)NC(=O)[C@H](CC(C)C)N(C)C(=O)[C@@H](NC(=O)[C@H](CC(C)(C)O)N(C)C(=O)[C@@H](SCCN(CC)CC)N(C)C1=O)C(C)C | Reverse transcriptase |
| CHEMBL89195 | Fc1ccc(cc1)C(=O)CC\N=C(/S)\Nc2ccc(Br)cn2 | Reverse transcriptase |
| CHEMBL313404 | CCc1cc(NCc2nc3CCCCc3cc2OC)c(O)nc1C | Reverse transcriptase |
| CHEMBL176594 | CC(C)c1cc(CCc2oc3ccccc3n2)c(O)nc1C | Reverse transcriptase |
| CHEMBL116474 | Brc1ccc(NC(=S)NCCc2cccs2)nc1 | Reverse transcriptase |
| CHEMBL102192 | CCN1c2nc(Cl)ccc2N(C)C(=O)c3cc(CCc4ccccc4)cnc13 | Reverse transcriptase |
| CHEMBL105318 | CC1=CN([C@H]2C[C@H](F)[C@@H](CO)O2)C(=O)NC1=O | Reverse transcriptase |
| CHEMBL341350 | Cc1nc(CCCCCCC(=O)c2ccccc2)n3nc(ccc13)N4CCCC4 | Reverse transcriptase |
| CHEMBL101091 | CCN1c2ncc(COc3cccc(N)c3)cc2C(=O)N(C)c4ccc(Cl)nc14 | Reverse transcriptase |
| CHEMBL317180 | CCN1c2ncc(CSc3[nH]cnc4nncc34)cc2C(=O)N(C)c5ccc(Cl)nc15 | Reverse transcriptase |
| CHEMBL123959 | CCN(CC)C(=O)\C=C\CSc1nc(N)cc(Cl)n1 | Reverse transcriptase |
| CHEMBL444080 | CCN1c2nc(OC)cc(CO)c2NC(=O)c3cccnc13 | Reverse transcriptase |
| CHEMBL2092833 | CC1=CN([C@@H]2C[C@@H](F)[C@H](COP(=O)(O)OP(=O)(O)OP(=O)(O)O)O2)C(=O)NC1=O | Reverse transcriptase |
| CHEMBL54900 | CN1C(=O)c2cc(ccc2Oc3cc(C)cc(C)c13)C#N | Reverse transcriptase |
| CHEMBL38679 | Cc1ncc([N+](=O)[O-])n1CCOC(c2ccccc2)c3ccccc3 | Reverse transcriptase |
| CHEMBL100352 | CCN1c2ncccc2N(C)C(=O)c3cc(CO)cnc13 | Reverse transcriptase |
| CHEMBL330416 | CCN1c2ncc(CCc3ccnc(c3)C(=O)N)cc2C(=O)N(C)c4ccc(Cl)nc14 | Reverse transcriptase |
| CHEMBL303089 | Fc1ccc2NC(=O)N[C@@](C#CC3CC3)(c2c1)C(F)(F)F | Reverse transcriptase |
| CHEMBL87393 | CCC(C)Sc1nc(O)cc(Cc2c(F)cccc2F)n1 | Reverse transcriptase |
| CHEMBL320663 | CCN1c2nc(Cl)ccc2N(C)C(=O)c3cc(CNc4ccccc4)cnc13 | Reverse transcriptase |
| CHEMBL929 | CC(C)Nc1cccnc1N2CCN(CC2)C(=O)c3cc4cc(NS(=O)(=O)C)ccc4[nH]3.CS(=O)(=O)O | Reverse transcriptase |
| CHEMBL83140 | CC(C)SC1=NC(=C(C)C(=O)N1)Cc2c(F)cccc2F | Reverse transcriptase |
| CHEMBL87451 | CC(C)SC1=NC(=CC(=O)N1)Cc2c(F)cccc2F | Reverse transcriptase |
| CHEMBL87235 | CCC(C)Sc1nc(O)c(C)c(Cc2c(F)cccc2F)n1 | Reverse transcriptase |
| CHEMBL101412 | CCN1c2ncccc2N(C)C(=O)c3cc(CCc4ccccc4)cnc13 | Reverse transcriptase |
| CHEMBL400546 | Cc1ccc(NC(=S)NCCc2ccccn2)nc1 | Reverse transcriptase |
| CHEMBL422123 | CN1C(=O)c2cccnc2N(C3CC3)c4nc(ccc14)c5cc[nH]c5 | Reverse transcriptase |
| CHEMBL44592 | CN(C)CCCOc1cc(C)c(NC(=O)Cc2ccc(Cl)cc2C(=O)c3ccccc3)c(C)c1 | Reverse transcriptase |
| CHEMBL123779 | Cc1cccc(CSc2nc(N)cc(Cl)n2)c1 | Reverse transcriptase |
| CHEMBL444314 | CC1=CN([C@H]2CC[C@@H](CO)O2)C(=O)NC1=O | Reverse transcriptase |
| CHEMBL142853 | CN1C(=O)c2cccnc2N(C3CC3)c4nc(ccc14)c5cn[nH]c5 | Reverse transcriptase |
| CHEMBL266007 | COC(=O)N[C@@H](C(C)C)C(=O)N[C@@H](Cc1ccccc1)[C@@H](O)CN(Cc2ccc(cc2)c3ccccc3)NC(=O)[C@@H](NC(=O)OC)C(C)(C)C | Reverse transcriptase |
| CHEMBL173575 | CCc1cc(CCc2oc3c(C)ccc(C)c3n2)c(S)nc1C | Reverse transcriptase |
| CHEMBL88966 | Fc1ccccc1C(=O)CCNC(=O)Nc2ccc(Br)cn2 | Reverse transcriptase |
| CHEMBL68137 | Oc1c(Cl)ccc2NC(=O)NC(C#CC3CC3)(c12)C(F)(F)F | Reverse transcriptase |
| CHEMBL208057 | Cc1cc(C)c(c(C)c1)n2nnnc2SCC(=O)Nc3ccc(cc3C)S(=O)(=O)N | Reverse transcriptase |
| CHEMBL62104 | CCC#C[C@@]1(NC(=O)Nc2ccc(F)cc12)C(F)(F)F | Reverse transcriptase |
| CHEMBL99992 | CCc1cc(NCc2oc3c(C)cccc3n2)c(O)nc1C | Reverse transcriptase |
| CHEMBL56052 | CN1C(=O)c2cccnc2Oc3cc(C)cc(C#N)c13 | Reverse transcriptase |
| CHEMBL207746 | Cc1cc(C)c(c(C)c1)n2nnnc2SCC(=O)Nc3ccc(cc3C)C(=O)N | Reverse transcriptase |
| CHEMBL123636 | CCc1cc(NCc2oc3c(Cl)ccc(Cl)c3c2)c(O)nc1C | Reverse transcriptase |
| CHEMBL56110 | CN1C(=O)c2ccccc2Oc3cc(C)cc(C)c13 | Reverse transcriptase |
| CHEMBL176463 | CCc1cc(CCC2=NC3CCCCC3O2)c(O)nc1C | Reverse transcriptase |
| CHEMBL125232 | CCc1cc(N(C)Cc2oc3c(Cl)ccc(Cl)c3n2)c(O)nc1C | Reverse transcriptase |
| CHEMBL124441 | CCCc1cc(NCN2C(=O)c3ccccc3C2=O)c(O)nc1C | Reverse transcriptase |
| CHEMBL324108 | COC(=O)N[C@H](C(=O)N[C@@H](Cc1ccccc1)[C@@H](O)CN(Cc2ccc(cc2)c3ccccc3)NC(=O)[C@@H](NC(=O)OC)C(C)(C)C)C(C)(C)C | Reverse transcriptase |
| CHEMBL59458 | Fc1ccc2NC(=O)N[C@@](C#CC3CC3)(c2c1Cl)C(F)(F)F | Reverse transcriptase |
| CHEMBL322869 | CCN1c2ncccc2N(C)C(=O)c3cc(CCc4ccc(N)cc4)cnc13 | Reverse transcriptase |
| CHEMBL100934 | CCN1c2nc(Cl)ccc2N(C)C(=O)c3cc(CCc4cccc(NC(=O)N)c4)cnc13 | Reverse transcriptase |
| CHEMBL335202 | Clc1ccc(NC(=S)NCCc2occc2C#N)nc1 | Reverse transcriptase |
| CHEMBL145049 | O=C1Nc2c(CNc3ccccc3)ccnc2N(C4CC4)c5ncccc15 | Reverse transcriptase |
| CHEMBL157741 | CCC(C)Sc1nc(O)c(C)c(Cc2c(Cl)cccc2Cl)n1 | Reverse transcriptase |
| CHEMBL323072 | CCN1c2ncc(CS(=O)(=O)c3ccccc3)cc2C(=O)N(C)c4ccc(Cl)nc14 | Reverse transcriptase |
| CHEMBL253994 | Fc1ccccc1CCNC(=S)Nc2nccs2 | Reverse transcriptase |
| CHEMBL254414 | CCOc1cccc(CCNC(=S)Nc2nccs2)c1 | Reverse transcriptase |
| CHEMBL66434 | CC1Cn2c(S)nc3cccc(CN1CC4CC4)c23 | Reverse transcriptase |
| CHEMBL329728 | CCN1c2ncc(CNc3cccc(N)c3)cc2C(=O)N(C)c4ccc(Cl)nc14 | Reverse transcriptase |
| CHEMBL100136 | CCN1c2ncc(CCc3ccnc(C)c3)cc2C(=O)N(C)c4ccc(Cl)nc14 | Reverse transcriptase |
| CHEMBL191502 | CCN1c2ncccc2N(C)C(=O)c3cc(CCOc4cccc5c(cccc45)C(=O)O)cnc13 | Reverse transcriptase |
| CHEMBL292608 | CCN1C(=O)c2cc(N)ccc2Oc3cccc(C)c13 | Reverse transcriptase |
| CHEMBL336635 | CCc1cccnc1N(C)C2CCN(CC2)C(=O)c3cc4cc(NS(=O)(=O)C)ccc4[nH]3 | Reverse transcriptase |
| CHEMBL176972 | CCc1cc(CCc2oc3cccc(Cl)c3n2)c(O)nc1C | Reverse transcriptase |
| CHEMBL64722 | Fc1cccc2NC(=O)N[C@@](C#CC3CC3)(c12)C(F)(F)F | Reverse transcriptase |
| CHEMBL70719 | CCN1c2cc(Cl)c(Cl)cc2N(C)C(=O)c3cccnc13 | Reverse transcriptase |
| CHEMBL124279 | Nc1cc(Cl)nc(SCc2ccc(Cl)c(Cl)c2)n1 | Reverse transcriptase |
| CHEMBL300608 | CN1C(=O)c2cc(O)ccc2Oc3cc(C)cc(C)c13 | Reverse transcriptase |
| CHEMBL372241 | CCN1c2ncccc2N(C)C(=O)c3cc(CCOc4ccc5c(cccc5c4)C(=O)O)cnc13 | Reverse transcriptase |
| CHEMBL309462 | Clc1ccc2NC(=O)NC(C#Cc3cccnc3)(C4CC4)c2c1 | Reverse transcriptase |
| CHEMBL339090 | CCc1cc(NCc2oc3c(Cl)cccc3n2)c(O)nc1C | Reverse transcriptase |
| CHEMBL82856 | CCc1cc(CCc2cc(CC)c(C)nc2OC)c(O)nc1C | Reverse transcriptase |
| CHEMBL3038087 | CC[C@@H]1NC(=O)[C@H]([C@H](O)[C@H](C)C\C=C\C)N(C)C(=O)[C@H](C(C)C)N(C)C(=O)[C@H](CC(C)C)N(C)C(=O)[C@H](CC(C)C)N(C)C(=O)[C@@H](C)NC(=O)[C@H](C)NC(=O)[C@H](CC(C)C)N(C)C(=O)[C@@H](NC(=O)[C@H](CC(C)C)N(C)C(=O)[C@@H](SC)N(C)C1=O)C(C)C | Reverse transcriptase |
| CHEMBL60545 | Fc1ccc2NC(=O)N[C@@](C#Cc3ccccn3)(c2c1F)C(F)(F)F | Reverse transcriptase |
| CHEMBL315290 | CCc1cc(NCc2nc(CC)c(C)cc2OC)c(O)nc1C | Reverse transcriptase |
| CHEMBL340381 | Nc1cc(Cl)nc(SC\C=C\c2ccccc2)n1 | Reverse transcriptase |
| CHEMBL343883 | CCN1c2nc(ccc2N(C)C(=O)c3cccnc13)c4cccc(N)c4 | Reverse transcriptase |
| CHEMBL66947 | COc1c(Cl)ccc2NC(=O)NC(C#CC3CC3)(c12)C(F)(F)F | Reverse transcriptase |
| CHEMBL79403 | C[C@@H]1Cn2c(S)nc3cccc(CN1CC=C(C)C)c23 | Reverse transcriptase |
| CHEMBL15411 | Cc1cc(C)c2NC(=O)c3cccnc3N(C4CC4)c2n1 | Reverse transcriptase |
| CHEMBL333128 | CN(C)C(=O)\C=C\CSc1nc(N)cc(Cl)n1 | Reverse transcriptase |
| CHEMBL135804 | Cn1cccc1CC\N=C(/S)\Nc2ccc(cn2)C#N | Reverse transcriptase |
| CHEMBL145322 | CCN1c2nc(ccc2N(C)C(=O)c3cccnc13)N(C)C | Reverse transcriptase |
| CHEMBL330180 | CCN1c2ncc(CSc3[nH]cnc4ncnc34)cc2C(=O)N(C)c5ccc(Cl)nc15 | Reverse transcriptase |
| CHEMBL103808 | CCN1c2ncc(C[S+]([O-])c3ccccc3)cc2C(=O)N(C)c4ccc(Cl)nc14 | Reverse transcriptase |
| CHEMBL357221 | CCN1c2nc(ccc2N(C)C(=O)c3cccnc13)c4ccc[nH]4 | Reverse transcriptase |
| CHEMBL173790 | Fc1cccc(F)c1CC2=CC(=O)NC(=N2)NC3CCCC3 | Reverse transcriptase |
| CHEMBL85574 | CCc1cc(NCc2nc(C)c(C)cc2OC)c(O)nc1C | Reverse transcriptase |
| CHEMBL99624 | CCN1c2nc(Cl)ccc2N(C)C(=O)c3cc(COc4ccncc4)cnc13 | Reverse transcriptase |
| CHEMBL46961 | C[C@H]1CN2C(=S)Nc3cccc(CN1CC=C(C)C)c23 | Reverse transcriptase |
| CHEMBL144604 | Oc1ccc(CC\N=C(/S)\Nc2ccc(Br)cn2)cc1 | Reverse transcriptase |
| CHEMBL2092834 | OP(=O)(O)OP(=O)(O)OP(=O)(O)OC[C@@H]1O[C@@H](C[C@H]1F)N2C=CC(=O)NC2=O | Reverse transcriptase |
| CHEMBL3038089 | CC[C@@H]1NC(=O)[C@H]([C@H](O)[C@H](C)C\C=C\C)N(C)C(=O)[C@H](C(C)C)N(C)C(=O)[C@H](CC(C)C)N(C)C(=O)[C@H](CC(C)C)N(C)C(=O)[C@@H](C)NC(=O)[C@H](C)NC(=O)[C@H](CC(C)C)N(C)C(=O)[C@@H](NC(=O)[C@H](CC(C)C)N(C)C(=O)[C@@H](SCCO)N(C)C1=O)C(C)C | Reverse transcriptase |
| CHEMBL156340 | Cc1c(O)nc(SC2CCCCC2)nc1Cc3c(F)cccc3F | Reverse transcriptase |
| CHEMBL3143102 | NC(=S)c1cn([C@@H]2O[C@H](CO)[C@@H](O)[C@@H]2F)c3ncnc(N)c13 | Reverse transcriptase |
| CHEMBL436200 | CCc1cc(NCc2oc3c(F)ccc(F)c3n2)c(O)nc1C | Reverse transcriptase |
| CHEMBL293332 | CCN1C(=O)c2cc(N)cnc2Oc3cc(C)c(C)cc13 | Reverse transcriptase |
| CHEMBL84217 | Cc1ccc(cc1)S(=O)(=O)c2c([nH]c3ccc(Cl)cc23)C(=O)N | Reverse transcriptase |
| CHEMBL3038088 | CC[C@@H]1NC(=O)[C@H]([C@H](O)[C@H](C)C\C=C\C)N(C)C(=O)[C@H](C(C)C)N(C)C(=O)[C@H](CC(C)C)N(C)C(=O)[C@H](CC(C)C)N(C)C(=O)[C@@H](C)NC(=O)[C@H](C)NC(=O)[C@H](CC(C)C)N(C)C(=O)[C@@H](NC(=O)[C@H](CC(C)C)N(C)C(=O)[C@@H](SCCN2CCCCC2)N(C)C1=O)C(C)C | Reverse transcriptase |
| CHEMBL325271 | COC(=O)N[C@H](C(=O)N[C@@H](Cc1ccccc1)[C@@H](O)CN(Cc2ccc(cc2)c3nnn(n3)C(C)(C)C)NC(=O)[C@@H](NC(=O)OC)C(C)(C)C)C(C)(C)C | Reverse transcriptase |
| CHEMBL299334 | CSCCN1C(=O)c2ccccc2Oc3nc(Cl)ccc13 | Reverse transcriptase |
| CHEMBL293037 | FC(F)(F)[C@]1(NC(=O)Nc2cccc(Cl)c12)C#CC3CC3 | Reverse transcriptase |
| CHEMBL292711 | CCC#C[C@@]1(NC(=O)Nc2ccc(F)c(F)c12)C(F)(F)F | Reverse transcriptase |
| CHEMBL62039 | Fc1ccc2NC(=O)N[C@@](C#CC3CC3)(c2c1F)C(F)(F)F | Reverse transcriptase |
| CHEMBL105859 | COC(=O)CCC\C=C(\c1ccc(OC)c(OC)c1)/c2cc(C)c(OC)c(c2)C(=O)OC | Reverse transcriptase |
| CHEMBL98159 | CCc1cc(CCc2oc3ccccc3c2)c(O)nc1C | Reverse transcriptase |
| CHEMBL320078 | Fc1cccc2NC(=O)OC(C#CC3CC3)(c12)C(F)(F)F | Reverse transcriptase |
| CHEMBL65881 | CC1N(CC=C(C)C)CCN2C(=S)Nc3cccc1c23 | Reverse transcriptase |
| CHEMBL107180 | CCc1c(O)nc(SCC(=O)c2ccc(F)cc2)nc1Cc3cccc4ccccc34 | Reverse transcriptase |
| CHEMBL64521 | CC(C)C#C[C@@]1(NC(=O)Nc2ccc(F)c(Cl)c12)C(F)(F)F | Reverse transcriptase |
| CHEMBL324237 | CC(C)c1c(O)nc(SCC(=O)c2ccc(F)cc2)nc1Cc3cccc4ccccc34 | Reverse transcriptase |
| CHEMBL3038086 | CC[C@@H]1NC(=O)[C@H]([C@H](O)[C@H](C)C\C=C\C)N(C)C(=O)[C@H](C(C)C)N(C)C(=O)[C@H](CC(C)C)N(C)C(=O)[C@H](CC(C)C)N(C)C(=O)[C@@H](C)NC(=O)[C@H](C)NC(=O)[C@H](CC(C)C)N(C)C(=O)[C@@H](NC(=O)[C@H](CC(C)(C)O)N(C)C(=O)[C@@H](SCCO)N(C)C1=O)C(C)C | Reverse transcriptase |
| CHEMBL320969 | [Na+].[Na+].[Na+].[Na+].CC1=CN([C@@H]2O[C@H](COP(=O)([O-])OP(=O)([O-])OP(=O)([O-])[O-])C=C2)C(=O)NC1=O | Reverse transcriptase |
| CHEMBL309899 | CC1=C(Cc2c(F)cccc2F)N=C(NC1=O)SC3CCCC3 | Reverse transcriptase |
| CHEMBL312874 | Fc1cccc(F)c1CC2=CC(=O)NC(=N2)SC3CCCC3 | Reverse transcriptase |
| CHEMBL313998 | COc1cccc(c1)C(=O)CC\N=C(/S)\Nc2ccc(Br)cn2 | Reverse transcriptase |
| CHEMBL445136 | CCNc1ccc(OCc2ccnc3N(C4CC4)c5ncccc5C(=O)Nc23)cc1 | Reverse transcriptase |
| CHEMBL421210 | CCN1c2ncccc2N(C)C(=O)c3cc(CCc4ccc5ccccc5c4)cnc13 | Reverse transcriptase |
| CHEMBL417394 | Fc1ccc2NC(=O)N[C@@](C#Cc3ccccn3)(c2c1Cl)C(F)(F)F | Reverse transcriptase |
| CHEMBL254207 | Cc1ccccc1CCNC(=S)Nc2nccs2 | Reverse transcriptase |
| CHEMBL103593 | CCN1c2ncccc2N(C)C(=O)c3cc(CCc4ccccc4N)cnc13 | Reverse transcriptase |
| CHEMBL318952 | CCN1c2nc(Cl)ccc2N(C)C(=O)c3cc(CCc4cccc(c4)N=[N+]=[N-])cnc13 | Reverse transcriptase |
| CHEMBL1203237 | Cl.CSc1ccc2c(C)nc(CCCCCCC(=O)c3ccccc3)n2n1 | Reverse transcriptase |
| CHEMBL3038092 | CC[C@@H]1NC(=O)[C@H]([C@H](O)[C@H](C)C\C=C\C)N(C)C(=O)[C@H](C(C)C)N(C)C(=O)[C@H](CC(C)C)N(C)C(=O)[C@H](CC(C)C)N(C)C(=O)[C@@H](C)NC(=O)[C@H](C)NC(=O)[C@H](CC(C)C)N(C)C(=O)[C@@H](NC(=O)[C@H](CC(C)C)N(C)C(=O)[C@@H](SCCn2ccnc2)N(C)C1=O)C(C)C | Reverse transcriptase |
| CHEMBL2114369 | C[C@@H]1O[C@@](C#CC2CC2)(c3cc(Cl)ccc3NC1=O)C(F)(F)F | Reverse transcriptase |
| CHEMBL173589 | CCCc1cc(CCc2oc3ccccc3n2)c(O)nc1C | Reverse transcriptase |
| CHEMBL317992 | Fc1ccc2NC(=O)OC(C#CC3CC3)(c2c1F)C(F)(F)F | Reverse transcriptase |
| CHEMBL2407585 | CC[C@@H]1NC(=O)[C@H]([C@H](O)[C@H](C)C\C=C\C)N(C)C(=O)[C@H](C(C)C)N(C)C(=O)[C@H](CC(C)C)N(C)C(=O)[C@H](CC(C)C)N(C)C(=O)[C@@H](C)NC(=O)[C@H](C)NC(=O)[C@H](CC(C)C)N(C)C(=O)[C@@H](NC(=O)[C@H](CC(C)(C)O)N(C)C(=O)[C@@H](SC)N(C)C1=O)C(C)C | Reverse transcriptase |
| CHEMBL84900 | Cc1cc(C)cc(c1)C23SCCN2C(=O)c4ccccc34 | Reverse transcriptase |
| CHEMBL113824 | COC(=O)N[C@@H](C(C)C)C(=O)NN(C[C@H](O)[C@H](Cc1ccccc1)NC(=O)[C@@H](NC(=O)OC)C(C)(C)C)Cc2ccc(cc2)c3ccccc3 | Reverse transcriptase |
| CHEMBL176631 | CCc1cc(OCc2oc3c(Cl)ccc(Cl)c3n2)c(O)nc1C | Reverse transcriptase |
| CHEMBL122343 | CN(C1CCN(CC1)C(=O)c2cc3cc(NS(=O)(=O)C)ccc3[nH]2)c4ncccc4NC5CC5 | Reverse transcriptase |
| CHEMBL262184 | CCN1c2ncc(COc3ccc4[nH]ccc4c3)cc2C(=O)N(C)c5ccc(Cl)nc15 | Reverse transcriptase |
| CHEMBL156637 | CC(C)CSc1nc(O)c(C)c(Cc2c(F)cccc2F)n1 | Reverse transcriptase |
| CHEMBL144820 | CN1C(=O)c2cccnc2N(C3CC3)c4nc(Cl)ccc14 | Reverse transcriptase |
| CHEMBL400685 | COc1cccc(OC)c1CCNC(=S)Nc2nccs2 | Reverse transcriptase |
| CHEMBL160869 | CCCCSc1nc(O)c(C)c(Cc2c(F)cccc2F)n1 | Reverse transcriptase |
| CHEMBL101501 | CCN1c2ncccc2N(C)C(=O)c3cc(CCC(=O)OC)cnc13 | Reverse transcriptase |
| CHEMBL156255 | Oc1cc(Cc2c(F)cccc2F)nc(SC3CCCCC3)n1 | Reverse transcriptase |
| CHEMBL318982 | CCN1c2nc(I)ccc2N(C)C(=O)c3cc(CCc4ccncc4)cnc13 | Reverse transcriptase |
| CHEMBL303446 | FC(F)(F)C12NC(=O)Nc3ccc(Cl)c(OC(=C1)C4CC4)c23 | Reverse transcriptase |
| CHEMBL135179 | Cn1cccc1CC\N=C(/S)\Nc2ccc(Cl)cn2 | Reverse transcriptase |
| CHEMBL292188 | CCN1C(=S)c2cccnc2Oc3cc(C)cc(C)c13 | Reverse transcriptase |
| CHEMBL191910 | Ic1ccc(NC(=S)OCCN2C(=O)c3ccccc3C2=O)cc1 | Reverse transcriptase |
| CHEMBL83632 | Clc1ccc2C(=O)N3CCS[C@]3(c4ccccc4)c2c1 | Reverse transcriptase |
| CHEMBL343625 | CCN1c2ncccc2C(=O)Nc3c(C)cc(NCCCO)nc13 | Reverse transcriptase |
| CHEMBL358947 | CCN1c2nc(CC)cc(C)c2NC(=O)c3cccnc13 | Reverse transcriptase |
| CHEMBL37343 | CCN1c2ncccc2N(C)C(=O)c3cccnc13 | Reverse transcriptase |
| CHEMBL341847 | CCN1c2nc(NCCO)cc(C)c2NC(=O)c3cccnc13 | Reverse transcriptase |
| CHEMBL422659 | CCN1c2ncccc2C(=O)N(C)c3ccc(nc13)n4cccc4 | Reverse transcriptase |
| CHEMBL312896 | CCc1cc(CNc2cc(CC)c(C)nc2O)c(O)nc1C | Reverse transcriptase |
| CHEMBL62179 | CC(C)C#C[C@@]1(NC(=O)Nc2ccc(F)c(F)c12)C(F)(F)F | Reverse transcriptase |
| CHEMBL125299 | CCc1cc(NCc2oc3c(F)cccc3n2)c(O)nc1C | Reverse transcriptase |
| CHEMBL2114370 | C[C@@H]1O[C@@](C#CC2CC2)(c3cc(F)ccc3NC1=O)C(F)(F)F | Reverse transcriptase |
| CHEMBL37842 | CCN1c2nccc(Cl)c2NC(=O)c3cccnc13 | Reverse transcriptase |
| CHEMBL193125 | CCN1c2ncccc2N(C)C(=O)c3cc(CCOc4ccc(cc4)C(=O)O)cnc13 | Reverse transcriptase |
| CHEMBL405301 | CC[C@@H]1NC(=O)C([C@H](O)[C@H](C)C\C=C\C)N(C)C(=O)[C@H](C(C)C)N(C)C(=O)[C@H](CC(C)C)N(C)C(=O)[C@H](CC(C)C)N(C)C(=O)[C@@H](C)NC(=O)[C@H](C)NC(=O)[C@H](CC(C)C)N(C)C(=O)[C@@H](NC(=O)[C@H](CC(C)(C)O)N(C)C(=O)[C@@H](CO)N(C)C1=O)C(C)C | Reverse transcriptase |
| CHEMBL174364 | Cc1cc(CCc2oc3c(Cl)ccc(Cl)c3n2)c(O)nc1C | Reverse transcriptase |
| CHEMBL336807 | CCN(C1CCN(CC1)C(=O)c2cc3cc(NS(=O)(=O)C)ccc3[nH]2)c4ncccc4CC | Reverse transcriptase |
| CHEMBL295678 | OC(c1ccccc1)c2cc(Cl)ccc2OCC(=O)Nc3ccccc3 | Reverse transcriptase |
| CHEMBL140532 | Cc1cc(C)cc(Sc2c(C)cnc(O)c2N)c1 | Reverse transcriptase |
| CHEMBL240416 | COc1ccc(OC)c(CCNC(=S)Nc2ccc(Br)cn2)c1 | Reverse transcriptase |
| CHEMBL1160260 | CC1=CN([C@H]2C[C@@H](N=[N+]=[N-])[C@@H](OP(=O)(O)OP(=O)(O)NP(=O)(O)O)O2)C(=O)NC1=O | Reverse transcriptase |
| CHEMBL611850 | CCCCCCCC(=O)NC1=NN(C)c2ncnc3c2c1cn3C4O[C@H](CO)[C@@H](O)[C@H]4O | Reverse transcriptase |
| CHEMBL434441 | Cc1cncn1c2ccc3c(C)nc(CCCCCCC(=O)c4ccccc4)n3n2 | Reverse transcriptase |
| CHEMBL46981 | Clc1ccc(OC\C=C\c2ccccc2)c(c1)C(=O)c3ccccc3 | Reverse transcriptase |
| CHEMBL146550 | CCN1c2ncccc2C(=O)N(C)c3ccc(nc13)c4ccsc4 | Reverse transcriptase |
| CHEMBL317130 | CCN1c2ncccc2N(C)C(=O)c3cc(N)cnc13 | Reverse transcriptase |
| CHEMBL135662 | Cc1cc2nn(CC\N=C(/S)\Nc3ccc(Br)cn3)nc2cc1C | Reverse transcriptase |
| CHEMBL125532 | Cc1nc(CCCCCCC(=O)c2ccccc2)n3nc(ccc13)n4cncn4 | Reverse transcriptase |
| CHEMBL144653 | CCN1c2nc(ccc2N(C)C(=O)c3cccnc13)c4cccc(O)c4 | Reverse transcriptase |
| CHEMBL357183 | CCN1c2ncccc2C(=O)N(C)c3ccc(nc13)c4nccs4 | Reverse transcriptase |
| CHEMBL433172 | CCN1c2ncc(COc3cc[n+]([O-])c(C)c3)cc2C(=O)N(C)c4ccc(Cl)nc14 | Reverse transcriptase |
| CHEMBL359195 | Nc1ccc(OCc2ccnc3N(C4CC4)c5ncccc5C(=O)Nc23)cc1 | Reverse transcriptase |
| CHEMBL318635 | CCN1c2ncccc2N(C)C(=O)c3cc(CCc4ccc(C)cc4)cnc13 | Reverse transcriptase |
| CHEMBL46865 | Clc1ccc(OCCNc2ccccc2)c(c1)C(=O)c3ccccc3 | Reverse transcriptase |
| CHEMBL99793 | CCN1c2nc(Cl)ccc2N(C)C(=O)c3cc(COc4cccc(OC)c4)cnc13 | Reverse transcriptase |
| CHEMBL44845 | Clc1ccc(OCC(=O)Nc2ccccc2)c(C(=O)c3ccccc3)c1Cl | Reverse transcriptase |
| CHEMBL100903 | CCN1c2ncc(COc3ccccc3O)cc2C(=O)N(C)c4ccc(Cl)nc14 | Reverse transcriptase |
| CHEMBL128931 | CCc1c(C)nc(O)c(N)c1C(=O)c2cc(C)cc(C)c2 | Reverse transcriptase |
| CHEMBL46446 | Clc1cc(Cl)c(OCC(=O)Nc2ccccc2)c(c1)C(=O)c3ccccc3 | Reverse transcriptase |
| CHEMBL46627 | CN(C)CCCOc1ccc(NC(=O)c2ccc(Cl)cc2C(=O)c3ccccc3)c(C)c1 | Reverse transcriptase |
| CHEMBL57733 | CCc1c(C)nc(O)c(N)c1Cc2cc(C)cc(C)c2 | Reverse transcriptase |
| CHEMBL154913 | CCC(C)Sc1nc(O)cc(Cc2c(Cl)cccc2Cl)n1 | Reverse transcriptase |
| CHEMBL254628 | Cc1csc(NC(=S)NCCc2ccccc2)n1 | Reverse transcriptase |
| CHEMBL40562 | COc1ccc(C(=O)C)c(F)c1CCNC(=O)Nc2ccc(Cl)cn2 | Reverse transcriptase |
| CHEMBL3038093 | CC[C@@H]1NC(=O)[C@H]([C@H](O)[C@H](C)C\C=C\C)N(C)C(=O)[C@H](C(C)C)N(C)C(=O)[C@H](CC(C)C)N(C)C(=O)[C@H](CC(C)C)N(C)C(=O)[C@@H](C)NC(=O)[C@H](C)NC(=O)[C@H](CC(C)C)N(C)C(=O)[C@@H](NC(=O)[C@H](CC(C)C)N(C)C(=O)[C@@H](SCCN2CCOCC2)N(C)C1=O)C(C)C | Reverse transcriptase |
| CHEMBL108154 | CC(C)C#CC1(OC(=O)Nc2cccc(F)c12)C(F)(F)F | Reverse transcriptase |
| CHEMBL305345 | Fc1c(Cl)ccc2NC(=O)N[C@@](C#CC3CC3)(c12)C(F)(F)F | Reverse transcriptase |
| CHEMBL124430 | CCc1cc(N(C)Cc2oc3c(C)ccc(C)c3n2)c(O)nc1C | Reverse transcriptase |
| CHEMBL122293 | CCc1cc(NCc2oc3c(Cl)ccc(F)c3n2)c(O)nc1C | Reverse transcriptase |
| CHEMBL104658 | CCCOC(C#CC1CC1)(c2cc(CC)c(C)nc2O)C(F)(F)F | Reverse transcriptase |
| CHEMBL29171 | FC(F)(F)C1(OCC(=O)Nc2ccc(Cl)cc12)\C=C\C3CC3 | Reverse transcriptase |
| CHEMBL49316 | CC(C)OC(=O)N1c2ccccc2NC(=O)C1(C#CC3CC3)C(F)(F)F | Reverse transcriptase |
| CHEMBL48628 | COc1ccc2NC(=O)C(C#CC3CC3)(N(CC4CC4)c2c1)C(F)(F)F | Reverse transcriptase |
| CHEMBL124786 | CCc1cc(NCc2oc3cccc(F)c3n2)c(O)nc1C | Reverse transcriptase |
| CHEMBL356998 | CCN1c2ncccc2C(=O)N(C)c3ccc(nc13)c4ocnc4 | Reverse transcriptase |
| CHEMBL144061 | C=Cc1ccnc2N(C3CC3)c4ncccc4C(=O)Nc12 | Reverse transcriptase |
| CHEMBL39792 | CCN1c2ncccc2C(=O)N(C)c3c(CC)ccnc13 | Reverse transcriptase |
| CHEMBL319032 | CCN1c2ncccc2N(C)C(=O)c3cc(CCc4cccc(C)c4)cnc13 | Reverse transcriptase |
| CHEMBL102163 | CCN1c2ncc(CCc3cccc(C)c3)cc2C(=O)N(C)c4ccc(Cl)nc14 | Reverse transcriptase |
| CHEMBL144652 | CCN1c2ncccc2C(=O)N(C)c3ccc(nc13)c4occc4 | Reverse transcriptase |
| CHEMBL145273 | CCN1c2nc(O)cc(COCc3ccccc3)c2NC(=O)c4cccnc14 | Reverse transcriptase |
| CHEMBL293894 | CCC#C[C@@]1(NC(=O)Nc2ccc(Cl)cc12)C(F)(F)F | Reverse transcriptase |
| CHEMBL73672 | CCN1c2nccc(CC)c2NC(=O)c3cccnc13 | Reverse transcriptase |
| CHEMBL420519 | Cc1nc(O)c(NCc2oc3c(Cl)ccc(Cl)c3n2)c4Cc14 | Reverse transcriptase |
| CHEMBL126634 | COc1cc(NCc2oc3c(Cl)ccc(Cl)c3n2)c(O)nc1C | Reverse transcriptase |
| CHEMBL318720 | CCN1c2ncccc2N(C)C(=O)c3cc(Cc4ccccc4)cnc13 | Reverse transcriptase |
| CHEMBL99018 | CCc1cc(NCc2oc3cccc(C)c3n2)c(O)nc1C | Reverse transcriptase |
| CHEMBL294179 | Nc1cccc(Sc2cc(Cl)cc(Cl)c2)c1C#N | Reverse transcriptase |
| CHEMBL102960 | CCN1c2ncccc2N(C)C(=O)c3cc(CCc4cccc(F)c4)cnc13 | Reverse transcriptase |
| CHEMBL413252 | CCN1c2ncc(COc3cc[n+]([O-])c(c3)C(F)(F)F)cc2C(=O)N(C)c4ccc(Cl)nc14 | Reverse transcriptase |
| CHEMBL144642 | O=C1Nc2c(COc3ccccc3)ccnc2N(C4CC4)c5ncccc15 | Reverse transcriptase |
| CHEMBL330597 | CCN1c2ncccc2N(C)C(=O)c3cc(CCCc4ccccc4)cnc13 | Reverse transcriptase |
| CHEMBL143107 | COc1ccc2N(C)C(=O)c3cccnc3N(C4CC4)c2n1 | Reverse transcriptase |
| CHEMBL288305 | CCN1c2ncccc2C(=O)N(C)c3ccc(C)nc13 | Reverse transcriptase |
| CHEMBL297897 | CCOC(=O)N1c2cc(F)ccc2NC(=O)C1(C#CC3CC3)C(F)(F)F | Reverse transcriptase |
| CHEMBL69005 | FC(F)(F)C12NC(=O)Nc3ccc(Cl)c(OC(=C1)c4cccnc4)c23 | Reverse transcriptase |
| CHEMBL93984 | COc1cc(Cl)cc2c1NC(=O)OC2(C#CC3CC3)C(F)(F)F | Reverse transcriptase |
| CHEMBL48376 | COc1ccc2NC(=O)C(C#CC3CC3)(N(CC=C)c2c1)C(F)(F)F | Reverse transcriptase |
| CHEMBL295606 | Fc1ccc2NC(=O)C(C#CC3CC3)(N(CC=C)c2c1)C(F)(F)F | Reverse transcriptase |
| CHEMBL64467 | COc1ccc2NC(=O)N[C@@](C#CC3CC3)(c2c1)C(F)(F)F | Reverse transcriptase |
| CHEMBL430601 | Fc1ccc2NC(=O)N[C@@](C#Cc3ccccn3)(c2c1)C(F)(F)F | Reverse transcriptase |
| CHEMBL299394 | CCOC(=O)N1c2cc(Cl)ccc2NC(=O)C1(C#CC3CC3)C(F)(F)F | Reverse transcriptase |
| CHEMBL324233 | CCC#CC1(OC(=O)Nc2cccc(F)c12)C(F)(F)F | Reverse transcriptase |
| CHEMBL50550 | FC(F)(F)C1(C#CC2CC2)N(CC3CC3)c4ccccc4NC1=O | Reverse transcriptase |
| CHEMBL122284 | CCCCc1cc(NCN2C(=O)c3ccccc3C2=O)c(O)nc1C | Reverse transcriptase |
| CHEMBL287650 | CC1OC(\C=C\C2CC2)(c3cc(F)ccc3NC1=O)C(F)(F)F | Reverse transcriptase |
| CHEMBL57113 | FC(F)(F)[C@@]1(C#CC2CC2)[C@@H](OCc3ccncc3)C(=O)Nc4ccc(Cl)cc14 | Reverse transcriptase |
| CHEMBL302622 | FC(F)(F)[C@]1(NC(=O)Nc2ccc(Cl)cc12)C#Cc3ccccn3 | Reverse transcriptase |
| CHEMBL107693 | CCCCOC(C#CC1CC1)(c2cc(CC)c(C)nc2O)C(F)(F)F | Reverse transcriptase |
| CHEMBL293736 | FC(F)(F)[C@]1(CC(=O)Nc2ccc(Cl)cc12)C#CC3CC3 | Reverse transcriptase |
| CHEMBL99845 | CCN1c2ncc(COc3cccc(O)c3)cc2C(=O)N(C)c4ccc(Cl)nc14 | Reverse transcriptase |
| CHEMBL422798 | C[C@H]1Oc2c3C=CC(C)(C)Oc3c4C(=CC(=O)Oc4c2[C@H](O)[C@@H]1C)c5ccccc5 | Reverse transcriptase |
| CHEMBL310306 | Clc1ccc2NC(=O)NC(C#Cc3ncccn3)(C4CC4)c2c1 | Reverse transcriptase |
| CHEMBL126107 | CCCNc1cccnc1N(CC)C2CCN(CC2)C(=O)c3cc4cc(NS(=O)(=O)C)ccc4[nH]3 | Reverse transcriptase |
| CHEMBL431936 | CC(C)OC(=O)N1c2cc(F)ccc2NC(=O)C1(C#CC3CC3)C(F)(F)F | Reverse transcriptase |
| CHEMBL87303 | O=C1N2CCSC2(c3ccccc13)c4cccc5ccccc45 | Reverse transcriptase |
| CHEMBL101979 | CCN1c2nc(Cl)ccc2N(C)C(=O)c3cc(COc4cccc(c4)C(=O)N)cnc13 | Reverse transcriptase |
| CHEMBL53451 | CCCN1C(=O)c2cc(N)ccc2Oc3cc(C)ccc13 | Reverse transcriptase |
| CHEMBL356779 | CCN1c2ncccc2C(=O)N(C)c3ccc(nc13)c4ncc[nH]4 | Reverse transcriptase |
| CHEMBL132770 | CCCN(C1CCN(CC1)C(=O)c2cc3cc(NS(=O)(=O)C)ccc3[nH]2)c4ncccc4CC | Reverse transcriptase |
| CHEMBL312397 | CCN1c2ncccc2C(=O)N(C)c3ccc(nc13)c4c[nH]c5ccncc45 | Reverse transcriptase |
| CHEMBL101944 | CCN1c2ncccc2N(C)C(=O)c3cc(CCc4cccnc4)cnc13 | Reverse transcriptase |
| CHEMBL97653 | COc1ccc2NC(=O)OC(C#CC3CC3)(c2c1)C(F)(F)F | Reverse transcriptase |
| CHEMBL299495 | CCN1C(=O)c2cccnc2Oc3cc(C)cc(N)c13 | Reverse transcriptase |
| CHEMBL432763 | Cc1ccc2NC(=O)OC(C#CC3CC3)(c2c1)C(F)(F)F | Reverse transcriptase |
| CHEMBL133779 | Oc1ccc(F)c(C2CC2NC(=O)Nc3ccc(Cl)cn3)c1Cl | Reverse transcriptase |
| CHEMBL58596 | CC(C)C#CC1(NS(=O)(=O)Nc2ccc(Cl)cc12)C(C)C | Reverse transcriptase |
| CHEMBL442519 | CC[C@@H]1NC(=O)C([C@H](O)[C@H](C)C\C=C\C)N(C)C(=O)[C@H](C(C)C)N(C)C(=O)[C@H](CC(C)C)N(C)C(=O)[C@H](CC(C)C)N(C)C(=O)[C@@H](C)NC(=O)[C@H](C)NC(=O)[C@H](CC(C)C)N(C)C(=O)[C@@H](NC(=O)[C@H](CC(C)(C)O)N(C)C(=O)CN(C)C1=O)C(C)C | Reverse transcriptase |
| CHEMBL305996 | COc1c(Cl)ccc2NC(=O)NC(C#Cc3cccnc3)(c12)C(F)(F)F | Reverse transcriptase |
| CHEMBL344288 | CCN1c2nc(ccc2N(C)C(=O)c3cccnc13)C#C | Reverse transcriptase |
| CHEMBL292891 | COc1cc(C)cc(Sc2cccc(N)c2C#N)c1 | Reverse transcriptase |
| CHEMBL145193 | CCN1c2nc(ccc2N(C)C(=O)c3cccnc13)c4cccs4 | Reverse transcriptase |
| CHEMBL327938 | CCN1c2nc(Cl)ccc2N(C)C(=O)c3cc(CCc4cccc(NS(=O)(=O)C)c4)cnc13 | Reverse transcriptase |
| CHEMBL280527 | CCNc1cccnc1N2CCN(CC2)C(=O)c3cc4cc(OC)ccc4[nH]3 | Reverse transcriptase |
| CHEMBL134106 | CCN(C1CCN(CC1)C(=O)c2cc3cc(NS(=O)(=O)C)ccc3[nH]2)c4ncccc4COC | Reverse transcriptase |
| CHEMBL132491 | COCc1cccnc1N(C)C2CCN(CC2)C(=O)c3cc4cc(NS(=O)(=O)C)ccc4[nH]3 | Reverse transcriptase |
| CHEMBL358292 | O=C1Nc2c(Cc3ccccc3)ccnc2N(C4CC4)c5ncccc15 | Reverse transcriptase |
| CHEMBL262633 | CCNc1cccnc1N(CC)C2CCN(CC2)C(=O)c3cc4cc(NC(=O)N5CCN(C)CC5)ccc4[nH]3 | Reverse transcriptase |
| CHEMBL74690 | CCN1c2cc(ccc2N(C)C(=O)c3cccnc13)N=[N+]=[N-] | Reverse transcriptase |
| CHEMBL340593 | Nc1cc(Cl)nc(SCc2cccc(Br)c2)n1 | Reverse transcriptase |
| CHEMBL331911 | CCN(C1CCN(CC1)C(=O)c2cc3cc(NC(=O)N4CCN(C)CC4)ccc3[nH]2)c5ncccc5NC(C)C | Reverse transcriptase |
| CHEMBL83524 | Clc1cccc(c1)C23SCCN2C(=O)c4ccccc34 | Reverse transcriptase |
| CHEMBL304028 | Fc1ccc2NC(=O)N[C@@](C#Cc3ccccc3)(c2c1)C(F)(F)F | Reverse transcriptase |
| CHEMBL304938 | COc1c(Cl)ccc2NC(=O)NC(C#Cc3ccccc3)(c12)C(F)(F)F | Reverse transcriptase |
| CHEMBL293077 | CCN1C(=O)c2cc(N)ccc2Oc3ccccc13 | Reverse transcriptase |
| CHEMBL420740 | CC1N(CC=C(C)C)CCN2C(=O)Nc3ccc(Cl)c1c23 | Reverse transcriptase |
| CHEMBL299978 | FC(F)(F)C1(C#CC2CC2)N(C(=O)OCC=C)c3ccccc3NC1=O | Reverse transcriptase |
| CHEMBL2114381 | CCN1c2cc(C)c(C)nc2N(C)C(=O)c3cc(N)cnc13 | Reverse transcriptase |
| CHEMBL124494 | Nc1cc(Cl)nc(SCc2ccc3ccccc3c2)n1 | Reverse transcriptase |
| CHEMBL125715 | CCCN(C1CCN(CC1)C(=O)c2cc3cc(NS(=O)(=O)C)ccc3[nH]2)c4ncccc4NCC | Reverse transcriptase |
| CHEMBL253995 | Fc1cccc(CCNC(=S)Nc2nccs2)c1 | Reverse transcriptase |
| CHEMBL400733 | COc1cccc(CCNC(=S)Nc2nccs2)c1 | Reverse transcriptase |
| CHEMBL254413 | [O-][N+](=O)c1ccccc1CCNC(=S)Nc2nccs2 | Reverse transcriptase |
| CHEMBL124526 | CCc1cc(NCc2oc3cccc(Cl)c3n2)c(O)nc1C | Reverse transcriptase |
| CHEMBL435030 | CCN1c2nc(cc(C)c2NC(=O)c3cccnc13)N4CCSCC4 | Reverse transcriptase |
| CHEMBL144290 | CCN1c2ncccc2C(=O)N(C)c3ccc(nc13)c4cccc(OC)c4 | Reverse transcriptase |
| CHEMBL315054 | O=C1N2CCS[C@@]2(c3ccccc13)c4cccc5ccccc45 | Reverse transcriptase |
| CHEMBL355918 | CN1C(=O)c2cccnc2N(C3CC3)c4nc(ccc14)c5ccncc5 | Reverse transcriptase |
| CHEMBL2368775 | COC[C@H]1O[C@H]([C@H](OC)[C@@H]1OP(=O)(C)O)n2cnc3C(=O)N(CC#C)C=Nc23 | Reverse transcriptase |
| CHEMBL101938 | CCN1c2nc(Cl)ccc2N(C)C(=O)c3cc(CCc4ccnc(c4)C(=O)OC)cnc13 | Reverse transcriptase |
| CHEMBL30752 | CCC1OC(\C=C\C2CC2)(c3cc(F)ccc3NC1=O)C(F)(F)F | Reverse transcriptase |
| CHEMBL42906 | CCN1c2ncccc2c3ncc(C)n3c4ccc(OC)nc14 | Reverse transcriptase |
| CHEMBL108750 | CCCC#CC1(OC(=O)Nc2cccc(F)c12)C(F)(F)F | Reverse transcriptase |
| CHEMBL330602 | CCN1c2ncc(CCc3ccc(N)cc3)cc2C(=O)N(C)c4ccc(Cl)nc14 | Reverse transcriptase |
| CHEMBL307994 | CC1N(CC=C(C)C)CCN2C(=O)Nc3cc(Cl)cc1c23 | Reverse transcriptase |
| CHEMBL125203 | CN(C1CCN(CC1)C(=O)c2cc3cc(NS(=O)(=O)C)ccc3[nH]2)c4ncccc4NC5(C)CC5 | Reverse transcriptase |
| CHEMBL295893 | CC(C)C(I)N1c2ccccc2NC(=O)C1(C#CC3CC3)C(F)(F)F | Reverse transcriptase |
| CHEMBL297851 | CC(C)OC(=O)N1c2cc(Cl)ccc2NC(=O)C1(C#CC3CC3)C(F)(F)F | Reverse transcriptase |
| CHEMBL69674 | CC(=CCN1CCN2C(=S)Nc3cc(Cl)cc(C1)c23)C | Reverse transcriptase |
| CHEMBL327744 | CCN1c2nc(Cl)ccc2N(C)C(=O)c3cc(CCc4ccnc(CO)c4)cnc13 | Reverse transcriptase |
| CHEMBL334129 | COc1cccc(CSc2nc(N)cc(Cl)n2)c1 | Reverse transcriptase |
| CHEMBL377310 | Cc1cc(C)c(c(C)c1)n2nnnc2SCC(=O)Nc3ccc(cc3C)[N+](=O)[O-] | Reverse transcriptase |
| CHEMBL59140 | CCCN1C(=O)c2cc(N)ccc2Oc3cc(C)c(C)cc13 | Reverse transcriptase |
| CHEMBL59917 | FC(F)(F)[C@]1(NC(=O)Nc2ccc(Cl)c(Cl)c12)C#CC3CC3 | Reverse transcriptase |
| CHEMBL50664 | CC(=C)OC(=O)N1c2ccccc2NC(=O)C1(C#CC3CC3)C(F)(F)F | Reverse transcriptase |
| CHEMBL132122 | COc1ccc2N=C(S)C(CSC)N(C(=O)OC(C)C)c2c1 | Reverse transcriptase |
| CHEMBL331790 | CC(C)Nc1cccnc1N(C)C2CCN(CC2)C(=O)c3cc4ccc(CO)cc4[nH]3 | Reverse transcriptase |
| CHEMBL316922 | CCN1c2nc(Cl)ccc2N(C)C(=O)c3cc(COc4cccc(c4)[N+](=O)[O-])cnc13 | Reverse transcriptase |
| CHEMBL103399 | CCN1c2ncc(COc3ccccc3F)cc2C(=O)N(C)c4ccc(Cl)nc14 | Reverse transcriptase |
| CHEMBL305965 | CCN1c2ccc(Cl)cc2N(C)C(=O)c3cccnc13 | Reverse transcriptase |
| CHEMBL426396 | Cc1cc(CCc2oc3ccccc3n2)c(O)nc1C | Reverse transcriptase |
| CHEMBL331330 | CCN(C1CCN(CC1)C(=O)c2cc3cc(NS(=O)(=O)C)ccc3[nH]2)c4ncccc4NC(C)C | Reverse transcriptase |
| CHEMBL69894 | CCN1c2cc(Cl)ccc2N(C)C(=O)c3cccnc13 | Reverse transcriptase |
| CHEMBL2114371 | CC[C@@H]1O[C@@](C#CC2CC2)(c3cc(F)ccc3NC1=O)C(F)(F)F | Reverse transcriptase |
| CHEMBL177256 | CCc1cc(CCc2oc3cc(C)ccc3n2)c(O)nc1C | Reverse transcriptase |
| CHEMBL281230 | CC1OC(\C=C\C2CC2)(c3c(F)c(F)ccc3NC1=O)C(F)(F)F | Reverse transcriptase |
| CHEMBL70077 | Oc1c(Cl)ccc2NC(=O)NC(C#Cc3ccccc3)(c12)C(F)(F)F | Reverse transcriptase |
| CHEMBL177191 | CCc1cnc(O)c(CCc2oc3ccccc3n2)c1 | Reverse transcriptase |
| CHEMBL324572 | CCOC(=O)N[C@@H](C(C)C)C(=O)N[C@@H](Cc1ccccc1)[C@@H](O)CN(CC2CCCCC2)NC(=O)[C@@H](NC(=O)OCC)C(C)C | Reverse transcriptase |
| CHEMBL301370 | CC[C@@H]1N(C(=O)OC(C)C)c2cc(F)ccc2NC1=O | Reverse transcriptase |
| CHEMBL539427 | Brc1ccc(NC(=N)N[C@@H]2C[C@@H]2c3ccccc3)nc1 | Reverse transcriptase |
| CHEMBL67675 | CC1Cn2c(O)nc3cc(Cl)cc(CN1CC=C(C)C)c23 | Reverse transcriptase |
| CHEMBL301022 | CN1C(=O)c2cc(N)ccc2Oc3ccccc13 | Reverse transcriptase |
| CHEMBL127564 | CCc1cc(NCc2oc3cccc(OC)c3n2)c(O)nc1C | Reverse transcriptase |
| CHEMBL125437 | CNC(=O)Nc1ccc2[nH]c(cc2c1)C(=O)N3CCC(CC3)N(C)c4ncccc4NC(C)C | Reverse transcriptase |
| CHEMBL110625 | Cc1c(O)nc(SCC(=O)c2ccc(F)cc2)nc1Cc3cccc4ccccc34 | Reverse transcriptase |
| CHEMBL145736 | CN1C(=O)c2cccnc2N(C3CC3)c4nc(ccc14)c5cccnc5 | Reverse transcriptase |
| CHEMBL144773 | CCN1c2nc(\C=C\C(=O)OC)ccc2N(C)C(=O)c3cccnc13 | Reverse transcriptase |
| CHEMBL254237 | CCOc1ccc(OCC)c(CCNC(=S)Nc2ccc(Br)cn2)c1 | Reverse transcriptase |
| CHEMBL302861 | Fc1ccc2NC(=O)N[C@@](C#Cc3ccccc3)(c2c1F)C(F)(F)F | Reverse transcriptase |
| CHEMBL131518 | CCOC(=O)CCCC=C(c1cc(C)c(OC)c(c1)C(=O)OC)c2cc(C)c(OC)c(c2)C(=O)OC | Reverse transcriptase |
| CHEMBL49733 | CC(=C)OC(=O)N1c2cc(F)ccc2NC(=O)C1(C#CC3CC3)C(F)(F)F | Reverse transcriptase |
| CHEMBL321174 | CCOC(C#CC1CC1)(c2cc(CC)c(C)nc2O)C(F)(F)F | Reverse transcriptase |
| CHEMBL284065 | CCC1OC(\C=C\C2CC2)(c3cc(Cl)ccc3NC1=O)C(F)(F)F | Reverse transcriptase |
| CHEMBL105400 | CCc1cc(c(O)nc1C)C(OCC2CC2)(C#CC3CC3)C(F)(F)F | Reverse transcriptase |
| CHEMBL441072 | CCN1c2nc(Cl)ccc2N(C)C(=O)c3cc(COc4cccnc4)cnc13 | Reverse transcriptase |
| CHEMBL339279 | CCCN(C1CCN(CC1)C(=O)c2cc3cc(NC(=O)N4CCN(C)CC4)ccc3[nH]2)c5ncccc5NC(C)C | Reverse transcriptase |
| CHEMBL147800 | Oc1ccccc1Cc2ccnc3N(C4CC4)c5ncccc5C(=O)Nc23 | Reverse transcriptase |
| CHEMBL102056 | CCN1c2ncccc2N(C)C(=O)c3cc(CCc4ccccn4)cnc13 | Reverse transcriptase |
| CHEMBL342068 | CCN1c2nc(NC)ccc2N(C)C(=O)c3cccnc13 | Reverse transcriptase |
| CHEMBL71311 | CCN1c2ccc(C)cc2N(C)C(=O)c3cccnc13 | Reverse transcriptase |
| CHEMBL124594 | CSc1cc(NCc2oc3ccccc3n2)c(O)nc1C | Reverse transcriptase |
| CHEMBL82803 | Clc1ccc2C(=O)N3CCSC3(c4ccccc4)c2c1 | Reverse transcriptase |
| CHEMBL94725 | Fc1ccc2NC(=O)OC(C#CC3CC3)(c2c1)C(F)(F)F | Reverse transcriptase |
| CHEMBL2115336 | CCN1c2cc(C)c(C)nc2N(C)C(=O)c3cccnc13 | Reverse transcriptase |
| CHEMBL174311 | CCc1cc(OCc2oc3ccccc3n2)c(O)nc1C | Reverse transcriptase |
| CHEMBL31771 | CC1OC(C#CC2CC2)(c3c(F)c(F)ccc3NC1=O)C(F)(F)F | Reverse transcriptase |
| CHEMBL111635 | CC(C)C#CC1(OC(=O)Nc2ccc(cc12)[N+](=O)[O-])C(F)(F)F | Reverse transcriptase |
| CHEMBL54600 | Cc1cccc(c1)S(=O)(=O)c2cccc(N)c2C#N | Reverse transcriptase |
| CHEMBL293989 | Nc1cccc(c1C#N)S(=O)(=O)c2cccc(Br)c2 | Reverse transcriptase |
| CHEMBL79339 | CCN1c2ncccc2C(=O)N(C)c3ccc(nc13)c4c[nH]c5ncccc45 | Reverse transcriptase |
| CHEMBL345190 | CCCCSc1nc(O)cc(Cc2c(F)cccc2F)n1 | Reverse transcriptase |
| CHEMBL358143 | Cc1ccc(Cc2ccnc3N(C4CC4)c5ncccc5C(=O)Nc23)c(O)c1 | Reverse transcriptase |
| CHEMBL281913 | Nc1ncnc2c1ncn2C3CC(OCP(=O)(O)OP(=O)(O)OP(=O)(O)O)C=C3 | Reverse transcriptase |
| CHEMBL337050 | NC1=NC(=O)N(C=C1F)[C@H]2CS[C@@H](CO)C2 | Reverse transcriptase |
| CHEMBL398764 | CCOC(=O)c1csc(NC(=S)NCCc2ccccc2)n1 | Reverse transcriptase |
| CHEMBL35434 | C[C@H]1C(Cl)n2c(S)nc3cccc(CN1CC=C(C)C)c23 | Reverse transcriptase |
| CHEMBL70483 | CC(C)N1c2ccccc2N(C)C(=O)c3cccnc13 | Reverse transcriptase |
| CHEMBL254415 | CCCOc1cccc(CCNC(=S)Nc2nccs2)c1 | Reverse transcriptase |
| CHEMBL254839 | CC(C)c1csc(NC(=S)NCCc2ccccc2)n1 | Reverse transcriptase |
| CHEMBL41694 | Cc1ccccc1C(OCCn2c(C)ncc2[N+](=O)[O-])c3ccccc3 | Reverse transcriptase |
| CHEMBL347823 | CC(C)CSc1nc(O)cc(Cc2c(F)cccc2F)n1 | Reverse transcriptase |
| CHEMBL157028 | CSc1nc(O)c(C)c(Cc2c(F)cccc2F)n1 | Reverse transcriptase |
| CHEMBL145709 | S=C(NCCc1ccccn1)Nc2nccs2 | Reverse transcriptase |
| CHEMBL338936 | CCC1=C(C)NC(=O)C(=C1Cc2cc(C)cc(C)c2)NC=O | Reverse transcriptase |
| CHEMBL2110293 | OC1C(O)[C@@H]2O[C@H]3OC(COP(=O)O)[C@@H](O[C@H]4OC(COP(=O)O)[C@@H](O[C@H]5OC(COP(=O)O)[C@@H](O[C@H]6OC(COP(=O)O)[C@@H](O[C@H]7OC(COP(=O)O)[C@@H](O[C@H]8OC(COP(=O)O)[C@@H](O[C@@H]1OC2COP(=O)O)C(Cl)C8O)C(Cl)C7O)C(Cl)C6O)C(OP(=O)O)C5O)C(Cl)C4O)C(Cl)C3O | Reverse transcriptase |
| CHEMBL319762 | CCN1c2ncccc2N(C)C(=O)c3cc(CCc4ccccc4C)cnc13 | Reverse transcriptase |
| CHEMBL381286 | Cc1cc(C)c(c(C)c1)n2nnnc2SCC(=O)Nc3cccc(c3)[N+](=O)[O-] | Reverse transcriptase |
| CHEMBL135235 | C[C@]1(Cl)CC2=C(CCC(=O)C2)[C@H]3CC[C@@]4(C)[C@@H](CC[C@@]4(O)C#C)[C@H]13 | Reverse transcriptase |
| CHEMBL148106 | S=C(NCCc1ccccc1)Nc2nc(cs2)C#N | Reverse transcriptase |
| CHEMBL147099 | S=C(NCCc1ccccc1)Nc2nc3ccccc3s2 | Reverse transcriptase |
| CHEMBL401167 | COc1ccc(OC)c(CCNC(=S)Nc2nccs2)c1 | Reverse transcriptase |
| CHEMBL83401 | CSc1nc(O)cc(n1)C(C)c2c(F)cccc2F | Reverse transcriptase |
| CHEMBL311356 | CCN1c2nc(ccc2N(C)C(=O)c3cccnc13)c4c[nH]c5ccc(OC)cc45 | Reverse transcriptase |
| CHEMBL176637 | CCc1cc(c(O)nc1C)S(=O)(=O)Cc2oc3c(Cl)ccc(Cl)c3n2 | Reverse transcriptase |
| CHEMBL415320 | CCc1cc(NCc2oc3ccccc3n2)c(O)nc1C | Reverse transcriptase |
| CHEMBL321847 | [O-][N+](=O)c1ccc2NC(=O)OC(C#CC3CC3)(c2c1)C(F)(F)F | Reverse transcriptase |
| CHEMBL294211 | CCCN1C(=O)c2ccccc2Oc3nc(Cl)ccc13 | Reverse transcriptase |
| CHEMBL103437 | CCN1c2ncccc2N(C)C(=O)c3cc(COc4cc[n+]([O-])cc4)cnc13 | Reverse transcriptase |
| CHEMBL147210 | CCCc1[nH]c(nc1C)c2[nH]c3ccc(Cl)cc3c2S(=O)(=O)c4ccccc4 | Reverse transcriptase |
| CHEMBL318646 | CCN1c2ncccc2N(C)C(=O)c3cc(CCC#N)cnc13 | Reverse transcriptase |
| CHEMBL99644 | CCN1c2ncc(COc3ccc(O)cc3)cc2C(=O)N(C)c4ccc(Cl)nc14 | Reverse transcriptase |
| CHEMBL101293 | CCN1c2nc(Cl)ccc2N(C)C(=O)c3cc(cnc13)N(C)C(=O)c4ccccc4 | Reverse transcriptase |
| CHEMBL43308 | Cc1ccnc2N(C3CC3)c4ncccc4c5nccn5c12 | Reverse transcriptase |
| CHEMBL123504 | Cc1cc(NCN2C(=O)c3ccccc3C2=O)c(O)nc1C | Reverse transcriptase |
| CHEMBL291662 | COc1ccc2NC(=O)N[C@@](C#Cc3ccccc3)(c2c1)C(F)(F)F | Reverse transcriptase |
| CHEMBL125478 | CCN(C1CCN(CC1)C(=O)c2cc3cc(NC(=O)N4CCN(C)CC4)ccc3[nH]2)c5ncccc5NC(C)(C)C | Reverse transcriptase |
| CHEMBL342056 | CCN1c2ncccc2C(=O)N(C)c3ccc(nc13)c4occn4 | Reverse transcriptase |
| CHEMBL84705 | COC(=O)[C@H]1CSC2(N1C(=O)c3ccccc23)c4ccccc4 | Reverse transcriptase |
| CHEMBL558604 | Nc1nc(cs1)c2[nH]c3ccc(Cl)cc3c2S(=O)(=O)c4ccccc4 | Reverse transcriptase |
| CHEMBL132259 | COc1c(CCNC(=O)Nc2ccc(Cl)cn2)c(F)ccc1C(=O)C | Reverse transcriptase |
| CHEMBL125019 | CC(C)Nc1cccnc1N(C)C2CCN(CC2)C(=O)c3cc4ccccc4[nH]3.CS(=O)(=O)O | Reverse transcriptase |
| CHEMBL304826 | C[C@@H]1Cn2c(O)nc3cc(Cl)cc([C@H](C)N1CC=C(C)C)c23 | Reverse transcriptase |
| CHEMBL121820 | Cc1nc(CCCCCCC(O)c2ccccc2)n3nc(ccc13)n4ccnc4 | Reverse transcriptase |
| CHEMBL436392 | CC(C)Nc1cccnc1N(C2CC2)C3CCN(CC3)C(=O)c4cc5cc(NS(=O)(=O)C)ccc5[nH]4 | Reverse transcriptase |
| CHEMBL204735 | [O-][N+](=O)c1ccccc1NC(=O)CSc2nnnn2c3ccc(Cl)c(Cl)c3 | Reverse transcriptase |
| CHEMBL308248 | CCN1c2cc(ccc2N(C)C(=O)c3cccnc13)C(F)(F)F | Reverse transcriptase |
| CHEMBL125928 | COC(=O)c1cccc(CSc2nc(N)cc(Cl)n2)c1 | Reverse transcriptase |
| CHEMBL101670 | CCN1c2ncc(CCc3ccc(C)cc3)cc2C(=O)N(C)c4ccc(Cl)nc14 | Reverse transcriptase |
| CHEMBL319425 | CCN1c2ncc(COc3cc[n+]([O-])cc3)cc2C(=O)N(C)c4ccc(Cl)nc14 | Reverse transcriptase |
| CHEMBL144378 | CCNc1ccc2N(C)C(=O)c3cccnc3N(CC)c2n1 | Reverse transcriptase |
| CHEMBL308686 | CCCN1c2ccccc2N(C)C(=O)c3cccnc13 | Reverse transcriptase |
| CHEMBL359187 | CCN1c2nc(ccc2N(C)C(=O)c3cccnc13)c4ccccc4 | Reverse transcriptase |
| CHEMBL129835 | CCOC(=O)CCCC=C(c1cc(Cl)c(OC)c(c1)C(=O)OC)c2cc(Cl)c(OC)c(c2)C(=O)OC | Reverse transcriptase |
| CHEMBL316785 | CCc1cc(NCc2oc3ccccc3c2)c(O)nc1C | Reverse transcriptase |
| CHEMBL31285 | FC(F)(F)CC1OC(C#CC2CC2)(c3cc(Cl)ccc3NC1=O)C(F)(F)F | Reverse transcriptase |
| CHEMBL292790 | COc1ccc2NC(=O)N[C@@](C#Cc3ccccn3)(c2c1)C(F)(F)F | Reverse transcriptase |
| CHEMBL55391 | CCN1C(=O)c2cc(N)ccc2Oc3ccc(C)cc13 | Reverse transcriptase |
| CHEMBL191146 | CCCC1=C(SC2CCCCC2)c3cc(Cl)ccc3NC1=O | Reverse transcriptase |
| CHEMBL340822 | CCCN(C1CCN(CC1)C(=O)c2cc3cc(NS(=O)(=O)C)ccc3[nH]2)c4ncccc4NC(C)C | Reverse transcriptase |
| CHEMBL108647 | CC(C)c1c(O)nc(SCC(=O)c2ccc(C)cc2C)nc1Cc3cccc4ccccc34 | Reverse transcriptase |
| CHEMBL108360 | CC(C)c1c(O)nc(SCC(=O)c2ccc(C)cc2)nc1Cc3cccc4ccccc34 | Reverse transcriptase |
| CHEMBL145796 | CCN1c2nc(OC)cc(C=O)c2NC(=O)c3cccnc13 | Reverse transcriptase |
| CHEMBL70850 | CCN1c2nc(C)c(C)cc2N(C)C(=O)c3cccnc13 | Reverse transcriptase |
| CHEMBL305521 | CCN1c2cccc(C)c2N(C)C(=O)c3cccnc13 | Reverse transcriptase |
| CHEMBL125112 | CCNc1cccnc1N(CC)C2CCN(CC2)C(=O)c3cc4cc(NS(=O)(=O)N5CCN(C)CC5)ccc4[nH]3 | Reverse transcriptase |
| CHEMBL294720 | Nc1ccc(CO[C@H]2C(=O)Nc3ccc(Cl)cc3[C@@]2(C#CC4CC4)C(F)(F)F)cc1F | Reverse transcriptase |
| CHEMBL68353 | FC(F)(F)C12NC(=O)Nc3ccc(Cl)c(OC(=C1)c4ccccc4)c23 | Reverse transcriptase |
| CHEMBL297982 | CCN1c2ncccc2c3nccn3c4c(C)ccnc14 | Reverse transcriptase |
| CHEMBL2115317 | C[C@H]1O[C@@](C#CC2CC2)(c3cc(Cl)ccc3NC1=O)C(F)(F)F | Reverse transcriptase |
| CHEMBL143976 | CCN1c2nc(\C=C\C(=O)N)ccc2N(C)C(=O)c3cccnc13 | Reverse transcriptase |
| CHEMBL335193 | NC1=NC(=O)N(C=C1F)[C@@H]2CC[C@H](CO)S2 | Reverse transcriptase |
| CHEMBL100629 | CCN1c2ncccc2N(C)C(=O)c3cc(\C=C/c4ccccc4)cnc13 | Reverse transcriptase |
| CHEMBL130177 | COC(=O)c1cccnc1N(C)C2CCN(CC2)C(=O)c3cc4cc(NS(=O)(=O)C)ccc4[nH]3 | Reverse transcriptase |
| CHEMBL56383 | CN1C(=O)c2cc(N)cnc2Oc3cc(C)ccc13 | Reverse transcriptase |
| CHEMBL607723 | CCCCCCCCCC(=O)NC1=NN(C)c2ncnc3c2c1cn3C4O[C@H](CO)[C@@H](O)[C@H]4O | Reverse transcriptase |
| CHEMBL329653 | CCC1(Oc2ccccc2n3cccc3C1=O)c4ccccc4 | Reverse transcriptase |
| CHEMBL295978 | CCN1c2ncccc2c3nccn3c4ccc(OC)nc14 | Reverse transcriptase |
| CHEMBL57697 | CCCN1C(=O)c2cc(N)ccc2Oc3cccc(C)c13 | Reverse transcriptase |
| CHEMBL124726 | CCc1cc(NCc2oc3c(CC)cccc3n2)c(O)nc1C | Reverse transcriptase |
| CHEMBL328168 | CCN1c2nc(Cl)ccc2N(C)C(=O)c3cc(NCc4ccccc4)cnc13 | Reverse transcriptase |
| CHEMBL340453 | CCNc1cccnc1N(CC)C2CCN(CC2)C(=O)c3cc4cc(NS(=O)(=O)CC)ccc4[nH]3 | Reverse transcriptase |
| CHEMBL108581 | CCc1c(O)nc(SCC(=O)c2ccc(Cl)cc2)nc1Cc3cccc4ccccc34 | Reverse transcriptase |
| CHEMBL299073 | Fc1ccc2NC(=O)C(C#CC3CC3)(N(CC4CC4)c2c1)C(F)(F)F | Reverse transcriptase |
| CHEMBL314061 | CCc1cc(NCc2ccccc2OC)c(O)nc1C | Reverse transcriptase |
| CHEMBL82481 | Clc1ccc2NC(=O)NC(C#Cc3cnccn3)(C4CC4)c2c1 | Reverse transcriptase |
| CHEMBL341512 | CN(C)c1ccc2c(C)nc(CCCCCCC(=O)c3ccccc3)n2n1 | Reverse transcriptase |
| CHEMBL341667 | O=C1Nc2c(COCc3ccccc3)ccnc2N(C4CC4)c5ncccc15 | Reverse transcriptase |
| CHEMBL612013 | CCN(CC)CC.NC1=NC(=O)N(C=C1F)[C@@H]2C[C@H](COP(=O)(O)OP(=O)(O)OP(=O)(O)O)C=C2 | Reverse transcriptase |
| CHEMBL132883 | CCCc1cccnc1N(CC)C2CCN(CC2)C(=O)c3cc4cc(NS(=O)(=O)C)ccc4[nH]3 | Reverse transcriptase |
| CHEMBL150424 | CC(C)OC(=O)c1cccn1S(=O)(=O)c2cc(Cl)ccc2N | Reverse transcriptase |
| CHEMBL109184 | CCC#CC1(OC(=O)Nc2ccc(cc12)[N+](=O)[O-])C(F)(F)F | Reverse transcriptase |
| CHEMBL291400 | FC(F)(F)[C@]1(NC(=O)Nc2ccc(Cl)cc12)C#Cc3ccccc3 | Reverse transcriptase |
| CHEMBL2115325 | CCN1c2cccnc2N(C)C(=O)c3cc(N)cnc13 | Reverse transcriptase |
| CHEMBL317080 | CCN1c2ncc(COc3ccc(F)cc3)cc2C(=O)N(C)c4ccc(Cl)nc14 | Reverse transcriptase |
| CHEMBL339354 | Nc1cc(nc(SCc2ccc3ccccc3c2)n1)C(F)(F)F | Reverse transcriptase |
| CHEMBL56628 | CN1C(=O)c2cc(ccc2Oc3cc(C)ccc13)C#N | Reverse transcriptase |
| CHEMBL85850 | O=C1N2CCS[C@]2(c3ccccc3)c4ccccc14 | Reverse transcriptase |
| CHEMBL430623 | O=C1N2CCSC2(c3ccccc3)c4ccccc14 | Reverse transcriptase |
| CHEMBL416853 | CC(C)C#C[C@@]1(NC(=O)Nc2ccc(Cl)cc12)C(F)(F)F | Reverse transcriptase |
| CHEMBL129094 | NC1=NC(=O)N(C=C1)[C@@H]2CC[C@H](CO)S2 | Reverse transcriptase |
| CHEMBL144561 | CCN1c2nc(ccc2N(C)C(=O)c3cccnc13)c4ccn[nH]4 | Reverse transcriptase |
| CHEMBL82630 | Clc1cccc2C(=O)N3CCSC3(c4ccccc4)c12 | Reverse transcriptase |
| CHEMBL2115328 | CN1C(=O)c2cccnc2N(C3CC3)c4ncc(Cl)cc14 | Reverse transcriptase |
| CHEMBL144872 | Cc1ccccc1OCc2ccnc3N(C4CC4)c5ncccc5C(=O)Nc23 | Reverse transcriptase |
| CHEMBL101998 | CCN1c2ncc(CCc3ccncc3)cc2C(=O)N(C)c4ccc(nc14)C#N | Reverse transcriptase |
| CHEMBL70255 | CCN1c2cc(cc(Cl)c2N(C)C(=O)c3cccnc13)C(F)(F)F | Reverse transcriptase |
| CHEMBL3038091 | CC[C@H](C)C[C@@H]1N(C)C(=O)CN(C)C(=O)[C@H](CC)NC(=O)[C@H]([C@H](O)[C@H](C)C\C=C\C)N(C)C(=O)[C@H](C(C)C)N(C)C(=O)[C@H](CC(C)C)N(C)C(=O)[C@H](CC(C)C)N(C)C(=O)[C@@H](C)NC(=O)[C@H](C)NC(=O)[C@H](CC(C)C)N(C)C(=O)[C@@H](NC1=O)C(C)C | Reverse transcriptase |
| CHEMBL294840 | COC(=O)CCCC=C(c1cc(Cl)c(OC)c(c1)C(=O)OC)c2cc(Cl)c(OC)c(c2)C(=O)OC | Reverse transcriptase |
| CHEMBL339406 | CC(C)Nc1cccnc1N(C)C2CCN(CC2)C(=O)c3cc4ccc(OCCOCCOCCO)cc4[nH]3 | Reverse transcriptase |
| CHEMBL157137 | CCC(C)Sc1nc(O)cc(Cc2cccc(c2)[N+](=O)[O-])n1 | Reverse transcriptase |
| CHEMBL100844 | CCN1c2ncccc2N(C)C(=O)c3cc(CCCO)cnc13 | Reverse transcriptase |
| CHEMBL124149 | CC(=O)c1cc(NCc2oc3c(Cl)ccc(Cl)c3n2)c(O)nc1C | Reverse transcriptase |
| CHEMBL37067 | CC1=C(Sc2ccccc2)N(COCc3ccccc3)C(=O)NC1=O | Reverse transcriptase |
| CHEMBL124557 | CCc1cc(NCc2ccc3ccccc3c2)c(S)nc1C | Reverse transcriptase |
| CHEMBL39021 | Cc1cccc(c1)C(OCCn2c(C)ncc2[N+](=O)[O-])c3ccccc3 | Reverse transcriptase |
| CHEMBL538904 | NC1=Nc2c(ncn2C3CC(OCP(=O)(O)OP(=O)(O)OP(=O)(O)O)C=C3)C(=O)N1 | Reverse transcriptase |
| CHEMBL301055 | Nc1cccc(c1C#N)S(=O)(=O)c2cc(Cl)ccc2Cl | Reverse transcriptase |
| CHEMBL107200 | COC(=O)CCCC=C(c1cc(Br)c(OC)c(c1)C(=O)OC)c2cc(Br)c(OC)c(c2)C(=O)OC | Reverse transcriptase |
| CHEMBL40632 | Cc1ncc([N+](=O)[O-])n1CCOC(c2ccccc2)c3cccc(c3)[N+](=O)[O-] | Reverse transcriptase |
| CHEMBL155689 | CCC(C)Sc1nc(O)cc(Cc2ccccc2F)n1 | Reverse transcriptase |
| CHEMBL357411 | CCN1c2nc(ccc2N(C)C(=O)c3cccnc13)N4CCCCC4 | Reverse transcriptase |
| CHEMBL347403 | CCC(C)Sc1nc(O)cc(Cc2ccccc2[N+](=O)[O-])n1 | Reverse transcriptase |
| CHEMBL110048 | CCCC#CC1(OC(=O)Nc2ccc(cc12)[N+](=O)[O-])C(F)(F)F | Reverse transcriptase |
| CHEMBL174953 | Cc1nc(O)c(CCc2oc3ccccc3n2)cc1C#N | Reverse transcriptase |
| CHEMBL144866 | CCN1c2ncccc2C(=O)N(C)c3ccc(nc13)n4cccn4 | Reverse transcriptase |
| CHEMBL46008 | CC(=C)OC(=O)N1c2cc(Cl)ccc2NC(=O)C1(C#CC3CC3)C(F)(F)F | Reverse transcriptase |
| CHEMBL320702 | CCc1c(O)nc(SCC(=O)c2ccc(C)cc2)nc1Cc3cccc4ccccc34 | Reverse transcriptase |
| CHEMBL133250 | COc1ccc2[nH]cc(CC\N=C(/S)\Nc3ccc(Br)cn3)c2c1 | Reverse transcriptase |
| CHEMBL316773 | CC(C)c1c(O)nc(SCC(=O)c2ccc(Cl)cc2)nc1Cc3cccc4ccccc34 | Reverse transcriptase |
| CHEMBL418104 | CN1C(=O)c2cc(N)cnc2Oc3cc(C)c(C)cc13 | Reverse transcriptase |
| CHEMBL49531 | FC(F)(F)C1(C#CC2CC2)N(CC3CC3)c4cc(Cl)ccc4NC1=O | Reverse transcriptase |
| CHEMBL100951 | CCN1c2ncc(COc3cccc4[nH]ccc34)cc2C(=O)N(C)c5ccc(Cl)nc15 | Reverse transcriptase |
| CHEMBL122460 | CCNc1cccnc1N(CC)C2CCN(CC2)C(=O)c3cc4cc(NS(=O)(=O)C(C)C)ccc4[nH]3 | Reverse transcriptase |
| CHEMBL101834 | CCN1c2nc(Cl)ccc2N(C)C(=O)c3cc(CCc4ccc(NS(=O)(=O)C)cc4)cnc13 | Reverse transcriptase |
| CHEMBL342342 | CCN1c2nc(cc(C=O)c2NC(=O)c3cccnc13)c4cccc(OC)c4 | Reverse transcriptase |
| CHEMBL123553 | CCc1cc(NCc2oc3c(Cl)cccc3c2)c(O)nc1C | Reverse transcriptase |
| CHEMBL125510 | CCc1cc(NCc2cnc3ccccc3c2)c(O)nc1C | Reverse transcriptase |
| CHEMBL48286 | FC(F)(F)C1(C#CC2CC2)N(CCC3CC3)c4ccccc4NC1=O | Reverse transcriptase |
| CHEMBL125205 | Clc1ccc2cnc(CCCCCCC(=O)c3ccccc3)n2n1 | Reverse transcriptase |
| CHEMBL357250 | CCN1c2nc(O)cc(Cc3ccccc3)c2NC(=O)c4cccnc14 | Reverse transcriptase |
| CHEMBL56567 | CC(C)N1C(=O)c2ccccc2Oc3ccccc13 | Reverse transcriptase |
| CHEMBL293785 | CCN1C(=O)c2cccnc2Oc3cccc(c13)[N+](=O)[O-] | Reverse transcriptase |
| CHEMBL431274 | CCc1cc(NCc2nc3ccccc3s2)c(O)nc1C | Reverse transcriptase |
| CHEMBL611245 | CCCCCC(=O)NC1=NN(C)c2ncnc3c2c1cn3C4O[C@H](CO)[C@@H](O)[C@H]4O | Reverse transcriptase |
| CHEMBL306032 | CCN1c2ccccc2N(C)C(=O)c3cccnc13 | Reverse transcriptase |
| CHEMBL85142 | CCOc1ccccc1CNc2cc(CC)c(C)nc2O | Reverse transcriptase |
| CHEMBL254206 | COc1ccc(CCNC(=S)Nc2nccs2)cc1 | Reverse transcriptase |
| CHEMBL124610 | CCC(C)(C)Nc1cccnc1N(C)C2CCN(CC2)C(=O)c3cc4cc(NS(=O)(=O)C)ccc4[nH]3 | Reverse transcriptase |
| CHEMBL289038 | CCN1c2cnccc2N(C)C(=O)c3cccnc13 | Reverse transcriptase |
| CHEMBL399069 | CCCc1csc(NC(=S)NCCc2ccccc2)n1 | Reverse transcriptase |
| CHEMBL132049 | NC1=NC(=O)N(C=C1)[C@H]2CS[C@@H](CO)C2 | Reverse transcriptase |
| CHEMBL264184 | Clc1ccc2[nH]c(C3=NCCN3)c(c2c1)S(=O)(=O)c4ccccc4 | Reverse transcriptase |
| CHEMBL340869 | CCOC(=O)CCCC=C(c1cc(Br)c(OC)c(c1)C(=O)OC)c2cc(Br)c(OC)c(c2)C(=O)OC | Reverse transcriptase |
| CHEMBL309574 | CCN1c2ncccc2N(C(=O)C)C(=O)c3cccnc13 | Reverse transcriptase |
| CHEMBL127283 | CCNc1cccnc1N(CC)C2CCN(CC2)C(=O)c3cc4cc(NS(=O)(=O)C)ccc4[nH]3 | Reverse transcriptase |
| CHEMBL121827 | CC(C)Nc1ccc(Cl)nc1N(C)C2CCN(CC2)C(=O)c3cc4cc(NS(=O)(=O)C)ccc4[nH]3 | Reverse transcriptase |
| CHEMBL107606 | COc1ccc(cc1)C(=O)CSc2nc(O)c(C)c(Cc3cccc4ccccc34)n2 | Reverse transcriptase |
| CHEMBL95426 | CCc1cc(CCc2nc3ccccc3s2)c(O)nc1C | Reverse transcriptase |
| CHEMBL56451 | CCCN1C(=O)c2ccccc2Oc3ccccc13 | Reverse transcriptase |
| CHEMBL58542 | CCCCCC=C(c1cc(Br)c(OC)c(c1)C(=O)OC)c2cc(Br)c(OC)c(c2)C(=O)OC | Reverse transcriptase |
| CHEMBL73845 | CCN1c2ccccc2NC(=O)c3cccnc13 | Reverse transcriptase |
| CHEMBL340556 | CCNc1cccnc1N(C(C)C)C2CCN(CC2)C(=O)c3cc4cc(NS(=O)(=O)C)ccc4[nH]3 | Reverse transcriptase |
| CHEMBL125084 | Cc1nc(CCCCCCC(O)c2ccccc2)n3nc(Cl)ccc13 | Reverse transcriptase |
| CHEMBL358564 | CCN1c2nc(ccc2N(C)C(=O)c3cccnc13)c4cncs4 | Reverse transcriptase |
| CHEMBL611745 | CCN(CC)CC.NC1=NC(=O)N(C=C1)C2CC(COP(=O)(O)OP(=O)(O)OP(=O)(O)O)C=C2 | Reverse transcriptase |
| CHEMBL332675 | CCN(C1CCN(CC1)C(=O)c2cc3cc(NS(=O)(=O)N4CCN(C)CC4)ccc3[nH]2)c5ncccc5NC(C)C | Reverse transcriptase |
| CHEMBL187438 | CCCc1c(O)nc2ccc(Cl)cc2c1[S+]([O-])C3CCCCC3 | Reverse transcriptase |
| CHEMBL70312 | CCN1C(=O)c2cccnc2N(CC)c3ccccc13 | Reverse transcriptase |
| CHEMBL173635 | CCc1cc(CCc2oc(cn2)c3ccccc3)c(O)nc1C | Reverse transcriptase |
| CHEMBL345006 | CCN1c2nc(N\C=C\C)ccc2N(C)C(=O)c3cccnc13 | Reverse transcriptase |
| CHEMBL315990 | Cc1ccc(cc1C)C23SCCN2C(=O)c4ccccc34 | Reverse transcriptase |
| CHEMBL285510 | CCC1OC(C#CC2CC2)(c3cc(Cl)ccc3NC1=O)C(F)(F)F | Reverse transcriptase |
| CHEMBL55997 | CCCCOc1cc(C)cc(c1)S(=O)(=O)c2cccc(N)c2C#N | Reverse transcriptase |
| CHEMBL291532 | Nc1cccc(c1C#N)S(=O)(=O)c2cccc(Cl)c2 | Reverse transcriptase |
| CHEMBL86015 | CC(c1cc(O)nc(SC2CCCC2)n1)c3cccc4ccccc34 | Reverse transcriptase |
| CHEMBL155483 | Oc1cc(Cc2c(Cl)cccc2Cl)nc(SC3CCCC3)n1 | Reverse transcriptase |
| CHEMBL155864 | CCC(C)Sc1nc(O)c(C)c(Cc2ccccc2Cl)n1 | Reverse transcriptase |
| CHEMBL341931 | CCc1c(C)nc(O)cc1Sc2cc(C)cc(C)c2 | Reverse transcriptase |
| CHEMBL43293 | Cc1ncc([N+](=O)[O-])n1CCOC(c2ccccc2)c3ccccc3F | Reverse transcriptase |
| CHEMBL93822 | Brc1ccc(NC(=S)NCCC2=CCCCC2)nc1 | Reverse transcriptase |
| CHEMBL55258 | CCCN1C(=O)c2ccccc2Sc3ccccc13 | Reverse transcriptase |
| CHEMBL254416 | CC(C)Oc1cccc(CCNC(=S)Nc2nccs2)c1 | Reverse transcriptase |
| CHEMBL299714 | CCOC(=O)c1cccn1S(=O)(=O)c2cc(Cl)ccc2N | Reverse transcriptase |
| CHEMBL356611 | CCCOC(=O)c1cccn1S(=O)(=O)c2cc(Cl)ccc2N | Reverse transcriptase |
| CHEMBL611744 | CCN(CC)CC.NC1=NC(=O)N(C=C1F)C2CC(COP(=O)(O)OP(=O)(O)OP(=O)(O)O)C=C2 | Reverse transcriptase |
| CHEMBL145295 | CCN1c2nc(ccc2N(C)C(=O)c3cccnc13)N4CCOCC4 | Reverse transcriptase |
| CHEMBL351121 | CCC(C)Sc1nc(O)c(C)c(Cc2cccc(c2)[N+](=O)[O-])n1 | Reverse transcriptase |
| CHEMBL91942 | CCOc1ccc(F)c(CC\N=C(/N)\Nc2ccc(Br)cn2)c1Cl | Reverse transcriptase |
| CHEMBL71927 | CCN1c2cc(ccc2N(C)C(=O)c3cccnc13)[N+](=O)[O-] | Reverse transcriptase |
| CHEMBL170942 | COc1cccc(CC\N=C(/S)\Nc2ccc(Br)cn2)c1 | Reverse transcriptase |
| CHEMBL83641 | CCc1cc(CCc2cc(CC)c(C)nc2O)c(O)nc1C | Reverse transcriptase |
| CHEMBL349806 | Oc1cc(Cc2c(Cl)cccc2Cl)nc(SC3CCCCC3)n1 | Reverse transcriptase |
| CHEMBL60513 | COc1ccc2NC(=O)N[C@@](C#CC(C)C)(c2c1)C(F)(F)F | Reverse transcriptase |
| CHEMBL300721 | CC(C)CN1c2cc(Cl)ccc2NC(=O)C1(C#CC3CC3)C(F)(F)F | Reverse transcriptase |
| CHEMBL321980 | CC(C)c1c(O)nc(SCC(=O)C)nc1Cc2cccc3ccccc23 | Reverse transcriptase |
| CHEMBL311349 | CCN1c2ncccc2C(=O)Nc3cc(C)c(C)nc13 | Reverse transcriptase |
| CHEMBL336318 | CCN(C1CCN(CC1)C(=O)c2cc3cc(NS(=O)(=O)C)ccc3[nH]2)c4ncccc4CC(C)C | Reverse transcriptase |
| CHEMBL348086 | CCC(C)Sc1nc(O)c(C)c(Cc2ccccc2F)n1 | Reverse transcriptase |
| CHEMBL146828 | O=C1Nc2c(COc3ccc(cc3)C#N)ccnc2N(C4CC4)c5ncccc15 | Reverse transcriptase |
| CHEMBL57216 | Nc1ccc(CO[C@H]2C(=O)Nc3ccc(Cl)cc3[C@@]2(C#CC4CC4)C(F)(F)F)cc1 | Reverse transcriptase |
| CHEMBL434122 | O=C1Nc2c(COCCc3ccccc3)ccnc2N(C4CC4)c5ncccc15 | Reverse transcriptase |
| CHEMBL143250 | CCN1c2nc(O)cc(CO)c2NC(=O)c3cccnc13 | Reverse transcriptase |
| CHEMBL70930 | CN1C(=O)c2cccnc2N(C(=O)C)c3cc(C)c(C)cc13 | Reverse transcriptase |
| CHEMBL283614 | CC(C)C#CC1(OCC(=O)Nc2ccc(Cl)cc12)C(F)(F)F | Reverse transcriptase |
| CHEMBL42132 | CCN1c2ncccc2c3ncc(C)n3c4cccnc14 | Reverse transcriptase |
| CHEMBL321765 | CCN1c2ncccc2N(C)C(=O)c3cc(CCO)cnc13 | Reverse transcriptase |
| CHEMBL124453 | CCSc1cc(NCc2oc3ccccc3n2)c(O)nc1C | Reverse transcriptase |
| CHEMBL424046 | O=C1Nc2cccnc2N(C3CCC3)c4ncccc14 | Reverse transcriptase |
| CHEMBL32290 | FC(F)(F)C1(OCC(=O)Nc2ccc(Cl)cc12)C#CC3CC3 | Reverse transcriptase |
| CHEMBL59215 | FC(F)(F)[C@@]1(C#CC2CC2)[C@@H](OCc3cccnc3)C(=O)Nc4ccc(Cl)cc14 | Reverse transcriptase |
| CHEMBL291024 | CCN1c2ncccc2NC(=O)c3cccnc13 | Reverse transcriptase |
| CHEMBL123778 | CCc1cc(NCc2oc3cccc(O)c3n2)c(O)nc1C | Reverse transcriptase |
| CHEMBL58970 | FC(F)(F)[C@@]1(C#CC2CC2)[C@@H](OCc3ccccc3)C(=O)Nc4ccc(Cl)cc14 | Reverse transcriptase |
| CHEMBL318730 | CCc1cc(NCc2ccc3ccccc3c2)c(O)nc1C | Reverse transcriptase |
| CHEMBL177898 | CCc1cc(CCc2oc3cc(F)ccc3n2)c(O)nc1C | Reverse transcriptase |
| CHEMBL94526 | Fc1cc2NC(=O)OC(C#CC3CC3)(c2c(F)c1F)C(F)(F)F | Reverse transcriptase |
| CHEMBL151896 | COC(=O)c1cccn1S(=O)(=O)c2cc(Cl)ccc2N | Reverse transcriptase |
| CHEMBL289816 | O=C1Nc2cccnc2N(C3CC3)c4ncccc14 | Reverse transcriptase |
| CHEMBL144136 | CC(C\N=C(/S)\Nc1nccs1)c2ccccc2 | Reverse transcriptase |
| CHEMBL290957 | CCCN1c2ncccc2N(C)C(=O)c3cccnc13 | Reverse transcriptase |
| CHEMBL79696 | Clc1ccc2NC(=O)NC(C#Cc3ccncc3)(C4CC4)c2c1 | Reverse transcriptase |
| CHEMBL160 | CC[C@@H]1NC(=O)[C@H]([C@H](O)[C@H](C)C\C=C\C)N(C)C(=O)[C@H](C(C)C)N(C)C(=O)[C@H](CC(C)C)N(C)C(=O)[C@H](CC(C)C)N(C)C(=O)[C@@H](C)NC(=O)[C@H](C)NC(=O)[C@H](CC(C)C)N(C)C(=O)[C@@H](NC(=O)[C@H](CC(C)C)N(C)C(=O)CN(C)C1=O)C(C)C | Reverse transcriptase |
| CHEMBL145158 | Oc1ccccc1OCc2ccnc3N(C4CC4)c5ncccc5C(=O)Nc23 | Reverse transcriptase |
| CHEMBL127456 | CCN(C1CCN(CC1)C(=O)c2cc3cc(NS(=O)(=O)C)ccc3[nH]2)c4nc(F)ccc4NC(C)C | Reverse transcriptase |
| CHEMBL301443 | C=CCN1C(=O)c2ccccc2Oc3ccccc13 | Reverse transcriptase |
| CHEMBL146169 | CCN1c2ncccc2C(=O)N(C)c3ccc(O)nc13 | Reverse transcriptase |
| CHEMBL144701 | COC(=O)CCCCC=C(c1cc(Cl)c(OC)c(c1)C(=O)OC)c2cc(Cl)c(OC)c(c2)C(=O)OC | Reverse transcriptase |
| CHEMBL125397 | CCc1cc(NCc2oc3ccc(F)cc3n2)c(O)nc1C | Reverse transcriptase |
| CHEMBL323413 | CNc1ccc2NC(=O)OC(C#CC(C)C)(c2c1)C(F)(F)F | Reverse transcriptase |
| CHEMBL316083 | FC(F)(F)C1(OC(=O)Nc2ccccc12)C#CC3CC3 | Reverse transcriptase |
| CHEMBL308644 | C[C@@H]1Cn2c(S)nc3cc(Cl)cc([C@H](C)N1CC=C(C)C)c23 | Reverse transcriptase |
| CHEMBL125324 | CCCN(C1CCN(CC1)C(=O)c2cc3cc(NS(=O)(=O)C)ccc3[nH]2)c4ncccc4NC(C)(C)C | Reverse transcriptase |
| CHEMBL145893 | CCN1c2nc(cc(COc3ccccc3)c2NC(=O)c4cccnc14)c5cccc(OC)c5 | Reverse transcriptase |
| CHEMBL131761 | COC(=O)c1cc(cc(Br)c1OC)C(=CCCN2CCOC2=O)c3cc(Br)c(OC)c(c3)C(=O)OC | Reverse transcriptase |
| CHEMBL2092835 | NC1=NC(=O)N(C=C1)[C@@H]2C[C@@H](F)[C@H](COP(=O)(O)OP(=O)(O)OP(=O)(O)O)O2 | Reverse transcriptase |
| CHEMBL37056 | CC(C)Nc1cccnc1N2CCN(CC2)C(=O)c3cc4ccc(NS(=O)(=O)C)cc4[nH]3 | Reverse transcriptase |
| CHEMBL305010 | CCN1c2ccccc2C(=O)N(C)c3cccnc13 | Reverse transcriptase |
| CHEMBL124338 | CC(C)Nc1cccnc1N(C)C2CCN(CC2)C(=O)c3cc4cc(NS(=O)(=O)C)ccc4[nH]3 | Reverse transcriptase |
| CHEMBL611242 | CCCC(=O)NC1=NN(C)c2ncnc3c2c1cn3C4O[C@H](CO)[C@@H](O)[C@H]4O | Reverse transcriptase |
| CHEMBL156083 | CCCCSc1nc(O)cc(Cc2c(Cl)cccc2Cl)n1 | Reverse transcriptase |
| CHEMBL54318 | Cc1cc(C)cc(c1)[S+]([O-])c2cccc(N)c2C#N | Reverse transcriptase |
| CHEMBL53554 | Fc1cccc(F)c1C2SCc3nc4ccccc4n23 | Reverse transcriptase |
| CHEMBL121835 | CCc1cc(NCc2cc3ccccc3s2)c(O)nc1C | Reverse transcriptase |
| CHEMBL387278 | CCCN1C(=O)N(C=C(C)C1=O)[C@@H]2O[C@H](CO[Si](C)(C)C(C)(C)C)[C@@]3(OS(=O)(=O)C=C3N)[C@H]2O[Si](C)(C)C(C)(C)C | Reverse transcriptase |
| CHEMBL112949 | Cc1c(O)nc(SC2CCCC2)nc1Cc3ccccc3 | Reverse transcriptase |
| CHEMBL213419 | CC1=CN([C@@H]2O[C@H](CO[Si](C)(C)C(C)(C)C)[C@@]3(OS(=O)(=O)C=C3N)[C@H]2O[Si](C)(C)C(C)(C)C)C(=O)N(\C=C\CO)C1=O | Reverse transcriptase |
| CHEMBL91891 | CCC1(Oc2ccccc2n3cccc3C1=O)c4ccc5ccccc5c4 | Reverse transcriptase |
| CHEMBL416617 | Cc1ncc([N+](=O)[O-])n1CCOC(c2ccccc2)c3cccc(Cl)c3 | Reverse transcriptase |
| CHEMBL288629 | Cc1ncc([N+](=O)[O-])n1CCOC(c2ccccc2)c3cccc(F)c3 | Reverse transcriptase |
| CHEMBL109389 | CCC(C)Sc1nc(O)c(C)c(Cc2ccccc2)n1 | Reverse transcriptase |
| CHEMBL199090 | CC(C)CC(=O)c1c(O)c(CC(=C(C)C)O)c2OC(=O)C=C(c3ccccc3)c2c1O | Reverse transcriptase |
| CHEMBL330537 | Clc1ccc(NC(=S)NCCC2=CCCCC2)nc1 | Reverse transcriptase |
| CHEMBL380643 | COC(=O)C1=C([C@H]2c3ccccc3S[C@@]12C(=O)OC)N4CCC[C@H]4C | Reverse transcriptase |
| CHEMBL297103 | COC(=O)N1c2ccccc2NC(=O)C1(C#CC3CC3)C(F)(F)F | Reverse transcriptase |
| CHEMBL82904 | CCc1ccc(CNc2cc(CC)c(C)nc2O)c(OC)c1 | Reverse transcriptase |
| CHEMBL133469 | CC(C)Cc1cccnc1N(C)C2CCN(CC2)C(=O)c3cc4cc(NS(=O)(=O)C)ccc4[nH]3 | Reverse transcriptase |
| CHEMBL103716 | CCN1c2nc(ccc2N(C)C(=O)c3cc(CCc4ccccc4)cnc13)C5=CCC=N5 | Reverse transcriptase |
| CHEMBL340193 | CN(C1CCN(CC1)C(=O)c2cc3cc(NS(=O)(=O)C)ccc3[nH]2)c4ncccc4NCC5CC5 | Reverse transcriptase |
| CHEMBL267747 | CCc1c(C)nn(c2ccccc2)c1c3ccccc3 | Reverse transcriptase |
| CHEMBL55862 | Cc1cc(Cl)cc(c1)[S+]([O-])c2cccc(N)c2C#N | Reverse transcriptase |
| CHEMBL123639 | CCc1cc(NCc2ccc3ccccc3n2)c(O)nc1C | Reverse transcriptase |
| CHEMBL330990 | CN(C1CCN(CC1)C(=O)c2cc3cc(NS(=O)(=O)C)ccc3[nH]2)c4nc(F)ccc4NC(C)(C)C | Reverse transcriptase |
| CHEMBL313695 | CCc1cc(NCc2ccccc2[N+](=O)[O-])c(O)nc1C | Reverse transcriptase |
| CHEMBL198050 | COC(=O)CCC\C=C(/c1cccc(c1)C#N)\c2cc(C)c(OC)c(c2)C(=O)OC | Reverse transcriptase |
| CHEMBL71766 | CCN1c2ncccc2N(C)C(=O)c3cc(Cl)cnc13 | Reverse transcriptase |
| CHEMBL100968 | CCN1c2ncccc2N(C)C(=O)c3cc(CCc4cncnc4)cnc13 | Reverse transcriptase |
| CHEMBL106661 | Cc1c(O)nc(SCC(=O)c2ccc(Cl)cc2)nc1Cc3cccc4ccccc34 | Reverse transcriptase |
| CHEMBL410771 | FC(F)(F)c1csc(NC(=S)NCCc2ccccc2)n1 | Reverse transcriptase |
| CHEMBL2115335 | CCN1c2cc(Br)cnc2N(C)C(=O)c3cccnc13 | Reverse transcriptase |
| CHEMBL127427 | Cc1cn(cn1)c2ccc3c(C)nc(CCCCCCC(=O)c4ccccc4)n3n2 | Reverse transcriptase |
| CHEMBL80118 | CCN1c2nc(ccc2N(C)C(=O)c3cccnc13)c4c[nH]c5ccc(F)cc45 | Reverse transcriptase |
| CHEMBL340248 | Cc1nc(CCCCCCC(=O)c2ccccc2)n3ncccc13 | Reverse transcriptase |
| CHEMBL264587 | CNC(=O)CCCCN1C(=O)N(C=C(C)C1=O)[C@@H]2O[C@H](CO[Si](C)(C)C(C)(C)C)[C@@]3(OS(=O)(=O)C=C3N)[C@H]2O[Si](C)(C)C(C)(C)C | Reverse transcriptase |
| CHEMBL299710 | CCN1C(=O)c2ccccc2Oc3ccccc13 | Reverse transcriptase |
| CHEMBL312340 | CCN1c2ccc(C)cc2C(=O)N(C)c3cccnc13 | Reverse transcriptase |
| CHEMBL356313 | Cc1ccc(OCc2ccnc3N(C4CC4)c5ncccc5C(=O)Nc23)cc1 | Reverse transcriptase |
| CHEMBL314633 | Clc1cc(Cl)cc(c1)C23SCCN2C(=O)c4ccccc34 | Reverse transcriptase |
| CHEMBL71023 | CC(C)N1c2ncccc2NC(=O)c3cccnc13 | Reverse transcriptase |
| CHEMBL421246 | CC(C)Nc1cccnc1N(C2CC2)C3CCN(CC3)C(=O)c4cc5ccccc5[nH]4 | Reverse transcriptase |
| CHEMBL102735 | CCN1c2ncc(CN(C)c3ccccc3)cc2C(=O)N(C)c4ccc(Cl)nc14 | Reverse transcriptase |
| CHEMBL32038 | CC(C)C1OC(C#CC2CC2)(c3cc(Cl)ccc3NC1=O)C(F)(F)F | Reverse transcriptase |
| CHEMBL254836 | CCc1csc(NC(=S)NCCc2ccccc2)n1 | Reverse transcriptase |
| CHEMBL298405 | CN1C(=O)c2ccccc2Oc3cc(C)ccc13 | Reverse transcriptase |
| CHEMBL483492 | CC1=CN([C@@H]2C[C@@H](F)[C@H](COP(=O)(O)OP(=O)(O)OP(=O)(O)O)O2)C(=O)N=C1N | Reverse transcriptase |
| CHEMBL357409 | CCOC(=O)c1c(O)nc(C)c(CC)c1Sc2cc(C)cc(C)c2 | Reverse transcriptase |
| CHEMBL415716 | CCc1c(C)nc(O)c(NCc2oc3ccccc3n2)c1C | Reverse transcriptase |
| CHEMBL288535 | Cc1ncc([N+](=O)[O-])n1CCOC(c2ccccc2)c3ccccc3Cl | Reverse transcriptase |
| CHEMBL295585 | Fc1ccccc1CC\N=C(/S)\Nc2ccc(Br)cn2 | Reverse transcriptase |
| CHEMBL151716 | Oc1ccc(CCOC(=O)c2cc(O)c(O)c(O)c2)cc1O | Reverse transcriptase |
| CHEMBL69952 | Brc1ccc(NC(=S)NCCN2CCCCC2)nc1 | Reverse transcriptase |
| CHEMBL55199 | COc1cccc(c1)S(=O)(=O)c2cccc(N)c2C#N | Reverse transcriptase |
| CHEMBL148698 | S=C(NCc1occc1)Nc2nccs2 | Reverse transcriptase |
| CHEMBL102957 | Clc1ccccc1CCNC(=S)Nc2nccs2 | Reverse transcriptase |
| CHEMBL156380 | CC(C)CSc1nc(O)cc(Cc2c(Cl)cccc2Cl)n1 | Reverse transcriptase |
| CHEMBL191655 | O=C1N(CCOC(=S)Nc2ccccc2)C(=O)c3ccccc13 | Reverse transcriptase |
| CHEMBL149161 | S=C(NCC12CC3CC(CC(C3)C1)C2)Nc4nccs4 | Reverse transcriptase |
| CHEMBL346023 | CCC(C)Sc1nc(O)cc(Cc2cccc(F)c2)n1 | Reverse transcriptase |
| CHEMBL340145 | Nc1cc(Cl)nc(SCC2=CCCc3ccccc23)n1 | Reverse transcriptase |
| CHEMBL46209 | FC(F)(F)C1(C#CC2CC2)N(CC=C)c3ccccc3NC1=O | Reverse transcriptase |
| CHEMBL322813 | CNc1ccc2NC(=O)OC(C#CC3CC3)(c2c1)C(F)(F)F | Reverse transcriptase |
| CHEMBL413751 | CCc1cc(c(O)nc1C)C(OCc2c(F)cccc2F)(C#CC3CC3)C(F)(F)F | Reverse transcriptase |
| CHEMBL1160976 | CC1=CN(C2CC(N=[N+]=[N-])C(COP(=O)(O)OP(=O)(O)C(F)(F)P(=O)(O)O)O2)C(=O)NC1=O | Reverse transcriptase |
| CHEMBL317072 | CCN1c2ncccc2N(C)C(=O)c3cc(CCC(=O)N)cnc13 | Reverse transcriptase |
| CHEMBL190170 | CC(C)C1=C(OC2CCCCC2)c3cc(Cl)ccc3NC1=O | Reverse transcriptase |
| CHEMBL268136 | [K+].[K+].[K+].[K+].[K+].[K+].[K+].[K+].[K+].[K+].[K+].[K+].[K+].[K+].[K+].[K+].[K+].OC1C(OS(=O)(=O)[O-])[C@@H]2OC(CSCc3ccccc3)[C@H]1O[C@H]4OC(COS(=O)(=O)[O-])[C@@H](O[C@H]5OC(CSCc6ccccc6)[C@@H](O[C@@H]7OC(COS(=O)(=O)[O-])[C@@H](O[C@H]8OC(CSCc9ccccc9)[C@@H](O[C@H]%10OC(COS(=O)(=O)[O-])[C@@H](O[C@H]%11OC(COS(=O)(=O)[O-])[C@@H](O2)C(OOS(=O)(=O)[O-])C%11OS(=O)(=O)[O-])C(OOS(=O)(=O)[O-])C%10OS(=O)(=O)[O-])C(OOS(=O)(=O)[O-])C8OS(=O)(=O)[O-])C(OOS(=O)(=O)[O-])C7OS(=O)(=O)[O-])C(OOS(=O)(=O)[O-])C5OS(=O)(=O)[O-])C(OOS(=O)(=O)[O-])C4OS(=O)(=O)[O-] | Reverse transcriptase |
| CHEMBL55610 | CN1C(=O)c2cc(O)ccc2Oc3cc(C)ccc13 | Reverse transcriptase |
| CHEMBL144591 | O=C1Nc2c(CC3=CCNC3)ccnc2N(C4CC4)c5ncccc15 | Reverse transcriptase |
| CHEMBL1203236 | Cl.COc1ccc2c(C)nc(CCCCCCC(=O)c3ccccc3)n2n1 | Reverse transcriptase |
| CHEMBL435017 | Cc1nc(CCCCCCC(=O)c2ccccc2)n3nc(ccc13)n4ccnn4 | Reverse transcriptase |
| CHEMBL69657 | CCOCCOCN1C(=O)NC(=O)C2=C1Sc3ccccc3NC2 | Reverse transcriptase |
| CHEMBL303466 | CN1C(=O)c2cccnc2N(C#C)c3ccccc13 | Reverse transcriptase |
| CHEMBL606999 | [Na+].[Na+].[Na+].[Na+].CC1=CN([C@H]2C=C[C@]3(COP(=O)([O-])OP(=O)([O-])OP(=O)([O-])[O-])C[C@H]23)C(=O)NC1=O | Reverse transcriptase |
| CHEMBL359410 | S=C(NCCc1occc1)Nc2nccs2 | Reverse transcriptase |
| CHEMBL8971 | CCc1c(CCO)nn(c2ccccc2)c1c3ccccc3 | Reverse transcriptase |
| CHEMBL48629 | FC(F)(F)C1(C#CC2CC2)N(CC=C)c3cc(Cl)ccc3NC1=O | Reverse transcriptase |
| CHEMBL302931 | CCN1C(=O)c2cccc(C)c2N(CC)c3ncccc13 | Reverse transcriptase |
| CHEMBL126214 | CCN(Cc1oc2c(Cl)ccc(Cl)c2n1)c3cc(CC)c(C)nc3O | Reverse transcriptase |
| CHEMBL294848 | CCCN1C(=O)c2cc(N)ccc2Oc3ccc(C)c(C)c13 | Reverse transcriptase |
| CHEMBL321026 | Cc1ccc(cc1)C(=O)CSc2nc(O)c(C)c(Cc3cccc4ccccc34)n2 | Reverse transcriptase |
| CHEMBL308222 | CC1CN(CC(=C)C)Cc2cc(Cl)cc3NC(=S)N1c23 | Reverse transcriptase |
| CHEMBL301986 | CCCCOC(=O)N1c2ccccc2NC(=O)C1(C#CC3CC3)C(F)(F)F | Reverse transcriptase |
| CHEMBL85533 | CCc1cc(NCc2cccnc2OC)c(O)nc1C | Reverse transcriptase |
| CHEMBL133010 | COC(=O)c1cc(cc(Cl)c1OC)C(=CCCCC(=O)N(C)C)c2cc(Cl)c(OC)c(c2)C(=O)OC | Reverse transcriptase |
| CHEMBL70562 | CCN1C(=O)c2ccccc2N(CC)c3ncccc13 | Reverse transcriptase |
| CHEMBL23361 | Nc1nc(O)c2ncn(C3CC(OCP(=O)(O)OP(=O)(O)OP(=O)(O)OP(=O)(O)COC4CC(C=C4)n5cnc6c(O)nc(N)nc56)C=C3)c2n1 | Reverse transcriptase |
| CHEMBL387370 | CC1=CN([C@@H]2O[C@H](CO[Si](C)(C)C(C)(C)C)[C@@]3(OS(=O)(=O)C=C3N)[C@H]2O[Si](C)(C)C(C)(C)C)C(=O)N(CCCO)C1=O | Reverse transcriptase |
| CHEMBL356278 | O=C1Nc2c(CN3CCCC3)ccnc2N(C4CC4)c5ncccc15 | Reverse transcriptase |
| CHEMBL215724 | COC(=O)CCC\C=C(\c1ccc(cc1)C#N)/c2cc(C)c3onc(OC)c3c2 | Reverse transcriptase |
| CHEMBL103235 | CCN1c2nc(Cl)ccc2N(C)C(=O)c3cc(COc4cccc(c4)C(=O)OC)cnc13 | Reverse transcriptase |
| CHEMBL40631 | Clc1ccccc1CCNC(=S)Nc2ccc(Br)cn2 | Reverse transcriptase |
| CHEMBL40625 | Fc1cccc(CC\N=C(/S)\Nc2ccc(Br)cn2)c1 | Reverse transcriptase |
| CHEMBL114559 | Brc1ccc(NC(=S)NCCc2ccc3OCOc3c2)nc1 | Reverse transcriptase |
| CHEMBL411261 | [Na+].[Na+].[Na+].[Na+].[Na+].[Na+].[Na+].[Na+].[Na+].[Na+].[Na+].[Na+].COc1ccc(NCC2O[C@@H]3O[C@@H]4C(CNc5ccc(OC)cc5)O[C@H](O[C@@H]6C(CNc7ccc(OC)cc7)O[C@H](O[C@H]8C(O)C(OS(=O)(=O)[O-])[C@H](OC8CNc9ccc(OC)cc9)O[C@@H]%10C(CNc%11ccc(OC)cc%11)O[C@@H](O[C@@H]%12C(CNc%13ccc(OC)cc%13)O[C@H](O[C@@H]%14C(CNc%15ccc(OC)cc%15)O[C@H](O[C@H]2C(O)C3OS(=O)(=O)[O-])C(OS(=O)(=O)[O-])C%14OOS(=O)(=O)[O-])C(OS(=O)(=O)[O-])C%12OOS(=O)(=O)[O-])C(OS(=O)(=O)[O-])C%10OOS(=O)(=O)[O-])C(OS(=O)(=O)[O-])C6OOS(=O)(=O)[O-])C(OS(=O)(=O)[O-])C4OOS(=O)(=O)[O-])cc1 | Reverse transcriptase |
| CHEMBL187718 | CCCC1=C(OC2CCCCC2)c3cc(Cl)ccc3NC1=O | Reverse transcriptase |
| CHEMBL134227 | C[C@H]1Oc2c3C=CC(C)(C)Oc3c4C(=CC(=O)Oc4c2[C@@H](OC(=O)C)[C@@H]1C)c5ccccc5 | Reverse transcriptase |
| CHEMBL314270 | Cc1ccc2C(=O)N3CCSC3(c4ccccc4)c2c1 | Reverse transcriptase |
| CHEMBL297453 | Oc1cc(O)c2C[C@@H](OC(=O)c3cc(O)c(O)c(O)c3)[C@H](Oc2c1)c4cc(O)c(O)c(O)c4 | Reverse transcriptase |
| CHEMBL123478 | CCN(C1CCN(CC1)C(=O)c2cc3cc(NS(=O)(=O)C)ccc3[nH]2)c4nc(F)ccc4NC(C)(C)C | Reverse transcriptase |
| CHEMBL121881 | CC(C)Nc1ccnnc1N(C)C2CCN(CC2)C(=O)c3cc4cc(NS(=O)(=O)C)ccc4[nH]3 | Reverse transcriptase |
| CHEMBL358555 | CCN1c2nc(ccc2N(C)C(=O)c3cccnc13)c4cnc[nH]4 | Reverse transcriptase |
| CHEMBL100942 | CCN1c2ncccc2N(C)C(=O)c3cc(C)cnc13 | Reverse transcriptase |
| CHEMBL198425 | COC(=O)CCC\C=C(/c1cccc(c1)C#N)\c2cc(Cl)c(OC)c(c2)C(=O)OC | Reverse transcriptase |
| CHEMBL2115334 | CN1C(=O)c2cccnc2N(C3CC3)c4cc(Cl)cnc14 | Reverse transcriptase |
| CHEMBL37789 | CC(C)Nc1cccnc1N2CCN(CC2)C(=O)c3cc4ccc(CO)cc4[nH]3 | Reverse transcriptase |
| CHEMBL39950 | CCN1c2ncc(C)cc2N(C)C(=O)c3cccnc13 | Reverse transcriptase |
| CHEMBL36327 | Oc1cc(O)c2C[C@@H](OC(=O)c3cc(O)c(O)c(O)c3)[C@H](Oc2c1)c4ccc(O)c(O)c4 | Reverse transcriptase |
| CHEMBL106646 | CCc1cc(c(O)nc1C)C(OC)(C#CC2CC2)C(F)(F)F | Reverse transcriptase |
| CHEMBL313175 | Cc1ccc(cc1)C23SCCN2C(=O)c4ccccc34 | Reverse transcriptase |
| CHEMBL48637 | CCN1c2ncccc2c3nccn3c4cccnc14 | Reverse transcriptase |
| CHEMBL85425 | CCc1cc(NCc2ccccc2C#N)c(O)nc1C | Reverse transcriptase |
| CHEMBL125896 | CCN(C1CCN(CC1)C(=O)c2cc3cc(NS(=O)(=O)N4CCN(C)CC4)ccc3[nH]2)c5ncccc5NC(C)(C)C | Reverse transcriptase |
| CHEMBL94779 | Fc1ccc(F)c2c1NC(=O)OC2(C#CC3CC3)C(F)(F)F | Reverse transcriptase |
| CHEMBL283630 | CCCC1OC(C#CC2CC2)(c3cc(Cl)ccc3NC1=O)C(F)(F)F | Reverse transcriptase |
| CHEMBL314572 | CCC(c1cc(O)nc(SC(C)C)n1)c2cccc3ccccc23 | Reverse transcriptase |
| CHEMBL607724 | CCCCCCCCC(=O)NC1=NN(C)c2ncnc3c2c1cn3C4O[C@H](CO)[C@@H](O)[C@H]4O | Reverse transcriptase |
| CHEMBL100394 | CCN1c2nc(Cl)ccc2N(C)C(=O)c3cc(COc4ccc(cc4)C(=O)OC)cnc13 | Reverse transcriptase |
| CHEMBL157777 | CSc1nc(O)cc(Cc2c(F)cccc2F)n1 | Reverse transcriptase |
| CHEMBL207123 | COC(=O)C1=C([C@H]2c3ccccc3S[C@@]12C(=O)OC)N4CCCC4 | Reverse transcriptase |
| CHEMBL608301 | CCCCC(=O)NC1=NN(C)c2ncnc3c2c1cn3C4O[C@H](CO)[C@@H](O)[C@H]4O | Reverse transcriptase |
| CHEMBL317765 | Fc1cc(F)c2NC(=O)OC(C#CC3CC3)(c2c1F)C(F)(F)F | Reverse transcriptase |
| CHEMBL145279 | CCN1c2nc(OC)cc(C3OCCCO3)c2NC(=O)c4cccnc14 | Reverse transcriptase |
| CHEMBL357187 | COC(=O)c1cc(cc(Cl)c1OC)C(=CCCCCN=[N+]=[N-])c2cc(Cl)c(OC)c(c2)C(=O)OC | Reverse transcriptase |
| CHEMBL305612 | Nc1ncnc2c1ncn2\C=C/3\CC3CO | Reverse transcriptase |
| CHEMBL214391 | CC1=CN([C@@H]2O[C@H](CO[Si](C)(C)C(C)(C)C)[C@@]3(OS(=O)(=O)C=C3N)[C@H]2O[Si](C)(C)C(C)(C)C)C(=O)N(CCc4ccccc4)C1=O | Reverse transcriptase |
| CHEMBL111413 | Nc1ccc2NC(=O)OC(C#CC3CC3)(c2c1)C(F)(F)F | Reverse transcriptase |
| CHEMBL94550 | CN(C)c1ccc2NC(=O)OC(C#CC3CC3)(c2c1)C(F)(F)F | Reverse transcriptase |
| CHEMBL145004 | CCN1c2ncccc2C(=O)N(C)c3ccc(nc13)c4ccccc4OC | Reverse transcriptase |
| CHEMBL2368776 | CCCN1C=Nc2c(ncn2[C@@H]3O[C@H](COC)[C@@H](OP(=O)(C)O)[C@H]3OC)C1=S | Reverse transcriptase |
| CHEMBL351375 | O=C1N2CCS[C@@H](c3ccccc23)c4ccccc14 | Reverse transcriptase |
| CHEMBL331684 | CCN(C1CCN(CC1)C(=O)c2cc3cc(NS(=O)(=O)N4CCN(C)CC4)ccc3[nH]2)c5nc(F)ccc5NC(C)(C)C | Reverse transcriptase |
| CHEMBL124248 | Cc1nc(CCCCCCC(=O)c2ccccc2)n3nc(Cl)ccc13 | Reverse transcriptase |
| CHEMBL310490 | CN1C(=O)c2cccnc2N(CC=C)c3ncccc13 | Reverse transcriptase |
| CHEMBL290410 | CSCN1c2ncccc2N(C)C(=O)c3cccnc13 | Reverse transcriptase |
| CHEMBL142478 | COc1ccc(O)c(Cc2ccnc3N(C4CC4)c5ncccc5C(=O)Nc23)c1 | Reverse transcriptase |
| CHEMBL70743 | CCN1c2nc(N)c(Cl)cc2N(C)C(=O)c3cccnc13 | Reverse transcriptase |
| CHEMBL1788126 | [Na+].[Na+].CCCCCCCCCCCCCCCC(=O)Oc1cc(cc2cc(cc(OC(=O)CCCCCCCCCCCCCCC)c12)S(=O)(=O)[O-])S(=O)(=O)[O-] | Reverse transcriptase |
| CHEMBL313212 | [O-][N+](=O)c1cccc(c1)C23SCCN2C(=O)c4ccccc34 | Reverse transcriptase |
| CHEMBL331726 | CCc1nc(O)c(NCN2C(=O)c3ccccc3C2=O)cc1C | Reverse transcriptase |
| CHEMBL305416 | CCN1c2cc(ccc2N(C)C(=O)c3cc(Br)cnc13)[N+](=O)[O-] | Reverse transcriptase |
| CHEMBL55387 | COc1cc(cc(c1)C(F)(F)F)[S+]([O-])c2cccc(N)c2C#N | Reverse transcriptase |
| CHEMBL111001 | CC(C)C#CC1(OC(=O)Nc2ccc(N)cc12)C(F)(F)F | Reverse transcriptase |
| CHEMBL57178 | Fc1ccc(CO[C@H]2C(=O)Nc3ccc(Cl)cc3[C@@]2(C#CC4CC4)C(F)(F)F)cc1 | Reverse transcriptase |
| CHEMBL146770 | S=C(NCCC1=CCCCC1)Nc2nccs2 | Reverse transcriptase |
| CHEMBL284241 | Cc1cccc(Cc2nc(SC3CCCCC3)nc(O)c2C)c1 | Reverse transcriptase |
| CHEMBL151791 | COc1cc(OC)c2CC(COc2c1)OC(=O)c3cc(O)c(O)c(O)c3 | Reverse transcriptase |
| CHEMBL372175 | COC(=O)CCC\C=C(/c1cc(F)cc(c1)C(F)(F)F)\c2cc(C)c3onc(OC)c3c2 | Reverse transcriptase |
| CHEMBL170810 | COc1ccc(CC\N=C(/S)\Nc2ccc(Br)cn2)cc1 | Reverse transcriptase |
| CHEMBL33981 | S=C(NCCc1ccccc1)Nc2nccs2 | Reverse transcriptase |
| CHEMBL345908 | CCC(C)Sc1nc(O)c(C)c(Cc2cccc(Cl)c2)n1 | Reverse transcriptase |
| CHEMBL314884 | O=C1N2CCSC2(c3cccc4CCCc34)c5ccccc15 | Reverse transcriptase |
| CHEMBL54977 | CCCN1C(=O)c2cc(N)ccc2Oc3c(C)cc(C)cc13 | Reverse transcriptase |
| CHEMBL149275 | Nc1ccc(Cl)cc1S(=O)(=O)n2cccc2C(=O)OCC=C | Reverse transcriptase |
| CHEMBL83362 | O=C1N2CCSC2(c3ccccc13)c4c5ccccc5cc6ccccc46 | Reverse transcriptase |
| CHEMBL357664 | CCN1c2ncccc2C(=O)Nc3c(COCc4ccccc4)cc(OC)nc13 | Reverse transcriptase |
| CHEMBL125213 | COC(=O)\C=C\CSc1nc(N)cc(Cl)n1 | Reverse transcriptase |
| CHEMBL1161005 | CC1=CN([C@H]2CC(N=[N+]=[N-])C(COP(=S)(S)OP(=O)(O)OP(=O)(O)O)O2)C(=O)NC1=O | Reverse transcriptase |
| CHEMBL296376 | Cc1cccc(Sc2cccc(N)c2C#N)c1 | Reverse transcriptase |
| CHEMBL281239 | CCC#CC1(OCC(=O)Nc2ccc(Cl)cc12)C(F)(F)F | Reverse transcriptase |
| CHEMBL316151 | COc1cccc(c1)C23SCCN2C(=O)c4ccccc34 | Reverse transcriptase |
| CHEMBL338872 | CCc1cc(NC(C)c2oc3ccccc3c2)c(O)nc1C | Reverse transcriptase |
| CHEMBL262374 | Cc1ccc(C)c(c1)C23SCCN2C(=O)c4ccccc34 | Reverse transcriptase |
| CHEMBL318343 | CCN1c2ncc(CCc3ccnc(c3)C(=O)O)cc2C(=O)N(C)c4ccc(Cl)nc14 | Reverse transcriptase |
| CHEMBL318562 | CCN1c2ncc(CCc3ccc4ccccc4c3)cc2C(=O)N(C)c5ccc(Cl)nc15 | Reverse transcriptase |
| CHEMBL356314 | CCN1c2ncccc2C(=O)N(C)c3ccc(N)nc13 | Reverse transcriptase |
| CHEMBL142664 | CCN1c2nc(ccc2N(C)C(=O)c3cccnc13)c4ccc(O)nc4 | Reverse transcriptase |
| CHEMBL307280 | CCN1c2ncccc2C(=O)N(C)c3cc(Cl)c(nc13)[N+](=O)[O-] | Reverse transcriptase |
| CHEMBL144949 | CCN1c2nc(cc(C)c2NC(=O)c3cccnc13)C(C)C | Reverse transcriptase |
| CHEMBL297956 | Nc1cccc(c1C#N)S(=O)(=O)c2cccc3ccccc23 | Reverse transcriptase |
| CHEMBL355333 | COc1ccccc1CC\N=C(/S)\Nc2ccc(Br)cn2 | Reverse transcriptase |
| CHEMBL425146 | O\C(=C/C(=O)c1cc[nH]c1)\c2oc(Cc3ccc(F)cc3)cc2 | Reverse transcriptase |
| CHEMBL2373970 | OC(=O)CN1C[C@]23[C@H]4[C@@H]5c6c7c8[C@H]9[C@@H]%10[C@@H]%11[C@H]%12C%13=C%14C%15=C%11c%16c%10c%17c8c%18c6c%19c5c%20c%21c%22c(c%23C%24=C2c%25c([C@@H]%13[C@H]%24c%26c%14c%27c%15c%28c%16c%29c%17c%18c%30c%19c%21c%31c%30c%29c%28c%32c%27c(c%23%26)c%22c%31%32)c%12c9c7c4%25)[C@]3%20[C@@H]1C(=O)O | Reverse transcriptase |
| CHEMBL306225 | CCN1c2nccc(O)c2NC(=O)c3cccnc13 | Reverse transcriptase |
| CHEMBL73015 | CCCCN1c2ccccc2N(C)C(=O)c3cccnc13 | Reverse transcriptase |
| CHEMBL412157 | CN1C(=O)c2cccnc2N(CC(=O)OC(C)(C)C)c3ccccc13 | Reverse transcriptase |
| CHEMBL72874 | CCN1c2ccc(cc2N(C)C(=O)c3cccnc13)[N+](=O)[O-] | Reverse transcriptase |
| CHEMBL306247 | CCN1c2cc(cc(Br)c2N(C)C(=O)c3cccnc13)C(F)(F)F | Reverse transcriptase |
| CHEMBL302034 | CN(C)CCN1C(=O)c2cccnc2N(C3CC3)c4ncccc14 | Reverse transcriptase |
| CHEMBL73753 | CN1C(=O)c2cccnc2N(C)c3ccccc13 | Reverse transcriptase |
| CHEMBL71063 | O=C1Nc2cccnc2N(C3CCCC3)c4ncccc14 | Reverse transcriptase |
| CHEMBL72392 | CN1c2ccccc2NC(=O)c3cccnc13 | Reverse transcriptase |
| CHEMBL70720 | CN1C(=O)c2ccccc2N(C)c3ncccc13 | Reverse transcriptase |
| CHEMBL307792 | O=C1Nc2cccnc2Nc3ccccc13 | Reverse transcriptase |
| CHEMBL311814 | CCN1C(=O)c2cccnc2Nc3ccccc13 | Reverse transcriptase |
| CHEMBL70881 | COCCN1C(=O)c2cccnc2Nc3ccccc13 | Reverse transcriptase |
| CHEMBL73468 | CCN1c2ncccc2NC(=O)c3cc(C)cnc13 | Reverse transcriptase |
| CHEMBL312072 | CCN1c2ccccc2N(CF)C(=O)c3cccnc13 | Reverse transcriptase |
| CHEMBL1738875 | CC1=CN([C@@H]2O[C@H](CO[Si](C)(C)C(C)(C)C)[C@@]3(OS(=O)(=O)C=C3N)[C@H]2O[Si](C)(C)C(C)(C)C)C(=O)NC1=O | Reverse transcriptase |
| CHEMBL111153 | CCC(C)Sc1nc(O)cc(Cc2ccccc2)n1 | Reverse transcriptase |
| CHEMBL428685 | COC(=O)CCCC=C(c1cc(C)c(OC)c(c1)C(=O)OC)c2cc(C)c(OC)c(c2)C(=O)OC | Reverse transcriptase |
| CHEMBL329938 | CCN1c2ncc(CCc3ccccc3C)cc2C(=O)N(C)c4ccc(Cl)nc14 | Reverse transcriptase |
| CHEMBL253996 | Fc1ccc(CCNC(=S)Nc2nccs2)cc1 | Reverse transcriptase |
| CHEMBL344134 | CCN1c2ncccc2C(=O)Nc3c(C)cc(nc13)C(C)(C)C | Reverse transcriptase |
| CHEMBL70753 | CCC(=O)N1c2ccccc2N(CC=C)C(=O)c3cccnc13 | Reverse transcriptase |
| CHEMBL308491 | CN1C(=O)c2cccnc2N(CCO)c3ccccc13 | Reverse transcriptase |
| CHEMBL100568 | CCN1c2ncccc2N(C)C(=O)c3cc(cnc13)c4cccc(OC)c4 | Reverse transcriptase |
| CHEMBL313273 | Cc1ccnc(c1)C23SCCN2C(=O)c4ccccc34 | Reverse transcriptase |
| CHEMBL70538 | CC(=O)N1c2ccccc2N(CC=C)C(=O)c3cccnc13 | Reverse transcriptase |
| CHEMBL310699 | CN1C(=O)c2cccnc2N(c3ncccc13)S(=O)(=O)C | Reverse transcriptase |
| CHEMBL302498 | Cc1cc(C)c2NC(=O)c3ccccc3Nc2n1 | Reverse transcriptase |
| CHEMBL70258 | CN1C(=O)c2cccnc2N(C3CCCCC3)c4ncccc14 | Reverse transcriptase |
| CHEMBL74240 | CCN1c2ccccc2N(C)C(=O)c3ccccc13 | Reverse transcriptase |
| CHEMBL70047 | CCN1c2ccc(CO)cc2N(C)C(=O)c3cccnc13 | Reverse transcriptase |
| CHEMBL70511 | CCN1c2nccc(OC)c2NC(=O)c3cccnc13 | Reverse transcriptase |
| CHEMBL419629 | CN1C(=O)c2cccnc2N(CCF)c3ccccc13 | Reverse transcriptase |
| CHEMBL70754 | CCOC(=O)N1c2ccccc2N(C)C(=O)c3cccnc13 | Reverse transcriptase |
| CHEMBL306895 | CN1C(=O)c2cccnc2N(CC(=O)O)c3ccccc13 | Reverse transcriptase |
| CHEMBL611243 | CCCCCCCCCCCC(=O)NC1=NN(C)c2ncnc3c2c1cn3C4O[C@H](CO)[C@@H](O)[C@H]4O | Reverse transcriptase |
| CHEMBL141323 | CCC(=O)CCCC=C(c1cc(Cl)c(OC)c(c1)C(=O)OC)c2cc(Cl)c(OC)c(c2)C(=O)OC | Reverse transcriptase |
| CHEMBL305041 | CCN1c2ncccc2N(C)C(=O)c3ccc(C)nc13 | Reverse transcriptase |
| CHEMBL433487 | CN1C(=O)c2cccnc2Oc3cc(C)cc(N)c13 | Reverse transcriptase |
| CHEMBL289072 | CCN1c2ncc(Cl)cc2N(C)C(=O)c3cccnc13 | Reverse transcriptase |
| CHEMBL291267 | CN1C(=O)c2cccnc2N(C3CC3)c4nccc(C)c14 | Reverse transcriptase |
| CHEMBL99844 | CCN1c2ncccc2N(C)C(=O)c3cc(\C=C\c4ccncc4)cnc13 | Reverse transcriptase |
| CHEMBL305754 | CN1C(=O)c2ccccc2N(C)c3ccccc13 | Reverse transcriptase |
| CHEMBL304563 | Clc1ccc2Nc3ncccc3C(=O)N(CC=C)c2c1 | Reverse transcriptase |
| CHEMBL73477 | O=C1Nc2cccnc2N(c3ccccc3)c4ncccc14 | Reverse transcriptase |
| CHEMBL72225 | Cc1ccnc2N(C3CC3)c4nccc(O)c4C(=O)Nc12 | Reverse transcriptase |
| CHEMBL72916 | CCN1c2ncc(N)cc2N(C)C(=O)c3cccnc13 | Reverse transcriptase |
| CHEMBL37518 | CC(C)Nc1cccnc1N2CCN(CC2)C(=O)c3cc4cc(N)ccc4[nH]3 | Reverse transcriptase |
| CHEMBL175566 | O\C(=C/C(=O)c1nc[nH]n1)\c2ccc(Cc3ccc(F)cc3)[nH]2 | Reverse transcriptase |
| CHEMBL305243 | CCN1c2ccc(O)cc2N(C)C(=O)c3cccnc13 | Reverse transcriptase |
| CHEMBL418886 | OCCN1C(=O)c2cccnc2Nc3ccccc13 | Reverse transcriptase |
| CHEMBL311908 | CN1C(=O)c2cccnc2Nc3ccccc13 | Reverse transcriptase |
| CHEMBL306167 | CN(C)C(=O)CN1C(=O)c2cccnc2Nc3ccccc13 | Reverse transcriptase |
| CHEMBL72545 | CCN1c2ccc(CN)cc2N(C)C(=O)c3cccnc13 | Reverse transcriptase |
| CHEMBL312019 | CCN1c2ccc(cc2N(C)C(=O)c3cccnc13)C(=O)O | Reverse transcriptase |
| CHEMBL70854 | CCN1c2ccc(CNC(=O)C(C)(C)C)cc2N(C)C(=O)c3cccnc13 | Reverse transcriptase |
| CHEMBL72150 | CN1C(=O)c2cccnc2N(C(=O)C)c3ccccc13 | Reverse transcriptase |
| CHEMBL411368 | [K+].[K+].[K+].[K+].[K+].[K+].[K+].[K+].[K+].[K+].[K+].[K+].[K+].[K+].OC1C(OS(=O)(=O)[O-])[C@@H]2OC(COS(=O)(=O)[O-])[C@H]1O[C@H]3OC(COS(=O)(=O)[O-])[C@@H](O[C@H]4OC(COS(=O)(=O)[O-])[C@@H](O[C@H]5OC(COS(=O)(=O)[O-])[C@@H](O[C@@H]6OC(COS(=O)(=O)[O-])[C@@H](O[C@H]7OC(COS(=O)(=O)[O-])[C@@H](O[C@H]8OC(COS(=O)(=O)[O-])[C@@H](O2)C(O)C8OS(=O)(=O)[O-])C(O)C7OS(=O)(=O)[O-])C(O)C6OS(=O)(=O)[O-])C(O)C5OS(=O)(=O)[O-])C(O)C4OS(=O)(=O)[O-])C(O)C3OS(=O)(=O)[O-] | Reverse transcriptase |
| CHEMBL2115323 | CN1C(=O)c2cccnc2N(C3CC3)c4ccc(Cl)nc14 | Reverse transcriptase |
| CHEMBL39092 | OC(=O)c1ccc2ccc(\C=C\c3ccc(O)c(O)c3)nc2c1O | Reverse transcriptase |
| CHEMBL2115140 | CC[C@@H](C)N1c2ncccc2NC(=O)c3cccnc13 | Reverse transcriptase |
| CHEMBL304158 | CN1C(=O)c2c(C)cc(C)nc2N(C)c3ccc(C)cc13 | Reverse transcriptase |
| CHEMBL72135 | O=C1Nc2ccccc2Nc3ncccc13 | Reverse transcriptase |
| CHEMBL307451 | CN(C)CCN1c2cc(C)c(C)cc2N(C)C(=O)c3cccnc13 | Reverse transcriptase |
| CHEMBL70401 | CCN1c2ncccc2NC(=O)c3c(C)ccnc13 | Reverse transcriptase |
| CHEMBL303837 | CCN1C(=O)c2cccnc2N(C)c3ccccc13 | Reverse transcriptase |
| CHEMBL71631 | CCN1c2ncc(cc2N(C)C(=O)c3cccnc13)[N+](=O)[O-] | Reverse transcriptase |
| CHEMBL335768 | CCCCCC=C(c1cc(C)c(OC)c(c1)C(=O)OC)c2cc(C)c(OC)c(c2)C(=O)OC | Reverse transcriptase |
| CHEMBL328408 | CCN1c2ncccc2N(C)C(=O)c3cc(\C=C\C(=O)OC)cnc13 | Reverse transcriptase |
| CHEMBL330183 | CCN1c2ncccc2N(C)C(=O)c3cc(cnc13)C(=O)O | Reverse transcriptase |
| CHEMBL306794 | CCN(CC)C(=O)c1ccc2N(CC)c3ncccc3C(=O)N(C)c2c1 | Reverse transcriptase |
| CHEMBL71306 | CN1C(=O)c2cccnc2N(CC#C)c3ncccc13 | Reverse transcriptase |
| CHEMBL2114091 | CC[C@H](C)N1c2ncccc2NC(=O)c3cccnc13 | Reverse transcriptase |
| CHEMBL414112 | [K+].[K+].[K+].[K+].[K+].[K+].[K+].[K+].[K+].[K+].[K+].[K+].[K+].[K+].[K+].[K+].Cc1cc(C)c(c(C)c1)S(=O)(=O)OCC2O[C@@H]3O[C@H]4C(O)C(OS(=O)(=O)[O-])[C@H](OC4COS(=O)(=O)[O-])O[C@@H]5C(COS(=O)(=O)[O-])O[C@H](O[C@H]6C(O)C(OS(=O)(=O)[O-])[C@H](OC6COS(=O)(=O)[O-])O[C@@H]7C(COS(=O)(=O)[O-])O[C@@H](O[C@@H]8C(COS(=O)(=O)[O-])O[C@H](O[C@H]9C(O)C(OS(=O)(=O)[O-])[C@H](OC9COS(=O)(=O)[O-])O[C@H]2C(O)C3OS(=O)(=O)[O-])C(OS(=O)(=O)[O-])C8OOS(=O)(=O)[O-])C(OS(=O)(=O)[O-])C7OOS(=O)(=O)[O-])C(OS(=O)(=O)[O-])C5OOS(=O)(=O)[O-] | Reverse transcriptase |
| CHEMBL198551 | COC(=O)c1cc(cc(C)c1OC)\C(=C/CCN2CCOC2=O)\c3ccc(OC)c(OC)c3 | Reverse transcriptase |
| CHEMBL257695 | CC1=CN([C@H]2C[C@H](N=[N+]=[N-])[C@@H](COP(=O)(O)O)O2)C(=O)NC1=O | Reverse transcriptase |
| CHEMBL70572 | Cc1cccc2Nc3ncccc3C(=O)Nc12 | Reverse transcriptase |
| CHEMBL307162 | CC(C)(C)OC(=O)CN1C(=O)c2cccnc2Nc3ccccc13 | Reverse transcriptase |
| CHEMBL304965 | CCN1c2ccccc2N(C)C(=O)c3cc(cnc13)C(F)(F)C(F)(F)F | Reverse transcriptase |
| CHEMBL70532 | CCN1c2cc(ccc2N(C)C(=O)c3cccnc13)C(=O)c4ccccc4 | Reverse transcriptase |
| CHEMBL308229 | CCN1c2cc(NC(=O)C)ccc2N(C)C(=O)c3cccnc13 | Reverse transcriptase |
| CHEMBL70702 | Cc1ccc2NC(=O)c3ccccc3Nc2n1 | Reverse transcriptase |
| CHEMBL69666 | CCN1c2ccc(cc2N(C)C(=O)c3cc(Br)cnc13)[N+](=O)[O-] | Reverse transcriptase |
| CHEMBL304615 | CCN1c2cccc(C)c2N(Cc3ccccc3)C(=O)c4cccnc14 | Reverse transcriptase |
| CHEMBL61152 | Nc1ccccc1CO[C@H]2C(=O)Nc3ccc(Cl)cc3[C@@]2(C#CC4CC4)C(F)(F)F | Reverse transcriptase |
| CHEMBL340403 | CCN(C1CCN(CC1)C(=O)c2cc3cc(NS(=O)(=O)C)ccc3[nH]2)c4nc(Cl)ccc4NC(C)(C)C | Reverse transcriptase |
| CHEMBL145730 | O=C1Nc2c(CSc3ccccc3)ccnc2N(C4CC4)c5ncccc15 | Reverse transcriptase |
| CHEMBL422839 | O=C1Nc2c(CN3CCOCC3)ccnc2N(C4CC4)c5ncccc15 | Reverse transcriptase |
| CHEMBL543611 | Cl.Brc1ccc(NC(=S)NCCc2ccccn2)nc1 | Reverse transcriptase |
| CHEMBL357877 | COc1ccc(OCc2ccnc3N(C4CC4)c5ncccc5C(=O)Nc23)cc1 | Reverse transcriptase |
| CHEMBL87799 | FC(F)(F)c1cccc(c1)C23SCCN2C(=O)c4ccccc34 | Reverse transcriptase |
| CHEMBL124556 | CC(O)c1cc(NCc2oc3c(Cl)ccc(Cl)c3n2)c(O)nc1C | Reverse transcriptase |
| CHEMBL124992 | CCc1cc(N(C)Cc2oc3c(Cl)ccc(Cl)c3c2)c(O)nc1C | Reverse transcriptase |
| CHEMBL294406 | CCCO[C@H]1C(=O)Nc2ccc(Cl)cc2[C@@]1(C#CC3CC3)C(F)(F)F | Reverse transcriptase |
| CHEMBL293806 | Nc1cccc(Sc2cccc(c2)C#N)c1C#N | Reverse transcriptase |
| CHEMBL294627 | Cc1cc(C)cc(Sc2cccc(N)c2C#N)c1 | Reverse transcriptase |
| CHEMBL60805 | Cc1ccc(c(C)c1)S(=O)(=O)n2ccc(c2)C(O)c3ccc(Cl)cc3Cl | Reverse transcriptase |
| CHEMBL420460 | CCN1c2ncccc2N(C)C(=O)c3cc(NC(=O)N)cnc13 | Reverse transcriptase |
| CHEMBL306257 | Nc1nc(O)c2ncn(\C=C/3\CC3CO)c2n1 | Reverse transcriptase |
| CHEMBL418885 | CCN1c2ccc(cc2N(C)C(=O)c3cccnc13)C(=O)OC | Reverse transcriptase |
| CHEMBL174267 | Cc1nc(O)c(CCc2oc3ccccc3n2)cc1c4ccccc4 | Reverse transcriptase |
| CHEMBL85697 | CCc1cccc(c1)C23SCCN2C(=O)c4ccccc34 | Reverse transcriptase |
| CHEMBL351323 | CC(C)CSc1nc(O)c(C)c(Cc2c(Cl)cccc2Cl)n1 | Reverse transcriptase |
| CHEMBL105018 | CC1=CN(C2OC(CO[Si](C)(C)C(C)(C)C)CC2O[Si](C)(C)C(C)(C)C)C(=O)N(CC(=O)O)C1=O | Reverse transcriptase |
| CHEMBL147375 | S=C(NCCc1cccc(Oc2ccccc2)c1)Nc3nccs3 | Reverse transcriptase |
| CHEMBL49334 | CCN1c2ncccc2c3ncc(CC)n3c4cccnc14 | Reverse transcriptase |
| CHEMBL400684 | Oc1ccccc1CCNC(=S)Nc2nccs2 | Reverse transcriptase |
| CHEMBL121834 | CCc1cc(NCC2=COc3ccccc3C2=O)c(O)nc1C | Reverse transcriptase |
| CHEMBL149055 | CC(C)Oc1cccnc1N2CCN(CC2)C(=O)c3cc4cc(NS(=O)(=O)C)ccc4[nH]3 | Reverse transcriptase |
| CHEMBL288062 | CCN1c2ncccc2c3ncc(C)n3c4ccc(nc14)N(C)C | Reverse transcriptase |
| CHEMBL106204 | CC(=O)CSc1nc(O)c(C)c(Cc2cccc3ccccc23)n1 | Reverse transcriptase |
| CHEMBL141443 | [O-][N+](=O)c1ccc(OCc2ccnc3N(C4CC4)c5ncccc5C(=O)Nc23)cc1 | Reverse transcriptase |
| CHEMBL124991 | CCc1cc(NC(C)c2ccc3ccccc3c2)c(O)nc1C | Reverse transcriptase |
| CHEMBL269591 | Cc1nn(c2ccccc2)c(c1CC#N)c3ccccc3 | Reverse transcriptase |
| CHEMBL2115319 | CC[C@H]1O[C@@](C#CC2CC2)(c3cc(F)ccc3NC1=O)C(F)(F)F | Reverse transcriptase |
| CHEMBL439929 | CC(C)COC(=O)N1c2ccccc2NC(=O)C1(C#CC3CC3)C(F)(F)F | Reverse transcriptase |
| CHEMBL125666 | Cc1nc(O)c(NCc2oc3ccccc3n2)cc1S(=O)(=O)C | Reverse transcriptase |
| CHEMBL303241 | C[C@@H]1CN2C(=S)Nc3cccc([C@H](C)N1CC=C(C)C)c23 | Reverse transcriptase |
| CHEMBL156636 | CCCCSc1nc(O)c(C)c(Cc2c(Cl)cccc2Cl)n1 | Reverse transcriptase |
| CHEMBL345580 | CC(C)Sc1nc(O)c(C)c(Cc2c(Cl)cccc2Cl)n1 | Reverse transcriptase |
| CHEMBL215542 | COC(=O)CCC\C=C(\c1ccc(cc1)C#N)/c2cc(C)c3OC(=O)N(C)c3c2 | Reverse transcriptase |
| CHEMBL114598 | Brc1ccc(NC(=S)NCCc2occc2)nc1 | Reverse transcriptase |
| CHEMBL2373486 | OC(=O)C1(C(=O)O)[C@]23[C@H]4[C@@H]5c6c7c8[C@H]9[C@H]%10c%11c%12c8c%13c6c%14c5c%15c%16c%17c%18c%19c%20c%21c%22c%18c(c%14%17)c%13c%12c%22c%23c%11c%24c%25C%26=C%27[C@H]%28[C@@H]%29C(=C2c%30c4c7c9c(c%28%30)[C@@]%27%31[C@@]%10%24C%31(C(=O)O)C(=O)O)c%32c(c%16c%19c%33c%32c%29c%26c(c%25c%21%23)c%20%33)[C@]13%15 | Reverse transcriptase |
| CHEMBL357658 | CCNC(=O)OCCCCC=C(c1cc(Cl)c(OC)c(c1)C(=O)OC)c2cc(Cl)c(OC)c(c2)C(=O)OC | Reverse transcriptase |
| CHEMBL292339 | C[C@@H](\N=C(/S)\Nc1ccc(Cl)cn1)c2ccccc2 | Reverse transcriptase |
| CHEMBL145287 | CCN1c2ncccc2C(=O)N(C)c3ccc(nc13)c4ccc(OC)nc4 | Reverse transcriptase |
| CHEMBL289969 | CC(C)Nc1cccnc1N2CCN(CC2)C(=O)c3cc4ccccc4[nH]3 | Reverse transcriptase |
| CHEMBL37080 | CC(C)Nc1cccnc1N2CCN(CC2)C(=O)c3cc4ccc(O)cc4[nH]3 | Reverse transcriptase |
| CHEMBL145897 | S=C(NCc1ccccc1)Nc2nccs2 | Reverse transcriptase |
| CHEMBL112886 | CCC(C)Sc1nc(O)cc(Cc2cccc(C)c2)n1 | Reverse transcriptase |
| CHEMBL294743 | C[C@@H](\N=C(/S)\Nc1ccc(Br)cn1)C2CCCCC2 | Reverse transcriptase |
| CHEMBL322898 | CCC(C)Sc1nc(O)cc(Cc2ccc(cc2)[N+](=O)[O-])n1 | Reverse transcriptase |
| CHEMBL206629 | COC(=O)C1=C(C2c3ccccc3SC12C(=O)OC)N4CCCC4 | Reverse transcriptase |
| CHEMBL96328 | FC(F)(F)Oc1ccc2NC(=O)OC(C#CC3CC3)(c2c1)C(F)(F)F | Reverse transcriptase |
| CHEMBL144742 | Clc1ccc2[nH]c(C3=NCCS3)c(c2c1)S(=O)(=O)c4ccccc4 | Reverse transcriptase |
| CHEMBL291113 | O=C1Nc2c(ccnc2N(C3CC3)c4ncccc14)C#N | Reverse transcriptase |
| CHEMBL175089 | CCc1cc(CNc2oc3ccccc3n2)c(O)nc1C | Reverse transcriptase |
| CHEMBL338658 | CCc1cc(NCc2oc3ccc(C)cc3n2)c(O)nc1C | Reverse transcriptase |
| CHEMBL124186 | CCc1cc(NCc2oc3cc(F)ccc3n2)c(O)nc1C | Reverse transcriptase |
| CHEMBL85945 | CCc1cc(NCc2cccc(OC)c2)c(O)nc1C | Reverse transcriptase |
| CHEMBL208008 | Cc1cc(C)c(c(C)c1)n2nnnc2SCC(=O)Nc3ccc(cc3)S(=O)(=O)N | Reverse transcriptase |
| CHEMBL443996 | Fc1ccccc1CO[C@H]2C(=O)Nc3ccc(Cl)cc3[C@@]2(C#CC4CC4)C(F)(F)F | Reverse transcriptase |
| CHEMBL298243 | CCN1c2cccnc2N(C)C(=O)c3cccnc13 | Reverse transcriptase |
| CHEMBL433925 | CCOCc1ccnc2N(C3CC3)c4ncccc4C(=O)Nc12 | Reverse transcriptase |
| CHEMBL86178 | CCc1cc(NCc2c(C)cccc2OC)c(O)nc1C | Reverse transcriptase |
| CHEMBL269742 | [I-].[I-].CCCCCCCCCC[N@@+]1(C)C[C@]23[C@H]4[C@@H]5C6=C7c8c4c9C2=C%10[C@H]%11[C@H]%12c9c%13c8[C@@H]%14[C@@H]%15c%16c%13c%12c%17c%18c%11c%19c%10c%20c%21c%22c(c5c%23c6c%24c%25c%26c(c%15c%27c%16c%17c%28c%18c%29c%30c%31c%28c%27c%26c%31c%32c(c%30c%21c%19%29)c%22c%23c%25%32)[C@]%24%33C[N+](C)(C)C[C@@]7%14%33)[C@@]3%20C1 | Reverse transcriptase |
| CHEMBL338339 | Cc1nc(CCCCCOC(=O)c2ccccc2)n3nc(Cl)ccc13 | Reverse transcriptase |
| CHEMBL38068 | CC(C)Nc1cccnc1N2CCN(CC2)C(=O)c3cc4cc(O)ccc4[nH]3 | Reverse transcriptase |
| CHEMBL289304 | COc1ccc2cc([nH]c2c1)C(=O)N3CCN(CC3)c4ncccc4NC(C)C | Reverse transcriptase |
| CHEMBL2115326 | CCN1c2ncccc2N(C)C(=O)c3cc(Br)cnc13 | Reverse transcriptase |
| CHEMBL338415 | Cc1nc(CCCCCCC(=O)c2ccccc2)n3nc(ccc13)n4nccn4 | Reverse transcriptase |
| CHEMBL105630 | CCc1cc(c(O)nc1C)C(OCc2ccccc2)(C#CC3CC3)C(F)(F)F | Reverse transcriptase |
| CHEMBL313481 | O=C1N2CCSC2(c3occc3)c4ccccc14 | Reverse transcriptase |
| CHEMBL314925 | CCC(c1cc(O)nc(SC2CCCCC2)n1)c3cccc4ccccc34 | Reverse transcriptase |
| CHEMBL72535 | C=CCN1C(=O)c2cccnc2Nc3ccccc13 | Reverse transcriptase |
| CHEMBL38129 | CC(C)Nc1cccnc1N2CCN(CC2)C(=O)c3cc4ccc(C=O)cc4[nH]3 | Reverse transcriptase |
| CHEMBL86261 | CCc1cc(NCc2ccccc2F)c(O)nc1C | Reverse transcriptase |
| CHEMBL144794 | CCN1c2ncccc2C(=O)N(C)c3ccc(nc13)c4ccc(OC)cc4 | Reverse transcriptase |
| CHEMBL41458 | Cc1ncc([N+](=O)[O-])n1CCOC(c2ccccc2)c3ccc(Cl)cc3Cl | Reverse transcriptase |
| CHEMBL2373427 | [I-].[I-].C[N+]1(C)C[C@]23[C@H]4[C@@H]5c6c7c8[C@H]9[C@H]%10c%11c%12c8c%13c6c%14c5c%15c%16c%17c%18c%19c%20c%21c%22c%18c(c%14%17)c%13c%12c%22c%23c%11c%24c%25C%26=C%27[C@H]%28[C@@H]%29C(=C2c%30c4c7c9c(c%28%30)[C@]%27%31C[N+](C)(C)C[C@]%10%24%31)c%32c(c%16c%19c%33c%32c%29c%26c(c%25c%21%23)c%20%33)[C@]3%15C1 | Reverse transcriptase |
| CHEMBL151955 | CC(C)Oc1cccnc1N(C)C2CCN(CC2)C(=O)c3cc4cc(NS(=O)(=O)C)ccc4[nH]3 | Reverse transcriptase |
| CHEMBL55308 | COc1ccccc1S(=O)(=O)c2cccc(N)c2C#N | Reverse transcriptase |
| CHEMBL176814 | CCc1cc([S+]([O-])Cc2oc3c(Cl)ccc(Cl)c3n2)c(O)nc1C | Reverse transcriptase |
| CHEMBL320056 | CCOC(=O)CSc1nc(O)c(C(C)C)c(Cc2cccc3ccccc23)n1 | Reverse transcriptase |
| CHEMBL358782 | C=CCOCc1ccnc2N(C3CC3)c4ncccc4C(=O)Nc12 | Reverse transcriptase |
| CHEMBL58408 | FC(F)(F)[C@@]1(C#CC2CC2)[C@@H](OCC3CC3)C(=O)Nc4ccc(Cl)cc14 | Reverse transcriptase |
| CHEMBL85829 | CCc1cc(NCc2ccccc2Cl)c(O)nc1C | Reverse transcriptase |
| CHEMBL86215 | [O-][S+]1CCN2C(=O)c3ccccc3C12c4ccccc4 | Reverse transcriptase |
| CHEMBL292468 | Nc1cccc(CO[C@H]2C(=O)Nc3ccc(Cl)cc3[C@@]2(C#CC4CC4)C(F)(F)F)c1 | Reverse transcriptase |
| CHEMBL124578 | Nc1cc(Cl)nc(SCc2ccccc2)n1 | Reverse transcriptase |
| CHEMBL87284 | CCc1cc(NCc2ccccc2SC)c(O)nc1C | Reverse transcriptase |
| CHEMBL309696 | Clc1ccc2NC(=O)NC(C#Cc3cncnc3)(C4CC4)c2c1 | Reverse transcriptase |
| CHEMBL41119 | Cc1ncc([N+](=O)[O-])n1CCOC(c2ccccc2)c3ccc(cc3)C(C)(C)C | Reverse transcriptase |
| CHEMBL151773 | CCC(\N=C(/S)\Nc1nccs1)c2ccccc2 | Reverse transcriptase |
| CHEMBL317866 | CN(\C(=N\CCc1ccccc1)\S)c2nccs2 | Reverse transcriptase |
| CHEMBL51738 | COc1cccc(Sc2cccc(N)c2C#N)c1 | Reverse transcriptase |
| CHEMBL156208 | CC(C)Sc1nc(O)cc(Cc2c(Cl)cccc2Cl)n1 | Reverse transcriptase |
| CHEMBL160781 | CCC(C)Sc1nc(O)c(C)c(Cc2cccc(F)c2)n1 | Reverse transcriptase |
| CHEMBL326224 | CCCC#CC1(OC(=O)Nc2ccc(N)cc12)C(F)(F)F | Reverse transcriptase |
| CHEMBL9014 | CC#Cc1c(C)nn(c2ccccc2)c1c3ccccc3 | Reverse transcriptase |
| CHEMBL315689 | CCc1cc(NCc2ncccc2OC)c(O)nc1C | Reverse transcriptase |
| CHEMBL291970 | FC(F)(F)[C@@]1(C#CC2CC2)[C@@H](OCc3ccccn3)C(=O)Nc4ccc(Cl)cc14 | Reverse transcriptase |
| CHEMBL58607 | Cc1cccc2c1nc(c3c(F)cccc3F)n2Cc4ccccc4 | Reverse transcriptase |
| CHEMBL157349 | CCC(C)Sc1nc(O)cc(Cc2ccccc2Cl)n1 | Reverse transcriptase |
| CHEMBL114404 | Oc1cc(Cc2ccccc2)nc(SC3CCCC3)n1 | Reverse transcriptase |
| CHEMBL414976 | [I-].[I-].C[N+]1(C)C[C@]23[C@H]4[C@@H]5c6c7c8[C@H]9[C@H]%10C%11=C%12c8c%13c6c%14c5c%15c%16c%17c%18c%19c%20c([C@@H]%21[C@H]%11[C@@]%22%23C[N+](C)(C)C[C@]%22%24C%25=C%26[C@H]%27[C@@H]%28C(=C2c%29c%27c(C%26=C%10%23)c9c7c4%29)c%30c(c%16c%31c%18c%32c%20c%21c%24c%33c%25c%28c%30c%31c%32%33)[C@]3%15C1)c%12c%13c%19c%14%17 | Reverse transcriptase |
| CHEMBL42656 | CCN1c2ccncc2N(C)C(=O)c3cccnc13 | Reverse transcriptase |
| CHEMBL378943 | CC1=CN([C@@H]2O[C@H](CO[Si](C)(C)C(C)(C)C)[C@@]3(OS(=O)(=O)C=C3N)[C@H]2O[Si](C)(C)C(C)(C)C)C(=O)N(\C=C/CO)C1=O | Reverse transcriptase |
| CHEMBL313366 | Cc1cccc(c1C)C23SCCN2C(=O)c4ccccc34 | Reverse transcriptase |
| CHEMBL125504 | CCc1cc(NCc2oc3cc(C)ccc3n2)c(O)nc1C | Reverse transcriptase |
| CHEMBL125392 | Oc1ncccc1NCN2C(=O)c3ccccc3C2=O | Reverse transcriptase |
| CHEMBL67451 | CCCN1Cc2cccc3NC(=S)N(CC1C)c23 | Reverse transcriptase |
| CHEMBL56324 | Cc1cc(Cl)cc(Sc2cccc(N)c2C#N)c1 | Reverse transcriptase |
| CHEMBL384970 | [I-].[I-].C[N+]1(C)C[C@]23[C@H]4[C@@H]5C6=C7c8c4c9C2=C%10[C@H]%11[C@H]%12c9c%13c8[C@@H]%14[C@@H]%15c%16c%13c%12c%17c%18c%11c%19c%10c%20c%21c%22c(c5c%23c6c%24c%25c%26c(c%15c%27c%16c%17c%28c%18c%29c%30c%31c%28c%27c%26c%31c%32c(c%30c%21c%19%29)c%22c%23c%25%32)[C@]%24%33C[N+](C)(C)C[C@@]7%14%33)[C@@]3%20C1 | Reverse transcriptase |
| CHEMBL157685 | Cc1c(O)nc(SC2CCCC2)nc1Cc3c(Cl)cccc3Cl | Reverse transcriptase |
| CHEMBL195108 | OC(=O)c1ccccc1C(=O)NCCO\C(=N/c2ccc(I)cc2)\S | Reverse transcriptase |
| CHEMBL175531 | CCc1cc(CCc2oc3ccc(F)cc3n2)c(O)nc1C | Reverse transcriptase |
| CHEMBL88121 | OC(=O)c1cc(Br)ccc1NC(=O)CCCCC(=O)Nc2ccc(Br)cc2C(=O)O | Reverse transcriptase |
| CHEMBL379493 | CC1=CN([C@@H]2O[C@H](CO[Si](C)(C)C(C)(C)C)[C@@]3(OS(=O)(=O)C=C3N)[C@H]2O[Si](C)(C)C(C)(C)C)C(=O)N(CC(=O)N)C1=O | Reverse transcriptase |
| CHEMBL123966 | CCOC(=O)c1cc(NCc2oc3ccccc3n2)c(O)nc1C | Reverse transcriptase |
| CHEMBL2115322 | CCN1c2ccc(C)nc2N(C)C(=O)c3cccnc13 | Reverse transcriptase |
| CHEMBL301109 | CCN1c2ncccc2c3ncc(C)n3c4ccc(nc14)N5CCCC5 | Reverse transcriptase |
| CHEMBL59059 | Fc1cccc(CO[C@H]2C(=O)Nc3ccc(Cl)cc3[C@@]2(C#CC4CC4)C(F)(F)F)c1 | Reverse transcriptase |
| CHEMBL36835 | CC(C)Nc1cccnc1N2CCN(CC2)C(=O)c3cc4cc(NC(=O)C)ccc4[nH]3 | Reverse transcriptase |
| CHEMBL356163 | CC(C)Oc1cccnc1N2CCN(CC2)C(=O)c3cc4ccccc4[nH]3 | Reverse transcriptase |
| CHEMBL89524 | OC(=O)c1cc(Cl)ccc1NC(=O)CCCCC(=O)Nc2ccc(Cl)cc2C(=O)O | Reverse transcriptase |
| CHEMBL323573 | CCC(C)Sc1nc(O)c(C)c(Cc2ccc(cc2)[N+](=O)[O-])n1 | Reverse transcriptase |
| CHEMBL299271 | Nc1cccc(c1C#N)S(=O)(=O)c2cccc(c2)C#N | Reverse transcriptase |
| CHEMBL49030 | FC(F)(F)C1(C#CC2CC2)N(CC#C)c3ccccc3NC1=O | Reverse transcriptase |
| CHEMBL84717 | O=C1N2CCSC2(c3cccc(c3)C#N)c4ccccc14 | Reverse transcriptase |
| CHEMBL301996 | FC(F)(F)C1(C#CC2CC2)N(Cc3ccccc3)c4ccccc4NC1=O | Reverse transcriptase |
| CHEMBL106659 | CCc1cc(c(O)nc1C)C(OCC#N)(C#CC2CC2)C(F)(F)F | Reverse transcriptase |
| CHEMBL177192 | CN(C)c1cc(CCc2oc3ccccc3n2)c(O)nc1C | Reverse transcriptase |
| CHEMBL321484 | CCC#CC1(OC(=O)Nc2ccc(N)cc12)C(F)(F)F | Reverse transcriptase |
| CHEMBL289429 | CCN1c2ncccc2C(=O)N(C)c3c(C)ccnc13 | Reverse transcriptase |
| CHEMBL144420 | S=C(NCCc1ccccc1)Nc2nncs2 | Reverse transcriptase |
| CHEMBL75956 | Nc1nc(O)c2ncn(\C=C\3/CC3CO)c2n1 | Reverse transcriptase |
| CHEMBL144130 | Cn1cccc1CC\N=C(/S)\Nc2nccs2 | Reverse transcriptase |
| CHEMBL422001 | CCC(C)Sc1nc(O)cc(Cc2cccc(Cl)c2)n1 | Reverse transcriptase |
| CHEMBL144654 | COC(=O)c1cc(cc(Cl)c1OC)C(=CCCCCBr)c2cc(Cl)c(OC)c(c2)C(=O)OC | Reverse transcriptase |
| CHEMBL124838 | CCc1cc(NCc2oc3cccnc3n2)c(O)nc1C | Reverse transcriptase |
| CHEMBL151173 | CCOc1cccnc1N2CCN(CC2)C(=O)c3cc4cc(NS(=O)(=O)C)ccc4[nH]3 | Reverse transcriptase |
| CHEMBL96591 | FC(F)(F)C1(OC(=O)Nc2cc3ccccc3cc12)C#CC4CC4 | Reverse transcriptase |
| CHEMBL335037 | CCOC(=O)NCCC=C(c1cc(Cl)c(OC)c(c1)C(=O)OC)c2cc(Cl)c(OC)c(c2)C(=O)OC | Reverse transcriptase |
| CHEMBL2115318 | C[C@H]1O[C@@](C#CC2CC2)(c3cc(F)ccc3NC1=O)C(F)(F)F | Reverse transcriptase |
| CHEMBL125120 | CCc1cc(NCc2oc3ccccc3c2C)c(O)nc1C | Reverse transcriptase |
| CHEMBL321786 | CCc1cc(c(O)nc1C)C(OCc2c(F)cccc2OC)(C#CC3CC3)C(F)(F)F | Reverse transcriptase |
| CHEMBL94541 | CC(C)c1ccc2NC(=O)OC(C#CC3CC3)(c2c1)C(F)(F)F | Reverse transcriptase |
| CHEMBL155936 | OC1(CC1)C#CC2(OC(=O)Nc3ccc(Cl)cc23)C(F)(F)F | Reverse transcriptase |
| CHEMBL407128 | CC(=O)N[C@H]1C(=O)Nc2ccc(Cl)cc2[C@@]1(C#CC3CC3)C(F)(F)F | Reverse transcriptase |
| CHEMBL58348 | CC(=CCO[C@H]1C(=O)Nc2ccc(Cl)cc2[C@@]1(C#CC3CC3)C(F)(F)F)C | Reverse transcriptase |
| CHEMBL358754 | CC(C)Oc1cccnc1N(C)C2CCN(CC2)C(=O)c3cc4ccccc4[nH]3 | Reverse transcriptase |
| CHEMBL166126 | Clc1ccc(cc1)N(C(=S)OCCN2C(=O)c3ccccc3C2=O)C(=O)c4ccnc(Cl)c4 | Reverse transcriptase |
| CHEMBL94269 | Fc1cc2c(NC(=O)OC2(C#CC3CC3)C(F)(F)F)c(F)c1F | Reverse transcriptase |
| CHEMBL111492 | CC(=O)Nc1ccc2NC(=O)OC(C#CC3CC3)(c2c1)C(F)(F)F | Reverse transcriptase |
| CHEMBL212394 | COC(=O)CCC\C=C(/c1cc(C)c(OC)c(c1)C(=O)OC)\c2cc(C)c3ON(C)C(=O)c3c2 | Reverse transcriptase |
| CHEMBL379813 | COc1ccc(cc1OC2CCCC2)[C@@H]3CN(C(=O)C3)c4cccc(N)c4 | Reverse transcriptase |
| CHEMBL63515 | COC(=O)c1cc(cc(Cl)c1OC)C(=CCCN=[N+]=[N-])c2cc(Cl)c(OC)c(c2)C(=O)OC | Reverse transcriptase |
| CHEMBL316479 | FC(F)(F)C1(OC(=O)Nc2c(Cl)cc(Cl)cc12)C#CC3CC3 | Reverse transcriptase |
| CHEMBL111598 | CC(C)C#CC1(OC(=O)Nc2ccc(NC(=O)C)cc12)C(F)(F)F | Reverse transcriptase |
| CHEMBL293069 | FC(F)(F)[C@@]1(C#CC2CC2)[C@@H](NCc3ccccc3)C(=O)Nc4ccc(Cl)cc14 | Reverse transcriptase |
| CHEMBL57650 | O[C@H]1C(=O)Nc2ccc(Cl)cc2[C@@]1(C#CC3CC3)C(F)(F)F | Reverse transcriptase |
| CHEMBL36656 | CCNc1cccnc1N2CCN(CC2)C(=O)c3cc4cc(O)ccc4[nH]3 | Reverse transcriptase |
| CHEMBL49441 | CCN1c2ncccc2c3nccn3c4ccc(nc14)N5CCCC5 | Reverse transcriptase |
| CHEMBL94884 | FC(F)(F)C1(OC(=O)Nc2ccc(cc12)c3ccccc3)C#CC4CC4 | Reverse transcriptase |
| CHEMBL293536 | COC(=O)N[C@H]1C(=O)Nc2ccc(Cl)cc2[C@@]1(C#CC3CC3)C(F)(F)F | Reverse transcriptase |
| CHEMBL123514 | CCc1nc(CCCCCCC(=O)c2ccccc2)n3nc(Cl)ccc13 | Reverse transcriptase |
| CHEMBL23358 | Nc1ncnc2c1ncn2C3CC(OCP(=O)(O)OP(=O)(O)OP(=O)(O)OP(=O)(O)COC4CC(C=C4)n5cnc6c(N)ncnc56)C=C3 | Reverse transcriptase |
| CHEMBL151565 | CC(C)Nc1cccnc1N(C)C2CCN(CC2)C(=O)c3cc4ccccc4[nH]3 | Reverse transcriptase |
| CHEMBL754 | [Na+].[Na+].[Na+].[O-]C(=O)P(=O)([O-])[O-] | Reverse transcriptase |
| CHEMBL125873 | CCc1cc(NCc2cccc3ccccc23)c(O)nc1C | Reverse transcriptase |
| CHEMBL177174 | COCc1cc(CCc2oc3ccccc3n2)c(O)nc1C | Reverse transcriptase |
| CHEMBL440879 | CC(C)c1cccc(c1)C23SCCN2C(=O)c4ccccc34 | Reverse transcriptase |
| CHEMBL315287 | CC(c1ccccc1)c2cc(O)nc(SC3CCCC3)n2 | Reverse transcriptase |
| CHEMBL38744 | Cc1ccc(cc1)C(OCCn2c(C)ncc2[N+](=O)[O-])c3ccccc3 | Reverse transcriptase |
| CHEMBL71310 | CCN1c2ccc(OC)cc2N(C)C(=O)c3cccnc13 | Reverse transcriptase |
| CHEMBL356173 | CCOc1cccnc1N2CCN(CC2)C(=O)c3cc4ccccc4[nH]3 | Reverse transcriptase |
| CHEMBL66704 | CC1CN(CC2CC2)Cc3cc(Cl)cc4NC(=S)N1c34 | Reverse transcriptase |
| CHEMBL43324 | CCN1c2ncccc2c3nccn3c4ccc(nc14)N(C)C | Reverse transcriptase |
| CHEMBL312172 | O=C1N2CCCSC2(c3ccccc3)c4ccccc14 | Reverse transcriptase |
| CHEMBL299002 | CCCN1C(=S)c2ccccc2Oc3ccccc13 | Reverse transcriptase |
| CHEMBL322539 | CC(C)CSc1nc(O)c(C)c(Cc2ccccc2)n1 | Reverse transcriptase |
| CHEMBL145272 | O=C1Nc2c(Cn3cccn3)ccnc2N(C4CC4)c5ncccc15 | Reverse transcriptase |
| CHEMBL372715 | CN1N=C(N)c2cn([C@@H]3O[C@H](CO)[C@@H](O)[C@H]3O)c4nnnc1c24 | Reverse transcriptase |
| CHEMBL424141 | CCNc1cccnc1N(C)C2CCN(CC2)C(=O)c3cc4ccccc4[nH]3 | Reverse transcriptase |
| CHEMBL356247 | S=C(NCCc1c[nH]c2ccccc12)Nc3nccs3 | Reverse transcriptase |
| CHEMBL343149 | COC(=O)CC=C(c1cc(Cl)c(OC)c(c1)C(=O)OC)c2cc(Cl)c(OC)c(c2)C(=O)OC | Reverse transcriptase |
| CHEMBL36776 | CC(C)Nc1cccnc1N2CCN(CC2)C(=O)c3cc4ccc(OS(=O)(=O)C)cc4[nH]3 | Reverse transcriptase |
| CHEMBL85830 | CCc1cc(NCc2ccccc2C)c(O)nc1C | Reverse transcriptase |
| CHEMBL66043 | CC1C(C)N2C(=S)Nc3cccc(CN1CC=C(C)C)c23 | Reverse transcriptase |
| CHEMBL48427 | Cc1cccc(n1)N2C(SCC2=O)c3c(Cl)cccc3Cl | Reverse transcriptase |
| CHEMBL355113 | Clc1ccc(cc1)N(C(=S)OCCN2C(=O)c3ccccc3C2=O)C(=O)c4occc4 | Reverse transcriptase |
| CHEMBL7264 | CCNc1cccnc1N2CCN(CC2)C(=O)c3cc4cc(OC)ccc4[nH]3.CS(=O)(=O)O | Reverse transcriptase |
| CHEMBL43348 | Cc1ncc([N+](=O)[O-])n1CCOC(c2ccccc2)c3ccc(F)cc3 | Reverse transcriptase |
| CHEMBL35109 | O\C(=C/C(=O)c1nn[nH]n1)\c2c[nH]c3ccc(Cl)cc23 | Reverse transcriptase |
| CHEMBL85368 | CC(C)Sc1nc(O)cc(n1)C(C)c2ccccc2 | Reverse transcriptase |
| CHEMBL87569 | CCC(c1ccccc1)c2cc(O)nc(SC(C)C)n2 | Reverse transcriptase |
| CHEMBL207575 | COC(=O)C1=C([C@H]2c3ccccc3S[C@@]12C(=O)OC)N4CCC[C@@H]4C | Reverse transcriptase |
| CHEMBL435216 | Cc1nc(CCCCCOCc2ccccc2)n3nc(Cl)ccc13 | Reverse transcriptase |
| CHEMBL1309 | Oc1cc(Cl)cc2c1NC(=O)O[C@]2(C#CC3CC3)C(F)(F)F | Reverse transcriptase |
| CHEMBL125052 | CCc1cc(NCc2oc(cn2)c3ccccc3)c(O)nc1C | Reverse transcriptase |
| CHEMBL132489 | COC(=O)NCCC=C(c1cc(Br)c(OC)c(c1)C(=O)OC)c2cc(Br)c(OC)c(c2)C(=O)OC | Reverse transcriptase |
| CHEMBL3143796 | CNC(=O)CCCCCN1C(=O)N(C=C(C)C1=O)[C@@H]2O[C@H](CO[Si](C)(C)C(C)(C)C)[C@@]3(OS(=O)(=O)C=C3N)[C@H]2O[Si](C)(C)C(C)(C)C | Reverse transcriptase |
| CHEMBL151315 | Oc1cc(O)c2CC(COc2c1)OC(=O)c3cc(O)c(O)c(O)c3 | Reverse transcriptase |
| CHEMBL101348 | Clc1cnc(NC(=S)NCCc2ccccc2)s1 | Reverse transcriptase |
| CHEMBL212867 | CC1=CN([C@@H]2O[C@H](CO[Si](C)(C)C(C)(C)C)[C@@]3(OS(=O)(=O)C=C3N)[C@H]2O[Si](C)(C)C(C)(C)C)C(=O)N(CC4CO4)C1=O | Reverse transcriptase |
| CHEMBL342794 | Cc1cccc(OCc2ccnc3N(C4CC4)c5ncccc5C(=O)Nc23)c1 | Reverse transcriptase |
| CHEMBL65800 | CCCN1CCN2C(=S)Nc3cccc(C1C)c23 | Reverse transcriptase |
| CHEMBL173529 | CCc1cc(CCc2ccc3ccccc3n2)c(O)nc1C | Reverse transcriptase |
| CHEMBL346356 | Oc1cc2NC(=O)OC(C#CC3CC3)(c2cc1Cl)C(F)(F)F | Reverse transcriptase |
| CHEMBL197069 | CC(C)C(=O)c1c(O)c(CC=C(C)C)c2OC(=O)C=C(c3ccccc3)c2c1O | Reverse transcriptase |
| CHEMBL254840 | CCCCc1csc(NC(=S)NCCc2ccccc2)n1 | Reverse transcriptase |
| CHEMBL415871 | COC(=O)Nc1ccc2[nH]c(cc2c1)C(=O)N3CCN(CC3)c4ncccc4NC(C)C | Reverse transcriptase |
| CHEMBL109551 | CC(C)Sc1nc(O)c(C)c(Cc2cccc(C)c2)n1 | Reverse transcriptase |
| CHEMBL123202 | Cc1nc(CCCCCCC(=O)c2ccccc2)n3nc(ccc13)n4ccnc4C | Reverse transcriptase |
| CHEMBL110007 | CSc1nc(O)c(C)c(Cc2cccc(C)c2)n1 | Reverse transcriptase |
| CHEMBL301449 | CC(C)c1ccc(cc1)S(=O)(=O)n2ccc(c2)C(O)c3ccc(Cl)cc3Cl | Reverse transcriptase |
| CHEMBL350396 | Clc1ccc(cc1)N(C(=S)OCCN2C(=O)c3ccccc3C2=O)C(=O)c4cccs4 | Reverse transcriptase |
| CHEMBL338826 | CCc1cc(NCc2nc(O)c3ccccc3n2)c(O)nc1C | Reverse transcriptase |
| CHEMBL110857 | CC(C)Sc1nc(O)c(C)c(Cc2ccccc2)n1 | Reverse transcriptase |
| CHEMBL31455 | FC(F)(F)C1(OCC(=O)Nc2ccc(Cl)cc12)C#Cc3cocc3 | Reverse transcriptase |
| CHEMBL110008 | Cc1cccc(Cc2cc(O)nc(SC3CCCCC3)n2)c1 | Reverse transcriptase |
| CHEMBL147336 | Oc1cc(cc(O)c1O)C(=O)OC2COc3ccccc3C2 | Reverse transcriptase |
| CHEMBL110009 | Cc1cccc(Cc2cc(O)nc(SC3CCCC3)n2)c1 | Reverse transcriptase |
| CHEMBL85949 | CC(c1cc(O)nc(SC2CCCCC2)n1)c3cccc4ccccc34 | Reverse transcriptase |
| CHEMBL126216 | Cc1nc(CCCCCCC(=O)c2ccccc2)n3nc(ccc13)n4cnnc4 | Reverse transcriptase |
| CHEMBL88459 | OC(=O)c1cc(Cl)ccc1NC(=O)CCCCCC(=O)Nc2ccc(Cl)cc2C(=O)O | Reverse transcriptase |
| CHEMBL85312 | O=C1N2CCSC2(c3cccs3)c4ccccc14 | Reverse transcriptase |
| CHEMBL292464 | COc1ccccc1Sc2cccc(N)c2C#N | Reverse transcriptase |
| CHEMBL105826 | CCc1cc(c(O)nc1C)C(OCc2ccc(Cl)cc2Cl)(C#CC3CC3)C(F)(F)F | Reverse transcriptase |
| CHEMBL124328 | CCc1cc(NCc2oc3ccc4ccccc4c3n2)c(O)nc1C | Reverse transcriptase |
| CHEMBL43283 | CCN1c2ncccc2c3ncc(C)n3c4ccc(nc14)N5CCC=CC5 | Reverse transcriptase |
| CHEMBL151192 | CCNc1cccnc1N2CCN(CC2)C(=O)c3cc4cc(NS(=O)(=O)C)ccc4[nH]3 | Reverse transcriptase |
| CHEMBL32469 | CCC(C)Sc1nc(O)c(C)c(Cc2cccc(C)c2)n1 | Reverse transcriptase |
| CHEMBL297115 | CCN1c2ncccc2c3nc(C)cn3c4cccnc14 | Reverse transcriptase |
| CHEMBL386338 | COC(=O)CCC\C=C(\c1cccc(c1)C#N)/c2cc(C)c3OC(=O)N(C)c3c2 | Reverse transcriptase |
| CHEMBL50019 | Cc1ccnc(c1)N2C(SCC2=O)c3c(F)cccc3F | Reverse transcriptase |
| CHEMBL359315 | Cc1cccc(n1)N2C(SCC2=O)c3c(C)cccc3F | Reverse transcriptase |
| CHEMBL124946 | Cc1cc(C)c(NCc2oc3c(Cl)ccc(Cl)c3n2)c(O)n1 | Reverse transcriptase |
| CHEMBL393003 | Fc1cccc(F)c1CN2C(=O)Nc3ccc(Cl)cc23 | Reverse transcriptase |
| CHEMBL37722 | CN1C(=O)c2cccnc2N(CCF)c3ncccc13 | Reverse transcriptase |
| CHEMBL3143764 | COCCN1C(=O)N(C=C(C)C1=O)[C@@H]2O[C@H](CO[Si](C)(C)C(C)(C)C)[C@@]3(OS(=O)(=O)C=C3N)[C@H]2O[Si](C)(C)C(C)(C)C | Reverse transcriptase |
| CHEMBL306079 | CCN1c2ccccc2N(C)C(=O)c3ccc(F)nc13 | Reverse transcriptase |
| CHEMBL111368 | CC(C)Sc1nc(O)cc(Cc2cccc(C)c2)n1 | Reverse transcriptase |
| CHEMBL296589 | Cc1cccc(n1)N2C(SCC2=O)c3c(F)cccc3F | Reverse transcriptase |
| CHEMBL133393 | COC(=O)c1cc(cc(C)c1OC)C(=CCCNC(=O)OCC(C)C)c2cc(C)c(OC)c(c2)C(=O)OC | Reverse transcriptase |
| CHEMBL3143760 | COC(=O)CN1C(=O)N(C=C(C)C1=O)[C@@H]2O[C@H](CO[Si](C)(C)C(C)(C)C)[C@@]3(OS(=O)(=O)C=C3N)[C@H]2O[Si](C)(C)C(C)(C)C | Reverse transcriptase |
| CHEMBL156109 | CSc1nc(O)cc(Cc2c(Cl)cccc2Cl)n1 | Reverse transcriptase |
| CHEMBL39343 | OCc1ccnc2N(C3CC3)c4ncccc4C(=O)Nc12 | Reverse transcriptase |
| CHEMBL338823 | Cc1nc(CCCCCCC(=O)c2ccccc2)n3nc(N)ccc13 | Reverse transcriptase |
| CHEMBL114292 | Oc1cc(Cc2ccccc2)nc(SC3CCCCC3)n1 | Reverse transcriptase |
| CHEMBL126811 | Cc1nc(CCCCCCCC(=O)c2ccccc2)n3nc(Cl)ccc13 | Reverse transcriptase |
| CHEMBL345252 | CC(=O)NC1=CC(=O)c2ccc(nc2C1=O)c3[nH]c(cc4c5ccccc5nc34)C(=O)OCCOP(=O)(O)O | Reverse transcriptase |
| CHEMBL176587 | CCc1cc(\C=C/c2oc3ccccc3n2)c(O)nc1C | Reverse transcriptase |
| CHEMBL144476 | Clc1ccccc1OCc2ccnc3N(C4CC4)c5ncccc5C(=O)Nc23 | Reverse transcriptase |
| CHEMBL3143792 | CNC(=O)CN1C(=O)N(C=C(C)C1=O)[C@@H]2O[C@H](CO[Si](C)(C)C(C)(C)C)[C@@]3(OS(=O)(=O)C=C3N)[C@H]2O[Si](C)(C)C(C)(C)C | Reverse transcriptase |
| CHEMBL3143768 | CC1=CN([C@@H]2O[C@H](CO[Si](C)(C)C(C)(C)C)[C@@]3(OS(=O)(=O)C=C3N)[C@H]2O[Si](C)(C)C(C)(C)C)C(=O)N(CCO)C1=O | Reverse transcriptase |
| CHEMBL338435 | CN(C1CCN(CC1)C(=O)c2cc3cc(NS(=O)(=O)C)ccc3[nH]2)c4ncccc4NC(C)(C)C | Reverse transcriptase |
| CHEMBL379794 | C[C@H](NC(=O)CN1C(=O)N(C=C(C)C1=O)[C@@H]2O[C@H](CO[Si](C)(C)C(C)(C)C)[C@@]3(OS(=O)(=O)C=C3N)[C@H]2O[Si](C)(C)C(C)(C)C)C(=O)O | Reverse transcriptase |
| CHEMBL63497 | CCCCCC=C(c1ccc(OC)c(c1)C(=O)OC)c2ccc(OC)c(c2)C(=O)OC | Reverse transcriptase |
| CHEMBL144209 | COC(=O)c1cc(cc(Cl)c1OC)C(=CCC2OCCO2)c3cc(Cl)c(OC)c(c3)C(=O)OC | Reverse transcriptase |
| CHEMBL379599 | CC1=CN([C@@H]2O[C@H](CO[Si](C)(C)C(C)(C)C)[C@@]3(OS(=O)(=O)C=C3N)[C@H]2O[Si](C)(C)C(C)(C)C)C(=O)N(CC(=O)O)C1=O | Reverse transcriptase |
| CHEMBL433258 | CCN1c2ncccc2c3ncc(C)n3c4ccc(O)nc14 | Reverse transcriptase |
| CHEMBL147224 | CCOc1cccnc1N(C)C2CCN(CC2)C(=O)c3cc4ccccc4[nH]3 | Reverse transcriptase |
| CHEMBL145842 | CCC1(CCCC=C(c2cc(Cl)c(OC)c(CO)c2)c3cc(Cl)c(OC)c(CO)c3)OCCO1 | Reverse transcriptase |
| CHEMBL1161627 | CC1=CN([C@H]2C[C@H](O)[C@@](COP(=O)(O)OP(=O)(O)OP(=O)(O)O)(O2)C#C)C(=O)NC1=O | Reverse transcriptase |
| CHEMBL305840 | CCN1C(=O)c2ccccc2N(C)c3nc(Cl)ccc13 | Reverse transcriptase |
| CHEMBL32460 | FC(F)(F)C1(OCC(=O)Nc2ccc(Cl)cc12)C#Cc3ccccc3 | Reverse transcriptase |
| CHEMBL66404 | CC1Cn2c(O)nc3cccc(CN1CC=C(C)C)c23 | Reverse transcriptase |
| CHEMBL124271 | CNc1ccc2c(C)nc(CCCCCCC(=O)c3ccccc3)n2n1 | Reverse transcriptase |
| CHEMBL338302 | Cc1nc(CCCCCCC(=O)c2ccccc2)n3nc(ccc13)n4cccn4 | Reverse transcriptase |
| CHEMBL33238 | Cc1cccc(Cc2nc(SC3CCCC3)nc(O)c2C)c1 | Reverse transcriptase |
| CHEMBL151316 | CCOc1cccnc1N(C)C2CCN(CC2)C(=O)c3cc4cc(NS(=O)(=O)C)ccc4[nH]3 | Reverse transcriptase |
| CHEMBL367204 | Cc1nc(O)c(CCc2oc3ccccc3n2)cc1CO | Reverse transcriptase |
| CHEMBL209842 | CCC1=CN([C@@H]2O[C@H](CO[Si](C)(C)C(C)(C)C)[C@@]3(OS(=O)(=O)C=C3N)[C@H]2O[Si](C)(C)C(C)(C)C)C(=O)NC1=O | Reverse transcriptase |
| CHEMBL84486 | O=C1N2CCSC2(c3ccccc3)c4ncccc14 | Reverse transcriptase |
| CHEMBL54698 | Nc1cccc(Sc2cc(Cl)ccc2Cl)c1C#N | Reverse transcriptase |
| CHEMBL313676 | S=C1N2CCSC2(c3ccccc3)c4ccccc14 | Reverse transcriptase |
| CHEMBL212649 | CC1=CN([C@@H]2O[C@H](CO[Si](C)(C)C(C)(C)C)[C@@]3(OS(=O)(=O)C(=C3N)I)[C@H]2O[Si](C)(C)C(C)(C)C)C(=O)NC1=O | Reverse transcriptase |
| CHEMBL3143793 | CN(C)C(=O)CN1C(=O)N(C=C(C)C1=O)[C@@H]2O[C@H](CO[Si](C)(C)C(C)(C)C)[C@@]3(OS(=O)(=O)C=C3N)[C@H]2O[Si](C)(C)C(C)(C)C | Reverse transcriptase |
| CHEMBL63150 | COC(=O)c1cc(cc(Cl)c1OC)C(=CCCCCO)c2cc(Cl)c(OC)c(c2)C(=O)OC | Reverse transcriptase |
| CHEMBL367508 | Nc1ccc([C@@H]2CS[C@H](OP(=O)(O)OP(=O)(O)OP(=O)(O)O)O2)c(O)n1 | Reverse transcriptase |
| CHEMBL315933 | COc1ccc2C(=O)N3CCSC3(c4ccccc4)c2c1 | Reverse transcriptase |
| CHEMBL82439 | CCc1cc(CCN2C(=O)c3ccccc3C2=O)c(O)nc1C | Reverse transcriptase |
| CHEMBL3142918 | CC1=CN([C@@H]2O[C@H](CO[Si](C)(C)C(C)(C)C)[C@@]3(OS(=O)(=O)C=C3N)[C@H]2O[Si](C)(C)C(C)(C)C)C(=O)N(CCCOP(=O)(O)C(=O)O)C1=O | Reverse transcriptase |
| CHEMBL1788125 | [Na+].CCCCCCCCCCCCCCCC(=O)Nc1cc(cc2cc(cc(O)c12)S(=O)(=O)[O-])S(=O)(=O)O | Reverse transcriptase |
| CHEMBL340286 | Oc1nc2CCCCc2cc1NCN3C(=O)c4ccccc4C3=O | Reverse transcriptase |
| CHEMBL86062 | CCC(c1ccccc1)c2cc(O)nc(SC3CCCCC3)n2 | Reverse transcriptase |
| CHEMBL313518 | OC(=O)c1cc(Br)ccc1NC(=O)CCCCCC(=O)Nc2ccc(Br)cc2C(=O)O | Reverse transcriptase |
| CHEMBL84821 | CCc1cc(NCc2cccc(C)c2OC)c(O)nc1C | Reverse transcriptase |
| CHEMBL263515 | CC(C)[C@H](N)C(=O)NCC(=O)NCCCN1C(=O)N(C=C(C)C1=O)C2OC(CO[Si](C)(C)C(C)(C)C)C3(OS(=O)(=O)C=C3N)C2O[Si](C)(C)C(C)(C)C | Reverse transcriptase |
| CHEMBL212868 | CC1=CN([C@@H]2O[C@H](CO[Si](C)(C)C(C)(C)C)[C@@]3(OS(=O)(=O)C=C3N=C(N)N)[C@H]2O[Si](C)(C)C(C)(C)C)C(=O)NC1=O | Reverse transcriptase |
| CHEMBL3142919 | CC1=CN([C@@H]2O[C@H](CO[Si](C)(C)C(C)(C)C)[C@@]3(OS(=O)(=O)C=C3N)[C@H]2O[Si](C)(C)C(C)(C)C)C(=O)N(CCCOP(=O)(O)C(=O)OCc4ccccc4)C1=O | Reverse transcriptase |
| CHEMBL328046 | Oc1cc(O)c2CC(COc2c1)OC(=O)c3ccc(O)c(O)c3O | Reverse transcriptase |
| CHEMBL107899 | CCc1cc(c(O)nc1C)C(OCc2cc(OC)cc(OC)c2)(C#CC3CC3)C(F)(F)F | Reverse transcriptase |
| CHEMBL332351 | Cc1nc(CCCCCC(=O)Cc2ccccc2)n3nc(Cl)ccc13 | Reverse transcriptase |
| CHEMBL431931 | CCN1c2ncccc2c3nccn3c4ccc(nc14)N5CCC=CC5 | Reverse transcriptase |
| CHEMBL38088 | CC(C)Nc1cccnc1N2CCN(CC2)C(=O)c3cc4ccc(cc4[nH]3)C#N | Reverse transcriptase |
| CHEMBL149100 | Cc1ccc(CC\N=C(/S)\Nc2nccs2)cc1 | Reverse transcriptase |
| CHEMBL320902 | S=C(NCCc1ccccc1)Nc2cnccn2 | Reverse transcriptase |
| CHEMBL7483 | CCNc1cccnc1N2CCN(CC2)C(=O)c3cc4ccccc4[nH]3 | Reverse transcriptase |
| CHEMBL84260 | Clc1ccc(cc1)C23SCCN2C(=O)c4ccccc34 | Reverse transcriptase |
| CHEMBL213257 | CC1=CN(C2OC(CO[Si](C)(C)C(C)(C)C)C3(OS(=O)(=O)C=C3N)C2O[Si](C)(C)C(C)(C)C)C(=O)N(CCCNC(=O)[C@@H]4CCCN4C(=O)[C@@H](N)CC(=O)N)C1=O | Reverse transcriptase |
| CHEMBL140048 | Cc1cccc(Sc2c(C)cnc(O)c2N)c1 | Reverse transcriptase |
| CHEMBL341880 | CC(=O)Nc1c(O)ncc(C)c1Sc2cc(C)cc(C)c2 | Reverse transcriptase |
| CHEMBL344562 | CCN1c2nc(OC)cc(COc3ccccc3)c2NC(=O)c4cccnc14 | Reverse transcriptase |
| CHEMBL151411 | CCOC(=O)c1cccn1S(=O)(=O)c2cc(C)ccc2N | Reverse transcriptase |
| CHEMBL126931 | CCC(=O)Nc1c(O)ncc(C)c1Sc2cc(C)cc(C)c2 | Reverse transcriptase |
| CHEMBL314054 | COc1ccc2c(c1)C(=O)N3CCSC23c4ccccc4 | Reverse transcriptase |
| CHEMBL82751 | Clc1ccc(cc1Cl)C23SCCN2C(=O)c4ccccc34 | Reverse transcriptase |
| CHEMBL92810 | FC(F)(F)c1ccc(\N=C(/S)\NCCC2=CCCCC2)nc1 | Reverse transcriptase |
| CHEMBL88591 | OC(=O)c1cc(I)ccc1NC(=O)CCCCC(=O)Nc2ccc(I)cc2C(=O)O | Reverse transcriptase |
| CHEMBL85783 | Cc1cccc(n1)C23SCCN2C(=O)c4ccccc34 | Reverse transcriptase |
| CHEMBL150025 | Cc1cc(C)nc(c1)N2C(SCC2=O)c3c(F)cccc3F | Reverse transcriptase |
| CHEMBL151152 | Cc1ccnc(c1)N2C(SCC2=O)c3c(F)cccc3Cl | Reverse transcriptase |
| CHEMBL343398 | COC(=O)c1cc(cc(Cl)c1OC)C(=CCCCC=O)c2cc(Cl)c(OC)c(c2)C(=O)OC | Reverse transcriptase |
| CHEMBL58711 | C[C@H]1CN2C(=S)Nc3ccc(Cl)c(CN1CC=C(C)C)c23 | Reverse transcriptase |
| CHEMBL417331 | CC(C)Nc1cccnc1N2CCN(CC2)C(=O)c3cc4cc(OCc5ccccc5)ccc4[nH]3 | Reverse transcriptase |
| CHEMBL88463 | COc1ccc(NC(=O)CCCCC(=O)Nc2ccc(OC)cc2C(=O)O)c(c1)C(=O)O | Reverse transcriptase |
| CHEMBL320735 | CCC(C)SC1=N\C(=C\c2ccccc2)\C(C)(C)C(=O)N1 | Reverse transcriptase |
| CHEMBL109637 | Cc1c(O)nc(SC2CCCCC2)nc1Cc3ccccc3 | Reverse transcriptase |
| CHEMBL108944 | CC(C)SC1=N\C(=C\c2ccccc2)\C(C)(C)C(=O)N1 | Reverse transcriptase |
| CHEMBL125867 | CCc1cc(NCc2cc3ccccc3[nH]2)c(O)nc1C | Reverse transcriptase |
| CHEMBL380100 | CC1=CN([C@@H]2O[C@H](CO[Si](C)(C)C(C)(C)C)[C@@]3(OS(=O)(=O)C=C3N)[C@H]2O[Si](C)(C)C(C)(C)C)C(=O)N(CCCN)C1=O | Reverse transcriptase |
| CHEMBL286325 | CC(C)Nc1cccnc1N2CCN(CC2)C(=O)c3cc4ccc(F)cc4[nH]3 | Reverse transcriptase |
| CHEMBL212339 | CC(C)[C@H](N)C(=O)N1CCC[C@H]1C(=O)N[C@@H](C(C)C)C(=O)NCCCN2C(=O)N(C=C(C)C2=O)C3OC(CO[Si](C)(C)C(C)(C)C)C4(OS(=O)(=O)C=C4N)C3O[Si](C)(C)C(C)(C)C | Reverse transcriptase |
| CHEMBL345996 | S=C(NCCOc1ccccc1)Nc2nccs2 | Reverse transcriptase |
| CHEMBL133678 | COC(=O)NCCC=C(c1cc(Cl)c(OC)c(c1)C(=O)OC)c2cc(Cl)c(OC)c(c2)C(=O)OC | Reverse transcriptase |
| CHEMBL54444 | Cc1ccccc1S(=O)(=O)c2cccc(N)c2C#N | Reverse transcriptase |
| CHEMBL82461 | Clc1cc2C(=O)N3CCSC3(c4ccccc4)c2cc1Cl | Reverse transcriptase |
| CHEMBL107562 | COC(=O)CSc1nc(O)c(C(C)C)c(Cc2cccc3ccccc23)n1 | Reverse transcriptase |
| CHEMBL3143798 | CC1=CN([C@@H]2O[C@H](CO[Si](C)(C)C(C)(C)C)[C@@]3(OS(=O)(=O)C=C3N)[C@H]2O[Si](C)(C)C(C)(C)C)C(=O)N(C\C=C\CO)C1=O | Reverse transcriptase |
| CHEMBL70324 | CCN1c2cc(N)ccc2N(C)C(=O)c3cccnc13 | Reverse transcriptase |
| CHEMBL145260 | S=C(NCCCc1ccccc1)Nc2nccs2 | Reverse transcriptase |
| CHEMBL85758 | Nc1ccc2C(=O)N3CCSC3(c4ccccc4)c2c1 | Reverse transcriptase |
| CHEMBL147069 | Oc1ccc(CCOC(=O)c2cc(O)c(O)c(O)c2)cc1 | Reverse transcriptase |
| CHEMBL85476 | CCc1cc(NCc2ccccc2O)c(O)nc1C | Reverse transcriptase |
| CHEMBL82166 | CC1C(=O)NC(=O)N(C2C[C@@H](N=[N+]=[N-])[C@@H](COCP(=O)(O)OP(=O)(O)OP(=O)(O)O)O2)C1=C | Reverse transcriptase |
| CHEMBL378037 | CC(C)C[C@H](NC(=O)[C@@H](N)C(C)C)C(=O)NCCCN1C(=O)N(C=C(C)C1=O)C2OC(CO[Si](C)(C)C(C)(C)C)C3(OS(=O)(=O)C=C3N)C2O[Si](C)(C)C(C)(C)C | Reverse transcriptase |
| CHEMBL86268 | Nc1cccc(c1)C23SCCN2C(=O)c4ccccc34 | Reverse transcriptase |
| CHEMBL314103 | Cc1ccnc(c1)C23SCCN2C(=O)c4cccnc34 | Reverse transcriptase |
| CHEMBL111088 | CC(C)CSC1=N\C(=C\c2ccccc2)\C(C)(C)C(=O)N1 | Reverse transcriptase |
| CHEMBL111118 | CC(C)CSc1nc(O)c(C)c(Cc2cccc(C)c2)n1 | Reverse transcriptase |
| CHEMBL58865 | CC(C)C#CC1(NS(=O)(=O)Nc2ccc(Cl)cc12)C3CC3 | Reverse transcriptase |
| CHEMBL380211 | CC1=CN(C2OC(CO[Si](C)(C)C(C)(C)C)C3(OS(=O)(=O)C=C3N)C2O[Si](C)(C)C(C)(C)C)C(=O)N(CCCNC(=O)[C@@H]4CCCN4C(=O)[C@@H](N)CCCCN)C1=O | Reverse transcriptase |
| CHEMBL294602 | Cc1ccc(cc1)C(O)c2ccn(c2)S(=O)(=O)c3ccc(C)cc3 | Reverse transcriptase |
| CHEMBL3142917 | [Na+].COC(=O)P(=O)([O-])OCCCN1C(=O)N(C=C(C)C1=O)[C@@H]2O[C@H](CO[Si](C)(C)C(C)(C)C)[C@@]3(OS(=O)(=O)C=C3N)[C@H]2O[Si](C)(C)C(C)(C)C | Reverse transcriptase |
| CHEMBL110328 | CCc1c(O)nc(SCC(=O)OC)nc1Cc2cccc3ccccc23 | Reverse transcriptase |
| CHEMBL55998 | Nc1cccc([S+]([O-])c2cccc(Br)c2)c1C#N | Reverse transcriptase |
| CHEMBL124246 | Cc1nc(CCCCCC(=O)c2ccccc2)n3nc(Cl)ccc13 | Reverse transcriptase |
| CHEMBL214795 | CC(C)[C@H](N)C(=O)N1C[C@H](O)[C@H](O)[C@H]1C(=O)NCCCN2C(=O)N(C=C(C)C2=O)C3OC(CO[Si](C)(C)C(C)(C)C)C4(OS(=O)(=O)C=C4N)C3O[Si](C)(C)C(C)(C)C | Reverse transcriptase |
| CHEMBL345128 | CCC(C)Sc1nc(O)cc(Cc2ccc(Cl)cc2)n1 | Reverse transcriptase |
| CHEMBL35655 | CSCSc1nc(O)c(C(C)C)c(Cc2cc(C)cc(C)c2)n1 | Reverse transcriptase |
| CHEMBL148532 | CC(C)(C)Nc1cccnc1N2CCN(CC2)C(=O)c3cc4cc(NS(=O)(=O)C)ccc4[nH]3 | Reverse transcriptase |
| CHEMBL379255 | CC1=CN([C@@H]2O[C@H](CO[Si](C)(C)C(C)(C)C)[C@@]3(OS(=O)(=O)C(=C3N)\C=C\CO)[C@H]2O[Si](C)(C)C(C)(C)C)C(=O)NC1=O | Reverse transcriptase |
| CHEMBL109721 | CSc1nc(O)c(C)c(Cc2ccccc2)n1 | Reverse transcriptase |
| CHEMBL432812 | Brc1ccc(NC(=S)NCCN2CCCC2)nc1 | Reverse transcriptase |
| CHEMBL347244 | Cc1c(O)nc(SC2CCCCC2)nc1Cc3c(Cl)cccc3Cl | Reverse transcriptase |
| CHEMBL130316 | COC(=O)NCCC=C(c1cc(C)c(OC)c(c1)C(=O)OC)c2cc(C)c(OC)c(c2)C(=O)OC | Reverse transcriptase |
| CHEMBL295567 | CCS(=O)(=O)N1c2ccccc2NC(=O)C1(C#CC3CC3)C(F)(F)F | Reverse transcriptase |
| CHEMBL48735 | FC(F)(F)C1(C#CC2CC2)N(C(=O)c3ccccc3)c4ccccc4NC1=O | Reverse transcriptase |
| CHEMBL327832 | CCC1(CC=C)Oc2ccccc2n3cccc3C1=O | Reverse transcriptase |
| CHEMBL53684 | Nc1cccc(c1C#N)S(=O)(=O)c2ccccc2F | Reverse transcriptase |
| CHEMBL205041 | Cc1cc(C)c(c(C)c1)n2nnnc2SCC(=O)Nc3ccc(cc3)[N+](=O)[O-] | Reverse transcriptase |
| CHEMBL48255 | CC(C)S(=O)(=O)N1c2ccccc2NC(=O)C1(C#CC3CC3)C(F)(F)F | Reverse transcriptase |
| CHEMBL316094 | CC1(Sc2ccccc2n3cccc3C1=O)c4ccccc4 | Reverse transcriptase |
| CHEMBL299788 | CCCS(=O)(=O)N1c2ccccc2NC(=O)C1(C#CC3CC3)C(F)(F)F | Reverse transcriptase |
| CHEMBL151077 | CCOC(=O)c1cccn1S(=O)(=O)c2cc(C)ccc2[N+](=O)[O-] | Reverse transcriptase |
| CHEMBL205094 | Cc1cc(C)c(c(C)c1)n2nnnc2SCC(=O)Nc3c(Cl)cccc3Cl | Reverse transcriptase |
| CHEMBL31871 | CC1=C(Sc2ccccc2)N(COCCO)C(=O)NC1=O | Reverse transcriptase |
| CHEMBL377493 | COC(=O)C1=C(C2c3ccccc3SC12C(=O)OC)N4CC=CC4 | Reverse transcriptase |
| CHEMBL370921 | Oc1nc2ccc(Cl)cc2n1Cc3ccc4ccccc4c3 | Reverse transcriptase |
| CHEMBL378474 | CC1=CN([C@@H]2O[C@H](CO[Si](C)(C)C(C)(C)C)[C@@]3(OS(=O)(=O)C=C3N)[C@H]2O[Si](C)(C)C(C)(C)C)C(=O)N(CCCN(CC(=O)O)CC(=O)O)C1=O | Reverse transcriptase |
| CHEMBL432894 | Nc1cccc(c1C#N)S(=O)(=O)c2cccc(c2)C(F)(F)F | Reverse transcriptase |
| CHEMBL86064 | CCc1cc(NCc2ccccc2)c(O)nc1C | Reverse transcriptase |
| CHEMBL1460 | OC[C@@H]1CC[C@@H](O1)n2cnc3C(=O)NC=Nc23 | Reverse transcriptase |
| CHEMBL172773 | CCc1cc(CCC(=O)Nc2ccccc2O)c(O)nc1C | Reverse transcriptase |
| CHEMBL379337 | COC(=O)C(=O)NC1=CS(=O)(=O)O[C@@]12[C@@H](COCc3ccccc3)O[C@H]([C@@H]2O[Si](C)(C)C(C)(C)C)N4C=C(C)C(=O)N(C)C4=O | Reverse transcriptase |
| CHEMBL383894 | CC(C)[C@H](N)C(=O)N[C@@H](C)C(=O)NCCCN1C(=O)N(C=C(C)C1=O)C2OC(CO[Si](C)(C)C(C)(C)C)C3(OS(=O)(=O)C=C3N)C2O[Si](C)(C)C(C)(C)C | Reverse transcriptase |
| CHEMBL89319 | OC(=O)c1cc(Br)ccc1NC(=O)c2ccc(nc2)C(=O)Nc3ccc(Br)cc3C(=O)O | Reverse transcriptase |
| CHEMBL37888 | CC(C)Nc1cccnc1N2CCN(CC2)C(=O)c3cc4cc(OS(=O)(=O)C)ccc4[nH]3 | Reverse transcriptase |
| CHEMBL34542 | CC(C)Nc1cccnc1N2CCN(CC2)C(=O)c3cc4cc(ccc4[nH]3)N(C)S(=O)(=O)C | Reverse transcriptase |
| CHEMBL40642 | CSCSc1cc(Cc2ccccc2)c(C(C)C)c(O)n1 | Reverse transcriptase |
| CHEMBL63982 | CCCCCC1OC1(c2cc(Br)c(OC)c(c2)C(=O)OC)c3cc(Br)c(OC)c(c3)C(=O)OC | Reverse transcriptase |
| CHEMBL44392 | CCN1c2ncccc2c3nc(CC)c(C)n3c4cccnc14 | Reverse transcriptase |
| CHEMBL130336 | COC(=O)c1cc(cc(C)c1OC)C(=CCCCC(=O)N(C)C)c2cc(C)c(OC)c(c2)C(=O)OC | Reverse transcriptase |
| CHEMBL198201 | COC(=O)CCC\C=C(/c1cccc(c1)C#N)\c2cc(C)c3onc(OC)c3c2 | Reverse transcriptase |
| CHEMBL51912 | Cc1ccc(Sc2cccc(N)c2C#N)cc1 | Reverse transcriptase |
| CHEMBL153896 | CCCCOC(=O)c1cc2c3ccccc3[nH]c2c(n1)c4ccc5C(=O)C=C(NC(=O)CCC)C(=O)c5n4 | Reverse transcriptase |
| CHEMBL288626 | Brc1ccc(NC(=S)NCN2CCCCC2)nc1 | Reverse transcriptase |
| CHEMBL58119 | C[C@H]1Cn2c(S)nc3ccc(F)c(CN1CC=C(C)C)c23 | Reverse transcriptase |
| CHEMBL86289 | CC(C)Sc1nc(O)cc(n1)C(C)c2cccc3ccccc23 | Reverse transcriptase |
| CHEMBL314558 | O=C1N2CCSC2(c3ccccn3)c4ccccc14 | Reverse transcriptase |
| CHEMBL301086 | Nc1cccc(c1C#N)S(=O)(=O)c2ccccc2Cl | Reverse transcriptase |
| CHEMBL175037 | CCc1cc(\C=C\c2oc3ccccc3n2)c(O)nc1C | Reverse transcriptase |
| CHEMBL298823 | Nc1cccc(c1C#N)S(=O)(=O)c2ccccc2C#N | Reverse transcriptase |
| CHEMBL325757 | COC(=O)c1cc(cc(Br)c1OC)C(=CCCCC#C)c2cc(Br)c(OC)c(c2)C(=O)OC | Reverse transcriptase |
| CHEMBL104738 | CCc1cc(c(O)nc1C)C(OCc2c(F)cccc2Cl)(C#CC3CC3)C(F)(F)F | Reverse transcriptase |
| CHEMBL313729 | O=C1N2CCOC2(c3ccccc3)c4ccccc14 | Reverse transcriptase |
| CHEMBL111523 | CSC1=N\C(=C\c2ccccc2)\C(C)(C)C(=O)N1 | Reverse transcriptase |
| CHEMBL35755 | CSCSc1nc(O)c(C(C)C)c(Cc2ccccc2)n1 | Reverse transcriptase |
| CHEMBL292012 | Nc1cccc([S+]([O-])c2cc(Cl)ccc2Cl)c1C#N | Reverse transcriptase |
| CHEMBL125615 | CCC1=C(C)NC(OC)C(=C1)CCN2C(=O)c3ccccc3C2=O | Reverse transcriptase |
| CHEMBL85280 | Cc1ccccc1C23SCCN2C(=O)c4ccccc34 | Reverse transcriptase |
| CHEMBL3142914 | CCOC(=O)P(=O)(O)OCCCN1C(=O)N(C=C(C)C1=O)[C@@H]2O[C@H](CO[Si](C)(C)C(C)(C)C)[C@@]3(OS(=O)(=O)C=C3N)[C@H]2O[Si](C)(C)C(C)(C)C | Reverse transcriptase |
| CHEMBL46562 | CCN1c2cnccc2C(=O)N(C)c3cccnc13 | Reverse transcriptase |
| CHEMBL287978 | Fc1ccc(CC\N=C(/S)\Nc2ccc(Br)cn2)cc1 | Reverse transcriptase |
| CHEMBL98394 | CCc1cc(NCc2nc3ccccc3[nH]2)c(O)nc1C | Reverse transcriptase |
| CHEMBL214143 | CN(C)C(=O)c1cnnn1[C@@H]2O[C@H](CO[Si](C)(C)C(C)(C)C)[C@@]3(OS(=O)(=O)C=C3N)[C@H]2O[Si](C)(C)C(C)(C)C | Reverse transcriptase |
| CHEMBL60866 | CC(C)(C)c1ccc(cc1)S(=O)(=O)n2ccc(c2)C(O)c3ccc(Cl)cc3Cl | Reverse transcriptase |
| CHEMBL82909 | Cc1cccc(n1)C23SCCN2C(=O)c4cccnc34 | Reverse transcriptase |
| CHEMBL438175 | CC(C)[C@H](N)C(=O)N1CCC[C@H]1C(=O)NCCCN2C(=O)N(C=C(C)C2=O)C3OC(CO[Si](C)(C)C(C)(C)C)C4(OS(=O)(=O)C=C4N)C3O[Si](C)(C)C(C)(C)C | Reverse transcriptase |
| CHEMBL308549 | CSCN1c2ccccc2N(C)C(=O)c3cccnc13 | Reverse transcriptase |
| CHEMBL3142813 | CC1=CN([C@@H]2O[C@H](CO[Si](C)(C)C(C)(C)C)[C@@H](O)[C@H]2O[Si](C)(C)C(C)(C)C)C(=O)N(CC(=O)O)C1=O | Reverse transcriptase |
| CHEMBL3143767 | CN(C)CCCN1C(=O)N(C=C(C)C1=O)[C@@H]2O[C@H](CO[Si](C)(C)C(C)(C)C)[C@@]3(OS(=O)(=O)C=C3N)[C@H]2O[Si](C)(C)C(C)(C)C | Reverse transcriptase |
| CHEMBL262415 | Cc1nc(CCCCCCC(=O)c2ccccc2)n3nc(ccc13)N4CCCCC4 | Reverse transcriptase |
| CHEMBL89669 | OC(=O)c1cc(O)ccc1NC(=O)CCCCC(=O)Nc2ccc(O)cc2C(=O)O | Reverse transcriptase |
| CHEMBL2368777 | CCCN1C=Nc2c(ncn2[C@@H]3O[C@H](COC)[C@@H](OP(=O)(C)O)[C@H]3OC)C1=O | Reverse transcriptase |
| CHEMBL385844 | CC(C)[C@H](N)C(=O)NCCCN1C(=O)N(C=C(C)C1=O)C2OC(CO[Si](C)(C)C(C)(C)C)C3(OS(=O)(=O)C=C3N)C2O[Si](C)(C)C(C)(C)C | Reverse transcriptase |
| CHEMBL301973 | CCN1c2ncccc2c3nnnn3c4c(C)ccnc14 | Reverse transcriptase |
| CHEMBL445417 | CC(C)[C@H](N)C(=O)N[C@@H](Cc1ccccc1)C(=O)NCCCN2C(=O)N(C=C(C)C2=O)C3OC(CO[Si](C)(C)C(C)(C)C)C4(OS(=O)(=O)C=C4N)C3O[Si](C)(C)C(C)(C)C | Reverse transcriptase |
| CHEMBL610949 | CN1N=C(NC(=O)C)c2cn(C3O[C@H](CO)[C@@H](O)[C@H]3O)c4ncnc1c24 | Reverse transcriptase |
| CHEMBL55271 | Nc1cccc(c1C#N)S(=O)(=O)c2ccccc2 | Reverse transcriptase |
| CHEMBL151146 | Oc1cc(cc(O)c1O)C(=O)OCCc2ccccc2 | Reverse transcriptase |
| CHEMBL83356 | CCc1ccccc1CNc2cc(CC)c(C)nc2O | Reverse transcriptase |
| CHEMBL198873 | CC(C)CC(=O)c1c(O)cc(O)c2C(=CC(=O)Oc12)c3ccccc3 | Reverse transcriptase |
| CHEMBL90333 | CSc1ccc(NC(=O)CCCCC(=O)Nc2ccc(SC)cc2C(=O)O)c(c1)C(=O)O | Reverse transcriptase |
| CHEMBL1160974 | CC1=CN(C2CC(N=[N+]=[N-])C(COP(=O)(O)OP(=O)(O)CP(=O)(O)O)O2)C(=O)NC1=O | Reverse transcriptase |
| CHEMBL48690 | CCN1c2ncccc2c3nccn3c4ccc(O)nc14 | Reverse transcriptase |
| CHEMBL108196 | COC(=O)CSc1nc(O)c(C)c(Cc2cccc3ccccc23)n1 | Reverse transcriptase |
| CHEMBL108605 | CCOC(=O)CSc1nc(O)c(C)c(Cc2cccc3ccccc23)n1 | Reverse transcriptase |
| CHEMBL442696 | CN(C1CCN(CC1)C(=O)c2cc3cc(NS(=O)(=O)C)ccc3[nH]2)c4ncccc4CO | Reverse transcriptase |
| CHEMBL290312 | CSCSc1cc(Cc2cc(C)cc(C)c2)c(C(C)C)c(O)n1 | Reverse transcriptase |
| CHEMBL293963 | Nc1cccc(Sc2cccc(c2)C(F)(F)F)c1C#N | Reverse transcriptase |
| CHEMBL156598 | CCCCCCCCOC(=O)c1cc2c3ccccc3[nH]c2c(n1)c4ccc5C(=O)C=C(NC=O)C(=O)c5n4 | Reverse transcriptase |
| CHEMBL264639 | COC(=O)c1cc(cc(C)c1OC)C(=CCCCC(=O)N2CCCCC2)c3cc(C)c(OC)c(c3)C(=O)OC | Reverse transcriptase |
| CHEMBL300757 | Nc1cccc(Sc2ccccc2Cl)c1C#N | Reverse transcriptase |
| CHEMBL405279 | CC1=CN(C2OC(CO[Si](C)(C)C(C)(C)C)C3(OS(=O)(=O)C=C3N)C2O[Si](C)(C)C(C)(C)C)C(=O)N(CCCNC(=O)[C@@H]4CCCN4C(=O)[C@@H](N)CC(=O)O)C1=O | Reverse transcriptase |
| CHEMBL142532 | COC(=O)CCC=C(c1cc(Cl)c(OC)c(c1)C(=O)OC)c2cc(Cl)c(OC)c(c2)C(=O)OC | Reverse transcriptase |
| CHEMBL53896 | Cc1ccc(cc1)S(=O)(=O)c2cccc(N)c2C#N | Reverse transcriptase |
| CHEMBL314625 | OC(=O)c1cc(Br)ccc1NC(=O)CCCC(=O)Nc2ccc(Br)cc2C(=O)O | Reverse transcriptase |
| CHEMBL415703 | CCC(C)Sc1nc(O)cc(Cc2ccc(F)cc2)n1 | Reverse transcriptase |
| CHEMBL290125 | CCC(C)Sc1nc(O)cc(Cc2ccc3ccccc3c2)n1 | Reverse transcriptase |
| CHEMBL88151 | OC(=O)c1cc(Br)ccc1NC(=O)CCCCCCC(=O)Nc2ccc(Br)cc2C(=O)O | Reverse transcriptase |
| CHEMBL85745 | CCc1cc(NCc2ccc(OC)cc2)c(O)nc1C | Reverse transcriptase |
| CHEMBL192976 | Oc1nc2ccc(Cl)cc2n1Cc3c(Cl)cccc3Cl | Reverse transcriptase |
| CHEMBL194923 | COC(=O)CCC\C=C(/c1cccc(c1)C#N)\c2cc(Cl)ccc2OC | Reverse transcriptase |
| CHEMBL387003 | COc1noc2c(C)cc(cc12)\C(=C/CCN3CCOC3=O)\c4cc(C)c5ON(C)C(=O)c5c4 | Reverse transcriptase |
| CHEMBL347273 | Oc1ccc2CC(COc2c1)OC(=O)c3cc(O)c(O)c(O)c3 | Reverse transcriptase |
| CHEMBL72272 | CCN1c2ccccc2N(C)C(=O)c3cc(Cl)cnc13 | Reverse transcriptase |
| CHEMBL380043 | COC(=O)C1=C([C@H]2c3ccccc3S[C@@]12C(=O)OC)N4CCCCC4 | Reverse transcriptase |
| CHEMBL290204 | Cc1nnc2c3cccnc3N(C4CC4)c5ncccc5n12 | Reverse transcriptase |
| CHEMBL83277 | CC1N(C2C[C@@H](N=[N+]=[N-])[C@@H](COCP(=O)(O)OP(=O)(O)OP(=O)(O)O)O2)C(=O)NC(=O)C1=C | Reverse transcriptase |
| CHEMBL175880 | Cc1ccc2[nH]c(nc2c1)\C(=C\c3oc(cc3)c4ccc(O)c(c4)C(=O)O)\C#N | Reverse transcriptase |
| CHEMBL23886 | Nc1nc(O)c2ncn(C3CC(OCP(=O)(O)OP(=O)(O)OP(=O)(O)COC4CC(C=C4)n5cnc6c(O)nc(N)nc56)C=C3)c2n1 | Reverse transcriptase |
| CHEMBL610657 | CC(C)C(=O)NC1=NN(C)c2ncnc3c2c1cn3C4O[C@H](CO)[C@@H](O)[C@H]4O | Reverse transcriptase |
| CHEMBL302957 | CCN1C(=O)c2cccnc2N(CC)c3cccc(C)c13 | Reverse transcriptase |
| CHEMBL84266 | Cc1cccc2c1C(=O)N3CCSC23c4ccccc4 | Reverse transcriptase |
| CHEMBL417311 | CCC(C)Sc1nc(O)cc(Cc2cccc3ccccc23)n1 | Reverse transcriptase |
| CHEMBL333772 | Cc1ccc(NCN2C(=O)c3ccccc3C2=O)c(O)n1 | Reverse transcriptase |
| CHEMBL379994 | CC1=CN(C2OC(CO[Si](C)(C)C(C)(C)C)C3(OS(=O)(=O)C=C3N)C2O[Si](C)(C)C(C)(C)C)C(=O)N(CCCNC(=O)[C@@H]4CCCN4C(=O)[C@@H](N)Cc5ccc(O)cc5)C1=O | Reverse transcriptase |
| CHEMBL107946 | Oc1ncccc1C(OCc2ccncc2)(C#CC3CC3)C(F)(F)F | Reverse transcriptase |
| CHEMBL196274 | COC(=O)CCC\C=C(\c1cc(C)c2ON(C)C(=O)c2c1)/c3cc(Cl)ccc3OC | Reverse transcriptase |
| CHEMBL326205 | CCCOC(=O)CCCC=C(c1cc(Cl)c(OC)c(c1)C(=O)OC)c2cc(Cl)c(OC)c(c2)C(=O)OC | Reverse transcriptase |
| CHEMBL36479 | CC(C)Nc1cccnc1N2CCN(CC2)C(=O)c3cc4cc(NS(=O)(=O)C(F)(F)F)ccc4[nH]3 | Reverse transcriptase |
| CHEMBL89674 | OC(=O)c1cc(ccc1NC(=O)CCCCC(=O)Nc2ccc(cc2C(=O)O)C(F)(F)F)C(F)(F)F | Reverse transcriptase |
| CHEMBL419025 | CC(C)CSc1nc(O)cc(Cc2cccc(C)c2)n1 | Reverse transcriptase |
| CHEMBL175235 | CCc1cc(CCc2ccc3ccccc3c2)c(O)nc1C | Reverse transcriptase |
| CHEMBL609191 | CCC(=O)NC1=NN(C)c2ncnc3c2c1cn3C4O[C@H](CO)[C@@H](O)[C@H]4O | Reverse transcriptase |
| CHEMBL437717 | CC(C)(C)[Si](C)(C)OC[C@H]1O[C@H]([C@H](O[Si](C)(C)C(C)(C)C)[C@]12OS(=O)(=O)C=C2N)n3nncc3C(=O)N | Reverse transcriptase |
| CHEMBL347255 | CCC(C)Sc1nc(O)c(C)c(Cc2ccc(Cl)cc2)n1 | Reverse transcriptase |
| CHEMBL52068 | Nc1cccc(Sc2ccccc2)c1C#N | Reverse transcriptase |
| CHEMBL151175 | Oc1cc(cc(O)c1O)C(=O)OC2COc3cccc(O)c3C2 | Reverse transcriptase |
| CHEMBL315965 | OC(=O)c1cc(Cl)ccc1NC(=O)CCCC(=O)Nc2ccc(Cl)cc2C(=O)O | Reverse transcriptase |
| CHEMBL163536 | O=C1c2ccccc2C34CC=CC[C@@]13c5ccccc5C4=O | Reverse transcriptase |
| CHEMBL114876 | CC1=CN([C@H]2CC(F)(F)[C@@H](CO)O2)C(=O)NC1=O | Reverse transcriptase |
| CHEMBL210790 | COC(=O)C1=C(C2c3ccccc3SC12C(=O)OC)N4CCCCC4 | Reverse transcriptase |
| CHEMBL315784 | OC(=O)c1ccccc1NC(=O)CCCCC(=O)Nc2ccccc2C(=O)O | Reverse transcriptase |
| CHEMBL73568 | CN1C(=O)c2cccnc2N(Cc3ccccc3)c4ccccc14 | Reverse transcriptase |
| CHEMBL134252 | C[C@H]1Oc2c3C=CC(C)(C)Oc3c4C(=CC(=O)Oc4c2C=C1C)c5ccccc5 | Reverse transcriptase |
| CHEMBL59586 | Nc1cccc(Sc2ccccc2C#N)c1C#N | Reverse transcriptase |
| CHEMBL264069 | CC1=CN([C@@H]2O[C@H](CO[Si](C)(C)C(C)(C)C)[C@@]3(OS(=O)(=O)C=C3N)[C@H]2O[Si](C)(C)C(C)(C)C)C(=O)N(CC(=O)N[C@@H](CC(=O)O)C(=O)O)C1=O | Reverse transcriptase |
| CHEMBL323016 | CCCOC(=O)CCCC=C(c1cc(Br)c(OC)c(c1)C(=O)OC)c2cc(Br)c(OC)c(c2)C(=O)OC | Reverse transcriptase |
| CHEMBL89744 | Cc1ccc(NC(=O)CCCCC(=O)Nc2ccc(C)cc2C(=O)O)c(c1)C(=O)O | Reverse transcriptase |
| CHEMBL311050 | Clc1ccc2NC(=O)N[C@@](C#Cc3ccccn3)(C4CC4)c2c1 | Reverse transcriptase |
| CHEMBL109213 | CC(C)CSc1nc(O)cc(Cc2ccccc2)n1 | Reverse transcriptase |
| CHEMBL265502 | Cc1ccc(cc1NC(=O)c2cccc(NC(=O)Nc3cccc(c3)C(=O)Nc4cc(ccc4C)C(=O)Nc5ccc(c6cc(cc(c56)S(=O)(=O)O)S(=O)(=O)O)S(=O)(=O)O)c2)C(=O)Nc7ccc(c8cc(cc(c78)S(=O)(=O)O)S(=O)(=O)O)S(=O)(=O)O | Reverse transcriptase |
| CHEMBL132790 | COC(=O)c1cc(cc(Cl)c1OC)C(=CCCN2CCOC2=O)c3cc(Cl)c(OC)c(c3)C(=O)OC | Reverse transcriptase |
| CHEMBL157342 | O[C@@H]1[C@@H](O)[C@H](Oc2cc(Cl)cc3c2NC(=O)O[C@]3(C#CC4CC4)C(F)(F)F)O[C@@H]([C@H]1O)C(=O)O | Reverse transcriptase |
| CHEMBL2368772 | COC[C@H]1O[C@H]([C@H](O)[C@@H]1OP(=O)(C)O)n2cnc3C(=O)N(CC=C)C=Nc23 | Reverse transcriptase |
| CHEMBL36031 | CCc1c(O)nc(SCSC)nc1Cc2ccccc2 | Reverse transcriptase |
| CHEMBL294212 | Nc1cccc([S+]([O-])c2ccccc2C#N)c1C#N | Reverse transcriptase |
| CHEMBL215346 | COC(=O)c1cc(cc(C)c1OC)\C(=C/CCN2CCOC2=O)\c3cc(C)c4ON(C)C(=O)c4c3 | Reverse transcriptase |
| CHEMBL386515 | CN(C)C(C)(C)c1nc(O)c(O)c(n1)C(=O)NCc2ccc(F)cc2 | Reverse transcriptase |
| CHEMBL358055 | S=C(NCCc1ccccc1)Nc2cc[nH]n2 | Reverse transcriptase |
| CHEMBL53893 | Cc1cccc(c1)[S+]([O-])c2cccc(N)c2C#N | Reverse transcriptase |
| CHEMBL90204 | COc1ccccc1CC\N=C(/NC#N)\Nc2nccs2 | Reverse transcriptase |
| CHEMBL414887 | Clc1cncc(NS(=O)(=O)NCCc2ccccc2)c1 | Reverse transcriptase |
| CHEMBL358507 | S=C(NCCc1cccnc1)Nc2nccs2 | Reverse transcriptase |
| CHEMBL83012 | [O-][N+](=O)c1ccc2C(=O)N3CCSC3(c4ccccc4)c2c1 | Reverse transcriptase |
| CHEMBL82621 | CCc1cc(NCc2cnccc2OC)c(O)nc1C | Reverse transcriptase |
| CHEMBL109512 | CSc1nc(O)cc(Cc2cccc(C)c2)n1 | Reverse transcriptase |
| CHEMBL131432 | CCCCCC=C(c1cc(F)c(OC)c(c1)C(=O)OC)c2cc(F)c(OC)c(c2)C(=O)OC | Reverse transcriptase |
| CHEMBL210297 | OC(=O)c1ccc2c(c1)nc(c3ccc(OCc4cc(ccc4c5ccc(Cl)cc5)N6CCCC6=O)cc3F)n2C7CCCCC7 | Reverse transcriptase |
| CHEMBL22714 | Nc1nc2cc(Cl)c(Cl)cc2n1Cc3ccc(Cl)cc3 | Reverse transcriptase |
| CHEMBL91318 | CC1(Oc2ccccc2n3cccc3C1=O)c4ccccc4 | Reverse transcriptase |
| CHEMBL89082 | Clc1ccc(\N=C(\NCCc2ccccc2)/NC#N)nc1 | Reverse transcriptase |
| CHEMBL143112 | Brc1cccnc1NC(=S)NCCc2ccccc2 | Reverse transcriptase |
| CHEMBL103316 | CN(CCc1ccccc1)\C(=N/c2nccs2)\S | Reverse transcriptase |
| CHEMBL315675 | O=C1N2CCCNC2(c3ccccc3)c4ccccc14 | Reverse transcriptase |
| CHEMBL83640 | CC(C)c1ccc(cc1)C23SCCN2C(=O)c4ccccc34 | Reverse transcriptase |
| CHEMBL84213 | Cc1ccc2c(c1)C(=O)N3CCSC23c4ccccc4 | Reverse transcriptase |
| CHEMBL277903 | Nc1ncnc2c1ncn2C3CC(OCP(=O)(O)OP(=O)(O)OP(=O)(O)COC4CC(C=C4)n5cnc6c(N)ncnc56)C=C3 | Reverse transcriptase |
| CHEMBL107607 | CC1(C)C(=O)NC(=N/C/1=C/c2ccccc2)SC3CCCC3 | Reverse transcriptase |
| CHEMBL334946 | C[C@H]1Oc2c3C=CC(C)(C)Oc3c4C(=CC(=O)Oc4c2C(=O)[C@@H]1C)c5ccccc5 | Reverse transcriptase |
| CHEMBL356674 | Cc1cc(C)cc(Sc2c(C)cnc(O)c2[N+](=O)[O-])c1 | Reverse transcriptase |
| CHEMBL340658 | CCc1cc(NCc2oc3cc4ccccc4cc3n2)c(O)nc1C | Reverse transcriptase |
| CHEMBL109807 | Cc1cccc(Cc2nc(S)nc(O)c2C)c1 | Reverse transcriptase |
| CHEMBL111181 | CC1(C)C(=O)NC(=N/C/1=C/c2ccccc2)SC3CCCCC3 | Reverse transcriptase |
| CHEMBL111449 | Cc1cccc(Cc2cc(O)nc(S)n2)c1 | Reverse transcriptase |
| CHEMBL82662 | [O-][N+](=O)c1ccnc(c1)C#CC2(NC(=O)Nc3ccc(Cl)cc23)C4CC4 | Reverse transcriptase |
| CHEMBL88213 | CCCC1(CC)Oc2ccccc2n3cccc3C1=O | Reverse transcriptase |
| CHEMBL142098 | Cc1cccc(Sc2c(C)cnc(O)c2[N+](=O)[O-])c1 | Reverse transcriptase |
| CHEMBL82628 | O=C1N2CCSC2(c3ccccc3)c4cc5ccccc5cc14 | Reverse transcriptase |
| CHEMBL296346 | Brc1ccc(NC(=S)NCN2CCOCC2)nc1 | Reverse transcriptase |
| CHEMBL147442 | CCCCCCOc1cccc(CC\N=C(/S)\Nc2nccs2)c1 | Reverse transcriptase |
| CHEMBL423020 | OC(=O)c1csc(\N=C(/S)\NCCc2ccccc2)n1 | Reverse transcriptase |
| CHEMBL359230 | O=C(NCCc1ccccc1)Nc2nccs2 | Reverse transcriptase |
| CHEMBL434323 | S=C(NCCc1ccncc1)Nc2nccs2 | Reverse transcriptase |
| CHEMBL1160257 | CC1=CN([C@H]2C[C@@H](O)[C@@H](OP(=O)(O)NP(=O)(O)OP(=O)(O)O)O2)C(=O)NC1=O | Reverse transcriptase |
| CHEMBL87637 | CCC(c1ccccc1)c2cc(O)nc(SC3CCCC3)n2 | Reverse transcriptase |
| CHEMBL210334 | COC(=O)C1=C(C2c3ccccc3SC12C(=O)OC)N4CCC4 | Reverse transcriptase |
| CHEMBL386710 | CN1CCOCC1C2=NC(=C(O)C(=O)N2C)C(=O)NCc3ccc(F)cc3 | Reverse transcriptase |
| CHEMBL144904 | CC(Cc1ccccc1)\N=C(/S)\Nc2nccs2 | Reverse transcriptase |
| CHEMBL84716 | O=C1N2CCS(=O)(=O)C2(c3ccccc3)c4ccccc14 | Reverse transcriptase |
| CHEMBL111152 | CC(C)Sc1nc(O)cc(Cc2ccccc2)n1 | Reverse transcriptase |
| CHEMBL109772 | CSc1nc(O)cc(Cc2ccccc2)n1 | Reverse transcriptase |
| CHEMBL419928 | Cc1c(O)nc(S)nc1Cc2ccccc2 | Reverse transcriptase |
| CHEMBL282787 | CC(C)(C)NC(=O)c1ccccc1C[C@@H](O)[C@H](Cc2ccccc2)NC(=O)[C@H](CC(=O)N)NC(=O)c3ccc4ccccc4n3 | Protease |
| CHEMBL355533 | CC(C)(C)NC(=O)c1ccccc1SC[C@@H](O)[C@H](Cc2ccccc2)NC(=O)[C@H](CC(=O)N)NC(=O)c3ccc4ccccc4n3 | Protease |
| CHEMBL187111 | CCCc1oc(C[C@H](C[C@H](O)CN2CCN(C[C@H]2C(=O)NCC(F)(F)F)C(C)(C)c3oc(cn3)c4ccc(Cl)cc4)C(=O)N[C@H]5CCOC[C@H]5O)nn1 | Protease |
| CHEMBL364855 | CC(C)(N1CCN(C[C@@H](O)C[C@@H](Cc2oc(nn2)c3ccccc3)C(=O)N[C@H]4CCOC[C@H]4O)[C@@H](C1)C(=O)NCC(F)(F)F)c5oc(cn5)c6ccc(Cl)cc6 | Protease |
| CHEMBL364376 | O[C@@H](C[C@@H](Cc1oc(nn1)c2ccccc2)C(=O)N[C@H]3CCOC[C@H]3O)CN4CCN(Cc5oc(cc5)c6ccc(Cl)cc6)C[C@H]4C(=O)NCC(F)(F)F | Protease |
| CHEMBL361443 | Cc1oc(C[C@H](C[C@H](O)CN2CCN(C[C@H]2C(=O)NCC(F)(F)F)C(C)(C)c3oc(cn3)c4ccc(Cl)cc4)C(=O)N[C@H]5CCOC[C@H]5O)nn1 | Protease |
| CHEMBL364409 | CCCc1oc(C[C@H](C[C@H](O)CN2CCN(C[C@H]2C(=O)NCC(F)(F)F)C(C)(C)c3oc(cn3)c4ccc(F)cc4)C(=O)N[C@H]5CCOC[C@H]5O)nn1 | Protease |
| CHEMBL361292 | Cc1oc(C[C@H](C[C@H](O)CN2CCN(Cc3oc(cc3)c4ccc(Cl)cc4)C[C@H]2C(=O)NCC(F)(F)F)C(=O)N[C@H]5CCOC[C@H]5O)nn1 | Protease |
| CHEMBL360212 | O[C@@H](C[C@@H](Cc1ocnn1)C(=O)N[C@H]2CCOC[C@H]2O)CN3CCN(Cc4oc(cc4)c5ccc(Cl)cc5)C[C@H]3C(=O)NCC(F)(F)F | Protease |
| CHEMBL366299 | CCCc1oc(C[C@H](C[C@H](O)CN2CCN(Cc3oc(cc3)c4ccc(Cl)cc4)C[C@H]2C(=O)NCC(F)(F)F)C(=O)N[C@H]5CCOC[C@H]5O)nn1 | Protease |
| CHEMBL184010 | CC(C)(N1CCN(C[C@@H](O)C[C@@H](Cc2ocnn2)C(=O)N[C@H]3CCOC[C@H]3O)[C@@H](C1)C(=O)NCC(F)(F)F)c4oc(cn4)c5ccc(Cl)cc5 | Protease |
| CHEMBL116954 | CC(C)(C)NC(=O)[C@@H]1CN(CCN1C[C@@H](O)C[C@@H](Cc2ccccc2)C(=O)NC3[C@H](O)Cc4ccccc34)S(=O)(=O)c5cccc6cccnc56 | Protease |
| CHEMBL433165 | CC(C)(C)NC(=O)[C@@H]1CN(CCN1C[C@@H](O)C[C@@H](Cc2ccncc2)C(=O)N[C@@H]3[C@H](O)Cc4ccccc34)S(=O)(=O)c5cccc6cccnc56 | Protease |
| CHEMBL444533 | O[C@@H](C[C@@H](Cc1cccnc1)C(=O)N[C@@H]2[C@H](O)COc3ccccc23)CN4CCN(Cc5oc(cc5)c6ccccc6)C[C@H]4C(=O)NCC(F)(F)F | Protease |
| CHEMBL364407 | CC(C)(N1CCN(C[C@@H](O)C[C@@H](Cc2ocnn2)C(=O)N[C@H]3CCOC[C@H]3O)[C@@H](C1)C(=O)NCC(F)(F)F)c4oc(cn4)c5ccc(F)cc5 | Protease |
| CHEMBL408036 | CC(C)(N1CCN(C[C@@H](O)C[C@@H](Cc2cc3c(Cl)cncc3s2)C(=O)N[C@@H]4[C@H](O)COc5ccccc45)[C@@H](C1)C(=O)NCC(F)(F)F)c6oc(cn6)c7ccc(Cl)cc7 | Protease |
| CHEMBL435095 | CC(C)(N1CCN(C[C@@H](O)C[C@@H](Cc2oc(nn2)c3ccccc3)C(=O)N[C@H]4CCOC[C@H]4O)[C@@H](C1)C(=O)NCC(F)(F)F)c5oc(cn5)c6ccc(F)cc6 | Protease |
| CHEMBL338142 | O[C@@H](C[C@@H](Cc1cccnc1)C(=O)N[C@@H]2[C@H](O)COc3ccccc23)CN4CCN(Cc5ccn(c5)c6ccc(Cl)c(Cl)c6)C[C@H]4C(=O)NCC(F)(F)F | Protease |
| CHEMBL445897 | CC(C)(N1CCN(C[C@@H](O)C[C@@H](Cc2ccccc2)C(=O)NC3[C@H](O)COC4C=CC=CC34)[C@@H](C1)C(=O)NCC(F)(F)F)c5oc(cn5)c6cnccc6Cl | Protease |
| CHEMBL321814 | O[C@@H](C[C@@H](Cc1cccnc1)C(=O)N[C@@H]2[C@H](O)COc3ccccc23)CN4CCN(Cc5oc6c(Cl)cccc6c5)C[C@H]4C(=O)NCC(F)(F)F | Protease |
| CHEMBL128395 | O[C@@H](C[C@@H](Cc1cccnc1)C(=O)N[C@@H]2[C@H](O)COc3ccccc23)CN4CCN(Cc5ccn(c5)c6cc(Cl)c(Cl)c(Cl)c6)C[C@H]4C(=O)NCC(F)(F)F | Protease |
| CHEMBL381630 | CC(C)(N1CCN(C[C@@H](O)C[C@@H](Cc2oc3cnccc3c2)C(=O)N[C@@H]4[C@H](O)COc5ccccc45)[C@@H](C1)C(=O)NCC(F)(F)F)c6oc(cn6)c7ccc(Cl)cc7 | Protease |
| CHEMBL313132 | COc1cncc(c1)c2oc(nc2)C(C)(C)N3CCN(C[C@@H](O)C[C@@H](Cc4ccccc4)C(=O)NC5[C@H](O)COC6C=CC=CC56)[C@@H](C3)C(=O)NCC(F)(F)F | Protease |
| CHEMBL380591 | CC(C)(N1CCN(C[C@@H](O)C[C@@H](Cc2cc3ccncc3s2)C(=O)N[C@@H]4[C@H](O)COc5ccccc45)[C@@H](C1)C(=O)NCC(F)(F)F)c6oc(cn6)c7ccc(Cl)cc7 | Protease |
| CHEMBL364404 | Cc1oc(C[C@H](C[C@H](O)CN2CCN(C[C@H]2C(=O)NCC(F)(F)F)C(C)(C)c3oc(cn3)c4ccc(F)cc4)C(=O)N[C@H]5CCOC[C@H]5O)nn1 | Protease |
| CHEMBL373039 | CC(C)(N1CCN(C[C@@H](O)C[C@@H](Cc2cc3cnccc3s2)C(=O)N[C@@H]4[C@H](O)COc5ccccc45)[C@@H](C1)C(=O)NCC(F)(F)F)c6oc(cn6)c7ccc(Cl)cc7 | Protease |
| CHEMBL197493 | O[C@@H](C[C@@H](Cc1cc2cnccc2s1)C(=O)N[C@@H]3[C@H](O)COc4ccccc34)CN5CCN(Cc6oc(cc6)c7ccc(Cl)cc7)C[C@H]5C(=O)NCC(F)(F)F | Protease |
| CHEMBL439513 | Cc1ccncc1c2oc(C[C@H](C[C@H](O)CN3CCN(Cc4oc(cn4)c5ccc(Cl)cc5)C[C@H]3C(=O)NCC(F)(F)F)C(=O)N[C@@H]6[C@H](O)COc7ccccc67)cc2 | Protease |
| CHEMBL321260 | O[C@@H](C[C@@H](Cc1ccncc1)C(=O)N[C@@H]2[C@H](O)COc3ccccc23)CN4CCN(Cc5oc6c(Cl)cccc6c5)C[C@H]4C(=O)NCC(F)(F)F | Protease |
| CHEMBL421568 | CC(C)(C)NC(=O)[C@@H]1CN(Cc2cc(Br)cs2)CCN1C[C@@H](O)C[C@@H](Cc3cccnc3)C(=O)N[C@@H]4[C@H](O)Cc5ccccc45 | Protease |
| CHEMBL127589 | O[C@@H](C[C@@H](Cc1cccnc1)C(=O)N[C@@H]2[C@H](O)COc3ccccc23)CN4CCN(Cc5ccn(c5)c6ccc(Cl)cc6)C[C@H]4C(=O)NCC(F)(F)F | Protease |
| CHEMBL3085527 | CC[C@H](C)[C@H](NC(=O)[C@H](C\C=C\c1ccccc1)C[C@H](O)[C@H](Cc2ccccc2)NC(=O)OC(C)(C)C)C(=O)NCc3nc4ccccc4[nH]3 | Protease |
| CHEMBL430469 | O[C@@H](C[C@@H](Cc1ccccc1)C(=O)N[C@@H]2[C@H](O)Cc3ccccc23)[C@H](Cc4ccccc4)NC(=O)O[C@H]5CCCOC5 | Protease |
| CHEMBL419396 | O[C@@H](C[C@@H](Cc1cccnc1)C(=O)N[C@@H]2[C@H](O)COc3ccccc23)CN4CCN(CC5=CC6C=CSC6S5)C[C@H]4C(=O)NCC(F)(F)F | Protease |
| CHEMBL445944 | O[C@@H](C[C@@H](Cc1ccccc1)C(=O)NC2[C@H](O)COC3C=CC=CC23)CN4CCN(Cc5oc(cc5)c6cccnc6)C[C@H]4C(=O)NCC(F)(F)F | Protease |
| CHEMBL319792 | O[C@@H](C[C@@H](Cc1ccccc1)C(=O)N[C@@H]2[C@H](O)Cc3ccccc23)[C@H](Cc4ccccc4)NC(=O)O[C@@H]5CCCOC5 | Protease |
| CHEMBL319408 | O[C@@H](C[C@@H](Cc1ccccc1)C(=O)N[C@@H]2[C@H](O)Cc3ccccc23)[C@H](Cc4ccccc4)NC(=O)O[C@H]5CCOC5 | Protease |
| CHEMBL372527 | Cc1ccncc1c2oc(C[C@H](C[C@H](O)CN3CCN(C[C@H]3C(=O)NCC(F)(F)F)C(C)(C)c4ncc(s4)c5ccc(Cl)cc5)C(=O)N[C@@H]6[C@H](O)COc7ccccc67)cc2 | Protease |
| CHEMBL96352 | CC(C)(C)NC(=O)[C@@H]1CN(CCN1C[C@@H](O)C[C@@H](Cc2cccnc2)C(=O)N[C@@H]3[C@H](O)Cc4ccccc34)S(=O)(=O)c5cccc6cccnc56 | Protease |
| CHEMBL52702 | Cc1cccc(C)c1CNC(=O)[C@H]2N(CSC2(C)C)C(=O)[C@H](O)C[C@@H](Cc3ccccc3)C(=O)N[C@@H]4[C@H](O)COc5ccccc45 | Protease |
| CHEMBL52669 | Cc1cccc(Cl)c1CNC(=O)[C@H]2N(CSC2(C)C)C(=O)[C@@H](O)C[C@@H](Cc3ccccc3)C(=O)N[C@@H]4[C@H](O)COc5ccccc45 | Protease |
| CHEMBL2062160 | O[C@@H](C[C@@H](Cc1ccccc1)C(=O)N[C@@H]2[C@H](O)Cc3ccccc23)[C@H](Cc4ccccc4)NC(=O)OC5CCOC5 | Protease |
| CHEMBL319092 | O[C@@H](C[C@@H](Cc1ccccc1)C(=O)N[C@@H]2[C@H](O)Cc3ccccc23)[C@H](Cc4ccccc4)NC(=O)O[C@@H]5CCOC5 | Protease |
| CHEMBL350857 | CC(C)(C)NC(=O)c1ccccc1SC[C@H](O)[C@H](Cc2ccccc2)NC(=O)[C@H](CC(=O)N)NC(=O)c3ccc4ccccc4n3 | Protease |
| CHEMBL382951 | CC(C)(N1CCN(C[C@@H](O)C[C@@H](Cc2cc3cnccc3s2)C(=O)N[C@@H]4[C@H](O)COc5ccccc45)[C@@H](C1)C(=O)NCC(F)(F)F)c6ncc(s6)c7ccc(Cl)cc7 | Protease |
| CHEMBL324461 | O[C@@H](C[C@@H](Cc1cccnc1)C(=O)N[C@@H]2[C@H](O)COc3ccccc23)CN4CCN(Cc5oc6ccccc6c5)C[C@H]4C(=O)NCC(F)(F)F | Protease |
| CHEMBL300140 | CC(C)[C@@H]1[C@@H](CCS1(=O)=O)OC(=O)N[C@@H](Cc2ccccc2)[C@H](O)CN3CCN(Cc4ccc(s4)c5cccs5)C[C@H]3C(=O)NC(C)(C)C | Protease |
| CHEMBL433270 | CC1(C)SCN([C@@H]1C(=O)NCc2c(Cl)cccc2Cl)C(=O)[C@@H](O)C[C@@H](Cc3ccccc3)C(=O)N[C@@H]4[C@H](O)COc5ccccc45 | Protease |
| CHEMBL295414 | CC(C)(C)OC(=O)N[C@@H](Cc1ccccc1)[C@@H](O)CC(Cc2ccc(OCC(=O)O)cc2)C(=O)NC3[C@H](O)Cc4ccccc34 | Protease |
| CHEMBL370179 | Cc1ccncc1c2oc(C[C@H](C[C@H](O)CN3CCN(Cc4ccc(s4)c5ccc(Cl)cc5)C[C@H]3C(=O)NCC(F)(F)F)C(=O)N[C@@H]6[C@H](O)COc7ccccc67)cc2 | Protease |
| CHEMBL334573 | O[C@@H](C[C@@H](Cc1cccnc1)C(=O)N[C@@H]2[C@H](O)COc3ccccc23)CN4CCN(Cc5ccn(c5)c6ccc(cc6)C(F)(F)F)C[C@H]4C(=O)NCC(F)(F)F | Protease |
| CHEMBL298457 | O=C(C[C@H]1C(=O)Nc2ccccc2c3cccn13)OCc4ccccc4 | Protease |
| CHEMBL313623 | CC(C)(N1CCN(C[C@@H](O)C[C@@H](Cc2ccccc2)C(=O)NC3[C@H](O)COC4C=CC=CC34)[C@@H](C1)C(=O)NCC(F)(F)F)c5oc(cn5)c6cccnc6 | Protease |
| CHEMBL298189 | Cc1cccc(C)c1CNC(=O)[C@H]2N(CSC2(C)C)C(=O)[C@@H](O)C[C@@H](Cc3ccccc3)C(=O)N[C@@H]4[C@H](O)Cc5ccccc45 | Protease |
| CHEMBL314062 | CC(C)(N1CCN(C[C@@H](O)C[C@@H](Cc2ccccc2)C(=O)NC3[C@H](O)COC4C=CC=CC34)[C@@H](C1)C(=O)NCC(F)(F)F)c5oc(cn5)c6ccccn6 | Protease |
| CHEMBL326796 | O[C@@H](C[C@@H](Cc1ccncc1)C(=O)N[C@@H]2[C@H](O)COc3ccccc23)CN4CCN(Cc5oc(cc5)c6ccccc6)C[C@H]4C(=O)NCC(F)(F)F | Protease |
| CHEMBL340729 | O[C@@H](C[C@@H](Cc1cccnc1)C(=O)N[C@@H]2[C@H](O)COc3ccccc23)CN4CCN(Cc5ccn(c5)c6cccc(F)c6)C[C@H]4C(=O)NCC(F)(F)F | Protease |
| CHEMBL276081 | O[C@@H](C[C@@H](Cc1cccnc1)C(=O)N[C@@H]2[C@H](O)COc3ccccc23)CN4CCN(Cc5ccn(c5)c6cccc(Cl)c6)C[C@H]4C(=O)NCC(F)(F)F | Protease |
| CHEMBL131941 | O[C@@H](C[C@@H](Cc1cccnc1)C(=O)N[C@@H]2[C@H](O)COc3ccccc23)CN4CCN(Cc5ccn(c5)c6cccc(c6)C(F)(F)F)C[C@H]4C(=O)NCC(F)(F)F | Protease |
| CHEMBL315682 | O[C@@H](C[C@@H](Cc1ccccc1)C(=O)NC2[C@H](O)COC3C=CC=CC23)CN4CCN(Cc5oc(cc5)c6ccccn6)C[C@H]4C(=O)NCC(F)(F)F | Protease |
| CHEMBL300754 | Cc1nsc(C)c1CNC(=O)[C@H]2N(CSC2(C)C)C(=O)[C@@H](O)C[C@@H](Cc3ccccc3)C(=O)N[C@@H]4[C@H](O)COc5ccccc45 | Protease |
| CHEMBL370736 | O[C@@H](C[C@@H](Cc1oc2cnccc2c1)C(=O)N[C@@H]3[C@H](O)COc4ccccc34)CN5CCN(Cc6oc(cc6)c7ccc(Cl)cc7)C[C@H]5C(=O)NCC(F)(F)F | Protease |
| CHEMBL199209 | O[C@@H](C[C@@H](Cc1cc2cnccc2s1)C(=O)N[C@@H]3[C@H](O)COc4ccccc34)CN5CCN(Cc6ccc(s6)c7ccc(Cl)cc7)C[C@H]5C(=O)NCC(F)(F)F | Protease |
| CHEMBL128336 | O[C@@H](C[C@@H](Cc1cccnc1)C(=O)N[C@@H]2[C@H](O)COc3ccccc23)CN4CCN(Cc5ccn(c5)c6ccc(F)cc6)C[C@H]4C(=O)NCC(F)(F)F | Protease |
| CHEMBL100310 | CC(C)[C@H](NC(=O)[C@@H](C[C@H](O)[C@H](Cc1ccccc1)NC(=O)OC(C)(C)C)Cc2ccccc2)C(=O)NCC(O)CO | Protease |
| CHEMBL96697 | CC(C)(C)NC(=O)[C@@H]1CN(Cc2cc3sccc3s2)CCN1C[C@@H](O)C[C@@H](Cc4ccncc4)C(=O)N[C@@H]5[C@H](O)Cc6ccccc56 | Protease |
| CHEMBL334379 | O[C@@H](C[C@@H](Cc1cccnc1)C(=O)N[C@@H]2[C@H](O)COc3ccccc23)CN4CCN(Cc5ccn(c5)c6ccc(F)c(F)c6F)C[C@H]4C(=O)NCC(F)(F)F | Protease |
| CHEMBL407828 | CC(C)(N1CCN(C[C@@H](O)C[C@@H](Cc2ccccc2)C(=O)NC3[C@H](O)COC4C=CC=CC34)[C@@H](C1)C(=O)NCC(F)(F)F)c5oc(cc5)c6ccncc6 | Protease |
| CHEMBL17612 | CC(C)(C)NC(=O)[C@@H]1C[C@@H]2CCCC[C@@H]2CN1C[C@@H](O)[C@H](Cc3ccccc3)NC(=O)[C@@H](NC(=O)c4ccc5ccccc5n4)C6CCOC6 | Protease |
| CHEMBL326600 | CC(C)(C)NC(=O)[C@@H]1CN(Cc2ccc(Br)cc2S)CCN1C[C@@H](O)C[C@@H](Cc3ccccc3)C(=O)NC4[C@H](O)Cc5ccccc45 | Protease |
| CHEMBL96100 | CC(C)(C)NC(=O)[C@@H]1CN(Cc2cc3ccsc3s2)CCN1C[C@@H](O)C[C@@H](Cc4ccncc4)C(=O)N[C@@H]5[C@H](O)Cc6ccccc56 | Protease |
| CHEMBL3085503 | CC[C@H](C)[C@H](NC(=O)[C@@H](C[C@H](O)[C@H](Cc1ccccc1)NC(=O)OC(C)(C)C)Cc2ccccc2)C(=O)NCc3nc4ccccc4[nH]3 | Protease |
| CHEMBL340733 | Cc1ccc(cc1)n2ccc(CN3CCN(C[C@@H](O)C[C@@H](Cc4cccnc4)C(=O)N[C@@H]5[C@H](O)COc6ccccc56)[C@@H](C3)C(=O)NCC(F)(F)F)c2 | Protease |
| CHEMBL75904 | CN(C)c1ncc(nc1Cl)C(=O)N[C@@H]([C@H]2CCOC2)C(=O)N[C@@H](Cc3ccccc3)[C@H](O)CN4C[C@H]5CCCC[C@H]5C[C@H]4C(=O)NC(C)(C)C | Protease |
| CHEMBL136146 | O[C@@H](C[C@@H](Cc1ccccc1)C(=O)NC2[C@@H](O)Cc3ccccc23)[C@H](Cc4ccccc4)NC(=O)OC5CCOC5 | Protease |
| CHEMBL197039 | O[C@@H](C[C@@H](Cc1cc2cnccc2s1)C(=O)N[C@@H]3[C@H](O)COc4ccccc34)CN5CCN(Cc6csc(n6)c7ccc(cc7)C(F)(F)F)C[C@H]5C(=O)NCC(F)(F)F | Protease |
| CHEMBL326849 | O[C@@H](C[C@@H](Cc1ccncc1)C(=O)N[C@@H]2[C@H](O)COc3ccccc23)CN4CCN(CC5=CC6C=CSC6S5)C[C@H]4C(=O)NCC(F)(F)F | Protease |
| CHEMBL297720 | Cc1onc(C)c1CNC(=O)[C@H]2N(CSC2(C)C)C(=O)[C@@H](O)C[C@@H](Cc3ccccc3)C(=O)N[C@@H]4[C@H](O)COc5ccccc45 | Protease |
| CHEMBL49768 | COCCOCOCCCc1ccc(C[C@H](C[C@H](O)[C@H](Cc2ccccc2)NC(=O)OC(C)(C)C)C(=O)N[C@@H]3[C@H](O)Cc4ccccc34)cc1 | Protease |
| CHEMBL295442 | CC(C)(C)OC(=O)N[C@@H](Cc1ccccc1)[C@@H](O)C[C@@H](Cc2ccc(OCCN3CC[S+]([O-])CC3)cc2)C(=O)NC4[C@H](O)Cc5ccccc45 | Protease |
| CHEMBL262643 | CC(C)(N1CCN(C[C@@H](O)C[C@@H](Cc2ccccc2)C(=O)NC3[C@H](O)COC4C=CC=CC34)[C@@H](C1)C(=O)NCC(F)(F)F)c5oc6ccncc6c5 | Protease |
| CHEMBL314510 | CC(C)(C)OC(=O)N[C@@H](Cc1ccccc1)[C@@H](O)C[C@@H](Cc2ccccc2)C(=O)N[C@H](C(=O)NCc3nc4ccccc4[nH]3)c5ccccc5 | Protease |
| CHEMBL52645 | Cc1ccccc1CNC(=O)[C@H]2N(CSC2(C)C)C(=O)[C@@H](O)C[C@@H](Cc3ccccc3)C(=O)N[C@@H]4[C@H](O)COc5ccccc45 | Protease |
| CHEMBL79236 | CC(C)(N1CCN(C[C@@H](O)C[C@@H](Cc2ccccc2)C(=O)N[C@@H]3[C@H](O)COc4ccccc34)[C@@H](C1)C(=O)NCC(F)(F)F)c5oc6ccncc6c5 | Protease |
| CHEMBL310924 | CN1CCN(CC1)c2ncc(nc2Cl)C(=O)N[C@@H]([C@H]3CCOC3)C(=O)N[C@@H](Cc4ccccc4)[C@H](O)CN5C[C@H]6CCCC[C@H]6C[C@H]5C(=O)NC(C)(C)C | Protease |
| CHEMBL312238 | CC(C)(C)NC(=O)[C@@H]1CN(CCN1C[C@@H](O)C[C@@H](Cc2ccccc2)C(=O)N[C@@H]3[C@H](O)COc4ccccc34)C(C)(C)c5oc6ccncc6c5 | Protease |
| CHEMBL416082 | CC(C)(C)OC(=O)N[C@@H](Cc1ccccc1)[C@@H](O)C[C@@H](Cc2ccc(OCCN3CCS(=O)(=O)CC3)cc2)C(=O)NC4[C@H](O)Cc5ccccc45 | Protease |
| CHEMBL198531 | Cc1ccncc1c2oc(C[C@H](C[C@H](O)CN3CCN(Cc4oc(cc4)c5ccc(Cl)cc5)C[C@H]3C(=O)NCC(F)(F)F)C(=O)N[C@@H]6[C@H](O)COc7ccccc67)cc2 | Protease |
| CHEMBL313383 | O[C@@H](C[C@@H](Cc1ccccc1)C(=O)NC2[C@H](O)COC3C=CC=CC23)CN4CCN(Cc5oc(cc5)c6ccncc6)C[C@H]4C(=O)NCC(F)(F)F | Protease |
| CHEMBL297294 | COC(CCc1ccc(C[C@H](C[C@H](O)[C@H](Cc2ccccc2)NC(=O)OC(C)(C)C)C(=O)N[C@@H]3[C@H](O)Cc4ccccc34)cc1)OC | Protease |
| CHEMBL114756 | O[C@@H](C[C@@H](Cc1ccccc1)C(=O)N[C@@H]2[C@H](O)COc3ccccc23)CN4CCN(Cc5oc(cc5)c6ccncc6)C[C@H]4C(=O)NCC(F)(F)F | Protease |
| CHEMBL316454 | CC(C)(C)NC(=O)[C@@H]1CN(Cc2cc3sccc3s2)CCN1C[C@@H](O)C[C@@H](Cc4cccnc4)C(=O)N[C@@H]5[C@H](O)Cc6ccccc56 | Protease |
| CHEMBL295549 | CC(C)(C)OC(=O)N[C@@H](Cc1ccccc1)[C@@H](O)C[C@@H](Cc2ccc(OCCO)cc2)C(=O)NC3[C@H](O)Cc4ccccc34 | Protease |
| CHEMBL334572 | O[C@@H](C[C@@H](Cc1cccnc1)C(=O)N[C@@H]2[C@H](O)COc3ccccc23)CN4CCN(Cc5ccn(c5)c6ccc(cc6)C#N)C[C@H]4C(=O)NCC(F)(F)F | Protease |
| CHEMBL421786 | CC(C)(C)NC(=O)[C@@H]1CN(Cc2ncc(s2)c3ccccc3)CCN1C[C@@H](O)C[C@@H](Cc4ccccc4)C(=O)N[C@@H]5[C@H](O)Cc6ccccc56 | Protease |
| CHEMBL85930 | O[C@@H](C[C@@H](Cc1ccccc1)C(=O)NC2[C@H](O)COC3C=CC=CC23)CN4CCN(Cc5oc(cn5)c6ccncc6)C[C@H]4C(=O)NCC(F)(F)F | Protease |
| CHEMBL419923 | CC(C)(C)OC(=O)N[C@@H](Cc1ccccc1)[C@@H](O)C[C@@H](Cc2ccccc2)C(=O)N[C@@H]3[C@H](O)[C@H](O)c4ccccc34 | Protease |
| CHEMBL325829 | O[C@@H](C[C@@H](Cc1ccncc1)C(=O)N[C@@H]2[C@H](O)COc3ccccc23)CN4CCN(Cc5oc6ccccc6c5)C[C@H]4C(=O)NCC(F)(F)F | Protease |
| CHEMBL95611 | CC(C)(C)NC(=O)[C@H]1CN(CCN1C[C@H](O)C[C@@H](Cc2ccccc2)C(=O)NC3[C@H](O)Cc4ccccc34)S(=O)(=O)c5cccs5 | Protease |
| CHEMBL422323 | Cn1nc(cc1c2ccc(CN3CCN(C[C@@H](O)C[C@@H](Cc4ccccc4)C(=O)N[C@@H]5[C@H](O)Cc6ccccc56)[C@@H](C3)C(=O)NC(C)(C)C)s2)C(F)(F)F | Protease |
| CHEMBL103400 | CC(C)(C)OC(=O)N[C@@H](Cc1ccccc1)[C@@H](O)C[C@@H](Cc2ccccc2)C(=O)N[C@H]3[C@H](O)[C@H](O)c4ccccc34 | Protease |
| CHEMBL341176 | Cc1cccc(c1)n2ccc(CN3CCN(C[C@@H](O)C[C@@H](Cc4cccnc4)C(=O)N[C@@H]5[C@H](O)COc6ccccc56)[C@@H](C3)C(=O)NCC(F)(F)F)c2 | Protease |
| CHEMBL1203459 | Cl.CN(C)c1ccc2cc(ccc2n1)S(=O)(=O)N3CCN(C[C@@H](O)C[C@@H](Cc4ccccc4)C(=O)N[C@@H]5[C@H](O)Cc6ccccc56)[C@@H](C3)C(=O)NC(C)(C)C | Protease |
| CHEMBL293992 | CC(C)[C@@H]1[C@@H](CCS1(=O)=O)OC(=O)N[C@@H](Cc2ccccc2)[C@H](O)CN3CCN(Cc4cc5ccccc5s4)C[C@H]3C(=O)NC(C)(C)C | Protease |
| CHEMBL138828 | CC(C)(C)OC(=O)N[C@@H](Cc1ccccc1)[C@@H](O)C[C@@H](Cc2ccccc2)C(=O)NC3[C@@H](O)C(O)c4ccccc34 | Protease |
| CHEMBL44489 | CC(C)(C)OC(=O)N[C@@H](Cc1ccccc1)[C@@H](O)C[C@@H](Cc2ccc(CCCO)cc2)C(=O)N[C@@H]3[C@H](O)Cc4ccccc34 | Protease |
| CHEMBL331155 | Cc1c(CN2CCN(CC(O)CC(Cc3ccccc3)C(=O)NC4C(O)Cc5ccccc45)C(C2)C(=O)NC(C)(C)C)oc6ccccc16 | Protease |
| CHEMBL341089 | O[C@@H](C[C@@H](Cc1cccnc1)C(=O)N[C@@H]2[C@H](O)COc3ccccc23)CN4CCN(Cc5ccn(c5)c6ccccc6)C[C@H]4C(=O)NCC(F)(F)F | Protease |
| CHEMBL294392 | CC(C)[C@@H]1[C@@H](CCS1(=O)=O)OC(=O)N[C@@H](Cc2ccccc2)[C@H](O)CN3CCN(Cc4cc5ccsc5s4)C[C@H]3C(=O)NC(C)(C)C | Protease |
| CHEMBL197114 | Cc1ccncc1c2oc(C[C@H](C[C@H](O)CN3CCN(Cc4csc(n4)c5ccc(F)cc5)C[C@H]3C(=O)NCC(F)(F)F)C(=O)N[C@@H]6[C@H](O)COc7ccccc67)cc2 | Protease |
| CHEMBL113845 | CC(C)(C)NC(=O)C1CN(Cc2cc3cccnc3s2)CCN1CC(O)CC(Cc4ccccc4)C(=O)NC5C(O)Cc6ccccc56 | Protease |
| CHEMBL73934 | CC(C)(C)NC(=O)[C@@H]1C[C@@H]2CCCC[C@@H]2CN1C[C@@H](O)[C@H](Cc3ccccc3)NC(=O)[C@@H](NC(=O)C4=CC(=O)c5ccccc5O4)[C@H]6CCOC6 | Protease |
| CHEMBL318777 | CC(C)(C)NC(=O)[C@@H]1CN(Cc2cc(Br)cs2)CCN1C[C@@H](O)C[C@@H](Cc3ccncc3)C(=O)N[C@@H]4[C@H](O)Cc5ccccc45 | Protease |
| CHEMBL285759 | CC(C)[C@]1(CCc2ccc(N)cc2)CC(=O)C(=C(O)O1)Sc3cc(C)c(CO)cc3C(C)(C)C | Protease |
| CHEMBL96645 | CC(C)(C)NC(=O)[C@@H]1CN(Cc2cc3ccsc3s2)CCN1C[C@@H](O)C[C@@H](Cc4cccnc4)C(=O)N[C@@H]5[C@H](O)Cc6ccccc56 | Protease |
| CHEMBL307918 | CC(C)(C)NC(=O)[C@@H]1C[C@@H]2CCCC[C@@H]2CN1C[C@@H](O)[C@H](Cc3ccccc3)NC(=O)[C@@H](NC(=O)c4cnc5ccccc5n4)[C@H]6CCOC6 | Protease |
| CHEMBL211715 | CC(C)CN([C@H](CO)CCCCNC(=O)[C@@H](NC(=O)OCc1ccccn1)C(c2ccccc2)c3ccccc3)S(=O)(=O)c4ccc(N)cc4 | Protease |
| CHEMBL297066 | COCCOCOCCc1ccc(C[C@H](C[C@H](O)[C@H](Cc2ccccc2)NC(=O)OC(C)(C)C)C(=O)N[C@@H]3[C@H](O)Cc4ccccc34)cc1 | Protease |
| CHEMBL2092879 | CC(C)[C@]1(CCc2ccsc2)CC(=O)C(=C(O)O1)Sc3cc(C)c(CO)cc3C(C)(C)C | Protease |
| CHEMBL85903 | O[C@@H](C[C@@H](Cc1ccccc1)C(=O)NC2[C@H](O)COC3C=CC=CC23)CN4CCN(Cc5oc(cn5)c6ccccn6)C[C@H]4C(=O)NCC(F)(F)F | Protease |
| CHEMBL149166 | Cn1nc(cc1c2ccc(CN3CCN(C[C@@H](O)C[C@@H](Cc4ccccc4)C(=O)NC5[C@H](O)Cc6ccccc56)[C@@H](C3)C(=O)NC(C)(C)C)s2)C(F)(F)F | Protease |
| CHEMBL301571 | Cc1cccnc1CNC(=O)[C@H]2N(CSC2(C)C)C(=O)[C@@H](O)C[C@@H](Cc3ccccc3)C(=O)N[C@@H]4[C@H](O)COc5ccccc45 | Protease |
| CHEMBL333828 | CC(C)(C)NC(=O)[C@@H]1CN(CCN1C[C@@H](O)C[C@@H](Cc2ccccc2)C(=O)NC3[C@H](O)Cc4ccccc34)C(=O)C5=Cc6ccccc6OC5=O | Protease |
| CHEMBL423611 | CC(C)(C)NC(=O)c1ccccc1SC[C@@H](O)[C@H](Cc2ccccc2)NC(=O)[C@H](Cc3c[nH]cn3)NC(=O)c4ccc5ccccc5n4 | Protease |
| CHEMBL318525 | CC(C)(C)NC(=O)[C@@H]1CN(Cc2c(F)cccc2F)CCN1C[C@@H](O)C[C@@H](Cc3cccnc3)C(=O)N[C@@H]4[C@H](O)Cc5ccccc45 | Protease |
| CHEMBL311953 | CC(C)(N1CCN(C[C@@H](O)C[C@@H](Cc2ccccc2)C(=O)N[C@@H]3[C@H](O)COc4ccccc34)[C@@H](C1)C(=O)NCC(F)(F)C(F)(F)F)c5oc6ccncc6c5 | Protease |
| CHEMBL313133 | O[C@@H](C[C@@H](Cc1ccccc1)C(=O)NC2[C@H](O)COC3C=CC=CC23)CN4CCN(Cc5oc(cn5)c6cccnc6)C[C@H]4C(=O)NCC(F)(F)F | Protease |
| CHEMBL326336 | Cc1nn(C)c2ncc(CN3CCN(C[C@@H](O)C[C@@H](Cc4ccccc4)C(=O)NC5[C@H](O)Cc6ccccc56)[C@@H](C3)C(=O)NC(C)(C)C)c(Cl)c12 | Protease |
| CHEMBL325951 | CC(C)(C)NC(=O)[C@@H]1CN(Cc2cnc3ccccc3c2)CCN1C[C@@H](O)C[C@@H](Cc4ccccc4)C(=O)NC5[C@H](O)Cc6ccccc56 | Protease |
| CHEMBL47480 | CC(C)C[C@H](NC(=O)[C@H](C\C=C\c1ccccc1)C[C@H](O)[C@H](Cc2ccccc2)NC(=O)OC(C)(C)C)C(=O)N[C@@H](Cc3ccccc3)C(=O)N | Protease |
| CHEMBL276121 | O[C@@H](C[C@@H](Cc1cccnc1)C(=O)N[C@@H]2[C@H](O)COc3ccccc23)CN4CCN(Cc5ccn(c5)c6cccc(c6)C#N)C[C@H]4C(=O)NCC(F)(F)F | Protease |
| CHEMBL309175 | CC(C)[C@H](NC(=O)C1=CC(=O)c2ccccc2O1)C(=O)N[C@@H](Cc3ccccc3)[C@H](O)CN4C[C@H]5CCCC[C@H]5C[C@H]4C(=O)NC(C)(C)C | Protease |
| CHEMBL283583 | CCC(C)C(NC(=O)[C@@H](C[C@H](O)[C@H](Cc1ccccc1)NC(=O)OC(C)(C)C)Cc2ccccc2)C(=O)NCC(O)CO | Protease |
| CHEMBL295470 | CC(C)(C)OC(=O)N[C@@H](Cc1ccc(O)cc1)[C@@H](O)C[C@@H](Cc2ccc(O)cc2)C(=O)NC3[C@H](O)Cc4ccccc34 | Protease |
| CHEMBL78876 | CC(C)(C)NC(=O)[C@@H]1CN(CCN1C[C@@H](O)C[C@@H](Cc2ccccc2)C(=O)N[C@@H]3[C@H](O)Cc4ccccc34)C(C)(C)c5oc6ccncc6c5 | Protease |
| CHEMBL431999 | CCC(C)[C@H](NC(=O)[C@@H](C[C@H](O)[C@H](Cc1ccccc1)NC(=O)OC(C)(C)C)Cc2ccccc2)C(=O)NCC(O)CO | Protease |
| CHEMBL298316 | CC(C)(C)OC(=O)N[C@@H](Cc1ccccc1)[C@@H](O)C[C@@H](Cc2ccc(O)cc2)C(=O)N[C@@H]3[C@H](O)Cc4ccccc34 | Protease |
| CHEMBL138300 | CC(C)(C)OC(=O)N[C@@H](Cc1ccccc1)[C@@H](O)C[C@@H](Cc2ccc(O)cc2)C(=O)NC3[C@@H](O)Cc4ccccc34 | Protease |
| CHEMBL299884 | Cc1ccnc(C)c1CNC(=O)[C@H]2N(CSC2(C)C)C(=O)[C@@H](O)C[C@@H](Cc3ccccc3)C(=O)N[C@@H]4[C@H](O)COc5ccccc45 | Protease |
| CHEMBL306974 | COC(=O)c1nc(Cl)c(N[C@@H]([C@H]2CCOC2)C(=O)N[C@@H](Cc3ccccc3)[C@H](O)CN4C[C@H]5CCCC[C@H]5C[C@H]4C(=O)NC(C)(C)C)nc1N | Protease |
| CHEMBL116628 | Cc1nc(c(C)s1)S(=O)(=O)N2CCN(C[C@@H](O)C[C@@H](Cc3ccccc3)C(=O)NC4[C@H](O)Cc5ccccc45)[C@@H](C2)C(=O)NC(C)(C)C | Protease |
| CHEMBL337449 | Cc1ccccc1n2ccc(CN3CCN(C[C@@H](O)C[C@@H](Cc4cccnc4)C(=O)N[C@@H]5[C@H](O)COc6ccccc56)[C@@H](C3)C(=O)NCC(F)(F)F)c2 | Protease |
| CHEMBL131994 | O[C@@H](C[C@@H](Cc1cccnc1)C(=O)N[C@@H]2[C@H](O)COc3ccccc23)CN4CCN(Cc5ccn(c5)c6ccccc6F)C[C@H]4C(=O)NCC(F)(F)F | Protease |
| CHEMBL318531 | CC(C)(C)OC(=O)N[C@@H](Cc1ccccc1)[C@@H](O)C[C@@H](Cc2ccc(O)cc2)C(=O)N[C@H]3[C@H](O)Cc4ccccc34 | Protease |
| CHEMBL291196 | CC(C)(C)OC(=O)N[C@@H](Cc1ccccc1)[C@@H](O)C[C@@H](Cc2ccc(CCO)cc2)C(=O)N[C@@H]3[C@H](O)Cc4ccccc34 | Protease |
| CHEMBL295471 | CC(C)(C)OC(=O)N[C@@H](Cc1ccccc1)[C@@H](O)C[C@@H](Cc2ccc(cc2)S(=O)(=O)C)C(=O)N[C@@H]3[C@H](O)Cc4ccccc34 | Protease |
| CHEMBL295530 | CC(C)(C)OC(=O)N[C@@H](Cc1ccccc1)[C@@H](O)C[C@@H](C\C=C\c2ccc(O)cc2)C(=O)N[C@@H]3[C@H](O)Cc4ccccc34 | Protease |
| CHEMBL46114 | CC(C)(C)OC(=O)N[C@@H](Cc1ccccc1)[C@@H](O)C[C@@H](Cc2ccc(CO)cc2)C(=O)N[C@@H]3[C@H](O)Cc4ccccc34 | Protease |
| CHEMBL306073 | CC(C)C1(CCc2ccsc2)CC(=O)C(=C(O)O1)Sc3cc(C)c(CO)cc3C(C)(C)C | Protease |
| CHEMBL408413 | CC(C)(C)NC(=O)[C@@H]1CN(Cc2cnc3ccoc3c2)CCN1C[C@@H](O)C[C@@H](Cc4ccccc4)C(=O)NC5[C@H](O)Cc6ccccc56 | Protease |
| CHEMBL297620 | CC(C)(C)OC(=O)N[C@@H](Cc1ccccc1)[C@@H](O)C[C@@H](Cc2ccc(cc2)C(C)(C)C)C(=O)N[C@@H]3[C@H](O)Cc4ccccc34 | Protease |
| CHEMBL319417 | CC(C)(C)OC(=O)N[C@@H](Cc1ccccc1)[C@@H](O)C[C@@H](Cc2ccc(cc2)C(C)(C)C)C(=O)N[C@H]3[C@H](O)Cc4ccccc34 | Protease |
| CHEMBL47610 | CC(C)(C)OC(=O)N[C@@H](Cc1ccccc1)[C@@H](O)C[C@@H](CCc2cnc3ccccc3n2)C(=O)N[C@@H]4[C@H](O)Cc5ccccc45 | Protease |
| CHEMBL136708 | CC(C)(C)OC(=O)N[C@@H](Cc1ccccc1)[C@@H](O)C[C@@H](Cc2ccccc2)C(=O)NC3[C@@H](O)COc4ccccc34 | Protease |
| CHEMBL111143 | CC(C)(C)OC(=O)N[C@@H](Cc1ccccc1)[C@@H](O)C[C@@H](Cc2ccccc2)C(=O)N[C@@H]3[C@H](O)COc4ccccc34 | Protease |
| CHEMBL45451 | CC(C)(C)OC(=O)N[C@@H](Cc1ccccc1)[C@@H](O)C[C@@H](Cc2ccc(OCc3ccccc3)cc2)C(=O)NC4[C@H](O)Cc5ccccc45 | Protease |
| CHEMBL116052 | CC(C)(C)NC(=O)C1CN(Cc2oc3ncc(CO)cc3c2)CCN1CC(O)CC(Cc4ccccc4)C(=O)NC5C(O)Cc6ccccc56 | Protease |
| CHEMBL1233940 | CC(C)(C)NC(=O)[C@@H]1CN(Cc2oc3ccccc3c2)CCN1C[C@@H](O)C[C@@H](Cc4ccccc4)C(=O)N[C@@H]5[C@H](O)Cc6ccccc56 | Protease |
| CHEMBL337701 | O[C@@H](C[C@@H](Cc1cccnc1)C(=O)N[C@@H]2[C@H](O)COc3ccccc23)CN4CCN(Cc5ccn(c5)c6ccccc6Cl)C[C@H]4C(=O)NCC(F)(F)F | Protease |
| CHEMBL292193 | CC(C)[C@@H]1[C@@H](CCS1(=O)=O)OC(=O)N[C@@H](Cc2ccccc2)[C@H](O)CN3CCN(Cc4cc(Br)cs4)C[C@H]3C(=O)NC(C)(C)C | Protease |
| CHEMBL103213 | CC(C)(C)OC(=O)N[C@@H](Cc1ccccc1)[C@@H](O)C[C@@H](Cc2ccccc2)C(=O)N[C@H]3[C@H](O)COc4ccccc34 | Protease |
| CHEMBL430875 | CC(C)(C)OC(=O)N[C@@H](Cc1ccccc1)[C@@H](O)C[C@@H](CCCc2ccccc2)C(=O)N[C@H]3[C@H](O)[C@H](O)c4ccccc34 | Protease |
| CHEMBL325013 | Cc1cc2oc(CN3CCN(C[C@@H](O)C[C@@H](Cc4ccccc4)C(=O)NC5[C@H](O)Cc6ccccc56)[C@@H](C3)C(=O)NC(C)(C)C)cc2cn1 | Protease |
| CHEMBL77786 | CC(C)C1(CCc2csc(CO)c2)CC(=O)C(=C(O)O1)Sc3cc(C)c(CO)cc3C(C)(C)C | Protease |
| CHEMBL322984 | CC(C)(C)OC(=O)N[C@@H](Cc1ccccc1)[C@@H](O)C[C@@H](CCCc2ccccc2)C(=O)N[C@@H]3[C@H](O)[C@H](O)c4ccccc34 | Protease |
| CHEMBL337349 | CC(C)(C)OC(=O)N[C@@H](Cc1ccccc1)[C@@H](O)C[C@@H](CCCc2ccccc2)C(=O)NC3[C@@H](O)C(O)c4ccccc34 | Protease |
| CHEMBL99267 | CC(C)(C)NC(=O)[C@@H]1C[C@@H]2SCC[C@@H]2CN1C[C@@H](O)[C@H](Cc3ccccc3)NC(=O)[C@@H](CS(=O)(=O)c4ccc5ccccc5c4)NC(=O)C(F)(F)F | Protease |
| CHEMBL297673 | CC(C)(C)OC(=O)N[C@@H](Cc1ccccc1)[C@@H](O)C[C@@H](Cc2ccc(OCC(=O)N3CCOCC3)cc2)C(=O)NC4[C@H](O)Cc5ccccc45 | Protease |
| CHEMBL114 | CC(C)(C)NC(=O)[C@@H]1C[C@@H]2CCCC[C@@H]2CN1C[C@@H](O)[C@H](Cc3ccccc3)NC(=O)[C@H](CC(=O)N)NC(=O)c4ccc5ccccc5n4 | Protease |
| CHEMBL98589 | CC(C)(C)NC(=O)[C@@H]1C[C@@H]2SCC[C@@H]2CN1C[C@@H](O)[C@H](Cc3ccccc3)NC(=O)[C@@H](CS(=O)(=O)c4ccc5ccccc5c4)NS(=O)(=O)C | Protease |
| CHEMBL44578 | CN1CCN(CCOc2ccc(C[C@H](C[C@H](O)[C@H](Cc3ccccc3)NC(=O)OC(C)(C)C)C(=O)NC4[C@H](O)Cc5ccccc45)cc2)CC1 | Protease |
| CHEMBL276447 | CC(C)[C@H](NC(=O)[C@@H](C[C@H](O)[C@H](Cc1ccccc1)NC(=O)OC(C)(C)C)Cc2ccccc2)C(=O)NCc3nc4ccccc4[nH]3 | Protease |
| CHEMBL46274 | CC(C)(C)OC(=O)N[C@@H](Cc1ccccc1)[C@@H](O)C[C@@H](Cc2ccc(OCC[N+]3([O-])CCOCC3)cc2)C(=O)NC4[C@H](O)Cc5ccccc45 | Protease |
| CHEMBL326337 | CC(C)(C)NC(=O)C1CN(Cc2csc3ncccc23)CCN1CC(O)CC(Cc4ccccc4)C(=O)NC5C(O)Cc6ccccc56 | Protease |
| CHEMBL318114 | Cc1cc(O)cc(C)c1CNC(=O)[C@@H](C[C@@H](O)CN2CCN(C[C@@H]2C(=O)NC(C)(C)C)S(=O)(=O)c3ccc4N(CCc4c3)c5ncccn5)Cc6ccccc6 | Protease |
| CHEMBL48565 | CC(C)[C@H](NC(=O)OCc1ccccc1)C(=O)N[C@@H](Cc2ccccc2)[C@@H](O)[C@@H](O)[C@H](Cc3ccccc3)NC(=O)[C@@H](NC(=O)OCc4ccccc4)C(C)C | Protease |
| CHEMBL100040 | CC(C)(C)NC(=O)[C@@H]1C[C@@H]2SCC[C@@H]2CN1C[C@@H](O)[C@H](Cc3ccccc3)NC(=O)[C@@H](CS(=O)(=O)c4ccc(F)cc4)NC(=O)C(F)(F)F | Protease |
| CHEMBL95733 | CSc1nccc(n1)c2ccc(s2)S(=O)(=O)N3CCN(C[C@H](O)C[C@@H](Cc4ccccc4)C(=O)NCc5c(C)cc(O)cc5C)[C@H](C3)C(=O)NC(C)(C)C | Protease |
| CHEMBL297189 | CC(C)(C)OC(=O)N[C@@H](Cc1ccccc1)[C@@H](O)C[C@@H](Cc2cccc(O)c2)C(=O)N[C@@H]3[C@H](O)Cc4ccccc34 | Protease |
| CHEMBL95228 | CC(C)(C)NC(=O)[C@@H]1C[C@@H]2SCC[C@@H]2CN1C[C@@H](O)[C@H](Cc3ccccc3)NC(=O)[C@@H](CS(=O)(=O)c4cccc5cccnc45)NC(=O)C(F)(F)F | Protease |
| CHEMBL295357 | CSc1ccc(C[C@H](C[C@H](O)[C@H](Cc2ccccc2)NC(=O)OC(C)(C)C)C(=O)N[C@@H]3[C@H](O)Cc4ccccc34)cc1 | Protease |
| CHEMBL83462 | CC(C)[C@@H](NC(=O)OCc1ccccc1)C(=O)N[C@@H](Cc2ccccc2)[C@@H](O)[C@H](O)[C@H](Cc3ccccc3)NC(=O)[C@@H](NC(=O)OCc4ccccc4)C(C)C | Protease |
| CHEMBL9544 | CC(C)C(NC(=O)OCc1ccccc1)C(=O)N[C@@H](Cc2ccccc2)[C@H](O)[C@@H](O)[C@H](Cc3ccccc3)NC(=O)C(NC(=O)OCc4ccccc4)C(C)C | Protease |
| CHEMBL324025 | CC(C)(C)NC(=O)[C@@H]1CN(Cc2oc3ccncc3c2)CCN1C[C@@H](O)C[C@@H](Cc4ccccc4)C(=O)NC5[C@H](O)Cc6ccccc56 | Protease |
| CHEMBL314298 | CC(C)[C@@H](NC(=O)OCc1ccccc1)C(=O)N[C@@H](Cc2ccccc2)[C@H](O)[C@H](O)[C@H](Cc3ccccc3)NC(=O)[C@@H](NC(=O)OCc4ccccc4)C(C)C | Protease |
| CHEMBL98954 | CC(C)(C)NC(=O)[C@@H]1C[C@@H]2SCC[C@@H]2CN1C[C@@H](O)[C@H](Cc3ccccc3)NC(=O)[C@@H](CS(=O)(=O)c4ccc(F)cc4)NS(=O)(=O)C | Protease |
| CHEMBL289846 | CC(C)[C@H](NC(=O)OCc1ccccc1)C(=O)N[C@@H](Cc2ccccc2)[C@@H](O)[C@H](O)[C@H](Cc3ccccc3)NC(=O)[C@@H](NC(=O)OCc4ccccc4)C(C)C | Protease |
| CHEMBL358139 | CN(C)c1ccc2ccc(cc2n1)S(=O)(=O)N3CCN(C[C@@H](O)C[C@@H](Cc4ccccc4)C(=O)NC5[C@H](O)Cc6ccccc56)[C@@H](C3)C(=O)NC(C)(C)C | Protease |
| CHEMBL9705 | CC(C)C(NC(=O)OCc1ccccc1)C(=O)N[C@@H](Cc2ccccc2)[C@@H](O)[C@@H](O)[C@H](Cc3ccccc3)NC(=O)C(NC(=O)OCc4ccccc4)C(C)C | Protease |
| CHEMBL433660 | CC(C)(C)OC(=O)N[C@@H](Cc1ccccc1)[C@@H](O)C[C@@H](C\C=C\c2ccccc2)C(=O)NC3[C@@H](O)Cc4ccccc34 | Protease |
| CHEMBL265514 | CC(C)(C)OC(=O)N[C@@H](Cc1ccccc1)[C@@H](O)C[C@@H](C\C=C\c2ccccc2)C(=O)N[C@@H]3[C@H](O)Cc4ccccc34 | Protease |
| CHEMBL81840 | CC(C)(C)NC(=O)[C@@H]1CN(Cc2oc3ccncc3c2)CCN1C[C@@H](O)C[C@@H](Cc4ccccc4)C(=O)N[C@@H]5[C@H](O)Cc6ccccc56 | Protease |
| CHEMBL296115 | CC(C)(C)OC(=O)N[C@@H](Cc1ccccc1)[C@@H](O)C[C@@H](Cc2ccccc2)C(=O)N[C@@H]3[C@H](O)Cc4ccccc34 | Protease |
| CHEMBL83506 | CC(C)(C)NC(=O)[C@@H]1C[C@@H]2CCCC[C@@H]2CN1C[C@@H](O)[C@H](Cc3ccccc3)NC(=O)[C@@H](CC(=O)N)NC(=O)c4ccc5ccccc5n4 | Protease |
| CHEMBL418986 | CC(C)(C)OC(=O)N[C@@H](Cc1ccccc1)[C@@H](O)C[C@@H](C\C=C\c2ccccc2)C(=O)N[C@H]3[C@H](O)Cc4ccccc34 | Protease |
| CHEMBL413515 | CN(C)c1ccc(CNC(=O)[C@@H](NC(=O)c2ccccc2c3ccccc3)[C@@H]4N[C@@H](C(=O)NCCNC(=O)[C@@H]5N[C@H](SC5(C)C)[C@H](NC(=O)c6ccccc6c7ccccc7)C(=O)NCc8ccc(cc8)N(C)C)C(C)(C)S4)cc1 | Protease |
| CHEMBL378044 | CC(C)CN([C@H](CO)CCCCNC(=O)[C@@H](NC(=O)Cc1cccnc1)C(c2ccccc2)c3ccccc3)S(=O)(=O)c4ccc(N)cc4 | Protease |
| CHEMBL432924 | CN(C)c1nc(N)c(nc1Cl)C(=O)N[C@@H]([C@H]2CCOC2)C(=O)N[C@@H](Cc3ccccc3)[C@H](O)CN4C[C@H]5CCCC[C@H]5C[C@H]4C(=O)NC(C)(C)C | Protease |
| CHEMBL77497 | CC(C)C1(CCc2cncs2)CC(=O)C(=C(O)O1)Sc3cc(C)c(CO)cc3C(C)(C)C | Protease |
| CHEMBL329750 | CC(C)[C@H](NC(=O)[C@@H](C[C@H](O)[C@H](Cc1ccccc1)NC(=O)OC(C)(C)C)Cc2ccccc2)C(=O)NCc3ccccc3 | Protease |
| CHEMBL434342 | COc1cncc(c1)c2oc(nc2)C(C)(C)N3CCN(C[C@@H](O)C[C@@H](Cc4ocnn4)C(=O)N[C@H]5CCOC[C@H]5O)[C@@H](C3)C(=O)NCC(F)(F)F | Protease |
| CHEMBL52670 | CC1(C)SCN([C@@H]1C(=O)NCc2c(F)cccc2F)C(=O)[C@@H](O)C[C@@H](Cc3ccccc3)C(=O)N[C@@H]4[C@H](O)COc5ccccc45 | Protease |
| CHEMBL318003 | CC(C)(C)OC(=O)N[C@@H](Cc1ccccc1)[C@@H](O)C[C@@H](Cc2ccccc2)C(=O)N[C@H]3[C@H](O)Cc4ccccc34 | Protease |
| CHEMBL268765 | CC(=O)N[C@@H](C[S+]([O-])c1ccc2ccccc2n1)C(=O)N[C@@H](Cc3ccccc3)[C@H](O)Cc4ccccc4C(=O)NC(C)(C)C | Protease |
| CHEMBL325181 | CC(C)(C)NC(=O)[C@@H]1CN(Cc2cnc3occc3c2Cl)CCN1C[C@@H](O)C[C@@H](Cc4ccccc4)C(=O)NC5[C@H](O)Cc6ccccc56 | Protease |
| CHEMBL1790766 | CC[C@H](C)[C@H](NC(=O)[C@@H](C[C@H](O)[C@H](Cc1ccccc1)NC(=O)OC(C)(C)C)Cc2ccccc2)C(=O)NC(=O)C(N)Cc3ccccc3 | Protease |
| CHEMBL108918 | CC(C)(C)OC(=O)N[C@@H](Cc1ccccc1)[C@@H](O)C[C@@H](CSc2ccccc2)C(=O)N[C@@H]3[C@H](O)Cc4ccccc34 | Protease |
| CHEMBL410311 | CC(C)[C@@H]1[C@@H](CCS1(=O)=O)OC(=O)N[C@@H](Cc2ccccc2)[C@H](O)CN3CCN(Cc4ccc(Br)s4)C[C@H]3C(=O)NC(C)(C)C | Protease |
| CHEMBL105319 | CC(C)(C)OC(=O)N[C@@H](Cc1ccccc1)[C@@H](O)C[C@@H](CSc2ccccc2)C(=O)N[C@H]3[C@H](O)Cc4ccccc34 | Protease |
| CHEMBL351834 | CC(C)(C)NC(=O)[C@@H]1C[C@@H]2CCCC[C@@H]2CN1C[C@@H](O)[C@H](CSc3ccc4ccccc4c3)NC(=O)[C@H](CC(=O)N)NC(=O)c5ccc6ccccc6n5 | Protease |
| CHEMBL164045 | CC(C)(C)NC(=O)c1ccccc1C[C@@H](O)[C@H](CSc2ccc3ccccc3c2)NC(=O)[C@H](CC(=O)N)NC(=O)c4ccc5ccccc5n4 | Protease |
| CHEMBL21226 | CC(C)(C)OC(=O)N[C@@H](Cc1ccccc1)[C@@H](O)C[C@@H](Cc2ccccc2)C(=O)NC3[C@@H](O)Cc4ccccc34 | Protease |
| CHEMBL137925 | CC(C)(C)OC(=O)NC(Cc1ccccc1)[C@@H](O)CC(CSc2ccccc2)C(=O)NC3[C@@H](O)Cc4ccccc34 | Protease |
| CHEMBL296484 | CC(C)(C)OC(=O)N[C@@H](Cc1ccccc1)[C@@H](O)C[C@@H](Cc2ccc(cc2)C(F)(F)F)C(=O)N[C@@H]3[C@H](O)Cc4ccccc34 | Protease |
| CHEMBL337315 | CC(C)(C)OC(=O)N[C@@H](Cc1ccccc1)[C@@H](O)C[C@@H](Cc2ccc(cc2)C(F)(F)F)C(=O)NC3[C@@H](O)Cc4ccccc34 | Protease |
| CHEMBL358228 | CC(C)(C)NC(=O)[C@@H]1CN(Cc2ncc(s2)c3ccccc3)CCN1C[C@@H](O)C[C@@H](Cc4ccccc4)C(=O)NC5[C@H](O)Cc6ccccc56 | Protease |
| CHEMBL95546 | CC(C)[C@H]1SCC[C@H]1OC(=O)N[C@@H](Cc2ccccc2)[C@H](O)CN3C[C@H]4CCS[C@H]4C[C@H]3C(=O)NC(C)(C)C | Protease |
| CHEMBL263938 | CC(C)(C)OC(=O)N[C@@H](Cc1ccccc1)[C@@H](O)C[C@@H](Cc2ccc(cc2)C(F)(F)F)C(=O)N[C@H]3[C@H](O)Cc4ccccc34 | Protease |
| CHEMBL112257 | CSc1ncccc1CN2CCN(C[C@@H](O)C[C@@H](Cc3ccccc3)C(=O)NC4[C@H](O)Cc5ccccc45)[C@@H](C2)C(=O)NC(C)(C)C | Protease |
| CHEMBL342700 | CC(C)(C)OC(=O)NC(Cc1ccccc1)[C@@H](O)CC(Cc2ccc(cc2)[N+](=O)[O-])C(=O)NC3[C@@H](O)Cc4ccccc34 | Protease |
| CHEMBL295904 | CC(C)(C)OC(=O)N[C@@H](Cc1ccccc1)[C@@H](O)C[C@@H](Cc2ccc(cc2)[N+](=O)[O-])C(=O)N[C@@H]3[C@H](O)Cc4ccccc34 | Protease |
| CHEMBL432633 | CCOC(=O)c1cnc2oc(CN3CCN(CC(O)CC(Cc4ccccc4)C(=O)NC5C(O)Cc6ccccc56)C(C3)C(=O)NC(C)(C)C)cc2c1 | Protease |
| CHEMBL322897 | CC(C)(C)OC(=O)N[C@@H](Cc1ccccc1)[C@@H](O)C[C@@H](Cc2ccc(cc2)[N+](=O)[O-])C(=O)N[C@H]3[C@H](O)Cc4ccccc34 | Protease |
| CHEMBL275237 | CC(=O)N[C@@H](CS(=O)(=O)c1ccc2ccccc2n1)C(=O)N[C@@H](Cc3ccccc3)[C@H](O)Cc4ccccc4C(=O)NC(C)(C)C | Protease |
| CHEMBL99336 | C[C@@H]1OCC[C@@H]1OC(=O)N[C@@H](CSc2ccccc2)[C@H](O)CN3C[C@H]4CCS[C@H]4C[C@H]3C(=O)NC(C)(C)C | Protease |
| CHEMBL298393 | Cc1cccc(C)c1CNC(=O)[C@H]2N(CSC2(C)C)C(=O)[C@@H](O)C[C@@H](Cc3ccccc3)C(=O)N[C@@H]4[C@H](O)COc5ccccc45 | Protease |
| CHEMBL305814 | CC(C)C1(CCc2cc[nH]n2)CC(=O)C(=C(O)O1)Sc3cc(C)c(CO)cc3C(C)(C)C | Protease |
| CHEMBL139804 | Cc1ccc(C[C@H](C[C@H](O)[C@H](Cc2ccccc2)NC(=O)OC(C)(C)C)C(=O)NC3[C@@H](O)Cc4ccccc34)cc1 | Protease |
| CHEMBL265516 | Cc1ccc(C[C@H](C[C@H](O)[C@H](Cc2ccccc2)NC(=O)OC(C)(C)C)C(=O)N[C@@H]3[C@H](O)Cc4ccccc34)cc1 | Protease |
| CHEMBL295924 | CC(C)(C)OC(=O)N[C@@H](Cc1ccccc1)[C@@H](O)C[C@@H](C\C=C\c2ccc(CCN3CCOCC3)cc2)C(=O)N[C@@H]4[C@H](O)Cc5ccccc45 | Protease |
| CHEMBL103821 | Cc1ccc(C[C@H](C[C@H](O)[C@H](Cc2ccccc2)NC(=O)OC(C)(C)C)C(=O)N[C@H]3[C@H](O)Cc4ccccc34)cc1 | Protease |
| CHEMBL262714 | CC(C)(C)NC(=O)c1ccccc1C[C@@H](O)[C@H](Cc2ccccc2)NC(=O)[C@H](CS(=O)(=O)c3ccc4ccccc4n3)NS(=O)(=O)C | Protease |
| CHEMBL277985 | CC(=O)N[C@H](CS(=O)(=O)c1ccc2ccccc2c1)C(=O)N[C@@H](Cc3ccccc3)[C@H](O)Cc4ccccc4C(=O)NC(C)(C)C | Protease |
| CHEMBL98710 | Cc1c(O)cccc1C(=O)N[C@@H](CSc2ccccc2)[C@H](O)CN3C[C@H]4CCS[C@H]4C[C@H]3C(=O)NC(C)(C)C | Protease |
| CHEMBL325885 | CC(C)(C)NC(=O)[C@@H]1CN(CCN1C[C@@H](O)C[C@@H](Cc2ccccc2)C(=O)NC3[C@H](O)Cc4ccccc34)S(=O)(=O)c5cccnc5 | Protease |
| CHEMBL326764 | O[C@@H]1[C@@H](O)[C@@H](OCc2cncs2)[C@@H](Cc3ccccc3)S(=O)(=O)[C@H](Cc4ccccc4)[C@@H]1OCc5cncs5 | Protease |
| CHEMBL324084 | CC(C)(C)NC(=O)[C@@H]1CN(Cc2ccccc2)CCN1C[C@@H](O)C[C@@H](Cc3ccccc3)C(=O)NC4[C@H](O)Cc5ccccc45 | Protease |
| CHEMBL308780 | CC(C)(C)OC(=O)N[C@H](Cc1ccccc1)C(O)C[C@H](Cc2ccccc2)C(=O)NC3[C@H](O)Cc4ccccc34 | Protease |
| CHEMBL296493 | CC(C)(C)OC(=O)N[C@@H](Cc1ccccc1)[C@@H](O)C[C@@H](Cc2ccc(N)cc2)C(=O)N[C@@H]3[C@H](O)Cc4ccccc34 | Protease |
| CHEMBL343053 | CC(C)(C)OC(=O)NC(Cc1ccccc1)[C@@H](O)CC(Cc2ccc(N)cc2)C(=O)NC3[C@@H](O)Cc4ccccc34 | Protease |
| CHEMBL317868 | CC(C)(C)OC(=O)N[C@@H](Cc1ccccc1)[C@@H](O)C[C@@H](Cc2ccc(N)cc2)C(=O)N[C@H]3[C@H](O)Cc4ccccc34 | Protease |
| CHEMBL326543 | CC(C)(C)NC(=O)[C@@H]1CN(Cc2ccc(F)cc2F)CCN1C[C@@H](O)C[C@@H](Cc3ccccc3)C(=O)NC4[C@H](O)Cc5ccccc45 | Protease |
| CHEMBL324516 | CC(C)(C)NC(=O)C1CN(Cc2oc3ncccc3c2)CCN1CC(O)CC(Cc4ccccc4)C(=O)NC5C(O)Cc6ccccc56 | Protease |
| CHEMBL366293 | CCCc1oc(C[C@H](C[C@H](O)CN2CCN(C[C@H]2C(=O)NCC(F)(F)F)C(C)(C)c3oc(cn3)c4cncc(OC)c4)C(=O)N[C@H]5CCOC[C@H]5O)nn1 | Protease |
| CHEMBL301269 | CC(C)[C@@H]1[C@@H](CCS1(=O)=O)OC(=O)N[C@@H](Cc2ccccc2)[C@H](O)CN3CCN(Cc4cc5sccc5s4)C[C@H]3C(=O)NC(C)(C)C | Protease |
| CHEMBL276379 | CC(=O)N[C@@H](CS(=O)(=O)c1ccc2ccccc2c1)C(=O)N[C@@H](Cc3ccccc3)[C@H](O)Cc4ccccc4C(=O)NC(C)(C)C | Protease |
| CHEMBL101560 | O[C@@H](C[C@@H](Cc1ccccc1)C(=O)N[C@@H]2[C@H](O)Cc3ccccc23)[C@H](Cc4ccccc4)NC(=O)OC5CCCC5 | Protease |
| CHEMBL381583 | CC(C)CN([C@H](CO)CCCCNC(=O)[C@@H](NC(=O)c1ccc(c(O)c1)[N+](=O)[O-])C(c2ccccc2)c3ccccc3)S(=O)(=O)c4ccc(N)cc4 | Protease |
| CHEMBL274186 | CC(C)(C)NC(=O)c1ccccc1C[C@@H](O)[C@H](Cc2ccccc2)NC(=O)[C@H](CS(=O)(=O)c3ccc(Cl)cc3)NS(=O)(=O)C | Protease |
| CHEMBL78261 | CC(C)C1(CCc2cocc2)CC(=O)C(=C(O)O1)Sc3cc(C)c(CO)cc3C(C)(C)C | Protease |
| CHEMBL352145 | CC(C)(C)NC(=O)c1ccccc1S(=O)(=O)C[C@@H](O)[C@H](Cc2ccccc2)NC(=O)[C@H](CC(=O)N)NC(=O)c3ccc4ccccc4n3 | Protease |
| CHEMBL77431 | CC(C)C1(CCc2cccs2)CC(=O)C(=C(O)O1)Sc3cc(C)c(CO)cc3C(C)(C)C | Protease |
| CHEMBL297045 | CC(C)(C)OC(=O)N[C@@H](Cc1ccc(O)cc1)[C@@H](O)C[C@@H](Cc2ccccc2)C(=O)NC3[C@H](O)Cc4ccccc34 | Protease |
| CHEMBL115080 | Cc1nn(C)c2ncc(CN3CCN(C[C@@H](O)C[C@@H](Cc4ccccc4)C(=O)NC5[C@H](O)Cc6ccccc56)[C@@H](C3)C(=O)NC(C)(C)C)cc12 | Protease |
| CHEMBL2062139 | CC(C)(C)NC(=O)[C@@H]1CN(CCN1C[C@@H](O)C[C@@H](Cc2ccccc2)C(=O)N[C@@H]3[C@H](O)Cc4ccccc34)C(=O)OCc5ccccc5 | Protease |
| CHEMBL2062137 | CC(C)(C)NC(=O)[C@@H]1CN(Cc2cnc3occc3c2)CCN1C[C@@H](O)C[C@@H](Cc4ccccc4)C(=O)N[C@@H]5[C@H](O)Cc6ccccc56 | Protease |
| CHEMBL291195 | CC(C)C[C@H](NC(=O)[C@H](CCCc1ccccc1)C[C@H](O)[C@H](Cc2ccccc2)NC(=O)OC(C)(C)C)C(=O)N[C@@H](Cc3ccccc3)C(=O)N | Protease |
| CHEMBL115 | CC(C)(C)NC(=O)[C@@H]1CN(Cc2cccnc2)CCN1C[C@@H](O)C[C@@H](Cc3ccccc3)C(=O)N[C@@H]4[C@H](O)Cc5ccccc45 | Protease |
| CHEMBL82245 | CC(C)(N1CCN(C[C@@H](O)C[C@@H](Cc2ccccc2)C(=O)N[C@@H]3[C@H](O)COc4ccccc34)[C@@H](C1)C(=O)NC5CCCC5)c6oc7ccncc7c6 | Protease |
| CHEMBL429722 | CC(C)C(NC(=O)OCc1ccccc1)C(=O)N[C@@H](Cc2ccccc2)[C@@H](O)[C@H](O)[C@H](Cc3ccccc3)NC(=O)C(NC(=O)OCc4ccccc4)C(C)C | Protease |
| CHEMBL11421 | CC(C)(C)NC(=O)c1ccccc1C[C@@H](O)[C@H](Cc2ccccc2)NC(=O)[C@H](C[S+]([O-])c3ccc(F)cc3)NS(=O)(=O)C | Protease |
| CHEMBL314758 | CC(C)[C@@H](NC(=O)OCc1ccccc1)C(=O)N[C@@H](Cc2ccccc2)[C@H](O)[C@@H](O)[C@H](Cc3ccccc3)NC(=O)[C@@H](NC(=O)OCc4ccccc4)C(C)C | Protease |
| CHEMBL113826 | CC(C)(C)NC(=O)[C@@H]1CN(Cc2ccncc2)CCN1C[C@@H](O)C[C@@H](Cc3ccccc3)C(=O)NC4[C@H](O)Cc5ccccc45 | Protease |
| CHEMBL325354 | CC(C)(C)NC(=O)[C@@H]1CN(Cc2oc3cnccc3c2)CCN1C[C@@H](O)C[C@@H](Cc4ccccc4)C(=O)NC5[C@H](O)Cc6ccccc56 | Protease |
| CHEMBL74472 | CC(C)[C@H](NC(=O)c1cnc2ccccc2n1)C(=O)N[C@@H](Cc3ccccc3)[C@H](O)CN4C[C@H]5CCCC[C@H]5C[C@H]4C(=O)NC(C)(C)C | Protease |
| CHEMBL384210 | CC(C)CN([C@H](CO)CCCCNC(=O)[C@@H](NC(=O)OCc1ccncc1)C(c2ccccc2)c3ccccc3)S(=O)(=O)c4ccc(N)cc4 | Protease |
| CHEMBL378829 | CC(C)CN([C@H](CO)CCCCNC(=O)[C@@H](NC(=O)c1cnccc1C)C(c2ccccc2)c3ccccc3)S(=O)(=O)c4ccc(N)cc4 | Protease |
| CHEMBL364552 | COc1cncc(c1)c2oc(nc2)C(C)(C)N3CCN(C[C@@H](O)C[C@@H](Cc4oc(C)nn4)C(=O)N[C@H]5CCOC[C@H]5O)[C@@H](C3)C(=O)NCC(F)(F)F | Protease |
| CHEMBL310417 | CC(C)CNC(=O)[C@@H]1CN(CCN1C[C@@H](O)C[C@@H](Cc2ccccc2)C(=O)N[C@@H]3[C@H](O)COc4ccccc34)C(C)(C)c5oc6ccncc6c5 | Protease |
| CHEMBL378134 | CC(C)CN([C@H](CO)CCCCNC(=O)[C@@H](NC(=O)c1cccc(O)c1)C(c2ccccc2)c3ccccc3)S(=O)(=O)c4ccc(N)cc4 | Protease |
| CHEMBL73880 | CC(C)(C)NC(=O)[C@H]1C[C@@H]2CCCC[C@@H]2CN1CC(O)[C@H](Cc3ccccc3)NC(=O)[C@H](CC(=O)N)NC(=O)c4ccc5ccccc5n4 | Protease |
| CHEMBL49002 | CC(C)(C)NC(=O)[C@@H]1C[C@@H]2CCCC[C@@H]2CN1C[C@@H](O)[C@H](Cc3ccccc3)NC(=O)[C@@H](C[S+]([O-])c4ccc5ccccc5c4)NS(=O)(=O)C | Protease |
| CHEMBL377826 | CC(C)CN([C@H](CO)CCCCNC(=O)[C@@H](NC(=O)c1cnccc1N)C(c2ccccc2)c3ccccc3)S(=O)(=O)c4ccc(N)cc4 | Protease |
| CHEMBL208879 | CC(C)CN([C@H](CO)CCCCNC(=O)[C@@H](NC(=O)c1cccnc1C)C(c2ccccc2)c3ccccc3)S(=O)(=O)c4ccc(N)cc4 | Protease |
| CHEMBL321949 | C[C@H]1[C@H](CCS1(=O)=O)OC(=O)N[C@@H](CSc2ccccc2)[C@H](O)CN3C[C@H]4CCS[C@H]4C[C@H]3C(=O)NC(C)(C)C | Protease |
| CHEMBL441528 | CC(C)CN([C@H](CO)CCCCNC(=O)[C@@H](NC(=O)OC(C)(C)C)C(c1ccccc1)c2ccccc2)S(=O)(=O)c3ccc(N)cc3 | Protease |
| CHEMBL1735 | CC(C)(C)NC(=O)[C@@H]1CN(Cc2cccnc2)CCN1C[C@@H](O)C[C@@H](Cc3ccccc3)C(=O)N[C@@H]4[C@H](O)Cc5ccccc45.OS(=O)(=O)O | Protease |
| CHEMBL265975 | CC(C)C1(CCc2cscc2C)CC(=O)C(=C(O)O1)Sc3cc(C)c(CO)cc3C(C)(C)C | Protease |
| CHEMBL289195 | CC(C)C[C@H](NC(=O)[C@@H](C[C@H](O)[C@H](Cc1ccccc1)NC(=O)OC(C)(C)C)Cc2ccccc2)C(=O)N[C@@H](Cc3ccccc3)C(=O)N | Protease |
| CHEMBL97585 | CC(C)[C@@H]1[C@@H](CCS1(=O)=O)OC(=O)N[C@@H](CSc2ccccc2)[C@H](O)CN3C[C@H]4CCS[C@H]4C[C@H]3C(=O)NC(C)(C)C | Protease |
| CHEMBL99357 | CC(=O)N[C@H](CS(=O)(=O)c1cccc2cccnc12)C(=O)N[C@@H](Cc3ccccc3)[C@H](O)CN4C[C@H]5CCS[C@H]5C[C@H]4C(=O)NC(C)(C)C | Protease |
| CHEMBL75363 | CC(C)C1(CCc2ccc(N)cc2)CC(=O)C(=C(O)O1)Sc3cc(C)c(CO)cc3C(C)(C)C | Protease |
| CHEMBL298788 | CC(C)C[C@H](NC(=O)[C@@H](C[C@H](O)[C@H](Cc1ccccc1)NC(=O)OC(C)(C)C)Cc2ccc(cc2)c3ccccc3)C(=O)N[C@@H](Cc4ccccc4)C(=O)N | Protease |
| CHEMBL49544 | CC(C)(C)OC(=O)N[C@@H](Cc1ccccc1)[C@@H](O)C[C@@H](Cc2ccc(CCCN3CCOCC3)cc2)C(=O)N[C@@H]4[C@H](O)Cc5ccccc45 | Protease |
| CHEMBL379751 | CC(C)CN([C@H](CO)CCCCNC(=O)[C@@H](NC(=O)c1ccc[nH]1)C(c2ccccc2)c3ccccc3)S(=O)(=O)c4ccc(N)cc4 | Protease |
| CHEMBL378557 | CC(C)CN([C@H](CO)CCCCNC(=O)[C@@H](NC(=O)C1CC1)C(c2ccccc2)c3ccccc3)S(=O)(=O)c4ccc(N)cc4 | Protease |
| CHEMBL11143 | CC(C)(C)NC(=O)c1ccccc1C[C@@H](O)[C@H](Cc2ccccc2)NC(=O)[C@H](CS(=O)(=O)c3cccc4ccccc34)NS(=O)(=O)C | Protease |
| CHEMBL95413 | CC(C)[C@H](NC(=O)[C@@H](C[C@H](O)[C@H](Cc1ccccc1)NC(=O)OC(C)(C)C)Cc2ccccc2)C(=O)NCCO | Protease |
| CHEMBL379162 | CC(C)CN([C@H](CO)CCCCNC(=O)[C@@H](NC(=O)N(C)C)C(c1ccccc1)c2ccccc2)S(=O)(=O)c3ccc(N)cc3 | Protease |
| CHEMBL202345 | O[C@@H](C[C@]1(Cc2ccccc2)N=CC(=C1O)C3C4OC(=O)N[C@@H]4c5ccccc35)[C@H](Cc6ccccc6)NC(=O)O[C@H]7CCOC7 | Protease |
| CHEMBL296137 | CC(C)(C)OC(=O)N[C@@H](Cc1ccccc1)[C@@H](O)C[C@@H](Cc2ccc(F)cc2)C(=O)N[C@@H]3[C@H](O)Cc4ccccc34 | Protease |
| CHEMBL11116 | CC(C)(C)NC(=O)c1ccccc1C[C@@H](O)[C@H](Cc2ccccc2)NC(=O)[C@H](C[S+]([O-])c3cccc4ccccc34)NS(=O)(=O)C | Protease |
| CHEMBL306963 | CC(C)C1(CCc2nc(C)cs2)CC(=O)C(=C(O)O1)Sc3cc(C)c(CO)cc3C(C)(C)C | Protease |
| CHEMBL2062158 | CC(C)(C)OC(=O)N[C@@H](Cc1ccccc1)[C@@H](O)C[C@@H](Cc2ccc(OCCN3CCOCC3)cc2)C(=O)N[C@@H]4[C@H](O)Cc5ccccc45 | Protease |
| CHEMBL205968 | CC(C)CN([C@H](CO)CCCCNC(=O)[C@@H](NC(=O)c1ccccn1)C(c2ccccc2)c3ccccc3)S(=O)(=O)c4ccc(N)cc4 | Protease |
| CHEMBL98129 | CCC(C)[C@H](NC(=O)[C@@H](C[C@H](O)[C@H](Cc1ccccc1)NC(=O)OC(C)(C)C)Cc2ccccc2)C(=O)NCCO | Protease |
| CHEMBL97253 | CC(C)[C@H](NC(=O)[C@@H](C[C@H](O)[C@H](Cc1ccccc1)NC(=O)OC(C)(C)C)Cc2ccccc2)C(=O)NCCN | Protease |
| CHEMBL406460 | O[C@@H](C[C@@H](Cc1cccnc1)C(=O)N[C@@H]2[C@H](O)COc3ccccc23)CN4CCN(Cc5ccn(c5)c6ccccc6C#N)C[C@H]4C(=O)NCC(F)(F)F | Protease |
| CHEMBL114834 | CC(C)(C)NC(=O)[C@@H]1CN(Cc2nc3ccccc3[nH]2)CCN1C[C@@H](O)C[C@@H](Cc4ccccc4)C(=O)NC5[C@H](O)Cc6ccccc56 | Protease |
| CHEMBL47712 | CC(C)(C)OC(=O)N[C@@H](Cc1ccccc1)[C@@H](O)C[C@@H](Cc2ccc(OCCN3CCSCC3)cc2)C(=O)NC4[C@H](O)Cc5ccccc45 | Protease |
| CHEMBL98289 | Cc1cc(O)cc(C)c1CNC(=O)[C@@H](C[C@@H](O)CN2CCN(C[C@@H]2C(=O)NC(C)(C)C)S(=O)(=O)c3cccs3)Cc4ccccc4 | Protease |
| CHEMBL60697 | CC(C)[C@@H]1[C@@H](CCS1(=O)=O)OC(=O)N[C@@H](Cc2ccccc2)[C@H](O)CN3CCN(Cc4csc5ccc(Cl)cc45)C[C@H]3C(=O)NC(C)(C)C | Protease |
| CHEMBL319053 | CC(C)(C)NC(=O)[C@@H]1CN(Cc2c(F)cccc2F)CCN1C[C@@H](O)C[C@@H](Cc3ccncc3)C(=O)N[C@@H]4[C@H](O)Cc5ccccc45 | Protease |
| CHEMBL361062 | COc1cncc(c1)c2oc(nc2)C(C)(C)N3CCN(C[C@@H](O)C[C@@H](Cc4oc(nn4)c5ccccc5)C(=O)N[C@H]6CCOC[C@H]6O)[C@@H](C3)C(=O)NCC(F)(F)F | Protease |
| CHEMBL377192 | CC(C)CN([C@H](CO)CCCCNC(=O)[C@@H](NC(=O)OCc1cccnc1)C(c2ccccc2)c3ccccc3)S(=O)(=O)c4ccc(N)cc4 | Protease |
| CHEMBL116107 | CC(C)(C)NC(=O)[C@@H]1CN(Cc2ccc(Cl)nc2)CCN1C[C@@H](O)C[C@@H](Cc3ccccc3)C(=O)NC4[C@H](O)Cc5ccccc45 | Protease |
| CHEMBL151663 | CC(C)(C)NC(=O)[C@@H]1CN(Cc2oc3ccccc3c2)CCN1C[C@@H](O)C[C@@H](Cc4ccccc4)C(=O)NC5[C@H](O)Cc6ccccc56 | Protease |
| CHEMBL407999 | CC(C)[C@H](NC(=O)[C@H](Cc1cccc2ccccc12)CS(=O)(=O)C(C)(C)C)C(=O)N[C@H](Cc3ccccc3)P(=O)(O)[C@@H](Cc4ccccc4)NC(=O)[C@@H](NC(=O)[C@H](Cc5cccc6ccccc56)CS(=O)(=O)C(C)(C)C)C(C)C | Protease |
| CHEMBL76352 | CC(C)(C)NC(=O)[C@@H]1C[C@@H]2CCCC[C@@H]2CN1C[C@@H](O)[C@H](Cc3ccccc3)NC(=O)[C@@H](NC(=O)c4ccc5ccccc5n4)[C@H]6CCS(=O)(=O)C6 | Protease |
| CHEMBL379272 | CC(C)CN([C@H](CO)CCCCNC(=O)[C@@H](NC(=O)c1cnccc1O)C(c2ccccc2)c3ccccc3)S(=O)(=O)c4ccc(N)cc4 | Protease |
| CHEMBL168640 | COC(=O)N[C@@H](C(c1ccccc1)c2ccccc2)C(=O)NCCCC[C@@H](CO)N(CC(C)C)S(=O)(=O)c3ccc(N)cc3 | Protease |
| CHEMBL98096 | Cc1c(O)cccc1C(=O)N[C@@H](Cc2ccccc2)[C@H](O)CN3C[C@H]4CCS[C@H]4C[C@H]3C(=O)NC(C)(C)C | Protease |
| CHEMBL100089 | CC(C)(C)NC(=O)[C@@H]1C[C@@H]2CCCC[C@@H]2CN1C[C@@H](O)[C@H](Cc3ccccc3)NC(=O)[C@@H](CS(=O)(=O)c4ccc5ccccc5c4)NS(=O)(=O)C | Protease |
| CHEMBL209452 | COc1cc(ccc1O)C(=O)N[C@H](C(c2ccccc2)c3ccccc3)C(=O)NCCCC[C@@H](CO)N(CC(C)C)S(=O)(=O)c4ccc(N)cc4 | Protease |
| CHEMBL209023 | CC(C)CN([C@H](CO)CCCCNC(=O)[C@@H](N)C(c1ccccc1)c2ccccc2)S(=O)(=O)c3ccc(N)cc3 | Protease |
| CHEMBL316694 | CC(=O)N[C@H](CS(=O)(=O)c1ccc2ccccc2c1)C(=O)N[C@@H](Cc3ccccc3)[C@H](O)CN4C[C@H]5CCS[C@H]5C[C@H]4C(=O)NC(C)(C)C | Protease |
| CHEMBL432550 | CC(C)(N1CCN(C[C@@H](O)C[C@@H](Cc2ccccc2)C(=O)N[C@@H]3[C@H](O)COc4ccccc34)[C@@H](C1)C(=O)NCC5CC5)c6oc7ccncc7c6 | Protease |
| CHEMBL47465 | C[S+]([O-])c1ccc(C[C@H](C[C@H](O)[C@H](Cc2ccccc2)NC(=O)OC(C)(C)C)C(=O)N[C@@H]3[C@H](O)Cc4ccccc34)cc1 | Protease |
| CHEMBL207077 | CC(C)CN([C@H](CO)CCCCNC(=O)[C@@H](NC(=O)N1CCOCC1)C(c2ccccc2)c3ccccc3)S(=O)(=O)c4ccc(N)cc4 | Protease |
| CHEMBL380545 | CC(C)CN([C@H](CO)CCCCNC(=O)[C@@H](NC(=O)N1CCNCC1)C(c2ccccc2)c3ccccc3)S(=O)(=O)c4ccc(N)cc4 | Protease |
| CHEMBL54182 | Cc1ccccc1CNC(=O)[C@H]2N(C[C@@H](O)C[C@@H](Cc3ccccc3)C(=O)N[C@@H]4[C@H](O)COc5ccccc45)CSC2(C)C | Protease |
| CHEMBL2374440 | COC(=O)N[C@H](Cc1ccccc1c2ccccc2)C(=O)NCCCC[C@@H](CO)N(CC(C)C)S(=O)(=O)c3ccc(N)cc3 | Protease |
| CHEMBL430692 | CC(C)(C)OC(=O)N[C@@H](Cc1ccccc1)[C@@H](O)C[C@@H](Cc2ccncc2)C(=O)N[C@@H]3[C@H](O)Cc4ccccc34 | Protease |
| CHEMBL423358 | CC(C)(C)OC(=O)N[C@@H](Cc1ccccc1)[C@@H](O)C[C@@H](Cc2ccncc2)C(=O)NC3[C@@H](O)Cc4ccccc34 | Protease |
| CHEMBL438249 | CC(C)CN([C@H](CO)CCCCNC(=O)[C@@H](NC(=O)c1ccc(O)cc1)C(c2ccccc2)c3ccccc3)S(=O)(=O)c4ccc(N)cc4 | Protease |
| CHEMBL322214 | CC(C)(C)OC(=O)N[C@@H](Cc1ccccc1)[C@@H](O)C[C@@H](Cc2ccncc2)C(=O)N[C@H]3[C@H](O)Cc4ccccc34 | Protease |
| CHEMBL55586 | CC(C)[C@@H]1[C@@H](CCS1(=O)=O)OC(=O)N[C@@H](Cc2ccccc2)[C@H](O)CN3CCN(Cc4cc(C)nc(Cl)c4)C[C@H]3C(=O)NC(C)(C)C | Protease |
| CHEMBL326493 | CC(C)(C)NC(=O)[C@@H]1CN(Cc2ccccc2S)CCN1C[C@@H](O)C[C@@H](Cc3ccccc3)C(=O)NC4[C@H](O)Cc5ccccc45 | Protease |
| CHEMBL324881 | CC1(C)S[C@@H](N[C@H]1C(=O)NCCNC(=O)[C@@H]2N[C@H](SC2(C)C)[C@H](NC(=O)Cc3ccccn3)C(=O)NCc4ccccc4)[C@H](NC(=O)Cc5ccccn5)C(=O)NCc6ccccc6 | Protease |
| CHEMBL45457 | CC(C)(C)OC(=O)N[C@@H](Cc1ccc(OCCN2CCOCC2)cc1)[C@@H](O)C[C@@H](Cc3ccccc3)C(=O)NC4[C@H](O)Cc5ccccc45 | Protease |
| CHEMBL378194 | CC(C)CN([C@H](CO)CCCCNC(=O)[C@@H](NC(=O)c1ccc(O)c(c1)[N+](=O)[O-])C(c2ccccc2)c3ccccc3)S(=O)(=O)c4ccc(N)cc4 | Protease |
| CHEMBL76040 | CC(C)C1(CCc2nccs2)CC(=O)C(=C(O)O1)Sc3cc(C)c(CO)cc3C(C)(C)C | Protease |
| CHEMBL3085518 | CC(C)(C)NC(=O)[C@@H]1CCCN1C(=O)[C@@H](O)[C@H](Cc2ccccc2)NC(=O)[C@H](CC(=O)N)NC(=O)COc3cccc4ccccc34 | Protease |
| CHEMBL77041 | CC(C)C1(CCc2ccc(CO)s2)CC(=O)C(=C(O)O1)Sc3cc(C)c(CO)cc3C(C)(C)C | Protease |
| CHEMBL299821 | COCCN(CCCc1ccc(C[C@H](C[C@H](O)[C@H](Cc2ccccc2)NC(=O)OC(C)(C)C)C(=O)N[C@@H]3[C@H](O)Cc4ccccc34)cc1)CCOC | Protease |
| CHEMBL301260 | CC(C)[C@@H]1[C@@H](CCS1(=O)=O)OC(=O)N[C@@H](Cc2ccccc2)[C@H](O)CN3CCN(CCc4cccs4)C[C@H]3C(=O)NC(C)(C)C | Protease |
| CHEMBL306570 | CC(C)(C)NC(=O)[C@@H]1C[C@@H]2CCCC[C@@H]2CN1C[C@@H](O)[C@H](Cc3ccccc3)NC(=O)[C@@H](NC(=O)c4ccc5ccccc5n4)[C@H]6CCSC6 | Protease |
| CHEMBL323814 | O[C@@H]1[C@@H](O)[C@@H](OCc2ccccc2)[C@@H](Cc3ccccc3)S(=O)(=O)[C@H](Cc4ccccc4)[C@@H]1OCc5ccccc5 | Protease |
| CHEMBL47514 | CC(C)(C)OC(=O)N[C@@H](Cc1ccccc1)[C@@H](O)C[C@@H](Cc2c(F)c(F)c(F)c(F)c2F)C(=O)N[C@@H]3[C@H](O)Cc4ccccc34 | Protease |
| CHEMBL57566 | CC(C)[C@@H]1[C@@H](CCS1(=O)=O)OC(=O)N[C@@H](Cc2ccccc2)[C@H](O)CN3CCN(Cc4cccs4)C[C@H]3C(=O)NC(C)(C)C | Protease |
| CHEMBL317793 | CC(C)(C)NC(=O)[C@@H]1C[C@@H]2CCCC[C@@H]2CN1C[C@@H](O)[C@H](Cc3ccccc3)NC(=O)[C@@H](C[S+]([O-])c4ccc(F)cc4)NS(=O)(=O)C | Protease |
| CHEMBL322634 | CC(C)(C)OC(=O)N[C@@H](Cc1ccccc1)[C@@H](O)C[C@@H](Cc2c(F)c(F)c(F)c(F)c2F)C(=O)N[C@H]3[C@H](O)Cc4ccccc34 | Protease |
| CHEMBL52868 | CC(C)C[C@H](NC(=O)[C@@H](C[C@H](O)[C@@H](CC(=O)OC(C)(C)C)Cc1ccccc1)Cc2ccccc2)C(=O)N[C@@H](Cc3ccccc3)C(=O)N | Protease |
| CHEMBL416814 | CN(C)CCCc1ccc(C[C@H](C[C@H](O)[C@H](Cc2ccccc2)NC(=O)OC(C)(C)C)C(=O)N[C@@H]3[C@H](O)Cc4ccccc34)cc1 | Protease |
| CHEMBL262300 | CC(C)C[C@H](NC(=O)[C@@H](C[C@H](O)[C@H](Cc1ccccc1)NC(=O)OC(C)(C)C)Cc2ccccc2)C(=O)NC(=O)C(N)Cc3ccccc3 | Protease |
| CHEMBL81811 | CC(C)(N1CCN(C[C@@H](O)C[C@@H](Cc2ccccc2)C(=O)N[C@@H]3[C@H](O)COc4ccccc34)[C@@H](C1)C(=O)NC5CC5)c6oc7ccncc7c6 | Protease |
| CHEMBL342365 | CC(C)(C)OC(=O)N[C@@H](Cc1ccccc1)[C@@H](O)C[C@@H](Cc2c(F)c(F)c(F)c(F)c2F)C(=O)NC3[C@@H](O)Cc4ccccc34 | Protease |
| CHEMBL311514 | CCNC(=O)[C@@H]1CN(CCN1C[C@@H](O)C[C@@H](Cc2ccccc2)C(=O)N[C@@H]3[C@H](O)COc4ccccc34)C(C)(C)c5oc6ccncc6c5 | Protease |
| CHEMBL77281 | CC(C)[C@H](NC(=O)[C@@H](C[C@H](O)[C@H](Cc1ccccc1)NC(=O)OC(C)(C)C)Cc2ccccc2)C(=O)NCc3ccncc3 | Protease |
| CHEMBL333607 | Cc1ccc(CN2CCN(C[C@@H](O)C[C@@H](Cc3ccccc3)C(=O)NC4[C@H](O)Cc5ccccc45)[C@@H](C2)C(=O)NC(C)(C)C)cn1 | Protease |
| CHEMBL384211 | CC(C)CN([C@H](CO)CCCCNC(=O)[C@@H](NC(=O)c1ccccc1O)C(c2ccccc2)c3ccccc3)S(=O)(=O)c4ccc(N)cc4 | Protease |
| CHEMBL208724 | CC(C)CN([C@H](CO)CCCCNC(=O)[C@@H](NC(=O)c1ccncc1)C(c2ccccc2)c3ccccc3)S(=O)(=O)c4ccc(N)cc4 | Protease |
| CHEMBL78514 | CC(C)(N1CCN(C[C@@H](O)C[C@@H](Cc2ccccc2)C(=O)N[C@@H]3[C@H](O)COc4ccccc34)[C@@H](C1)C(=O)NCc5cccs5)c6oc7ccncc7c6 | Protease |
| CHEMBL261952 | CC(C)CN([C@H](CO)CCCCNC(=O)[C@@H](NC(=O)c1cccnc1)C(c2ccccc2)c3ccccc3)S(=O)(=O)c4ccc(N)cc4 | Protease |
| CHEMBL325946 | CC(C)(C)NC(=O)[C@@H]1CN(Cc2cnc3OCCc3c2)CCN1C[C@@H](O)C[C@@H](Cc4ccccc4)C(=O)NC5[C@H](O)Cc6ccccc56 | Protease |
| CHEMBL327564 | CC(C)[C@H](NC(=O)[C@@H](C[C@H](O)[C@H](Cc1ccccc1)NC(=O)OC(C)(C)C)Cc2ccccc2)C(=O)NCc3cccnc3 | Protease |
| CHEMBL109952 | OC(C[C@@H](Cc1ccccc1)C(=O)N[C@@H]2[C@H](O)Cc3ccccc23)C[C@@H](Cc4ccccc4)C(=O)N[C@@H]5[C@H](O)Cc6ccccc56 | Protease |
| CHEMBL325551 | CC(C)(C)OC(=O)N[C@@H](Cc1ccccc1)[C@@H](O)C[C@@H](Cc2ccccc2)C(=O)N[C@@H]3[C@H](O)CCc4ccccc34 | Protease |
| CHEMBL317446 | CC(C)(C)OC(=O)N[C@@H](Cc1ccccc1)[C@@H](O)C[C@@H](Cc2ccccc2)C(=O)N[C@H]3[C@H](O)CCc4ccccc34 | Protease |
| CHEMBL342135 | OC(C[C@@H](Cc1ccccc1)C(=O)NC2[C@@H](O)Cc3ccccc23)C[C@@H](Cc4ccccc4)C(=O)NC5[C@@H](O)Cc6ccccc56 | Protease |
| CHEMBL75082 | CC(C)[C@H](NC(=O)c1ccc2ccccc2n1)C(=O)N[C@@H](Cc3ccccc3)[C@H](O)CN4C[C@H]5CCCC[C@H]5C[C@H]4C(=O)NC(C)(C)C | Protease |
| CHEMBL282654 | CCC(C)C(NC(=O)[C@@H](C[C@H](O)[C@H](Cc1ccccc1)NC(=O)OC(C)(C)C)Cc2ccccc2)C(=O)NCc3ccccc3 | Protease |
| CHEMBL430868 | CCC(C)[C@H](NC(=O)[C@@H](C[C@H](O)[C@H](Cc1ccccc1)NC(=O)OC(C)(C)C)Cc2ccccc2)C(=O)NCc3ccccc3 | Protease |
| CHEMBL263423 | CC(C)(C)NC(=O)[C@@H]1C[C@@H]2SCC[C@@H]2CN1C[C@@H](O)[C@H](Cc3ccccc3)NC(=O)[C@@H](CS(=O)(=O)c4cccc5cccnc45)NS(=O)(=O)C | Protease |
| CHEMBL420466 | CC(C)(C)OC(=O)N[C@@H](Cc1ccccc1)[C@@H](O)C[C@@H](Cc2ccc(I)cc2)C(=O)N[C@H]3[C@H](O)Cc4ccccc34 | Protease |
| CHEMBL296855 | CC(C)(C)OC(=O)N[C@@H](Cc1ccccc1)[C@@H](O)C[C@@H](Cc2ccc(I)cc2)C(=O)N[C@@H]3[C@H](O)Cc4ccccc34 | Protease |
| CHEMBL308615 | CC(C)C1(CCc2c[nH]c3ccccc23)CC(=O)C(=C(O)O1)Sc4cc(C)c(CO)cc4C(C)(C)C | Protease |
| CHEMBL327565 | CC(C)[C@H](NC(=O)[C@@H](C[C@H](O)[C@H](Cc1ccccc1)NC(=O)OC(C)(C)C)Cc2ccccc2)C(=O)NCc3ccccn3 | Protease |
| CHEMBL99214 | C[C@@H]1[C@@H](CCS1(=O)=O)OC(=O)N[C@@H](CSc2ccccc2)[C@H](O)CN3C[C@H]4CCS[C@H]4C[C@H]3C(=O)NC(C)(C)C | Protease |
| CHEMBL301193 | CCc1ccc(CCN2CCN(C[C@@H](O)[C@H](Cc3ccccc3)NC(=O)O[C@@H]4CCS(=O)(=O)[C@@H]4C(C)C)[C@@H](C2)C(=O)NC(C)(C)C)s1 | Protease |
| CHEMBL276656 | CC(C)(C)NC(=O)c1ccccc1C[C@@H](O)[C@H](Cc2ccccc2)NC(=O)[C@H](CS(=O)(=O)c3ccc(F)cc3)NS(=O)(=O)C | Protease |
| CHEMBL73875 | CCCC[C@H](CN[C@H](CCCC)C(=O)N[C@@H](CCC(=O)N)C(=O)N[C@@H](CCCN=C(N)N)C(=O)N)NC(=O)[C@@H](NC(=O)[C@@H](NC(=O)C)[C@@H](C)O)[C@@H](C)CC | Protease |
| CHEMBL311555 | CC(C)C1(CCc2occc2)CC(=O)C(=C(O)O1)Sc3cc(C)c(CO)cc3C(C)(C)C | Protease |
| CHEMBL116867 | Cc1cc(O)c(C)c(c1)C(=O)N[C@@H](Cc2ccc3ccccc3c2)[C@H](O)C(=O)N4C[C@@H](Cl)C[C@H]4C(=O)NC(C)(C)C | Protease |
| CHEMBL113958 | Cc1c(O)cccc1C(=O)N[C@@H](Cc2ccccc2)[C@H](O)C(=O)N3C[C@@H](Cl)C[C@H]3C(=O)NC(C)(C)C | Protease |
| CHEMBL75070 | CC(C)c1ncsc1CCC2(CC(=O)C(=C(O)O2)Sc3cc(C)c(CO)cc3C(C)(C)C)C(C)C | Protease |
| CHEMBL115176 | CC(C)(C)NC(=O)[C@@H]1CN(CC(=O)OC(C)(C)C)CCN1C[C@@H](O)C[C@@H](Cc2ccccc2)C(=O)NC3[C@H](O)Cc4ccccc34 | Protease |
| CHEMBL95116 | CC(C)[C@H](NC(=O)[C@@H](C[C@H](O)[C@H](Cc1ccccc1)NC(=O)OC(C)(C)C)Cc2ccccc2)C(=O)NCC(O)CN3CCOCC3 | Protease |
| CHEMBL97825 | CCC(C)[C@H](NC(=O)[C@@H](C[C@H](O)[C@H](Cc1ccccc1)NC(=O)OC(C)(C)C)Cc2ccccc2)C(=O)NCc3ncc[nH]3 | Protease |
| CHEMBL295482 | COCCN(CCOC)CCOc1ccc(C[C@H](C[C@H](O)[C@H](Cc2ccccc2)NC(=O)OC(C)(C)C)C(=O)NC3[C@H](O)Cc4ccccc34)cc1 | Protease |
| CHEMBL98355 | CC(C)(C)OC(=O)N[C@@H](Cc1ccccc1)[C@@H](O)C[C@@H](Cc2ccccc2)C(=O)N[C@H](C(=O)N)c3ccccc3 | Protease |
| CHEMBL102664 | CC1(C)S[C@@H](N[C@H]1C(=O)NCCNC(=O)[C@@H]2N[C@H](SC2(C)C)[C@H](NC(=O)Cc3ccccc3)C(=O)NCc4ccccc4)[C@H](NC(=O)Cc5ccccc5)C(=O)NCc6ccccc6 | Protease |
| CHEMBL72386 | CC(C)[C@H](NC(=O)c1ccc2ccccc2c1)C(=O)N[C@@H](Cc3ccccc3)[C@H](O)CN4CC5CCCCC5C[C@H]4C(=O)NC(C)(C)C | Protease |
| CHEMBL209878 | CC(C)CN([C@H](CO)CCCCNC(=O)[C@@H](NC(=O)c1ncccc1O)C(c2ccccc2)c3ccccc3)S(=O)(=O)c4ccc(N)cc4 | Protease |
| CHEMBL433072 | [I-].CC(C)(C)OC(=O)N[C@@H](Cc1ccccc1)[C@@H](O)C[C@@H](Cc2ccc(CCC[N+](C)(C)C)cc2)C(=O)N[C@@H]3[C@H](O)Cc4ccccc34 | Protease |
| CHEMBL74508 | CC(C)C1(CCc2cccnc2)CC(=O)C(=C(O)O1)Sc3cc(C)c(CO)cc3C(C)(C)C | Protease |
| CHEMBL151080 | CC(C)(C)NC(=O)[C@@H]1CN(Cc2oc3ccc(F)cc3c2)CCN1C[C@@H](O)C[C@@H](Cc4ccccc4)C(=O)NC5[C@H](O)Cc6ccccc56 | Protease |
| CHEMBL316717 | CC(C)[C@H]1[C@H](CCS1(=O)=O)OC(=O)N[C@@H](CSc2ccccc2)[C@H](O)CN3C[C@H]4CCS[C@H]4C[C@H]3C(=O)NC(C)(C)C | Protease |
| CHEMBL78327 | CC(C)C1(CCc2csc(N)n2)CC(=O)C(=C(O)O1)Sc3cc(C)c(CO)cc3C(C)(C)C | Protease |
| CHEMBL72497 | CCCCc1cc(CN2CCN(C[C@@H](O)[C@H](Cc3ccccc3)NC(=O)O[C@@H]4CCS(=O)(=O)[C@@H]4C(C)C)[C@@H](C2)C(=O)NC(C)(C)C)cc(CCCC)n1 | Protease |
| CHEMBL306932 | CC(C)C1(CCc2ccc3[nH]ccc3c2)CC(=O)C(=C(O)O1)Sc4cc(C)c(CO)cc4C(C)(C)C | Protease |
| CHEMBL266918 | CC(C)C(NC(=O)N(C)Cc1ccccn1)C(=O)N[C@@H](Cc2ccccc2)[C@@H](O)[C@@H](O)[C@H](Cc3ccccc3)NC(=O)C(NC(=O)N(C)Cc4ccccn4)C(C)C | Protease |
| CHEMBL68313 | CC(C)[C@@H]1NC(=O)CCCCCCOc2ccc(C[C@H](NC1=O)[C@H](O)CN3CC4CCCCC4C[C@H]3C(=O)NC(C)(C)C)cc2 | Protease |
| CHEMBL45928 | CC(C)(C)OC(=O)N[C@@H](Cc1ccccc1)[C@@H](O)C[C@@H](Cc2ccc(CCCN3CCSCC3)cc2)C(=O)N[C@@H]4[C@H](O)Cc5ccccc45 | Protease |
| CHEMBL331814 | CC(C)[C@@H](NC(=O)OCc1ccccn1)C(=O)N[C@@H](Cc2ccccc2)[C@@H](O)[C@H](O)[C@H](Cc3ccccc3)NC(=O)[C@@H](NC(=O)OCc4ccccn4)C(C)C | Protease |
| CHEMBL109992 | CC(C)[C@@H](NS(=O)(=O)CCc1ccccn1)C(=O)N[C@@H](Cc2ccccc2)[C@H](O)[C@H](O)[C@H](Cc3ccccc3)NC(=O)[C@@H](NS(=O)(=O)CCc4ccccn4)C(C)C | Protease |
| CHEMBL115978 | CC(C)[C@@H](NC(=O)N(C)Cc1cscn1)C(=O)N[C@@H](Cc2ccccc2)[C@@H](O)[C@H](O)[C@H](Cc3ccccc3)NC(=O)[C@@H](NC(=O)N(C)Cc4cscn4)C(C)C | Protease |
| CHEMBL324746 | CC(C)[C@@H](NC(=O)N(C)Cc1cscn1)C(=O)N[C@@H](Cc2ccccc2)[C@H](O)[C@H](O)[C@H](Cc3ccccc3)NC(=O)[C@@H](NC(=O)N(C)Cc4cscn4)C(C)C | Protease |
| CHEMBL322477 | CC(C)[C@@H](NS(=O)(=O)CCc1ccccn1)C(=O)N[C@@H](Cc2ccccc2)[C@@H](O)[C@H](O)[C@H](Cc3ccccc3)NC(=O)[C@@H](NS(=O)(=O)CCc4ccccn4)C(C)C | Protease |
| CHEMBL114972 | CC(C)[C@@H](NC(=O)N(C)Cc1cscn1)C(=O)N[C@@H](Cc2ccccc2)[C@@H](O)[C@@H](O)[C@H](Cc3ccccc3)NC(=O)[C@@H](NC(=O)N(C)Cc4cscn4)C(C)C | Protease |
| CHEMBL46062 | CN(C)CCOc1ccc(C[C@H](C[C@H](O)[C@H](Cc2ccccc2)NC(=O)OC(C)(C)C)C(=O)NC3[C@H](O)Cc4ccccc34)cc1 | Protease |
| CHEMBL1790545 | CC[C@@H](C)[C@H](NC(=O)[C@H](C(C)C)[C@@H](O)[C@H](O)[C@H](CC1CCCCC1)NC(=O)[C@H](Cc2c[nH]cn2)NC(=O)COc3cccc4ccccc34)C(=O)NCc5ccccn5 | Protease |
| CHEMBL3143349 | CC(C)C[C@H](NC(=O)[C@@H](C[C@H](O)[C@H](Cc1ccccc1)NC(=O)[C@H](Cc2ccccc2)NC(=O)[C@H](Cc3ccccc3)NC(=O)OC(C)(C)C)Cc4ccccc4)C(=O)N[C@@H](Cc5ccccc5)C(=O)N | Protease |
| CHEMBL324596 | CC(C)[C@@H](NC(=O)OCc1cccnc1)C(=O)N[C@@H](Cc2ccccc2)[C@@H](O)[C@@H](O)[C@H](Cc3ccccc3)NC(=O)[C@@H](NC(=O)OCc4cccnc4)C(C)C | Protease |
| CHEMBL323731 | CC(C)[C@@H](NC(=O)N(C)Cc1csc(N)n1)C(=O)N[C@@H](Cc2ccccc2)[C@H](O)[C@@H](O)[C@H](Cc3ccccc3)NC(=O)[C@@H](NC(=O)N(C)Cc4nccs4)C(C)C | Protease |
| CHEMBL269617 | CC(C)[C@@H](NC(=O)N(C)Cc1nccs1)C(=O)N[C@@H](Cc2ccccc2)[C@H](O)[C@@H](O)[C@H](Cc3ccccc3)NC(=O)[C@@H](NC(=O)N(C)Cc4ccccn4)C(C)C | Protease |
| CHEMBL113377 | CC(C)[C@@H](NC(=O)N(C)Cc1ccccn1)C(=O)N[C@@H](Cc2ccccc2)[C@@H](O)[C@@H](O)[C@H](Cc3ccccc3)NC(=O)[C@@H](NC(=O)N(C)Cc4ccccn4)C(C)C | Protease |
| CHEMBL101111 | COc1cc(CC(=O)N[C@@H](C(C)C)C(=O)N[C@H](C[C@H](O)[C@H](Cc2ccccc2)NC(=O)[C@@H](NC(=O)Cc3cc(OC)c(OC)c(OC)c3)C(C)C)Cc4ccccc4)cc(OC)c1OC | Protease |
| CHEMBL100412 | CC(C)[C@H](NC(=O)CSc1ncccn1)C(=O)N[C@H](C[C@H](O)[C@H](Cc2ccccc2)NC(=O)[C@@H](NC(=O)CSc3ncccn3)C(C)C)Cc4ccccc4 | Protease |
| CHEMBL37037 | CC(C)[C@]1(CCc2ccc(O)cc2)CC(=O)C(=C(O)O1)Sc3cc(C)c(CO)cc3C(C)(C)C | Protease |
| CHEMBL307582 | CC(C)C1(CCc2ccc(N)cc2)CC(=O)C(=C(O)O1)Sc3cc(C)c(OS(=O)(=O)N4CCN(C)CC4)cc3C(C)(C)C | Protease |
| CHEMBL283537 | CC(C)[C@H](NC(=O)OCc1ccccc1)C(=O)NC(Cc2ccc(OCc3ccccc3)cc2)C(=O)C(F)(F)C(=O)N[C@@H](COCc4ccccc4)c5ccccc5 | Protease |
| CHEMBL322941 | CC(C)[C@@H](NC(=O)OCc1cccnc1)C(=O)N[C@@H](Cc2ccccc2)[C@H](O)[C@H](O)[C@H](Cc3ccccc3)NC(=O)[C@@H](NC(=O)OCc4cccnc4)C(C)C | Protease |
| CHEMBL432806 | CC(C)[C@@H](NC(=O)N(C)Cc1nccs1)C(=O)N[C@@H](Cc2ccccc2)[C@H](O)[C@H](O)[C@H](Cc3ccccc3)NC(=O)[C@@H](NC(=O)N(C)Cc4nccs4)C(C)C | Protease |
| CHEMBL61756 | CC(C)[C@]1(CCc2ccc(O)cc2)CC(=O)C(=C(O)O1)Sc3sc4ccccc4c3C(C)(C)C | Protease |
| CHEMBL116 | CC(C)CN(C[C@@H](O)[C@H](Cc1ccccc1)NC(=O)O[C@H]2CCOC2)S(=O)(=O)c3ccc(N)cc3 | Protease |
| CHEMBL333601 | CC(C)[C@@H](NC(=O)N(C)Cc1cccnc1)C(=O)N[C@@H](Cc2ccccc2)[C@@H](O)[C@H](O)[C@H](Cc3ccccc3)NC(=O)[C@@H](NC(=O)N(C)Cc4cccnc4)C(C)C | Protease |
| CHEMBL323595 | CC(C)[C@@H](NC(=O)OCc1cccnc1)C(=O)N[C@@H](Cc2ccccc2)[C@@H](O)[C@H](O)[C@H](Cc3ccccc3)NC(=O)[C@@H](NC(=O)OCc4cccnc4)C(C)C | Protease |
| CHEMBL413390 | CC(C)[C@H](NC(=O)[C@H](Cc1cccc2ccccc12)CS(=O)(=O)C(C)(C)C)C(=O)N[C@H](Cc3ccccc3)P(=O)(N)[C@@H](Cc4ccccc4)NC(=O)[C@@H](NC(=O)[C@H](Cc5cccc6ccccc56)CS(=O)(=O)C(C)(C)C)C(C)C | Protease |
| CHEMBL304545 | CC(C)C(NC(=O)OCc1ccccc1)C(=O)N[C@H](CC[C@H](O)[C@H](Cc2ccccc2)NC(=O)C(NC(=O)OCc3ccccc3)C(C)C)Cc4ccccc4 | Protease |
| CHEMBL323918 | CC(C)[C@@H](NC(=O)N(C)Cc1nccs1)C(=O)N[C@@H](Cc2ccccc2)[C@@H](O)[C@H](O)[C@H](Cc3ccccc3)NC(=O)[C@@H](NC(=O)N(C)Cc4nccs4)C(C)C | Protease |
| CHEMBL333600 | CC(C)[C@@H](NC(=O)N(C)Cc1nc2ccccc2[nH]1)C(=O)N[C@@H](Cc3ccccc3)[C@H](O)[C@H](O)[C@H](Cc4ccccc4)NC(=O)[C@@H](NC(=O)N(C)Cc5nc6ccccc6[nH]5)C(C)C | Protease |
| CHEMBL324163 | CC(C)[C@@H](NC(=O)N(C)Cc1cscn1)C(=O)N[C@@H](Cc2ccccc2)[C@H](O)[C@@H](O)[C@H](Cc3ccccc3)NC(=O)[C@@H](NC(=O)N(C)Cc4ccccn4)C(C)C | Protease |
| CHEMBL112859 | CC(C)[C@@H](NC(=O)OCc1ccccn1)C(=O)N[C@@H](Cc2ccccc2)[C@H](O)[C@H](O)[C@H](Cc3ccccc3)NC(=O)[C@@H](NC(=O)OCc4ccccn4)C(C)C | Protease |
| CHEMBL320743 | CC(C)[C@@H](NC(=O)N(C)Cc1csc(N)n1)C(=O)N[C@@H](Cc2ccccc2)[C@@H](O)[C@H](O)[C@H](Cc3ccccc3)NC(=O)[C@@H](NC(=O)N(C)Cc4csc(N)n4)C(C)C | Protease |
| CHEMBL112958 | CC(C)[C@@H](NC(=O)OCc1ccccn1)C(=O)N[C@@H](Cc2ccccc2)[C@@H](O)[C@@H](O)[C@H](Cc3ccccc3)NC(=O)[C@@H](NC(=O)OCc4ccccn4)C(C)C | Protease |
| CHEMBL111388 | CC(C)[C@@H](NC(=O)N(C)Cc1cccnc1)C(=O)N[C@@H](Cc2ccccc2)[C@H](O)[C@@H](O)[C@H](Cc3ccccc3)NC(=O)[C@@H](NC(=O)N(C)Cc4ccccn4)C(C)C | Protease |
| CHEMBL317907 | CC(C)[C@H](NC(=O)CC1NC(=O)NC1=O)C(=O)N[C@H](C[C@H](O)[C@H](Cc2ccccc2)NC(=O)[C@@H](NC(=O)CC3NC(=O)NC3=O)C(C)C)Cc4ccccc4 | Protease |
| CHEMBL113225 | CC(C)[C@@H](NC(=O)c1cc2ccccc2[nH]1)C(=O)N[C@@H](Cc3ccccc3)[C@H](O)[C@H](O)[C@H](Cc4ccccc4)NC(=O)[C@@H](NC(=O)c5cc6ccccc6[nH]5)C(C)C | Protease |
| CHEMBL326620 | CC(C)[C@@H](NC(=O)N(C)Cc1csc(N)n1)C(=O)N[C@@H](Cc2ccccc2)[C@H](O)[C@@H](O)[C@H](Cc3ccccc3)NC(=O)[C@@H](NC(=O)N(C)Cc4ccccn4)C(C)C | Protease |
| CHEMBL324071 | Nc1cccc(CO[C@@H]2[C@H](O)[C@@H](O)[C@@H](OCc3cccc(N)c3)[C@@H](Cc4ccccc4)S(=O)(=O)[C@@H]2Cc5ccccc5)c1 | Protease |
| CHEMBL109976 | CC(C)[C@@H](NC(=O)OCc1ccccc1)C(=O)N[C@@H](Cc2ccccc2)[C@@H](O)[C@@H](O)[C@H](Cc3ccccc3)NC(=O)[C@@H](NC(=O)OCc4ccccc4)C(C)C | Protease |
| CHEMBL267793 | CC(C)[C@@H](NC(=O)N(C)Cc1csc(N)n1)C(=O)N[C@@H](Cc2ccccc2)[C@H](O)[C@@H](O)[C@H](Cc3ccccc3)NC(=O)[C@@H](NC(=O)N(C)Cc4cscn4)C(C)C | Protease |
| CHEMBL113451 | CC(C)[C@@H](NC(=O)\C=C\c1ccccn1)C(=O)N[C@@H](Cc2ccccc2)[C@H](O)[C@H](O)[C@H](Cc3ccccc3)NC(=O)[C@@H](NC(=O)\C=C\c4ccccn4)C(C)C | Protease |
| CHEMBL113355 | CC(C)[C@@H](NC(=O)N(C)Cc1nc2ccccc2[nH]1)C(=O)N[C@@H](Cc3ccccc3)[C@@H](O)[C@H](O)[C@H](Cc4ccccc4)NC(=O)[C@@H](NC(=O)N(C)Cc5nc6ccccc6[nH]5)C(C)C | Protease |
| CHEMBL282042 | CC(C)(C)NC(=O)[C@@H]1C[C@@H]2CCCC[C@@H]2CN1C[C@@H](O)[C@H](Cc3ccccc3)NC(=O)[C@H](CC(=O)N)NC(=O)c4ccc5ccccc5n4.CS(=O)(=O)O | Protease |
| CHEMBL112416 | CC(C)[C@@H](NC(=O)N(C)Cc1nc2ccccc2[nH]1)C(=O)N[C@@H](Cc3ccccc3)[C@@H](O)[C@@H](O)[C@H](Cc4ccccc4)NC(=O)[C@@H](NC(=O)N(C)Cc5nc6ccccc6[nH]5)C(C)C | Protease |
| CHEMBL323809 | CC(C)[C@@H](NC(=O)N(C)Cc1cccnc1)C(=O)N[C@@H](Cc2ccccc2)[C@@H](O)[C@@H](O)[C@H](Cc3ccccc3)NC(=O)[C@@H](NC(=O)N(C)Cc4cccnc4)C(C)C | Protease |
| CHEMBL322805 | CC(C)[C@@H](NC(=O)N(C)Cc1nccs1)C(=O)N[C@@H](Cc2ccccc2)[C@@H](O)[C@@H](O)[C@H](Cc3ccccc3)NC(=O)[C@@H](NC(=O)N(C)Cc4nccs4)C(C)C | Protease |
| CHEMBL112138 | CC(C)[C@@H](NC(=O)N(C)Cc1ccccn1)C(=O)N[C@@H](Cc2ccccc2)[C@@H](O)[C@H](O)[C@H](Cc3ccccc3)NC(=O)[C@@H](NC(=O)N(C)Cc4ccccn4)C(C)C | Protease |
| CHEMBL329239 | CC(C)[C@H](NC(=O)Cc1csc(N)n1)C(=O)N[C@H](C[C@H](O)[C@H](Cc2ccccc2)NC(=O)[C@@H](NC(=O)Cc3csc(N)n3)C(C)C)Cc4ccccc4 | Protease |
| CHEMBL324260 | CC(C)(C)NC(=O)[C@@H]1CN(CCOCc2ccccc2)CCN1C[C@@H](O)C[C@@H](Cc3ccccc3)C(=O)NC4[C@H](O)Cc5ccccc45 | Protease |
| CHEMBL37383 | CC(C)(C)NC(=O)[C@@H]1CN(Cc2ccc(Cl)c(Cl)c2)CCN1C[C@@H](O)C[C@@H](Cc3ccccc3)C(=O)N[C@@H]4[C@H](O)Cc5ccccc45 | Protease |
| CHEMBL163045 | CC(C)(C)NC(=O)c1ccccc1C[C@@H](O)[C@H](CSc2ccccc2)NC(=O)[C@H](CC(=O)N)NC(=O)c3ccc4ccccc4n3 | Protease |
| CHEMBL277579 | CC(C)[C@H](NC(=O)[C@H](Cc1cccc2ccccc12)CS(=O)(=O)C(C)(C)C)C(=O)NCP(=O)(O)CNC(=O)[C@@H](NC(=O)[C@H](Cc3cccc4ccccc34)CS(=O)(=O)C(C)(C)C)C(C)C | Protease |
| CHEMBL337283 | CC(C)(C)NC(=O)[C@@H]1CCCN1C(=O)[C@@H](O)[C@H](Cc2ccccc2)NC(=O)[C@H](CC(=O)N)NC(=O)c3ccc4ccccc4n3 | Protease |
| CHEMBL72399 | CCCc1cc(CN2CCN(C[C@@H](O)[C@H](Cc3ccccc3)NC(=O)O[C@@H]4CCS(=O)(=O)[C@@H]4C(C)C)[C@@H](C2)C(=O)NC(C)(C)C)ccn1 | Protease |
| CHEMBL194856 | CCC(C)[C@@H](NC(=O)[C@@H](NC(=O)CCCCCCCCCCCCCCC(=O)N[C@H](CC(=O)N[C@@H](Cc1ccccc1)C(=O)O)C(=O)N)C(C)O)C(=O)N[C@@H](Cc2ccccc2)C(=O)N | Protease |
| CHEMBL372040 | CC(C)C1(CCc2ccccc2)CC(=O)C(=C(O)O1)Sc3cc(C)ccc3C(C)(C)C | Protease |
| CHEMBL352273 | CC(=O)N[C@@H](CC(=O)N)C(=O)N[C@@H](CSc1ccc2ccccc2c1)[C@H](O)Cc3ccccc3C(=O)NC(C)(C)C | Protease |
| CHEMBL95419 | CC(C)[C@H](NC(=O)[C@@H](C[C@H](O)[C@H](Cc1ccccc1)NC(=O)OC(C)(C)C)Cc2ccccc2)C(=O)N | Protease |
| CHEMBL323882 | COc1ncccc1CN2CCN(C[C@@H](O)C[C@@H](Cc3ccccc3)C(=O)NC4[C@H](O)Cc5ccccc45)[C@@H](C2)C(=O)NC(C)(C)C | Protease |
| CHEMBL347802 | CC(C)c1nc(CN(C)C(=O)NCCC(=O)N[C@H](C[C@H](O)[C@H](Cc2ccccc2)NC(=O)OCc3cncs3)Cc4ccccc4)cs1 | Protease |
| CHEMBL98228 | COC(=O)[C@@H](NC(=O)[C@@H](C[C@H](O[Si](C)(C)C(C)(C)C)[C@H](Cc1ccccc1)NC(=O)OC(C)(C)C)Cc2ccccc2)C(C)C | Protease |
| CHEMBL295777 | CC(C)(C)OC(=O)N[C@@H](Cc1ccccc1)[C@@H](O)C[C@@H](Cc2ccc(CN3CCOCC3)cc2)C(=O)N[C@@H]4[C@H](O)Cc5ccccc45 | Protease |
| CHEMBL311979 | CC(C)(C)NC(=O)[C@@H]1CN(CCN1C[C@@H](O)C[C@@H](Cc2ccccc2)C(=O)N[C@@H]3[C@H](O)Cc4ccccc34)C(C)(C)c5cccnc5 | Protease |
| CHEMBL310459 | CC(C)(C)CNC(=O)[C@@H]1CN(CCN1C[C@@H](O)C[C@@H](Cc2ccccc2)C(=O)N[C@@H]3[C@H](O)COc4ccccc34)C(C)(C)c5oc6ccncc6c5 | Protease |
| CHEMBL359236 | CC(C)(C)NC(=O)[C@@H]1CN(Cc2noc3ccccc23)CCN1C[C@@H](O)C[C@@H](Cc4ccccc4)C(=O)NC5[C@H](O)Cc6ccccc56 | Protease |
| CHEMBL422786 | Cc1c(O)cc(F)cc1C(=O)N[C@@H](Cc2ccc3ccccc3c2)[C@H](O)C(=O)N4C[C@@H](Cl)C[C@H]4C(=O)NC(C)(C)C | Protease |
| CHEMBL371085 | COc1cc(CN2[C@H](Cc3ccccc3)[C@H](O)CN(N(Cc4ccc(O)c(OC)c4)C2=O)C(=O)CCc5ccncc5)ccc1O | Protease |
| CHEMBL431455 | CC(=O)N[C@H](CS(=O)(=O)c1ccc(F)cc1)C(=O)N[C@@H](Cc2ccccc2)[C@H](O)CN3C[C@H]4CCS[C@H]4C[C@H]3C(=O)NC(C)(C)C | Protease |
| CHEMBL75118 | CC(C)C1(CCc2ccccn2)CC(=O)C(=C(O)O1)Sc3cc(C)c(CO)cc3C(C)(C)C | Protease |
| CHEMBL2374438 | COC(=O)N[C@H](Cc1cccc2ccccc12)C(=O)NCCCC[C@@H](CO)N(CC(C)C)S(=O)(=O)c3ccc(N)cc3 | Protease |
| CHEMBL195073 | CCC(C)[C@@H](NC(=O)[C@H](C(C)O)N(C)C(=O)CCCCCCCCCCCCCCC(=O)NC(Cc1ccc(N)cc1)C(=O)N[C@@H](Cc2ccccc2)C(=O)O)C(=O)NC(CO)C(=O)N | Protease |
| CHEMBL114674 | Cc1cc(O)c(C)c(c1)C(=O)N[C@@H](Cc2cc(F)cc(F)c2)[C@H](O)C(=O)N3C[C@@H](Cl)C[C@H]3C(=O)NC(C)(C)C | Protease |
| CHEMBL359237 | CC(C)(C)NC(=O)[C@@H]1CN(Cc2nc3ccccc3s2)CCN1C[C@@H](O)C[C@@H](Cc4ccccc4)C(=O)NC5[C@H](O)Cc6ccccc56 | Protease |
| CHEMBL406991 | CC(C)(C)NC(=O)c1ccccc1C[C@@H](O)[C@H](Cc2ccccc2)NC(=O)[C@H](CSc3cccc4ccccc34)NS(=O)(=O)C | Protease |
| CHEMBL71153 | CNS(=O)(=O)Oc1cc(c(SC2=C(O)OC(CCc3ccc(O)cc3)(CC2=O)C(C)C)cc1C)C(C)(C)C | Protease |
| CHEMBL147227 | CC(C)(C)NC(=O)[C@@H]1CN(Cc2oc3ccccc3n2)CCN1C[C@@H](O)C[C@@H](Cc4ccccc4)C(=O)NC5[C@H](O)Cc6ccccc56 | Protease |
| CHEMBL307966 | CCC(C)c1cc(CN2CCN(C[C@@H](O)[C@H](Cc3ccccc3)NC(=O)O[C@@H]4CCS(=O)(=O)[C@@H]4C(C)C)[C@@H](C2)C(=O)NC(C)(C)C)ccn1 | Protease |
| CHEMBL154831 | CCCN(Cc1csc(n1)C(C)C)C(=O)NCC(=O)N[C@H](C[C@H](O)[C@H](Cc2ccccc2)NC(=O)OCc3cncs3)Cc4ccccc4 | Protease |
| CHEMBL2374439 | COC(=O)N[C@H](Cc1ccc2ccccc2c1)C(=O)NCCCC[C@@H](CO)N(CC(C)C)S(=O)(=O)c3ccc(N)cc3 | Protease |
| CHEMBL295345 | CC(C)C[C@H](NC(=O)[C@@H](C[C@H](O)[C@H](Cc1ccccc1)NC(=O)OC(C)(C)C)Cc2cccc3ccccc23)C(=O)N[C@@H](Cc4ccccc4)C(=O)N | Protease |
| CHEMBL320126 | CC(C)[C@@H](NC(=O)c1cc2ccccc2[nH]1)C(=O)N[C@@H](Cc3ccccc3)C(O)[C@H](Cc4ccccc4)NC(=O)[C@@H](NC(=O)c5cc6ccccc6[nH]5)C(C)C | Protease |
| CHEMBL98885 | O[C@@H](C[C@@H](Cc1ccccc1)C(=O)N[C@@H]2[C@H](O)Cc3ccccc23)[C@H](Cc4ccccc4)NC(=O)OC5CCOCC5 | Protease |
| CHEMBL136336 | CC(C)(C)OC(=O)N[C@@H](Cc1ccccc1)[C@@H](O)\C=C(\Cc2ccccc2)/C(=O)NC3[C@@H](O)Cc4ccccc34 | Protease |
| CHEMBL322037 | CC(C)(C)OC(=O)N[C@@H](Cc1ccccc1)[C@@H](O)\C=C(\Cc2ccccc2)/C(=O)N[C@@H]3[C@H](O)Cc4ccccc34 | Protease |
| CHEMBL334985 | CC(C)(C)NC(=O)[C@@H]1CCCN1C(=O)[C@@H](O)[C@H](Cc2ccccc2)NC(=O)[C@H](CC(=O)N)NC(=O)COc3ccc4ccccc4c3 | Protease |
| CHEMBL316718 | CC(C)[C@@H]1[C@@H](CCS1(=O)=O)OC(=O)N[C@@H](Cc2ccccc2)[C@H](O)CN3C[C@H]4CCS[C@H]4C[C@H]3C(=O)NC(C)(C)C | Protease |
| CHEMBL113665 | Cc1cc(O)c(C)c(c1)C(=O)N[C@@H](Cc2ccccc2)[C@H](O)C(=O)N3C[C@@H](Cl)C[C@H]3C(=O)NC(C)(C)C | Protease |
| CHEMBL77498 | CC(C)C1(CCc2cscn2)CC(=O)C(=C(O)O1)Sc3cc(C)c(CO)cc3C(C)(C)C | Protease |
| CHEMBL90751 | O[C@@H](C[C@H](Cc1ccccc1)NC(=O)c2ccc(O)cc2Cl)[C@H](Cc3ccccc3)NC(=O)OCc4ccccc4 | Protease |
| CHEMBL304805 | CC(C)C[C@]1(N=CC(=C1O)[C@@H](Cc2ccccc2)C(=O)N)C3=C(O)[C@@](C[C@H](O)[C@H](Cc4ccccc4)NC(=O)O[C@@H]5CCOC5)(Cc6ccccc6)N=C3 | Protease |
| CHEMBL346120 | CC(C)c1nc(CN(C)C(=O)NCCC(=O)N[C@@H](Cc2ccccc2)[C@@H](O)C[C@H](Cc3ccccc3)NC(=O)OCc4cncs4)cs1 | Protease |
| CHEMBL94991 | CCC(C)[C@H](NC(=O)[C@@H](C[C@H](O)[C@H](Cc1ccccc1)NC(=O)OC(C)(C)C)Cc2ccccc2)C(=O)NCc3ccccn3 | Protease |
| CHEMBL53107 | CC(C)C[C@]1(N=CC(=C1O)[C@@H](Cc2ccccc2)C(=O)N)C3=C(O)[C@@](C[C@H](O)[C@@H](CC(=O)OC4CCOC4)Cc5ccccc5)(Cc6ccccc6)N=C3 | Protease |
| CHEMBL78209 | CC(C)C1(CCc2ncsc2C)CC(=O)C(=C(O)O1)Sc3cc(C)c(CO)cc3C(C)(C)C | Protease |
| CHEMBL331585 | Cc1cccc(C[C@H](NC(=O)c2cc(C)cc(O)c2C)[C@H](O)C(=O)N3C[C@@H](Cl)C[C@H]3C(=O)NC(C)(C)C)c1 | Protease |
| CHEMBL168525 | CC(C)C1(CCc2ccc(N)cc2)CC(=O)C(=C(O)O1)Sc3cc(C)c(OS(=O)(=O)c4ccc(cc4)C#N)cc3C(C)(C)C | Protease |
| CHEMBL432436 | Cc1cc(O)c(C)c(c1)C(=O)N[C@@H](Cc2ccc(cc2)c3ccccc3)[C@H](O)C(=O)N4C[C@@H](Cl)C[C@H]4C(=O)NC(C)(C)C | Protease |
| CHEMBL72487 | CC(C)[C@@H]1[C@@H](CCS1(=O)=O)OC(=O)N[C@@H](Cc2ccccc2)[C@H](O)CN3CCN(Cc4ccncc4)C[C@H]3C(=O)NC(C)(C)C | Protease |
| CHEMBL437449 | O[C@@H](C[C@@H](Cc1cccnc1)C(=O)N[C@@H]2[C@H](O)COc3ccccc23)CN4CCN(Cc5ccn(c5)c6ccccc6C(F)(F)F)C[C@H]4C(=O)NCC(F)(F)F | Protease |
| CHEMBL282041 | CC(C)CC(NC(=O)[C@@H](C[C@H](O)[C@H](Cc1ccccc1)NC(=O)OC(C)(C)C)Cc2ccccc2)C(=O)NCc3ccccc3 | Protease |
| CHEMBL318129 | C[C@H]1[C@@H](CCS1(=O)=O)OC(=O)N[C@@H](CSc2ccccc2)[C@H](O)CN3C[C@H]4CCS[C@H]4C[C@H]3C(=O)NC(C)(C)C | Protease |
| CHEMBL320322 | CC(C)[C@@H](NC(=O)N1CCN(CC1)c2ccccc2)C(=O)N[C@@H](Cc3ccccc3)[C@H](O)[C@H](O)[C@H](Cc4ccccc4)NC(=O)[C@@H](NC(=O)N5CCN(CC5)c6ccccc6)C(C)C | Protease |
| CHEMBL327599 | CC(C)(C)NC(=O)[C@@H]1CCCC[C@H]1C[C@@H](O)[C@H](Cc2ccccc2)NC(=O)[C@H](CC(=O)N)NC(=O)c3ccc4ccccc4n3 | Protease |
| CHEMBL46061 | CN(C)Cc1ccc(C[C@H](C[C@H](O)[C@H](Cc2ccccc2)NC(=O)OC(C)(C)C)C(=O)N[C@@H]3[C@H](O)Cc4ccccc34)cc1 | Protease |
| CHEMBL308786 | CC(C)C1(CCc2nc3ccccc3s2)CC(=O)C(=C(O)O1)Sc4cc(C)c(CO)cc4C(C)(C)C | Protease |
| CHEMBL333906 | Cc1cc(SC2=C(O)OC(CCc3ccccc3)(CC2=O)c4ccc(OCCO)cc4)c(cc1CO)C(C)(C)C | Protease |
| CHEMBL307091 | CC(C)[C@@H]1[C@@H](CCS1(=O)=O)OC(=O)N[C@@H](Cc2ccccc2)[C@H](O)CN3CCN(Cc4ccnc(C)c4)C[C@H]3C(=O)NC(C)(C)C | Protease |
| CHEMBL93589 | CC(C)(C)OC(=O)N[C@H](C[C@H](O)[C@H](Cc1ccccc1)NC(=O)c2ccc(O)cc2Cl)Cc3ccccc3 | Protease |
| CHEMBL97824 | CC(C)C[C@H](NC(=O)[C@@H](C[C@H](O)[C@H](Cc1ccccc1)NC(=O)OC(C)(C)C)Cc2ccccc2)C(=O)NCc3ccccc3 | Protease |
| CHEMBL306534 | CC(C)C1(CCc2ccncc2)CC(=O)C(=C(O)O1)Sc3cc(C)c(CO)cc3C(C)(C)C | Protease |
| CHEMBL208907 | CC(C)CN([C@H](CO)CCCCNC(=O)[C@@H](NC(=O)C)C(c1ccccc1)c2ccccc2)S(=O)(=O)c3ccc(N)cc3 | Protease |
| CHEMBL164158 | CC(C)(C)NC(=O)c1ccccc1C[C@@H](O)[C@H](CSc2ccc3ccccc3c2)NC(=O)[C@H](CC(=O)N)NC(=O)OCc4ccccc4 | Protease |
| CHEMBL97299 | CC(C)C[C@H](NC(=O)[C@@H](C[C@H](O)[C@H](Cc1ccccc1)NC(=O)OC(C)(C)C)Cc2ccccc2)C(=O)NC(CO)Cc3ccccc3 | Protease |
| CHEMBL275969 | Cc1cc(S[C@@H]2CCN(C[C@@H](O)[C@H](Cc3ccccc3)NC(=O)COc4c(C)cccc4C)[C@@H](C2)C(=O)NC(C)(C)C)nc(C)n1 | Protease |
| CHEMBL351145 | CC(C)C(NC(=O)N(C)Cc1csc(n1)C(C)C)C(=O)N[C@@H](Cc2ccccc2)[C@@H](O)C[C@H](Cc3ccccc3)NC(=O)OCc4cnc(s4)C(C)C | Protease |
| CHEMBL351726 | CC(C)CCN(C[C@@H](O)[C@H](Cc1ccccc1)NC(=O)[C@H](CC(=O)N)NC(=O)c2ccc3ccccc3n2)S(=O)(=O)c4ccccc4 | Protease |
| CHEMBL168270 | Cc1cc(SC2=C(O)OC(C)(CCc3ccc(O)cc3)CC2=O)c(cc1NS(=O)(=O)c4ccccc4)C(C)(C)C | Protease |
| CHEMBL114216 | CC(C)(C)NC(=O)[C@@H]1C[C@H](Cl)CN1C(=O)[C@@H](O)[C@H](Cc2ccccc2)NC(=O)c3cccc(O)c3Br | Protease |
| CHEMBL324916 | CC(C)[C@@H](NC(=O)CCc1cccnc1)C(=O)N[C@@H](Cc2ccccc2)[C@H](O)[C@H](O)[C@H](Cc3ccccc3)NC(=O)[C@@H](NC(=O)CCc4cccnc4)C(C)C | Protease |
| CHEMBL433309 | CC(C)[C@@H]1NC(=O)CCCCCOc2ccc(C[C@H](NC1=O)[C@H](O)CN3CC4CCCCC4C[C@H]3C(=O)NC(C)(C)C)cc2 | Protease |
| CHEMBL433704 | CC(C)(C)NC(=O)[C@@H]1C[C@@H]2CCCC[C@@H]2CN1C[C@@H](O)[C@H](CSc3ccccc3)NC(=O)[C@H](CC(=O)N)NC(=O)c4ccc5ccccc5n4 | Protease |
| CHEMBL308410 | CCc1cc(CN2CCN(C[C@@H](O)[C@H](Cc3ccccc3)NC(=O)O[C@@H]4CCS(=O)(=O)[C@@H]4C(C)C)[C@@H](C2)C(=O)NC(C)(C)C)ccn1 | Protease |
| CHEMBL155636 | CC(C)CN(Cc1csc(n1)C(C)C)C(=O)NCC(=O)N[C@H](C[C@H](O)[C@H](Cc2ccccc2)NC(=O)OCc3cncs3)Cc4ccccc4 | Protease |
| CHEMBL324747 | CC(C)[C@@H](NC(=O)c1ccc2ccccc2n1)C(=O)N[C@@H](Cc3ccccc3)C(O)[C@H](Cc4ccccc4)NC(=O)[C@@H](NC(=O)c5ccc6ccccc6n5)C(C)C | Protease |
| CHEMBL93718 | Cc1c(O)cccc1C(=O)N[C@@H](Cc2ccccc2)[C@@H](O)C[C@H](Cc3ccccc3)NC(=O)OC(C)(C)C | Protease |
| CHEMBL432101 | CC(C)(C)NC(=O)[C@@H]1CN(CCN1C[C@@H](O)C[C@@H](Cc2ccccc2)C(=O)N[C@@H]3[C@H](O)Cc4ccccc34)S(=O)(=O)C | Protease |
| CHEMBL331363 | CC(C)[C@@H](NC(=O)N(C)Cc1cccnc1)C(=O)N[C@@H](Cc2ccccc2)[C@H](O)[C@H](O)[C@H](Cc3ccccc3)NC(=O)[C@@H](NC(=O)N(C)Cc4cccnc4)C(C)C | Protease |
| CHEMBL57238 | CC(C)[C@@H]1[C@@H](CCS1(=O)=O)OC(=O)N[C@@H](Cc2ccccc2)[C@H](O)CN3CCN(CCc4ccsc4)C[C@H]3C(=O)NC(C)(C)C | Protease |
| CHEMBL432784 | CC(=O)N[C@H](CS(=O)(=O)c1ccc2ccccc2c1)C(=O)N[C@@H](Cc3ccccc3)[C@H](O)CN4C[C@H]5CCCC[C@H]5C[C@H]4C(=O)NC(C)(C)C | Protease |
| CHEMBL81974 | CC(C)(N1CCN(C[C@@H](O)C[C@@H](Cc2ccccc2)C(=O)N[C@@H]3[C@H](O)COc4ccccc34)[C@@H](C1)C(=O)NCCC(F)(F)F)c5oc6ccncc6c5 | Protease |
| CHEMBL74787 | CC(C)c1nc(CCC2(CC(=O)C(=C(O)O2)Sc3cc(C)c(CO)cc3C(C)(C)C)C(C)C)cs1 | Protease |
| CHEMBL112930 | CC(C)[C@@H](NS(=O)(=O)CCc1ccccn1)C(=O)N[C@@H](Cc2ccccc2)[C@@H](O)[C@@H](O)[C@H](Cc3ccccc3)NC(=O)[C@@H](NS(=O)(=O)CCc4ccccn4)C(C)C | Protease |
| CHEMBL70386 | CN(C)S(=O)(=O)Oc1cc(c(SC2=C(O)OC(C)(CCc3ccc(O)cc3)CC2=O)cc1C)C(C)(C)C | Protease |
| CHEMBL267571 | Cc1cccc(C)c1OCC(=O)N[C@@H](Cc2ccccc2)[C@H](O)CN3CC[C@H](C[C@H]3C(=O)NC(C)(C)C)Sc4ccncc4 | Protease |
| CHEMBL291203 | CC(C)(C)OC(=O)N[C@@H](Cc1ccccc1)[C@@H](O)C[C@@H](Cc2ccc(OCCN3CCCCC3)cc2)C(=O)NC4[C@H](O)Cc5ccccc45 | Protease |
| CHEMBL113575 | CC(C)[C@@H](NC(=O)N(C)Cc1ccccn1)C(=O)N[C@@H](Cc2ccccc2)[C@H](O)[C@H](O)[C@H](Cc3ccccc3)NC(=O)[C@@H](NC(=O)N(C)Cc4ccccn4)C(C)C | Protease |
| CHEMBL313876 | CC(C)(C)NC(=O)[C@@H]1CN(Cc2cccnc2)CCN1C[C@@H](O)C[C@@H](Cc3ccncc3)C(=O)N[C@@H]4[C@H](O)Cc5ccccc45 | Protease |
| CHEMBL58043 | Cc1ccc(C[C@@H](O)C[C@@H](Cc2ccccc2)C(=O)N[C@@H]3[C@H](O)Cc4ccccc34)c(c1)C(=O)NC(C)(C)C | Protease |
| CHEMBL325395 | CC(C)(C)NC(=O)C1CN(Cc2c[nH]c3ccccc23)CCN1CC(O)CC(Cc4ccccc4)C(=O)NC5C(O)Cc6ccccc56 | Protease |
| CHEMBL415318 | CC(=O)N[C@H](CS(=O)(=O)c1cccc2cccnc12)C(=O)N[C@@H](Cc3ccccc3)[C@H](O)CN4C[C@H]5CCCC[C@H]5C[C@H]4C(=O)NC(C)(C)C | Protease |
| CHEMBL121545 | Cc1cc(SC2=C(O)OC(CCc3ccc(O)cc3)(CC2=O)c4ccccc4)c(cc1CO)C(C)(C)C | Protease |
| CHEMBL317926 | CC(C)(C)NC(=O)[C@@H]1CN(Cc2cccnc2)CCN1C[C@@H](O)C[C@@H](Cc3cccnc3)C(=O)N[C@@H]4[C@H](O)Cc5ccccc45 | Protease |
| CHEMBL172271 | CCNS(=O)(=O)Nc1cc(c(SC2=C(O)OC(CCc3ccc(O)cc3)(CC2=O)C(C)C)cc1C)C(C)(C)C | Protease |
| CHEMBL100048 | CC(C)(C)NC(=O)[C@@H]1C[C@@H]2SCC[C@@H]2CN1C[C@@H](O)[C@H](CSc3ccccc3)NC(=O)O[C@H]4CCS(=O)(=O)C4 | Protease |
| CHEMBL322804 | CC(C)[C@@H](NC(=O)OCc1ccncc1)C(=O)N[C@@H](Cc2ccccc2)[C@H](O)[C@H](O)[C@H](Cc3ccccc3)NC(=O)[C@@H](NC(=O)OCc4ccncc4)C(C)C | Protease |
| CHEMBL332498 | CC(C)(C)NC(=O)C1CN(Cc2ccc3occc3c2)CCN1CC(O)CC(Cc4ccccc4)C(=O)NC5C(O)Cc6ccccc56 | Protease |
| CHEMBL325394 | Cc1c(O)cc(F)cc1C(=O)N[C@@H](Cc2ccccc2)[C@H](O)C(=O)N3C[C@@H](Cl)C[C@H]3C(=O)NC(C)(C)C | Protease |
| CHEMBL13011 | Cc1cccc(C)c1OCC(=O)N[C@@H](Cc2ccccc2)[C@H](O)CN3CC[C@H](C[C@H]3C(=O)NC(C)(C)C)SCc4cccnc4 | Protease |
| CHEMBL116845 | CC(C)(C)NC(=O)[C@@H]1CN(CCN1C[C@@H](O)C[C@@H](Cc2ccccc2)C(=O)NC3[C@H](O)Cc4ccccc34)C(=O)c5cnc6occc6c5 | Protease |
| CHEMBL95516 | CNCCNC(=O)[C@@H](NC(=O)[C@@H](C[C@H](O)[C@H](Cc1ccccc1)NC(=O)OC(C)(C)C)Cc2ccccc2)C(C)C | Protease |
| CHEMBL317160 | CC(C)(C)NC(=O)[C@@H]1C[C@@H]2SCC[C@@H]2CN1C[C@@H](O)[C@H](CSc3ccccc3)NC(=O)[C@@H](CS(=O)(=O)c4ccc5ccccc5c4)NC(=O)C(F)(F)F | Protease |
| CHEMBL13217 | Cc1cccc(C)c1OCC(=O)N[C@@H](Cc2ccccc2)[C@H](O)CN3CC[C@H](C[C@H]3C(=O)NC(C)(C)C)SCc4ccncc4 | Protease |
| CHEMBL72385 | CCc1cc(CN2CCN(C[C@@H](O)[C@H](Cc3ccccc3)NC(=O)O[C@@H]4CCS(=O)(=O)[C@@H]4C(C)C)[C@@H](C2)C(=O)NC(C)(C)C)cc(CC)n1 | Protease |
| CHEMBL328205 | CC(=O)N[C@H](CS(=O)(=O)c1ccc(F)cc1)C(=O)N[C@@H](CSc2ccccc2)[C@H](O)CN3C[C@H]4CCS[C@H]4C[C@H]3C(=O)NC(C)(C)C | Protease |
| CHEMBL295609 | CC(C)(C)OC(=O)N[C@@H](Cc1ccccc1)[C@@H](O)C[C@@H](Cc2ccc(OCCN3CCCC3)cc2)C(=O)NC4[C@H](O)Cc5ccccc45 | Protease |
| CHEMBL20546 | CC(C)(C)OC(=O)N[C@@H](Cc1ccccc1)[C@@H](O)C[C@@H](Cc2ccccc2)C(=O)NC3[C@@H](Cc4ccccc34)C(=O)N | Protease |
| CHEMBL11050 | CC(C)(C)NC(=O)c1ccccc1C[C@@H](O)[C@H](Cc2ccccc2)NC(=O)[C@H](CSc3ccc4ccccc4c3)NS(=O)(=O)C | Protease |
| CHEMBL71440 | Cc1cc(SC2=C(O)OC(C)(CCc3ccc(O)cc3)CC2=O)c(cc1OS(=O)(=O)N)C(C)(C)C | Protease |
| CHEMBL357039 | O[C@@H](C[C@]1(Cc2ccccc2)CCN([C@@H]3CS(=O)(=O)Cc4ccccc34)C1=O)[C@H](Cc5ccccc5)NC(=O)O[C@H]6CCOC6 | Protease |
| CHEMBL287361 | CC(C)C1(CCc2ccc(O)cc2)CC(=O)C(=C(O)O1)Sc3cc(C)c(NS(=O)(=O)c4ccccc4)cc3C(C)(C)C | Protease |
| CHEMBL280358 | CC(C)C[C@H](COCc1ccccc1)NC(=O)C(F)(F)C(=O)C(Cc2ccc(OCc3ccccc3)cc2)NC(=O)[C@@H](NC(=O)OCc4ccccc4)C(C)C | Protease |
| CHEMBL315403 | CC(C)(C)NC(=O)[C@@H]1C[C@@H]2CCCC[C@@H]2CN1C[C@@H](O)[C@H](Cc3ccccc3)NC(=O)O[C@H]4CO[C@H]5OCC[C@@H]45 | Protease |
| CHEMBL350861 | CC(C)c1ccccc1SC2=C(O)OC(CCCCC(=O)O)(CC2=O)c3ccccc3 | Protease |
| CHEMBL1790964 | CC[C@@H](C)[C@H](NC(=O)[C@H](C(C)C)C(O)C(O)[C@H](CC1CCCCC1)NC(=O)C(Cc2c[nH]cn2)NC(=O)COc3cccc4ccccc34)C(=O)NCc5ccccn5 | Protease |
| CHEMBL295013 | CC(C)(C)OC(=O)N[C@@H](Cc1ccc(OCCN2CCOCC2)cc1)[C@@H](O)C[C@@H](Cc3ccc(OCCN4CCOCC4)cc3)C(=O)NC5[C@H](O)Cc6ccccc56 | Protease |
| CHEMBL21041 | CC(C)(C)OC(=O)N[C@@H](Cc1ccccc1)[C@@H](O)C[C@@H](Cc2ccccc2)C(=O)NC3[C@H](O)[C@H](N)c4ccccc34 | Protease |
| CHEMBL117088 | Cc1cc(O)c(C)c(c1)C(=O)N[C@@H](Cc2cccc(c2)C(F)(F)F)[C@H](O)C(=O)N3C[C@@H](Cl)C[C@H]3C(=O)NC(C)(C)C | Protease |
| CHEMBL169878 | CC(C)C1(CCc2ccccc2)CC(=O)C(=C(O)O1)Sc3cc(C)c(NS(=O)(=O)Cn4ccnc4)cc3C(C)(C)C | Protease |
| CHEMBL335391 | CC(C)[C@H](NC(=O)COc1cccc2ccccc12)C(=O)N[C@@H](Cc3ccccc3)[C@H](O)C(=O)N4CCC[C@H]4C(=O)NC(C)(C)C | Protease |
| CHEMBL71334 | CCC(CC)NC(=O)C[C@@H](C(=O)NC[C@@H](O)[C@H](Cc1ccccc1)NC(=O)[C@H](CC(=O)N)NC(=O)c2cnc3ccccc3n2)C(C)(C)C | Protease |
| CHEMBL171434 | Cc1cc(SC2=C(O)OC(C)(CCc3ccc(O)cc3)CC2=O)c(cc1NS(=O)(=O)Cn4ccnc4)C(C)(C)C | Protease |
| CHEMBL346943 | CC(C)C(NC(=O)OCc1nc(cs1)C(C)C)C(=O)N[C@@H](Cc2ccccc2)[C@@H](O)C[C@H](Cc3ccccc3)NC(=O)OCc4cncs4 | Protease |
| CHEMBL11137 | CC(C)(C)NC(=O)c1ccccc1C[C@@H](O)[C@H](Cc2ccccc2)NC(=O)[C@H](C[S+]([O-])c3ccccc3)NS(=O)(=O)C | Protease |
| CHEMBL283522 | CC(C)[C@H](NC(=O)COc1cccc2ccccc12)C(=O)N[C@@H](Cc3ccccc3)C(O)C(=O)N4CCC[C@@H]4C(=O)NC(C)(C)C | Protease |
| CHEMBL348147 | CC(C)C(NC(=O)N(Cc1csc(n1)C(C)C)C2CC2)C(=O)N[C@@H](Cc3ccccc3)[C@@H](O)C[C@H](Cc4ccccc4)NC(=O)OCc5cncs5 | Protease |
| CHEMBL293808 | O[C@@H](C[C@]1(Cc2ccccc2)N=CC(=C1O)C3[C@H](O)Cc4ccccc34)[C@H](Cc5ccccc5)NC(=O)O[C@H]6CCOC6 | Protease |
| CHEMBL382463 | NC(=O)O[C@@H]1Cc2ccccc2[C@@H]1C3=C(O)[C@@](C[C@H](O)[C@H](Cc4ccccc4)NC(=O)O[C@H]5CCOC5)(Cc6ccccc6)N=C3 | Protease |
| CHEMBL202110 | O[C@@H](C[C@]1(Cc2ccccc2)N=CC(=C1O)[C@@H]3[C@H](O)Cc4ccccc34)[C@H](Cc5ccccc5)NC(=O)O[C@H]6CCOC6 | Protease |
| CHEMBL45474 | CC(C)(C)OC(=O)N[C@@H](Cc1ccccc1)[C@@H](O)C[C@@H](Cc2ccc(OCCCN3CCOCC3)cc2)C(=O)NC4[C@H](O)Cc5ccccc45 | Protease |
| CHEMBL55070 | NC(=O)O[C@@H]1Cc2ccccc2C1C3=C(O)[C@@](C[C@H](O)[C@H](Cc4ccccc4)NC(=O)O[C@H]5CCOC5)(Cc6ccccc6)N=C3 | Protease |
| CHEMBL132777 | CC(C)(C)NC(=O)C1CCCCN1C[C@H](O)[C@H](Cc2ccccc2)NC(=O)C(CC(=O)N)NC(=O)c3ccc4ccccc4n3 | Protease |
| CHEMBL12790 | CC(C)(C)NC(=O)[C@@H]1CCCCN1C[C@@H](O)[C@H](Cc2ccccc2)NC(=O)[C@H](CC(=O)N)NC(=O)c3ccc4ccccc4n3 | Protease |
| CHEMBL75310 | CC(C)[C@@H]1[C@@H](CCS1(=O)=O)OC(=O)N[C@@H](Cc2ccccc2)[C@H](O)CN3CCN(Cc4ccc(C)nc4)C[C@H]3C(=O)NC(C)(C)C | Protease |
| CHEMBL307029 | CC(C)(C)NC(=O)C1CCCCN1CC(O)[C@H](Cc2ccccc2)NC(=O)[C@H](CC(=O)N)NC(=O)c3ccc4ccccc4n3 | Protease |
| CHEMBL182360 | COc1ccc(cc1)S(=O)(=O)N(C[C@@H](O)CN2[C@@H](Cc3ccccc3)COC(CC=C)C2=O)CC4CCCC4 | Protease |
| CHEMBL274569 | CC(C)(C)NC(=O)c1ccccc1C[C@@H](O)[C@H](Cc2ccccc2)NC(=O)[C@H](CS(=O)(=O)c3ccc(cc3)C(F)(F)F)NS(=O)(=O)C | Protease |
| CHEMBL316608 | CC(C)(C)NC(=O)[C@@H]1C[C@@H]2SCC[C@@H]2CN1C[C@@H](O)[C@H](CSc3ccccc3)NC(=O)O[C@@H]4CCS(=O)(=O)C4 | Protease |
| CHEMBL2114261 | CC(C)(C)NC(=O)[C@@H]1CCCC[C@H]1C[C@@H](O)[C@H](Cc2ccccc2)NC(=O)[C@H](CC(=O)N)NC(=O)OCc3ccccc3 | Protease |
| CHEMBL169256 | Cc1cc(SC2=C(O)OC(C)(CCc3ccc(O)cc3)CC2=O)c(cc1OS(=O)(=O)Cn4ccnc4)C(C)(C)C | Protease |
| CHEMBL318668 | CC(C)(C)NC(=O)[C@@H]1C[C@@H]2CCCC[C@@H]2CN1C[C@@H](O)[C@H](Cc3ccccc3)NC(=O)[C@@H](CS(=O)(=O)c4ccc(F)cc4)NS(=O)(=O)C | Protease |
| CHEMBL314234 | CC(C)[C@H](NC(=O)OCc1ccccc1)C(=O)NC(Cc2ccc(OCc3ccccc3)cc2)C(=O)C(F)(F)C(=O)N4CCc5ccccc5C4 | Protease |
| CHEMBL115332 | Cc1c(O)cc(F)cc1C(=O)N[C@@H](Cc2cccc(c2)C(F)(F)F)[C@H](O)C(=O)N3C[C@@H](Cl)C[C@H]3C(=O)NC(C)(C)C | Protease |
| CHEMBL318285 | CC(C)CN(C[C@@H](O)[C@H](Cc1ccccc1)NC(=O)[C@@H](C[S+]([O-])c2ccc3ccccc3c2)NS(=O)(=O)C)C(=O)NC(C)(C)C | Protease |
| CHEMBL98284 | CC(C)C[C@H](NC(=O)[C@@H](C[C@H](O)[C@H](Cc1ccccc1)NC(=O)OC(C)(C)C)Cc2ccccc2)C(=O)NCCO | Protease |
| CHEMBL168369 | Cc1cc(SC2=C(O)OC(C)(CCc3ccc(O)cc3)CC2=O)c(cc1NS(=O)(=O)c4ccc(cc4)C#N)C(C)(C)C | Protease |
| CHEMBL200296 | COc1cc(CN2[C@H](Cc3ccccc3)[C@H](O)CN(N(Cc4ccc(O)c(OC)c4)C2=O)C(=O)CCCCN5CCOCC5)ccc1O | Protease |
| CHEMBL178421 | CC(C)CN([C@@H](CCCCNC(=O)OCC1c2ccccc2c3ccccc13)C(=O)O)S(=O)(=O)c4ccc(N)cc4 | Protease |
| CHEMBL276833 | Cn1nnnc1[S+]([O-])C[C@H](NS(=O)(=O)C)C(=O)N[C@@H](Cc2ccccc2)[C@H](O)Cc3ccccc3C(=O)NC(C)(C)C | Protease |
| CHEMBL421330 | CC(C)(N1CCN(C[C@@H](O)C[C@@H](Cc2ccccc2)C(=O)N[C@@H]3[C@H](O)COc4ccccc34)[C@@H](C1)C(=O)NCc5ccccc5)c6oc7ccncc7c6 | Protease |
| CHEMBL166757 | CC(C)C1(CCc2ccc(O)cc2)CC(=O)C(=C(O)O1)Sc3cc(C)c(OS(=O)(=O)C)cc3C(C)(C)C | Protease |
| CHEMBL66399 | CCC(CC)NC(=O)C[C@@H](C(=O)NC[C@@H](O)[C@H](Cc1ccccc1)NC(=O)[C@H](CC(=O)N)NC(=O)c2ccc3ccccc3n2)C(C)(C)C | Protease |
| CHEMBL169391 | CC(C)C1(CCc2ccc(O)cc2)CC(=O)C(=C(O)O1)Sc3cc(C)c(NS(=O)(=O)c4ccc(cc4)C#N)cc3C(C)(C)C | Protease |
| CHEMBL29259 | Cc1ccc(C(=O)NC(C)(C)C)c(C[C@@H](O)[C@H](Cc2ccccc2)NC(=O)[C@H](CC(=O)N)NC(=O)c3ccc4ccccc4n3)c1 | Protease |
| CHEMBL66460 | CCC(CC)NC(=O)C[C@@H](C(=O)NC[C@@H](O)[C@H](Cc1ccccc1)NC(=O)[C@@H](NC(=O)c2cnc3ccccc3n2)C(C)C)C(C)(C)C | Protease |
| CHEMBL113626 | CC(C)[C@@H](NC(=O)\C=C\c1cccnc1)C(=O)N[C@@H](Cc2ccccc2)C(O)[C@H](Cc3ccccc3)NC(=O)[C@@H](NC(=O)\C=C\c4cccnc4)C(C)C | Protease |
| CHEMBL171229 | CCCC1(CCc2ccc(O)cc2)CC(=O)C(=C(O)O1)Sc3cc(C)c(OS(=O)(=O)Cn4ccnc4)cc3C(C)(C)C | Protease |
| CHEMBL86226 | CC(C)C1(CCc2ccc(N)cc2)CC(=O)C(=C(O)O1)Sc3cc(C)c(OS(=O)(=O)Cn4ccnc4)cc3C(C)(C)C | Protease |
| CHEMBL351994 | CC(C)CCN(C[C@@H](O)[C@H](Cc1ccccc1)NC(=O)[C@H](CC(=O)N)NC(=O)OCc2ccccn2)S(=O)(=O)c3ccccc3 | Protease |
| CHEMBL356205 | CN1C=C(CN2CCN(C[C@@H](O)C[C@@H](Cc3ccccc3)C(=O)NC4[C@H](O)Cc5ccccc45)[C@@H](C2)C(=O)NC(C)(C)C)C=CC1=O | Protease |
| CHEMBL306772 | CC(C)[C@@H]1[C@@H](CCS1(=O)=O)OC(=O)N[C@@H](Cc2ccccc2)[C@H](O)CN3CCN(Cc4cc(nc(c4)C(C)C)C(C)C)C[C@H]3C(=O)NC(C)(C)C | Protease |
| CHEMBL418528 | CC(C)(C)NC(=O)c1ccccc1C[C@@H](O)[C@H](Cc2ccccc2)NC(=O)[C@H](C[S+]([O-])c3ccc(cc3)C(F)(F)F)NS(=O)(=O)C | Protease |
| CHEMBL71472 | CCNS(=O)(=O)Oc1cc(c(SC2=C(O)OC(C)(CCc3ccc(O)cc3)CC2=O)cc1C)C(C)(C)C | Protease |
| CHEMBL279971 | CCC(C)C(NC(=O)[C@@H]1CCCN1C[C@@H](O)[C@@H](Cc2ccccc2)NC(=O)C(CC(=O)N)NC(=O)c3ccc4ccccc4n3)C(=O)NC(Cc5ccccc5)C(=O)OC | Protease |
| CHEMBL347824 | CCN(CCc1csc(n1)C(C)C)C(=O)NC(C)C(=O)N[C@H](C[C@H](O)[C@H](Cc2ccccc2)NC(=O)OCc3cncs3)Cc4ccccc4 | Protease |
| CHEMBL109221 | CC(C)[C@@H](NC(=O)OCc1ccncc1)C(=O)N[C@@H](Cc2ccccc2)[C@@H](O)[C@@H](O)[C@H](Cc3ccccc3)NC(=O)[C@@H](NC(=O)OCc4ccncc4)C(C)C | Protease |
| CHEMBL1790969 | CC[C@H](C)[C@H](NC(=O)[C@@H]1CCCN1CC(O)[C@H](Cc2ccccc2)NC(=O)[C@H](CC(=O)N)NC(=O)c3ccc4ccccc4n3)C(=O)N[C@@H](Cc5ccccc5)C(=O)OC | Protease |
| CHEMBL104118 | CCNC(=O)[C@@H](NC(=O)CCc1ccccc1)[C@@H]2N[C@@H](C(=O)NCCNC(=O)[C@@H]3N[C@H](SC3(C)C)[C@H](NC(=O)CCc4ccccc4)C(=O)NCC)C(C)(C)S2 | Protease |
| CHEMBL383732 | NC(=O)C[C@H]1CC(C2=C(O)[C@@](C[C@H](O)[C@H](Cc3ccccc3)NC(=O)O[C@H]4CCOC4)(Cc5ccccc5)N=C2)c6ccccc16 | Protease |
| CHEMBL266398 | COc1ccc(cc1)S(=O)(=O)C[C@H](NS(=O)(=O)C)C(=O)N[C@@H](Cc2ccccc2)[C@H](O)Cc3ccccc3C(=O)NC(C)(C)C | Protease |
| CHEMBL170162 | CC(C)C1(CCc2ccc(O)cc2)CC(=O)C(=C(O)O1)Sc3cc(C)c(OS(=O)(=O)Cn4ccnc4)cc3C(C)(C)C | Protease |
| CHEMBL304086 | CCC(CC)NC(=O)C[C@@H](C(=O)NC[C@@H](O)[C@H](Cc1ccccc1)NC(=O)[C@@H](NC(=O)c2ccc3ccccc3n2)C(C)C)C(C)(C)C | Protease |
| CHEMBL123229 | Cc1cc(SC2=C(O)OC(CCc3cccc(O)c3)(CC2=O)c4ccccc4)c(cc1CO)C(C)(C)C | Protease |
| CHEMBL117495 | Cc1cccc(C[C@H](NC(=O)c2ccc(C)c(O)c2C)[C@H](O)C(=O)N3C[C@@H](Cl)C[C@H]3C(=O)NC(C)(C)C)c1 | Protease |
| CHEMBL419072 | CC(C)[C@@H]1[C@@H](CCS1(=O)=O)OC(=O)N[C@@H](Cc2ccccc2)[C@H](O)CN3CCN(Cc4cc(C)nc(C)c4)C[C@H]3C(=O)NC(C)(C)C | Protease |
| CHEMBL116723 | Cc1ccc(O)c(C)c1C(=O)N[C@@H](Cc2ccccc2)[C@H](O)C(=O)N3C[C@@H](Cl)C[C@H]3C(=O)NC(C)(C)C | Protease |
| CHEMBL377274 | CC(C)(C)OC(=O)N[C@@H](Cc1ccccc1)[C@@H](O)C[C@]2(Cc3ccccc3)N=CC(=C2O)C4C5OC(=O)N[C@@H]5c6ccccc46 | Protease |
| CHEMBL302289 | CC(C)C1(CCc2ccc(O)cc2)CC(=O)C(=C(O)O1)Sc3cc(C)c(OS(=O)(=O)N4CCOCC4)cc3C(C)(C)C | Protease |
| CHEMBL169714 | CC(C)C1(CCc2ccc(O)cc2)CC(=O)C(=C(O)O1)Sc3cc(C)c(NS(=O)(=O)c4ccccn4)cc3C(C)(C)C | Protease |
| CHEMBL354867 | CC(C)C1(CCc2ccc(O)cc2)CC(=O)C(=C(O)O1)Sc3cc(C)c(NS(=O)(=O)c4cccnc4)cc3C(C)(C)C | Protease |
| CHEMBL100271 | CC(C)[C@@H]1SCC[C@@H]1OC(=O)N[C@@H](Cc2ccccc2)[C@H](O)CN3C[C@H]4CCS[C@H]4C[C@H]3C(=O)NC(C)(C)C | Protease |
| CHEMBL265588 | CC(C)CN(C[C@@H](O)[C@H](Cc1ccccc1)NC(=O)COc2c(C)cccc2C)S(=O)(=O)c3ccc(N)cc3 | Protease |
| CHEMBL307926 | CC(C)(C)NC(=O)[C@@H]1C[C@@H]2CCCC[C@@H]2CN1C[C@@H](O)[C@H](Cc3ccccc3)NC(=O)[C@@H](NC(=O)c4ccc5ccccc5n4)C6CCCC6 | Protease |
| CHEMBL321778 | CC(C)[C@H]1[C@H](CCS1(=O)=O)OC(=O)N[C@@H](Cc2ccccc2)[C@H](O)CN3C[C@H]4CCS[C@H]4C[C@H]3C(=O)NC(C)(C)C | Protease |
| CHEMBL90090 | Cc1c(N)cccc1C(=O)N[C@@H](Cc2ccccc2)[C@@H](O)C[C@H](Cc3ccccc3)NC(=O)OC(C)(C)C | Protease |
| CHEMBL25123 | CC(C)(C)NC(=O)C1CC2CCCCC2CN1CC(O)C(Cc3ccccc3)NC(=O)C(CC(=O)N)NC(=O)OCc4ccccc4 | Protease |
| CHEMBL38268 | CC(C)C1(CCc2ccc(O)cc2)CC(=O)C(=C(O)O1)Sc3cc(C)c(N)cc3C(C)(C)C | Protease |
| CHEMBL97847 | CCC(C)[C@H](NC(=O)[C@@H](C[C@H](O)[C@H](Cc1ccccc1)NC(=O)OC(C)(C)C)Cc2ccccc2)C(=O)N | Protease |
| CHEMBL323014 | CC(C)(C)NC(=O)C1CC2CCCCC2CN1C[C@@H](O)[C@H](Cc3ccccc3)NC(=O)[C@H](CC(=O)N)NC(=O)OCc4ccccc4 | Protease |
| CHEMBL337053 | CC(C)(C)NC(=O)[C@@H]1C[C@@H]2CCCC[C@@H]2CN1C[C@H](O)[C@H](Cc3ccccc3)NC(=O)[C@H](CC(=O)N)NC(=O)OCc4ccccc4 | Protease |
| CHEMBL275592 | CC(C)(C)NC(=O)c1ccccc1C[C@@H](O)[C@H](Cc2ccccc2)NC(=O)C(CS(=O)(=O)c3ccc4ccccc4n3)NS(=O)(=O)C | Protease |
| CHEMBL324710 | Cc1ccc(O)c(C)c1C(=O)N[C@@H](Cc2ccc3ccccc3c2)[C@H](O)C(=O)N4C[C@@H](Cl)C[C@H]4C(=O)NC(C)(C)C | Protease |
| CHEMBL265026 | CC(C)(C)NC(=O)[C@@H]1C[C@@H]2CCCC[C@@H]2CN1C[C@@H](O)[C@H](Cc3ccccc3)NC(=O)[C@@H](NC(=O)c4cnccn4)[C@H]5CCOC5 | Protease |
| CHEMBL96996 | CC(C)[C@H](NC(=O)[C@@H](C[C@H](O)[C@H](Cc1ccccc1)NC(=O)OC(C)(C)C)Cc2ccccc2)C(=O)NCCCN3CCOCC3 | Protease |
| CHEMBL69062 | CCC(CC)NC(=O)C[C@@H](C(=O)NC[C@@H](O)[C@H](Cc1ccccc1)NC(=O)[C@@H](NC(=O)OCc2ccccc2)C(C)C)C(C)(C)C | Protease |
| CHEMBL1790668 | CC[C@H](C)[C@H](NC(=O)[C@H](C)N(CC=C)C(=O)[C@H](Cc1ccccc1)NC(=O)[C@H](C)NC(=O)[C@H](C)NC(=O)OC(C)(C)C)C(=O)N[C@@H](C(C)C)C(=O)OC | Protease |
| CHEMBL106633 | CCNC(=O)[C@@H](NC(=O)\C=C/c1ccccc1)[C@@H]2N[C@@H](C(=O)NCCNC(=O)[C@@H]3N[C@H](SC3(C)C)[C@H](NC(=O)\C=C/c4ccccc4)C(=O)NCC)C(C)(C)S2 | Protease |
| CHEMBL98002 | CC(C)(C)NC(=O)N(C[C@@H](O)[C@H](Cc1ccccc1)NC(=O)[C@H](CC(=O)N)NC(=O)c2ccc3ccccc3n2)Cc4ccccc4 | Protease |
| CHEMBL430891 | CC(C)[C@@H](NC(=O)OCc1ccccc1)C(=O)N[C@@H](Cc2ccccc2)[C@H](O)[C@H](Cc3ccccc3)NC(=O)[C@@H](NC(=O)OCc4ccccc4)C(C)C | Protease |
| CHEMBL169381 | CC(C)C1(CCc2ccccc2)CC(=O)C(=C(O)O1)Sc3cc(C)c(NS(=O)(=O)c4ccccn4)cc3C(C)(C)C | Protease |
| CHEMBL336684 | COc1ccc(cc1)S(=O)(=O)N(CC(C)C)C[C@@H](O)[C@H](Cc2ccccc2)NC(=O)OCc3ccccc3 | Protease |
| CHEMBL68721 | CC(C)[C@H](NC(=O)OCc1ccccc1)C(=O)N[C@@H](Cc2ccccc2)[C@H](O)CNC(=O)[C@H](CC(=O)NC(C)(C)C)C(C)(C)C | Protease |
| CHEMBL354829 | CC(C)C1(CCc2ccccc2)CC(=O)C(=C(O)O1)Sc3cc(C)c(OS(=O)(=O)Cn4ccnc4)cc3C(C)(C)C | Protease |
| CHEMBL18067 | CC(C)[C@@H]1[C@@H](CCS1(=O)=O)OC(=O)N[C@@H](Cc2ccccc2)[C@H](O)CN3C[C@H]4CCCC[C@H]4C[C@H]3C(=O)NC(C)(C)C | Protease |
| CHEMBL69995 | CC(C)(C)NC(=O)N(CC(O)[C@H](Cc1ccccc1)NC(=O)[C@H](CC(=O)N)NC(=O)c2ccc3ccccc3n2)Cc4ccccc4 | Protease |
| CHEMBL40760 | CC[C@H](C)[C@H](NC(=O)[C@@H](CC(O)C[C@@H](Cc1ccccc1)C(=O)N[C@@H]([C@@H](C)CC)C(=O)NCc2nc3ccccc3[nH]2)Cc4ccccc4)C(=O)NCc5nc6ccccc6[nH]5 | Protease |
| CHEMBL283950 | NC(=O)c1[nH]c2ccc(Cl)cc2c1S(=O)(=O)c3ccccc3 | Protease |
| CHEMBL72474 | CC(C)[C@H](NC(=O)C)C(=O)N[C@@H](Cc1ccccc1)[C@H](O)CN2CC3CCCCC3C[C@H]2C(=O)NC(C)(C)C | Protease |
| CHEMBL124555 | CC(C)(C)c1ccccc1SC2=C(O)OC(CCc3ccccc3)(CC2=O)c4ccccc4 | Protease |
| CHEMBL70444 | CCCNS(=O)(=O)Oc1cc(c(SC2=C(O)OC(CCc3ccc(O)cc3)(CC2=O)C(C)C)cc1C)C(C)(C)C | Protease |
| CHEMBL132432 | CC(C)CCNC(=O)N(C[C@@H](O)[C@H](Cc1ccccc1)NC(=O)[C@H](CC(=O)N)NC(=O)c2ccc3ccccc3n2)C(C)(C)C | Protease |
| CHEMBL315121 | CCCC1(CCc2ccccc2)OC(=C(C(CC)c3cccc(NS(=O)(=O)c4ccc(cn4)C#N)c3)C(=O)O1)O | Protease |
| CHEMBL318894 | C[C@H]1OCC[C@H]1OC(=O)N[C@@H](CSc2ccccc2)[C@H](O)CN3C[C@H]4CCS[C@H]4C[C@H]3C(=O)NC(C)(C)C | Protease |
| CHEMBL318379 | CCNC(=O)[C@@H](NC(=O)Cc1ccccc1)[C@@H]2N[C@@H](C(=O)NCCNC(=O)C3N[C@H](SC3(C)C)[C@H](NC(=O)Cc4ccccc4)C(=O)NCC)C(C)(C)S2 | Protease |
| CHEMBL99616 | CC(C)CCN(C[C@@H](O)[C@H](Cc1ccccc1)NC(=O)[C@H](CC(=O)N)NC(=O)c2ccc3ccccc3n2)C(=O)NC(C)(C)C | Protease |
| CHEMBL105695 | CC1(C)S[C@@H](N[C@H]1C(=O)NCCNC(=O)[C@@H]2N[C@H](SC2(C)C)[C@H](NC(=O)Cc3ccccc3)C(=O)N)[C@H](NC(=O)Cc4ccccc4)C(=O)N | Protease |
| CHEMBL263540 | COc1ccc(cc1)S(=O)(=O)N(CC(C)C)C[C@@H](O)[C@H](Cc2ccccc2)NC(=O)c3cccc(O)c3C | Protease |
| CHEMBL307193 | CC(C)[C@H](NC(=O)OCc1ccccc1)C(=O)N[C@@H](Cc2ccccc2)C(O)[C@H](Cc3ccccc3)NC(=O)[C@@H](NC(=O)OCc4ccccc4)C(C)C | Protease |
| CHEMBL69392 | CC(C)CCN(CC(O)[C@H](Cc1ccccc1)NC(=O)[C@H](CC(=O)N)NC(=O)c2ccc3ccccc3n2)C(=O)NC(C)(C)C | Protease |
| CHEMBL17788 | CC(C)[C@@H]1[C@@H](CCS1(=O)=O)OC(=O)N[C@@H](Cc2ccccc2)[C@H](O)CN3C[C@H]4CCC[C@H]4C[C@H]3C(=O)NC(C)(C)C | Protease |
| CHEMBL1790508 | CCC(CC)NC(=O)C[C@@H](C(=O)NC[C@@H](O)[C@H](Cc1ccccc1)NC(=O)[C@@H](NC(=O)c2cnc3ccccc3n2)[C@H](C)O)C(C)(C)C | Protease |
| CHEMBL109371 | CC(C)[C@@H](NC(=O)OCc1ccccc1)C(=O)N[C@@H](Cc2ccccc2)[C@H](O)[C@H](Cc3ccccc3)NC(=O)[C@@H](NC(=O)OCc4ccccn4)C(C)C | Protease |
| CHEMBL362312 | O[C@H](CN(OC1CCCCC1)S(=O)(=O)c2ccc3OCCOc3c2)[C@H](Cc4ccccc4)NC(=O)O[C@H]5CO[C@H]6OCC[C@@H]56 | Protease |
| CHEMBL133552 | CC(C)(C)N(C[C@@H](O)[C@H](Cc1ccccc1)NC(=O)[C@H](CC(=O)N)NC(=O)c2ccc3ccccc3n2)C(=O)NCc4ccccc4 | Protease |
| CHEMBL1794048 | CC[C@H](C)[C@H](NC(=O)[C@H]1CCCN1C(=O)C(O)[C@H](Cc2ccccc2)NC(=O)[C@@H](CC(=O)N)NC(=O)OCc3ccccc3)C(=O)N[C@@H](C(C)C)C(=O)OC | Protease |
| CHEMBL123287 | Cc1cc(SC2=C(O)OC(CCc3ccc(N)cc3)(CC2=O)c4ccccc4)c(cc1CO)C(C)(C)C | Protease |
| CHEMBL71509 | CC(C)C1(CCc2ccc(O)cc2)CC(=O)C(=C(O)O1)Sc3cc(C)c(OS(=O)(=O)N4CCN(C)CC4)cc3C(C)(C)C | Protease |
| CHEMBL117271 | Cc1ccc(C(=O)N[C@@H](Cc2ccc3ccccc3c2)[C@H](O)C(=O)N4C[C@@H](Cl)C[C@H]4C(=O)NC(C)(C)C)c(C)c1O | Protease |
| CHEMBL93566 | CC(C)(C)OC(=O)N[C@H](C[C@H](O)[C@H](Cc1ccccc1)NC(=O)c2cc(N)ccc2Cl)Cc3ccccc3 | Protease |
| CHEMBL74077 | CC(C)(C)NC(=O)[C@@H]1C[C@@H]2CCCC[C@@H]2CN1C[C@@H](O)[C@H](Cc3ccccc3)NC(=O)[C@@H](NC(=O)c4ccc5ccccc5n4)[C@@H]6CCSC6 | Protease |
| CHEMBL288033 | CC(C)C1(CCc2ccccc2)CC(=O)C(=C(O)O1)Sc3cc(C)c(N)cc3C(C)(C)C | Protease |
| CHEMBL93540 | CC(C)(C)OC(=O)N[C@H](C[C@H](O)[C@H](Cc1ccccc1)NC(=O)c2ccc(O)cc2)Cc3ccccc3 | Protease |
| CHEMBL318217 | CC(C)(C)NC(=O)[C@@H]1C[C@@H]2SCC[C@@H]2CN1C[C@@H](O)[C@H](CSc3ccccc3)NC(=O)OCc4ccccc4 | Protease |
| CHEMBL169074 | CCNS(=O)(=O)Nc1cc(c(SC2=C(O)OC(CCc3ccccc3)(CC2=O)C(C)C)cc1C)C(C)(C)C | Protease |
| CHEMBL327611 | COc1cc(ccc1O)C(=O)N[C@@H](Cc2ccccc2)[C@@H](O)C[C@H](Cc3ccccc3)NC(=O)OC(C)(C)C | Protease |
| CHEMBL93481 | CC(C)(C)OC(=O)N[C@H](C[C@H](O)[C@H](Cc1ccccc1)NC(=O)c2ccc(N)cc2Cl)Cc3ccccc3 | Protease |
| CHEMBL134160 | COC(=O)[C@@H](NC(=O)[C@H](CC(C)C)NC(=O)[C@@H]1CCCN1C[C@@H](O)[C@H](Cc2ccccc2)NC(=O)[C@H](CC(=O)N)NC(=O)[C@H](CC(C)C)NC(=O)C(CO)NC(=O)C)C(C)C | Protease |
| CHEMBL312293 | CC(C)C1(CCc2ccc(O)cc2)CC(=O)C(=C(O)O1)Sc3cc(C)c(OS(=O)(=O)N(C)C)cc3C(C)(C)C | Protease |
| CHEMBL101962 | COc1cc(CC(=O)N[C@@H](C(C)C)C(=O)N[C@@H](Cc2ccccc2)[C@@H](O)[C@H](O)[C@H](Cc3ccccc3)NC(=O)[C@@H](NC(=O)Cc4cc(OC)c(OC)c(OC)c4)C(C)C)cc(OC)c1OC | Protease |
| CHEMBL265095 | CC(C)C1(CCc2ccc(O)cc2)CC(=O)C(=C(O)O1)Sc3cc(C)c(NC(=O)C)cc3C(C)(C)C | Protease |
| CHEMBL431387 | CC(C)[C@@H]1NC(=O)CCCCCCCOc2ccc(C[C@H](NC1=O)[C@H](O)CN3CC4CCCCC4C[C@H]3C(=O)NC(C)(C)C)cc2 | Protease |
| CHEMBL325267 | CCOc1ccc(CN2CCN(C[C@@H](O)C[C@@H](Cc3ccccc3)C(=O)NC4[C@H](O)Cc5ccccc45)[C@@H](C2)C(=O)NC(C)(C)C)cn1 | Protease |
| CHEMBL327147 | Cc1cccc(C[C@H](NC(=O)c2c(C)ccc(O)c2C)[C@H](O)C(=O)N3C[C@@H](Cl)C[C@H]3C(=O)NC(C)(C)C)c1 | Protease |
| CHEMBL273562 | CC(C)(C)OC(=O)N[C@H](C[C@H](O)[C@H](Cc1ccccc1)NC(=O)c2cccc(O)c2)Cc3ccccc3 | Protease |
| CHEMBL21001 | CCC(C)C(NC(=O)[C@@H]1CCCN1C[C@@H](O)[C@@H](Cc2ccccc2)NC(=O)C(CC(=O)N)NC(=O)[C@H](CC(C)C)NC(=O)C(CO)NC(=O)C)C(=O)NC(C(C)C)C(=O)OC | Protease |
| CHEMBL58522 | CC(C)[C@H](NC(=O)[C@@H](C[C@H](O)[C@H](Cc1ccccc1)NC(=O)OC(C)(C)C)Cc2ccccc2)C(=O)NCCCN(C)C | Protease |
| CHEMBL27120 | Cc1ccc(C[C@@H](O)[C@H](Cc2ccccc2)NC(=O)[C@H](CC(=O)N)NC(=O)c3ccc4ccccc4n3)c(c1)C(=O)NC(C)(C)C | Protease |
| CHEMBL354616 | Cc1cc(SC2=C(O)OC(C)(CCc3ccc(O)cc3)CC2=O)c(cc1NS(=O)(=O)c4ccc(Cl)cc4)C(C)(C)C | Protease |
| CHEMBL116439 | Cc1ccc(C(=O)N[C@@H](Cc2cccc(c2)C(F)(F)F)[C@H](O)C(=O)N3C[C@@H](Cl)C[C@H]3C(=O)NC(C)(C)C)c(C)c1O | Protease |
| CHEMBL148371 | Clc1ccc2[nH]c(c3ncc[nH]3)c(c2c1)S(=O)(=O)c4ccccc4 | Protease |
| CHEMBL36608 | CC(C)C1(CCc2ccccc2)CC(=O)C(=C(O)O1)Sc3cc(C)c(NC(=O)C)cc3C(C)(C)C | Protease |
| CHEMBL353086 | CC(C)C1(CCc2ccccc2)CC(=O)C(=C(O)O1)Sc3cc(C)c(NS(=O)(=O)c4cccnc4)cc3C(C)(C)C | Protease |
| CHEMBL29312 | CC(C)(C)NC(=O)c1c(C[C@@H](O)[C@H](Cc2ccccc2)NC(=O)[C@H](CC(=O)N)NC(=O)c3ccc4ccccc4n3)ccc5ccccc15 | Protease |
| CHEMBL273368 | CC(C)(C)NC(=O)c1ccccc1C[C@@H](O)[C@H](Cc2ccccc2)NC(=O)[C@H](C[S+]([O-])c3ccc4ccccc4c3)NS(=O)(=O)C | Protease |
| CHEMBL77671 | CC[C@H](C)[C@H](NC(=O)[C@@H]1CCCN1C[C@H](Cc2ccccc2)NC(=O)[C@H](CC(=O)N)NC(=O)[C@H](CCC(=O)N)NC(=O)[C@H](CO)NC(=O)[C@@H](N)C(C)C)C(=O)N[C@@H](C(C)C)C(=O)O | Protease |
| CHEMBL171786 | CC(C)C1(CCc2ccc(O)cc2)CC(=O)C(=C(O)O1)Sc3cc(C)c(OS(=O)(=O)c4cccnc4)cc3C(C)(C)C | Protease |
| CHEMBL324388 | Cc1cccc(C)c1C(=O)N[C@@H](Cc2ccccc2)[C@H](O)C(=O)N3C[C@@H](Cl)C[C@H]3C(=O)NC(C)(C)C | Protease |
| CHEMBL354783 | CC(C)C1(CCc2ccc(O)cc2)CC(=O)C(=C(O)O1)Sc3cc(C)c(NS(=O)(=O)N(C)C)cc3C(C)(C)C | Protease |
| CHEMBL325199 | Cc1ccc(C[C@H](NC(=O)c2c(C)ccc(O)c2C)[C@H](O)C(=O)N3C[C@@H](Cl)C[C@H]3C(=O)NC(C)(C)C)cc1 | Protease |
| CHEMBL351155 | CC(C)C1(CCc2ccccc2)CC(=O)C(=C(O)O1)Sc3cc(C)c(NS(=O)(=O)c4cccs4)cc3C(C)(C)C | Protease |
| CHEMBL1790509 | CCC(CC)NC(=O)C[C@@H](C(=O)NC[C@@H](O)[C@H](Cc1ccccc1)NC(=O)[C@@H](NC(=O)c2ccc3ccccc3n2)[C@H](C)O)C(C)(C)C | Protease |
| CHEMBL168269 | Cc1cc(SC2=C(O)OC(CCc3ccc(O)cc3)(CC2=O)C4CC4)c(cc1NS(=O)(=O)c5ccccc5)C(C)(C)C | Protease |
| CHEMBL123883 | CC(C)C1(CCc2ccc(O)cc2)CC(=O)C(=C(O)O1)Sc3cc(C)c(CO)cc3C(C)(C)C | Protease |
| CHEMBL433616 | CC(C)CC1(CCc2ccc(O)cc2)CC(=O)C(=C(O)O1)Sc3cc(C)c(OS(=O)(=O)Cn4ccnc4)cc3C(C)(C)C | Protease |
| CHEMBL56247 | CC(C)[C@@H]1[C@@H](CCS1(=O)=O)OC(=O)N[C@@H](Cc2ccccc2)[C@H](O)CN3CCN(Cc4ccsc4)C[C@H]3C(=O)NC(C)(C)C | Protease |
| CHEMBL123042 | Cc1cc(SC2=C(O)OC(CCc3ccccc3)(CC2=O)c4ccc(OCCO)cc4)c(cc1O)C(C)(C)C | Protease |
| CHEMBL170117 | Cc1cc(SC2=C(O)OC(C)(CCc3ccc(O)cc3)CC2=O)c(cc1NS(=O)(=O)c4ccc(F)cc4)C(C)(C)C | Protease |
| CHEMBL104576 | CC1(C)S[C@@H](N[C@H]1C(=O)N[C@H](Cc2ccccc2)[C@H](O)CC(=O)Nc3nc4ccccc4[nH]3)[C@H](NC(=O)Cc5ccccc5)C(=O)NCc6ccccc6 | Protease |
| CHEMBL198734 | COc1cc(CN2[C@H](Cc3ccccc3)[C@H](O)CN(N(Cc4ccc(O)c(OC)c4)C2=O)C(=O)CCCN5CCOCC5)ccc1O | Protease |
| CHEMBL69755 | CCC(CC)NC(=O)C[C@@H](C(=O)NC[C@@H](O)[C@H](Cc1ccccc1)NC(=O)[C@H](CC(=O)N)NC(=O)OCc2ccccc2)C(C)(C)C | Protease |
| CHEMBL290442 | CCCC1(CCc2ccc(O)cc2)CC(=O)C(=C(O)O1)Sc3cc(C)c(N)cc3C(C)(C)C | Protease |
| CHEMBL379540 | CC(C)CN([C@H](CO)CCCCNC(=O)[C@@H](NC(=O)C1CCCCC1)C(c2ccccc2)c3ccccc3)S(=O)(=O)c4ccc(N)cc4 | Protease |
| CHEMBL330565 | CC(C)[C@@H]1[C@@H](CCS1(=O)=O)OC(=O)N[C@@H](Cc2ccccc2)[C@H](O)CN3CCN(C[C@H]3C(=O)NC(C)(C)C)C4CC(C4)c5ccccc5 | Protease |
| CHEMBL68032 | CC(C)C1(CCc2ccccc2)CC(=O)C(=C(O)O1)Sc3cc(C)c(OS(=O)(=O)N4CCN(C)CC4)cc3C(C)(C)C | Protease |
| CHEMBL306421 | CC(C)C1(CCc2ccc(N)cc2)CC(=O)C(=C(O)O1)Sc3cc(C)c(OS(=O)(=O)N(C)C)cc3C(C)(C)C | Protease |
| CHEMBL116552 | Cc1ccc(O)c(C)c1C(=O)N[C@@H](Cc2ccc(cc2)C(F)(F)F)[C@H](O)C(=O)N3C[C@@H](Cl)C[C@H]3C(=O)NC(C)(C)C | Protease |
| CHEMBL123022 | Cc1cc(SC2=C(O)OC(CCc3cccc(N)c3)(CC2=O)c4ccccc4)c(cc1CO)C(C)(C)C | Protease |
| CHEMBL20642 | CC(C)C(NC(=O)C(F)(F)C(=O)C(Cc1ccc(OCc2ccccc2)cc1)NC(=O)[C@@H](NC(=O)OCc3ccccc3)C(C)C)C=O | Protease |
| CHEMBL305458 | CC(C)[C@@H](N[C@@H](C)C(=O)OCc1ccccc1)C(=O)N[C@@H](Cc2ccccc2)[C@@H](O)[C@H](O)[C@H](Cc3ccccc3)NC(=O)[C@@H](NC(=O)[C@H](C)NC(=O)OCc4ccccc4)C(C)C | Protease |
| CHEMBL20143 | CC(C)[C@@H](NC(=O)C(F)(F)C(=O)C(Cc1ccc(OCc2ccccc2)cc1)NC(=O)[C@@H](NC(=O)OCc3ccccc3)C(C)C)C(=O)O | Protease |
| CHEMBL113627 | CC(C)[C@@H](NC(=O)OCc1ccccc1)C(=O)N[C@@H](Cc2ccccc2)[C@H](O)[C@H](Cc3ccccc3)NC(=O)[C@@H](NC(=O)OCc4cccnc4)C(C)C | Protease |
| CHEMBL13134 | CC(C)[C@H](NC(=O)c1ccc2ccccc2n1)C(=O)N[C@@H](Cc3ccccc3)[C@H](O)CN4CC[C@H](C[C@H]4C(=O)NC(C)(C)C)OCc5ccncc5 | Protease |
| CHEMBL19989 | CCC(C)C(NC(=O)[C@@H]1CCCN1C[C@@H](O)[C@@H](Cc2ccccc2)NC(=O)C(CC(=O)N)NC(=O)OCc3ccccc3)C(=O)NC(Cc4ccccc4)C(=O)OC | Protease |
| CHEMBL87092 | CC(C)(C)OC(=O)NC(Cc1ccccc1)C(=O)C(F)(F)C(=O)NCc2ccccc2 | Protease |
| CHEMBL1790886 | CC[C@@H](C)[C@H](NC(=O)[C@@H]1CCCN1C[C@@H](O)[C@H](Cc2ccccc2)NC(=O)[C@H](CC(=O)N)NC(=O)OCc3ccccc3)C(=O)N[C@@H](Cc4ccccc4)C(=O)OC | Protease |
| CHEMBL352964 | CC(C)C1(CCc2ccc(O)cc2)CC(=O)C(=C(O)O1)Sc3cc(C)c(NS(=O)(=O)C)cc3C(C)(C)C | Protease |
| CHEMBL274534 | CC(C)(C)OC(=O)CNC(=O)[C@@H](NC(=O)c1ccccc1c2ccccc2)[C@@H]3N[C@@H](C(=O)NCCNC(=O)[C@@H]4N[C@H](SC4(C)C)[C@H](NC(=O)c5ccccc5c6ccccc6)C(=O)NCC(=O)OC(C)(C)C)C(C)(C)S3 | Protease |
| CHEMBL167243 | CC(C)C1(CCc2ccc(N)cc2)CC(=O)C(=C(O)O1)Sc3cc(C)c(OS(=O)(=O)c4cccnc4)cc3C(C)(C)C | Protease |
| CHEMBL157673 | CCCN(CCc1csc(n1)C(C)C)C(=O)NC(C)C(=O)N[C@H](C[C@H](O)[C@H](Cc2ccccc2)NC(=O)OCc3cncs3)Cc4ccccc4 | Protease |
| CHEMBL35271 | Cc1cc(SC2=C(O)OC(CCc3ccc(O)cc3)(CC2=O)C4CC4)c(cc1N)C(C)(C)C | Protease |
| CHEMBL171720 | CC(C)C1(CCc2ccc(O)cc2)CC(=O)C(=C(O)O1)Sc3cc(C)c(NS(=O)(=O)c4ccc(F)cc4)cc3C(C)(C)C | Protease |
| CHEMBL328531 | CC(C)(C)OC(=O)N[C@H](C[C@H](O)[C@H](Cc1ccccc1)NC(=O)c2ccc(O)c(O)c2)Cc3ccccc3 | Protease |
| CHEMBL122847 | Cc1cc(SC2=C(O)OC(C)(CCc3ccc(O)cc3)CC2=O)c(cc1CO)C(C)(C)C | Protease |
| CHEMBL3085539 | CC(C)[C@H](NC(=O)OCc1ccccc1)C(=O)N[C@@H](Cc2ccccc2)[C@H](O)C(=O)N3CCC[C@@H]3C(=O)NC(C)(C)C | Protease |
| CHEMBL305204 | CCNS(=O)(=O)Oc1cc(c(SC2=C(O)OC(CCc3ccc(N)cc3)(CC2=O)C(C)C)cc1C)C(C)(C)C | Protease |
| CHEMBL38384 | CCCCC1(CCc2ccc(O)cc2)CC(=O)C(=C(O)O1)Sc3cc(C)c(N)cc3C(C)(C)C | Protease |
| CHEMBL432969 | CC1(C)S[C@@H](N[C@H]1C(=O)NCCNC(=O)C2N[C@H](SC2(C)C)[C@H](NC(=O)Cc3ccccc3)C(=O)NCC(F)(F)F)[C@H](NC(=O)Cc4ccccc4)C(=O)NCC(F)(F)F | Protease |
| CHEMBL11426 | CC(C)(C)NC(=O)c1ccccc1C[C@@H](O)[C@H](Cc2ccccc2)NC(=O)[C@H](CSc3ccc(F)cc3)NS(=O)(=O)C | Protease |
| CHEMBL432962 | CC1(C)S[C@@H](N[C@H]1C(=O)NCCNC(=O)[C@@H]2N[C@H](SC2(C)C)[C@H](NC(=O)Cc3ccccc3)C(=O)NCC(F)(F)F)[C@H](NC(=O)Cc4ccccc4)C(=O)NCC(F)(F)F | Protease |
| CHEMBL122802 | COCc1cc(c(SC2=C(O)OC(CCc3ccccc3)(CC2=O)c4ccc(OCCO)cc4)cc1C)C(C)(C)C | Protease |
| CHEMBL168839 | CC(C)C1(CCc2ccc(O)cc2)CC(=O)C(=C(O)O1)Sc3cc(C)c(OS(=O)(=O)c4ccc(cc4)C#N)cc3C(C)(C)C | Protease |
| CHEMBL334426 | CC[C@@H](C)[C@H](NC(=O)[C@H](C(C)C)C(O)C(O)[C@H](CC1CCCCC1)NC(=O)C(Cc2c[nH]cn2)NC(=O)COc3ccccc3)C(=O)NCc4ccccn4 | Protease |
| CHEMBL91600 | CC(C)(C)OC(=O)N[C@H](C[C@H](O)[C@H](Cc1ccccc1)NC(=O)c2ccccc2)Cc3ccccc3 | Protease |
| CHEMBL325824 | Cc1ccc(O)c(C)c1C(=O)N[C@@H](Cc2cccc(c2)C(F)(F)F)[C@H](O)C(=O)N3C[C@@H](Cl)C[C@H]3C(=O)NC(C)(C)C | Protease |
| CHEMBL106765 | CC1(C)S[C@@H](N[C@H]1C(=O)N[C@H](Cc2ccccc2)[C@H](O)CC(=O)NCc3ccccc3)[C@H](NC(=O)Cc4ccccc4)C(=O)NCc5ccccc5 | Protease |
| CHEMBL58277 | CC(C)(C)NC(=O)c1ccccc1C[C@@H](O)C[C@@H](Cc2ccccc2)C(=O)N[C@@H]3[C@H](O)Cc4ccccc34 | Protease |
| CHEMBL253018 | Cc1cnc([nH]1)c2[nH]c3ccc(Cl)cc3c2S(=O)(=O)c4ccccc4 | Protease |
| CHEMBL90313 | CC(C)[C@@H]1[C@@H](CCS1(=O)=O)OC(=O)N[C@@H](Cc2ccccc2)[C@H](O)CN3CCN(C[C@H]3C(=O)NC(C)(C)C)C4CC4 | Protease |
| CHEMBL105851 | CCNC(=O)[C@@H](NC(=O)Cc1ccccc1)[C@@H]2N[C@@H](C(=O)NCCNC(=O)[C@@H]3N[C@H](SC3(C)C)[C@H](NC(=O)Cc4ccccc4)C(=O)NCC)C(C)(C)S2 | Protease |
| CHEMBL306297 | CCNS(=O)(=O)Oc1cc(c(SC2=C(O)OC(CCc3ccc(O)cc3)(CC2=O)C4CCCCC4)cc1C)C(C)(C)C | Protease |
| CHEMBL106486 | CC(C)[C@@H](NC(=O)OCc1ccccn1)C(=O)N[C@@H](Cc2ccccc2)C(O)[C@H](Cc3ccccc3)NC(=O)[C@@H](NC(=O)OCc4ccccn4)C(C)C | Protease |
| CHEMBL70986 | CCNS(=O)(=O)Oc1cc(c(SC2=C(O)OC(CCc3ccc(O)cc3)(CC2=O)C(C)C)cc1C)C(C)(C)C | Protease |
| CHEMBL90174 | CC[C@H](C)[C@@H](NC(=O)OCc1ccccc1)C(=O)N[C@@H](Cc2ccccc2)C(O)[C@H](Cc3ccccc3)NC(=O)[C@@H](NC(=O)OCc4ccccc4)[C@@H](C)CC | Protease |
| CHEMBL340368 | CCNC(=O)[C@@H](NC(=O)Cc1ccccc1)[C@@H]2N[C@@H](C(=O)NCCNC(=O)[C@@H]3N[C@H](SC3(C)C)C(NC(=O)Cc4ccccc4)C(=O)NCC)C(C)(C)S2 | Protease |
| CHEMBL317882 | CC1(C)S[C@@H](N[C@H]1C(=O)NCC(O)CNC(=O)[C@@H]2N[C@H](SC2(C)C)[C@H](NC(=O)Cc3ccccc3)C(=O)NCc4ccccc4)[C@H](NC(=O)Cc5ccccc5)C(=O)NCc6ccccc6 | Protease |
| CHEMBL169535 | CC(C)C1(CCc2ccc(O)cc2)CC(=O)C(=C(O)O1)Sc3cc(C)c(OS(=O)(=O)c4ccccn4)cc3C(C)(C)C | Protease |
| CHEMBL37597 | CC(C)C1(CCc2ccc(O)cc2)CC(=O)C(=C(O)O1)Sc3cc(C)c(NC(=O)c4ccc(cc4)C#N)cc3C(C)(C)C | Protease |
| CHEMBL19752 | CC(C)[C@H](COCc1ccccc1)NC(=O)C(F)(F)[C@H](O)C(Cc2ccc(OCc3ccccc3)cc2)NC(=O)[C@@H](NC(=O)OCc4ccccc4)C(C)C | Protease |
| CHEMBL125448 | OC(=O)CCCCC1(CC(=O)C(=C(O)O1)SCCc2ccccc2)c3ccccc3 | Protease |
| CHEMBL305460 | CC(C)(C)NC(=O)N(CC(O)[C@H](Cc1ccccc1)NC(=O)[C@H](CC(=O)N)NC(=O)c2ccc3ccccc3n2)C4CCCCC4 | Protease |
| CHEMBL359756 | CC(C)CN([C@@H](CCCCNC(=O)OCC1c2ccccc2c3ccccc13)C(=O)O)S(=O)(=O)c4ccc(C)cc4 | Protease |
| CHEMBL139538 | COc1ccc(cc1)S(=O)(=O)N(CC(C)C)C[C@@H](O)[C@H](Cc2ccccc2)NC(=O)c3ccccc3C | Protease |
| CHEMBL19973 | CC(C)NC(=O)C(F)(F)C(=O)C(Cc1ccc(OCc2ccccc2)cc1)NC(=O)[C@@H](NC(=O)OCc3ccccc3)C(C)C | Protease |
| CHEMBL430117 | CC1(C)S[C@@H](N[C@H]1C(=O)NCC(O)CNC(=O)C2N[C@H](SC2(C)C)[C@H](NC(=O)Cc3ccccc3)C(=O)NCc4ccccc4)[C@H](NC(=O)Cc5ccccc5)C(=O)NCc6ccccc6 | Protease |
| CHEMBL78217 | CC(C)C1(CCC2CCCO2)CC(=O)C(=C(O)O1)Sc3cc(C)c(CO)cc3C(C)(C)C | Protease |
| CHEMBL361082 | O[C@H](CN(OC1CCCCC1)S(=O)(=O)c2ccc(O)cc2)[C@H](Cc3ccccc3)NC(=O)O[C@H]4CO[C@H]5OCC[C@@H]45 | Protease |
| CHEMBL39130 | CCCC[C@H](CC(O)C[C@@H](CCCC)C(=O)N[C@@H](C(C)C)C(=O)N[C@@H](C(C)C)C(=O)OC)C(=O)N[C@@H](C(C)C)C(=O)N[C@@H](C(C)C)C(=O)OC | Protease |
| CHEMBL264739 | CC(C)CNC(=O)C(F)(F)C(=O)C(Cc1ccc(OCc2ccccc2)cc1)NC(=O)[C@@H](NC(=O)OCc3ccccc3)C(C)C | Protease |
| CHEMBL281439 | COC(=O)[C@@H](NC(=O)[C@@H](NC(=O)[C@@H](CC(O)C[C@@H](Cc1ccccc1)C(=O)N[C@@H](C(C)C)C(=O)N[C@@H](C(C)C)C(=O)OC)Cc2ccccc2)C(C)C)C(C)C | Protease |
| CHEMBL139444 | COc1ccc(cc1)S(=O)(=O)N(CC(C)C)C[C@@H](O)[C@H](Cc2ccccc2)NC(=O)c3ccc(O)cc3C | Protease |
| CHEMBL38154 | CC(C)C1(CCc2ccc(O)cc2)CC(=O)C(=C(O)O1)Sc3cc(C)c(NC(=O)c4ccncc4)cc3C(C)(C)C | Protease |
| CHEMBL418978 | CC(C)(C)NC(=O)[C@@H]1C[C@@H]2CCCC[C@@H]2CN1C[C@@H](O)[C@H](Cc3ccccc3)NC(=O)[C@@H](CSc4ccc5ccccc5c4)NS(=O)(=O)C | Protease |
| CHEMBL148372 | Cc1nc([nH]c1C)c2[nH]c3ccc(Cl)cc3c2S(=O)(=O)c4ccccc4 | Protease |
| CHEMBL432665 | CC(C)C1(CCc2ccc(O)cc2)CC(=O)C(=C(O)O1)Sc3cc(C)c(NC(=O)c4cccnc4)cc3C(C)(C)C | Protease |
| CHEMBL99016 | CC(C)(C)OC(=O)N[C@@H](Cc1ccccc1)[C@@H](O)C[C@@H](Cc2ccccc2)C(=O)N[C@@H](C3CCCCC3)C(=O)N | Protease |
| CHEMBL323955 | CC(C)[C@@H](NC(=O)N(C)CCc1ccccn1)C(=O)N[C@@H](Cc2ccccc2)[C@@H](O)[C@H](O)[C@H](Cc3ccccc3)NC(=O)[C@@H](NC(=O)N(C)CCc4ccccn4)C(C)C | Protease |
| CHEMBL322285 | CN(C)C(=O)[C@@H](NC(=O)Cc1ccccc1)[C@@H]2N[C@@H](C(=O)NCCNC(=O)[C@@H]3N[C@H](SC3(C)C)[C@H](NC(=O)Cc4ccccc4)C(=O)N(C)C)C(C)(C)S2 | Protease |
| CHEMBL320602 | C[C@@H]1[C@H](CCS1(=O)=O)OC(=O)N[C@@H](CSc2ccccc2)[C@H](O)CN3C[C@H]4CCS[C@H]4C[C@H]3C(=O)NC(C)(C)C | Protease |
| CHEMBL93323 | CC(C)(C)OC(=O)N[C@H](C[C@H](O)[C@H](Cc1ccccc1)NC(=O)c2cccc(N)c2Cl)Cc3ccccc3 | Protease |
| CHEMBL169543 | CC(C)C1(CCc2ccc(O)cc2)CC(=O)C(=C(O)O1)Sc3cc(C)c(NS(=O)(=O)c4ccc(Cl)cc4)cc3C(C)(C)C | Protease |
| CHEMBL94078 | CC(C)(C)OC(=O)N[C@H](C[C@H](O)[C@H](Cc1ccccc1)NC(=O)OC(C)(C)C)Cc2ccccc2 | Protease |
| CHEMBL111957 | CC(C)[C@@H](NC(=O)\C=C\c1cccnc1)C(=O)N[C@@H](Cc2ccccc2)[C@H](O)[C@H](O)[C@H](Cc3ccccc3)NC(=O)[C@@H](NC(=O)\C=C\c4cccnc4)C(C)C | Protease |
| CHEMBL91610 | CC(C)[C@@H]1[C@@H](CCS1(=O)=O)OC(=O)N[C@@H](Cc2ccccc2)[C@H](O)CN3CCN(C[C@H]3C(=O)NC(C)(C)C)C4Cc5ccccc5C4 | Protease |
| CHEMBL312788 | CC(C)(C)NC(=O)[C@@H]1C[C@@H]2CCCC[C@@H]2CN1C[C@@H](O)[C@H](Cc3ccccc3)NC(=O)[C@@H](NC(=O)c4ccc5ccccc5n4)[C@@H]6CCOC6 | Protease |
| CHEMBL422112 | CC(C)(C)OC(=O)N[C@@H](Cc1ccccc1)[C@@H](O)C[C@H](C(=O)NC2[C@@H](O)Cc3ccccc23)C(=O)c4ccccc4 | Protease |
| CHEMBL268512 | CC(C)(C)OC(=O)N[C@@H](Cc1ccccc1)[C@@H](O)C[C@@H](CC(=O)c2ccccc2)C(=O)N[C@@H]3[C@H](O)Cc4ccccc34 | Protease |
| CHEMBL315253 | NC(=O)CC(NC(=O)c1ccc2ccccc2n1)C(=O)N[C@@H](Cc3ccccc3)[C@H](O)CN[C@@H](Cc4ccccc4)C(=O)NC5[C@H](O)Cc6ccccc56 | Protease |
| CHEMBL195072 | CC(C)C[C@H](NC(=O)[C@@H](NC(=O)CCCCCCCCCCCCCCC(=O)N[C@H](CC(=O)N[C@@H](Cc1ccccc1)C(=O)O)C(=O)N)C(C)O)C(=O)N[C@@H](Cc2ccccc2)C(=O)N | Protease |
| CHEMBL429036 | CC(C)(C)NC(=O)c1ccccc1C[C@@H](O)[C@H](Cc2ccccc2)NC(=O)[C@H](CC(=O)OCc3ccccc3)NS(=O)(=O)C | Protease |
| CHEMBL324971 | Cc1c(N)cccc1C(=O)N[C@@H](Cc2ccccc2)[C@H](O)C(=O)N3C[C@@H](Cl)C[C@H]3C(=O)NC(C)(C)C | Protease |
| CHEMBL325356 | CCNC(=O)[C@@H](NC(=O)c1c(C)onc1c2ccccc2)[C@@H]3N[C@@H](C(=O)NCCNC(=O)[C@@H]4N[C@H](SC4(C)C)[C@H](NC(=O)c5c(C)onc5c6ccccc6)C(=O)NCC)C(C)(C)S3 | Protease |
| CHEMBL17600 | CC[C@H]1[C@H](CCS1(=O)=O)OC(=O)N[C@@H](Cc2ccccc2)[C@H](O)CN3C[C@H]4CCCC[C@H]4C[C@H]3C(=O)NC(C)(C)C | Protease |
| CHEMBL319400 | CCNC(=O)[C@@H](NC(=O)c1c(C)onc1c2ccccc2)[C@@H]3N[C@@H](C(=O)NCCNC(=O)C4N[C@H](SC4(C)C)[C@H](NC(=O)c5c(C)onc5c6ccccc6)C(=O)NCC)C(C)(C)S3 | Protease |
| CHEMBL318319 | CC(C)(C)OC(=O)N[C@@H](Cc1ccccc1)[C@@H](O)C[C@@H](CC(=O)c2ccccc2)C(=O)N[C@H]3[C@H](O)Cc4ccccc34 | Protease |
| CHEMBL274912 | CC(C)[C@H](NC(=O)[C@H](Cc1cccc2ccccc12)CS(=O)(=O)C(C)(C)C)C(=O)NCP(=O)(N)CNC(=O)[C@@H](NC(=O)[C@H](Cc3cccc4ccccc34)CS(=O)(=O)C(C)(C)C)C(C)C | Protease |
| CHEMBL305466 | CC(C)[C@@H]1NC(=O)c2cc3ccccc3cc2OCCCOc4ccc(C[C@H](NC1=O)[C@H](O)CN5CC6CCCCC6C[C@H]5C(=O)NC(C)(C)C)cc4 | Protease |
| CHEMBL314753 | O[C@@H](C[C@H](Cc1ccccc1)NC(=O)OCc2ccccc2)[C@H](Cc3ccccc3)NC(=O)OCc4ccccc4 | Protease |
| CHEMBL300812 | O[C@@H](C[C@]1(Cc2ccccc2)N=CC(=C1O)C3CNC(=O)c4ccccc34)[C@H](Cc5ccccc5)NC(=O)O[C@H]6CCOC6 | Protease |
| CHEMBL112578 | CC(C)[C@@H](NS(=O)(=O)CCc1ccncc1)C(=O)N[C@@H](Cc2ccccc2)[C@H](O)[C@H](O)[C@H](Cc3ccccc3)NC(=O)[C@@H](NS(=O)(=O)CCc4ccncc4)C(C)C | Protease |
| CHEMBL371678 | O[C@@H](C[C@]1(Cc2ccccc2)N=CC(=C1O)[C@@H]3CNC(=O)c4ccccc34)[C@H](Cc5ccccc5)NC(=O)O[C@H]6CCOC6 | Protease |
| CHEMBL267092 | CC(C)(C)NC(=O)c1ccccc1C[C@@H](O)[C@H](Cc2ccccc2)NC(=O)[C@H](CS(=O)(=O)c3ccccc3)NS(=O)(=O)C | Protease |
| CHEMBL319013 | CC(C)(C)OC(=O)N[C@@H](Cc1ccccc1)[C@@H](O)C[C@@H](Cc2ccccc2)C(=O)N[C@H](C(=O)NCc3ccccc3)c4ccccc4 | Protease |
| CHEMBL77819 | CC(C)c1scnc1CCC2(CC(=O)C(=C(O)O2)Sc3cc(C)c(CO)cc3C(C)(C)C)C(C)C | Protease |
| CHEMBL2448127 | CC(C)c1ccc(C)cc1SC2=C(O)O[C@@](CCc3ccccc3)(CC2=O)c4ccccc4 | Protease |
| CHEMBL45953 | CC(C)C[C@H](NC(=O)[C@H](C)C[C@H](O)[C@H](Cc1ccccc1)NC(=O)OC(C)(C)C)C(=O)N[C@@H](Cc2ccccc2)C(=O)N | Protease |
| CHEMBL430445 | CC(C)(C)OC(=O)N[C@@H](Cc1ccccc1)[C@@H](O)C[C@]2(Cc3ccc(O)cc3)N=CC(=C2O)C4[C@H](O)Cc5ccccc45 | Protease |
| CHEMBL141265 | CC(C)CN(C[C@@H](O)[C@H](Cc1ccccc1)NC(=O)OCc2ccccc2)S(=O)(=O)c3ccccc3 | Protease |
| CHEMBL74166 | CC(C)[C@H](NC(=O)[C@H](Cc1ccc(O)cc1)NC(=O)C)C(=O)N[C@@H](Cc2ccccc2)[C@@H](O)C(=O)N[C@H]3Cc4ccc(OCCCNC(=O)[C@@H](NC3=O)C(C)C)cc4 | Protease |
| CHEMBL344233 | COc1ccc(cc1)S(=O)(=O)N(CC(C)C)C[C@@H](O)[C@H](Cc2ccccc2)NC(=O)c3ccccc3Cl | Protease |
| CHEMBL342367 | COc1ccc(cc1)S(=O)(=O)N(CC(C)C)C[C@@H](O)[C@H](Cc2ccccc2)NC(=O)c3cccc(N)c3C | Protease |
| CHEMBL58406 | CC(C)(C)NS(=O)(=O)c1ccccc1C[C@@H](O)C[C@@H](Cc2ccccc2)C(=O)N[C@@H]3[C@H](O)Cc4ccccc34 | Protease |
| CHEMBL99081 | CC(=O)N[C@H](CSCc1ccccc1)C(=O)N[C@@H](Cc2ccccc2)[C@H](O)CN3C[C@H]4CCCC[C@H]4C[C@H]3C(=O)NC(C)(C)C | Protease |
| CHEMBL37541 | Cc1cc(SC2=C(O)OC(CCc3ccc(O)cc3)(CC2=O)C4CCCC4)c(cc1N)C(C)(C)C | Protease |
| CHEMBL19545 | CC(C)[C@@H](NC(=O)C(F)(F)C(=O)C(Cc1ccc(OCc2ccccc2)cc1)NC(=O)[C@@H](NC(=O)OCc3ccccc3)C(C)C)C(=O)OC(C)(C)C | Protease |
| CHEMBL129 | CC1=CN([C@H]2C[C@H](N=[N+]=[N-])[C@@H](CO)O2)C(=O)NC1=O | Protease |
| CHEMBL82829 | FC(F)(C(=O)NCc1ccccc1)C(=O)C(Cc2ccc(OCc3ccccc3)cc2)NC(=O)C(NC(=O)OCc4ccccc4)C5CCCC5 | Protease |
| CHEMBL264818 | COc1ccc(cc1)S(=O)(=O)N(C[C@@H](O)[C@H](Cc2ccccc2)NC(=O)O[C@@H]3COC4OCCC34)OC5CCCC5 | Protease |
| CHEMBL305463 | CCCCCCC(=O)N[C@@H](C(C)C)C(=O)N[C@@H](Cc1ccccc1)[C@H](O)CN2CC3CCCCC3C[C@H]2C(=O)NC(C)(C)C | Protease |
| CHEMBL3143353 | CC(C)C[C@H](NC(=O)[C@@H](C[C@H](O)[C@H](Cc1ccccc1)NC(=O)CC(C)(C)C)Cc2ccccc2)C(=O)N[C@@H](Cc3ccccc3)C(=O)N | Protease |
| CHEMBL322241 | CC(C)CN(C[C@@H](O)[C@H](Cc1ccccc1)NC(=O)[C@H](CC(=O)N)NC(=O)c2ccc3ccccc3n2)C(=O)NC(C)(C)C | Protease |
| CHEMBL305731 | CC(C)CN(CC(O)[C@H](Cc1ccccc1)NC(=O)[C@H](CC(=O)N)NC(=O)c2ccc3ccccc3n2)C(=O)NC(C)(C)C | Protease |
| CHEMBL335093 | CC(C)CNC(=O)N(C[C@@H](O)[C@H](Cc1ccccc1)NC(=O)[C@H](CC(=O)N)NC(=O)c2ccc3ccccc3n2)C(C)(C)C | Protease |
| CHEMBL333610 | CCN1CCN(C[C@@H](O)C[C@@H](Cc2ccccc2)C(=O)NC3[C@H](O)Cc4ccccc34)[C@@H](C1)C(=O)NC(C)(C)C | Protease |
| CHEMBL38236 | CC(C)C1(CCc2ccc(O)cc2)CC(=O)C(=C(O)O1)Sc3cc(C)c(NC(=O)c4ccccn4)cc3C(C)(C)C | Protease |
| CHEMBL347662 | Cc1ccc(cc1C(=O)N[C@@H](Cc2ccccc2)[C@H](O)Cc3ccccc3C(=O)NC(C)(C)C)C(=O)N4CCc5ccccc5C4 | Protease |
| CHEMBL91657 | Nc1ccc(C(=O)N[C@H](C[C@H](O)[C@H](Cc2ccccc2)NC(=O)OCc3ccccc3)Cc4ccccc4)c(Cl)c1 | Protease |
| CHEMBL434190 | COc1cc(CN2[C@H](Cc3ccccc3)[C@H](O)CN(N(Cc4ccc(O)c(OC)c4)C2=O)C(=O)CN5CCNCC5)ccc1O | Protease |
| CHEMBL308889 | CCCNS(=O)(=O)Oc1cc(c(SC2=C(O)OC(C)(CCc3ccc(O)cc3)CC2=O)cc1C)C(C)(C)C | Protease |
| CHEMBL17494 | CCC[C@H]1[C@H](CCS1(=O)=O)OC(=O)N[C@@H](Cc2ccccc2)[C@H](O)CN3C[C@H]4CCCC[C@H]4C[C@H]3C(=O)NC(C)(C)C | Protease |
| CHEMBL278416 | CC(C)(C)NC(=O)[C@@H]1C[C@@H]2CCCC[C@@H]2CN1C[C@@H](O)[C@H](Cc3ccccc3)NC(=O)O[C@H]4CCS(=O)(=O)[C@H]4C5CCCC5 | Protease |
| CHEMBL98229 | CCN(CC)CC(O)CNC(=O)[C@@H](NC(=O)[C@@H](C[C@H](O)[C@H](Cc1ccccc1)NC(=O)OC(C)(C)C)Cc2ccccc2)C(C)C | Protease |
| CHEMBL169871 | Cc1cc(SC2=C(O)OC(C)(CCc3ccc(O)cc3)CC2=O)c(cc1NS(=O)(=O)c4ccc(cc4)c5cccs5)C(C)(C)C | Protease |
| CHEMBL117769 | Cc1ccc(C(=O)N[C@@H](Cc2ccc(cc2)C(F)(F)F)[C@H](O)C(=O)N3C[C@@H](Cl)C[C@H]3C(=O)NC(C)(C)C)c(C)c1O | Protease |
| CHEMBL83768 | CC(C)(C)NC(=O)[C@@H]1C[C@@H]2CCCC[C@@H]2CN1C[C@@H](O)[C@H](Cc3ccccc3)NC(=O)O[C@@H]4CO[C@@H]5OCC[C@H]45 | Protease |
| CHEMBL333211 | Cc1cc(SC2=C(O)OC(CCc3ccccc3)(CC2=O)c4ccc(OCCO)cc4)c(cc1OCCO)C(C)(C)C | Protease |
| CHEMBL2374444 | COC(=O)N(C)[C@H](Cc1ccccc1)C(=O)NCCCC[C@@H](CO)N(CC(C)C)S(=O)(=O)c2ccc(N)cc2 | Protease |
| CHEMBL37668 | Cc1cc(SC2=C(O)OC(C)(CCc3ccc(O)cc3)CC2=O)c(cc1O)C(C)(C)C | Protease |
| CHEMBL264184 | Clc1ccc2[nH]c(C3=NCCN3)c(c2c1)S(=O)(=O)c4ccccc4 | Protease |
| CHEMBL99875 | CC(C)(C)NC(=O)[C@@H]1C[C@@H]2SCC[C@@H]2CN1C[C@@H](O)[C@H](Cc3ccccc3)NC(=O)OCc4ccccc4 | Protease |
| CHEMBL278084 | CC(C)[C@H](COCc1ccccc1)NC(=O)C(F)(F)[C@@H](O)C(Cc2ccc(OCc3ccccc3)cc2)NC(=O)[C@@H](NC(=O)OCc4ccccc4)C(C)C | Protease |
| CHEMBL331412 | Cc1cc(SC2=C(O)OC(CCc3ccccc3)(CC2=O)c4ccccc4)c(cc1CO)C(C)(C)C | Protease |
| CHEMBL149053 | CC1=CSC2C(CN3CCN(C[C@@H](O)C[C@@H](Cc4ccccc4)C(=O)NC5[C@H](O)Cc6ccccc56)[C@@H](C3)C(=O)NC(C)(C)C)N=CN12 | Protease |
| CHEMBL354615 | CC(C)C1(CCc2ccccc2)CC(=O)C(=C(O)O1)Sc3cc(C)c(OS(=O)(=O)c4ccccn4)cc3C(C)(C)C | Protease |
| CHEMBL151824 | Cn1cc(CN2CCN(C[C@@H](O)C[C@@H](Cc3ccccc3)C(=O)NC4[C@H](O)Cc5ccccc45)[C@@H](C2)C(=O)NC(C)(C)C)c6ccccc16 | Protease |
| CHEMBL333427 | Cc1cc(SC2=C(O)OC(CCc3ccccc3)(CC2=O)c4ccccc4)c(cc1OCCO)C(C)(C)C | Protease |
| CHEMBL112186 | CC(C)[C@@H](NC(=O)OCc1cccnc1)C(=O)N[C@@H](Cc2ccccc2)C(O)[C@H](Cc3ccccc3)NC(=O)[C@@H](NC(=O)OCc4cccnc4)C(C)C | Protease |
| CHEMBL106748 | CC1(C)S[C@@H](N[C@H]1C(=O)NCCNC(=O)[C@@H]2N[C@H](SC2(C)C)[C@H](NC(=O)Cc3ccccc3)C(=O)N4CCCCC4)[C@H](NC(=O)Cc5ccccc5)C(=O)N6CCCCC6 | Protease |
| CHEMBL59522 | CC(C)(C)c1c(SC2=C(O)OC(CCc3ccccc3)(CC2=O)c4ccccc4)sc5ccccc15 | Protease |
| CHEMBL190372 | O[C@H](CN(OC1CCCC1)S(=O)(=O)c2ccc3OCOc3c2)[C@H](Cc4ccccc4)NC(=O)O[C@H]5CO[C@H]6OCC[C@@H]56 | Protease |
| CHEMBL112546 | CC(C)[C@@H](NC(=O)OCc1ccccc1)C(=O)N[C@@H](Cc2ccccc2)[C@H](O)[C@H](Cc3ccccc3)NC(=O)[C@@H](NC(=O)OCc4ccncc4)C(C)C | Protease |
| CHEMBL108960 | CC(C)(C)OC(=O)N[C@@H](Cc1ccccc1)[C@@H](O)C[C@@](C)(Cc2ccccc2)C(=O)N[C@@H]3[C@H](O)Cc4ccccc34 | Protease |
| CHEMBL439853 | O[C@H](CN(OC1CCCC1)S(=O)(=O)c2ccc3OCCOc3c2)[C@H](Cc4ccccc4)NC(=O)O[C@H]5CO[C@H]6OCC[C@@H]56 | Protease |
| CHEMBL70388 | CC(C)[C@H](NC(=O)[C@H](Cc1ccccc1)NC(=O)C)C(=O)N[C@@H](Cc2ccccc2)[C@@H](O)C(=O)N[C@H]3Cc4ccc(OCCCNC(=O)[C@@H](NC3=O)C(C)C)cc4 | Protease |
| CHEMBL272599 | Cc1cccc(C)c1OCC(=O)N[C@@H](Cc2ccccc2)[C@H](O)CN3CC[C@H](C[C@H]3C(=O)NC(C)(C)C)OCc4ccncc4 | Protease |
| CHEMBL94266 | C[C@H](NC(=O)[C@@H](C[C@H](O)[C@H](Cc1ccccc1)NC(=O)OC(C)(C)C)Cc2ccccc2)C(=O)N | Protease |
| CHEMBL122426 | CC(C)c1ccc(C)cc1SC2=C(O)OC(CCc3ccccc3)(CC2=O)c4ccccc4 | Protease |
| CHEMBL419580 | CC(C)[C@@H](NC(=O)C1Cc2ccccc2CN1)C(=O)N[C@@H](Cc3ccccc3)C(O)[C@H](Cc4ccccc4)NC(=O)[C@@H](NC(=O)C5Cc6ccccc6CN5)C(C)C | Protease |
| CHEMBL97362 | CCC(C)[C@H](NC(=O)[C@@H](C[C@H](O)[C@H](Cc1ccccc1)NC(=O)OC(C)(C)C)Cc2ccccc2)C(=O)NCCN(C)C | Protease |
| CHEMBL70502 | CN1CCN(CC1)S(=O)(=O)Oc2cc(c(SC3=C(O)OC(C)(CCc4ccc(O)cc4)CC3=O)cc2C)C(C)(C)C | Protease |
| CHEMBL306727 | CC(C)[C@@H]1NC(=O)\C=C/CCCOc2ccc(C[C@H](NC1=O)[C@H](O)CN3CC4CCCCC4C[C@H]3C(=O)NC(C)(C)C)cc2 | Protease |
| CHEMBL263665 | CC(C)(C)NC(=O)C1CCCN1C(=O)[C@@H](O)[C@H](Cc2ccccc2)NC(=O)[C@H](CC(=O)N)NC(=O)OCc3ccccc3 | Protease |
| CHEMBL439366 | CC1(C)S[C@@H](N[C@H]1C(=O)NCCNC(=O)[C@@H]2N[C@H](SC2(C)C)[C@H](NC(=O)c3ccccc3c4ccccc4)C(=O)NCc5ccccc5)[C@H](NC(=O)c6ccccc6c7ccccc7)C(=O)NCc8ccccc8 | Protease |
| CHEMBL105880 | CC1(C)S[C@@H](N[C@H]1C(=O)NCCNC(=O)[C@@H]2N[C@H](SC2(C)C)[C@H](NC(=O)Cc3ccccn3)C(=O)NCc4ccc(Cl)cc4)[C@H](NC(=O)Cc5ccccn5)C(=O)NCc6ccc(Cl)cc6 | Protease |
| CHEMBL38269 | CC(C)C1(CCc2ccc(N)cc2)CC(=O)C(=C(O)O1)Sc3cc(C)c(N)cc3C(C)(C)C | Protease |
| CHEMBL441065 | COc1cc(ccc1N)C(=O)N[C@@H](Cc2ccccc2)[C@@H](O)C[C@H](Cc3ccccc3)NC(=O)OC(C)(C)C | Protease |
| CHEMBL112846 | CC(C)[C@@H](NC(=O)CN1CCOCC1)C(=O)N[C@@H](Cc2ccccc2)[C@H](O)[C@H](O)[C@H](Cc3ccccc3)NC(=O)[C@@H](NC(=O)CN4CCOCC4)C(C)C | Protease |
| CHEMBL322577 | CC(C)(C)NC(=O)[C@@H]1C[C@@H]2CCCC[C@@H]2CN1C[C@@H](O)C[C@@H](Cc3ccccc3)C(=O)N[C@@H]4[C@H](O)Cc5ccccc45 | Protease |
| CHEMBL355291 | CC(C)(C)NC(=O)[C@@H]1C[C@@H]2CCCC[C@@H]2CN1C[C@@H](O)C[C@@H](Cc3ccccc3)C(=O)N[C@@H]4C(O)Cc5ccccc45 | Protease |
| CHEMBL90139 | CC(C)(C)OC(=O)N[C@H](C[C@H](O)[C@H](Cc1ccccc1)NC(=O)c2ccc(O)c(N)c2)Cc3ccccc3 | Protease |
| CHEMBL36448 | CCCCCC1(CCc2ccc(O)cc2)CC(=O)C(=C(O)O1)Sc3cc(C)c(N)cc3C(C)(C)C | Protease |
| CHEMBL347500 | CC(C)(C)NC(=O)[C@@H]1CN(CC2(CC2)c3ccccc3)CCN1C[C@@H](O)C[C@@H](Cc4ccccc4)C(=O)NC5[C@H](O)Cc6ccccc56 | Protease |
| CHEMBL320150 | CC(C)(C)OC(=O)N[C@@H](Cc1ccccc1)[C@@H](O)C[C@@](C)(Cc2ccccc2)C(=O)N[C@H]3[C@H](O)Cc4ccccc34 | Protease |
| CHEMBL320321 | CC(C)[C@@H](NC(=O)N1CCN(Cc2ccccc2)CC1)C(=O)N[C@@H](Cc3ccccc3)[C@H](O)[C@H](O)[C@H](Cc4ccccc4)NC(=O)[C@@H](NC(=O)N5CCN(Cc6ccccc6)CC5)C(C)C | Protease |
| CHEMBL113825 | CC(C)(C)NC(=O)[C@@H]1CC2CCCCC2CN1C[C@@H](O)C[C@@H](Cc3ccccc3)C(=O)NC4[C@H](O)Cc5ccccc45 | Protease |
| CHEMBL273827 | CC(C)(C)NC(=O)c1ccccc1C[C@@H](O)[C@H](Cc2ccccc2)NC(=O)[C@H](C[S+]([O-])c3ccccn3)NS(=O)(=O)C | Protease |
| CHEMBL370853 | COc1cc(CN2[C@H](Cc3ccccc3)[C@H](O)CN(N(Cc4ccc(O)c(OC)c4)C2=O)C(=O)CN5CCCC5)ccc1O | Protease |
| CHEMBL318700 | CCC(C)[C@H](NC(=O)[C@@H](C[C@H](O)[C@H](Cc1ccccc1)NC(=O)OC(C)(C)C)Cc2ccccc2)C(=O)NCCCN(C)C | Protease |
| CHEMBL362524 | Nc1ccc(cc1)S(=O)(=O)N(C[C@@H](O)[C@H](Cc2ccccc2)NC(=O)O[C@H]3CO[C@H]4OCC[C@@H]34)OC5CCCCC5 | Protease |
| CHEMBL302464 | CC(C)(C)OC(=O)N[C@@H](Cc1ccccc1)C(=O)N2CCC(=O)[C@H]2Sc3ccccc3 | Protease |
| CHEMBL79362 | CC(C)[C@H](NC(=O)CCc1cccnc1)C(=O)NC(Cc2ccc(OCc3ccccc3)cc2)C(=O)C(F)(F)C(=O)NCc4ccccc4 | Protease |
| CHEMBL407052 | CC(C)(C)NC(=O)c1ccccc1C[C@@H](O)[C@H](Cc2ccccc2)NC(=O)[C@H](CC(=O)NCc3ccc4ccccc4n3)NS(=O)(=O)C | Protease |
| CHEMBL306897 | CC(C)[C@@H]1NC(=O)c2ccccc2OCCCOc3ccc(C[C@H](NC1=O)[C@H](O)CN4CC5CCCCC5C[C@H]4C(=O)NC(C)(C)C)cc3 | Protease |
| CHEMBL169490 | Cc1cc(SC2=C(O)OC(CCc3ccc(O)cc3)(CC2=O)C4CCCCC4)c(cc1OS(=O)(=O)Cn5ccnc5)C(C)(C)C | Protease |
| CHEMBL261340 | CC(C)[C@H]1[C@H](CCS1(=O)=O)OC(=O)N[C@@H](Cc2ccccc2)[C@H](O)CN3C[C@H]4CCCC[C@H]4C[C@H]3C(=O)NC(C)(C)C | Protease |
| CHEMBL147790 | Clc1ccc2[nH]c(c3nc4CCCCc4[nH]3)c(c2c1)S(=O)(=O)c5ccccc5 | Protease |
| CHEMBL1790510 | CCC(CC)NC(=O)C[C@@H](C(=O)NC[C@@H](O)[C@H](Cc1ccccc1)NC(=O)[C@@H](NC(=O)OCc2ccccc2)[C@H](C)O)C(C)(C)C | Protease |
| CHEMBL324524 | Cc1ccc(C(=O)N[C@@H](Cc2ccccc2)[C@H](O)C(=O)N3C[C@@H](Cl)C[C@H]3C(=O)NC(C)(C)C)c(C)c1O | Protease |
| CHEMBL328233 | CC(C)[C@@H]1[C@@H](CCS1(=O)=O)OC(=O)N[C@@H](Cc2ccccc2)[C@H](O)CN3CCN(C[C@H]3C(=O)NC(C)(C)C)C4CCSC4 | Protease |
| CHEMBL327194 | CCCCCCCCN1CCN(C[C@@H](O)[C@H](Cc2ccccc2)NC(=O)O[C@@H]3CCS(=O)(=O)[C@@H]3C(C)C)[C@@H](C1)C(=O)NC(C)(C)C | Protease |
| CHEMBL12900 | Cc1cccc(C)c1OCC(=O)N[C@@H](Cc2ccccc2)[C@H](O)CN3C[C@H]4CCCC[C@H]4C[C@H]3C(=O)NC(C)(C)C | Protease |
| CHEMBL323203 | COC(=O)N[C@@H](C(C)C)C(=O)N[C@@H](Cc1ccccc1)[C@H](O)CN(CC2CCCCC2)NC(=O)[C@@H](NC(=O)C)C(C)C | Protease |
| CHEMBL275123 | CC(C)[C@H](NC(=O)[C@H](Cc1cccc2ccccc12)CS(=O)(=O)C(C)(C)C)C(=O)NCP(=O)(CNC(=O)[C@@H](NC(=O)[C@H](Cc3cccc4ccccc34)CS(=O)(=O)C(C)(C)C)C(C)C)NN | Protease |
| CHEMBL163243 | CN(Cc1ccccn1)C(=O)c2ccc(C)c(c2)C(=O)N[C@@H](Cc3ccccc3)[C@H](O)CN4C[C@H]5CCCC[C@H]5C[C@H]4C(=O)NC(C)(C)C | Protease |
| CHEMBL117024 | Cc1ccc(C[C@H](NC(=O)c2ccc(C)c(O)c2C)[C@H](O)C(=O)N3C[C@@H](Cl)C[C@H]3C(=O)NC(C)(C)C)cc1 | Protease |
| CHEMBL169278 | Cc1cc(SC2=C(O)OC(CCc3ccc(O)cc3)(CC2=O)C4CCCC4)c(cc1NS(=O)(=O)c5ccccc5)C(C)(C)C | Protease |
| CHEMBL104683 | CCNC(=O)[C@@H](NC(=O)CC(C)C)[C@@H]1N[C@@H](C(=O)NCCNC(=O)[C@@H]2N[C@H](SC2(C)C)[C@H](NC(=O)CC(C)C)C(=O)NCC)C(C)(C)S1 | Protease |
| CHEMBL354803 | CC(C)C1(CCc2ccc(O)cc2)CC(=O)C(=C(O)O1)Sc3cc(C)c(NS(=O)(=O)c4cccs4)cc3C(C)(C)C | Protease |
| CHEMBL67867 | CCNS(=O)(=O)Oc1cc(c(SC2=C(O)OC(CCc3ccccc3)(CC2=O)C(C)C)cc1C)C(C)(C)C | Protease |
| CHEMBL336941 | CC[C@@H](C)[C@H](NC(=O)[C@H](C(C)C)C(O)C(O)[C@H](CC1CCCCC1)NC(=O)C(Cc2c[nH]cn2)NC(=O)CO)C(=O)NCc3ccccn3 | Protease |
| CHEMBL330915 | CC(C)[C@@H](NC(=O)N1CCN(Cc2ccccc2)CC1)C(=O)N[C@@H](Cc3ccccc3)[C@@H](O)[C@@H](O)[C@H](Cc4ccccc4)NC(=O)[C@@H](NC(=O)N5CCN(Cc6ccccc6)CC5)C(C)C | Protease |
| CHEMBL112412 | CC(C)[C@@H](NC(=O)OCc1ccccc1)C(=O)N[C@@H](Cc2ccccc2)[C@H](O)[C@H](Cc3ccccc3)NC(=O)[C@@H](NC(=O)OCCN4CCOCC4)C(C)C | Protease |
| CHEMBL292264 | CC(C)(C)c1cc2CCCc2cc1SC3=C(O)OC(CCc4ccccc4)(CC3=O)c5ccccc5 | Protease |
| CHEMBL75180 | CC(C)C[C@H](NC(=O)[C@@H](Cc1ccccc1)CP(=O)(O)[C@@H](Cc2ccccc2)NC(=O)OC(C)(C)C)C(=O)N[C@@H](Cc3ccccc3)C(=O)N | Protease |
| CHEMBL414648 | CC(C)[C@H]1SCC[C@H]1OC(=O)N[C@@H](Cc2ccccc2)[C@H](O)CN3C[C@H]4CCCC[C@H]4C[C@H]3C(=O)NC(C)(C)C | Protease |
| CHEMBL326750 | CC(C)(C)NC(=O)[C@@H]1C[C@H](Cl)CN1C(=O)[C@@H](O)[C@H](Cc2ccccc2)NC(=O)c3cccc(O)c3 | Protease |
| CHEMBL342037 | COc1ccc(cc1)S(=O)(=O)N(CC(C)C)C[C@@H](O)[C@H](Cc2ccccc2)NC(=O)c3ccc(N)cc3C | Protease |
| CHEMBL431569 | CC(C)[C@@H]1NC(=O)\C=C/CCCCOc2ccc(C[C@H](NC1=O)[C@H](O)CN3CC4CCCCC4C[C@H]3C(=O)NC(C)(C)C)cc2 | Protease |
| CHEMBL189347 | Nc1ccc(cc1)S(=O)(=O)N(C[C@@H](O)[C@H](Cc2ccccc2)NC(=O)O[C@H]3CO[C@H]4OCC[C@@H]34)OC5CCCC5 | Protease |
| CHEMBL283958 | CCC(C)C(NC(=O)[C@@H]1CCCN1CC(O)[C@@H](Cc2ccccc2)NC(=O)C(CC(=O)N)NC(=O)[C@H](CC(C)C)NC(=O)C(CO)NC(=O)C)C(=O)NC(C(C)C)C(=O)OC | Protease |
| CHEMBL94797 | CC(C)[C@H](NC(=O)C)C(=O)N[C@@H](Cc1ccccc1)[C@@H](O)CN(CC2CCCCC2)NC(=O)[C@@H](NC(=O)C)C(C)C | Protease |
| CHEMBL169037 | CC(C)C1(CCc2ccccc2)CC(=O)C(=C(O)O1)Sc3cc(C)c(NS(=O)(=O)c4ccc(cc4)C#N)cc3C(C)(C)C | Protease |
| CHEMBL355316 | CC(C)(C)NC(=O)[C@@H]1C[C@@H]2CCCC[C@@H]2CN1C[C@@H](O)[C@H](Cc3ccccc3)NC(=O)[C@@H]4CCCS(=O)(=O)C4 | Protease |
| CHEMBL147210 | CCCc1[nH]c(nc1C)c2[nH]c3ccc(Cl)cc3c2S(=O)(=O)c4ccccc4 | Protease |
| CHEMBL155588 | CC(C)c1nc(CN(C2CC2)C(=O)NC(C)C(=O)N[C@@H](Cc3ccccc3)[C@@H](O)C[C@H](Cc4ccccc4)NC(=O)OCc5cncs5)cs1 | Protease |
| CHEMBL421019 | CC(C)(C)OC(=O)N[C@@H](Cc1ccccc1)[C@@H](O)C[C@@H](Cc2ccccc2)C(=O)N[C@@H]3C=CC[C@H]3O | Protease |
| CHEMBL444057 | CC(C)(C)OC(=O)N[C@@H](Cc1ccccc1)[C@@H](O)C[C@@H](Cc2ccccc2)C(=O)N[C@H]3C=CC[C@H]3O | Protease |
| CHEMBL138336 | CC(C)(C)OC(=O)N[C@@H](Cc1ccccc1)[C@@H](O)C[C@@H](Cc2ccccc2)C(=O)NC3C=CC[C@@H]3O | Protease |
| CHEMBL167022 | CC(C)(C)OC(=O)N[C@@H](Cc1ccccc1)[C@@H](O)C[C@@H](Cc2ccccc2)C(=O)NC3C=CC[C@H]3O | Protease |
| CHEMBL37755 | Cc1cc(SC2=C(O)OC(C)(CCc3ccc(O)cc3)CC2=O)c(cc1N)C(C)(C)C | Protease |
| CHEMBL354216 | CC(C)C1(CCc2ccc(O)cc2)CC(=O)C(=C(O)O1)Sc3cc(C)c(OS(=O)(=O)c4ccc(F)cc4)cc3C(C)(C)C | Protease |
| CHEMBL103783 | CC(C)[C@H](NC(=O)CC1NC(=O)NC1=O)C(=O)N[C@@H](Cc2ccccc2)[C@@H](O)[C@H](O)[C@H](Cc3ccccc3)NC(=O)[C@@H](NC(=O)CC4NC(=O)NC4=O)C(C)C | Protease |
| CHEMBL90699 | CC(C)(C)OC(=O)N[C@H](C[C@H](O)[C@H](Cc1ccccc1)NC(=O)c2ccc(N)c(N)c2)Cc3ccccc3 | Protease |
| CHEMBL71662 | CN1CCN(CC1)S(=O)(=O)Oc2cc(c(SC3=C(O)OC(CCc4ccc(O)cc4)(CC3=O)C5CCCCC5)cc2C)C(C)(C)C | Protease |
| CHEMBL354173 | Cc1cc(SC2=C(O)OC(C)(CCc3ccc(O)cc3)CC2=O)c(cc1NS(=O)(=O)C)C(C)(C)C | Protease |
| CHEMBL172622 | Cc1cc(SC2=C(O)OC(C)(CCc3ccc(O)cc3)CC2=O)c(cc1OS(=O)(=O)c4ccccc4)C(C)(C)C | Protease |
| CHEMBL107748 | CCNC(=O)[C@@H](NC(=O)c1ccccc1c2ccccc2)[C@@H]3N[C@@H](C(=O)NCCNC(=O)[C@@H]4N[C@H](SC4(C)C)[C@H](NC(=O)c5ccccc5c6ccccc6)C(=O)NCC)C(C)(C)S3 | Protease |
| CHEMBL323318 | CCNC(=O)[C@@H](NC(=O)c1ccccc1c2ccccc2)[C@@H]3N[C@@H](C(=O)NCCNC(=O)C4N[C@H](SC4(C)C)[C@H](NC(=O)c5ccccc5c6ccccc6)C(=O)NCC)C(C)(C)S3 | Protease |
| CHEMBL290900 | COC(=O)[C@H](Cc1ccccc1)NC(=O)[C@@H](NC(=O)[C@@H](CC(O)C[C@@H](Cc2ccccc2)C(=O)N[C@@H](C(C)C)C(=O)N[C@@H](Cc3ccccc3)C(=O)OC)Cc4ccccc4)C(C)C | Protease |
| CHEMBL317204 | CC(C)CN(C[C@H](O)[C@H](Cc1ccccc1)NC(=O)[C@@H](NC(=O)OCc2ccccc2)C(C)C)NC(=O)[C@@H](NC(=O)OCc3ccccc3)C(C)C | Protease |
| CHEMBL88255 | CC(C)[C@@H](NC(=O)C)C(=O)N[C@H](C(C)C)C(=O)N[C@@H](Cc1ccccc1)C(O)[C@H](Cc2ccccc2)NC(=O)[C@@H](NC(=O)[C@@H](NC(=O)C)C(C)C)C(C)C | Protease |
| CHEMBL70787 | CC(C)(C)NC(=O)[C@@H]1CC2CCCCC2CN1C[C@@H](O)[C@@H]3Cc4ccc(OCCCOc5cc6ccccc6cc5C(=O)N[C@@H](CC(=O)N)C(=O)N3)cc4 | Protease |
| CHEMBL99878 | CC(C)(C)NC(=O)[C@@H]1C[C@@H]2SCC[C@@H]2CN1C[C@@H](O)[C@H](CSc3ccccc3)NC(=O)[C@@H](CS(=O)(=O)c4ccc(F)cc4)NC(=O)C(F)(F)F | Protease |
| CHEMBL277801 | C[C@@H]1[C@@H](CCS1(=O)=O)OC(=O)N[C@@H](Cc2ccccc2)[C@H](O)CN3C[C@H]4CCCC[C@H]4C[C@H]3C(=O)NC(C)(C)C | Protease |
| CHEMBL354502 | OCc1ccc(CN2[C@H](COc3ccccc3)[C@H](O)[C@@H](O)[C@@H](COc4ccccc4)N(Cc5ccc(CO)cc5)C2=O)cc1 | Protease |
| CHEMBL99412 | CC(C)C[C@H](NC(=O)[C@@H](C[C@H](O)[C@H](Cc1ccccc1)NC(=O)OC(C)(C)C)Cc2ccccc2)C(=O)N | Protease |
| CHEMBL70140 | CC(C)C[C@]1(N=CC(=C1O)[C@@H](Cc2ccccc2)C(=O)N)C3=C(O)[C@@](C[C@H](O)[C@H](Cc4ccccc4)NC(=O)OC(C)(C)C)(Cc5ccccc5)N=C3 | Protease |
| CHEMBL114192 | CC(C)[C@@H](NC(=O)NCc1ccccn1)C(=O)N[C@@H](Cc2ccccc2)C(O)[C@H](Cc3ccccc3)NC(=O)[C@@H](NC(=O)NCc4ccccn4)C(C)C | Protease |
| CHEMBL344856 | COc1ccc(cc1)S(=O)(=O)N(CC(C)C)C[C@@H](O)[C@H](Cc2ccccc2)NC(=O)c3cc(N)ccc3C | Protease |
| CHEMBL418511 | Cc1ccc(c(SC2=C(O)OC(CCc3ccccc3)(CC2=O)c4ccccc4)c1)C(C)(C)C | Protease |
| CHEMBL1790657 | CC[C@H](C)[C@H](NC(=O)[C@H](C)N(CC=C)C(=O)[C@H](Cc1ccccc1)NC(=O)OC(C)(C)C)C(=O)N[C@@H](C(C)C)C(=O)OC | Protease |
| CHEMBL343108 | CCc1ccccc1C(=O)N[C@@H](Cc2ccccc2)[C@H](O)CN(CC(C)C)S(=O)(=O)c3ccc(OC)cc3 | Protease |
| CHEMBL121995 | CCC(C)c1ccccc1SC2=C(O)OC(CCc3ccccc3)(CC2=O)c4ccccc4 | Protease |
| CHEMBL341883 | CCc1nc([nH]c1CC)c2[nH]c3ccc(Cl)cc3c2S(=O)(=O)c4ccccc4 | Protease |
| CHEMBL347671 | CC(C)CCN(C[C@@H](O)[C@H](Cc1ccccc1)NC(=O)OCc2ccccc2)S(=O)(=O)c3ccc(Cl)cc3 | Protease |
| CHEMBL71961 | C[C@@H](N[C@@H](C(C)C)C(=O)N[C@@H](Cc1ccccc1)[C@@H](O)C(=O)N[C@H]2Cc3ccc(OCCCNC(=O)[C@@H](NC2=O)C(C)C)cc3)NC(=O)OCc4ccccc4 | Protease |
| CHEMBL431534 | CC(C)C[C@]1(N=CC(=C1O)[C@@H](Cc2ccccc2)C(=O)N)C3=C(O)[C@@](C[C@H](O)[C@@H](CC(=O)OC(C)(C)C)Cc4ccccc4)(Cc5ccccc5)N=C3 | Protease |
| CHEMBL278375 | COC(=O)[C@H](NC(=O)C(F)(F)C(=O)C(Cc1ccc(OCc2ccccc2)cc1)NC(=O)[C@@H](NC(=O)OCc3ccccc3)C(C)C)C(C)C | Protease |
| CHEMBL88964 | CCCC1(CCc2ccccc2)OC(=C(C(CC)c3cccc(NS(=O)(=O)c4ccc(cn4)C(F)(F)F)c3)C(=O)O1)O | Protease |
| CHEMBL309837 | CC(C)[C@@H](NC(=O)C[C@@H]1O[C@@H]1[C@H](Cc2ccccc2)NC(=O)[C@H](CC(=O)N)NC(=O)c3ccc4ccccc4n3)c5ccccc5 | Protease |
| CHEMBL288163 | CC(C)[C@H](NC(=O)[C@@H](CC(O)C[C@@H](Cc1ccccc1)C(=O)N[C@@H](C(C)C)C(=O)NCc2nc3ccccc3[nH]2)Cc4ccccc4)C(=O)NCc5nc6ccccc6[nH]5 | Protease |
| CHEMBL302958 | CC(C)[C@H](NC(=O)[C@H](Cc1c[nH]c2ccccc12)NC(=O)C)C(=O)N[C@@H](Cc3ccccc3)[C@@H](O)C(=O)N[C@H]4Cc5ccc(OCCCNC(=O)[C@@H](NC4=O)C(C)C)cc5 | Protease |
| CHEMBL430863 | CC(C)CN(C[C@@H](O)[C@H](Cc1ccccc1)NC(=O)[C@@H](CS(=O)(=O)c2ccc(F)cc2)NS(=O)(=O)C)C(=O)NC(C)(C)C | Protease |
| CHEMBL289402 | Cc1ccc(c(SC2=C(O)O[C@@](CCc3ccccc3)(CC2=O)c4ccccc4)c1)C(C)(C)C | Protease |
| CHEMBL19886 | COCCOCO[C@H](NC(=O)C(F)(F)C(=O)C(Cc1ccc(OCc2ccccc2)cc1)NC(=O)[C@@H](NC(=O)OCc3ccccc3)C(C)C)C(C)C | Protease |
| CHEMBL283726 | CO[C@H](NC(=O)C(F)(F)C(=O)C(Cc1ccc(OCc2ccccc2)cc1)NC(=O)[C@@H](NC(=O)OCc3ccccc3)C(C)C)C(C)C | Protease |
| CHEMBL3143979 | CC(C)[C@H](NC(=O)C)C(=O)N[C@@H](C(C)C)C(=O)N[C@@H](Cc1ccccc1)C(O)[C@H](Cc2ccccc2)NC(=O)[C@@H](NC(=O)[C@@H](NC(=O)C)C(C)C)C(C)C | Protease |
| CHEMBL79128 | CC(C)[C@H](Cc1ccccc1)NC(=O)C[C@@H]2O[C@@H]2[C@H](Cc3ccccc3)NC(=O)[C@H](CC(=O)N)NC(=O)c4ccc5ccccc5n4 | Protease |
| CHEMBL78312 | CONC(=O)[C@H](NC(=O)C[C@@H]1O[C@@H]1[C@H](Cc2ccccc2)NC(=O)[C@H](CC(=O)N)NC(=O)c3ccc4ccccc4n3)C(C)C | Protease |
| CHEMBL353812 | CC(C)C1(CCc2ccc(O)cc2)CC(=O)C(=C(O)O1)Sc3cc(C)c(OS(=O)(=O)c4ccccc4)cc3C(C)(C)C | Protease |
| CHEMBL191849 | COc1cc(ccc1N)C(=O)N[C@H](C[C@H](O)[C@H](Cc2ccccc2)NC(=O)OCc3ccccc3)Cc4ccccc4 | Protease |
| CHEMBL105879 | CC1(C)S[C@@H](N[C@H]1C(=O)NCCNC(=O)[C@@H]2N[C@H](SC2(C)C)[C@H](NC(=O)Cc3ccccc3)C(=O)NCc4ccccn4)[C@H](NC(=O)Cc5ccccc5)C(=O)NCc6ccccn6 | Protease |
| CHEMBL35088 | CC(C)C1(CCc2ccc(O)cc2)CC(=O)C(=C(O)O1)Sc3cc(C)c(cc3C(C)(C)C)N(C)C | Protease |
| CHEMBL362510 | Nc1cccc(c1)S(=O)(=O)N(C[C@@H](O)[C@H](Cc2ccccc2)NC(=O)O[C@H]3CO[C@H]4OCC[C@@H]34)OC5CCCCC5 | Protease |
| CHEMBL323653 | CC(C)(C)NC(=O)[C@@H]1C[C@H](Cl)CN1C(=O)[C@@H](O)[C@H](Cc2ccccc2)NC(=O)c3ccccc3Cl | Protease |
| CHEMBL150003 | CCCNC(=O)[C@@H](NC(=O)Cc1ccccc1)[C@@H]2N[C@@H](C(=O)N(C)CCN(C)C(=O)[C@@H]3N[C@H](SC3(C)C)[C@H](NC(=O)Cc4ccccc4)C(=O)NCC)C(C)(C)S2 | Protease |
| CHEMBL37813 | Cc1cc(SC2=C(O)OC(CCc3ccccc3)(CC2=O)c4ccccc4)c(cc1N)C(C)(C)C | Protease |
| CHEMBL121758 | Cc1ccc(c(SC2=C(O)OC(CCc3ccc(O)cc3)(CC2=O)c4ccccc4)c1)C(C)(C)C | Protease |
| CHEMBL293860 | CC(C)(C)c1c(oc2ccccc12)SC3=C(O)OC(CCc4ccccc4)(CC3=O)c5ccccc5 | Protease |
| CHEMBL91443 | CC(C)(C)OC(=O)N[C@H](C[C@H](O)[C@H](Cc1ccccc1)NC(=O)c2ccc(N)c(O)c2)Cc3ccccc3 | Protease |
| CHEMBL1790513 | CC[C@H](C)[C@H](NC(=O)[C@H](C(C)C)[C@@H](O)[C@H](O)[C@H](CC1CCCCC1)NC(=O)c2ccccc2OCSc3ccccc3)C(=O)NCc4nc5ccccc5[nH]4 | Protease |
| CHEMBL336336 | CC[C@@H](C)[C@H](NC(=O)[C@H](C(C)C)C(O)C(O)C(Cc1c[nH]cn1)NC(=O)COc2ccccc2)C(=O)NCc3ccccn3 | Protease |
| CHEMBL130039 | CC[C@H](C)[C@H](NC(=O)C(C(C)C)C(O)C(O)[C@H](CC(C)C)NC(=O)C(Cc1c[nH]cn1)NC(=O)COc2cccc3ccccc23)C(=O)NCc4ccccn4 | Protease |
| CHEMBL1790965 | CC[C@@H](C)[C@H](NC(=O)[C@H](CC(C)C)C(O)C(O)[C@H](CC1CCCCC1)NC(=O)C(Cc2c[nH]cn2)NC(=O)COc3cccc4ccccc34)C(=O)NCc5ccccn5 | Protease |
| CHEMBL279793 | CC(C)(C)NC(=O)[C@@H]1C[C@@H]2CCC[C@@H]2CN1C[C@@H](O)[C@H](Cc3ccccc3)NC(=O)O[C@H]4CCS(=O)(=O)C4 | Protease |
| CHEMBL18068 | C[C@H]1[C@H](CCS1(=O)=O)OC(=O)N[C@@H](Cc2ccccc2)[C@H](O)CN3C[C@H]4CCCC[C@H]4C[C@H]3C(=O)NC(C)(C)C | Protease |
| CHEMBL1907857 | CC(C)[C@@]1(CCc2ccsc2)CC(=O)C(=C(O)O1)Sc3cc(C)c(CO)cc3C(C)(C)C | Protease |
| CHEMBL291974 | CC(C)(C)OC(=O)N[C@@H](Cc1ccccc1)[C@@H](O)C[C@]2(Cc3ccccc3)N=CC(=C2O)C4[C@H](O)Cc5ccccc45 | Protease |
| CHEMBL335916 | CSCC(NC(=O)c1ccc2ccccc2n1)C(=O)N[C@@H](Cc3ccccc3)[C@@H](O)CN4CCCCC4C(=O)NC(C)(C)C | Protease |
| CHEMBL249788 | CC(C)CC(NC(=O)[C@@H](C[C@H](O)[C@H](Cc1ccccc1)NC(=O)OC(C)(C)C)Cc2ccccc2)C(=O)NCCc3ccccc3 | Protease |
| CHEMBL331829 | CC(C)[C@@H](NC(=O)CCc1ccccn1)C(=O)N[C@@H](Cc2ccccc2)C(O)[C@H](Cc3ccccc3)NC(=O)[C@@H](NC(=O)CCc4ccccn4)C(C)C | Protease |
| CHEMBL91601 | CC(C)(C)OC(=O)N[C@H](C[C@H](O)[C@H](Cc1ccccc1)NC(=O)c2cc(O)ccc2Cl)Cc3ccccc3 | Protease |
| CHEMBL325944 | CC(C)(C)NC(=O)[C@@H]1C[C@H](Cl)CN1C(=O)[C@@H](O)[C@H](Cc2ccccc2)NC(=O)c3ccccc3Br | Protease |
| CHEMBL189244 | O[C@H](CN(OC1CCCC1)S(=O)(=O)c2ccc(O)cc2)[C@H](Cc3ccccc3)NC(=O)O[C@H]4CO[C@H]5OCC[C@@H]45 | Protease |
| CHEMBL321514 | CC(C)[C@H](NC(=O)Cc1csc(N)n1)C(=O)N[C@@H](Cc2ccccc2)[C@@H](O)[C@H](O)[C@H](Cc3ccccc3)NC(=O)[C@@H](NC(=O)Cc4csc(N)n4)C(C)C | Protease |
| CHEMBL119488 | Cc1ccc(c(SC2=C(O)OC(CCc3ccccc3)(CC2=O)c4ccc(OCCO)cc4)c1)C(C)(C)C | Protease |
| CHEMBL331724 | Cc1ccc(c(SC2=C(O)OC(CC2=O)(c3ccccc3)c4ccccc4)c1)C(C)(C)C | Protease |
| CHEMBL2311105 | CC(C)[C@H](NC(=O)C)C(=O)N[C@@H](Cc1ccccc1)C(O)[C@H](Cc2ccccc2)NC(=O)[C@@H](NC(=O)C)C(C)C | Protease |
| CHEMBL423106 | CC(C)CC(NC(=O)[C@@H](C[C@H](O)[C@H](Cc1ccccc1)NC(=O)OC(C)(C)C)Cc2ccccc2)C(=O)N | Protease |
| CHEMBL276431 | Cc1c(O)cccc1C(=O)N[C@@H](Cc2ccccc2)[C@H](O)CN3CC[C@H](C[C@H]3C(=O)NC(C)(C)C)OCc4ccncc4 | Protease |
| CHEMBL2374441 | COC(=O)N[C@H](Cc1ccccc1)C(=O)NCCCC[C@@H](CO)N(CC(C)C)S(=O)(=O)c2ccc(N)cc2 | Protease |
| CHEMBL353815 | CC(C)C[C@H]1[C@H](CCS1(=O)=O)OC(=O)N[C@@H](Cc2ccccc2)[C@H](O)CN3C[C@H]4CCCC[C@H]4C[C@H]3C(=O)NC(C)(C)C | Protease |
| CHEMBL275554 | CC(C)(C)OC(=O)N[C@@H](Cc1ccccc1)[C@@H](O)[C@@H](O)[C@H](Cc2ccccc2)NC(=O)OC(C)(C)C | Protease |
| CHEMBL17853 | CC(C)(C)NC(=O)[C@@H]1C[C@@H]2CCCC[C@@H]2CN1C[C@@H](O)[C@H](Cc3ccccc3)NC(=O)C4CO[C@H]5CCS(=O)(=O)[C@H]5C4 | Protease |
| CHEMBL36600 | CC(C)C1(CCc2ccc(O)cc2)CC(=O)C(=C(O)O1)Sc3cc(C)c(NC(=O)c4ccc(cc4)C(F)(F)F)cc3C(C)(C)C | Protease |
| CHEMBL263268 | Cc1cc(SC2=C(O)OC(C)(CCc3ccc(O)cc3)CC2=O)c(cc1NS(=O)(=O)c4ccc(cc4)C(F)(F)F)C(C)(C)C | Protease |
| CHEMBL276077 | C[C@H]1[C@H](CCS1(=O)=O)OC(=O)N[C@@H](Cc2ccccc2)[C@H](O)CN3C[C@H]4CCC[C@H]4C[C@H]3C(=O)NC(C)(C)C | Protease |
| CHEMBL99607 | Cc1c(O)cccc1C(=O)N[C@@H](Cc2ccccc2)[C@H](O)CN3C[C@H]4CCCC[C@H]4C[C@H]3C(=O)NC(C)(C)C | Protease |
| CHEMBL431754 | CC(C)(C)NC(=O)[C@@H]1CC2CCCCC2CN1C[C@@H](O)[C@@H]3Cc4ccc(OCCOc5cc6ccccc6cc5C(=O)N[C@@H](CC(=O)N)C(=O)N3)cc4 | Protease |
| CHEMBL90449 | CCCC1(CCc2ccccc2)OC(=C(C(CC)c3cccc(NS(=O)(=O)c4ccccn4)c3)C(=O)O1)O | Protease |
| CHEMBL312909 | CCCC1(CCc2ccccc2)OC(=C(C(CC)c3cccc(NS(=O)(=O)c4ccc(N)cn4)c3)C(=O)O1)O | Protease |
| CHEMBL113264 | CC(C)[C@@H](NC(=O)OCc1ccccc1)C(=O)N[C@@H](Cc2ccccc2)[C@H](O)[C@H](Cc3ccccc3)NC(=O)[C@@H](NC(=O)OCC4CCCN(C)C4)C(C)C | Protease |
| CHEMBL72129 | CC(C)[C@H](NC(=O)N[C@@H](C)S(=O)(=O)c1ccc(C)cc1)C(=O)N[C@@H](Cc2ccccc2)[C@@H](O)C(=O)N[C@H]3Cc4ccc(OCCCNC(=O)[C@@H](NC3=O)C(C)C)cc4 | Protease |
| CHEMBL100039 | CC(C)CCN(C[C@@H](O)[C@H](Cc1ccccc1)NC(=O)[C@H](CC(=O)N)NC(=O)OCc2ccccc2)C(=O)NC(C)(C)C | Protease |
| CHEMBL208651 | COC(=O)N[C@H](C(c1ccccc1)c2ccccc2)C(=O)NCCCC[C@@H](CO)N(CC(C)C)S(=O)(=O)c3ccc(N)cc3 | Protease |
| CHEMBL321377 | CC[C@H](C)[C@H](NC(=O)[C@@H]1CCCN1C[C@H](O)[C@H](Cc2ccccc2)NC(=O)[C@H](CC(=O)N)NC(=O)OCc3ccccc3)C(=O)N[C@@H](C(C)C)C(=O)OC | Protease |
| CHEMBL307123 | CC(C)CCN(CC(O)[C@H](Cc1ccccc1)NC(=O)[C@H](CC(=O)N)NC(=O)OCc2ccccc2)C(=O)NC(C)(C)C | Protease |
| CHEMBL99725 | CC(C)[C@H](NC(=O)c1sc(C)nc1C)C(=O)N[C@H](C[C@H](O)[C@H](Cc2ccccc2)NC(=O)[C@@H](NC(=O)c3sc(C)nc3C)C(C)C)Cc4ccccc4 | Protease |
| CHEMBL20135 | CC(C)[C@H](CCc1ccccc1)NC(=O)C(F)(F)C(=O)C(Cc2ccc(OCc3ccccc3)cc2)NC(=O)[C@@H](NC(=O)OCc4ccccc4)C(C)C | Protease |
| CHEMBL24870 | CCC(C)[C@H](NC(=O)[C@H]1CCCN1CC(O)[C@H](Cc2ccccc2)NC(=O)[C@@H](CC(=O)N)NC(=O)OCc3ccccc3)C(=O)N[C@@H](C(C)C)C(=O)OC | Protease |
| CHEMBL430995 | CC(C)[C@@H]1NC(=O)[C@H](Cc2ccc(OCCCNC1=O)cc2)NC(=O)C(=O)C(Cc3ccccc3)NC(=O)OC(C)(C)C | Protease |
| CHEMBL335379 | CC(C)CCNC(=O)N(C[C@@H](O)[C@H](Cc1ccccc1)NC(=O)[C@H](CC(=O)N)NC(=O)OCc2ccccc2)C(C)(C)C | Protease |
| CHEMBL364424 | O[C@H](CN(OC1CCCCC1)S(=O)(=O)c2ccc3OCOc3c2)[C@H](Cc4ccccc4)NC(=O)O[C@H]5CO[C@H]6OCC[C@@H]56 | Protease |
| CHEMBL111958 | CC[C@H](C)[C@H](NC(=O)[C@@H]1CCCN1C[C@@H](O)[C@H](Cc2ccccc2)NC(=O)[C@H](CC(=O)N)NC(=O)OCc3ccccc3)C(=O)N[C@@H](C(C)C)C(=O)OC | Protease |
| CHEMBL77685 | CC(C)C1(CCc2csc(C)n2)CC(=O)C(=C(O)O1)Sc3cc(C)c(CO)cc3C(C)(C)C | Protease |
| CHEMBL110865 | CC(C)[C@@H](NS(=O)(=O)CCc1ccncc1)C(=O)N[C@@H](Cc2ccccc2)[C@@H](O)[C@@H](O)[C@H](Cc3ccccc3)NC(=O)[C@@H](NS(=O)(=O)CCc4ccncc4)C(C)C | Protease |
| CHEMBL279326 | CC[C@@H]1[C@@H](CCS1(=O)=O)OC(=O)N[C@@H](Cc2ccccc2)[C@H](O)CN3C[C@H]4CCCC[C@H]4C[C@H]3C(=O)NC(C)(C)C | Protease |
| CHEMBL35664 | CC(C)C1(CCc2ccc(O)cc2)CC(=O)C(=C(O)O1)Sc3cc(C)c(NC(=O)c4ccccc4)cc3C(C)(C)C | Protease |
| CHEMBL93162 | CC(C)[C@@H]1[C@@H](CCS1(=O)=O)OC(=O)N[C@@H](Cc2ccccc2)[C@H](O)CN3CCN(C[C@H]3C(=O)NC(C)(C)C)C4CCCCC4 | Protease |
| CHEMBL287118 | CC(C)CC1(CCc2ccc(O)cc2)CC(=O)C(=C(O)O1)Sc3cc(C)c(N)cc3C(C)(C)C | Protease |
| CHEMBL91085 | CCCN1CCN(C[C@@H](O)[C@H](Cc2ccccc2)NC(=O)O[C@@H]3CCS(=O)(=O)[C@@H]3C(C)C)[C@@H](C1)C(=O)NC(C)(C)C | Protease |
| CHEMBL350200 | Cc1ccc(cc1C(=O)N[C@@H](Cc2ccccc2)[C@H](O)CN3C[C@H]4CCCC[C@H]4C[C@H]3C(=O)NC(C)(C)C)C(=O)N5CCC6C=CC=CC6C5 | Protease |
| CHEMBL82490 | CC(C)(N1CCN(C[C@@H](O)C[C@@H](Cc2ccccc2)C(=O)N[C@@H]3[C@H](O)COc4ccccc34)[C@@H](C1)C(=O)Nc5ccccc5)c6oc7ccncc7c6 | Protease |
| CHEMBL275546 | CC(C)c1ccccc1SC2=C(O)C=C(OC2=O)c3cccc(C)c3 | Protease |
| CHEMBL141046 | COc1ccc(cc1)S(=O)(=O)N(CC(C)C)C[C@@H](O)[C@H](Cc2ccccc2)NC(=O)c3ccccc3 | Protease |
| CHEMBL269252 | CC1(C)S[C@@H](N[C@H]1C(=O)NCCNC(=O)[C@@H]2N[C@H](SC2(C)C)[C@H](NC(=O)Cc3ccccn3)C(=O)NCc4ccc(Cl)cc4Cl)[C@H](NC(=O)Cc5ccccn5)C(=O)NCc6ccc(Cl)cc6Cl | Protease |
| CHEMBL307001 | CC(C)(C)[C@@H](CC(=O)Nc1ccccc1)C(=O)NC[C@@H](O)[C@H](Cc2ccccc2)NC(=O)OCc3ccccc3 | Protease |
| CHEMBL159378 | CC(C)(C)NC(=O)c1ccccc1C[C@@H](O)[C@H](CSc2ccccc2)NC(=O)[C@H](CC(=O)N)NC(=O)OCc3ccccc3 | Protease |
| CHEMBL124789 | CC(C)c1ccccc1SC2=C(O)OC(CCc3ccccc3)(CC2=O)c4ccccc4 | Protease |
| CHEMBL436694 | CC1(C)S[C@@H](N[C@H]1C(=O)NC[C@@H](O)[C@@H](O)CNC(=O)[C@@H]2N[C@H](SC2(C)C)[C@H](NC(=O)Cc3ccccc3)C(=O)NCc4ccccc4)[C@H](NC(=O)Cc5ccccc5)C(=O)NCc6ccccc6 | Protease |
| CHEMBL308167 | CC(C)C1(CCc2ccsc2CO)CC(=O)C(=C(O)O1)Sc3cc(C)c(CO)cc3C(C)(C)C | Protease |
| CHEMBL78185 | CC(C)c1csc(CCC2(CC(=O)C(=C(O)O2)Sc3cc(C)c(CO)cc3C(C)(C)C)C(C)C)n1 | Protease |
| CHEMBL73308 | O[C@@H](CC[C@H](Cc1ccccc1)NC(=O)c2ccccc2NC(=O)OCc3ccccn3)[C@H](Cc4ccccc4)NC(=O)c5ccccc5NC(=O)OCc6ccccn6 | Protease |
| CHEMBL334770 | CC(C)C[C@H](NC(=O)C(CO)NC(=O)C)C(=O)N[C@@H](CC(=O)N)C(=O)N[C@@H](Cc1ccccc1)[C@@H](O)CN2CCCC2C(=O)OC(C)(C)C | Protease |
| CHEMBL284341 | CC(C)C1(CCc2ccccc2)CC(=O)C(=C(O)O1)Sc3cc(C)c(NC(=O)c4ccc(cc4)C#N)cc3C(C)(C)C | Protease |
| CHEMBL341237 | OC1=C(Sc2ccccc2C3CCCC3)C(=O)CC(O1)(c4ccccc4)c5ccccc5 | Protease |
| CHEMBL354499 | CC(C)C1(CCc2ccccc2)CC(=O)C(=C(O)O1)Sc3cc(C)c(OS(=O)(=O)c4cccnc4)cc3C(C)(C)C | Protease |
| CHEMBL348282 | O[C@H](CN(CC1CCCCC1)S(=O)(=O)c2ccccc2)[C@H](Cc3ccccc3)NC(=O)OCc4ccccc4 | Protease |
| CHEMBL333833 | CC(C)(C)NC(=O)[C@@H]1C[C@H](Cl)CN1C(=O)[C@@H](O)[C@H](Cc2ccccc2)NC(=O)c3ccccc3I | Protease |
| CHEMBL19998 | CC(C)C[C@H](NC(=O)C(CO)NC(=O)C)C(=O)NC(CC(=O)N)C(=O)N[C@H](Cc1ccccc1)[C@@H](O)CN2CCC[C@H]2C(=O)OC(C)(C)C | Protease |
| CHEMBL131802 | CC(C)C[C@H](NC(=O)[C@H](CO)NC(=O)C)C(=O)N[C@@H](CC(=O)N)C(=O)N[C@@H](Cc1ccccc1)[C@H](O)CN2CCC[C@H]2C(=O)OC(C)(C)C | Protease |
| CHEMBL357706 | CC(C)c1nc(CN(C2CC2)C(=O)NCCC(=O)N[C@H](C[C@H](O)[C@H](Cc3ccccc3)NC(=O)OCc4cncs4)Cc5ccccc5)cs1 | Protease |
| CHEMBL277464 | CC(C)C[C@H](NC(=O)C(CO)NC(=O)C)C(=O)NC(CC(=O)N)C(=O)N[C@H](Cc1ccccc1)[C@H](O)CN2CCC[C@H]2C(=O)OC(C)(C)C | Protease |
| CHEMBL146361 | C[C@@H]1CC[C@@H](O)[C@H]1N2CC[C@](C[C@H](O)[C@H](Cc3ccccc3)NC(=O)OC(C)(C)C)(Cc4ccccc4)C2=O | Protease |
| CHEMBL286759 | NC(=O)c1[nH]c2ccc(Cl)cc2c1Sc3ccccc3 | Protease |
| CHEMBL112569 | CC(C)[C@@H](NC(=O)OCc1ccccc1)C(=O)N[C@@H](Cc2ccccc2)[C@H](O)[C@H](Cc3ccccc3)NC(=O)[C@@H](NC(=O)OCCN4CCCC4)C(C)C | Protease |
| CHEMBL121855 | CC(C)c1ccc(C(C)C)c(SC2=C(O)OC(CCc3ccccc3)(CC2=O)c4ccccc4)c1 | Protease |
| CHEMBL276203 | CC(=O)N[C@@H](CSc1ccc2ccccc2n1)C(=O)N[C@@H](Cc3ccccc3)[C@H](O)Cc4ccccc4C(=O)NC(C)(C)C | Protease |
| CHEMBL430153 | CC(C)(C)NC(=O)c1ccccc1C[C@@H](O)[C@H](Cc2ccccc2)NC(=O)[C@H](CSCc3ccccc3)NS(=O)(=O)C | Protease |
| CHEMBL17621 | CCCC[C@H]1[C@H](CCS1(=O)=O)OC(=O)N[C@@H](Cc2ccccc2)[C@H](O)CN3C[C@H]4CCCC[C@H]4C[C@H]3C(=O)NC(C)(C)C | Protease |
| CHEMBL69134 | CCC(CC)NC(=O)C[C@@H](C(=O)NC[C@@H](O)[C@H](Cc1ccccc1)NC(=O)OCc2ccccc2)C(C)(C)C | Protease |
| CHEMBL34008 | CC(C)C1(CCc2ccc(O)cc2)CC(=O)C(=C(O)O1)Sc3cc(C)c(NC(=O)c4cccc(c4)C#N)cc3C(C)(C)C | Protease |
| CHEMBL50382 | CC(C)(C)OC(=O)N[C@@H](Cc1ccccc1)[C@H](O)CNC[C@@H](O)[C@H](Cc2ccccc2)NC(=O)C3(O)CCCC3(C)C | Protease |
| CHEMBL21145 | OCc1ccc(CN2[C@H](Cc3ccccc3)[C@H](O)[C@@H](O)[C@@H](Cc4ccccc4)N(Cc5ccc(CO)cc5)C2=O)cc1 | Protease |
| CHEMBL97558 | CC(C)C[C@H](NC(=O)[C@@H](C[C@H](O)[C@H](Cc1ccccc1)NC(=O)OC(C)(C)C)Cc2ccccc2)C(=O)NCCc3ccccc3 | Protease |
| CHEMBL276671 | CC(C)c1ccccc1SC2=C(O)C=C(OC2=O)c3cc(C)cc(C)c3 | Protease |
| CHEMBL105459 | CNC(=O)[C@@H](NC(=O)[C@H](OCc1ccccc1)[C@H](O)[C@@H](O)[C@@H](OCc2ccccc2)C(=O)N[C@@H](C(C)C)C(=O)NC)C(C)C | Protease |
| CHEMBL75833 | CC(C)[C@@H]1[C@@H](CCS1(=O)=O)OC(=O)N[C@@H](Cc2ccccc2)[C@H](O)CN3CCN(Cc4cccnc4)C[C@H]3C(=O)NC(C)(C)C | Protease |
| CHEMBL79345 | CCN(Cc1ccccc1)C(=O)C(F)(F)C(=O)C(Cc2ccc(OCc3ccccc3)cc2)NC(=O)[C@@H](NC(=O)OCc4ccccc4)C(C)C | Protease |
| CHEMBL427631 | CC1(C)S[C@@H](N[C@H]1C(=O)NC[C@@H](O)[C@H](O)CNC(=O)[C@@H]2N[C@H](SC2(C)C)[C@H](NC(=O)Cc3ccccc3)C(=O)NCc4ccccc4)[C@H](NC(=O)Cc5ccccc5)C(=O)NCc6ccccc6 | Protease |
| CHEMBL107393 | CCNC(=O)[C@@H](NC(=O)Cc1cccs1)[C@@H]2N[C@@H](C(=O)NCCNC(=O)[C@@H]3N[C@H](SC3(C)C)[C@H](NC(=O)Cc4cccs4)C(=O)NCC)C(C)(C)S2 | Protease |
| CHEMBL331458 | COc1cc(c(SC2=C(O)OC(CCc3ccccc3)(CC2=O)c4ccccc4)cc1C)C(C)(C)C | Protease |
| CHEMBL89038 | CCCC1(CCc2ccccc2)OC(=C(C(CC)c3cccc(NS(=O)(=O)c4ccc(cc4)C#N)c3)C(=O)O1)O | Protease |
| CHEMBL423189 | COc1ccc(cc1)S(=O)(=O)N(CC(C)C)C[C@@H](O)[C@H](Cc2ccccc2)NC(=O)c3ccc(C)cc3 | Protease |
| CHEMBL116677 | CC(C)(C)NC(=O)[C@@H]1C[C@@H](CN1C[C@@H](O)C[C@@H](Cc2ccccc2)C(=O)NC3[C@H](O)Cc4ccccc34)Oc5ccc6ccccc6c5 | Protease |
| CHEMBL269569 | COCCOCCO[C@H](NC(=O)C(F)(F)C(=O)C(Cc1ccc(OCc2ccccc2)cc1)NC(=O)[C@@H](NC(=O)OCc3ccccc3)C(C)C)C(C)C | Protease |
| CHEMBL309464 | CC(C)C[C@H](NC(=O)OCc1ccccc1)C(=O)NC(Cc2ccc(OCc3ccccc3)cc2)C(=O)C(F)(F)C(=O)NCc4ccccc4 | Protease |
| CHEMBL151617 | CC(C)(C)NC(=O)[C@@H]1CN(CCCC(F)(F)F)CCN1C[C@@H](O)C[C@@H](Cc2ccccc2)C(=O)NC3[C@H](O)Cc4ccccc34 | Protease |
| CHEMBL1790972 | CC[C@@H](C)[C@H](NC(=O)[C@H](CC(C)C)C(O)C(O)[C@H](CC(C)C)NC(=O)C(Cc1c[nH]cn1)NC(=O)COc2cccc3ccccc23)C(=O)NCc4ccccn4 | Protease |
| CHEMBL20930 | CC(C)(C)OC(=O)N[C@@H](Cc1ccccc1)[C@@H](O)C[C@@H](Cc2ccccc2)C(=O)NC3CCCc4ccccc34 | Protease |
| CHEMBL335073 | COC(=O)[C@@H](NC(=O)[C@H](CC(C)C)NC(=O)[C@@H]1CCCN1C[C@@H](O)[C@H](Cc2ccccc2)NC(=O)[C@H](CC(=O)N)NC(=O)OC(C)(C)C)C(C)C | Protease |
| CHEMBL321165 | COC(=O)N[C@@H](C(C)C)C(=O)N[C@@H](Cc1ccccc1)C(O)CN(Cc2ccc(OC)cc2)NC(=O)[C@@H](NC(=O)OC)C(C)C | Protease |
| CHEMBL11287 | COc1ccc(SC[C@H](NS(=O)(=O)C)C(=O)N[C@@H](Cc2ccccc2)[C@H](O)Cc3ccccc3C(=O)NC(C)(C)C)cc1 | Protease |
| CHEMBL324398 | COC(=O)N[C@@H](C(C)C)C(=O)N[C@@H](Cc1ccccc1)C(O)CN(CCC(C)C)NC(=O)[C@@H](NC(=O)OC)C(C)C | Protease |
| CHEMBL357009 | CC(C)(CO)OC(=O)N[C@@H](Cc1ccccc1)[C@H](O)CNC[C@@H](O)[C@H](Cc2ccccc2)NC(=O)OC(C)(C)CO | Protease |
| CHEMBL347431 | CC(C)CCN(C[C@@H](O)[C@H](Cc1ccccc1)NC(=O)OCc2ccccc2)S(=O)(=O)c3ccccc3 | Protease |
| CHEMBL2093070 | CC[C@H](C)[C@H](NC(=O)[C@H]1CCCN1C[C@H](O)[C@H](Cc2ccccc2)NC(=O)[C@H](CC(=O)N)NC(=O)OC(C)(C)C)C(=O)N[C@@H](C(C)C)C(=O)OC | Protease |
| CHEMBL316767 | CC(C)[C@H](NC(=O)CSc1ncccn1)C(=O)N[C@@H](Cc2ccccc2)[C@@H](O)[C@H](O)[C@H](Cc3ccccc3)NC(=O)[C@@H](NC(=O)CSc4ncccn4)C(C)C | Protease |
| CHEMBL21196 | CCC(C)C(NC(=O)[C@@H]1CCCN1C[C@@H](O)[C@@H](Cc2ccccc2)NC(=O)C(CC(=O)N)NC(=O)OC(C)(C)C)C(=O)NC(C(C)C)C(=O)OC | Protease |
| CHEMBL144742 | Clc1ccc2[nH]c(C3=NCCS3)c(c2c1)S(=O)(=O)c4ccccc4 | Protease |
| CHEMBL168384 | Cc1cc(SC2=C(O)OC(C)(CCc3ccc(O)cc3)CC2=O)c(cc1OS(=O)(=O)c4ccc(F)cc4)C(C)(C)C | Protease |
| CHEMBL74275 | CC(C)C1(CCc2cncnc2)CC(=O)C(=C(O)O1)Sc3cc(C)c(CO)cc3C(C)(C)C | Protease |
| CHEMBL326913 | COC(=O)N[C@@H](C(C)C)C(=O)N[C@@H](Cc1ccccc1)C(O)CN(Cc2cccs2)NC(=O)[C@@H](NC(=O)OC)C(C)C | Protease |
| CHEMBL263995 | CC(C)(C)c1ccccc1SC2=C(O)C=C(OC2=O)c3ccccc3 | Protease |
| CHEMBL189858 | Nc1cccc(c1)S(=O)(=O)N(C[C@@H](O)[C@H](Cc2ccccc2)NC(=O)O[C@H]3CO[C@H]4OCC[C@@H]34)OC5CCCC5 | Protease |
| CHEMBL125506 | CCC(C)c1ccccc1SC2=C(O)OC(CC2=O)(c3ccccc3)c4ccccc4 | Protease |
| CHEMBL126058 | CC(C)c1ccc(C)cc1SC2=C(O)C=C(OC2=O)c3ccccc3 | Protease |
| CHEMBL146875 | CC(C)(C)OC(=O)N[C@@H](Cc1ccccc1)[C@@H](O)C[C@]2(Cc3ccccc3)CCN([C@@H]4CS(=O)(=O)Cc5ccccc45)C2=O | Protease |
| CHEMBL314752 | CC(C)[C@@H]1[C@@H](CCS1(=O)=O)OC(=O)N[C@@H](Cc2ccccc2)[C@H](O)CN3CCN(C[C@H]3C(=O)NC(C)(C)C)C4CCC4 | Protease |
| CHEMBL275792 | Cn1nnnc1SC[C@H](NS(=O)(=O)C)C(=O)N[C@@H](Cc2ccccc2)[C@H](O)Cc3ccccc3C(=O)NC(C)(C)C | Protease |
| CHEMBL93082 | CC(C)(C)NC(=O)[C@@H]1CCCCN1C[C@@H](O)[C@H](Cc2ccccc2)NC(=O)[C@H](CC(=O)N)NC(=O)OCc3ccccc3 | Protease |
| CHEMBL1790515 | CC[C@H](C)[C@H](NC(=O)[C@H](C(C)C)C(O)[C@H](O)[C@H](CC1CCCCC1)NC(=O)c2ccccc2OCOc3ccccc3)C(=O)NCc4nc5ccccc5[nH]4 | Protease |
| CHEMBL431976 | CC(C)C1(CCc2csc(NC(=O)C)n2)CC(=O)C(=C(O)O1)Sc3cc(C)c(CO)cc3C(C)(C)C | Protease |
| CHEMBL292791 | CC(C)(C)c1oc2ccccc2c1SC3=C(O)OC(CCc4ccccc4)(CC3=O)c5ccccc5 | Protease |
| CHEMBL324657 | COC(=O)N[C@@H](C(C)C)C(=O)N[C@@H](Cc1ccccc1)C(O)CN(Cc2ccc(O)cc2)NC(=O)[C@@H](NC(=O)OC)C(C)C | Protease |
| CHEMBL171873 | CC(C)C1(CCc2ccc(O)cc2)CC(=O)C(=C(O)O1)Sc3cc(C)c(NS(=O)(=O)c4ccc(cc4)C(F)(F)F)cc3C(C)(C)C | Protease |
| CHEMBL90768 | CC(C)[C@@H]1[C@@H](CCS1(=O)=O)OC(=O)N[C@@H](Cc2ccccc2)[C@H](O)CN3CCN(C[C@H]3C(=O)NC(C)(C)C)C4CCCC4 | Protease |
| CHEMBL169247 | CC(C)C1(CCc2ccc(N)cc2)CC(=O)C(=C(O)O1)Sc3cc(C)c(OS(=O)(=O)c4cccs4)cc3C(C)(C)C | Protease |
| CHEMBL309346 | CC(C)(C)NC(=O)[C@@H]1CN(CCN1C[C@@H](O)[C@H](Cc2ccccc2)NC(=O)O[C@H]3CCOC3)C(=O)OCc4ccccc4 | Protease |
| CHEMBL98302 | CC(C)(C)NC(=O)N(C[C@@H](O)[C@H](Cc1ccccc1)NC(=O)[C@H](CC(=O)N)NC(=O)c2ccc3ccccc3n2)Cc4ccncc4 | Protease |
| CHEMBL324377 | COC(=O)N[C@@H](C(C)C)C(=O)N[C@@H](Cc1ccccc1)C(O)CN(Cc2ccc(C)cc2)NC(=O)[C@@H](NC(=O)OC)C(C)C | Protease |
| CHEMBL69651 | CC(C)(C)NC(=O)C[C@@H](C(=O)NC[C@@H](O)[C@H](Cc1ccccc1)NC(=O)OCc2ccccc2)C(C)(C)C | Protease |
| CHEMBL432787 | CCNC(=O)[C@@H](NC(=O)Cc1ccccn1)[C@@H]2N[C@@H](C(=O)NCCNC(=O)[C@@H]3N[C@H](SC3(C)C)[C@H](NC(=O)Cc4ccccn4)C(=O)NCC)C(C)(C)S2 | Protease |
| CHEMBL325107 | CC(C)(C)OC(=O)N[C@@H](Cc1ccccc1)[C@@H](O)C[C@@H](Cc2ccccc2)C(=O)N[C@@H]3CCc4ccccc34 | Protease |
| CHEMBL137282 | CC(C)(C)NC(=O)[C@@H]1CCCN1C(=O)[C@@H](O)[C@@H]2Cc3ccc(OCCCOc4cc5ccccc5cc4C(=O)N[C@@H](CC(=O)N)C(=O)N2)cc3 | Protease |
| CHEMBL329068 | CC(C)(C)NC(=O)N(C[C@@H](O)[C@H](Cc1ccccc1)NC(=O)[C@H](CC(=O)N)NC(=O)OCc2ccccc2)Cc3ccccc3 | Protease |
| CHEMBL423648 | CC(=O)N[C@@H](CC(=O)N)C(=O)N[C@@H](CSc1ccccc1)[C@H](O)Cc2ccccc2C(=O)NC(C)(C)C | Protease |
| CHEMBL74654 | CC(C)C1(CCc2ncc(C)s2)CC(=O)C(=C(O)O1)Sc3cc(C)c(CO)cc3C(C)(C)C | Protease |
| CHEMBL71852 | CC(C)NC(=O)C[C@@H](C(=O)NC[C@@H](O)[C@H](Cc1ccccc1)NC(=O)OCc2ccccc2)C(C)(C)C | Protease |
| CHEMBL77621 | CC(C)[C@H](NC(=O)[C@@H](NC(=O)[C@@H]1CCCN1C[C@H](Cc2ccccc2)NC(=O)[C@H](C)NC(=O)[C@H](C)NC(=O)[C@@H](N)CO)C(C)C)C(=O)N | Protease |
| CHEMBL133883 | CC(C)(C)N(C[C@@H](O)[C@H](Cc1ccccc1)NC(=O)[C@H](CC(=O)N)NC(=O)c2ccc3ccccc3n2)C(=O)NCc4ccncc4 | Protease |
| CHEMBL134296 | CC(C)(C)N(C[C@@H](O)[C@H](Cc1ccccc1)NC(=O)[C@H](CC(=O)N)NC(=O)OCc2ccccc2)C(=O)NCc3ccccc3 | Protease |
| CHEMBL290765 | CC(C)(C)NC(=O)N(CC(O)[C@H](Cc1ccccc1)NC(=O)[C@H](CC(=O)N)NC(=O)OCc2ccccc2)Cc3ccccc3 | Protease |
| CHEMBL71134 | CC(C)(C)NC(=O)N(CC(O)[C@H](Cc1ccccc1)NC(=O)[C@H](CC(=O)N)NC(=O)c2ccc3ccccc3n2)Cc4ccncc4 | Protease |
| CHEMBL273424 | CC(C)(C)NC(=O)c1ccccc1C[C@@H](O)[C@H](Cc2ccccc2)NC(=O)[C@H](CS(=O)(=O)Cc3ccccc3)NS(=O)(=O)C | Protease |
| CHEMBL274128 | CC(Oc1c(C)cccc1C)C(=O)N[C@@H](Cc2ccccc2)[C@H](O)CN3CC[C@H](C[C@H]3C(=O)NC(C)(C)C)OCc4ccncc4 | Protease |
| CHEMBL294097 | CC(C)(C)OC(=O)N[C@@H](Cc1ccc(OCc2ccccc2)cc1)C(=O)N3CCC(=O)[C@H]3Sc4ccccc4 | Protease |
| CHEMBL24181 | CC(C)(C)NC(=O)[C@H]1CCCN1C(=O)C(=O)[C@H](Cc2ccccc2)NC(=O)[C@H](CC(=O)N)NC(=O)OCc3ccccc3 | Protease |
| CHEMBL1790663 | CC[C@H](C)[C@H](NC(=O)CN(CC=C)C(=O)[C@H](Cc1ccccc1)NC(=O)[C@H](C)NC(=O)[C@H](C)NC(=O)[C@H](CO)NC(=O)OC(C)(C)C)C(=O)N[C@@H](C(C)C)C(=O)OC | Protease |
| CHEMBL275960 | CC(=O)N[C@@H](CS(=O)(=O)c1ccccc1)C(=O)N[C@@H](Cc2ccccc2)[C@H](O)Cc3ccccc3C(=O)NC(C)(C)C | Protease |
| CHEMBL3085515 | CC(C)(C)NC(=O)[C@@H]1CCCN1C(=O)C(=O)[C@H](Cc2ccccc2)NC(=O)[C@H](CC(=O)N)NC(=O)OCc3ccccc3 | Protease |
| CHEMBL362787 | COc1ccc(cc1)S(=O)(=O)N(C[C@@H](O)[C@H](Cc2ccccc2)NC(=O)O[C@H]3CO[C@H]4OCC[C@@H]34)OC5CCCC5 | Protease |
| CHEMBL445105 | CC(C)(C)OC(=O)N[C@@H](Cc1ccc(OCc2ccccc2)cc1)C(=O)N3CCC(=O)[C@@H]3Sc4ccccc4 | Protease |
| CHEMBL71980 | CN(C)S(=O)(=O)Oc1cc(c(SC2=C(O)OC(CCc3ccc(O)cc3)(CC2=O)C4CCCCC4)cc1C)C(C)(C)C | Protease |
| CHEMBL558604 | Nc1nc(cs1)c2[nH]c3ccc(Cl)cc3c2S(=O)(=O)c4ccccc4 | Protease |
| CHEMBL289799 | Cc1cc(SC2=C(O)OC(CCc3ccc(O)cc3)(CC2=O)C4CCCCC4)c(cc1N)C(C)(C)C | Protease |
| CHEMBL333611 | CC(C)[C@@H](NC(=O)OCCN1CCOCC1)C(=O)N[C@@H](Cc2ccccc2)[C@H](O)[C@H](O)[C@H](Cc3ccccc3)NC(=O)[C@@H](NC(=O)OCCN4CCOCC4)C(C)C | Protease |
| CHEMBL3143341 | CC(C)C[C@H](NC(=O)[C@@H](C[C@H](O)[C@H](Cc1ccccc1)NC(=O)[C@H](Cc2ccccc2)NC(=O)OC(C)(C)C)Cc3ccccc3)C(=O)N[C@@H](Cc4ccccc4)C(=O)N | Protease |
| CHEMBL17969 | CCC[C@@H]1[C@@H](CCS1(=O)=O)OC(=O)N[C@@H](Cc2ccccc2)[C@H](O)CN3C[C@H]4CCCC[C@H]4C[C@H]3C(=O)NC(C)(C)C | Protease |
| CHEMBL201918 | CC(C)(C)OC(=O)N[C@@H](Cc1ccccc1)[C@@H](O)C[C@]2(Cc3ccccc3)N=CC(=C2O)C4C[C@H](CC(=O)N)c5ccccc45 | Protease |
| CHEMBL122402 | CC(C)(C)NC(=O)[C@H]1N(CC(O)CNC(=O)[C@@H]2N[C@H](SC2(C)C)[C@H](NC(=O)Cc3ccccc3)C(=O)NCc4ccccc4)CSC1(C)C | Protease |
| CHEMBL106991 | CC(C)(C)OC(=O)CNC(=O)[C@@H](NC(=O)Cc1ccccc1)[C@@H]2N[C@@H](C(=O)NCCNC(=O)[C@@H]3N[C@H](SC3(C)C)[C@H](NC(=O)Cc4ccccc4)C(=O)NCC(=O)OC(C)(C)C)C(C)(C)S2 | Protease |
| CHEMBL2079666 | CC(C)(C)OC(=O)N[C@@H](Cc1ccccc1)[C@@H](O)C[C@@H](Cc2ccccc2)C(=O)N[C@H]3CCc4ccccc34 | Protease |
| CHEMBL171404 | Cc1cc(SC2=C(O)OC(CCc3ccc(O)cc3)(CC2=O)C4CCCCC4)c(cc1OS(=O)(=O)c5ccc(cc5)C#N)C(C)(C)C | Protease |
| CHEMBL169142 | CC(C)C1(CCc2ccc(O)cc2)CC(=O)C(=C(O)O1)Sc3cc(C)c(OS(=O)(=O)c4cccs4)cc3C(C)(C)C | Protease |
| CHEMBL90510 | Nc1ccc(cc1O)C(=O)N[C@H](C[C@H](O)[C@H](Cc2ccccc2)NC(=O)OCc3ccccc3)Cc4ccccc4 | Protease |
| CHEMBL442010 | CC(C)[C@@H](NC(=O)N(C)CCc1ccccn1)C(=O)N[C@@H](Cc2ccccc2)[C@H](O)[C@H](O)[C@H](Cc3ccccc3)NC(=O)[C@@H](NC(=O)N(C)CCc4ccccn4)C(C)C | Protease |
| CHEMBL109220 | CC(C)[C@@H](NC(=O)CCc1cccnc1)C(=O)N[C@@H](Cc2ccccc2)C(O)[C@H](Cc3ccccc3)NC(=O)[C@@H](NC(=O)CCc4cccnc4)C(C)C | Protease |
| CHEMBL13136 | CC(C)c1ccccc1SC2=C(O)C=C(OC2=O)c3ccc(Cl)c(Cl)c3 | Protease |
| CHEMBL273437 | CC(C)(C)NC(=O)c1ccccc1C[C@@H](O)[C@H](Cc2ccccc2)NC(=O)[C@H](CS(=O)(=O)c3ccccn3)NS(=O)(=O)C | Protease |
| CHEMBL23456 | CNC(=O)c1[nH]c2ccc(Cl)cc2c1Sc3ccccc3 | Protease |
| CHEMBL279279 | CC(C)(C)OC(=O)N[C@@H](Cc1ccccc1)[C@@H](O)C[C@@H](Cc2ccccc2)C(=O)NC3Cc4ccccc34 | Protease |
| CHEMBL97512 | CC(C)[C@H](NC(=O)OCc1ccccc1)C(=O)N[C@@H](Cc2ccccc2)[C@@H](O)CN(Cc3ccc(F)cc3)NC(=O)[C@@H](NC(=O)OCc4ccccc4)C(C)C | Protease |
| CHEMBL277825 | CC(C)(C)NC(=O)[C@@H]1C[C@@H]2CCCC[C@@H]2CN1C[C@@H](O)[C@H](Cc3ccccc3)NC(=O)O[C@@H]4CCS(=O)(=O)[C@@H]4C5CCCC5 | Protease |
| CHEMBL139237 | CC(C)(C)OC(=O)[C@@H]1CCCN1C[C@@H](O)[C@H](Cc2ccccc2)NC(=O)[C@H](CC(=O)N)NC(=O)c3ccc4ccccc4n3 | Protease |
| CHEMBL263236 | CC(C)C[C@H](NC(=O)[C@@H](C[C@H](O)[C@H](Cc1ccccc1)NC(=O)OC(C)(C)C)Cc2ccccc2)C(=O)NCCCN(C)C | Protease |
| CHEMBL121920 | CC(C)(C)NC(=O)[C@@H]1C[C@@H]2CCCC[C@@H]2CN1C[C@@H](O)CNC(=O)[C@@H]3N[C@H](SC3(C)C)C(NC(=O)Cc4ccccc4)C(=O)N | Protease |
| CHEMBL23401 | NC(=O)c1[nH]c2ccc(Cl)cc2c1[S+]([O-])c3ccccc3 | Protease |
| CHEMBL324630 | COC(=O)NC(C(C)C)C(=O)NN(CC(C)C)C[C@H](O)[C@H](Cc1ccccc1)NC(=O)[C@H](CC(=O)N)NC(=O)c2ccc3ccccc3n2 | Protease |
| CHEMBL122171 | CC(C)(C)NC(=O)[C@@H]1C[C@@H]2CCCC[C@@H]2CN1C[C@@H](O)CNC(=O)[C@@H]3N[C@H](SC3(C)C)C(NC(=O)Cc4ccccc4)C(=O)NCc5ccccc5 | Protease |
| CHEMBL12714 | CC(C)c1ccccc1SC2=C(O)C=C(OC2=O)c3cccc(Cl)c3 | Protease |
| CHEMBL124892 | CC(C)c1ccccc1SC2=C(O)OC(CC2=O)(c3ccccc3)c4ccccc4 | Protease |
| CHEMBL541982 | COc1cccc(c1)C(=O)N[C@@H](Cc2ccccc2)[C@@H](O)C[C@H](Cc3ccccc3)NC(=O)OC(C)(C)C | Protease |
| CHEMBL109699 | CC(C)[C@@H](NC(=O)CCN1CCOCC1)C(=O)N[C@@H](Cc2ccccc2)[C@H](O)[C@H](O)[C@H](Cc3ccccc3)NC(=O)[C@@H](NC(=O)CCN4CCOCC4)C(C)C | Protease |
| CHEMBL133585 | CC(C)(C)NC(=O)C1CCCCN1C[C@H](O)[C@H](Cc2ccccc2)NC(=O)C(CC#N)NC(=O)OCc3ccccc3 | Protease |
| CHEMBL337681 | CC(C)(C)OC(=O)C1CCCN1C[C@H](O)[C@H](Cc2ccccc2)NC(=O)[C@H](CC(=O)N)NC(=O)c3ccc4ccccc4n3 | Protease |
| CHEMBL436252 | CC(C)(C)NC(=O)[C@@H]1C[C@@H]2CCCC[C@@H]2CN1C[C@@H](O)[C@H](Cc3ccccc3)NC(=O)[C@H]4CCCS(=O)(=O)C4 | Protease |
| CHEMBL322939 | CC(C)[C@@H](NS(=O)(=O)CCc1ccncc1)C(=O)N[C@@H](Cc2ccccc2)[C@@H](O)[C@H](O)[C@H](Cc3ccccc3)NC(=O)[C@@H](NS(=O)(=O)CCc4ccncc4)C(C)C | Protease |
| CHEMBL187707 | COc1ccc(cc1)S(=O)(=O)N(CC(O)C(Cc2ccccc2)NC(=O)[C@H](CC(=O)N)NC(=O)c3ccc4ccccc4n3)OC5CCCC5 | Protease |
| CHEMBL95462 | CC(C)[C@H](NC(=O)OCc1ccccc1)C(=O)N[C@@H](Cc2ccccc2)[C@@H](O)CN(Cc3ccc(cc3)C#N)NC(=O)[C@@H](NC(=O)OCc4ccccc4)C(C)C | Protease |
| CHEMBL99774 | CC(C)(C)NC(=O)[C@@H]1C[C@@H]2CCCC[C@@H]2CN1C[C@@H](O)[C@H](Cc3ccccc3)NC(=O)[C@@H](CSc4ccc(F)cc4)NS(=O)(=O)C | Protease |
| CHEMBL123183 | Cc1ccc(c(SC2=C(O)OC(CCc3ccc(N)cc3)(CC2=O)c4ccccc4)c1)C(C)(C)C | Protease |
| CHEMBL133764 | CC[C@@H](C)[C@H](NC(=O)[C@H](C(C)C)C(O)C(O)[C@H](CC1CCCCC1)NC(=O)C(Cc2c[nH]cn2)NC(=O)CN)C(=O)NCc3ccccn3 | Protease |
| CHEMBL305063 | CC(C)[C@@H]1NC(=O)\C=C/CCCCCOc2ccc(C[C@H](NC1=O)[C@H](O)CN3CC4CCCCC4C[C@H]3C(=O)NC(C)(C)C)cc2 | Protease |
| CHEMBL1790968 | CC[C@H](C)[C@H](NC(=O)C1CCCN1C(=O)[C@H](O)[C@H](Cc2ccccc2)NC(=O)[C@H](CC(=O)N)NC(=O)OCc3ccccc3)C(=O)N[C@@H](C(C)C)C(=O)OC | Protease |
| CHEMBL356947 | CCCNC(=O)[C@@H](NC(=O)Cc1ccccc1)[C@@H]2N[C@@H](C(=O)NCC(CO)NC(=O)[C@@H]3N[C@H](SC3(C)C)[C@H](NC(=O)Cc4ccccc4)C(=O)NCC)C(C)(C)S2 | Protease |
| CHEMBL281159 | CCC(C)[C@H](NC(=O)[C@H]1CCCN1C(=O)C(O)[C@H](Cc2ccccc2)NC(=O)[C@H](CC(=O)N)NC(=O)OCc3ccccc3)C(=O)N[C@@H](C(C)C)C(=O)OC | Protease |
| CHEMBL302902 | CC(C)(C)OC(=O)N[C@@H](Cc1ccc(O)cc1)C(=O)N2CCC(=O)[C@@H]2Sc3ccccc3 | Protease |
| CHEMBL304804 | CC(C)[C@H](NC(=O)OCc1ccccc1)C(=O)N[C@@H](Cc2ccccc2)[C@H](O)CNC(=O)[C@@H](CC(=O)NC(C)(C)C)C(C)(C)C | Protease |
| CHEMBL378214 | COC(=O)N[C@@H](C(c1ccccc1)c2ccccc2)C(=O)NCCCC[C@H](CO)N(CC(C)C)S(=O)(=O)c3ccc(N)cc3 | Protease |
| CHEMBL113268 | CC(C)[C@@H](NC(=O)OCc1ccncc1)C(=O)N[C@@H](Cc2ccccc2)C(O)[C@H](Cc3ccccc3)NC(=O)[C@@H](NC(=O)OCc4ccncc4)C(C)C | Protease |
| CHEMBL329974 | CN(C)CCCNC(=O)[C@@H](NC(=O)[C@@H](C[C@H](O)[C@H](Cc1ccccc1)NC(=O)OC(C)(C)C)Cc2ccccc2)c3ccccc3 | Protease |
| CHEMBL333982 | CN(C)c1ccc(CNC(=O)C(NC(=O)Cc2ccccc2)[C@@H]3N[C@@H](C(=O)NC[C@H](O)CN4C[C@H]5CCCC[C@H]5C[C@H]4C(=O)NC(C)(C)C)C(C)(C)S3)cc1 | Protease |
| CHEMBL324504 | CC(C)(C)NC(=O)[C@@H]1C[C@H](Cl)CN1C(=O)[C@@H](O)[C@H](Cc2ccccc2)NC(=O)c3ccccc3 | Protease |
| CHEMBL3085514 | CC(C)(C)NC(=O)[C@@H]1CCCCN1C(=O)[C@@H](O)[C@H](Cc2ccccc2)NC(=O)[C@H](CC(=O)N)NC(=O)OCc3ccccc3 | Protease |
| CHEMBL324989 | CCC(C)[C@H](NC(=O)C[C@H](O)[C@H](CC1CCCCC1)NC(=O)CC2CCCN3N2C(=O)C(CN)CCC3=O)C(=O)N[C@@H](Cc4ccccc4)C(=O)N | Protease |
| CHEMBL293394 | CC(C)(C)OC(=O)N[C@@H](Cc1ccc(O)cc1)C(=O)N2CCC(=O)[C@H]2Sc3ccccc3 | Protease |
| CHEMBL164570 | CC(C)c1ccccc1SC2=C(O)OC(CCCCC(=O)NCc3ccccc3)(CC2=O)c4ccccc4 | Protease |
| CHEMBL12224 | CC(C)c1ccccc1SC2=C(O)C=C(OC2=O)c3cc(Cl)cc(Cl)c3 | Protease |
| CHEMBL48804 | CC[C@H](C)[C@H](NC(=O)[C@H](Cc1ccccc1)[C@@H](O)[C@H](O)[C@H](Cc2ccccc2)NC(=O)[C@H](Cc3c[nH]cn3)NC(=O)COc4cccc5ccccc45)C(=O)NCc6ccccn6 | Protease |
| CHEMBL3085513 | CC(C)(C)NC(=O)[C@@H]1C[C@@H]2CCCC[C@@H]2CN1C(=O)[C@@H](O)[C@H](Cc3ccccc3)NC(=O)[C@H](CC(=O)N)NC(=O)c4ccc5ccccc5n4 | Protease |
| CHEMBL341697 | COc1ccc(cc1)S(=O)(=O)N(CC(C)C)C[C@@H](O)[C@H](Cc2ccccc2)NC(=O)c3cccc(C)c3 | Protease |
| CHEMBL154952 | CC(C)CCN(C[C@@H](O)[C@H](Cc1ccccc1)NC(=O)[C@H](CC(=O)N)NC(=O)c2ccc3ccccc3n2)S(=O)(=O)C | Protease |
| CHEMBL224483 | CC(C)(C)[C@H](NC(=O)OCc1ccccc1)C(=O)NC(Cc2ccccc2)C(=O)C(F)(F)C(=O)NCc3ccccc3 | Protease |
| CHEMBL322119 | COC(=O)NC(C(C)C)C(=O)NN(C[C@H](O)[C@H](Cc1ccccc1)NC(=O)[C@H](CC(=O)N)NC(=O)c2ccc3ccccc3n2)CC4CCCCC4 | Protease |
| CHEMBL91995 | CC(C)CN1CCN(C[C@@H](O)[C@H](Cc2ccccc2)NC(=O)O[C@@H]3CCS(=O)(=O)[C@@H]3C(C)C)[C@@H](C1)C(=O)NC(C)(C)C | Protease |
| CHEMBL321503 | CC(C)(C)OC(=O)N[C@@H](Cc1ccccc1)[C@@H](O)C[C@@H](CC=C)C(=O)N[C@H]2[C@H](O)Cc3ccccc23 | Protease |
| CHEMBL321641 | CC(C)(C)OC(=O)N[C@@H](Cc1ccccc1)[C@@H](O)C[C@@H](CC=C)C(=O)N[C@@H]2[C@H](O)Cc3ccccc23 | Protease |
| CHEMBL138829 | CC(C)(C)OC(=O)N[C@@H](Cc1ccccc1)[C@@H](O)C[C@@H](CC=C)C(=O)NC2[C@@H](O)Cc3ccccc23 | Protease |
| CHEMBL405134 | CC(C)(C)NC(=O)[C@@H]1CN(Cc2cccc(c2)C(C)(C)NC(=O)OC(C)(C)C)CCN1C[C@@H](O)C[C@@H](Cc3ccccc3)C(=O)NC4[C@H](O)Cc5ccccc45 | Protease |
| CHEMBL29010 | Cc1cccc(C[C@@H](O)[C@H](Cc2ccccc2)NC(=O)[C@H](CC(=O)N)NC(=O)c3ccc4ccccc4n3)c1C(=O)NC(C)(C)C | Protease |
| CHEMBL340472 | Cc1ccc(c(SC2=C(O)C=C(OC2=O)c3ccccc3)c1)C(C)(C)C | Protease |
| CHEMBL61755 | CCc1c(SC2=C(O)OC(CCc3ccccc3)(CC2=O)c4ccccc4)sc5ccccc15 | Protease |
| CHEMBL418680 | CC(C)(C)c1cc2CCCCc2cc1SC3=C(O)OC(CCc4ccccc4)(CC3=O)c5ccccc5 | Protease |
| CHEMBL411507 | CC(C)[C@@H](NC(=O)OCc1ccccc1)C(=O)N[C@@H](Cc2ccccc2)[C@H](O)[C@H](Cc3ccccc3)NC(=O)[C@@H](NC(=O)N4CCN(C)CC4)C(C)C | Protease |
| CHEMBL320671 | CC(C)[C@H](NC(=O)C)C(=O)N[C@@H](Cc1ccccc1)[C@@H](O)CN(Cc2ccc(F)cc2)NC(=O)[C@@H](NC(=O)C)C(C)C | Protease |
| CHEMBL97423 | CC(C)(C)OC(=O)N[C@@H](Cc1ccccc1)[C@@H](O)C[C@@H](Cc2ccccc2)C(=O)N[C@@H](Cc3ccccc3)C(=O)N | Protease |
| CHEMBL355735 | Cc1cc(SC2=C(O)OC(CCc3ccc(O)cc3)(CC2=O)C4CCCCC4)c(cc1OS(=O)(=O)c5ccc(F)cc5)C(C)(C)C | Protease |
| CHEMBL144409 | Clc1ccc2[nH]c(C3=N[C@H]4CCCC[C@@H]4N3)c(c2c1)S(=O)(=O)c5ccccc5 | Protease |
| CHEMBL70394 | CC(C)(C)NC(=O)N(CC(O)[C@H](Cc1ccccc1)NC(=O)[C@H](CC(=O)N)NC(=O)OCc2ccccc2)C3CCCCC3 | Protease |
| CHEMBL40829 | CC[C@H](C)[C@H](NC(=O)[C@@H](CC(O)CN1CCCCC1C(=O)NC(C)(C)C)Cc2ccccc2)C(=O)NCc3nc4ccccc4[nH]3 | Protease |
| CHEMBL2115378 | CC[C@H](C)[C@H](NC(=O)[C@@H](C[C@H](O)CN1C[C@H]2CCCC[C@H]2C[C@H]1C(=O)NC(C)(C)C)Cc3ccccc3)C(=O)NCc4nc5ccccc5[nH]4 | Protease |
| CHEMBL17852 | CC(C)(C)NC(=O)[C@@H]1C[C@@H]2CCCC[C@@H]2CN1C[C@@H](O)[C@H](Cc3ccccc3)NC(=O)C4CO[C@@H]5CCS(=O)(=O)[C@@H]5C4 | Protease |
| CHEMBL222559 | CCC[C@@]1(CCc2ccccc2)CC(=C([C@H](CC)c3cccc(NS(=O)(=O)c4ccc(cn4)C(F)(F)F)c3)C(=O)O1)O | Protease |
| CHEMBL2114445 | CC[C@H](C)[C@H](NC(=O)[C@@H](C[C@@H](O)CN1C[C@H]2CCCC[C@H]2C[C@H]1C(=O)NC(C)(C)C)Cc3ccccc3)C(=O)NCc4nc5ccccc5[nH]4 | Protease |
| CHEMBL94497 | CC(C)[C@@H](NC(=O)C)C(=O)NN(C[C@H](O)[C@H](Cc1ccccc1)NC(=O)[C@@H](NC(=O)C)C(C)C)Cc2ccccc2 | Protease |
| CHEMBL291773 | CC(C)(C)OC(=O)N[C@@H](Cc1ccccc1)[C@H](O)CNC[C@@H](O)[C@H](Cc2ccc(COC(=O)N3CCOCC3)cc2)NC(=O)OC(C)(C)C | Protease |
| CHEMBL170576 | CC(C)C[C@@H]1[C@@H](CCS1(=O)=O)OC(=O)N[C@@H](Cc2ccccc2)[C@H](O)CN3C[C@H]4CCCC[C@H]4C[C@H]3C(=O)NC(C)(C)C | Protease |
| CHEMBL415896 | CC[C@H](C)[C@H](NC(=O)[C@H](CC(C)C)CC(O)CN1C[C@H]2CCCC[C@H]2C[C@H]1C(=O)NC(C)(C)C)C(=O)NCc3nc4ccccc4[nH]3 | Protease |
| CHEMBL289409 | CC(C)CN1CC(O)(CNC(=O)[C@@H]2N[C@H](SC2(C)C)[C@H](NC(=O)Cc3ccccc3)C(=O)NCc4ccccc4)C[C@H]1C(=O)NC(C)(C)C | Protease |
| CHEMBL359518 | COc1ccc(cc1)S(=O)(=O)N(C[C@@H](O)CN2[C@@H](Cc3ccccc3)COC(Cc4ccccc4)C2=O)CC5CCCC5 | Protease |
| CHEMBL9650 | CCC(C)[C@H](NC(=O)[C@@H]1CCCN1[P@@](=O)(OC)[C@@H](Cc2ccccc2)NC(=O)[C@H](CC(=O)N)NC(=O)OC(C)(C)C)C(=O)NCC(C)C | Protease |
| CHEMBL290163 | COC(=O)[C@H](Cc1ccccc1)NC(=O)[C@H](CC(C)C)NC(=O)[C@@H](CC(O)C[C@@H](Cc2ccccc2)C(=O)N[C@@H](CC(C)C)C(=O)N[C@@H](Cc3ccccc3)C(=O)OC)Cc4ccccc4 | Protease |
| CHEMBL283619 | FC(F)(F)[C@]1(NC(=O)Nc2ccc(Cl)cc12)C#CC3CC3 | Protease |
| CHEMBL320783 | COC(=O)NC(C(C)C)C(=O)NN(C[C@H](O)[C@H](Cc1ccccc1)NC(=O)[C@H](CC(=O)N)NC(=O)c2ccc3ccccc3n2)Cc4ccc(OC)cc4 | Protease |
| CHEMBL330980 | CCNC(=O)C(NC(=O)Cc1ccccc1)[C@@H]2N[C@@H](C(=O)NCC(O)CN3C[C@H]4CCCC[C@H]4C[C@H]3C(=O)NC(C)(C)C)C(C)(C)S2 | Protease |
| CHEMBL93120 | CC(C)(C)OC(=O)N[C@H](C[C@H](O)[C@H](Cc1ccccc1)NC(=O)c2c(Cl)ccc(N)c2Cl)Cc3ccccc3 | Protease |
| CHEMBL263534 | CC(C)[C@@H](NC(=O)N(C)CCc1ccccn1)C(=O)N[C@@H](Cc2ccccc2)[C@@H](O)[C@@H](O)[C@H](Cc3ccccc3)NC(=O)[C@@H](NC(=O)N(C)CCc4ccccn4)C(C)C | Protease |
| CHEMBL91102 | O[C@@H](C[C@H](Cc1ccccc1)NC(=O)c2ccccc2)[C@H](Cc3ccccc3)NC(=O)OCc4ccccc4 | Protease |
| CHEMBL122320 | OC1=C(Sc2ccccc2C3CCCCC3)C(=O)CC(CCc4ccccc4)(O1)c5ccccc5 | Protease |
| CHEMBL331846 | Cc1ccc(c(SC2=C(O)OC(CCc3ccccc3)(CC2=O)c4ccc(N)cc4)c1)C(C)(C)C | Protease |
| CHEMBL97547 | CC(C)[C@@H](NC(=O)OCc1ccccc1)C(=O)NN(C[C@H](O)[C@H](Cc2ccccc2)NC(=O)[C@@H](NC(=O)OCc3ccccc3)C(C)C)Cc4ccccc4 | Protease |
| CHEMBL280464 | COC(=O)N[C@@H](C(C)C)C(=O)N[C@@H](Cc1ccccc1)C(O)CN(Cc2ccc(cc2)C(F)(F)F)NC(=O)[C@@H](NC(=O)OC)C(C)C | Protease |
| CHEMBL339407 | Cc1cc(SC2=C(O)OC(CCc3ccccc3)(CC2=O)c4ccccc4)c(cc1O)C(C)(C)C | Protease |
| CHEMBL324732 | CC(C)(C)NC(=O)[C@@H]1C[C@H](Cl)CN1C(=O)[C@@H](O)[C@H](Cc2ccccc2)NC(=O)c3ccc(O)cc3 | Protease |
| CHEMBL1790661 | CC[C@H](C)[C@H](NC(=O)CN(CC(Cl)CCl)C(=O)[C@H](Cc1ccccc1)NC(=O)OC(C)(C)C)C(=O)N[C@@H](C(C)C)C(=O)OC | Protease |
| CHEMBL21166 | CC(C)(C)OC(=O)N[C@@H](Cc1ccccc1)[C@@H](O)C[C@@H](Cc2ccccc2)C(=O)NC3CCc4ccccc34 | Protease |
| CHEMBL325561 | COC(=O)N[C@@H](C(C)C)C(=O)N[C@@H](Cc1ccccc1)C(O)CN(Cc2ccc(OC)c(OC)c2OC)NC(=O)[C@@H](NC(=O)OC)C(C)C | Protease |
| CHEMBL276097 | CC(C)OC(=O)c1ccccc1CSC2=C(O)C=C(OC2=O)c3ccccc3 | Protease |
| CHEMBL322221 | CC(C)(C)OC(=O)N[C@@H](Cc1ccccc1)C(O)C[C@@H](Cc2ccccc2)C(=O)N[C@H]3CCc4ccccc34 | Protease |
| CHEMBL333827 | Cc1ccccc1C[C@H](NC(=O)c2c(C)ccc(O)c2C)[C@H](O)C(=O)N3C[C@@H](Cl)C[C@H]3C(=O)NC(C)(C)C | Protease |
| CHEMBL88757 | CC(C)[C@@H]1[C@@H](CCS1(=O)=O)OC(=O)N[C@@H](Cc2ccccc2)[C@H](O)CN3CCN(C[C@H]3C(=O)NC(C)(C)C)C4CCOCC4 | Protease |
| CHEMBL297633 | CC[C@H](C[C@H](O)[C@H](Cc1ccccc1)NC(=O)OC(C)(C)C)C(=O)N[C@@H]2[C@H](O)Cc3ccccc23 | Protease |
| CHEMBL71928 | CC(C)CN(CC(O)[C@H](Cc1ccccc1)NC(=O)[C@H](CC(=O)N)NC(=O)OCc2ccccc2)C(=O)NC(C)(C)C | Protease |
| CHEMBL133022 | CC(C)CNC(=O)N(C[C@@H](O)[C@H](Cc1ccccc1)NC(=O)[C@H](CC(=O)N)NC(=O)OCc2ccccc2)C(C)(C)C | Protease |
| CHEMBL12768 | CCOC(=O)c1ccc(cc1)C2=CC(=C(Sc3ccccc3C(C)C)C(=O)O2)O | Protease |
| CHEMBL148090 | CC(C)[C@@H](CO)N1CC[C@](C[C@H](O)[C@H](Cc2ccccc2)NC(=O)OC(C)(C)C)(Cc3ccccc3)C1=O | Protease |
| CHEMBL325800 | CCNC(=O)[C@@H](NC(=O)Cc1ccccc1)[C@@H]2N[C@@H](C(=O)NCCNC(=O)[C@@H]3N[C@@H](CNC(=O)Cc4ccccc4)SC3(C)C)C(C)(C)S2 | Protease |
| CHEMBL143470 | CC(C)(C)OC(=O)N[C@@H](Cc1ccccc1)[C@H](O)CNC[C@@H](O)[C@H](Cc2ccccc2)NC(=O)OC(C)(C)CO | Protease |
| CHEMBL143235 | CCCC1(CCc2ccccc2)CC(=O)C(=C(O)O1)C(CC)c3ccccc3 | Protease |
| CHEMBL99211 | CC(C)CN(C[C@@H](O)[C@H](Cc1ccccc1)NC(=O)[C@H](CC(=O)N)NC(=O)OCc2ccccc2)C(=O)NC(C)(C)C | Protease |
| CHEMBL1790647 | CC[C@H](C)[C@H](NC(=O)CN(CC=C)C(=O)[C@H](Cc1ccccc1)NC(=O)[C@H](C)NC(=O)OC(C)(C)C)C(=O)N[C@@H](C(C)C)C(=O)OC | Protease |
| CHEMBL71863 | CC(C)[C@H]([C@@H](O)[C@H](O)[C@H](CC1CCCCC1)NC(=O)c2ccccc2)C(=O)N[C@H]3Cc4ccccc4[C@H]3O | Protease |
| CHEMBL268871 | CCc1cc(NCc2oc3c(Cl)ccc(Cl)c3n2)c(O)nc1C | Protease |
| CHEMBL408108 | CC1(C)S[C@@H](N[C@H]1C(=O)NC[C@H](O)[C@@H](O)CNC(=O)[C@@H]2N[C@H](SC2(C)C)[C@H](NC(=O)Cc3ccccc3)C(=O)NCc4ccccc4)[C@H](NC(=O)Cc5ccccc5)C(=O)NCc6ccccc6 | Protease |
| CHEMBL12844 | CC(C)c1ccccc1SC2=C(O)C=C(OC2=O)c3ccccc3 | Protease |
| CHEMBL140653 | COc1ccc(cc1)S(=O)(=O)N(CC(C)C)C[C@@H](O)[C@H](Cc2ccccc2)NC(=O)c3ccccc3C(F)(F)F | Protease |
| CHEMBL2062145 | CC(C)(C)OC(=O)N[C@@H](Cc1ccccc1)[C@@H](O)C[C@@]2(Cc3ccccc3)CCN([C@@H]4[C@H](O)Cc5ccccc45)C2=O | Protease |
| CHEMBL325778 | CC(C)CN(C[C@H](O)[C@H](Cc1ccccc1)NC(=O)[C@H](CC(=O)N)NC(=O)c2ccc3ccccc3n2)NC(=O)C(NC(=O)OCc4ccccc4)C(C)C | Protease |
| CHEMBL122480 | CC(C)c1ccc(C)cc1SC2=C(O)OC(CC2=O)(c3ccccc3)c4ccccc4 | Protease |
| CHEMBL949 | CC(C)(C)NC(=O)[C@@H]1CNCCN1C[C@@H](O)C[C@@H](Cc2ccccc2)C(=O)N[C@@H]3[C@H](O)Cc4ccccc34 | Protease |
| CHEMBL325731 | CC(C)[C@@H](NS(=O)(=O)N(C)Cc1ccccn1)C(=O)N[C@@H](Cc2ccccc2)[C@@H](O)[C@@H](O)[C@H](Cc3ccccc3)NC(=O)[C@@H](NS(=O)(=O)N(C)Cc4ccccn4)C(C)C | Protease |
| CHEMBL12421 | CC(C)c1ccccc1SC2=C(O)C=C(OC2=O)c3cccc(c3)c4ccccc4 | Protease |
| CHEMBL430701 | CC(C)(C)NC(=O)[C@@H]1CNCCN1C[C@@H](O)C[C@@H](Cc2ccccc2)C(=O)NC3[C@H](O)Cc4ccccc34 | Protease |
| CHEMBL2367850 | CC(C)(C)OC(=O)N[C@@H](Cc1ccccc1)[C@@H](O)C[C@@H](Cc2ccccc2)C(=O)N[C@H](CO)c3ccccc3 | Protease |
| CHEMBL324948 | CC(C)(C)NC(=O)[C@@H]1C[C@H](Cl)CN1C(=O)[C@@H](O)[C@H](Cc2ccccc2)NC(=O)c3ccccc3N | Protease |
| CHEMBL107310 | CC1(C)S[C@@H](N[C@H]1C(=O)NCCNC(=O)[C@@H]2N[C@H](SC2(C)C)[C@H](NC(=O)Cc3ccccc3)C(=O)NCC(=O)O)[C@H](NC(=O)Cc4ccccc4)C(=O)NCC(=O)O | Protease |
| CHEMBL306810 | CC(C)(C)NC(=O)[C@@H]1C[C@@H]2CCCC[C@@H]2CN1C[C@@H](O)[C@H](Cc3ccccc3)NC(=O)[C@@H](NC(=O)c4ccc5ccccc5n4)[C@@H]6CCS(=O)(=O)C6 | Protease |
| CHEMBL386631 | CC1(C)S[C@@H](N[C@H]1C(=O)NCCNC(=O)[C@@H]2N[C@H](SC2(C)C)[C@H](NC(=O)c3ccccc3c4ccccc4)C(=O)NCC(=O)O)[C@H](NC(=O)c5ccccc5c6ccccc6)C(=O)NCC(=O)O | Protease |
| CHEMBL68267 | CCCC(CCC)NC(=O)C[C@@H](C(=O)NC[C@@H](O)[C@H](Cc1ccccc1)NC(=O)OCc2ccccc2)C(C)(C)C | Protease |
| CHEMBL141002 | COc1ccc(cc1)S(=O)(=O)N(CC(C)C)C[C@@H](O)[C@H](Cc2ccccc2)NC(=O)c3cc(O)ccc3C | Protease |
| CHEMBL125719 | Cc1ccc(c(SC2=C(O)OC(CCc3ccccc3)(CC2=O)c4ccc(O)cc4)c1)C(C)(C)C | Protease |
| CHEMBL45838 | CC(C)(C)OC(=O)N[C@@H](Cc1ccccc1)[C@H](O)CNC[C@@H](O)[C@H](Cc2ccccc2)NC(=O)C(O)C(F)(F)F | Protease |
| CHEMBL301046 | CN(C)C(=O)OCCOc1ccc(C[C@H](NC(=O)OC(C)(C)C)[C@H](O)CNC[C@@H](O)[C@H](Cc2ccccc2)NC(=O)OC(C)(C)C)cc1 | Protease |
| CHEMBL2112565 | CCC[C@@]1(CCc2ccccc2)CC(=O)C(=C(O)O1)[C@H](CC)c3cccc(NS(=O)(=O)c4ccc(cn4)C#N)c3 | Protease |
| CHEMBL2092977 | CCC[C@]1(CCc2ccccc2)CC(=O)C(=C(O)O1)[C@H](CC)c3cccc(NS(=O)(=O)c4ccc(cn4)C#N)c3 | Protease |
| CHEMBL2374443 | COC(=O)N[C@H](CCc1ccccc1)C(=O)NCCCC[C@@H](CO)N(CC(C)C)S(=O)(=O)c2ccc(N)cc2 | Protease |
| CHEMBL288759 | CC(C)(C)OC(=O)N[C@@H](Cc1ccccc1)[C@H](O)CNC[C@@H](O)[C@H](Cc2ccccc2)NC(=O)[C@@](C)(O)C(C)(C)C | Protease |
| CHEMBL326679 | CC1(C)S[C@@H](N[C@H]1C(=O)NCCNC(=O)[C@@H]2N[C@H](SC2(C)C)[C@H](NC(=O)Cc3ccccc3)C(=O)NCCO)[C@H](NC(=O)Cc4ccccc4)C(=O)NCCO | Protease |
| CHEMBL345868 | OC(C[C@@H](Cc1ccccc1)C(=O)N[C@H]2CCCC[C@@H]2NC(=O)c3ccc4ccccc4n3)C[C@@H](Cc5ccccc5)C(=O)N[C@H]6CCCC[C@@H]6NC(=O)c7ccc8ccccc8n7 | Protease |
| CHEMBL9618 | CC(C)(C)OC(=O)N[C@@H](Cc1ccccc1)[C@@H](O)[C@H](O)[C@H](Cc2ccccc2)NC(=O)OC(C)(C)C | Protease |
| CHEMBL9726 | CC(C)(C)OC(=O)N[C@@H](Cc1ccccc1)[C@H](O)[C@@H](O)[C@H](Cc2ccccc2)NC(=O)OC(C)(C)C | Protease |
| CHEMBL280398 | CC(C)[C@@H](COCc1ccccc1)NC(=O)C(F)(F)C(=O)C(Cc2ccc(OCc3ccccc3)cc2)NC(=O)[C@@H](NC(=O)OCc4ccccc4)C(C)C | Protease |
| CHEMBL60782 | CC(C)(C)OC(=O)N[C@@H](Cc1ccccc1)[C@H](O)CNC[C@@H](O)[C@H](Cc2ccc(OCCN3CCOC3=O)cc2)NC(=O)OC(C)(C)C | Protease |
| CHEMBL110348 | CC(C)(C)OC(=O)N[C@@H](Cc1ccccc1)[C@@H](O)C[C@@H](Cc2ccccc2)C(=O)N[C@H]3[C@@H](O)COc4ccccc34 | Protease |
| CHEMBL99949 | CC(C)(C)OC(=O)N[C@@H](Cc1ccccc1)[C@@H](O)C[C@@H](Cc2ccccc2)C(=O)N[C@@H]3[C@@H](O)COc4ccccc34 | Protease |
| CHEMBL108807 | COC(=O)N[C@@H](C(C)C)C(=O)N[C@@H](Cc1ccccc1)C(O)CN(CC(C)C)NC(=O)[C@@H](NC(=O)OC)C(C)C | Protease |
| CHEMBL175214 | CC(C)(C)NC(=O)c1ccccc1OC[C@@H](O)[C@H](Cc2ccccc2)NC(=O)[C@H](CC(=O)N)NC(=O)c3ccc4C(=O)c5ccccc5C(=O)c4c3 | Protease |
| CHEMBL111259 | CC(C)C(NC(=O)OCc1ccccc1)C(=O)NN(C[C@H](O)[C@H](Cc2ccccc2)NC(=O)[C@H](CC(=O)N)NC(=O)c3ccc4ccccc4n3)CC5CCCCC5 | Protease |
| CHEMBL418527 | CC(=O)N[C@@H](CSc1ccc2ccccc2c1)C(=O)N[C@@H](Cc3ccccc3)[C@H](O)Cc4ccccc4C(=O)NC(C)(C)C | Protease |
| CHEMBL108102 | COC(=O)N[C@@H](C(C)C)C(=O)N[C@@H](Cc1ccccc1)C(O)CN(Cc2ccc(F)cc2)NC(=O)[C@@H](NC(=O)OC)C(C)C | Protease |
| CHEMBL1790662 | CC[C@H](C)[C@H](NC(=O)CN(CC(Br)CBr)C(=O)[C@H](Cc1ccccc1)NC(=O)OC(C)(C)C)C(=O)N[C@@H](C(C)C)C(=O)OC | Protease |
| CHEMBL113554 | CC(C)[C@@H](NC(=O)N(C)Cc1ccccn1)C(=O)N[C@@H](Cc2ccccc2)C(O)[C@H](Cc3ccccc3)NC(=O)[C@@H](NC(=O)N(C)Cc4ccccn4)C(C)C | Protease |
| CHEMBL322964 | CC(C)[C@@H](NC(=O)N1CCOCC1)C(=O)N[C@@H](Cc2ccccc2)[C@@H](O)[C@@H](O)[C@H](Cc3ccccc3)NC(=O)[C@@H](NC(=O)N4CCOCC4)C(C)C | Protease |
| CHEMBL355811 | CC(C)(C)NC(=O)c1ccccc1OC[C@@H](O)[C@H](Cc2ccccc2)NC(=O)[C@H](CC(=O)N)NC(=O)c3ccc4C(=O)c5ccccc5S(=O)(=O)c4c3 | Protease |
| CHEMBL10777 | CC(C)(C)NC(=O)c1ccccc1C[C@@H](O)[C@H](Cc2ccccc2)NC(=O)[C@H](CSc3ccccc3)NS(=O)(=O)C | Protease |
| CHEMBL348047 | OC(C[C@@H](Cc1ccccc1)C(=O)N[C@H]2CCCC[C@@H]2NC(=O)c3cc4ccccc4cn3)C[C@@H](Cc5ccccc5)C(=O)N[C@H]6CCCC[C@@H]6NC(=O)c7cc8ccccc8cn7 | Protease |
| CHEMBL442738 | CC(C)CCN(C[C@@H](O)[C@@H]1Cc2ccc(OCCCCCC(=O)N[C@@H](C(C)C)C(=O)N1)cc2)S(=O)(=O)c3ccc(N)cc3 | Protease |
| CHEMBL292161 | COc1cc(Oc2ccccc2)ccc1C[C@@H](O)C[C@@H](Cc3ccccc3)C(=O)N[C@@H]4[C@H](O)Cc5ccccc45 | Protease |
| CHEMBL276704 | CC(=O)N[C@@H](CSCc1ccccc1)C(=O)N[C@@H](Cc2ccccc2)[C@H](O)Cc3ccccc3C(=O)NC(C)(C)C | Protease |
| CHEMBL97189 | CC(C)CN(C[C@H](O)[C@H](Cc1ccccc1)NC(=O)[C@@H](NC(=O)C)C(C)C)NC(=O)[C@@H](NC(=O)C)C(C)C | Protease |
| CHEMBL368352 | CC(C)(C)NC(=O)c1ccccc1OC[C@@H](O)[C@H](Cc2ccccc2)NC(=O)[C@H](CC(=O)N)NC(=O)c3ccc4ccccc4n3 | Protease |
| CHEMBL279372 | CCCC[C@@H]1[C@@H](CCS1(=O)=O)OC(=O)N[C@@H](Cc2ccccc2)[C@H](O)CN3C[C@H]4CCCC[C@H]4C[C@H]3C(=O)NC(C)(C)C | Protease |
| CHEMBL122547 | CC(C)c1ccc(c(SC2=C(O)OC(CCc3ccccc3)(CC2=O)c4ccccc4)c1)C(C)(C)C | Protease |
| CHEMBL217396 | CN(C)c1ccc(CNC(=O)[C@@H](NC(=O)Cc2ccccc2)[C@@H]3N[C@@H](C(=O)NCCNC(=O)[C@@H]4N[C@H](SC4(C)C)[C@H](NC(=O)Cc5ccccc5)C(=O)NCc6ccc(cc6)N(C)C)C(C)(C)S3)cc1 | Protease |
| CHEMBL330739 | CC(C)[C@@H]1[C@@H](CCS1(=O)=O)OC(=O)N[C@@H](Cc2ccccc2)[C@H](O)CN3CCN(CC(C)(C)C)C[C@H]3C(=O)NC(C)(C)C | Protease |
| CHEMBL103217 | COC(=O)c1c(Br)c(OC)c2OCOc2c1c3c4OCOc4c(OC)c(Br)c3C(=O)OC | Protease |
| CHEMBL99600 | CC(C)(C)NC(=O)[C@@H]1C[C@@H]2CCCC[C@@H]2CN1C[C@@H](O)[C@H](Cc3ccccc3)NC(=O)O[C@H]4COCC=C4 | Protease |
| CHEMBL139309 | COc1ccc(cc1)S(=O)(=O)N(CC(C)C)C[C@@H](O)[C@H](Cc2ccccc2)NC(=O)c3ccccc3C(C)C | Protease |
| CHEMBL325815 | COc1ccc(CN(C[C@H](O)[C@H](Cc2ccccc2)NC(=O)[C@H](CC(=O)N)NC(=O)c3ccc4ccccc4n3)NC(=O)C(NC(=O)OCc5ccccc5)C(C)C)cc1 | Protease |
| CHEMBL341294 | CC(C)(C)c1ccccc1SC2=C(O)OC(CC2=O)(c3ccccc3)c4ccccc4 | Protease |
| CHEMBL53979 | CC(O)COc1ccc(C[C@H](NC(=O)OC(C)(C)C)[C@H](O)CNC[C@@H](O)[C@H](Cc2ccccc2)NC(=O)OC(C)(C)C)cc1 | Protease |
| CHEMBL3085517 | CC[C@H](C)[C@H](NC(=O)[C@@H](C(C)C)C(O)C(O)[C@H](CC1CCCCC1)NC(=O)CC2CCCCC2)C(=O)NCc3ccccn3 | Protease |
| CHEMBL1790518 | CC[C@H](C)[C@H](NC(=O)[C@H](C(C)C)[C@@H](O)[C@H](O)[C@H](CC1CCCCC1)NC(=O)C2CCCCC2)C(=O)NCc3ccccn3 | Protease |
| CHEMBL324285 | Cc1ccccc1C[C@H](NC(=O)c2ccc(C)c(O)c2C)[C@H](O)C(=O)N3C[C@@H](Cl)C[C@H]3C(=O)NC(C)(C)C | Protease |
| CHEMBL14592 | OC1=C(SCc2ccccc2C(=O)OC3CCCC3)C(=O)C=C(O1)c4ccccc4 | Protease |
| CHEMBL48917 | CC(C)(C)OC(=O)N[C@@H](Cc1ccccc1)[C@H](O)CNC[C@@H](O)[C@H](Cc2ccccc2)NC(=O)[C@H](O)C(C)(C)C | Protease |
| CHEMBL356040 | CC1(C)CN=C(N1)c2[nH]c3ccc(Cl)cc3c2S(=O)(=O)c4ccccc4 | Protease |
| CHEMBL312809 | CC(C)[C@H](NC(=O)OCc1ccccc1)C(=O)NC(Cc2ccccc2)C(=O)C(F)(F)C(=O)N3CCc4ccccc4C3 | Protease |
| CHEMBL59667 | COCCCCOc1ccc(C[C@H](NC(=O)OC(C)(C)C)[C@H](O)CNC[C@@H](O)[C@H](Cc2ccccc2)NC(=O)OC(C)(C)C)cc1 | Protease |
| CHEMBL120090 | CC(C)(C)NC(=O)c1ccccc1C[C@@H](O)[C@H](Cc2ccccc2)NC(=O)c3cccc(c3)C(=O)N4CCc5ccccc5C4 | Protease |
| CHEMBL327752 | CC(C)[C@H](NC(=O)C)C(=O)N[C@@H](Cc1ccccc1)[C@@H](O)CN(Cc2ccc(cc2)C#N)NC(=O)[C@@H](NC(=O)C)C(C)C | Protease |
| CHEMBL59706 | CC(C)(C)OC(=O)N[C@@H](Cc1ccccc1)[C@H](O)CNC[C@@H](O)[C@H](Cc2ccc(OCCc3ccccn3)cc2)NC(=O)OC(C)(C)C | Protease |
| CHEMBL69811 | CC(C)[C@H](NC(=O)OCc1ccccc1)C(=O)N[C@@H](Cc2ccccc2)[C@@H](O)CNC(=O)[C@H](CC(=O)NC(C)(C)C)C(C)(C)C | Protease |
| CHEMBL326831 | CC1=C(C(COC(=O)C2=CC2)OC(=O)C3=CC3)c4cc5[nH]c(cc6[nH]c(cc7nc(cc1n4)c(C)c7CCC(=O)O)c(CCC(=O)O)c6C)C(=C5C)C(COC(=O)C8=CC8)OC(=O)C9=CC9 | Protease |
| CHEMBL98709 | CC(C)CN(C[C@@H](O)[C@H](Cc1ccccc1)NC(=O)[C@@H](CSc2ccc3ccccc3c2)NS(=O)(=O)C)C(=O)NC(C)(C)C | Protease |
| CHEMBL420235 | CCC[C@@]1(CCc2ccccc2)OC(=C([C@H](CC)c3cccc(NS(=O)(=O)c4ccc(cn4)C(F)(F)F)c3)C(=O)O1)O | Protease |
| CHEMBL268605 | CC(C)(C)NC(=O)c1ccccc1C[C@@H](O)[C@H](Cc2ccccc2)NC(=O)[C@H](CC(=O)OCc3ccccc3)NC(=O)N | Protease |
| CHEMBL24199 | CC(C)(C)OC(=O)[C@H]1CCCN1CC(O)[C@H](Cc2ccccc2)NC(=O)[C@H](CC(=O)N)NC(=O)OCc3ccccc3 | Protease |
| CHEMBL282054 | CC(C)(C)OC(=O)[C@@H]1CCCN1C[C@H](O)[C@@H](Cc2ccccc2)NC(=O)C(CC(=O)N)NC(=O)OCc3ccccc3 | Protease |
| CHEMBL91062 | CC(C)(C)OC(=O)N[C@H](C[C@H](O)[C@H](Cc1ccccc1)NC(=O)c2cc(O)cc(O)c2)Cc3ccccc3 | Protease |
| CHEMBL323425 | CC(C)C(NC(=O)OCC=C)C(=O)N[C@@H](Cc1ccccc1)[C@H](O)CN(CC2CCCCC2)NC(=O)C(NC(=O)OCC=C)C(C)C | Protease |
| CHEMBL340811 | OC1=C(SCCc2ccccc2)C(=O)CC(CCc3ccccc3)(O1)c4ccccc4 | Protease |
| CHEMBL18031 | C[C@@H]1OCC[C@@H]1OC(=O)N[C@@H](Cc2ccccc2)[C@H](O)CN3C[C@H]4CCCC[C@H]4C[C@H]3C(=O)NC(C)(C)C | Protease |
| CHEMBL106530 | CC(C)[C@H](NC(=O)[C@@H](C[C@H](O)CN1C[C@H]2CCCC[C@H]2C[C@H]1C(=O)NC(C)(C)C)Cc3ccccc3)C(=O)Nc4ccc5ccccc5n4 | Protease |
| CHEMBL316975 | CC1(C)S[C@@H](N[C@H]1C(=O)N[C@H](Cc2ccccc2)C(O)CNC(=O)Cc3ccccc3)[C@H](NC(=O)Cc4ccccc4)C(=O)NCc5ccccc5 | Protease |
| CHEMBL122641 | COC(=O)C(NC(=O)Cc1ccccc1)[C@@H]2N[C@@H](C(=O)NC[C@H](O)CN3C[C@H]4CCCC[C@H]4C[C@H]3C(=O)NC(C)(C)C)C(C)(C)S2 | Protease |
| CHEMBL310485 | CC(C)[C@@H]1[C@@H](CCS1(=O)=O)OC(=O)N[C@@H](Cc2ccccc2)[C@H](O)CN3CCN(Cc4ccc5ccccc5c4)C[C@H]3C(=O)NC(C)(C)C | Protease |
| CHEMBL320637 | CC1(C)S[C@@H](N[C@H]1C(=O)N[C@H](Cc2ccccc2)[C@H](O)CC(=O)NCCCO)[C@H](NC(=O)Cc3ccccc3)C(=O)NCc4ccccc4 | Protease |
| CHEMBL12950 | CC(C)c1ccccc1SC2=C(O)C=C(OC2=O)c3ccc(O)cc3 | Protease |
| CHEMBL354884 | CC(C)(C)NC(=O)[C@@H]1C[C@@H]2CCCC[C@@H]2CN1C[C@@H](O)[C@H](Cc3ccccc3)NC(=O)C4=CCCS(=O)(=O)C4 | Protease |
| CHEMBL107559 | CCOC(=O)NC(C(C)C)C(=O)NN(C[C@@H](O)[C@H](Cc1ccccc1)NC(=O)C(NC(=O)OC)C(C)C)CC2CCCCC2 | Protease |
| CHEMBL331945 | CCC(C)[C@@H]1NC(=O)[C@H](Cc2ccc(OCCCCCNC1=O)cc2)NC[C@@H](O)C[C@@H](Cc3ccccc3)C(=O)N[C@@H]4[C@H](O)Cc5ccccc45 | Protease |
| CHEMBL317773 | COC(=O)c1cc(OC)c2OCOc2c1c3c4OCOc4c(OC)c(Br)c3C(=O)OC | Protease |
| CHEMBL2115250 | CC(C)(C)NC(=O)[C@@H]1CCCC[C@@H]1C[C@@H](O)[C@H](Cc2ccccc2)NC(=O)[C@H](CC(=O)N)NC(=O)OCc3ccccc3 | Protease |
| CHEMBL88755 | CC(C)[C@@H]1[C@@H](CCS1(=O)=O)OC(=O)N[C@@H](Cc2ccccc2)[C@H](O)CN3CCN(C[C@H]3C(=O)NC(C)(C)C)C(C)C | Protease |
| CHEMBL2112601 | CC(C)(C)NC(=O)[C@@H]1CN(CCN1C[C@@H](O)CNC(=O)[C@@H]2N[C@H](SC2(C)C)C(NC(=O)Cc3ccccc3)C(=O)NCc4ccccc4)C(=O)Oc5ccccc5 | Protease |
| CHEMBL107015 | CCNC(=O)[C@@H](NS(=O)(=O)Cc1ccccc1)[C@@H]2N[C@@H](C(=O)NCCNC(=O)[C@@H]3N[C@H](SC3(C)C)[C@H](NS(=O)(=O)Cc4ccccc4)C(=O)NCC)C(C)(C)S2 | Protease |
| CHEMBL170320 | CC(C)CC(SC1CCCC1)C2=C(O)C=C(OC2=O)c3ccccc3 | Protease |
| CHEMBL71741 | CC(C)C(NC(=O)C[C@@H](C(=O)NC[C@@H](O)[C@H](Cc1ccccc1)NC(=O)OCc2ccccc2)C(C)(C)C)C(C)C | Protease |
| CHEMBL2304144 | CC[C@@H](C)[C@H](NC(=O)[C@H](CC1CCCCC1)C(O)C(O)[C@H](CC2CCCCC2)NC(=O)[C@H](Cc3c[nH]cn3)NC(=O)COc4cccc5ccccc45)C(=O)NCc6ccccn6 | Protease |
| CHEMBL12257 | CC(C)c1ccccc1SC2=C(O)C=C(OC2=O)c3ccc(C)cc3 | Protease |
| CHEMBL421622 | OC1=C(SCc2ccccc2)C(=O)CC(CCc3ccccc3)(O1)c4ccccc4 | Protease |
| CHEMBL104525 | COC(=O)[C@@H](NC(=O)Cc1ccccc1)[C@@H]2N[C@@H](C(=O)NCCNC(=O)[C@@H]3N[C@H](SC3(C)C)[C@H](NC(=O)Cc4ccccc4)C(=O)OC)C(C)(C)S2 | Protease |
| CHEMBL61670 | CC(C)(C)OC(=O)N[C@@H](Cc1ccccc1)[C@H](O)CNC[C@@H](O)[C@H](Cc2ccc(OCCOc3cccnc3)cc2)NC(=O)OC(C)(C)C | Protease |
| CHEMBL49305 | CC(C)(C)OC(=O)N[C@@H](Cc1ccccc1)[C@H](O)CNC[C@@H](O)[C@H](Cc2ccccc2)NC(=O)C(O)(CO)C(C)(C)C | Protease |
| CHEMBL34479 | CC(C)C1(CCc2ccc(O)cc2)CC(=O)C(=C(O)O1)Sc3cc(C)c(NC(=O)CC(C)(C)C)cc3C(C)(C)C | Protease |
| CHEMBL262702 | CC(C)[C@H](COCc1ccccc1)NC(=O)C(F)(F)C(=O)C(Cc2ccc(OCc3ccccc3)cc2)NC(=O)[C@H](NC(=O)OCc4ccccc4)C(C)C | Protease |
| CHEMBL312014 | CC(C)CCCC(C)C1CCC2C3CC=C4CC(CC[C@]4(C)C3CC[C@]12C)OC(=O)Nc5cc(cc6cc(cc(O)c56)S(=O)(=O)O)S(=O)(=O)O | Protease |
| CHEMBL10725 | CC(C)(C)NC(=O)c1ccccc1C[C@@H](O)[C@H](Cc2ccccc2)NC(=O)[C@H](CSc3ccc4ccccc4n3)NS(=O)(=O)C | Protease |
| CHEMBL292653 | CN1CCN(CCOc2ccc(C[C@H](NC(=O)OC(C)(C)C)[C@H](O)CNC[C@@H](O)[C@H](Cc3ccccc3)NC(=O)OC(C)(C)C)cc2)C1=O | Protease |
| CHEMBL177011 | CC(C)(C)NC(=O)c1c(OC[C@@H](O)[C@H](Cc2ccccc2)NC(=O)[C@H](CC(=O)N)NC(=O)c3ccc4C(=O)c5ccccc5S(=O)(=O)c4c3)ccc6ccccc16 | Protease |
| CHEMBL262764 | CC(C)(C)OC(=O)N[C@@H](Cc1ccccc1)[C@@H](O)C[C@@H](Cc2ccccc2)C(=O)N[C@@H](Cc3cc4ccccc4[nH]3)C(=O)N | Protease |
| CHEMBL405657 | CC(C)(C)NC(=O)[C@@H]1C[C@@H]2CCC[C@@H]2CN1C[C@@H](O)[C@H](Cc3ccccc3)NC(=O)O[C@H]4CCOC4 | Protease |
| CHEMBL175385 | CC(C)(C)NC(=O)c1c(OC[C@@H](O)[C@H](Cc2ccccc2)NC(=O)[C@H](CC(=O)N)NC(=O)c3ccc4C(=O)c5ccccc5C(=O)c4c3)ccc6ccccc16 | Protease |
| CHEMBL302532 | CC(C)[C@H](NC(=O)OCc1ccccc1)C(=O)N[C@@H](Cc2ccccc2)[C@H](O)CN3CCCC[C@H]3C(=O)NC(C)(C)C | Protease |
| CHEMBL327087 | COCCOC(=O)NC(C(C)C)C(=O)NC(Cc1ccccc1)C(O)CN(CC2CCCCC2)NC(=O)C(NC(=O)OC)C(C)C | Protease |
| CHEMBL273234 | COC(=O)c1cc(ccc1O)C2=CC(=C(Sc3ccccc3C(C)C)C(=O)O2)O | Protease |
| CHEMBL323161 | COC(=O)N[C@@H](C(C)C)C(=O)N[C@@H](Cc1ccccc1)C(O)CN(Cc2ccc(cc2)C#N)NC(=O)[C@@H](NC(=O)OC)C(C)C | Protease |
| CHEMBL297349 | C[C@H](C[C@H](O)[C@H](Cc1ccccc1)NC(=O)OC(C)(C)C)C(=O)N[C@@H]2[C@H](O)Cc3ccccc23 | Protease |
| CHEMBL2304146 | CC[C@@H](C)[C@H](NC(=O)[C@H](C(C)C)C(O)C(O)[C@H](CC(C)C)NC(=O)[C@H](Cc1c[nH]cn1)NC(=O)[C@H](Cc2ccccc2)NC(=O)OC(C)(C)C)C(=O)NCc3ccccn3 | Protease |
| CHEMBL110603 | CC(C)[C@@H](NC(=O)OCc1ccccc1)C(=O)N[C@@H](Cc2ccccc2)[C@H](O)[C@H](Cc3ccccc3)NC(=O)[C@@H](NC(=O)OCC4CCCCN4C)C(C)C | Protease |
| CHEMBL280551 | [O-][S+](Cc1[nH]c2ccccc2c1Sc3ccccc3)c4ccccc4 | Protease |
| CHEMBL415758 | CC(C)(C)NC(=O)c1ccccc1OC[C@@H](O)[C@H](Cc2ccccc2)NC(=O)[C@H](CC(=O)N)NC(=O)c3cnc4ccccc4n3 | Protease |
| CHEMBL61188 | CC(C)(C)OC(=O)N[C@@H](Cc1ccccc1)[C@H](O)CNC[C@@H](O)[C@H](Cc2ccc(OCCO)cc2)NC(=O)OC(C)(C)C | Protease |
| CHEMBL97146 | CC(C)[C@@H](NC(=O)c1ccc2ccccc2n1)C(=O)NN(C[C@H](O)[C@H](Cc3ccccc3)NC(=O)[C@@H](NC(=O)c4ccc5ccccc5n4)C(C)C)Cc6ccccc6 | Protease |
| CHEMBL20416 | CCC(C)C(NC(=O)[C@@H]1CCCN1C[C@H](O)[C@@H](Cc2ccccc2)NC(=O)C(CC(=O)N)NC(=O)[C@H](CC(C)C)NC(=O)C(CO)NC(=O)C)C(=O)NC(C(C)C)C(=O)OC | Protease |
| CHEMBL87806 | CCCC1(CCc2ccccc2)OC(=C(C(CC)c3cccc(NS(=O)(=O)c4ccc(cn4)[N+](=O)[O-])c3)C(=O)O1)O | Protease |
| CHEMBL334628 | COC(=O)C(NC(=O)[C@H](CC(C)C)NC(=O)C1CCCN1C[C@H](O)[C@H](Cc2ccccc2)NC(=O)[C@H](CC(=O)N)NC(=O)[C@H](CC(C)C)NC(=O)[C@H](CO)NC(=O)C)C(C)C | Protease |
| CHEMBL139756 | COC(=O)C1Cc2ccccc2C1NC(=O)[C@@H](C[C@H](O)[C@H](Cc3ccccc3)NC(=O)OC(C)(C)C)Cc4ccccc4 | Protease |
| CHEMBL107369 | COC(=O)[C@@H]1Cc2ccccc2[C@@H]1NC(=O)[C@@H](C[C@H](O)[C@H](Cc3ccccc3)NC(=O)OC(C)(C)C)Cc4ccccc4 | Protease |
| CHEMBL321551 | COC(=O)[C@@H]1Cc2ccccc2[C@H]1NC(=O)[C@@H](C[C@H](O)[C@H](Cc3ccccc3)NC(=O)OC(C)(C)C)Cc4ccccc4 | Protease |
| CHEMBL1790658 | CC[C@H](C)[C@H](NC(=O)CNCC=C)C(=O)N[C@@H](C(C)C)C(=O)OC | Protease |
| CHEMBL320343 | COCCOCCOC(=O)NC(C(C)C)C(=O)N[C@@H](Cc1ccccc1)[C@H](O)CN(CC2CCCCC2)NC(=O)C(NC(=O)OCCOCCOC)C(C)C | Protease |
| CHEMBL92272 | CC(C)(C)OC(=O)N[C@H](C[C@H](O)[C@H](Cc1ccccc1)NC(=O)c2c(Cl)cccc2Cl)Cc3ccccc3 | Protease |
| CHEMBL326025 | COCCOC(=O)NC(C(C)C)C(=O)NN(C[C@@H](O)[C@H](Cc1ccccc1)NC(=O)C(NC(=O)OC)C(C)C)CC2CCCCC2 | Protease |
| CHEMBL275937 | CC(C)c1ccccc1SC2=C(O)C=C(OC2=O)c3cccc(c3)[N+](=O)[O-] | Protease |
| CHEMBL418902 | CC(C)(C)NC(=O)[C@@H]1C[C@@H]2CCCC[C@@H]2CN1C[C@@H](O)[C@H](Cc3ccccc3)NC(=O)[C@@H](NC(=O)c4ccc5ccccc5n4)C6CCOCC6 | Protease |
| CHEMBL275560 | CC(C)c1ccccc1SC2=C(O)C=C(OC2=O)c3ccc(cc3)C(=O)O | Protease |
| CHEMBL308845 | CC(C)[C@@H]1[C@@H](CCS1(=O)=O)OC(=O)N[C@@H](Cc2ccccc2)[C@H](O)CN3CCN(Cc4cc(Cl)cc(Cl)c4)C[C@H]3C(=O)NC(C)(C)C | Protease |
| CHEMBL106805 | CC(C)CNC(=O)C[C@@H](O)[C@@H](Cc1ccccc1)NC(=O)[C@@H]2N[C@H](SC2(C)C)[C@H](NC(=O)Cc3ccccc3)C(=O)NCc4ccccc4 | Protease |
| CHEMBL321789 | CC1(C)S[C@@H](N[C@H]1C(=O)NCCNC(=O)[C@@H]2N[C@H](SC2(C)C)[C@@H](COC(=O)c3ccccc3)NC(=O)Cc4ccccc4)[C@@H](COC(=O)c5ccccc5)NC(=O)Cc6ccccc6 | Protease |
| CHEMBL290749 | CC[C@H](C)[C@H](NC(=O)[C@H](Cc1ccccc1)CC(O)(CO)CN2C[C@H]3CCCC[C@H]3C[C@H]2C(=O)NC(C)(C)C)C(=O)NCc4nc5ccccc5[nH]4 | Protease |
| CHEMBL412878 | CC(C)(C)NC(=O)[C@@H]1C[C@@H]2CCCC[C@@H]2CN1CC(O)C[C@@H](Cc3ccccc3)C(=O)N[C@H]4CCCC[C@@H]4NC(=O)c5cc6ccccc6cn5 | Protease |
| CHEMBL291155 | CC(C)(C)NC(=O)[C@@H]1C[C@@H]2CCCC[C@@H]2CN1CC(O)C[C@@H](Cc3ccccc3)C(=O)N[C@H]4CCCC[C@@H]4NC(=O)c5ccc6ccccc6n5 | Protease |
| CHEMBL285836 | CC(C)CN1CC(O)(CNC(=O)[C@H]2N[C@H](SC2(C)C)[C@H](NC(=O)Cc3ccccc3)C(=O)NCc4ccccc4)C[C@H]1C(=O)NC(C)(C)C | Protease |
| CHEMBL305845 | CC(C)C1(CCc2c[nH]cn2)CC(=O)C(=C(O)O1)Sc3cc(C)c(CO)cc3C(C)(C)C | Protease |
| CHEMBL17419 | C[C@H]1CS(=O)(=O)C[C@H]1OC(=O)N[C@@H](Cc2ccccc2)[C@H](O)CN3C[C@H]4CCCC[C@H]4C[C@H]3C(=O)NC(C)(C)C | Protease |
| CHEMBL81135 | FC(F)(C(=O)NCc1ccccc1)C(=O)C(Cc2ccc(OCc3ccccc3)cc2)NC(=O)C(NC(=O)OCc4ccccc4)C5CCCCC5 | Protease |
| CHEMBL159658 | CC(C)c1ccccc1SC2=C(O)OC(CCCCC(=O)NCCc3ccccc3)(CC2=O)c4ccccc4 | Protease |
| CHEMBL326053 | CC(C)[C@@H](NS(=O)(=O)N(C)Cc1ccccn1)C(=O)N[C@@H](Cc2ccccc2)[C@@H](O)[C@H](O)[C@H](Cc3ccccc3)NC(=O)[C@@H](NS(=O)(=O)N(C)Cc4ccccn4)C(C)C | Protease |
| CHEMBL292276 | COCCOc1ccc(C[C@H](NC(=O)OC(C)(C)C)[C@H](O)CNC[C@@H](O)[C@H](Cc2ccccc2)NC(=O)OC(C)(C)C)cc1 | Protease |
| CHEMBL106282 | CCNC(=O)[C@@H](NC(=O)c1ccccc1Cc2ccccc2)[C@@H]3N[C@@H](C(=O)NCCNC(=O)[C@@H]4N[C@H](SC4(C)C)[C@H](NC(=O)c5ccccc5Cc6ccccc6)C(=O)NCC)C(C)(C)S3 | Protease |
| CHEMBL339426 | Cc1ccccc1SC2=C(O)OC(CCc3ccccc3)(CC2=O)c4ccccc4 | Protease |
| CHEMBL158358 | CC(C)c1nc(CN(C2CC2)C(=O)NCCC(=O)N[C@@H](Cc3ccccc3)[C@@H](O)C[C@H](Cc4ccccc4)NC(=O)OCc5cncs5)cs1 | Protease |
| CHEMBL266620 | CCC(=O)N[C@@H](CC(=O)OCc1ccccc1)C(=O)N[C@@H](Cc2ccccc2)[C@H](O)Cc3ccccc3C(=O)NC(C)(C)C | Protease |
| CHEMBL64513 | Cc1c(SC2=C(O)OC(CCc3ccccc3)(CC2=O)c4ccccc4)sc5ccccc15 | Protease |
| CHEMBL273317 | CC(C)(C)NC(=O)[C@@H]1C[C@@H]2CCCC[C@@H]2CN1C[C@@H](O)[C@H](Cc3ccccc3)NC(=O)O[C@H]4CCS(=O)(=O)C4 | Protease |
| CHEMBL41965 | CCCC[C@H](CC(O)C[C@@H](CCCC)C(=O)N[C@@H](C)C(=O)N[C@@H](C)C(=O)OC)C(=O)N[C@@H](C)C(=O)N[C@@H](C)C(=O)OC | Protease |
| CHEMBL106623 | CC1(C)S[C@@H](N[C@H]1C(=O)NCCNC(=O)[C@@H]2N[C@H](SC2(C)C)[C@H](NC(=O)Cc3ccccc3)C(=O)OCc4ccccc4)[C@H](NC(=O)Cc5ccccc5)C(=O)OCc6ccccc6 | Protease |
| CHEMBL322995 | CCOC(=O)NC(C(C)C)C(=O)N[C@@H](Cc1ccccc1)[C@H](O)CN(CC2CCCCC2)NC(=O)C(NC(=O)OC)C(C)C | Protease |
| CHEMBL12600 | CC(C)c1ccccc1SC2=C(O)C=C(OC2=O)c3cccc(c3)C(F)(F)F | Protease |
| CHEMBL275730 | CC(C)c1ccccc1SC2=C(O)C=C(OC2=O)c3ccc(Cl)cc3 | Protease |
| CHEMBL76338 | CC(C)[C@@H]1[C@@H](CCS1(=O)=O)OC(=O)N[C@@H](Cc2ccccc2)[C@H](O)CN3CCN(Cc4cc(C)cc(C)c4)C[C@H]3C(=O)NC(C)(C)C | Protease |
| CHEMBL170195 | CC(C)(C)NC(=O)[C@@H]1C[C@@H]2CCCC[C@@H]2CN1C[C@@H](O)[C@H](Cc3ccccc3)NC(=O)[C@@H]4CCCSC4 | Protease |
| CHEMBL289928 | CC[C@H](C)[C@H](NC(=O)[C@@H](CC(O)CN1CCC[C@@H]1C(=O)NC(C)(C)C)Cc2ccccc2)C(=O)NCc3nc4ccccc4[nH]3 | Protease |
| CHEMBL9346 | CCC(C)[C@H](NC(=O)[C@@H]1CCCN1P(=O)(O)C(Cc2ccccc2)NC(=O)[C@H](CC(=O)N)NC(=O)OC(C)(C)C)C(=O)NCC(C)C | Protease |
| CHEMBL436034 | CC(C)(C)NC(=O)c1ccccc1C[C@@H](O)[C@H](CSc2ccc3ccccc3c2)NC(=O)CCC(=O)N | Protease |
| CHEMBL327025 | CC(C)(C)NC(=O)[C@@H]1CCCCN1C[C@@H](O)C[C@@H](Cc2ccccc2)C(=O)NC3[C@H](O)Cc4ccccc34 | Protease |
| CHEMBL293777 | CC(C)(C)NC(=O)[C@@H]1CCCCN1C[C@@H](O)C[C@@H](Cc2ccccc2)C(=O)N[C@@H]3[C@H](O)Cc4ccccc34 | Protease |
| CHEMBL171148 | O[C@@H]1[C@@H](O)[C@@H](COc2ccccc2)N(Cc3ccccc3)C(=O)N(Cc4ccccc4)[C@@H]1COc5ccccc5 | Protease |
| CHEMBL293727 | CC(C)(C)OC(=O)N[C@@H](Cc1ccccc1)[C@H](O)CNC[C@@H](O)[C@H](Cc2ccc(OCCCN3CCOCC3=O)cc2)NC(=O)OC(C)(C)C | Protease |
| CHEMBL13067 | COC(=O)c1cccc(c1)C2=CC(=C(Sc3ccccc3C(C)C)C(=O)O2)O | Protease |
| CHEMBL57069 | COc1ccccc1C[C@@H](O)C[C@@H](Cc2ccccc2)C(=O)N[C@@H]3[C@H](O)Cc4ccccc34 | Protease |
| CHEMBL1790517 | CC[C@H](C)[C@H](NC(=O)[C@H](C(C)C)[C@@H](O)[C@H](O)[C@H](CC1CCCCC1)NC(=O)c2ccccc2)C(=O)NCc3nc4ccccc4[nH]3 | Protease |
| CHEMBL159602 | CC(C)c1ccccc1SC2=C(O)OC(CCCC(=O)NCc3ccccc3)(CC2=O)c4ccccc4 | Protease |
| CHEMBL2111802 | CC(C)(C)NC(=O)[C@H]1CN(CCN1C[C@@H](O)CNC(=O)[C@@H]2N[C@H](SC2(C)C)C(NC(=O)Cc3ccccc3)C(=O)NCc4ccccc4)C(=O)Oc5ccccc5 | Protease |
| CHEMBL429193 | C[C@@H]1C[C@@H](CS1(=O)=O)OC(=O)N[C@@H](Cc2ccccc2)[C@H](O)CN3C[C@H]4CCCC[C@H]4C[C@H]3C(=O)NC(C)(C)C | Protease |
| CHEMBL104375 | CC1(C)S[C@@H](N[C@H]1C(=O)N[C@H](Cc2ccccc2)[C@H](O)CC(=O)N)[C@H](NC(=O)Cc3ccccc3)C(=O)NCc4ccccc4 | Protease |
| CHEMBL440834 | COc1ccc(cc1)C2=CC(=C(Sc3ccccc3C(C)C)C(=O)O2)O | Protease |
| CHEMBL132945 | CC(C)(C)NC(=O)[C@@H]1C[C@@H]2CCCC[C@@H]2CN1C(=O)[C@@H](O)[C@H](Cc3ccccc3)NC(=O)[C@H](CC(=O)N)NC(=O)OCc4ccccc4 | Protease |
| CHEMBL322716 | COC(=O)N[C@@H](C(C)C)C(=O)N[C@@H](Cc1ccccc1)C(O)CN(CC2CCCCC2)NC(=O)[C@@H](NC(=O)OC)C(C)C | Protease |
| CHEMBL340859 | CCCCCC1(CC(=O)C(=C(O)O1)SCCc2ccccc2)c3ccccc3 | Protease |
| CHEMBL170804 | OC1=C(C(CC2CC2)SCc3ccccc3)C(=O)C=C(O1)c4ccccc4 | Protease |
| CHEMBL370663 | O[C@H](C[C@H](CCc1ccc(Cl)cc1Cl)S(=O)(=O)c2ccc(F)cc2)[C@H](Cc3cccc(Br)c3)NC(=O)[C@H]4CCCO4 | Protease |
| CHEMBL371457 | CC(C)C[C@@H](C[C@@H](O)[C@H](Cc1cccc(Br)c1)NC(=O)COc2c(C)cccc2C)S(=O)(=O)c3ccc(F)cc3 | Protease |
| CHEMBL64701 | CC(C)(C)OC(=O)N[C@@H](Cc1ccccc1)[C@H](O)CNC[C@@H](O)[C@H](Cc2ccc(CCC(=O)N3CCOCC3)cc2)NC(=O)OC(C)(C)C | Protease |
| CHEMBL11193 | CC(=O)N[C@@H](CC(=O)OCc1ccccc1)C(=O)N[C@@H](Cc2ccccc2)[C@H](O)Cc3ccccc3C(=O)NC(C)(C)C | Protease |
| CHEMBL330620 | CC(C)CN(C[C@@H](O)[C@H](Cc1ccccc1)NC(=O)[C@@H](CSc2ccc(F)cc2)NS(=O)(=O)C)C(=O)NC(C)(C)C | Protease |
| CHEMBL122031 | CCCCCCCNC(=O)C(NC(=O)Cc1ccccc1)[C@@H]2N[C@@H](C(=O)NCC(O)CN3C[C@H]4CCCC[C@H]4C[C@H]3C(=O)NC(C)(C)C)C(C)(C)S2 | Protease |
| CHEMBL62133 | CC(C)(C)OC(=O)N[C@@H](Cc1ccccc1)[C@H](O)CNC[C@@H](O)[C@H](Cc2ccc(OCCCN3CCOCC3)cc2)NC(=O)OC(C)(C)C | Protease |
| CHEMBL430984 | CC(C)(C)NC(=O)[C@@H]1CC2CCCCC2CN1C[C@@H](O)[C@@H]3Cc4ccc(OCCCCOc5cc6ccccc6cc5C(=O)N[C@@H](CC(=O)N)C(=O)N3)cc4 | Protease |
| CHEMBL164110 | CCCCCC1(CC(=O)C(=C(O)O1)Sc2ccccc2C(C)C)c3ccccc3 | Protease |
| CHEMBL123428 | OC1=C(SCc2ccccc2)C(=O)CC(CC3CCCC3)(O1)c4ccccc4 | Protease |
| CHEMBL12408 | CC(C)c1ccccc1SC2=C(O)C=C(OC2=O)c3cccc(O)c3 | Protease |
| CHEMBL35215 | CCCC[C@H](CC(O)C[C@@H](CCCC)C(=O)N[C@@H](CC(C)C)C(=O)N[C@@H](Cc1ccccc1)C(=O)OC)C(=O)N[C@@H](CC(C)C)C(=O)N[C@@H](Cc2ccccc2)C(=O)OC | Protease |
| CHEMBL304249 | CC(C)(C)OC(=O)N[C@@H](Cc1ccccc1)[C@H](O)CNC[C@@H](O)[C@H](Cc2ccc(OCC(=O)N3CCCCC3)cc2)NC(=O)OC(C)(C)C | Protease |
| CHEMBL304231 | CC(C)(C)OC(=O)N[C@@H](Cc1ccccc1)[C@H](O)CNC[C@@H](O)[C@H](Cc2ccc(OCCn3ccnc3)cc2)NC(=O)OC(C)(C)C | Protease |
| CHEMBL172724 | CC(C)(C)NC(=O)c1ccccc1OC[C@H](O)[C@H](Cc2ccccc2)NC(=O)[C@H](CC(=O)N)NC(=O)c3ccc4C(=O)c5ccccc5C(=O)c4c3 | Protease |
| CHEMBL423628 | CC(C)c1ccccc1SC2=C(O)OC(CCCCC(=O)Nc3ccccc3)(CC2=O)c4ccccc4 | Protease |
| CHEMBL286861 | CC(C)C1(CCc2ccc(O)cc2)CC(=O)C(=C(O)O1)Sc3cc(C)c(NC(=O)OC(C)(C)C)cc3C(C)(C)C | Protease |
| CHEMBL265548 | CC(C)[C@@H]1[C@@H](CCS1(=O)=O)OC(=O)N[C@@H](Cc2ccccc2)[C@H](O)CN3CCN(C[C@H]3C(=O)NC(C)(C)C)C4CCCCCC4 | Protease |
| CHEMBL163941 | CC(C)c1ccccc1SC2=C(O)OC(CCCCC(=O)N)(CC2=O)c3ccccc3 | Protease |
| CHEMBL156424 | CC(C)CCN(C[C@@H](O)[C@H](Cc1ccccc1)NC(=O)OCc2ccccc2)S(=O)(=O)c3cccc(Cl)c3 | Protease |
| CHEMBL331232 | CC(C)(C)NC(=O)C1N(C[C@H](O)CNC(=O)[C@@H]2N[C@H](SC2(C)C)C(NC(=O)Cc3ccccc3)C(=O)NCc4ccccc4)C(C)(C)SC1(C)C | Protease |
| CHEMBL273396 | CCC(C)[C@H](NC(=O)[C@@H]1CCCN1[P@@](=O)(OC)[C@H](Cc2ccccc2)NC(=O)[C@H](CC(=O)N)NC(=O)OC(C)(C)C)C(=O)NCC(C)C | Protease |
| CHEMBL174803 | CC(C)(C)NC(=O)c1ccccc1NC[C@@H](O)[C@H](Cc2ccccc2)NC(=O)[C@H](CC(=O)N)NC(=O)c3ccc4ccccc4n3 | Protease |
| CHEMBL1790230 | CC[C@@H](C)[C@@H]1NC(=O)[C@H](Cc2ccc(OCCCNC1=O)cc2)NC[C@@H](O)[C@H](Cc3ccccc3)NC(=O)[C@@H](NC(=O)c4ccc5ccccc5n4)C(C)C | Protease |
| CHEMBL325764 | CC1(C)S[C@@H](N[C@H]1C(=O)N[C@H](Cc2ccccc2)[C@H](O)CC(=O)NCC(O)CO)[C@H](NC(=O)Cc3ccccc3)C(=O)NCc4ccccc4 | Protease |
| CHEMBL57987 | CSc1ccccc1C[C@@H](O)C[C@@H](Cc2ccccc2)C(=O)N[C@@H]3[C@H](O)Cc4ccccc34 | Protease |
| CHEMBL322171 | CC(C)[C@H](NC(=O)OCc1ccccc1)C(=O)N[C@@H](Cc2ccccc2)[C@@H](O)CN(CC3CCCCC3)NC(=O)[C@@H](NC(=O)OCc4ccccc4)C(C)C | Protease |
| CHEMBL201216 | CC(C)(C)CC(=O)N[C@@H](Cc1ccccc1)[C@@H](O)C[C@]2(Cc3ccccc3)N=CC(=C2O)C4C5OC(=O)N[C@@H]5c6ccccc46 | Protease |
| CHEMBL123513 | CC(C)CCC1(CC(=O)C(=C(O)O1)SCCc2ccccc2)c3ccccc3 | Protease |
| CHEMBL58701 | CC(C)COc1ccccc1C[C@@H](O)C[C@@H](Cc2ccccc2)C(=O)N[C@@H]3[C@H](O)Cc4ccccc34 | Protease |
| CHEMBL124841 | CC(C)CCC1(CC(=O)C(=C(O)O1)SCc2ccccc2)c3ccccc3 | Protease |
| CHEMBL428005 | CCC(C)[C@H](NC(=O)[C@@H]1CCCN1[P@](=O)(OC)[C@H](Cc2ccccc2)NC(=O)[C@H](CC(=O)N)NC(=O)OC(C)(C)C)C(=O)NCC(C)C | Protease |
| CHEMBL93113 | CCC(CC)N1CCN(C[C@@H](O)[C@H](Cc2ccccc2)NC(=O)O[C@@H]3CCS(=O)(=O)[C@@H]3C(C)C)[C@@H](C1)C(=O)NC(C)(C)C | Protease |
| CHEMBL352747 | CC(C)(C)NC(=O)[C@@H]1C[C@@H]2CCCC[C@@H]2CN1C[C@@H](O)[C@H](Cc3ccccc3)NC(=O)[C@H]4CCCSC4 | Protease |
| CHEMBL324206 | CC[C@H](C)[C@H](NC(=O)C1CC2CCCCC2CN1C[C@@H](O)[C@H](Cc3ccccc3)NC(=O)[C@H](CC(=O)N)NC(=O)OCc4ccccc4)C(=O)N[C@@H](C(C)C)C(=O)OC | Protease |
| CHEMBL415082 | CC(C)C(NC(=O)OCc1ccccc1)C(=O)NC(Cc2ccccc2)C(=O)C(F)(F)C(=O)CCc3ccccc3 | Protease |
| CHEMBL294635 | O=C(N[C@@H](Cc1ccccc1)C(=O)N2CCC(=O)C2Sc3ccccc3)c4cccnc4 | Protease |
| CHEMBL321448 | CC(C)(C)NC(=O)C1CC2CCCCC2CN1C[C@H](O)[C@H](Cc3ccccc3)NC(=O)[C@H](CC(=O)N)NC(=O)c4ccc5ccccc5n4 | Protease |
| CHEMBL435594 | CC(C)(C)OC(=O)N[C@@H](Cc1ccccc1)[C@H](O)CNC[C@@H](O)[C@H](Cc2ccccc2)NC(=O)OC3(CO)CCC3 | Protease |
| CHEMBL2369614 | CC[C@H](C)[C@H](NC(=O)CN(CC=C)C(=O)[C@@H](N)Cc1ccccc1)C(=O)N[C@@H](C(C)C)C(=O)OC | Protease |
| CHEMBL321398 | CC(C)(C)NC(=O)C1CC2CCCCC2CN1C[C@H](O)[C@H](Cc3ccccc3)NC(=O)[C@H](CC(=O)N)NC(=O)OCc4ccccc4 | Protease |
| CHEMBL61118 | CC(C)(C)OC(=O)N[C@@H](Cc1ccccc1)[C@H](O)CNC[C@@H](O)[C@H](Cc2ccc(OCCN3CCOCC3)cc2)NC(=O)OC(C)(C)C | Protease |
| CHEMBL281158 | CCC(C)[C@H](NC(=O)[C@H]1CCCN1CC(O)[C@H](Cc2ccccc2)NC(=O)[C@H](CC(=O)N)NC(=O)OCc3ccccc3)C(=O)N[C@@H](C(C)C)C(=O)OC | Protease |
| CHEMBL1790667 | CC[C@H](C)[C@H](NC(=O)CN(CC=C)C(=O)[C@H](Cc1ccccc1)NC(=O)OC(C)(C)C)C(=O)N[C@@H](C(C)C)C(=O)OC | Protease |
| CHEMBL2369615 | CC[C@H](C)[C@H](NC(=O)[C@H](C)N(CC=C)C(=O)[C@H](Cc1ccccc1)NC(=O)[C@H](C)NC(=O)[C@H](C)NC(=O)[C@H](CO)NC(=O)OC(C)(C)C)C(=O)N[C@@H](C(C)C)C(=O)OC | Protease |
| CHEMBL1790653 | CC[C@H](C)[C@H](NC(=O)CN(CC(Cl)CCl)C(=O)[C@H](Cc1ccccc1)NC(=O)[C@H](C)NC(=O)OC(C)(C)C)C(=O)N[C@@H](C(C)C)C(=O)OC | Protease |
| CHEMBL60357 | CC(C)(C)OC(=O)N[C@@H](Cc1ccccc1)[C@H](O)CNC[C@@H](O)[C@H](Cc2ccc(O)cc2)NC(=O)OC(C)(C)C | Protease |
| CHEMBL262044 | CC[C@H](C)[C@H](NC(=O)C1CC2CCCCC2CN1C[C@H](O)[C@H](Cc3ccccc3)NC(=O)[C@H](CC(=O)N)NC(=O)OCc4ccccc4)C(=O)N[C@@H](C(C)C)C(=O)OC | Protease |
| CHEMBL88254 | CC(C)C[C@H](NC(=O)OCc1ccccc1)C(=O)N[C@@H](Cc2ccccc2)C(O)[C@H](Cc3ccccc3)NC(=O)[C@@H](CC(C)C)NC(=O)OCc4ccccc4 | Protease |
| CHEMBL71143 | CC(C)[C@@H]1NC(=O)[C@H](Cc2ccc(OCCCNC1=O)cc2)NC(=O)[C@@H](O)[C@H](Cc3ccccc3)NC(=O)OC(C)(C)C | Protease |
| CHEMBL154252 | CC(C)CCN(C[C@@H](O)[C@H](Cc1ccccc1)NC(=O)[C@H](CC(=O)N)NC(=O)OCc2ccccc2)S(=O)(=O)C | Protease |
| CHEMBL1790654 | CC[C@H](C)[C@H](NC(=O)CN(CC=C)C(=O)OC(C)(C)C)C(=O)N[C@@H](C(C)C)C(=O)OC | Protease |
| CHEMBL59705 | CC(C)(C)OC(=O)N[C@@H](Cc1ccccc1)[C@H](O)CNC[C@@H](O)[C@H](Cc2ccc(OCC(=O)N3CCOCC3)cc2)NC(=O)OC(C)(C)C | Protease |
| CHEMBL367466 | CC(C)(C)NC(=O)c1ccccc1OC[C@H](O)[C@H](Cc2ccccc2)NC(=O)[C@H](CC(=O)N)NC(=O)c3cnc4ccccc4n3 | Protease |
| CHEMBL48805 | CC(C)(C)OC(=O)N[C@@H](Cc1ccccc1)[C@H](O)CNC[C@@H](O)C(Cc2ccc(O)cc2)NC(=O)OC(C)(C)C | Protease |
| CHEMBL1790659 | CC[C@H](C)[C@H](NC(=O)CN(CC=C)C(=O)[C@H](Cc1ccccc1)NC(=O)[C@H](C)N)C(=O)N[C@@H](C(C)C)C(=O)OC | Protease |
| CHEMBL162922 | CC(C)c1ccccc1SC2=C(O)OC(CCCC(=O)N)(CC2=O)c3ccccc3 | Protease |
| CHEMBL57 | Cc1ccnc2N(C3CC3)c4ncccc4C(=O)Nc12 | Protease |
| CHEMBL74862 | CC(C)(C)NC(=O)[C@@H]1CN(Cc2ccccc2O)CCN1C[C@@H](O)[C@H](Cc3ccccc3)NC(=O)O[C@H]4CCOC4 | Protease |
| CHEMBL74629 | CC(C)(C)NC(=O)[C@@H]1CN(Cc2ccccc2)CCN1C[C@@H](O)[C@H](Cc3ccccc3)NC(=O)O[C@H]4CCOC4 | Protease |
| CHEMBL17571 | CC(C)(C)NC(=O)[C@@H]1C[C@@H]2CCCC[C@@H]2CN1C[C@@H](O)[C@H](Cc3ccccc3)NC(=O)OC4CS(=O)(=O)C4 | Protease |
| CHEMBL58606 | COc1ccc(C[C@@H](O)C[C@@H](Cc2ccccc2)C(=O)N[C@@H]3[C@H](O)Cc4ccccc34)c(OC)c1 | Protease |
| CHEMBL343727 | C[C@H](O)C(C)(C)OC(=O)N[C@@H](Cc1ccccc1)[C@H](O)CNC[C@@H](O)[C@H](Cc2ccccc2)NC(=O)OC(C)(C)C | Protease |
| CHEMBL430674 | CC(C)(C)NC(=O)N(C[C@@H](O)[C@H](Cc1ccccc1)NC(=O)[C@H](CC(=O)N)NC(=O)OCc2ccccc2)Cc3ccncc3 | Protease |
| CHEMBL335056 | CC(C)(C)N(C[C@@H](O)[C@H](Cc1ccccc1)NC(=O)[C@H](CC(=O)N)NC(=O)OCc2ccccc2)C(=O)NCc3ccncc3 | Protease |
| CHEMBL305967 | CC(C)(C)NC(=O)N(CC(O)[C@H](Cc1ccccc1)NC(=O)[C@H](CC(=O)N)NC(=O)OCc2ccccc2)Cc3ccncc3 | Protease |
| CHEMBL1790516 | CC[C@H](C)[C@H](NC(=O)[C@H](C(C)C)[C@@H](O)[C@H](O)[C@H](CC1CCCCC1)NC(=O)c2cccc(C)c2OCOc3ccccc3)C(=O)NCc4nc5ccccc5[nH]4 | Protease |
| CHEMBL84217 | Cc1ccc(cc1)S(=O)(=O)c2c([nH]c3ccc(Cl)cc23)C(=O)N | Protease |
| CHEMBL341006 | OC1=C(Sc2ccccc2)C(=O)CC(O1)(c3ccccc3)c4ccccc4 | Protease |
| CHEMBL62046 | CC(C)(C)OC(=O)N[C@@H](Cc1ccccc1)[C@H](O)CNC[C@@H](O)[C@H](Cc2ccc(OCC(O)c3ccccn3)cc2)NC(=O)OC(C)(C)C | Protease |
| CHEMBL277034 | CC(C)(C)OC(=O)N[C@@H](Cc1ccccc1)[C@@H](O)C[C@@H](Cc2ccccc2)C(=O)NCc3ccccc3 | Protease |
| CHEMBL356749 | CC(C)(C)OC(=O)N[C@@H](Cc1ccccc1)[C@@H](O)C[C@]2(Cc3ccccc3)CCN([C@@H]4CSCc5ccccc45)C2=O | Protease |
| CHEMBL285799 | [O-][S+](Cc1[nH]c2ccc(Cl)cc2c1Sc3ccccc3)c4ccccc4 | Protease |
| CHEMBL127754 | CCCCCCC1(CC(=O)C(=C(O)O1)SCCc2ccccc2)c3ccccc3 | Protease |
| CHEMBL12884 | CC(C)c1ccccc1SC2=C(O)C=C(OC2=O)c3cccc(c3)N(C)C | Protease |
| CHEMBL124529 | CC(C)c1ccc(C(C)C)c(SC2=C(O)OC(CC2=O)(c3ccccc3)c4ccccc4)c1 | Protease |
| CHEMBL291506 | CC(C)(C)OC(=O)N[C@@H](Cc1ccccc1)[C@H](O)CNC[C@@H](O)[C@H](Cc2ccc(OCC(C)(C)O)cc2)NC(=O)OC(C)(C)C | Protease |
| CHEMBL12449 | Cc1ccccc1OCC(=O)N[C@@H](Cc2ccccc2)[C@H](O)CN3CC[C@H](C[C@H]3C(=O)NC(C)(C)C)OCc4ccncc4 | Protease |
| CHEMBL122388 | Cc1cc(SC2=C(O)OC(CCc3ccc(O)cc3)(CC2=O)c4ccc(O)cc4)c(cc1CO)C(C)(C)C | Protease |
| CHEMBL333043 | CC(C)(C)NC(=O)[C@@H]1C[C@@H]2CCCC[C@@H]2CN1CC(O)CNC(=O)[C@@H]3N[C@H](SC3(C)C)C(NC(=O)Cc4ccccc4)C(=O)NCCNC(=O)CCCC[C@@H]5SC[C@@H]6NC(=O)N[C@H]56 | Protease |
| CHEMBL45699 | CC(C)(C)OC(=O)N[C@@H](Cc1ccccc1)[C@H](O)CNC[C@@H](O)[C@H](Cc2ccccc2)NC(=O)C(C)(O)C(C)(C)CO | Protease |
| CHEMBL108471 | CC(C)(C)OC(=O)N[C@@H](Cc1ccccc1)[C@@H](O)C[C@@H](Cc2ccccc2)C(=O)N[C@H]3CCCC[C@H]3O | Protease |
| CHEMBL437643 | CC(C)(C)OC(=O)N[C@@H](Cc1ccccc1)[C@@H](O)C[C@@H](Cc2ccccc2)C(=O)N[C@@H]3CCCC[C@H]3O | Protease |
| CHEMBL125097 | CCCCCC1(CC(=O)C(=C(O)O1)SCc2ccccc2)c3ccccc3 | Protease |
| CHEMBL274027 | CC(C)c1ccccc1SC2=C(O)OC(CCCC(=O)N3CCN(CC3)C(=O)OC(C)(C)C)(CC2=O)c4ccccc4 | Protease |
| CHEMBL275301 | CC(C)c1ccccc1SC2=C(O)C=C(OC2=O)c3ccc(F)cc3 | Protease |
| CHEMBL47476 | CC(C)(C)OC(=O)N[C@@H](Cc1ccccc1)[C@H](O)CNC[C@@H](O)[C@H](Cc2ccccc2)NC(=O)OC(C)(C)C | Protease |
| CHEMBL69200 | CC(C)[C@H](NC(=O)OCc1ccccc1)C(=O)N[C@@H](Cc2ccccc2)[C@H](O)CNC(=O)C[C@@H](C(=O)NC(C)(C)C)C(C)(C)C | Protease |
| CHEMBL100034 | CCCCNC(=O)N(CC(C)C)C[C@@H](O)[C@H](Cc1ccccc1)NC(=O)[C@H](CC(=O)N)NC(=O)c2ccc3ccccc3n2 | Protease |
| CHEMBL110557 | CCOC(=O)NC(C(C)C)C(=O)NN(C[C@H](O)[C@H](Cc1ccccc1)NC(=O)[C@H](CC(=O)N)NC(=O)c2ccc3ccccc3n2)CC4CCCCC4 | Protease |
| CHEMBL278875 | CCCCN(C[C@@H](O)[C@H](Cc1ccccc1)NC(=O)[C@H](CC(=O)N)NC(=O)c2ccc3ccccc3n2)C(=O)NCC(C)C | Protease |
| CHEMBL302061 | CCCCNC(=O)N(CC(C)C)CC(O)[C@H](Cc1ccccc1)NC(=O)[C@H](CC(=O)N)NC(=O)c2ccc3ccccc3n2 | Protease |
| CHEMBL309582 | CC(C)[C@@H]1[C@@H](CCS1(=O)=O)OC(=O)N[C@@H](Cc2ccccc2)[C@H](O)CN3CCN(Cc4cccc(O)c4)C[C@H]3C(=O)NC(C)(C)C | Protease |
| CHEMBL338912 | OC1=C(Sc2ccccc2C3CCCCC3)C(=O)CC(O1)(c4ccccc4)c5ccccc5 | Protease |
| CHEMBL317103 | CC(C)(C)NC(=O)[C@@H]1C[C@@H]2CCCC[C@@H]2CN1C[C@@H](O)[C@H](Cc3ccccc3)NC(=O)OCc4ccccc4 | Protease |
| CHEMBL162769 | CC(C)c1ccccc1SC2=C(O)OC(CCCCC(=O)N3CCN(CC3)C(=O)OC(C)(C)C)(CC2=O)c4ccccc4 | Protease |
| CHEMBL439725 | CC(C)(C)OC(=O)N[C@@H](Cc1ccccc1)[C@@H](O)C[C@@H](Cc2ccccc2)C(=O)NC3Cc4ccccc4C3 | Protease |
| CHEMBL85059 | Cc1cc(C)cc(c1)S(=O)(=O)c2c([nH]c3ccc(Cl)cc23)C(=O)N | Protease |
| CHEMBL339288 | OC1=C(Sc2ccccc2)C(=O)CC(CCc3ccccc3)(O1)c4ccccc4 | Protease |
| CHEMBL174527 | CC(C)(C)NC(=O)c1ccccc1OC[C@@H](O)[C@H](Cc2ccccc2)NC(=O)[C@H](CC(=O)N)NC(=O)c3cnnc4ccccc34 | Protease |
| CHEMBL294261 | CC(C)(C)OC(=O)N[C@@H](Cc1ccccc1)[C@H](O)CNC[C@@H](O)[C@H](Cc2ccc3ncccc3c2)NC(=O)OC(C)(C)C | Protease |
| CHEMBL2092978 | CCC[C@]1(CCc2ccccc2)CC(=O)C(=C(O)O1)[C@@H](CC)c3cccc(NS(=O)(=O)c4cn(C)cn4)c3 | Protease |
| CHEMBL41789 | CC[C@H](C)[C@H](NC(=O)[C@@H](CC(O)CN1C[C@H]2CCCC[C@H]2C[C@H]1C(=O)NC(C)(C)C)Cc3ccccc3)C(=O)NCCc4nc5ccccc5[nH]4 | Protease |
| CHEMBL2112562 | CCC[C@@]1(CCc2ccccc2)CC(=O)C(=C(O)O1)[C@H](CC)c3cccc(NS(=O)(=O)c4cn(C)cn4)c3 | Protease |
| CHEMBL21405 | CCCC1(CCc2ccccc2)CC(=O)C(=C(O)O1)C(CC)c3cccc(NS(=O)(=O)c4ccc(cn4)C#N)c3 | Protease |
| CHEMBL2111824 | CC(C)c1ccc(C)cc1SC2=C(O)O[C@](CCc3ccccc3)(CC2=O)c4ccccc4 | Protease |
| CHEMBL355438 | Cc1cc(SC2=C(O)OC(CCc3ccc(O)cc3)(CC2=O)C4CCCCC4)c(cc1OS(=O)(=O)c5ccccc5)C(C)(C)C | Protease |
| CHEMBL18389 | CC(C)(C)NC(=O)[C@@H]1C[C@@H]2CCCC[C@@H]2CN1C[C@@H](O)[C@H](Cc3ccccc3)NC(=O)O[C@H]4CCOC4 | Protease |
| CHEMBL308837 | CC(C)(C)NC(=O)[C@@H]1CN(CCN1C[C@@H](O)[C@H](Cc2ccccc2)NC(=O)O[C@H]3CCOC3)C(=O)c4ccncc4 | Protease |
| CHEMBL277725 | CC(C)(C)NC(=O)[C@@H]1C[C@@H]2CCCC[C@@H]2CN1C[C@@H](O)[C@H](Cc3ccccc3)NC(=O)O[C@H]4CCSC4 | Protease |
| CHEMBL107701 | CCOC(=O)NC(C(C)C)C(=O)NN(C[C@@H](O)[C@H](Cc1ccccc1)NC(=O)C(NC(=O)OCCOC)C(C)C)CC2CCCCC2 | Protease |
| CHEMBL273732 | CC(C)c1ccccc1SC2=C(O)C=C(OC2=O)c3ccc(cc3)C(=O)N | Protease |
| CHEMBL126157 | OC1=C(SCc2ccccc2)C(=O)CC(CC3CCCCC3)(O1)c4ccccc4 | Protease |
| CHEMBL337040 | CC(C)(C)OC(=O)[C@@H]1CCCN1CC(O)[C@H](Cc2ccccc2)NC(=O)[C@H](CC(=O)N)NC(=O)OCc3ccccc3 | Protease |
| CHEMBL113062 | CC(C)(C)OC(=O)[C@@H]1CCCN1C[C@@H](O)[C@H](Cc2ccccc2)NC(=O)[C@H](CC(=O)N)NC(=O)OCc3ccccc3 | Protease |
| CHEMBL275389 | CC(C)(C)NC(=O)[C@@H]1C[C@@H]2CCCC[C@@H]2CN1C[C@@H](O)[C@H](Cc3ccccc3)NC(=O)O[C@@H]4CCS(=O)(=O)C4 | Protease |
| CHEMBL63319 | CC(C)(C)OC(=O)N[C@@H](Cc1ccccc1)[C@H](O)CNC[C@@H](O)[C@H](Cc2cccc(OCc3ccccc3)c2)NC(=O)OC(C)(C)C | Protease |
| CHEMBL332684 | CCC[C@]1(CCc2ccccc2)CC(=O)C(=C(O)O1)C(CC)c3cccc(NS(=O)(=O)c4ccc(cn4)C(F)(F)F)c3 | Protease |
| CHEMBL2112563 | CCC[C@]1(CCc2ccccc2)CC(=O)C(=C(O)O1)[C@H](CC)c3cccc(NS(=O)(=O)c4ccc(cn4)C(F)(F)F)c3 | Protease |
| CHEMBL40291 | CC[C@H](C)[C@H](NC(=O)[C@H](Cc1ccccc1)CC(O)(CO)CN(CC2CCCCC2)CC(=O)NC(C)(C)C)C(=O)NCc3nc4ccccc4[nH]3 | Protease |
| CHEMBL75416 | CC(C)(C)OC(=O)C1CCCN1CC(O)[C@H](Cc2ccccc2)NC(=O)[C@H](CC(=O)N)NC(=O)OCc3ccccc3 | Protease |
| CHEMBL325454 | CCCC1(CCc2ccccc2)CC(=O)C(=C(O)O1)C(c3cccc(NS(=O)(=O)c4ccc(cn4)C#N)c3)C(C)(C)C | Protease |
| CHEMBL308693 | CC(C)[C@H](NC(=O)OCc1ccccc1)C(=O)N[C@@H](Cc2ccccc2)[C@H](O)CNC(=O)C3CCCC[C@H]3C(=O)NC(C)(C)C | Protease |
| CHEMBL107250 | CC1(C)S[C@@H](N[C@H]1C(=O)NCCNC(=O)[C@@H]2N[C@H](SC2(C)C)[C@@H](CO)NC(=O)Cc3ccccc3)[C@@H](CO)NC(=O)Cc4ccccc4 | Protease |
| CHEMBL2367852 | C[C@@H](NC(=O)[C@@H](C[C@H](O)[C@H](Cc1ccccc1)NC(=O)OC(C)(C)C)Cc2ccccc2)c3ccccc3 | Protease |
| CHEMBL91557 | CC(C)(C)OC(=O)N[C@H](C[C@H](O)[C@H](Cc1ccccc1)NC(=O)c2ccccc2O)Cc3ccccc3 | Protease |
| CHEMBL163390 | CC(C)c1ccccc1SC2=C(O)OC(CCCC(=O)NCCc3ccccc3)(CC2=O)c4ccccc4 | Protease |
| CHEMBL174342 | OC1=C(C(CC2CC2)SC3CCCCC3)C(=O)C=C(O1)c4ccccc4 | Protease |
| CHEMBL13036 | CCOC(=O)c1cc(ccc1O)C2=CC(=C(Sc3ccccc3C(C)C)C(=O)O2)O | Protease |
| CHEMBL28462 | CC(C)(C)NC(=O)c1cc2ccccc2cc1C[C@@H](O)[C@H](Cc3ccccc3)NC(=O)[C@H](CC(=O)N)NC(=O)c4ccc5ccccc5n4 | Protease |
| CHEMBL321429 | CCOC(=O)NC(C(C)C)C(=O)N[C@@H](Cc1ccccc1)[C@H](O)CN(CC2CCCCC2)NC(=O)C(NC(=O)OCCOC)C(C)C | Protease |
| CHEMBL318814 | CC1(C)S[C@@H](N[C@H]1C(=O)N[C@@H](CO)Cc2ccccc2)[C@H](NC(=O)Cc3ccccc3)C(=O)NCc4ccccc4 | Protease |
| CHEMBL421230 | CCCC1(CCc2ccccc2)CC(=O)C(=C(O)O1)C(c3cccc(NS(=O)(=O)c4cn(C)cn4)c3)C(C)(C)C | Protease |
| CHEMBL163275 | CC(C)(C)NC(=O)c1ccccc1C[C@@H](O)[C@H](CSc2ccccc2)NC(=O)CCC(=O)N | Protease |
| CHEMBL43739 | CC[C@H](C)[C@H](NC(=O)[C@@H](CC(O)CN1C[C@H]2CCCC[C@H]2C[C@H]1C(=O)NC(C)(C)C)Cc3ccccc3)C(=O)NCc4ccccn4 | Protease |
| CHEMBL293525 | CC(C)(C)OC(=O)N[C@@H](Cc1ccccc1)[C@H](O)CNC[C@@H](O)[C@H](Cc2ccc(OCC(=O)O)cc2)NC(=O)OC(C)(C)C | Protease |
| CHEMBL13037 | CC(C)c1ccccc1SC2=C(O)C=C(OC2=O)c3cccc(c3)C(=O)O | Protease |
| CHEMBL124731 | OC1=C(SCCc2ccccc2)C(=O)CC(O1)(c3ccccc3)c4ccccc4 | Protease |
| CHEMBL74699 | CC(C)(C)NC(=O)[C@@H]1CN(Cc2ccc(O)cc2)CCN1C[C@@H](O)[C@H](Cc3ccccc3)NC(=O)O[C@H]4CCOC4 | Protease |
| CHEMBL419595 | CC(C)CCCC1(CC(=O)C(=C(O)O1)SCCc2ccccc2)c3ccccc3 | Protease |
| CHEMBL24273 | COC(=O)c1[nH]c2ccc(Cl)cc2c1Sc3ccccc3 | Protease |
| CHEMBL140062 | C[C@@H](O)C(C)(C)OC(=O)N[C@@H](Cc1ccccc1)[C@H](O)CNC[C@@H](O)[C@H](Cc2ccccc2)NC(=O)OC(C)(C)C | Protease |
| CHEMBL316766 | CC(C)[C@H](NC(=O)C(O)(c1ccccc1)c2ccccc2)C(=O)N[C@H](C[C@H](O)[C@H](Cc3ccccc3)NC(=O)[C@@H](NC(=O)C(O)(c4ccccc4)c5ccccc5)C(C)C)Cc6ccccc6 | Protease |
| CHEMBL106965 | CCNC(=O)[C@@H](NC(=O)c1ccccc1Oc2ccccc2)[C@@H]3N[C@@H](C(=O)NCCNC(=O)[C@@H]4N[C@H](SC4(C)C)[C@H](NC(=O)c5ccccc5Oc6ccccc6)C(=O)NCC)C(C)(C)S3 | Protease |
| CHEMBL333738 | CC(C)(C)NC(=O)[C@@H]1C[C@@H]2CCCC[C@@H]2CN1C[C@H](O)CNC(=O)[C@@H]3N[C@H](SC3(C)C)C(NC(=O)Cc4ccccc4)C(=O)NCc5ccccc5 | Protease |
| CHEMBL125827 | CC(C)(C)NC(=O)[C@@H]1C[C@@H]2CCCC[C@@H]2CN1CC(O)CNC(=O)[C@@H]3N[C@H](SC3(C)C)C(NC(=O)Cc4ccccc4)C(=O)NCCNC(=O)OC(C)(C)C | Protease |
| CHEMBL2367848 | CC(C)[C@H](NC(=O)[C@@H](C[C@H](O)[C@H](Cc1ccccc1)NC(=O)OC(C)(C)C)Cc2ccccc2)C(=O)O | Protease |
| CHEMBL108429 | COCCOC(=O)NC(C(C)C)C(=O)N[C@@H](Cc1ccccc1)[C@H](O)CN(CC2CCCCC2)NC(=O)C(NC(=O)OCCOC)C(C)C | Protease |
| CHEMBL354379 | CC(C)(C)NC(=O)[C@@H]1C[C@@H]2CCCC[C@@H]2CN1C[C@@H](O)[C@H](Cc3ccccc3)NC(=O)C4=CCCSC4 | Protease |
| CHEMBL18253 | C[C@H]1OCC[C@H]1OC(=O)N[C@@H](Cc2ccccc2)[C@H](O)CN3C[C@H]4CCCC[C@H]4C[C@H]3C(=O)NC(C)(C)C | Protease |
| CHEMBL316959 | CC1(C)S[C@@H](N[C@H]1C(=O)N[C@H](Cc2ccccc2)[C@H](O)CC(=O)N[C@H](CO)c3ccccc3)[C@H](NC(=O)Cc4ccccc4)C(=O)NCc5ccccc5 | Protease |
| CHEMBL276653 | CC(C)(C)NC(=O)c1ccccc1C[C@@H](O)[C@H](Cc2ccccc2)NC(=O)[C@H](CSc3ccc(cc3)C(F)(F)F)NS(=O)(=O)C | Protease |
| CHEMBL318797 | CC1(C)S[C@@H](N[C@H]1C(=O)N[C@H](Cc2ccccc2)[C@H](O)CC(=O)N[C@@H](CO)Cc3ccccc3)[C@H](NC(=O)Cc4ccccc4)C(=O)NCc5ccccc5 | Protease |
| CHEMBL124672 | OC1=C(SCc2ccccc2)C(=O)CC(COc3ccccc3)(O1)c4ccccc4 | Protease |
| CHEMBL72784 | CC(C)(C)NC(=O)[C@@H]1CN(CCCc2ccccc2)CCN1C[C@@H](O)[C@H](Cc3ccccc3)NC(=O)O[C@H]4CCOC4 | Protease |
| CHEMBL432252 | CCOC(=O)NC(C(C)C)C(=O)N[C@@H](Cc1ccccc1)[C@H](O)CN(CC2CCCCC2)NC(=O)C(NC(=O)OCC)C(C)C | Protease |
| CHEMBL325847 | CC(C)[C@@H](NC(=O)OCCN1CCOCC1)C(=O)N[C@@H](Cc2ccccc2)C(O)[C@H](Cc3ccccc3)NC(=O)[C@@H](NC(=O)OCCN4CCOCC4)C(C)C | Protease |
| CHEMBL125556 | CCCCCCC1(CC(=O)C(=C(O)O1)SCc2ccccc2)c3ccccc3 | Protease |
| CHEMBL123324 | CC(C)CCCC1(CC(=O)C(=C(O)O1)SCc2ccccc2)c3ccccc3 | Protease |
| CHEMBL606565 | CN([C@@H]1[C@H](O)Cc2ccccc12)C(=O)[C@@H](C[C@H](O)[C@H](Cc3ccccc3)NC(=O)OC(C)(C)C)Cc4ccccc4 | Protease |
| CHEMBL163633 | CC(C)c1ccccc1SC2=C(O)OC(CCCC(=O)N3CCOCC3)(CC2=O)c4ccccc4 | Protease |
| CHEMBL122104 | CC(C)(C)NC(=O)C1N(C[C@@H](O)CNC(=O)[C@@H]2N[C@H](SC2(C)C)C(NC(=O)Cc3ccccc3)C(=O)NCc4ccccc4)C(C)(C)SC1(C)C | Protease |
| CHEMBL141398 | COc1ccc(cc1)S(=O)(=O)N(CC(C)C)C[C@@H](O)[C@H](Cc2ccccc2)NC(=O)c3ccccc3OC | Protease |
| CHEMBL15053 | CC(C)(C)NC(=O)c1ccccc1CSC2=C(O)C=C(OC2=O)c3ccccc3 | Protease |
| CHEMBL107744 | CC1(C)S[C@@H](N[C@H]1C(=O)N[C@H](Cc2ccccc2)[C@H](O)CC(=O)N[C@@H](CO)c3ccccc3)[C@H](NC(=O)Cc4ccccc4)C(=O)NCc5ccccc5 | Protease |
| CHEMBL12671 | CC(C)c1ccccc1SC2=C(O)C=C(OC2=O)c3ccc(cc3)[N+](=O)[O-] | Protease |
| CHEMBL354805 | CC(C)(C)NC(=O)[C@@H]1C[C@@H]2CCCC[C@@H]2CN1C[C@@H](O)[C@H](Cc3ccccc3)NC(=O)[C@H]4CCS(=O)(=O)C4 | Protease |
| CHEMBL62390 | Cc1ccsc1SC2=C(O)OC(CCc3ccccc3)(CC2=O)c4ccccc4 | Protease |
| CHEMBL162317 | CC(C)c1ccccc1SC2=C(O)OC(CCCCC(=O)N3CCOCC3)(CC2=O)c4ccccc4 | Protease |
| CHEMBL142990 | CC(C)(C)OC(=O)N[C@@H](Cc1ccccc1)[C@H](O)CNC[C@@H](O)[C@H](Cc2ccccc2)NC(=O)OC(C)(CO)CO | Protease |
| CHEMBL354169 | CC(C)(C)NC(=O)[C@@H]1C[C@@H]2CCCC[C@@H]2CN1C[C@@H](O)[C@H](Cc3ccccc3)NC(=O)[C@@H]4CCS(=O)(=O)C4 | Protease |
| CHEMBL130998 | O[C@@H]1[C@@H](O)[C@@H](COc2ccccc2)N(Cc3ccccc3)S(=O)(=O)N(Cc4ccccc4)[C@@H]1COc5ccccc5 | Protease |
| CHEMBL121005 | OC(C(COCc1ccccc1F)OCc2ccc(F)cc2)C(O)C(COCc3ccccc3F)OCc4ccc(F)cc4 | Protease |
| CHEMBL356345 | CC(C)(C)OC(=O)N[C@@H](Cc1ccccc1)[C@H](O)CNC[C@@H](O)[C@H](Cc2ccccc2)NC(=O)OC(C)(C)CCO | Protease |
| CHEMBL170517 | O[C@@H]1[C@@H](O)[C@@H](CCc2ccccc2)N(Cc3ccccc3)S(=O)(=O)N(Cc4ccccc4)[C@@H]1CCc5ccccc5 | Protease |
| CHEMBL71514 | CC(C)C(NC(=O)OCc1ccccc1)C(=O)N[C@H](CC[C@@H](O)[C@H](Cc2ccccc2)NC(=O)C(NC(=O)OCc3ccccc3)C(C)C)Cc4ccccc4 | Protease |
| CHEMBL292423 | CC(C)(C)OC(=O)N[C@@H](Cc1ccccc1)[C@H](O)CNC[C@@H](O)[C@H](Cc2cccc(O)c2)NC(=O)OC(C)(C)C | Protease |
| CHEMBL1790669 | CC[C@H](C)[C@H](NC(=O)[C@H](C)N(CC=C)C(=O)[C@H](Cc1ccccc1)NC(=O)[C@H](C)NC(=O)OC(C)(C)C)C(=O)N[C@@H](C(C)C)C(=O)OC | Protease |
| CHEMBL189903 | COC(=O)N[C@H](C(=O)N[C@@H](Cc1ccccc1)[C@H](O)CN(OC2CCCC2)S(=O)(=O)c3ccc(OC)cc3)C(C)(C)C | Protease |
| CHEMBL12485 | CC(C)c1ccccc1SC2=C(O)C=C(OC2=O)c3ccc(cc3)C#N | Protease |
| CHEMBL3085516 | CC(C)(C)NC(=O)[C@@H]1CCCN1C[C@H](O)[C@H](Cc2ccccc2)NC(=O)[C@H](CC(=O)N)NC(=O)OCc3ccccc3 | Protease |
| CHEMBL25011 | CC(C)(C)NC(=O)C1CCCN1CC(O)C(Cc2ccccc2)NC(=O)C(CC(=O)N)NC(=O)OCc3ccccc3 | Protease |
| CHEMBL267553 | Cc1ccccc1COC(=O)N[C@@H](Cc2ccccc2)[C@H](O)CN3CC[C@H](C[C@H]3C(=O)NC(C)(C)C)OCc4ccncc4 | Protease |
| CHEMBL155051 | CCCS(=O)(=O)N(CCC(C)C)C[C@@H](O)[C@H](Cc1ccccc1)NC(=O)OCc2ccccc2 | Protease |
| CHEMBL71913 | CC(C)[C@H](NC(=O)[C@H](Cc1ccc(O)cc1)NC(=O)[C@H](O)[C@H](Cc2ccccc2)NC(=O)[C@@H](NC(=O)[C@H](Cc3ccc(O)cc3)NC(=O)C)C(C)C)C(=O)N | Protease |
| CHEMBL105299 | CC(C)(C)OC(=O)N[C@@H](Cc1ccccc1)[C@@H](O)C[C@@H](Cc2ccccc2)C(=O)N[C@]3(C)[C@H](O)Cc4ccccc34 | Protease |
| CHEMBL279323 | C[C@@H]1CS(=O)(=O)C[C@@H]1OC(=O)N[C@@H](Cc2ccccc2)[C@H](O)CN3C[C@H]4CCCC[C@H]4C[C@H]3C(=O)NC(C)(C)C | Protease |
| CHEMBL351243 | CC(C)c1ccccc1SC2=C(O)OC(CCCC(=O)N(C)CCc3ccccc3)(CC2=O)c4ccccc4 | Protease |
| CHEMBL318595 | CC1(C)S[C@@H](N[C@H]1C(=O)N[C@H](Cc2ccccc2)[C@H](O)CC(=O)N[C@H](CO)Cc3ccccc3)[C@H](NC(=O)Cc4ccccc4)C(=O)NCc5ccccc5 | Protease |
| CHEMBL11008 | CC(=O)N[C@@H](CSc1ccccc1)C(=O)N[C@@H](Cc2ccccc2)[C@H](O)Cc3ccccc3C(=O)NC(C)(C)C | Protease |
| CHEMBL279097 | CC(C)(C)NC(=O)[C@@H]1C[C@@H]2CCCC[C@@H]2CN1C[C@@H](O)[C@H](Cc3ccccc3)NC(=O)O[C@@H]4CCSC4 | Protease |
| CHEMBL46067 | CC(C)(C)OC(=O)N[C@@H](Cc1ccccc1)[C@H](O)CNC[C@@H](O)[C@H](Cc2ccccc2)NC(=O)C3(O)COCC3(C)C | Protease |
| CHEMBL273775 | CC(C)[C@H](NC(=O)OCc1ccccc1)C(=O)N[C@@H](Cc2ccccc2)[C@]3(O)CCN(C3)[C@@H](Cc4ccccc4)C(=O)N[C@@H]5[C@H](O)Cc6ccccc56 | Protease |
| CHEMBL353679 | OC1=C(C(SC2CCCC2)C3CCCC3)C(=O)C=C(O1)c4ccccc4 | Protease |
| CHEMBL12707 | CC(C)c1ccccc1SC2=C(O)C=C(OC2=O)c3ccccc3Cl | Protease |
| CHEMBL69604 | CC(C)(C)OC(=O)N[C@@H](Cc1ccccc1)[C@H](O)CN[C@@H](Cc2ccccc2)C(=O)N[C@@H]3[C@H](O)Cc4ccccc34 | Protease |
| CHEMBL2092982 | CC(C)(C)OC(=O)N[C@@H](Cc1ccccc1)[C@@H](O)C[C@@H](Cc2ccccc2)C(=O)N[C@@H]3[C@@H](O)Cc4ccccc34 | Protease |
| CHEMBL143415 | CC(C)(C)OC(=O)N[C@@H](Cc1ccccc1)[C@H](O)CNC[C@@H](O)[C@H](Cc2ccccc2)NC(=O)OCCO | Protease |
| CHEMBL116068 | CCCC1(CCc2ccccc2)CC(=O)C(=C(O)O1)C(c3cccc(NS(=O)(=O)c4ccc(N)cn4)c3)C(C)(C)C | Protease |
| CHEMBL423618 | CC(C)c1ccccc1SC2=C(O)OC(CCCCC(=O)N3CCNCC3)(CC2=O)c4ccccc4 | Protease |
| CHEMBL90645 | CC(C)[C@@H]1[C@@H](CCS1(=O)=O)OC(=O)N[C@@H](Cc2ccccc2)[C@H](O)CN3CCN(C[C@H]3C(=O)NC(C)(C)C)C4CCSCC4 | Protease |
| CHEMBL432178 | OCc1ccc(CN2C(Cc3ccccc3)P(=O)(O)C(Cc4ccccc4)N(Cc5ccc(CO)cc5)C2=O)cc1 | Protease |
| CHEMBL320243 | CC(C)[C@@H](NS(=O)(=O)N(C)Cc1ccccn1)C(=O)N[C@@H](Cc2ccccc2)[C@H](O)[C@H](O)[C@H](Cc3ccccc3)NC(=O)[C@@H](NS(=O)(=O)N(C)Cc4ccccn4)C(C)C | Protease |
| CHEMBL14437 | Cc1cc(cc(C)c1O)C2=CC(=C(SCc3ccccc3)C(=O)O2)O | Protease |
| CHEMBL93389 | CC(C)(C)OC(=O)N[C@H](C[C@H](O)[C@H](Cc1ccccc1)NC(=O)c2cc(N)cc(N)c2)Cc3ccccc3 | Protease |
| CHEMBL372611 | CC(C)(C)CC(=O)N[C@@H](Cc1ccccc1)[C@@H](O)C[C@]2(Cc3ccccc3)N=CC(=C2O)C4C[C@H](CC(=O)N)c5ccccc45 | Protease |
| CHEMBL42366 | CC[C@H](C)[C@H](NC(=O)[C@@H](CC(O)CN1CCCCC1C(=O)NC(C)(C)C)Cc2ccccc2)C(=O)NCc3ccccn3 | Protease |
| CHEMBL319188 | CCCCC(CO)NC(=O)[C@@H]1N[C@H](SC1(C)C)[C@H](NC(=O)Cc2ccccc2)C(=O)NCc3ccccc3 | Protease |
| CHEMBL135948 | CC(C)(C)OC(=O)N[C@@H](Cc1ccccc1)C(O)C[C@@H](Cc2ccccc2)C(=O)N[C@@H]3[C@H](O)Cc4ccccc34 | Protease |
| CHEMBL76460 | CC(C)(C)NC(=O)[C@@H]1CN(CCN1C[C@@H](O)[C@H](Cc2ccccc2)NC(=O)O[C@H]3CCOC3)C(=O)c4ccccn4 | Protease |
| CHEMBL13205 | CC(C)(C)NC(=O)[C@@H]1C[C@@H](CCN1C[C@@H](O)[C@H](Cc2ccccc2)NC(=O)O[C@@H]3CCOC3)OCc4ccncc4 | Protease |
| CHEMBL295472 | OP1(=O)C(Cc2ccccc2)N(Cc3ccc4ccccc4c3)C(=O)N(Cc5ccc6ccccc6c5)C1Cc7ccccc7 | Protease |
| CHEMBL317077 | CC(C)CN(C[C@@H](O)[C@H](Cc1ccccc1)NC(=O)[C@H](CC(=O)N)NC(=O)OCc2ccccc2)C(=O)NC(C)C | Protease |
| CHEMBL445886 | CC(C)(C)OC(=O)N[C@@H](Cc1ccccc1)[C@H](O)CNC[C@@H](O)[C@H](Cc2ccccc2)NC(=O)[C@@](C)(O)c3ccccc3 | Protease |
| CHEMBL14751 | Cc1cc(C)cc(c1)C2=CC(=C(SCc3ccccc3)C(=O)O2)O | Protease |
| CHEMBL171551 | CC(C)CC(SCc1ccccc1)C2=C(O)C=C(OC2=O)c3ccccc3 | Protease |
| CHEMBL71108 | CC(C)CN(CC(O)[C@H](Cc1ccccc1)NC(=O)[C@H](CC(=O)N)NC(=O)OCc2ccccc2)C(=O)NC(C)C | Protease |
| CHEMBL335881 | CC(C)CNC(=O)N(C[C@@H](O)[C@H](Cc1ccccc1)NC(=O)[C@H](CC(=O)N)NC(=O)OCc2ccccc2)C(C)C | Protease |
| CHEMBL12229 | CCc1cccc(c1)C2=CC(=C(Sc3ccccc3C(C)C)C(=O)O2)O | Protease |
| CHEMBL125948 | OC1=C(SCc2ccccc2)C(=O)CC(O1)(c3ccccc3)c4ccccc4 | Protease |
| CHEMBL126166 | OC(=O)CCCC1(CC(=O)C(=C(O)O1)SCCc2ccccc2)c3ccccc3 | Protease |
| CHEMBL123561 | CCCCC1(CC(=O)C(=C(O)O1)SCc2ccccc2)c3ccccc3 | Protease |
| CHEMBL324788 | CC1=C(CCC(=O)O)c2cc3[nH]c(cc4[nH]c(cc5nc(cc1n2)c(C)c5CCOC(=O)C6=CC6)C(=C4CCOC(=O)C7=CC7)C)c(C)c3CCC(=O)O | Protease |
| CHEMBL292691 | COCOc1ccccc1C[C@@H](O)C[C@@H](Cc2ccccc2)C(=O)N[C@@H]3[C@H](O)Cc4ccccc34 | Protease |
| CHEMBL301401 | CC(C)(C)OC(=O)N[C@@H](Cc1ccccc1)[C@H](O)CNC[C@@H](O)[C@H](Cc2ccc(OCCCc3oc4ccccc4n3)cc2)NC(=O)OC(C)(C)C | Protease |
| CHEMBL163435 | CC(C)c1ccccc1SC2=C(O)OC(CCCC(=O)N3CCSCC3)(CC2=O)c4ccccc4 | Protease |
| CHEMBL59022 | NS(=O)(=O)c1ccccc1C[C@@H](O)C[C@@H](Cc2ccccc2)C(=O)N[C@@H]3[C@H](O)Cc4ccccc34 | Protease |
| CHEMBL338010 | CCCC1(CC(=O)C(=C(O)O1)SCc2ccccc2)c3ccccc3 | Protease |
| CHEMBL405969 | CC(C)(C)NC(=O)[C@@H]1C[C@@H]2CCCC[C@@H]2CN1C[C@H](O)[C@H](Cc3ccccc3)NC(=O)[C@H](CC(=O)N)NC(=O)c4ccc5ccccc5n4 | Protease |
| CHEMBL417238 | OC1=C(Sc2cccs2)C(=O)CC(CCc3ccccc3)(O1)c4ccccc4 | Protease |
| CHEMBL12573 | CC(C)c1ccccc1SC2=C(O)C=C(OC2=O)c3ccc(O)c(c3)C(=O)O | Protease |
| CHEMBL308574 | CC(C)(C)NC(=O)[C@@H]1CN(Cc2ccncc2)CCN1C[C@@H](O)[C@H](Cc3ccccc3)NC(=O)O[C@H]4CCOC4 | Protease |
| CHEMBL309507 | CC(C)(C)OC(=O)NC(CC(=O)N)C(=O)N[C@@H](Cc1ccccc1)C(O)CN[C@@H](Cc2ccccc2)C(=O)NC3[C@H](O)Cc4ccccc34 | Protease |
| CHEMBL336981 | CC(C)(C)OC(=O)N[C@@H](CC(=O)N)C(=O)N[C@@H](Cc1ccccc1)C(O)C[C@@H](Cc2ccccc2)C(=O)N[C@@H]3[C@H](O)Cc4ccccc34 | Protease |
| CHEMBL90115 | CCC1(CC=C(C)C)Oc2ccccc2n3cccc3C1=O | Protease |
| CHEMBL2374442 | COC(=O)N[C@@H](C(=O)NCCCC[C@@H](CO)N(CC(C)C)S(=O)(=O)c1ccc(N)cc1)c2ccccc2 | Protease |
| CHEMBL115292 | CC(C)(C)OC(=O)[C@@H]1CCCN1C[C@H](O)[C@H](Cc2ccccc2)NC(=O)[C@H](CC(=O)N)NC(=O)OCc3ccccc3 | Protease |
| CHEMBL377238 | COC(=O)N[C@H](C(c1ccccc1)c2ccccc2)C(=O)NCCCC[C@H](CO)N(CC(C)C)S(=O)(=O)c3ccc(N)cc3 | Protease |
| CHEMBL170802 | CC(C)(C)CC(SC1CCCCC1)C2=C(O)C=C(OC2=O)c3ccccc3 | Protease |
| CHEMBL62196 | CCN(C)C(=O)Oc1nsnc1c2c(Cl)cccc2Cl | Protease |
| CHEMBL431043 | CC1(C)S[C@@H](N[C@H]1C(=O)N[C@H](Cc2ccccc2)[C@H](O)CC(=O)NCCc3ncc[nH]3)[C@H](NC(=O)Cc4ccccc4)C(=O)NCc5ccccc5 | Protease |
| CHEMBL280269 | CC(C)(C)OC(=O)[C@@H]1CCCN1C[C@@H](O)[C@@H](Cc2ccccc2)NC(=O)C(CC(=O)N)NC(=O)OCc3ccccc3 | Protease |
| CHEMBL291802 | CC(C)(C)OC(=O)N[C@@H](Cc1ccccc1)[C@H](O)CNC[C@@H](O)[C@H](Cc2ccc(OCc3ccccc3)cc2)NC(=O)OC(C)(C)C | Protease |
| CHEMBL59816 | CC(C)(C)OC(=O)N[C@@H](Cc1ccccc1)[C@H](O)CNC[C@@H](O)[C@H](Cc2ccccc2O)NC(=O)OC(C)(C)C | Protease |
| CHEMBL321571 | CC(C)(C)NC(=O)[C@@H]1C[C@@H]2CCCC[C@@H]2CN1C[C@@H](O)[C@H](Cc3ccccc3)NC(=O)O[C@@H]4CCCOC4 | Protease |
| CHEMBL293498 | C[C@H]1CN2C(=S)Nc3cc(Cl)cc(CN1CC=C(C)C)c23 | Protease |
| CHEMBL330615 | CC(C)(C)NC(=O)[C@@H]1C[C@@H]2CCCC[C@@H]2CN1C[C@@H](O)[C@H](Cc3ccccc3)NC(=O)O[C@@H]4COCC=C4 | Protease |
| CHEMBL299475 | CS(=O)(=O)c1ccccc1C[C@@H](O)C[C@@H](Cc2ccccc2)C(=O)N[C@@H]3[C@H](O)Cc4ccccc34 | Protease |
| CHEMBL1790512 | CC[C@H](C)[C@H](NC(=O)[C@H](C(C)C)[C@@H](O)[C@H](O)[C@H](CC1CCCCC1)NC(=O)c2ncccc2OCSc3ccccc3)C(=O)NCc4nc5ccccc5[nH]4 | Protease |
| CHEMBL334806 | CC(C)(C)NC(=O)[C@@H]1C[C@@H]2CCCC[C@@H]2CN1C[C@@H](O)[C@H](Cc3ccccc3)NC(=O)[C@H](CC(=O)N)NC(=O)OCc4ccccc4 | Protease |
| CHEMBL1790967 | CC[C@H](C)[C@H](NC(=O)C1C[C@@H]2CCCC[C@@H]2CN1C[C@@H](O)[C@H](Cc3ccccc3)NC(=O)[C@H](CC(=O)N)NC(=O)OCc4ccccc4)C(=O)NC(C(C)C)C(=O)OC | Protease |
| CHEMBL1790971 | CC[C@H](C)[C@H](NC(=O)[C@@H]1CCCN1CC(O)[C@H](Cc2ccccc2)NC(=O)[C@H](CC(=O)N)NC(=O)OCc3ccccc3)C(=O)N[C@@H](C(C)C)C(=O)OC | Protease |
| CHEMBL281922 | CC(C)(C)NC(=O)c1ccccc1C[C@H](O)[C@H](Cc2ccccc2)NC(=O)[C@H](CC(=O)N)NC(=O)c3ccc4ccccc4n3 | Protease |
| CHEMBL267447 | CCCC1=CC(=O)Oc2c3[C@@H](O)[C@H](C)[C@@H](C)Oc3c4C=CC(C)(C)Oc4c12 | Protease |
| CHEMBL413271 | CC(C)(C)OC(=O)N[C@@H](Cc1ccccc1)[C@H](O)CNC[C@@H](O)[C@H](Cc2ccc(OCc3oc4ccccc4n3)cc2)NC(=O)OC(C)(C)C | Protease |
| CHEMBL49773 | CC(C)(C)OC(=O)N[C@@H](Cc1ccccc1)[C@H](O)CNC[C@@H](O)[C@H](Cc2ccccc2)NC(=O)[C@H](O)c3ccccc3 | Protease |
| CHEMBL64802 | CCCCCCc1cc2C(O)C(C(C3=C(O)Oc4cc(O)c(CCCCCC)cc4C3=O)c5ccccc5)C(=O)Oc2cc1O | Protease |
| CHEMBL353836 | CC(C)CC(SC1CCCCC1)C2=C(O)C=C(OC2=O)c3ccccc3 | Protease |
| CHEMBL11142 | CCCC(=O)N[C@@H](CC(=O)OCc1ccccc1)C(=O)N[C@@H](Cc2ccccc2)[C@H](O)Cc3ccccc3C(=O)NC(C)(C)C | Protease |
| CHEMBL120342 | OC(C(COCc1c(F)cccc1F)OCc2ccc(F)cc2)C(O)C(COCc3c(F)cccc3F)OCc4ccc(F)cc4 | Protease |
| CHEMBL98533 | CCNC(=O)N(CC(C)C)C[C@@H](O)[C@H](Cc1ccccc1)NC(=O)[C@H](CC(=O)N)NC(=O)OCc2ccccc2 | Protease |
| CHEMBL332088 | OC(C(COCc1c(F)cccc1F)OCc2ccccc2)C(O)C(COCc3c(F)cccc3F)OCc4ccccc4 | Protease |
| CHEMBL337679 | CCN(C[C@@H](O)[C@H](Cc1ccccc1)NC(=O)[C@H](CC(=O)N)NC(=O)OCc2ccccc2)C(=O)NCC(C)C | Protease |
| CHEMBL430788 | CCNC(=O)N(CC(C)C)CC(O)[C@H](Cc1ccccc1)NC(=O)[C@H](CC(=O)N)NC(=O)OCc2ccccc2 | Protease |
| CHEMBL125597 | NC(=O)CCCCC1(CC(=O)C(=C(O)O1)SCCc2ccccc2)c3ccccc3 | Protease |
| CHEMBL354309 | CC(C)(C)NC(=O)[C@@H]1C[C@@H]2CCCC[C@@H]2CN1C[C@@H](O)[C@H](Cc3ccccc3)NC(=O)[C@@H]4CCSC4 | Protease |
| CHEMBL326288 | CC(C)(C)NC(=O)[C@@H]1CCCN1C[C@@H](O)C[C@@H](Cc2ccccc2)C(=O)NC3[C@H](O)Cc4ccccc34 | Protease |
| CHEMBL156839 | OC(C[C@@H](Cc1ccccc1)C(=O)N[C@H]2CCCC[C@@H]2NC(=O)c3ccncc3)C[C@@H](Cc4ccccc4)C(=O)N[C@H]5CCCC[C@@H]5NC(=O)c6ccncc6 | Protease |
| CHEMBL2369717 | CC[C@H](C)[C@H](NC(=O)[C@H](CCC(=O)N)NC(=O)[C@@H]1CCCN1C(=O)CCCCCCCCCCCCCCC(=O)N[C@@H](CO)C(=O)N[C@@H]([C@@H](C)O)C(=O)N[C@@H](CC(C)C)C(=O)N[C@@H](CC(=O)N)C(=O)N[C@@H](Cc2ccccc2)C(=O)O)C(=O)N[C@@H]([C@H](C)O)C(=O)N[C@@H](CC(C)C)C(=O)N[C@@H](Cc3c[nH]c4ccccc34)C(=O)O | Protease |
| CHEMBL3138267 | CC(C)CCCC(C)[C@H]1CC[C@H]2[C@@H]3CCC4CC(=C(c5cc(Cl)c(O)c(c5)C(=O)O)c6cc(Cl)c(O)c(c6)C(=O)O)CC[C@]4(C)[C@H]3CC[C@]12C | Protease |
| CHEMBL292735 | [K+].[K+].[K+].[K+].CC1(C)CC2(CC(C)(C)c3c(Br)c(OS(=O)(=O)[O-])c(OS(=O)(=O)[O-])c(Br)c23)c4c(Br)c(OS(=O)(=O)[O-])c(OS(=O)(=O)[O-])c(Br)c14 | Protease |
| CHEMBL14859 | OC(=O)c1ccc(cc1)C2=CC(=C(SCc3ccccc3)C(=O)O2)O | Protease |
| CHEMBL94335 | CC(C)[C@@H](NC(=O)Cc1ccccc1)C(=O)NN(C[C@H](O)[C@H](Cc2ccccc2)NC(=O)[C@@H](NC(=O)Cc3ccccc3)C(C)C)Cc4ccccc4 | Protease |
| CHEMBL62158 | OC1=C(Sc2cc3ccccc3s2)C(=O)CC(CCc4ccccc4)(O1)c5ccccc5 | Protease |
| CHEMBL141281 | OC(=O)c1cc(cc(Cl)c1O)C(=CCC23CC4CC(CC(C4)C2)C3)c5cc(Cl)c(O)c(c5)C(=O)O | Protease |
| CHEMBL308771 | CC(C)(C)NC(=O)[C@@H]1CN(CCN1C[C@@H](O)[C@H](Cc2ccccc2)NC(=O)O[C@H]3CCOC3)C(=O)c4cccnc4 | Protease |
| CHEMBL303444 | CC(C)(C)OC(=O)N[C@H](CC[C@H](O)[C@H](Cc1ccccc1)NC(=O)OC(C)(C)C)Cc2ccccc2 | Protease |
| CHEMBL154600 | CC(C)CCN(C[C@@H](O)[C@H](Cc1ccccc1)NC(=O)OCc2ccccc2)S(=O)(=O)c3ccccc3Cl | Protease |
| CHEMBL338554 | Cc1ccccc1SC2=C(O)OC(CC2=O)(c3ccccc3)c4ccccc4 | Protease |
| CHEMBL176742 | CC(C)(C)NC(=O)c1ccccc1SC[C@H](O)[C@H](Cc2ccccc2)NC(=O)[C@H](CC(=O)N)NC(=O)c3ccc4ccccc4c3 | Protease |
| CHEMBL170981 | CC(C)CCC(Sc1ccccc1)C2=C(O)C=C(OC2=O)c3ccccc3 | Protease |
| CHEMBL3138268 | CC(CCC=C(c1cc(Cl)c(O)c(c1)C(=O)O)c2cc(Cl)c(O)c(c2)C(=O)O)[C@H]3CC[C@H]4[C@@H]5CC[C@@H]6CC(F)(F)CC[C@]6(C)[C@H]5CC[C@]34C | Protease |
| CHEMBL320268 | CCNC(=O)[C@@H](NC(=O)c1ccccc1)[C@@H]2N[C@@H](C(=O)NCCNC(=O)[C@@H]3N[C@H](SC3(C)C)[C@H](NC(=O)c4ccccc4)C(=O)NCC)C(C)(C)S2 | Protease |
| CHEMBL318180 | CCNC(=O)[C@@H](NC(=O)c1ccccc1)[C@@H]2N[C@@H](C(=O)NCCNC(=O)C3N[C@H](SC3(C)C)[C@H](NC(=O)c4ccccc4)C(=O)NCC)C(C)(C)S2 | Protease |
| CHEMBL327277 | CC(C)(C)NC(=O)[C@@H]1C[C@@H]2CCCC[C@@H]2CN1C[C@@H](O)[C@H](Cc3ccccc3)NC(=O)O[C@H]4CCCOC4 | Protease |
| CHEMBL9661 | CCC(C)[C@H](NC(=O)[C@@H]1CCCN1[P@](=O)(OC)[C@@H](Cc2ccccc2)NC(=O)[C@H](CC(=O)N)NC(=O)OC(C)(C)C)C(=O)NCC(C)C | Protease |
| CHEMBL12413 | CC(C)(C)NC(=O)[C@@H]1C[C@@H](CCN1C[C@@H](O)[C@H](Cc2ccccc2)NC(=O)OCc3ccccc3)OCc4ccncc4 | Protease |
| CHEMBL122565 | CCCCC1(CC(=O)C(=C(O)O1)SCCc2ccccc2)c3ccccc3 | Protease |
| CHEMBL300492 | CC(C)(C)NC(=O)N[C@@H](Cc1ccccc1)[C@H](O)CNC[C@@H](O)[C@H](Cc2ccccc2)NC(=O)OC(C)(C)C | Protease |
| CHEMBL20303 | CCCC[C@H](CC1(C[C@@H](CCCC)C(=O)N[C@@H](CC(C)C)C(=O)N[C@@H](Cc2ccccc2)C(=O)OC)CO1)C(=O)N[C@@H](CC(C)C)C(=O)N[C@@H](Cc3ccccc3)C(=O)OC | Protease |
| CHEMBL305289 | CC(C)(C)NC(=O)[C@@H]1CCCN1C[C@@H](O)[C@@H]2Cc3ccc(OCCCOc4cc5ccccc5cc4C(=O)N[C@@H](CC(=O)N)C(=O)N2)cc3 | Protease |
| CHEMBL153845 | OC(C[C@@H](Cc1ccccc1)C(=O)N[C@H]2CCCC[C@@H]2NC(=O)c3ccccn3)C[C@@H](Cc4ccccc4)C(=O)N[C@H]5CCCC[C@@H]5NC(=O)c6ccccn6 | Protease |
| CHEMBL299054 | CC(C)(C)OC(=O)N[C@@H](Cc1ccccc1)[C@H](O)CNC[C@@H](O)[C@H](Cc2ccncc2)NC(=O)OC(C)(C)C | Protease |
| CHEMBL2111768 | CCC[C@@]1(CCc2ccccc2)CC(=O)C(=C(O)O1)[C@@H](CC)c3cccc(NS(=O)(=O)c4ccc(cn4)C(F)(F)F)c3 | Protease |
| CHEMBL369369 | CC(C)(C)NC(=O)c1ccccc1OC[C@H](O)[C@H](Cc2ccccc2)NC(=O)[C@H](CC(=O)N)NC(=O)c3cc(O)c4ccccc4n3 | Protease |
| CHEMBL73723 | CC(C)(C)NC(=O)[C@@H]1CN(CCN1C[C@@H](O)[C@H](Cc2ccccc2)NC(=O)O[C@H]3CCOC3)C(=O)c4ccccc4 | Protease |
| CHEMBL115376 | CC[C@H](C)[C@H](NC(=O)[C@@H]1CCCN1CC(O)[C@H](Cc2ccccc2)NC(=O)[C@H](CC(=O)N)NC(=O)[C@H](CC(C)C)NC(=O)[C@H](CO)NC(=O)C)C(=O)OC | Protease |
| CHEMBL344953 | COCC(C)(C)OC(=O)N[C@@H](Cc1ccccc1)[C@H](O)CNC[C@@H](O)[C@H](Cc2ccccc2)NC(=O)OC(C)(C)C | Protease |
| CHEMBL265333 | Cc1cccc(C)c1COC(=O)N[C@@H](Cc2ccccc2)[C@H](O)CN3CC[C@H](C[C@H]3C(=O)NC(C)(C)C)OCc4ccncc4 | Protease |
| CHEMBL368994 | CC(C)(C)NC(=O)c1ccccc1OC[C@H](O)[C@H](Cc2ccccc2)NC(=O)[C@H](CC(=O)N)NS(=O)(=O)c3ccc4ccccc4c3 | Protease |
| CHEMBL159387 | CC(C)c1ccccc1SC2=C(O)OC(CCCC(=O)Nc3ccccc3)(CC2=O)c4ccccc4 | Protease |
| CHEMBL122292 | CC(C)CC1(CC(=O)C(=C(O)O1)SCc2ccccc2)c3ccccc3 | Protease |
| CHEMBL302516 | CC(C)[C@H]([C@@H](O)[C@H](O)[C@H](CC1CCCCC1)NC(=O)c2ccccc2OCSc3ccccc3)C(=O)N[C@H]4Cc5ccccc5[C@H]4O | Protease |
| CHEMBL302486 | CC(C)(C)NC(=O)[C@@H]1CCCN1C[C@@H](O)[C@@H]2Cc3ccc(OCCOc4cc5ccccc5cc4C(=O)N[C@@H](CC(=O)N)C(=O)N2)cc3 | Protease |
| CHEMBL287984 | CC[C@H](C)[C@H](NC(=O)[C@@H](CC(O)C[C@@H](Cc1ccccc1)C(=O)N[C@@H]([C@@H](C)CC)C(=O)NCc2ccccn2)Cc3ccccc3)C(=O)NCc4ccccn4 | Protease |
| CHEMBL154638 | CC(C)CCN(C[C@H](O)[C@H](Cc1ccccc1)NC(=O)OCc2ccccc2)S(=O)(=O)c3ccccc3 | Protease |
| CHEMBL46020 | CC(C)(C)OC(=O)N[C@@H](Cc1ccccc1)[C@H](O)CNC[C@@H](O)[C@H](Cc2ccccc2)NC(=O)C(C)(C)O | Protease |
| CHEMBL331645 | CC(C)(C)NC(=O)[C@@H]1CSCN1CC(O)CNC(=O)[C@@H]2N[C@H](SC2(C)C)C(NC(=O)Cc3ccccc3)C(=O)NCc4ccccc4 | Protease |
| CHEMBL25206 | CC(C)(C)NC(=O)[C@H]1CCCN1C(=O)C(O)[C@H](Cc2ccccc2)NC(=O)OCc3ccccc3 | Protease |
| CHEMBL336818 | CC(C)(C)NC(=O)[C@@H]1CCCN1C(=O)[C@@H](O)[C@H](Cc2ccccc2)NC(=O)OCc3ccccc3 | Protease |
| CHEMBL428686 | CC(C)CCNC(=O)[C@@H](C[C@H](O)[C@H](Cc1ccccc1)NC(=O)OC(C)(C)C)Cc2ccccc2 | Protease |
| CHEMBL73662 | CC(C)(C)NC(=O)[C@@H]1CN(CCc2ccccc2)CCN1C[C@@H](O)[C@H](Cc3ccccc3)NC(=O)O[C@H]4CCOC4 | Protease |
| CHEMBL171802 | OC1=C(C(SCc2ccccc2)c3ccccc3)C(=O)C=C(O1)c4ccccc4 | Protease |
| CHEMBL366779 | OC1=C(C(SC2CCCCC2)c3ccccc3)C(=O)C=C(O1)c4ccccc4 | Protease |
| CHEMBL75270 | CC(C)(C)NC(=O)[C@@H]1CN(Cc2cccc(O)c2)CCN1C[C@@H](O)[C@H](Cc3ccccc3)NC(=O)O[C@H]4CCOC4 | Protease |
| CHEMBL438376 | CC(C)(C)NC(=O)[C@@H]1C[C@@H]2CCCC[C@@H]2CN1C[C@@H](O)[C@@H](Cc3ccccc3)NC(=O)[C@H](CC(=O)N)NC(=O)c4ccc5ccccc5n4 | Protease |
| CHEMBL2111769 | CCC[C@@]1(CCc2ccccc2)CC(=O)C(=C(O)O1)[C@@H](CC)c3cccc(NS(=O)(=O)c4ccc(cn4)C#N)c3 | Protease |
| CHEMBL277060 | OC1=C(SCc2ccccc2)C(=O)C=C(O1)c3ccc4OCOc4c3 | Protease |
| CHEMBL2304142 | CC[C@@H](C)[C@H](NC(=O)[C@@H](CC(C)C)C(O)C(O)[C@H](CC(C)C)NC(=O)C(Cc1c[nH]cn1)NC(=O)COc2cccc3ccccc23)C(=O)NCc4ccccn4 | Protease |
| CHEMBL21188 | CCCC1(CCc2ccccc2)CC(=O)C(=C(O)O1)C(CC)c3cccc(NS(=O)(=O)c4cn(C)cn4)c3 | Protease |
| CHEMBL94271 | CC(C)[C@@H](NC(=O)Cc1cccnc1)C(=O)NN(C[C@H](O)[C@H](Cc2ccccc2)NC(=O)[C@@H](NC(=O)Cc3cccnc3)C(C)C)Cc4ccccc4 | Protease |
| CHEMBL37624 | CC(=O)c1ccc(C)cc1NC(C(=O)N)c2c(Cl)cccc2Cl | Protease |
| CHEMBL163563 | Cc1cc(cc(c1)C(=O)N2CCc3ccccc3C2)C(=O)N[C@@H](Cc4ccccc4)[C@H](O)Cc5ccccc5C(=O)NC(C)(C)C | Protease |
| CHEMBL174480 | CC(C)(C)NC(=O)c1ccccc1OC[C@H](O)[C@H](Cc2ccccc2)NC(=O)[C@H](CC(=O)N)NC(=O)c3cnnc4ccccc34 | Protease |
| CHEMBL319892 | COC(=O)[C@@H](NC(=O)Cc1ccccc1)[C@@H]2N[C@@H](C(=O)NCCNC(=O)[C@@H]3N4[C@H](SC3(C)C)C(NC(=O)Cc5ccccc5)C4=O)C(C)(C)S2 | Protease |
| CHEMBL48891 | CC(C)(C)OC(=O)N[C@@H](Cc1ccccc1)[C@H](O)CNC[C@@H](O)C(CCc2ccccc2)NC(=O)OC(C)(C)C | Protease |
| CHEMBL1790660 | CC[C@H](C)[C@H](NC(=O)CN(CC(Br)CBr)C(=O)[C@H](Cc1ccccc1)NC(=O)[C@H](C)NC(=O)OC(C)(C)C)C(=O)N[C@@H](C(C)C)C(=O)OC | Protease |
| CHEMBL291354 | CC(C)(C)n1c(SC2=C(O)OC(CCc3ccccc3)(CC2=O)c4ccccc4)nc5ccccc15 | Protease |
| CHEMBL333568 | OC1=C(SCCc2ccccc2)C(=O)CC(CC3CCCC3)(O1)c4ccccc4 | Protease |
| CHEMBL99934 | CCCNC(=O)N(CC(C)C)C[C@@H](O)[C@H](Cc1ccccc1)NC(=O)[C@H](CC(=O)N)NC(=O)OCc2ccccc2 | Protease |
| CHEMBL135148 | CCCN(C[C@@H](O)[C@H](Cc1ccccc1)NC(=O)[C@H](CC(=O)N)NC(=O)OCc2ccccc2)C(=O)NCC(C)C | Protease |
| CHEMBL313250 | COc1ccccc1CCCCC2=C(O)Oc3cc(O)ccc3C2=O | Protease |
| CHEMBL436266 | CC1(C)S[C@@H](N[C@H]1C(=O)NCCCCNC(=O)[C@@H]2N[C@H](SC2(C)C)[C@H](NC(=O)Cc3ccccc3)C(=O)NCc4ccccc4)[C@H](NC(=O)Cc5ccccc5)C(=O)NCc6ccccc6 | Protease |
| CHEMBL117389 | CCCC1(CCc2ccccc2)CC(=O)C(=C(O)O1)C(c3cccc(NS(=O)(=O)c4ccc(cn4)C(F)(F)F)c3)C(C)(C)C | Protease |
| CHEMBL584 | Cc1c(O)cccc1C(=O)N[C@@H](CSc2ccccc2)[C@H](O)CN3C[C@H]4CCCC[C@H]4C[C@H]3C(=O)NC(C)(C)C | Protease |
| CHEMBL382924 | CNc1ccc(cc1)S(=O)(=O)[C@@H](CC(C)C)C[C@@H](O)[C@H](Cc2cccc(Br)c2)NC(=O)COc3c(C)cccc3C | Protease |
| CHEMBL302062 | CC(C)[C@H]([C@@H](O)[C@H](O)[C@H](CC1CCCCC1)NC(=O)c2ncccc2OCOc3ccccc3)C(=O)N[C@H]4Cc5ccccc5[C@H]4O | Protease |
| CHEMBL1790514 | CC[C@H](C)[C@H](NC(=O)[C@H](C(C)C)C(O)[C@H](O)[C@H](CC1CCCCC1)NC(=O)c2ncccc2OCOc3ccccc3)C(=O)NCc4nc5ccccc5[nH]4 | Protease |
| CHEMBL71396 | CC(C)[C@H]([C@@H](O)[C@H](O)[C@H](CC1CCCCC1)NC(=O)c2ccccc2OCOc3ccccc3)C(=O)N[C@H]4Cc5ccccc5[C@H]4O | Protease |
| CHEMBL104509 | CC1(C)S[C@@H](N[C@H]1C(=O)NCCCNC(=O)C2N[C@H](SC2(C)C)[C@H](NC(=O)Cc3ccccc3)C(=O)NCc4ccccc4)[C@H](NC(=O)Cc5ccccc5)C(=O)NCc6ccccc6 | Protease |
| CHEMBL318844 | CC1(C)S[C@@H](N[C@H]1C(=O)NCCCNC(=O)[C@@H]2N[C@H](SC2(C)C)[C@H](NC(=O)Cc3ccccc3)C(=O)NCc4ccccc4)[C@H](NC(=O)Cc5ccccc5)C(=O)NCc6ccccc6 | Protease |
| CHEMBL2112468 | CO.CCC(C)C(CO)NC(=O)[C@@H]1N[C@H](SC1(C)C)[C@H](NC(=O)Cc2ccccc2)C(=O)NCc3ccccc3 | Protease |
| CHEMBL2111680 | O.CCC(C)C(CO)NC(=O)[C@@H]1N[C@H](SC1(C)C)[C@H](NC(=O)Cc2ccccc2)C(=O)NCc3ccccc3 | Protease |
| CHEMBL170264 | OC1=C(C(CC2CCCC2)SC3CCCCC3)C(=O)C=C(O1)c4ccccc4 | Protease |
| CHEMBL71266 | CCCNC(=O)N(CC(C)C)CC(O)[C@H](Cc1ccccc1)NC(=O)[C@H](CC(=O)N)NC(=O)OCc2ccccc2 | Protease |
| CHEMBL319126 | CC(C)(C)OC(=O)N[C@@H](Cc1ccccc1)[C@@H](O)C[C@@H](Cc2ccccc2)C(=O)NCC(=O)N | Protease |
| CHEMBL168314 | CC(C)(C)OC(=O)N[C@@H](Cc1ccccc1)[C@@H](O)CC(=O)N[C@@H]2[C@H](O)Cc3ccccc23 | Protease |
| CHEMBL336868 | CC(C)(C)OC(=O)N[C@@H](Cc1ccccc1)[C@@H](O)CC(=O)NC2[C@@H](O)Cc3ccccc23 | Protease |
| CHEMBL422197 | CC(C)c1ccccc1SC2=C(O)OC(CCCC(=O)N3CCNCC3)(CC2=O)c4ccccc4 | Protease |
| CHEMBL320099 | CC(C)(C)OC(=O)N[C@@H](Cc1ccccc1)[C@@H](O)CC(=O)NC2[C@H](O)Cc3ccccc23 | Protease |
| CHEMBL2112566 | CCC[C@]1(CCc2ccccc2)CC(=O)C(=C(O)O1)[C@H](CC)c3cccc(NS(=O)(=O)c4cn(C)cn4)c3 | Protease |
| CHEMBL163539 | CC(C)c1ccccc1SC2=C(O)OC(CCCCC(=O)N3CCSCC3)(CC2=O)c4ccccc4 | Protease |
| CHEMBL107259 | CC(C)(C)OC(=O)N[C@@H](Cc1ccc(O)cc1)[C@@H](O)[C@@H]2CCC[C@H]2C(=O)NCc3ccccc3 | Protease |
| CHEMBL136707 | CC(C)(C)OC(=O)N[C@@H](Cc1ccc(O)cc1)[C@@H](O)C2CCCC2C(=O)NCc3ccccc3 | Protease |
| CHEMBL273367 | CC(C)C(N)C(=O)N[C@@H](Cc1ccccc1)C(O)[C@H](Cc2ccccc2)NC(=O)C(N)C(C)C | Protease |
| CHEMBL335023 | CC(C)(C)NC(=O)[C@@H]1CCCN1C(=O)C(=O)[C@H](Cc2ccccc2)NC(=O)OCc3ccccc3 | Protease |
| CHEMBL117719 | CCCC1(CCC)CC(=O)C(=C(O)O1)C(c2cccc(NS(=O)(=O)c3cn(C)cn3)c2)C(C)(C)C | Protease |
| CHEMBL121287 | OC(C(COCc1ccccc1F)OCc2ccccc2)C(O)C(COCc3ccccc3F)OCc4ccccc4 | Protease |
| CHEMBL345630 | OC(C[C@@H](Cc1ccccc1)C(=O)N[C@H]2CCCC[C@@H]2NC(=O)c3cccnc3)C[C@@H](Cc4ccccc4)C(=O)N[C@H]5CCCC[C@@H]5NC(=O)c6cccnc6 | Protease |
| CHEMBL312178 | CCC(Cc1ccccc1)C2=CC(=C(C(C3CC3)c4cccc(NS(=O)(=O)c5cn(C)cn5)c4)C(=O)O2)O | Protease |
| CHEMBL278499 | OC1=C(SCc2ccccc2)C(=O)C=C(O1)c3cccc(c3)C(F)(F)F | Protease |
| CHEMBL68528 | CC(C)(C)OC(=O)N[C@@H](Cc1ccccc1)[C@]2(O)CCN(C2)[C@@H](Cc3ccccc3)C(=O)N[C@@H]4[C@H](O)Cc5ccccc45 | Protease |
| CHEMBL25116 | CC(C)(C)NC(=O)[C@H]1CCCN1C(=O)C(=O)[C@H](Cc2ccccc2)NC(=O)OCc3ccccc3 | Protease |
| CHEMBL95316 | CC(C)(C)OC(=O)N[C@@H](Cc1ccccc1)[C@@H](O)C[C@@H](Cc2ccccc2)C(=O)N[C@@H](CO)C(=O)N | Protease |
| CHEMBL15217 | CC(C)COC(=O)c1ccccc1CSC2=C(O)C=C(OC2=O)c3ccccc3 | Protease |
| CHEMBL354917 | CC(C)c1ccccc1SC2=C(O)OC(CCCC(=O)N(C)c3ccccc3)(CC2=O)c4ccccc4 | Protease |
| CHEMBL274046 | OC1=C(SCc2ccccc2)C(=O)C=C(O1)c3cc(cc(c3)C(F)(F)F)C(F)(F)F | Protease |
| CHEMBL71646 | CC(C)[C@@H]1NC(=O)[C@H](Cc2ccc(OCCCNC1=O)cc2)NC(=O)[C@H](O)[C@H](Cc3ccccc3)NC(=O)OC(C)(C)C | Protease |
| CHEMBL116965 | CCC(C1=C(O)OC(CCc2ccccc2)(CCc3ccccc3)CC1=O)c4cccc(NS(=O)(=O)c5cn(C)cn5)c4 | Protease |
| CHEMBL124123 | CC(C)(C)NC(=O)[C@@H]1C[C@@H](CN1CC(O)CNC(=O)[C@@H]2N[C@H](SC2(C)C)C(NC(=O)Cc3ccccc3)C(=O)NCc4ccccc4)OCc5ccccc5 | Protease |
| CHEMBL355133 | CC(C)(C)NC(=O)[C@@H]1C[C@@H]2CCCC[C@@H]2CN1C[C@@H](O)[C@H](Cc3ccccc3)NC(=O)[C@H]4CCSC4 | Protease |
| CHEMBL163616 | Cc1ccc(cc1C(=O)N2CCc3ccccc3C2)C(=O)N[C@@H](Cc4ccccc4)[C@H](O)Cc5ccccc5C(=O)NC(C)(C)C | Protease |
| CHEMBL91025 | OC1=C(CCCOc2cccc(c2)C#N)C(=O)Oc3ccccc13 | Protease |
| CHEMBL159290 | CC(C)c1ccccc1SC2=C(O)OC(CCCC(=O)N(C)Cc3ccccc3)(CC2=O)c4ccccc4 | Protease |
| CHEMBL420379 | CC(C)(C)NC(=O)[C@@H]1CN(Cc2cccnc2)CCN1C[C@@H](O)[C@H](Cc3ccccc3)NC(=O)O[C@H]4CCOC4 | Protease |
| CHEMBL2367851 | CC(C)(C)OC(=O)N[C@@H](Cc1ccccc1)[C@@H](O)C[C@@H](Cc2ccccc2)C(=O)N[C@H](CO)Cc3ccccc3 | Protease |
| CHEMBL14692 | OC1=C(SCc2ccccc2)C(=O)C=C(O1)c3ccc(O)cc3 | Protease |
| CHEMBL14759 | OC1=C(SCc2ccccc2)C(=O)C=C(O1)c3cccc(Cl)c3 | Protease |
| CHEMBL99213 | CC(C)(C)NC(=O)[C@@H]1C[C@@H]2CCCC[C@@H]2CN1C[C@@H](O)[C@H](Cc3ccccc3)NC(=O)O[C@@H]4CCOC4 | Protease |
| CHEMBL354897 | NC(=O)OC[C@@H]1CCCOC1 | Protease |
| CHEMBL408667 | CC1=C(C(COC(=O)C2=CCC2)OC(=O)C3=CCC3)c4cc5[nH]c(cc6[nH]c(cc7nc(cc1n4)c(C)c7CCC(=O)O)c(CCC(=O)O)c6C)C(=C5C)C(COC(=O)C8=CCC8)OC(=O)C9=CCC9 | Protease |
| CHEMBL117500 | CCCC1(CCc2ccc(F)cc2)CC(=O)C(=C(O)O1)C(CC)c3cccc(NS(=O)(=O)c4cn(C)cn4)c3 | Protease |
| CHEMBL282382 | CCCC1=CC(=O)Oc2c3[C@@H](O)C(C)[C@H](C)Oc3c4C=CC(C)(C)Oc4c12 | Protease |
| CHEMBL51366 | CC(C)(C)OC(=O)N[C@@H](Cc1ccccc1)[C@H](O)CNC[C@@H](O)[C@H](Cc2ccccc2)NC(=O)SC(C)(C)C | Protease |
| CHEMBL121034 | OC(C(COCc1ccccc1)OCc2ccc(F)cc2)C(O)C(COCc3ccccc3)OCc4ccc(F)cc4 | Protease |
| CHEMBL314409 | OP1(=O)C(Cc2ccccc2)N(Cc3ccc(CBr)cc3)C(=O)N(Cc4ccc(CBr)cc4)C1Cc5ccccc5 | Protease |
| CHEMBL333259 | CCNC(=O)C(NC(=O)Cc1ccccc1)[C@@H]2N[C@@H](C(=O)NCC(O)CN(C)[C@@H](CC3CCCCC3)C(=O)NC(C)(C)C)C(C)(C)S2 | Protease |
| CHEMBL313213 | CCCCCCCCCCCCCCCC(=O)Nc1c(OC(=O)CCCCCCCCCCCCCCC)cc(c2ccccc12)S(=O)(=O)O | Protease |
| CHEMBL371589 | COc1ccc(cc1)S(=O)(=O)N(C[C@@H](O)[C@H](Cc2ccccc2)NC(=O)OC3CCOC3)OC4CCCC4 | Protease |
| CHEMBL14298 | COc1ccc(cc1C)C2=CC(=C(SCc3ccccc3)C(=O)O2)O | Protease |
| CHEMBL286378 | CNC(=O)c1[nH]c2ccccc2c1Sc3ccccc3 | Protease |
| CHEMBL66033 | OC1=C(C(C2=C(O)C(=O)c3ccccc3C2=O)c4ccc(cc4)C(C5=C(O)C(=O)c6ccccc6C5=O)C7=C(O)C(=O)c8ccccc8C7=O)C(=O)c9ccccc9C1=O | Protease |
| CHEMBL94316 | CC(C)(C)OC(=O)N[C@@H](Cc1ccccc1)[C@@H](O)C[C@@H](Cc2ccccc2)C(=O)N[C@@H](Cc3ncc[nH]3)C(=O)N | Protease |
| CHEMBL1791334 | CC[C@H](C)[C@@H](NC(=O)[C@H]([C@H](C)O)N(C)C(=O)CCCCCCCCCCCCCCC(=O)NC(Cc1ccc(O)cc1)C(=O)N[C@@H](Cc2ccccc2)C(=O)O)C(=O)NC(CO)C(=O)N | Protease |
| CHEMBL143401 | CCC(C1=C(O)OC2(CCCCC2)CC1=O)c3ccccc3 | Protease |
| CHEMBL14578 | OC1=C(SCc2ccccc2)C(=O)C=C(O1)c3ccc4OCCOc4c3 | Protease |
| CHEMBL421475 | OC1=C(SCCc2ccccc2)C(=O)CC(O1)(c3ccccc3)c4ccncc4 | Protease |
| CHEMBL383583 | CC(C)C[C@@H](C[C@@H](O)[C@H](Cc1cccc(Br)c1)NC(=O)COc2c(C)cccc2C)S(=O)(=O)c3ccc(cc3)N(C)C | Protease |
| CHEMBL334149 | Cc1ccccc1COC(COCc2ccccc2F)C(O)C(O)C(COCc3ccccc3F)OCc4ccccc4C | Protease |
| CHEMBL48302 | CC(C)(C)OC(=O)NC(CC1CCCCC1)[C@H](O)CNC[C@@H](O)C(CC2CCCCC2)NC(=O)OC(C)(C)C | Protease |
| CHEMBL60314 | CCCN(C)C(=O)Oc1nsnc1c2c(Cl)cccc2Cl | Protease |
| CHEMBL118006 | CCCC1(CCC)CC(=O)C(=C(O)O1)C(CC)c2cccc(NS(=O)(=O)c3cn(C)cn3)c2 | Protease |
| CHEMBL134468 | CC[C@H](C)CNC(=O)[C@H](C(C)C)C(O)C(O)[C@H](CC(C)C)NC(=O)C(Cc1c[nH]cn1)NC(=O)[C@H](Cc2ccccc2)NC(=O)OC(C)(C)C | Protease |
| CHEMBL421909 | CC[C@H](C)CNC(=O)[C@H](C(C)C)C(O)C(O)C(Cc1c[nH]cn1)NC(=O)COc2ccccc2 | Protease |
| CHEMBL447834 | CCNC(=O)[C@@H](NC(=O)Cc1ccc2ccccc2c1)[C@@H]3N[C@@H](C(=O)NCCNC(=O)[C@@H]4N[C@H](SC4(C)C)[C@H](NC(=O)Cc5ccc6ccccc6c5)C(=O)NCC)C(C)(C)S3 | Protease |
| CHEMBL316755 | CN(Cc1ccccc1)C(=O)[C@@H](NC(=O)Cc2ccccc2)[C@@H]3N[C@@H](C(=O)NCCNC(=O)[C@@H]4N[C@H](SC4(C)C)[C@H](NC(=O)Cc5ccccc5)C(=O)N(C)Cc6ccccc6)C(C)(C)S3 | Protease |
| CHEMBL325546 | CC1(C)S[C@@H](N[C@H]1C(=O)NCCNC(=O)[C@@H]2N[C@H](SC2(C)C)[C@H](NC(=O)Cc3ccccc3)C(=O)N(CCO)CCO)[C@H](NC(=O)Cc4ccccc4)C(=O)N(CCO)CCO | Protease |
| CHEMBL133427 | COC(=O)C(NC(=O)[C@H](CC(C)C)NC(=O)C1CCCN1C[C@H](O)[C@H](Cc2ccccc2)NC(=O)C(CC(=O)N)NC(=O)OC(C)(C)C)C(C)C | Protease |
| CHEMBL276962 | CCC(C)C(NC(=O)[C@@H]1CCCN1C[C@H](O)[C@@H](Cc2ccccc2)NC(=O)C(CC(=O)N)NC(=O)OC(C)(C)C)C(=O)NC(C(C)C)C(=O)OC | Protease |
| CHEMBL41063 | CC[C@H](C)[C@H](NC(=O)[C@@H](CC(O)C[C@@H](Cc1ccccc1)C(=O)N[C@@H]([C@@H](C)CC)C(=O)NCCN2CCOCC2)Cc3ccccc3)C(=O)NCCN4CCOCC4 | Protease |
| CHEMBL2092733 | CC[C@H](C)[C@H](NC(=O)[C@H]1CCCN1C[C@@H](O)[C@H](Cc2ccccc2)NC(=O)[C@H](CC(=O)N)NC(=O)OC(C)(C)C)C(=O)N[C@@H](C(C)C)C(=O)OC | Protease |
| CHEMBL14792 | Cc1cccc(c1)C2=CC(=C(SCc3ccccc3)C(=O)O2)O | Protease |
| CHEMBL318387 | CC(C)[C@H](NC(=O)c1sc(C)nc1C)C(=O)N[C@@H](Cc2ccccc2)[C@@H](O)[C@H](O)[C@H](Cc3ccccc3)NC(=O)[C@@H](NC(=O)c4sc(C)nc4C)C(C)C | Protease |
| CHEMBL290872 | CC[C@H](C)[C@H](NC(=O)[C@@H](CC(O)CN1C[C@H]2CCCC[C@H]2C[C@H]1C(=O)NC(C)(C)C)Cc3ccccc3)C(=O)OC | Protease |
| CHEMBL139346 | COc1ccc(cc1)S(=O)(=O)N(CC(C)C)C[C@@H](O)[C@H](Cc2ccccc2)NC(=O)c3ccccc3O | Protease |
| CHEMBL304369 | CC(C)(C)NC(=O)[C@@H]1CCCN1C[C@@H](O)[C@@H]2Cc3ccc(OCCCCOc4cc5ccccc5cc4C(=O)N[C@@H](CC(=O)N)C(=O)N2)cc3 | Protease |
| CHEMBL48175 | CC(C)OC(=O)N[C@@H](Cc1ccccc1)[C@H](O)CNC[C@@H](O)[C@H](Cc2ccccc2)NC(=O)OC(C)C | Protease |
| CHEMBL48921 | CC(C)(C)OC(=O)N[C@@H](Cc1ccccc1)[C@H](O)CNC[C@@H](O)[C@H](Cc2ccccc2)NC(=O)[C@](C)(O)C(C)(C)C | Protease |
| CHEMBL11424 | CC(C)(C)NC(=O)c1ccccc1C[C@@H](O)[C@H](Cc2ccccc2)NC(=O)[C@H](CSc3ccccn3)NS(=O)(=O)C | Protease |
| CHEMBL99015 | CCCCNC(=O)N(CC(C)C)C[C@@H](O)[C@H](Cc1ccccc1)NC(=O)[C@H](CC(=O)N)NC(=O)OCc2ccccc2 | Protease |
| CHEMBL131640 | CCCCN(C[C@@H](O)[C@H](Cc1ccccc1)NC(=O)[C@H](CC(=O)N)NC(=O)OCc2ccccc2)C(=O)NCC(C)C | Protease |
| CHEMBL68155 | CCCCNC(=O)N(CC(C)C)CC(O)[C@H](Cc1ccccc1)NC(=O)[C@H](CC(=O)N)NC(=O)OCc2ccccc2 | Protease |
| CHEMBL275161 | COP(=O)(CNC(=O)[C@@H](NC(=O)[C@H](Cc1cccc2ccccc12)CS(=O)(=O)C(C)(C)C)C(C)C)CNC(=O)[C@@H](NC(=O)[C@H](Cc3cccc4ccccc34)CS(=O)(=O)C(C)(C)C)C(C)C | Protease |
| CHEMBL73804 | CC(C)(C)NC(=O)[C@@H]1CN(Cc2ccccn2)CCN1C[C@@H](O)[C@H](Cc3ccccc3)NC(=O)O[C@H]4CCOC4 | Protease |
| CHEMBL217577 | CCC(C)[C@@H](NC(=O)C(CCc1ccccc1)NC(=O)CCCCCCCCCCCCCCC(=O)N[C@H](CC(=O)N[C@@H](Cc2ccccc2)C(=O)O)C(=O)N)C(=O)N[C@@H](Cc3ccccc3)C(=O)N | Protease |
| CHEMBL14828 | COc1cccc(c1)C2=CC(=C(SCc3ccccc3)C(=O)O2)O | Protease |
| CHEMBL117095 | CC(C)(C)NC(=O)[C@@H]1C[C@H](Cl)CN1C(=O)[C@@H](O)[C@H](Cc2ccccc2)NC(=O)c3ccccc3O | Protease |
| CHEMBL443822 | CCCC1(CCC)CC(=O)C(=C(O)O1)C(c2ccccc2)C(C)(C)C | Protease |
| CHEMBL117638 | CCCC1(CCc2ccccc2)CC(=O)C(=C(O)O1)C(C3CC3)c4cccc(NS(=O)(=O)c5cn(C)cn5)c4 | Protease |
| CHEMBL31266 | CC(C)(C)NC(=O)c1ccccc1C[C@@H](O)[C@H](Cc2ccccc2)NC(=O)[C@@H](CC(=O)OCc3ccccc3)NS(=O)(=O)C | Protease |
| CHEMBL311259 | CC(C)[C@H](NC(=O)OC(C)(C)C)C(=O)NC(Cc1ccc(OCc2ccccc2)cc1)C(=O)C(F)(F)C(=O)NCc3ccccc3 | Protease |
| CHEMBL31510 | CC(=O)N[C@H](CSCc1ccccc1)C(=O)N[C@@H](Cc2ccccc2)[C@H](O)Cc3ccccc3C(=O)NC(C)(C)C | Protease |
| CHEMBL289572 | CC(C)CNC(=O)[C@@H](CC(O)C[C@@H](Cc1ccccc1)C(=O)NCC(C)C)Cc2ccccc2 | Protease |
| CHEMBL441894 | CC(C)(C)NC(=O)c1ccccc1C[C@@H](O)[C@H](Cc2ccccc2)NC(=O)[C@H](CC(=O)OCc3ccccc3)NC(=O)OC(C)(C)C | Protease |
| CHEMBL20846 | CCCC1(CCc2ccccc2)CC(=O)C(=C(O)O1)C(CC)c3cccc(NS(=O)(=O)c4cccc5cccnc45)c3 | Protease |
| CHEMBL376190 | COC(=O)[C@@H](NC(=O)[C@H](CC(C)C)NC(=O)[C@@H](NC(=O)CCCOc1ccc2ccc(OCCCC(=O)N[C@@H](Cc3ccc(O)cc3)C(=O)N[C@@H](CCC(=O)OC(C)(C)C)C(=O)N[C@@H](CC(C)C)C(=O)N)nc2c1)C(C)C)C(C)C | Protease |
| CHEMBL14934 | COC(=O)c1ccccc1CSC2=C(O)C=C(OC2=O)c3ccccc3 | Protease |
| CHEMBL336000 | OC1=C(C(C=C)c2ccccc2)C(=O)CC3(CCCCC3Cc4ccccc4)O1 | Protease |
| CHEMBL324579 | CCCC1(CCC)CC(=O)C(=C(O)O1)C(C2CC2)c3cccc(NS(=O)(=O)c4cn(C)cn4)c3 | Protease |
| CHEMBL16694 | CCC(C1=C(O)Oc2ccccc2C1=O)c3ccccc3 | Protease |
| CHEMBL2111770 | CCC[C@]1(CCc2ccccc2)CC(=O)C(=C(O)O1)[C@@H](CC)c3cccc(NS(=O)(=O)c4ccc(cn4)C#N)c3 | Protease |
| CHEMBL10890 | CC(C)(C)NC(=O)c1ccccc1C[C@@H](O)[C@H](Cc2ccccc2)NC(=O)[C@@H](N)CC(=O)OCc3ccccc3 | Protease |
| CHEMBL142661 | CCC(C1=C(O)OC2(CCC(=O)CC2)CC1=O)c3ccccc3 | Protease |
| CHEMBL343637 | OC1=C(C(C=C)c2ccccc2)C(=O)CC3(CCCCCC3)O1 | Protease |
| CHEMBL116265 | CCCCCC1(CCCCC)CC(=O)C(=C(O)O1)C(CC)c2cccc(NS(=O)(=O)c3cn(C)cn3)c2 | Protease |
| CHEMBL16982 | NC1C(OCc2ccccc2)OC(CO)C(OCc3ccccc3)C1N=[N+]=[N-] | Protease |
| CHEMBL322033 | COC(=O)N(C)C(C(C)C)C(=O)N[C@@H](Cc1ccccc1)[C@H](O)CN(CC2CCCCC2)NC(=O)C(C(C)C)N(C)C(=O)OC | Protease |
| CHEMBL351680 | Cc1c(cccc1C(=O)N2CCc3ccccc3C2)C(=O)N[C@@H](Cc4ccccc4)[C@H](O)Cc5ccccc5C(=O)NC(C)(C)C | Protease |
| CHEMBL113810 | COc1cccc(C(=O)N[C@@H](Cc2ccccc2)[C@H](O)C(=O)N3C[C@@H](Cl)C[C@H]3C(=O)NC(C)(C)C)c1O | Protease |
| CHEMBL2111771 | CCC[C@@]1(CCc2ccccc2)CC(=O)C(=C(O)O1)[C@@H](CC)c3cccc(NS(=O)(=O)c4cn(C)cn4)c3 | Protease |
| CHEMBL63717 | CC(C)(C)OC(=O)N[C@@H](Cc1ccccc1)C(=O)N2CCC(=O)[C@@H]2Sc3ccccc3 | Protease |
| CHEMBL122233 | CCNC(=O)C(NC(=O)Cc1ccccc1)[C@@H]2N[C@@H](C(=O)NCC(O)CN[C@@H](CC3CCCCC3)C(=O)NC(C)(C)C)C(C)(C)S2 | Protease |
| CHEMBL24442 | CC(=O)N[C@H](CC(=O)OCc1ccccc1)C(=O)N[C@@H](Cc2ccccc2)[C@H](O)Cc3ccccc3C(=O)NC(C)(C)C | Protease |
| CHEMBL104471 | CC(CCC=C(C)C)C1=C(O)C(CCc2ccccc2)(OC1=O)C(=O)NC(C)(C)C | Protease |
| CHEMBL17704 | CC(C)(C)NC(=O)[C@@H]1C[C@@H]2CCCC[C@@H]2CN1C[C@@H](O)[C@H](Cc3ccccc3)NC(=O)OCCS(=O)(=O)C | Protease |
| CHEMBL107758 | COC(=O)N[C@@H](C(C)C)C(=O)N[C@@H](Cc1ccccc1)[C@H](O)CN(CC2CCCCC2)NC(=O)[C@@H](NC(=O)OC)C(C)C | Protease |
| CHEMBL21281 | CCCC1(CCc2ccccc2)CC(=O)C(=C(O)O1)C(CC)c3cccc(NS(=O)(=O)c4ccc(cc4)C#N)c3 | Protease |
| CHEMBL309402 | NC(CC(=O)N)C(=O)N[C@@H](Cc1ccccc1)[C@H](O)CN[C@@H](Cc2ccccc2)C(=O)NC3[C@H](O)Cc4ccccc34 | Protease |
| CHEMBL375250 | COC(=O)[C@@H](NC(=O)[C@H](CC(C)C)NC(=O)[C@@H](NC(=O)CCCOc1ccc2ccc(OCCCC(=O)N[C@@H](Cc3ccc(O)cc3)C(=O)N[C@@H](CCC(=O)OC(C)(C)C)C(=O)N[C@@H](CC(C)C)C(=O)N)cc2c1)C(C)C)C(C)C | Protease |
| CHEMBL348226 | CC(C)(C)OC(=O)N[C@@H](Cc1ccccc1)[C@H](O)CNC[C@@H](O)[C@H](Cc2ccccc2)NC(=O)OC(C)(C)CCCO | Protease |
| CHEMBL421410 | CC1(C)S[C@@H](N[C@H]1C(=O)N[C@H](Cc2ccccc2)[C@H](O)CC(=O)NCCO)[C@H](NC(=O)Cc3ccccc3)C(=O)NCc4ccccc4 | Protease |
| CHEMBL385753 | CCC(C)[C@@H](NC(=O)C(Cc1ccc(F)c(F)c1)NC(=O)CCCCCCCCCCCCCCC(=O)N[C@H](CC(=O)N[C@@H](Cc2ccccc2)C(=O)O)C(=O)N)C(=O)N[C@@H](Cc3ccccc3)C(=O)N | Protease |
| CHEMBL267183 | COC(=O)[C@@H](NC(=O)[C@H](CC(C)C)NC(=O)[C@@H](NC(=O)CCCOc1ccc2ccc(OCCCC(=O)N[C@@H](CCC(=O)OC(C)(C)C)C(=O)N[C@@H](CC(C)C)C(=O)N)cc2c1)C(C)C)C(C)C | Protease |
| CHEMBL12705 | CC(C)(C)NC(=O)[C@@H]1C[C@@H](CCN1C[C@@H](O)[C@H](Cc2ccccc2)NC(=O)COc3ccccc3)OCc4ccncc4 | Protease |
| CHEMBL439833 | CC(C)c1ccccc1SC2=C(O)OC(CCC(=O)N3CCSCC3)(CC2=O)c4ccccc4 | Protease |
| CHEMBL161954 | CC(C)c1ccccc1SC2=C(O)OC(CCC(=O)N3CCNCC3)(CC2=O)c4ccccc4 | Protease |
| CHEMBL2304143 | CC[C@@H](C)[C@H](NC(=O)[C@H](C(C)C)C(O)(S)C(O)[C@H](CC(C)C)NC(=O)[C@H](Cc1c[nH]cn1)NC(=O)[C@H](Cc2ccccc2)NC(=O)OC(C)(C)C)C(=O)NCc3ccccn3 | Protease |
| CHEMBL15182 | OC1=C(SCc2ccccc2)C(=O)C=C(O1)c3cccc(O)c3 | Protease |
| CHEMBL2111673 | C[C@H](C1=C(O)[C@](CCc2ccccc2)(OC1=O)C(=O)NCc3ccc(cc3)C(C)(C)C)c4ccccc4 | Protease |
| CHEMBL339998 | OC(=O)CCC1(CC(=O)C(=C(O)O1)SCCc2ccccc2)c3ccccc3 | Protease |
| CHEMBL78887 | Cn1cnc(c1)S(=O)(=O)Nc2cccc(c2)C(C3CC3)C4=C(O)C=C(OC4=O)C(CC5CC5)CC6CC6 | Protease |
| CHEMBL58998 | O[C@@H](C[C@@H](Cc1ccccc1)C(=O)N[C@@H]2[C@H](O)Cc3ccccc23)Cc4ccccc4 | Protease |
| CHEMBL297829 | CC(C)(C)OC(=O)N[C@@H](Cc1ccccc1)[C@H](O)CNC[C@@H](O)[C@H](Cc2ccccc2)NC(=O)[C@@H](O)C(C)(C)C | Protease |
| CHEMBL326566 | CCCC1(CCc2ccccc2)CC(=O)C(=C(O)O1)C(CC)c3cccc(NS(=O)(=O)c4ccc(F)cc4)c3 | Protease |
| CHEMBL334150 | OCc1ccc(COCC(OCc2ccccc2)C(O)C(O)C(COCc3ccc(CO)cc3)OCc4ccccc4)cc1 | Protease |
| CHEMBL37465 | CC[C@H](C)[C@H](NC(=O)[C@@H](CC(O)C[C@@H](Cc1ccccc1)C(=O)N[C@@H]([C@@H](C)CC)C(=O)NCC2CCc3ccccc3N2)Cc4ccccc4)C(=O)NCC5CCc6ccccc6N5 | Protease |
| CHEMBL14889 | OC1=C(SCc2ccccc2C(=O)OC3CCCCC3)C(=O)C=C(O1)c4ccccc4 | Protease |
| CHEMBL121976 | CC(C)CC1(CC(=O)C(=C(O)O1)SCCc2ccccc2)c3ccccc3 | Protease |
| CHEMBL117856 | CCCC1(CCc2ccccc2)CC(=O)C(=C(O)O1)C(c3cccc(NS(=O)(=O)c4ccc(cc4)C#N)c3)C(C)(C)C | Protease |
| CHEMBL111175 | CC(C)(C)OC(=O)N[C@@H](Cc1ccccc1)[C@@H](O)C[C@]2(Cc3ccccc3)CCCN([C@@H]4[C@H](O)Cc5ccccc45)C2=O | Protease |
| CHEMBL342599 | CC(C)(C)OC(=O)N[C@@H](Cc1ccccc1)[C@@H](O)CC2(Cc3ccccc3)CCCN(C4[C@@H](O)Cc5ccccc45)C2=O | Protease |
| CHEMBL340206 | OCCCCC1(CC(=O)C(=C(O)O1)SCc2ccccc2)c3ccccc3 | Protease |
| CHEMBL353796 | CC(C)c1ccccc1SC2=C(O)OC(CCC(=O)N3CCN(CC3)C(=O)OC(C)(C)C)(CC2=O)c4ccccc4 | Protease |
| CHEMBL1791322 | CC[C@H](C)[C@@H](NC(=O)[C@@H](NC(=O)CCCCCCCCCCCCCCC(=O)N[C@H](CC(=O)N[C@@H](Cc1ccccc1)C(=O)O)C(=O)N)[C@H](C)O)C(=O)NC(CCc2ccccc2)C(=O)N | Protease |
| CHEMBL121837 | NC(=O)CCCC1(CC(=O)C(=C(O)O1)SCCc2ccccc2)c3ccccc3 | Protease |
| CHEMBL325673 | CC(C)[C@@H](NS(=O)(=O)N(C)Cc1ccccn1)C(=O)N[C@@H](Cc2ccccc2)C(O)[C@H](Cc3ccccc3)NC(=O)[C@@H](NS(=O)(=O)N(C)Cc4ccccn4)C(C)C | Protease |
| CHEMBL2021324 | O.CCNC(=O)[C@@H](NC(=O)Cc1ccccc1)[C@@H]2N[C@@H](C(=O)NCCNC(=O)[C@@H]3N[C@H](SC3(C)C)[C@H](NC(=O)Cc4ccccc4)C(=O)NCC)C(C)(C)S2 | Protease |
| CHEMBL2021323 | O.O.CCNC(=O)[C@@H](NC(=O)Cc1ccccc1)[C@@H]2N[C@@H](C(=O)NCCNC(=O)[C@@H]3N[C@H](SC3(C)C)[C@H](NC(=O)Cc4ccccc4)C(=O)NCC)C(C)(C)S2 | Protease |
| CHEMBL122797 | OC1=C(SCCc2ccccc2)C(=O)CC3(CCCc4ccccc34)O1 | Protease |
| CHEMBL280527 | CCNc1cccnc1N2CCN(CC2)C(=O)c3cc4cc(OC)ccc4[nH]3 | Protease |
| CHEMBL12484 | CC(C)c1ccccc1SC2=C(O)C=C(OC2=O)c3ccccc3C | Protease |
| CHEMBL435401 | CC(C)CCC1(CC(=O)C(=C(O)O1)Sc2ccccc2)c3ccccc3 | Protease |
| CHEMBL289282 | CC[C@H](C)[C@H](NC(=O)[C@@H](CC(O)CN1CCC[C@@H]1C(=O)N[C@@H]([C@@H](C)CC)C(=O)OC(C)(C)C)Cc2ccccc2)C(=O)NCc3nc4ccccc4[nH]3 | Protease |
| CHEMBL41955 | CN(CC(O)C[C@@H](Cc1ccccc1)C(=O)N[C@H]2CCCC[C@@H]2NC(=O)c3ccc4ccccc4n3)[C@@H](CC5CCCCC5)C(=O)NC(C)(C)C | Protease |
| CHEMBL375050 | COC(=O)[C@@H](NC(=O)[C@H](CC(C)C)NC(=O)[C@@H](NC(=O)CCCOc1ccc2ccc(OCCCC(=O)N[C@@H](C(C)C)C(=O)N[C@@H](CC(C)C)C(=O)N[C@@H](C(C)C)C(=O)OC)nc2c1)C(C)C)C(C)C | Protease |
| CHEMBL375566 | COC(=O)[C@H](CC(C)C)NC(=O)[C@@H](NC(=O)CCCOc1ccc2ccc(OCCCC(=O)N[C@@H](C(C)C)C(=O)N[C@@H](CC(C)C)C(=O)N[C@@H](C(C)C)C(=O)OC)cc2n1)[C@@H](C)O | Protease |
| CHEMBL327912 | OC1=C(CCCOc2cccc(Cl)c2)C(=O)Oc3ccccc13 | Protease |
| CHEMBL328732 | CCOC(=O)c1cccc(OCCCC2=C(O)Oc3ccccc3C2=O)c1 | Protease |
| CHEMBL115329 | CC(C)CN(C[C@@H](O)[C@H](NC(=O)O[C@@H]1CCOC1)Sc2ccccc2)S(=O)(=O)c3ccc(N)cc3 | Protease |
| CHEMBL1791323 | CC[C@H](C)[C@@H](NC(=O)[C@@H](NC(=O)CCCCCCCCCCCCCCC(=O)N[C@H](CC(=O)N[C@@H](Cc1ccccc1)C(=O)O)C(=O)N)[C@H](C)O)C(=O)N[C@H](Cc2ccc(cc2)C#N)C(=O)N | Protease |
| CHEMBL79063 | CCC(Cc1ccccc1)C2=CC(=C(C(C3CC3)c4cccc(NS(=O)(=O)c5ccccc5)c4)C(=O)O2)O | Protease |
| CHEMBL274291 | CC(C)(C)NC(=O)[C@@H]1C[C@@H](CCN1C[C@@H](O)[C@H](Cc2ccccc2)NC(=O)CCc3ccccc3)OCc4ccncc4 | Protease |
| CHEMBL12797 | COc1ccccc1C2=CC(=C(Sc3ccccc3C(C)C)C(=O)O2)O | Protease |
| CHEMBL306640 | CC(C)(C)OC(=O)N[C@@H](Cc1ccccc1)C2(O)CCN(CC2)[C@@H](Cc3ccccc3)C(=O)N[C@@H]4[C@H](O)Cc5ccccc45 | Protease |
| CHEMBL134127 | CC(C)CNC(=O)N(C)C[C@@H](O)[C@H](Cc1ccccc1)NC(=O)[C@H](CC(=O)N)NC(=O)OCc2ccccc2 | Protease |
| CHEMBL70804 | CNC(=O)N(CC(C)C)CC(O)[C@H](Cc1ccccc1)NC(=O)[C@H](CC(=O)N)NC(=O)OCc2ccccc2 | Protease |
| CHEMBL69781 | CC(C)C(NC(=O)OCc1ccccc1)C(=O)N[C@H](CC(C[C@H](Cc2ccccc2)NC(=O)C(NC(=O)OCc3ccccc3)C(C)C)[N+](=O)[O-])Cc4ccccc4 | Protease |
| CHEMBL122464 | CC(C)(C)NC(=O)[C@@H]1CCCN1CC(O)CNC(=O)[C@@H]2N[C@H](SC2(C)C)C(NC(=O)Cc3ccccc3)C(=O)NCc4ccccc4 | Protease |
| CHEMBL99527 | CNC(=O)N(CC(C)C)C[C@@H](O)[C@H](Cc1ccccc1)NC(=O)[C@H](CC(=O)N)NC(=O)OCc2ccccc2 | Protease |
| CHEMBL346860 | CC(C)(C)OC(=O)N[C@@H](Cc1ccccc1)[C@H](O)CNC[C@@H](O)[C@H](Cc2ccccc2)NC(=O)OC(C)(C)CNC=O | Protease |
| CHEMBL3138187 | CC(C)CCCC(C)[C@H]1CC[C@H]2[C@@H]3CCC4C[C@@H](CCC=C(c5cc(Cl)c(O)c(c5)C(=O)O)c6cc(Cl)c(O)c(c6)C(=O)O)CC[C@]4(C)[C@H]3CC[C@]12C | Protease |
| CHEMBL121722 | OC(C(COCc1cccc(F)c1)OCc2ccccc2)C(O)C(COCc3cccc(F)c3)OCc4ccccc4 | Protease |
| CHEMBL3137943 | CC(CCC=C(c1cc(Cl)c(O)c(c1)C(=O)O)c2cc(Cl)c(O)c(c2)C(=O)O)[C@H]3CC[C@H]4[C@@H]5CC[C@@H]6CCCC[C@]6(C)[C@H]5CC[C@]34C | Protease |
| CHEMBL115743 | CCCC1(CCc2ccccc2)CC(=O)C(=C(O)O1)C(CC)c3cccc(NS(=O)(=O)c4ccccc4)c3 | Protease |
| CHEMBL330840 | OC(C(COCc1ccc(F)cc1)OCc2ccc(F)cc2)C(O)C(COCc3ccc(F)cc3)OCc4ccc(F)cc4 | Protease |
| CHEMBL82443 | CCCCCCCCCCCCCCCC(=O)Nc1ccc(c2cccc(c12)S(=O)(=O)O)S(=O)(=O)O | Protease |
| CHEMBL76486 | CCC(Cc1ccccc1)C2=CC(=C(C(C3CC3)c4cccc(NS(=O)(=O)c5ccc(cc5)C#N)c4)C(=O)O2)O | Protease |
| CHEMBL2068409 | CC(C)C[C@H](NC(=O)[C@@H](NC(=O)[C@H](C)NC(=O)CNC(=O)CSCC1CCN2CCC(CO[Si](c3ccccc3)(c4ccccc4)C(C)(C)C)N=C2N1)[C@@H](C)OCc5ccccc5)C(=O)N[C@@H](CC(=O)N)C(=O)N[C@@H](Cc6ccccc6)C(=O)OCc7ccccc7 | Protease |
| CHEMBL122261 | CC(C)(C)NC(=O)[C@@H]1C[C@H](CN1CC(O)CNC(=O)[C@@H]2N[C@H](SC2(C)C)C(NC(=O)Cc3ccccc3)C(=O)NCc4ccccc4)OC(C)(C)C | Protease |
| CHEMBL370393 | CCCCC(NC(=O)CCCCCCCCCCCCCCC(=O)N[C@H](CC(=O)N[C@@H](Cc1ccccc1)C(=O)O)C(=O)N)C(=O)N[C@H](C(C)CC)C(=O)N[C@@H](Cc2ccccc2)C(=O)N | Protease |
| CHEMBL428519 | C[C@H]1C[C@H](CS1(=O)=O)OC(=O)N[C@@H](Cc2ccccc2)[C@H](O)CN3C[C@H]4CCCC[C@H]4C[C@H]3C(=O)NC(C)(C)C | Protease |
| CHEMBL291732 | O[C@@H](C[C@@H](Cc1ccccc1)C(=O)N[C@@H]2[C@H](O)Cc3ccccc23)Cc4ccccc4O | Protease |
| CHEMBL274011 | COc1ccc(cc1)C2=CC(=C(SCc3ccccc3)C(=O)O2)O | Protease |
| CHEMBL62905 | CCCCN(C)C(=O)Oc1nsnc1c2c(Cl)cccc2Cl | Protease |
| CHEMBL293914 | OC1=C(C(C2=C(O)Oc3ccccc3C2=O)c4ccc(cc4)C(C5=C(O)Oc6ccccc6C5=O)C7=C(O)Oc8ccccc8C7=O)C(=O)c9ccccc9O1 | Protease |
| CHEMBL14930 | OC1=C(SCc2ccccc2)C(=O)C=C(O1)c3ccccc3 | Protease |
| CHEMBL89444 | COc1ccccc1CCCCC2=C(O)Oc3ccccc3C2=O | Protease |
| CHEMBL222213 | CC[C@H](C)[C@H](NC(=O)CCCOc1ccc2ccc(OCCCC(=O)N[C@@H](C(C)C)C(=O)N[C@@H](CC(C)C)C(=O)N[C@@H](C(C)C)C(=O)OC)cc2n1)C(=O)N[C@@H]([C@@H](C)O)C(=O)N | Protease |
| CHEMBL1791324 | CC[C@H](C)[C@@H](NC(=O)[C@@H](NC(=O)CCCCCCCCCCCCCCC(=O)N[C@H](CC(=O)N[C@@H](Cc1ccccc1)C(=O)O)C(=O)N)[C@H](C)O)C(=O)N[C@H](Cc2ccc(Br)cc2)C(=O)N | Protease |
| CHEMBL18086 | CC(C)(C)NC(=O)[C@@H]1C[C@@H]2CCCC[C@@H]2CN1C[C@@H](O)[C@H](Cc3ccccc3)NC(=O)O[C@H]4CCCS(=O)(=O)C4 | Protease |
| CHEMBL65504 | CC1(C)CC2(CC(C)(C)c3c(Br)c(O)c(O)c(Br)c23)c4c(Br)c(O)c(O)c(Br)c14 | Protease |
| CHEMBL267184 | COC(=O)[C@@H](NC(=O)[C@H](CC(C)C)NC(=O)[C@@H](NC(=O)CCCOc1ccc2ccc(OCCCC(=O)N[C@@H](CCC(=O)OC(C)(C)C)C(=O)N[C@@H](CC(C)C)C(=O)N)nc2c1)C(C)C)C(C)C | Protease |
| CHEMBL116798 | CCC(C1=C(O)OC(CC(C)C)(CC(C)C)CC1=O)c2cccc(NS(=O)(=O)c3cn(C)cn3)c2 | Protease |
| CHEMBL430629 | CCC(Cc1ccccc1)C2=CC(=C(C(C3CC3)c4cccc(NS(=O)(=O)c5ccc(OC)cc5)c4)C(=O)O2)O | Protease |
| CHEMBL332043 | CCC(C1=C(O)OC(CCC2CC2)(CCC3CC3)CC1=O)c4cccc(NS(=O)(=O)c5cn(C)cn5)c4 | Protease |
| CHEMBL120214 | OC(C(COCc1ccccc1)OCc2cccc(F)c2)C(O)C(COCc3ccccc3)OCc4cccc(F)c4 | Protease |
| CHEMBL319872 | CC1(C)S[C@@H](N[C@H]1C(=O)N[C@H](Cc2ccccc2)[C@@H](O)CC(=O)N)[C@H](NC(=O)Cc3ccccc3)C(=O)NCc4ccccc4 | Protease |
| CHEMBL2068549 | COC(=O)[C@H](Cc1ccccc1)NC(=O)[C@H](CC(=O)N)NC(=O)[C@H](CC(C)C)NC(=O)[C@@H](NC(=O)[C@H](C)NC(=O)CCCCC(=O)OC[C@@H]2CCN3CC[C@@H](CO[Si](c4ccccc4)(c5ccccc5)C(C)(C)C)N=C3N2)[C@@H](C)O | Protease |
| CHEMBL48996 | CCCC[C@@H](CN[C@@H](CCCC)C(=O)N[C@@H](CCC(=O)N)C(=O)N[C@@H](CCCNC(=N)N)C(=O)N)NC(=O)[C@@H](NC(=O)[C@@H](NC(=O)C)[C@@H](C)O)[C@@H](C)CC | Protease |
| CHEMBL12622 | CCOC(=O)c1cccc(c1)C2=CC(=C(Sc3ccccc3C(C)C)C(=O)O2)O | Protease |
| CHEMBL121033 | OC(C(COCc1ccccc1)OCc2ccc(Cl)cc2)C(O)C(COCc3ccccc3)OCc4ccc(Cl)cc4 | Protease |
| CHEMBL327985 | CC(C)[C@@H](NC(=O)N1CCOCC1)C(=O)NN(C[C@H](O)[C@H](Cc2ccccc2)NC(=O)[C@@H](NC(=O)N3CCOCC3)C(C)C)Cc4ccccc4 | Protease |
| CHEMBL194238 | CC(C)C[C@H](C[C@H](O)[C@@H](Cc1cccc(Br)c1)NC(=O)COc2c(C)cccc2C)S(=O)(=O)c3ccc(F)cc3 | Protease |
| CHEMBL38501 | CCCC[C@H](CC(O)(CO)C[C@@H](CCCC)C(=O)N[C@@H](CC(C)C)C(=O)N[C@@H](Cc1ccccc1)C(=O)OC)C(=O)N[C@@H](CC(C)C)C(=O)N[C@@H](Cc2ccccc2)C(=O)OC | Protease |
| CHEMBL78454 | CCC(Cc1ccccc1)C2=CC(=C(C(C3CC3)c4cccc(NS(=O)(=O)c5ccc(Cl)cc5)c4)C(=O)O2)O | Protease |
| CHEMBL324640 | CC(C1=C(O)C(CCc2ccccc2)(OC1=O)C(=O)NC(C)(C)C)c3ccccc3 | Protease |
| CHEMBL278823 | CC(C)[C@@H](COCc1ccccc1)NC(=O)C(F)(F)C(=O)C(Cc2ccc(OCc3ccccc3)cc2)NC(=O)[C@H](NC(=O)OCc4ccccc4)C(C)C | Protease |
| CHEMBL412382 | COC(=O)[C@@H](NC(=O)[C@H](CC(C)C)NC(=O)[C@@H](NC(=O)CCCOc1ccc2ccc(OCCCC(=O)N[C@@H](C(C)C)C(=O)N[C@@H](CC(C)C)C(=O)N[C@@H](C(C)C)C(=O)OC)cc2c1)C(C)C)C(C)C | Protease |
| CHEMBL331919 | COC(=O)c1ccc(COCC(OCc2ccccc2)C(O)C(O)C(COCc3ccc(cc3)C(=O)OC)OCc4ccccc4)cc1 | Protease |
| CHEMBL2062136 | OC1=C(C(C2CC2)c3cccc(NS(=O)(=O)c4ccc(cc4)C#N)c3)C(=O)OC5=C1CCCCCC5 | Protease |
| CHEMBL292736 | [K+].[K+].[K+].[K+].CC1(C)CC2(CC(C)(C)c3cc(OS(=O)(=O)[O-])c(OS(=O)(=O)[O-])cc23)c4cc(OS(=O)(=O)[O-])c(OS(=O)(=O)[O-])cc14 | Protease |
| CHEMBL1790830 | CC[C@H](C)[C@H](NC(=O)[C@H](CCC(=O)N)NC(=O)[C@@H]1CCCN1C(=O)CCCCCCCCCCCCCCC(=O)N[C@H](CO)C(=O)N[C@H]([C@H](C)O)C(=O)N[C@H](CC(C)C)C(=O)N[C@H](CC(=O)N)C(=O)N[C@H](Cc2ccccc2)C(=O)O)C(=O)N[C@@H]([C@@H](C)O)C(=O)N[C@@H](CC(C)C)C(=O)N[C@@H](Cc3c[nH]c4ccccc34)C(=O)O | Protease |
| CHEMBL15246 | CCc1cccc(c1)C2=CC(=C(SCc3ccccc3)C(=O)O2)O | Protease |
| CHEMBL18603 | CC(C)(C)NC(=O)[C@@H]1C[C@@H]2CCCC[C@@H]2CN1C[C@@H](O)[C@H](Cc3ccccc3)NC(=O)O[C@@H]4CCCS(=O)(=O)C4 | Protease |
| CHEMBL120694 | OC(C(COCc1ccccc1)OCc2ccc(Br)cc2)C(O)C(COCc3ccccc3)OCc4ccc(Br)cc4 | Protease |
| CHEMBL332603 | OC1=C(SCCc2ccccc2)C(=O)CC(O1)c3ccccc3 | Protease |
| CHEMBL195816 | CCC(C)[C@@H](NC(=O)[C@H](C(C)O)N(C)C(=O)CCCCCCCCCCCCCCC(=O)NC(Cc1ccc(OP(=O)(O)O)cc1)C(=O)N[C@@H](Cc2ccccc2)C(=O)O)C(=O)NC(CO)C(=O)N | Protease |
| CHEMBL193943 | CCC(C)[C@@H](NC(=O)C(NC(=O)CCCCCCCCCCCCCCC(=O)N[C@H](CC(=O)N[C@@H](Cc1ccccc1)C(=O)O)C(=O)N)c2ccccc2)C(=O)N[C@@H](Cc3ccccc3)C(=O)N | Protease |
| CHEMBL441952 | OC1=C(SCc2ccccc2)C(=O)C=C(O1)c3cccc(OC(F)(F)F)c3 | Protease |
| CHEMBL80882 | OC1=C(C(C2CC2)c3cccc(NS(=O)(=O)c4ccc(F)cc4)c3)C(=O)C=C(O1)C(CC5CC5)CC6CC6 | Protease |
| CHEMBL123243 | CC(C)(C)NC(=O)[C@@H]1CCCCN1C[C@H](O)CNC(=O)[C@@H]2N[C@H](SC2(C)C)C(NC(=O)Cc3ccccc3)C(=O)NCc4ccccc4 | Protease |
| CHEMBL80494 | OC1=C(C(C2CC2)c3cccc(NS(=O)(=O)c4ccc(cc4)C#N)c3)C(=O)C=C(O1)C(CC5CC5)CC6CC6 | Protease |
| CHEMBL123370 | CN(CC1(CC(=O)C(=C(O)O1)SCCc2ccccc2)c3ccccc3)c4ccccc4 | Protease |
| CHEMBL343642 | Clc1ccc2[nH]c(C3=NCCCN3)c(c2c1)S(=O)(=O)c4ccccc4 | Protease |
| CHEMBL88443 | OC1=C(CCCOc2ccccc2)C(=O)Oc3ccccc13 | Protease |
| CHEMBL334126 | OC1=C(SCc2ccccc2)C(=O)CC3(CCCc4ccccc34)O1 | Protease |
| CHEMBL78822 | CCC(Cc1ccccc1)C2=CC(=C(C(C3CC3)c4cccc(NS(=O)(=O)c5cccc6cccnc56)c4)C(=O)O2)O | Protease |
| CHEMBL119906 | CCCC1(CCc2ccccc2)CC(=O)C(=C(O)O1)C(c3cccc(NS(=O)(=O)c4ccc(cn4)[N+](=O)[O-])c3)C(C)(C)C | Protease |
| CHEMBL314852 | OCc1cccc(OCCCC2=C(O)Oc3ccccc3C2=O)c1 | Protease |
| CHEMBL311570 | OC1=C(C(C2CC2)c3cccc(NS(=O)(=O)c4cccc5cccnc45)c3)C(=O)C=C(O1)C(CC6CC6)CC7CC7 | Protease |
| CHEMBL328046 | Oc1cc(O)c2CC(COc2c1)OC(=O)c3ccc(O)c(O)c3O | Protease |
| CHEMBL144356 | Clc1ccc2[nH]c(c(c2c1)S(=O)(=O)c3ccccc3)n4ccnc4 | Protease |
| CHEMBL308607 | CC(C)[C@H]([C@@H](O)[C@H](O)[C@H](CC1CCCCC1)NC(=O)c2ncccc2OCSc3ccccc3)C(=O)N[C@H]4Cc5ccccc5[C@H]4O | Protease |
| CHEMBL12906 | Cc1cccc(C)c1CCC(=O)N[C@@H](Cc2ccccc2)[C@H](O)CN3CC[C@H](C[C@H]3C(=O)NC(C)(C)C)OCc4ccncc4 | Protease |
| CHEMBL118872 | O[C@H]([C@@H](COCc1ccccc1)OCc2ccccc2)[C@H](O)[C@@H](COCc3ccccc3)OCc4ccccc4 | Protease |
| CHEMBL414635 | CC(C)c1ccccc1SC2=C(O)OC(CCC(=O)N3CCOCC3)(CC2=O)c4ccccc4 | Protease |
| CHEMBL172909 | OC1=C(C(Sc2ccc3ccccc3c2)c4ccccc4)C(=O)C=C(O1)c5ccccc5 | Protease |
| CHEMBL286404 | COC(=O)c1[nH]c2ccccc2c1Sc3ccccc3 | Protease |
| CHEMBL13075 | Cc1ccccc1CCC(=O)N[C@@H](Cc2ccccc2)[C@H](O)CN3CC[C@H](C[C@H]3C(=O)NC(C)(C)C)OCc4ccncc4 | Protease |
| CHEMBL45627 | C\C=C\C\C=C\CCC(=O)[C@H]1O[C@H]1C(=O)N | Protease |
| CHEMBL331664 | OC(C(COCc1ccc(F)cc1)OCc2ccccc2)C(O)C(COCc3ccc(F)cc3)OCc4ccccc4 | Protease |
| CHEMBL14707 | OC1=C(SCc2ccccc2)C(=O)C=C(O1)c3ccccc3Cl | Protease |
| CHEMBL78357 | CCC(Cc1ccccc1)C2=CC(=C(C(C3CC3)c4cccc(NS(=O)(=O)c5cccnc5)c4)C(=O)O2)O | Protease |
| CHEMBL120167 | OC(C(COCc1ccccc1)OCc2ccc(F)cc2F)C(O)C(COCc3ccccc3)OCc4ccc(F)cc4F | Protease |
| CHEMBL59548 | OC(=O)c1ccc2ccc(\C=C\c3ccc(O)c(c3)C(=O)O)nc2c1O | Protease |
| CHEMBL3084791 | CC(C)[C@H](NC(=O)OCc1ccccc1)C(=O)N[C@@H](Cc2ccccc2)CC3(C[C@H](Cc4ccccc4)NC(=O)[C@@H](NC(=O)OCc5ccccc5)C(C)C)CCC(=O)N3 | Protease |
| CHEMBL317881 | CC(C)CNC(=O)C[C@H](O)[C@@H](Cc1ccccc1)NC(=O)[C@@H]2N[C@H](SC2(C)C)[C@H](NC(=O)Cc3ccccc3)C(=O)NCc4ccccc4 | Protease |
| CHEMBL422928 | CC(C)(C)OC(=O)N[C@@H](Cc1ccccc1)[C@@H](O)C[C@@H](Cc2ccccc2)C(=O)NCCc3ccccc3 | Protease |
| CHEMBL376450 | COC(=O)[C@@H](NC(=O)[C@H](CC(C)C)NC(=O)[C@@H](NC(=O)CCCOc1ccc2ccc(OCCCC(=O)N[C@@H](CCC(=O)O)C(=O)N[C@@H](CC(C)C)C(=O)N)cc2c1)C(C)C)C(C)C | Protease |
| CHEMBL388957 | CC[C@H](C)[C@H](NC(=O)[C@H](CCC(=O)N)NC(=O)CCCOc1ccc2ccc(OCCCC(=O)N[C@@H](C(C)C)C(=O)N[C@@H](CC(C)C)C(=O)N[C@@H](C(C)C)C(=O)OC)cc2c1)C(=O)N[C@@H]([C@@H](C)O)C(=O)N | Protease |
| CHEMBL2113708 | O.CC(C)(C)OC(=O)N[C@H](CCNC[C@@H](O)[C@H](Cc1ccccc1)NC(=O)OC(C)(C)C)Cc2ccccc2 | Protease |
| CHEMBL316974 | CC1(C)S[C@@H](N[C@H]1C(=O)N[C@H](Cc2ccccc2)[C@@H](O)CC(=O)NCc3ccccc3)[C@H](NC(=O)Cc4ccccc4)C(=O)NCc5ccccc5 | Protease |
| CHEMBL110385 | CC(C)(C)OC(=O)N[C@@H](Cc1ccccc1)[C@@H](O)CCC(=O)N[C@@H]2[C@H](O)Cc3ccccc23 | Protease |
| CHEMBL319192 | CC(C)(C)OC(=O)N[C@@H](Cc1ccccc1)[C@@H](O)CCC(=O)N[C@H]2[C@H](O)Cc3ccccc23 | Protease |
| CHEMBL136086 | CC(C)(C)OC(=O)N[C@@H](Cc1ccccc1)[C@@H](O)CCC(=O)NC2[C@@H](O)Cc3ccccc23 | Protease |
| CHEMBL369273 | COC(=O)[C@@H](NC(=O)[C@H](CC(C)C)NC(=O)[C@@H](NC(=O)CCCOc1cccc(OCCCC(=O)N[C@@H](C(C)C)C(=O)N[C@@H](CC(C)C)C(=O)N[C@@H](C(C)C)C(=O)OC)n1)C(C)C)C(C)C | Protease |
| CHEMBL310487 | OC1=C(Sc2ccccc2)C(=O)C=C(O1)c3ccccc3 | Protease |
| CHEMBL344838 | CCCCC1(Cc2ccccc2)CC(=O)C(=C(O)O1)C(CC)c3ccccc3 | Protease |
| CHEMBL82338 | CC(C)(C)NC(=O)[C@@H]1C[C@@H]2CCCC[C@@H]2CN1C[C@@H](O)[C@H](Cc3ccccc3)NC(=O)OC(C)(C)C | Protease |
| CHEMBL48281 | CCCCCCCCCCCCCCC[C@H](C[C@H](O)[C@H](Cc1ccccc1)NC(=O)OC(C)(C)C)C(=O)N[C@@H]2[C@H](O)Cc3ccccc23 | Protease |
| CHEMBL356524 | OC1=C(C(C=C)c2ccccc2)C(=O)CC3(CCCC3)O1 | Protease |
| CHEMBL22164 | CCC(Cc1ccccc1)C2=CC(=C(C(CC)c3ccccc3)C(=O)O2)O | Protease |
| CHEMBL105320 | COCCOCCC(CCOCCOC)C1=CC(=C(C(C2CC2)c3cccc(NS(=O)(=O)c4cn(C)cn4)c3)C(=O)O1)O | Protease |
| CHEMBL121243 | Cc1ccc(COCC(OCc2ccccc2)C(O)C(O)C(COCc3ccc(C)cc3)OCc4ccccc4)cc1 | Protease |
| CHEMBL439641 | CC(=O)N[C@H]1CCCC[C@@H]1NC(=O)[C@@H](CC(O)C[C@@H](Cc2ccccc2)C(=O)N[C@H]3CCCC[C@@H]3NC(=O)C)Cc4ccccc4 | Protease |
| CHEMBL78470 | CC1=CC(=C(C(C2CC2)c3cccc(NS(=O)(=O)c4cn(C)cn4)c3)C(=O)O1)O | Protease |
| CHEMBL79687 | OC1=C(Cc2ccc(Cl)cc2)C(=O)C(=O)c3ccccc13 | Protease |
| CHEMBL78958 | Cn1cnc(c1)S(=O)(=O)Nc2cccc(c2)C(C3CC3)C4=C(O)C=C(CCc5ccccc5)OC4=O | Protease |
| CHEMBL316216 | CC(C)(C)OC(=O)N[C@@H](Cc1ccccc1)[C@H](O)CN2C[C@H]3CCCC[C@H]3C[C@H]2C(=O)NC4C(O)Cc5ccccc45 | Protease |
| CHEMBL9409 | CC(C)(C)OC(=O)N[C@@H](Cc1ccccc1)C(O)[C@H](Cc2ccccc2)NC(=O)OC(C)(C)C | Protease |
| CHEMBL310123 | CCC(CC)C1=CC(=C(C(C2CC2)c3cccc(NS(=O)(=O)c4cn(C)cn4)c3)C(=O)O1)O | Protease |
| CHEMBL432552 | COCCOCCCC1=CC(=C(C(C2CC2)c3cccc(NS(=O)(=O)c4cn(C)cn4)c3)C(=O)O1)O | Protease |
| CHEMBL141176 | CCC(C1=C(O)OC2(CCCCCCC2)CC1=O)c3ccccc3 | Protease |
| CHEMBL1161303 | CC(C)CCC[C@@H](C)[C@H]1CCC2C3CC=C4C[C@@H](CC[C@]4(C)C3CC[C@]12C)SC[C@@H]5CCN6CC[C@@H](CSCC(=O)NCC(=O)N[C@@H](C)C(=O)N[C@@H]([C@@H](C)OCc7ccccc7)C(=O)N[C@@H](CC(C)C)C(=O)N[C@@H](CC(=O)N)C(=O)N[C@@H](Cc8ccccc8)C(=O)OCc9ccccc9)NC6=N5 | Protease |
| CHEMBL305436 | O[C@@H](CC[C@H](Cc1ccccc1)NC(=O)c2ccccc2NS(=O)(=O)c3ccccn3)[C@H](Cc4ccccc4)NC(=O)c5ccccc5NS(=O)(=O)c6ccccn6 | Protease |
| CHEMBL104253 | CC(C)(C)NC(=O)C1(CCc2ccccc2)OC(=C(CCc3ccccc3)C1=O)O | Protease |
| CHEMBL82770 | CCCC1=CC(=C(C(C2CC2)c3cccc(NS(=O)(=O)c4cn(C)cn4)c3)C(=O)O1)O | Protease |
| CHEMBL110144 | CC(C)(C)NC(=O)[C@@H]1C[C@@H]2CCCC[C@@H]2CN1CC(O)[C@H](Cc3ccccc3)NC(=O)OC(C)(C)C | Protease |
| CHEMBL335676 | CC(C)(C)NC(=O)[C@@H]1CCCN1C(=O)[C@H](O)[C@H](Cc2ccccc2)NC(=O)[C@H](CC(=O)N)NC(=O)OCc3ccccc3 | Protease |
| CHEMBL97544 | CC(C)C[C@@H](NC(=O)[C@@H](C[C@H](O)[C@H](Cc1ccccc1)NC(=O)OC(C)(C)C)Cc2ccccc2)C(=O)N | Protease |
| CHEMBL2111803 | O.CC(C)(C)NC(=O)[C@@H]1CCCN1CC(O)CNC(=O)[C@@H]2N[C@H](SC2(C)C)C(NC(=O)Cc3ccccc3)C(=O)NCc4ccccc4 | Protease |
| CHEMBL121035 | OC(C(COCc1ccc(cc1)C#N)OCc2ccc(cc2)C#N)C(O)C(COCc3ccc(cc3)C#N)OCc4ccc(cc4)C#N | Protease |
| CHEMBL1791325 | CC[C@H](C)[C@@H](NC(=O)[C@@H](NC(=O)CCCCCCCCCCCCCCC(=O)N[C@H](CC(=O)N[C@@H](Cc1ccccc1)C(=O)O)C(=O)N)[C@H](C)O)C(=O)N2Cc3[nH]c4ccccc4c3CC2C(=O)N | Protease |
| CHEMBL62901 | CC1(C)CC2(CC(C)(C)c3cc(I)c(O)cc23)c4cc(O)c(I)cc14 | Protease |
| CHEMBL154798 | CC(C)CCN(C[C@@H](O)[C@H](Cc1ccccc1)NC(=O)OCc2ccccc2)S(=O)(=O)C | Protease |
| CHEMBL311950 | CCCCCCCCCCCCCCCC(=O)Nc1c(O)cc(c2ccccc12)S(=O)(=O)O | Protease |
| CHEMBL2068526 | CC[C@H](C)[C@H](NC(=O)CNC(=O)CSCC1CCN2CCC(CO[Si](c3ccccc3)(c4ccccc4)C(C)(C)C)N=C2N1)C(=O)N[C@@H](COCc5ccccc5)C(=O)N[C@@H](Cc6ccc(OCc7ccccc7)cc6)C(=O)N[C@@H](CC(=O)N)C(=O)N[C@@H](CC(C)C)C(=O)OC | Protease |
| CHEMBL92492 | COc1ccc2C(=C(CCCCc3ccccc3OC)C(=O)Oc2c1)O | Protease |
| CHEMBL430447 | OS(=O)(=O)c1ccc2c(OC(=O)c3ccc(cc3)c4ccc(cc4)C(=O)Oc5cc(cc6cc(ccc56)S(=O)(=O)O)S(=O)(=O)O)cc(cc2c1)S(=O)(=O)O | Protease |
| CHEMBL48147 | O[C@H](CNC[C@@H](O)[C@H](Cc1ccccc1)NC(=O)OCc2ccccc2)[C@H](Cc3ccccc3)NC(=O)OCc4ccccc4 | Protease |
| CHEMBL40148 | OC(=O)c1ccc2ccc(\C=C\c3ccccc3)nc2c1O | Protease |
| CHEMBL14342 | OC1=C(SCc2ccc3OCCOc3c2)C(=O)C=C(O1)c4ccccc4 | Protease |
| CHEMBL309533 | CCCS(=O)(=O)Nc1cccc(c1)C(C2CC2)C3=C(O)C=C(OC3=O)C(CC)Cc4ccccc4 | Protease |
| CHEMBL1791326 | CC[C@H](C)[C@@H](NC(=O)[C@@H](NC(=O)CCCCCCCCCCCCCCC(=O)N[C@H](CC(=O)N[C@@H](Cc1ccccc1)C(=O)O)C(=O)N)[C@H](C)O)C(=O)N[C@@H](Cc2ccc(N)cc2)C(=O)N | Protease |
| CHEMBL120092 | OC(C(COCc1ccccc1)OCc2ccccc2F)C(O)C(COCc3ccccc3)OCc4ccccc4F | Protease |
| CHEMBL185130 | CCC(C)[C@H](NC(=O)CCCCCCCCCCCNC(=O)[C@H](Cc1ccc(O)c(c1)[N+](=O)[O-])NC(=O)[C@H](CC2CCCCC2)NC(=O)\C=C\C(=O)O)C(=O)N | Protease |
| CHEMBL120218 | Cc1ccc(COC(COCc2ccccc2)C(O)C(O)C(COCc3ccccc3)OCc4ccc(C)cc4)cc1 | Protease |
| CHEMBL324230 | CC1(C)S[C@@H](N[C@H]1C(=O)N[C@H](Cc2ccccc2)[C@@H](O)CC(=O)Nc3nc4ccccc4[nH]3)[C@H](NC(=O)Cc5ccccc5)C(=O)NCc6ccccc6 | Protease |
| CHEMBL342585 | CC(C)(C)NC(=O)[C@@H]1CCCN1C(=O)[C@H](O)[C@@H]2Cc3ccc(OCCCOc4cc5ccccc5cc4C(=O)N[C@@H](CC(=O)N)C(=O)N2)cc3 | Protease |
| CHEMBL91773 | NC(=O)c1cccc(OCCCC2=C(O)Oc3ccccc3C2=O)c1 | Protease |
| CHEMBL298572 | CC(C)(C)CC(=O)N[C@@H](Cc1ccccc1)[C@H](O)CNC[C@@H](O)[C@H](Cc2ccccc2)NC(=O)OC(C)(C)C | Protease |
| CHEMBL90188 | OC1=C(CCOCc2ccccc2)C(=O)Oc3ccccc13 | Protease |
| CHEMBL69731 | O[C@@H]1[C@@H](O)[C@@H](CCc2ccccc2)N(Cc3ccccc3)C(=O)N(Cc4ccccc4)[C@@H]1CCc5ccccc5 | Protease |
| CHEMBL88511 | OC1=C(CCCOc2cccc(c2)[N+](=O)[O-])C(=O)Oc3ccccc13 | Protease |
| CHEMBL1790828 | CC[C@H](C)[C@H](NC(=O)[C@H](CCC(=O)N)NC(=O)[C@@H]1CCCN1C(=O)CCCCCCCCCCCCCCCCC(=O)N[C@H](CO)C(=O)N[C@H]([C@H](C)O)C(=O)N[C@H](CC(C)C)C(=O)N[C@H](CC(=O)N)C(=O)N[C@H](Cc2ccccc2)C(=O)O)C(=O)N[C@@H]([C@@H](C)O)C(=O)N[C@@H](CC(C)C)C(=O)N[C@@H](Cc3c[nH]c4ccccc34)C(=O)O | Protease |
| CHEMBL375606 | COC(=O)[C@H](CC(C)C)NC(=O)[C@@H](NC(=O)CCCOc1ccc2ccc(OCCCC(=O)N[C@@H](C(C)C)C(=O)N[C@@H](CC(C)C)C(=O)N[C@@H](C(C)C)C(=O)OC)cc2c1)[C@@H](C)O | Protease |
| CHEMBL312027 | OC1=C(C(C2CC2)c3cccc(NS(=O)(=O)c4ccccc4)c3)C(=O)C=C(O1)C(CC5CC5)CC6CC6 | Protease |
| CHEMBL332379 | OC(C(COCc1ccc(F)cc1F)OCc2ccccc2)C(O)C(COCc3ccc(F)cc3F)OCc4ccccc4 | Protease |
| CHEMBL79116 | CCC(CC1CCOCC1)C2=CC(=C(C(C3CC3)c4cccc(NS(=O)(=O)c5cn(C)cn5)c4)C(=O)O2)O | Protease |
| CHEMBL367849 | CC[C@@H](C)[C@H](NC(=O)[C@@H](NC(=O)CCCOc1cccc(OCCCC(=O)N[C@@H](C(C)O)C(=O)N[C@@H]([C@H](C)CC)C(=O)N[C@@H](C(C)C)C(=O)OC)n1)C(C)O)C(=O)N[C@@H](C(C)C)C(=O)OC | Protease |
| CHEMBL12886 | CC(C)(C)NC(=O)[C@@H]1C[C@@H](CCN1C[C@@H](O)[C@H](Cc2ccccc2)NC(=O)CSc3ccccc3)OCc4ccncc4 | Protease |
| CHEMBL418613 | OC(=O)c1ccc2ccc(\C=C\c3ccc(O)cc3)nc2c1O | Protease |
| CHEMBL121427 | OC(C(COCc1ccc(cc1)C#N)OCc2ccccc2)C(O)C(COCc3ccc(cc3)C#N)OCc4ccccc4 | Protease |
| CHEMBL222971 | CC[C@H](C)[C@H](NC(=O)CCCOc1ccc2ccc(OCCCC(=O)N[C@@H](C(C)C)C(=O)N[C@@H](CC(C)C)C(=O)N[C@@H](C(C)C)C(=O)OC)cc2c1)C(=O)N[C@@H]([C@@H](C)O)C(=O)N | Protease |
| CHEMBL85517 | OS(=O)(=O)c1ccc2c(OS(=O)(=O)c3cccc4c(cccc34)S(=O)(=O)Oc5cc(cc6cc(ccc56)S(=O)(=O)O)S(=O)(=O)O)cc(cc2c1)S(=O)(=O)O | Protease |
| CHEMBL415804 | COC(=O)[C@H](Cc1ccccc1)NC(=O)[C@H](CC(=O)N)NC(=O)[C@H](CC(C)C)NC(=O)[C@@H](NC(=O)CCCOc2ccc3ccc(OCCCC(=O)N[C@@H](C(C)O)C(=O)N[C@@H](CC(C)C)C(=O)N[C@@H](CC(=O)N)C(=O)N[C@@H](Cc4ccccc4)C(=O)OC)cc3c2)C(C)O | Protease |
| CHEMBL1790835 | CC[C@H](C)[C@H](NC(=O)[C@H](CCC(=O)N)NC(=O)[C@@H]1CCCN1C(=O)CCCCCCC\C=C/CCCCCCCC(=O)N[C@H](CO)C(=O)N[C@H]([C@H](C)O)C(=O)N[C@H](CC(C)C)C(=O)N[C@H](CC(=O)N)C(=O)N[C@H](Cc2ccccc2)C(=O)O)C(=O)N[C@@H]([C@@H](C)O)C(=O)N[C@@H](CC(C)C)C(=O)N[C@@H](Cc3c[nH]c4ccccc34)C(=O)O | Protease |
| CHEMBL294938 | CO[C@@H]1OC(CNC(=O)OCc2ccccc2)[C@@H](Oc3ccc(OC)cc3)C(OCc4ccccc4)[C@H]1O | Protease |
| CHEMBL295314 | CC(C)(C)OC(=O)N[C@@H](Cc1ccccc1)[C@H](O)CNC[C@H](O)[C@H](Cc2ccccc2)NC(=O)OC(C)(C)C | Protease |
| CHEMBL46316 | CC[C@@H](C)C(NC(=O)OC(C)(C)C)[C@H](O)CNC[C@@H](O)C(NC(=O)OC(C)(C)C)[C@@H](C)CC | Protease |
| CHEMBL1790139 | CC(C)C[C@H](NC(=O)[C@@H](NC(=O)CCCCCCCCCCCCCCC(=O)N(C)[C@@H](C(=O)N)C(=O)N[C@H](Cc1ccccc1)C(=O)O)[C@H](C)O)C(=O)N[C@@H](Cc2ccccc2)C(=O)N | Protease |
| CHEMBL1790833 | CC[C@H](C)[C@H](NC(=O)[C@H](CCC(=O)N)NC(=O)[C@@H]1CCCN1C(=O)CCCCCCC#CCCCCCCC(=O)N[C@H](CO)C(=O)N[C@H]([C@H](C)O)C(=O)N[C@H](CC(C)C)C(=O)N[C@H](CC(=O)N)C(=O)N[C@H](Cc2ccccc2)C(=O)O)C(=O)N[C@@H]([C@@H](C)O)C(=O)N[C@@H](CC(C)C)C(=O)N[C@@H](Cc3c[nH]c4ccccc34)C(=O)O | Protease |
| CHEMBL161243 | COC(=O)[C@@H](\N=C(/S)\[C@@H](NC(=O)[C@H](Cc1ccccc1)NC(=O)[C@H](Cc2ccccc2)NC(=O)[C@H](C)NC(=O)[C@H](C)NC(=O)OC(C)(C)C)C(C)C)C(C)C | Protease |
| CHEMBL382513 | COc1cc(Br)c(cc1OC)C(=O)N[C@@H](Cc2cccc(Br)c2)[C@H](O)C[C@H](CCc3ccc(Cl)cc3Cl)S(=O)(=O)c4ccc(F)cc4 | Protease |
| CHEMBL14262 | Cc1ccc(CSC2=C(O)C=C(OC2=O)c3ccccc3)cc1 | Protease |
| CHEMBL334057 | CC1(C)S[C@@H](N[C@H]1C(=O)N[C@H](Cc2ccccc2)[C@H](O)CC(=O)NCc3ccccc3)C(NC(=O)Cc4ccccc4)C(=O)NCc5ccccc5 | Protease |
| CHEMBL108174 | CC(C)C[C@H](CO)NC(=O)[C@@H]1N[C@H](SC1(C)C)[C@H](NC(=O)Cc2ccccc2)C(=O)NCc3ccccc3 | Protease |
| CHEMBL2111679 | O.CCCCC(CO)NC(=O)[C@@H]1N[C@H](SC1(C)C)[C@H](NC(=O)Cc2ccccc2)C(=O)NCc3ccccc3 | Protease |
| CHEMBL50563 | CO[C@@H]1OC(CNC(=O)OCc2ccccc2)[C@@H](O)C(OCc3ccccc3)[C@H]1O | Protease |
| CHEMBL106005 | CC(C)(C)OC(=O)CN(C[C@@H](O)[C@H](Cc1ccccc1)NC(=O)OC(C)(C)C)Cc2ccccc2 | Protease |
| CHEMBL105679 | CC(C)[C@H](CO)NC(=O)[C@@H]1N[C@H](SC1(C)C)[C@H](NC(=O)Cc2ccccc2)C(=O)NCc3ccccc3 | Protease |
| CHEMBL2114201 | CC(C)(C)OC(=O)N[C@@H](Cc1ccccc1)[C@@H](O)CN[C@@H](Cc2ccccc2)C(=O)N[C@@H]3[C@H](O)Cc4ccccc34 | Protease |
| CHEMBL419560 | CCNC(=O)[C@@H](NC(=O)c1ccccc1Nc2ccccc2)[C@@H]3N[C@@H](C(=O)NCCNC(=O)[C@@H]4N[C@H](SC4(C)C)[C@H](NC(=O)c5ccccc5Nc6ccccc6)C(=O)NCC)C(C)(C)S3 | Protease |
| CHEMBL302989 | CC(C)[C@H](NC(=O)OCc1ccccc1)C(=O)N[C@@H](Cc2ccccc2)[C@H](O)CNC(=O)C[C@H](C(=O)NC(C)(C)C)C(C)(C)C | Protease |
| CHEMBL50700 | CO[C@@H]1OC2COC(O[C@H]2C(OCc3ccccc3)[C@H]1O)c4ccccc4 | Protease |
| CHEMBL333458 | OC(C(COCc1ccc(cc1)C(F)(F)F)OCc2ccccc2)C(O)C(COCc3ccc(cc3)C(F)(F)F)OCc4ccccc4 | Protease |
| CHEMBL177918 | Cc1cccc(C)c1OCC(=O)N[C@@H](Cc2ccccc2)[C@H](O)C(=O)N(Cc3ccccc3)NC(=O)COc4c(C)cccc4C | Protease |
| CHEMBL268410 | CC1=C(C(COC(=O)C23C[C@@H]4C[C@@H](C[C@@H](C4)C2)C3)OC(=O)C56C[C@@H]7C[C@@H](C[C@@H](C7)C5)C6)c8cc9nc(cc%10[nH]c(cc%11[nH]c(cc1n8)C(=C%11CCC(=O)O)C)c(CCC(=O)O)c%10C)c(C(COC(=O)C%12%13C[C@@H]%14C[C@@H](C[C@@H](C%14)C%12)C%13)OC(=O)C%15%16C[C@@H]%17C[C@@H](C[C@@H](C%17)C%15)C%16)c9C | Protease |
| CHEMBL302877 | O=C(N[C@@H](Cc1ccccc1)C(=O)N2CCC(=O)C2Sc3ccccc3)c4ccc5ccccc5n4 | Protease |
| CHEMBL2112467 | CC(C1=C(O)[C@](CCc2ccccc2)(OC1=O)C(=O)NCc3ccc(cc3)C(C)(C)C)c4ccccc4 | Protease |
| CHEMBL304266 | CC1(C)CC2(CC(C)(C)c3cc(O)c(O)cc23)c4cc(O)c(O)cc14 | Protease |
| CHEMBL2068513 | CC[C@H](C)[C@H](NC(=O)CNC(=O)CSCC1CCN2CCC(CO[Si](c3ccccc3)(c4ccccc4)C(C)(C)C)N=C2N1)C(=O)N[C@@H](CO)C(=O)N[C@@H](Cc5ccc(O)cc5)C(=O)N[C@@H](CC(=O)N)C(=O)N[C@@H](CC(C)C)C(=O)OC | Protease |
| CHEMBL2068515 | CC[C@H](C)[C@H](NC(=O)[C@H](C)NC(=O)CSCC1CCN2CCC(CO[Si](c3ccccc3)(c4ccccc4)C(C)(C)C)N=C2N1)C(=O)N[C@@H]([C@@H](C)OCc5ccccc5)C(=O)N[C@@H](CC(C)C)C(=O)N[C@@H](Cc6c[nH]c7ccccc67)C(=O)OC | Protease |
| CHEMBL120705 | OC(C(COCc1ccc(cc1)C(=O)O)OCc2ccccc2)C(O)C(COCc3ccc(cc3)C(=O)O)OCc4ccccc4 | Protease |
| CHEMBL124434 | COC(=O)[C@@H](NC(=O)[C@H](OCc1ccccc1)[C@H](O)[C@@H](O)[C@@H](OCc2ccccc2)C(=O)N[C@@H](C(C)C)C(=O)OC)C(C)C | Protease |
| CHEMBL274071 | C[C@H](NC(=O)N(C[C@@H](O)[C@H](Cc1ccccc1)NC(=O)[C@H](CC(=O)N)NC(=O)OCc2ccccc2)C(C)(C)C)c3ccccc3 | Protease |
| CHEMBL71012 | CC(N(CC(O)[C@H](Cc1ccccc1)NC(=O)[C@H](CC(=O)N)NC(=O)OCc2ccccc2)C(=O)NC(C)(C)C)c3ccccc3 | Protease |
| CHEMBL376817 | COC(=O)[C@H](CC(=O)N)NC(=O)[C@H](CC(C)C)NC(=O)[C@@H](NC(=O)CCCOc1ccc2ccc(OCCCC(=O)N[C@@H](C(C)C)C(=O)N[C@@H](CC(C)C)C(=O)N[C@@H](C(C)C)C(=O)OC)cc2n1)[C@@H](C)O | Protease |
| CHEMBL125639 | CCCC1(CC(=O)C(=C(O)O1)SCCc2ccccc2)c3ccccc3 | Protease |
| CHEMBL121923 | CC(C)(C)NC(=O)[C@@H]1C[C@@H](O)CN1CC(O)CNC(=O)[C@@H]2N[C@H](SC2(C)C)C(NC(=O)Cc3ccccc3)C(=O)NCc4ccccc4 | Protease |
| CHEMBL607102 | C[C@H](N(C[C@@H](O)[C@H](Cc1ccccc1)NC(=O)[C@H](CC(=O)N)NC(=O)OCc2ccccc2)C(=O)NC(C)(C)C)c3ccccc3 | Protease |
| CHEMBL16796 | OC(C(COCc1ccc(Cl)cc1)OCc2ccccc2)C(O)C(COCc3ccc(Cl)cc3)OCc4ccccc4 | Protease |
| CHEMBL224644 | CC[C@H](C)[C@H](NC(=O)CCCOc1ccc2ccc(OCCCC(=O)N[C@@H](C(C)C)C(=O)N[C@@H](CC(C)C)C(=O)N[C@@H](C(C)C)C(=O)OC)cc2n1)C(=O)N[C@@H]([C@@H](C)O)C(=O)N[C@@H](CC(C)C)C(=O)OC | Protease |
| CHEMBL343163 | CCCC1CCCCCCC2=C1OC(=C(C(C3CC3)c4ccccc4)C2=O)O | Protease |
| CHEMBL278942 | Cc1ccccc1CSC2=C(O)C=C(OC2=O)c3ccccc3 | Protease |
| CHEMBL120466 | OC(C(COCc1ccccc1)OCc2ccc(cc2)C#N)C(O)C(COCc3ccccc3)OCc4ccc(cc4)C#N | Protease |
| CHEMBL449777 | CC(C)C[C@H](NC(=O)[C@@H](NC(=O)CCCCCCCCCCCCCCC(=O)N[C@@H](CC(=O)N)C(=O)N[C@@H](Cc1ccccc1)C(=O)O)[C@H](C)O)C(=O)N[C@@H](Cc2ccccc2)C(=O)N | Protease |
| CHEMBL315189 | OC1=C(CCCN(c2ccccc2)c3ccc(F)cc3)C(=O)Oc4ccccc14 | Protease |
| CHEMBL78955 | CCC(CCOCCOC)C1=CC(=C(C(C2CC2)c3cccc(NS(=O)(=O)c4cn(C)cn4)c3)C(=O)O1)O | Protease |
| CHEMBL1790126 | CC(C)C[C@H](NC(=O)[C@@H](NC(=O)CCCCCCCCCCCCCCC(=O)N[C@@H](C(=O)N)C(=O)N(C)[C@H](Cc1ccccc1)C(=O)O)[C@H](C)O)C(=O)NCCc2ccccc2 | Protease |
| CHEMBL1790133 | CC(C)C[C@H](NC(=O)[C@@H](NC(=O)CCCCCCCCCCCCCCC(=O)N[C@@H](C(=O)N)C(=O)N[C@H](Cc1ccccc1)C(=O)O)[C@H](C)O)C(=O)N[C@@H](Cc2ccccc2)C(=O)N | Protease |
| CHEMBL14620 | OC1=C(SCc2ccccc2)C(=O)C=C(O1)c3ccccc3O | Protease |
| CHEMBL417029 | CC1(C)CC2(CC(C)(C)c3cc(C=O)c(O)cc23)c4cc(O)c(C=O)cc14 | Protease |
| CHEMBL331284 | OC(C(COCc1ccc(Br)cc1)OCc2ccccc2)C(O)C(COCc3ccc(Br)cc3)OCc4ccccc4 | Protease |
| CHEMBL419262 | CC(C)(C)OC(=O)N[C@@H](Cc1ccccc1)[C@@]2(O)CCN(C2)[C@@H](Cc3ccccc3)C(=O)N[C@@H]4[C@H](O)Cc5ccccc45 | Protease |
| CHEMBL49341 | CC(C)(C)COC(=O)N[C@@H](Cc1ccccc1)[C@H](O)CNC[C@@H](O)[C@H](Cc2ccccc2)NC(=O)OCC(C)(C)C | Protease |
| CHEMBL120693 | OC(C(COCc1ccc(cc1)C(F)(F)F)OCc2ccc(cc2)C(F)(F)F)C(O)C(COCc3ccc(cc3)C(F)(F)F)OCc4ccc(cc4)C(F)(F)F | Protease |
| CHEMBL14855 | OC(=O)c1cccc(CSC2=C(O)C=C(OC2=O)c3ccccc3)c1 | Protease |
| CHEMBL1790825 | CC[C@H](C)[C@H](NC(=O)[C@H](CCC(=O)N)NC(=O)[C@@H]1CCCN1C(=O)CCCCCCC\C=C\CCCCCCCC(=O)N[C@H](CO)C(=O)N[C@H]([C@H](C)O)C(=O)N[C@H](CC(C)C)C(=O)N[C@H](CC(=O)N)C(=O)N[C@H](Cc2ccccc2)C(=O)O)C(=O)N[C@@H]([C@@H](C)O)C(=O)N[C@@H](CC(C)C)C(=O)N[C@@H](Cc3c[nH]c4ccccc34)C(=O)O | Protease |
| CHEMBL92138 | Oc1cc2CC(CCOC(=O)c3ccc(O)c(O)c3O)COc2cc1O | Protease |
| CHEMBL331741 | C[C@H](N(CC(O)CNC(=O)[C@@H]1N[C@H](SC1(C)C)C(NC(=O)Cc2ccccc2)C(=O)NCc3ccccc3)CC4CCCCC4)C(=O)NC(C)(C)C | Protease |
| CHEMBL119968 | OC(C(COCc1ccccc1)OCc2c(F)cccc2F)C(O)C(COCc3ccccc3)OCc4c(F)cccc4F | Protease |
| CHEMBL322644 | CC(C)[C@H](NC(=O)C(O)(c1ccccc1)c2ccccc2)C(=O)N[C@@H](Cc3ccccc3)[C@@H](O)[C@H](O)[C@H](Cc4ccccc4)NC(=O)[C@@H](NC(=O)C(O)(c5ccccc5)c6ccccc6)C(C)C | Protease |
| CHEMBL92171 | Nc1cccc(OCCCC2=C(O)Oc3ccccc3C2=O)c1 | Protease |
| CHEMBL607101 | C[C@@H](N(C[C@@H](O)[C@H](Cc1ccccc1)NC(=O)[C@H](CC(=O)N)NC(=O)OCc2ccccc2)C(=O)NC(C)(C)C)c3ccccc3 | Protease |
| CHEMBL25119 | CC(C)(C)OC(=O)[C@H]1CCCN1CC(O)[C@H](Cc2ccccc2)NC(=O)OCc3ccccc3 | Protease |
| CHEMBL48993 | CC(C)(C)OC(=O)N[C@@H](Cc1ccccc1)[C@H](O)CNC[C@@H](O)[C@H](Cc2ccccc2)NC(=O)[C@](C)(O)c3ccccc3 | Protease |
| CHEMBL273621 | CC(C)(C)NC(=O)[C@@H]1CCCCN1C[C@@H](O)CNC(=O)[C@@H]2N[C@H](SC2(C)C)C(NC(=O)Cc3ccccc3)C(=O)NCc4ccccc4 | Protease |
| CHEMBL305767 | C[C@@H](N(CC(O)[C@H](Cc1ccccc1)NC(=O)[C@H](CC(=O)N)NC(=O)OCc2ccccc2)C(=O)NC(C)(C)C)c3ccccc3 | Protease |
| CHEMBL435001 | CC(C)(C)OC(=O)[C@@H]1CCCN1C[C@H](O)[C@H](Cc2ccccc2)NC(=O)OCc3ccccc3 | Protease |
| CHEMBL134292 | C[C@@H](NC(=O)N(C[C@@H](O)[C@H](Cc1ccccc1)NC(=O)[C@H](CC(=O)N)NC(=O)OCc2ccccc2)C(C)(C)C)c3ccccc3 | Protease |
| CHEMBL323225 | CC(C)(C)NC(=O)[C@@H]1C[C@@H]2CCCC[C@@H]2CN1CC(O)CN3C[C@H]4CCCC[C@H]4C[C@H]3C(=O)NC(C)(C)C | Protease |
| CHEMBL319652 | COC(=O)[C@@H](NC(=O)Cc1ccccc1)[C@@H]2N[C@@H](C(=O)NNC(=O)[C@@H]3N[C@H](SC3(C)C)[C@H](NC(=O)Cc4ccccc4)C(=O)OC)C(C)(C)S2 | Protease |
| CHEMBL96857 | CC(C)(C)NC(=O)c1cc(ccc1CCC(O)Cc2ccc(cc2C(=O)NC(C)(C)C)C(C)(C)C)C(C)(C)C | Protease |
| CHEMBL90889 | OC(=O)c1cccc(OCCCC2=C(O)Oc3ccccc3C2=O)c1 | Protease |
| CHEMBL442513 | CN(C)c1ccc(cc1)C(=O)OCC(OC(=O)c2ccc(cc2)N(C)C)C3=C(C)c4cc5[nH]c(cc6[nH]c(cc7nc(cc3n4)c(C)c7C(COC(=O)c8ccc(cc8)N(C)C)OC(=O)c9ccc(cc9)N(C)C)c(C)c6CCC(=O)O)C(=C5C)CCC(=O)O | Protease |
| CHEMBL261987 | Cc1ccc(COCC(OCc2ccc(C)cc2)C(O)C(O)C(COCc3ccc(C)cc3)OCc4ccc(C)cc4)cc1 | Protease |
| CHEMBL1790832 | CC[C@H](C)[C@H](NC(=O)[C@H](CCC(=O)N)NC(=O)[C@@H]1CCCN1C(=O)CCCCCCCC#CCCCCCCCC(=O)N[C@H](CO)C(=O)N[C@H]([C@H](C)O)C(=O)N[C@H](CC(C)C)C(=O)N[C@H](CC(=O)N)C(=O)N[C@H](Cc2ccccc2)C(=O)O)C(=O)N[C@@H]([C@@H](C)O)C(=O)N[C@@H](CC(C)C)C(=O)N[C@@H](Cc3c[nH]c4ccccc34)C(=O)O | Protease |
| CHEMBL2114200 | CC(C)(C)OC(=O)N[C@@H](Cc1ccccc1)[C@H](O)CN[C@H](Cc2ccccc2)C(=O)N[C@@H]3[C@H](O)Cc4ccccc34 | Protease |
| CHEMBL1791315 | CC[C@H](C)[C@@H](NC(=O)C(NC(=O)CCCCCCCCCCCCCCC(=O)N[C@H](CC(=O)N[C@@H](Cc1ccccc1)C(=O)O)C(=O)N)C(C)(C)C)C(=O)N[C@@H](Cc2ccccc2)C(=O)N | Protease |
| CHEMBL51564 | CO[C@@H]1OC(CNC(=O)C)[C@@H](Oc2ccc(OC)cc2)C(OCc3ccccc3)[C@H]1O | Protease |
| CHEMBL107001 | CC1(C)S[C@H](CNC(=O)Cc2ccccc2)N[C@H]1C(=O)NCCNC(=O)[C@@H]3N[C@@H](CNC(=O)Cc4ccccc4)SC3(C)C | Protease |
| CHEMBL352550 | OC1=C(C(Sc2ccccc2)c3ccc4ccccc4c3)C(=O)C=C(O1)c5ccccc5 | Protease |
| CHEMBL1790973 | CC[C@H](C)[C@H](NC(=O)[C@@H](NC(=O)[C@@H](N)CCC(=O)N)C(C)C)C(=O)N[C@@H](CC(C)C)[C@@H](O)CC(=O)N[C@@H](CCC(=O)N)C(=O)N[C@@H](CO)C(=O)O | Protease |
| CHEMBL1790128 | CC(C)C[C@H](N(C)C(=O)[C@@H](NC(=O)CCCCCCCCCCCCCCC(=O)N[C@@H](C(=O)N)C(=O)N[C@H](Cc1ccccc1)C(=O)O)[C@H](C)O)C(=O)N[C@@H](Cc2ccccc2)C(=O)N | Protease |
| CHEMBL171235 | COC(=O)c1ccc(CN2[C@H](COc3ccccc3)[C@H](O)[C@@H](O)[C@@H](COc4ccccc4)N(Cc5ccc(cc5)C(=O)OC)C2=O)cc1 | Protease |
| CHEMBL67968 | CC(C)[C@H](NC(=O)OCc1ccccc1)C(=O)N[C@@H](Cc2ccccc2)[C@H](O)CNC(=O)CCC(=O)NC(C)(C)C | Protease |
| CHEMBL1790135 | CC(C)C[C@H](NC(=O)[C@H]([C@H](C)O)N(C)C(=O)CCCCCCCCCCCCCCC(=O)N[C@@H](C(=O)N)C(=O)N[C@H](Cc1ccccc1)C(=O)O)C(=O)N[C@@H](Cc2ccccc2)C(=O)N | Protease |
| CHEMBL120747 | COC(=O)c1ccc(COC(COCc2ccccc2)C(O)C(O)C(COCc3ccccc3)OCc4ccc(cc4)C(=O)OC)cc1 | Protease |
| CHEMBL299429 | CN([C@@H](Cc1ccccc1)[C@H](O)CNC[C@@H](O)[C@H](Cc2ccccc2)NC(=O)OC(C)(C)C)C(=O)OC(C)(C)C | Protease |
| CHEMBL81875 | OC1=C(CCCC2CCC3CCCCC3C2)C(=O)C(=O)c4ccccc14 | Protease |
| CHEMBL2115206 | CC(C)(C)OC(=O)N[C@@H](Cc1ccccc1)[C@H](O)CNC(C(O)c2ccccc2)C(=O)N[C@@H]3[C@H](O)Cc4ccccc34 | Protease |
| CHEMBL15001 | Cc1cccc(CSC2=C(O)C=C(OC2=O)c3ccccc3)c1 | Protease |
| CHEMBL326249 | CCNC(=O)[C@@H](NC(=O)Cc1ccccc1)[C@@H]2N[C@@H](C(=O)NCCNC(=O)[C@@H]3NCSC3(C)C)C(C)(C)S2 | Protease |
| CHEMBL124921 | OC1=C(SCc2ccccc2)C(=O)CC(O1)c3ccccc3 | Protease |
| CHEMBL52487 | CC(=O)NCC1O[C@@H](Oc2ccccc2)[C@@H](O)C(O)[C@@H]1O | Protease |
| CHEMBL290349 | CC(C)CN1C[C@@](O)(C[C@H]1C(=O)NC(C)(C)C)[C@H](CC2CCCCC2)NC(=O)CC(C)(C)C | Protease |
| CHEMBL21296 | OC1[C@H](Cc2ccccc2)N(CC3CC3)C(=O)N(CC4CC4)[C@H]1Cc5ccccc5 | Protease |
| CHEMBL1790127 | CC(C)C[C@H](NC(=O)[C@@H](NC(=O)CCCCCCCCCCCCCCC(=O)N[C@@H](C(=O)N)C(=O)N(C)[C@H](Cc1ccccc1)C(=O)O)[C@H](C)O)C(=O)N[C@@H](Cc2ccccc2)C(=O)N | Protease |
| CHEMBL359972 | CCC(C)[C@H](NC(=O)CCCCCCCCCCCNC(=O)[C@H](NC(=O)[C@@H](Cc1ccccc1)NC(=O)\C=C\C(=O)O)C(=O)N)C(=O)N[C@@H](Cc2ccccc2)C(=O)N | Protease |
| CHEMBL99317 | CC(C)CN(C[C@H](O)[C@H](Cc1ccccc1)NC(=O)[C@H](CC(=O)N)NC(=O)c2ccc3ccccc3n2)C(=O)NC(C)(C)C | Protease |
| CHEMBL40710 | COC(=O)[C@@H](NC(=O)[C@@H](NC(=O)[C@@H](CC(=O)C[C@@H](Cc1ccccc1)C(=O)N[C@@H](C(C)C)C(=O)N[C@@H](C(C)C)C(=O)OC)Cc2ccccc2)C(C)C)C(C)C | Protease |
| CHEMBL80362 | COc1ccc2C(=C(C(C3CC3)c4cccc(NS(=O)(=O)c5cccc6cccnc56)c4)C(=O)Oc2c1)O | Protease |
| CHEMBL369126 | [O-][N+](=O)c1ccc2c(c1)C(=O)Oc3ccc(CBr)cc23 | Protease |
| CHEMBL355782 | O[C@@H]1[C@@H](O)[C@@H](CCCc2ccccc2)N(Cc3ccccc3)C(=O)N(Cc4ccccc4)[C@@H]1CCCc5ccccc5 | Protease |
| CHEMBL301382 | CC(C)(C)OC(=O)N[C@@H](Cc1ccccc1)[C@H](O)CNC[C@@H](O)[C@H](Cc2ccccc2)NC(=O)[C@@H](O)c3ccccc3 | Protease |
| CHEMBL48484 | CC(C)(C)OC(=O)N[C@@H](Cc1ccccc1)[C@H](O)COC[C@@H](O)[C@H](Cc2ccccc2)NC(=O)OC(C)(C)C | Protease |
| CHEMBL143086 | CCC(C1=C(O)OC(Cc2ccccc2)(CC1=O)c3ccccc3)c4ccccc4 | Protease |
| CHEMBL291441 | COc1ccc(C[C@H](NC(=O)OC(C)(C)C)C(=O)N2CCC(=O)[C@H]2Sc3ccccc3)cc1 | Protease |
| CHEMBL9735 | CC(=O)N[C@@H](Cc1ccccc1)C(O)[C@H](Cc2ccccc2)NC(=O)C | Protease |
| CHEMBL52153 | CC(C)(C)OC(=O)N[C@@H](Cc1ccccc1)[C@H](O)CNC[C@@H](O)[C@@H](Cc2ccccc2)NC(=O)OC(C)(C)C | Protease |
| CHEMBL80630 | COc1ccc2C(=C(C(C3CC3)c4cccc(NS(=O)(=O)c5ccc(F)cc5)c4)C(=O)Oc2c1)O | Protease |
| CHEMBL442133 | COC(=O)[C@H](Cc1ccccc1)NC(=O)[C@H](CC(=O)N)NC(=O)[C@H](CC(C)C)NC(=O)[C@@H](NC(=O)CCCOc2cccc(OCCCC(=O)N[C@@H](C(C)O)C(=O)N[C@@H](CC(C)C)C(=O)N[C@@H](CC(=O)N)C(=O)N[C@@H](Cc3ccccc3)C(=O)OC)n2)C(C)O | Protease |
| CHEMBL143085 | CCC(C1=C(O)OC(Cc2ccccc2)(Cc3ccccc3)CC1=O)c4ccccc4 | Protease |
| CHEMBL95011 | CC(C)(C)NC(=O)[C@@H]1CCCN1C[C@H](O)CCc2ccccc2C(=O)NC(C)(C)C | Protease |
| CHEMBL51021 | CC(C)(C)OC(=O)N[C@@H](Cc1ccccc1)[C@@H](O)CNCC(O)[C@@H](Cc2ccccc2)NC(=O)OC(C)(C)C | Protease |
| CHEMBL51820 | CC(C)(C)OC(=O)N[C@@H](Cc1ccccc1)[C@H](O)CNC[C@H](O)[C@@H](Cc2ccccc2)NC(=O)OC(C)(C)C | Protease |
| CHEMBL302862 | CC1(C)CC2(CC(C)(C)c3cc(Br)c(O)cc23)c4cc(O)c(Br)cc14 | Protease |
| CHEMBL173001 | COC(=O)CCCOc1ccc2ccc(OCCCC(=O)N[C@@H](C(C)O)C(=O)N[C@@H](CC(C)C)C(=O)N[C@@H](C(C)C)C(=O)N[C@@H](C(C)C)C(=O)OC)cc2c1 | Protease |
| CHEMBL337360 | OC1=C(C(C=C)c2ccccc2)C(=O)CC3(CCOCC3)O1 | Protease |
| CHEMBL419513 | CC(C)(C)NC(=O)[C@@H]1CCCN1C[C@@H](O)CCc2ccccc2C(=O)NC(C)(C)C | Protease |
| CHEMBL62621 | COc1ccc(C[C@H](NC(=O)OC(C)(C)C)C(=O)N2CCC(=O)[C@@H]2Sc3ccccc3)cc1 | Protease |
| CHEMBL121327 | OCc1ccc(COC(COCc2ccccc2)C(O)C(O)C(COCc3ccccc3)OCc4ccc(CO)cc4)cc1 | Protease |
| CHEMBL120261 | OC(C(COCc1ccccc1)OCc2ccc(cc2)C(F)(F)F)C(O)C(COCc3ccccc3)OCc4ccc(cc4)C(F)(F)F | Protease |
| CHEMBL140755 | CC(C)(C)OC(=O)N[C@@H](Cc1ccccc1)[C@H](O)CNC[C@@H](O)[C@@H](N)Cc2ccccc2 | Protease |
| CHEMBL9417 | N[C@@H](Cc1ccccc1)C(O)[C@@H](N)Cc2ccccc2 | Protease |
| CHEMBL80556 | COc1ccc2C(=C(C(C3CC3)c4cccc(NS(=O)(=O)c5ccc(cc5)C#N)c4)C(=O)Oc2c1)O | Protease |
| CHEMBL157450 | CC(C)(C)OC(=O)N[C@H]1CCCC[C@@H]1NC(=O)[C@@H](CC(O)C[C@@H](Cc2ccccc2)C(=O)N[C@H]3CCCC[C@@H]3NC(=O)OC(C)(C)C)Cc4ccccc4 | Protease |
| CHEMBL436088 | O[C@H](C[C@H](CCc1ccc(Cl)cc1Cl)S(=O)(=O)c2ccc(F)cc2)[C@H](Cc3cccc(Br)c3)NC(=O)COc4ccc(Cl)cc4Cl | Protease |
| CHEMBL141252 | CC(C)(C)OC(=O)N[C@@H](Cc1ccccc1)[C@H](O)CNC[C@@H](O)[C@H](Cc2ccccc2)NC(=O)OC(C)(C)CN | Protease |
| CHEMBL49605 | CN(C[C@@H](O)[C@H](Cc1ccccc1)NC(=O)OC(C)(C)C)CC(O)[C@H](Cc2ccccc2)NC(=O)OC(C)(C)C | Protease |
| CHEMBL14482 | COC(=O)c1ccc(CSC2=C(O)C=C(OC2=O)c3ccccc3)cc1 | Protease |
| CHEMBL155260 | CC(C)CCN(C[C@@H](O)[C@H](Cc1ccccc1)NC(=O)OCc2ccccc2)S(=O)(=O)Cc3ccccc3 | Protease |
| CHEMBL290992 | CO[C@H](CNC[C@@H](O)[C@H](Cc1ccccc1)NC(=O)OC(C)(C)C)[C@H](Cc2ccccc2)NC(=O)OC(C)(C)C | Protease |
| CHEMBL51182 | CC(C)(C)OC(=O)N[C@@H](Cc1ccccc1)[C@@H](O)CNC[C@H](O)[C@H](Cc2ccccc2)NC(=O)OC(C)(C)C | Protease |
| CHEMBL80972 | COc1ccc2C(=C(C(C3CC3)c4cccc(NS(=O)(=O)c5cn(C)cn5)c4)C(=O)Oc2c1)O | Protease |
| CHEMBL172440 | CC(C)C[C@H](NC(=O)[C@@H](NC(=O)CCCOc1ccc2ccc(OCCCC(=O)N[C@@H](C(C)O)C(=O)N[C@@H](CC(C)C)C(=O)N[C@@H](C(C)C)C(=O)O)cc2c1)C(C)O)C(=O)N[C@@H](C(C)C)C(=O)O | Protease |
|  |  |  |
| --- | --- | --- |
|  |  |  |

  

---

  
';
